# First profiling of lysine crotonylation of myofilament proteins and ribosomal proteins in zebrafish embryos

**DOI:** 10.1038/s41598-018-22069-3

**Published:** 2018-02-26

**Authors:** Oh Kwang Kwon, Sun Joo Kim, Sangkyu Lee

**Affiliations:** BK21 Plus KNU Multi-Omics based Creative Drug Research Team, College of Pharmacy, Research Institute of Pharmaceutical Sciences, Kyungpook National University, Daegu, 41566 Republic of Korea

## Abstract

Zebrafish embryos are translucent and develop rapidly in individual eggs *ex utero*; they are widely used as models for embryogenesis and organ development for human diseases and drug discovery. Lysine crotonylation (Kcr) is a type of histone post-translational modifications discovered in 2011. Kcr dynamics are involved in gene expression regulation and acute kidney injury; however, little is known about the effects of Kcr on non-histone proteins. In the present study, we conducted the first proteome-wide profiling of Kcr in zebrafish larvae and identified 557 Kcr sites on 218 proteins, representing the Kcr event in zebrafish. We identified two types of Kcr motifs containing hydrophobic (Leu, Ile, Val) and acidic (Asp and Glu) amino acids near the modified lysine residues. Our results show that both crotonylated proteins and sites of crotonylation were evolutionarily conserved between zebrafish embryos and humans. Specifically, Kcr on ribosomal proteins and myofilament proteins, including myosin, tropomyosin and troponin, were widely enriched. Interestingly, 55 lysine crotonylation sites on myosin were distributed throughout coiled coil regions. Therefore, Kcr may regulate muscle contraction and protein synthesis. Our results provide a foundation for future studies on the effects of lysine crotonylation on aging and heart failure.

## Introduction

The zebrafish (*Danio rerio*) is a popular vertebrate model organism in genetic and biological research, such as embryogenesis and organ development and has been used as a model for human diseases and drug discovery^1,2^. The zebrafish genome was sequenced in 2010, revealing evolutionary homology with approximately 70% of the human genome^3^. Zebrafish embryo development is relatively rapid; the embryo assumes its basic body shape 24 h after fertilization and major organs are formed only 5 days after fertilization^4^. Larval zebrafish are commonly used in safety pharmacology and toxicology screening where they are immersed in medium containing a dissolved experimental compound^5^. Although zebrafish have been used as model organisms in a variety of fields, a systemic proteomics approach focusing on protein post-translational modifications (PTMs) has not been reported.

PTMs are dynamic and reversible chemical modifications to proteins that regulate protein functions in various organisms^6^. Covalent binding of small molecules to amino acid residues causes the 3D structure of the protein to change, increasing protein diversity^7^. Among known PTMs, the phosphoproteome in zebrafish embryos has been well-studied; 1067 endogenous phosphorylation sites from 60 embryos were identified in 2008^8^. Moreover, we previously identified 3500 non-redundant phosphorylation sites on 2166 phosphoproteins and quantified 1564 phosphoproteins in developing zebrafish embryos^9^.

Acylation at lysine residues such as formylation, acetylation (Kac), propionylation, butyrylation, malonylation, succinylation, myristoylation, glutarylation and crotonylation (Kcr), plays a crucial role in the functional regulation of many eukaryotic proteins^10,11^. Kcr is a lysine acyl-modification in histones that was discovered in 2011. Kcr modification on histones marked active chromatin and was enriched in promoter and enhancer regions^11^. Recent studies have shown that Kcr is stimulated by intracellular crotonyl-CoA through p300-catalyzed reactions in histones^12^. Sirtuin 3 is a decrotonylase that regulates histone Kcr dynamics and gene transcription in living cells^13^. Histone crotonylation and cellular crotonyl-CoA levels regulate gene expression^12^. Recently, several studies profiled non-histone protein crotonylation in the mammalian cell lines like HeLa, H1299 and A549 cells, respectively, which also showed that Kcr in non-histone proteins is involved in diverse signaling pathways and nuclei-related cellular processes^14–16^. In addition, 637 crotonylated proteins were identified in Nicotiana tabacum, which were implicated in the biosynthesis, folding or degradation of protein^17^.

Myofilament proteins, including myosin, tropomyosin (TM) and troponin, play critical roles in diverse biological functions, such as cell motility, muscle contraction, transcription and intracellular transport. A recent study found that these proteins are closely conserved between unicellular organisms and the origin of multicellular organisms^18^. Particularly, myofilament proteins are significantly modulated by intracellular Ca^2+^, which is required for muscle contraction. It was recently demonstrated that PTMs, such as nitrosylation, phosphorylation and citrullination, of myofilament proteins alter Ca^2+^ sensitivity^19–21^. Alterations of myofilament proteins by PTMs can affect physical ability, leading to aging and diseases such as heart failure^22^. Thus, identifying PTM pathways in myofilament proteins is important for understanding the mechanisms of aging and muscle-associated disease.

Most studies on lysine crotonylation have focused on histone modification in the regulation of epigenetics and transcription factors^23–25^. Here, we studied Kcr of non-histone proteins in zebrafish embryos. We hypothesized that crotonylation plays an important role in signal pathways and other biological functions, similarly to Kac of non-histone proteins. In the present study, we performed a global Kcr analysis in zebrafish larvae using immunoprecipitation and a nano-liquid chromatography (LC)-mass spectrometry (MS)/MS proteomics approach. We identified 557 novel Kcr sites on 218 crotonylated proteins in zebrafish larvae. Our results suggest that both Kcr proteins and sites in zebrafish are evolutionarily conserved in humans. Interestingly, Kcr sites were highly enriched on myofilament proteins, such as myosin, TM and troponin. In addition, many Kcr sites remain to be identified on ribosomal proteins. Our results indicate that Kcr on non-histone proteins regulates muscle contraction and protein synthesis through crotonylated myofilament proteins and ribosomal proteins, respectively.

## Results

### Profiling lysine crotonylation in zebrafish embryos

**Figure 1:**
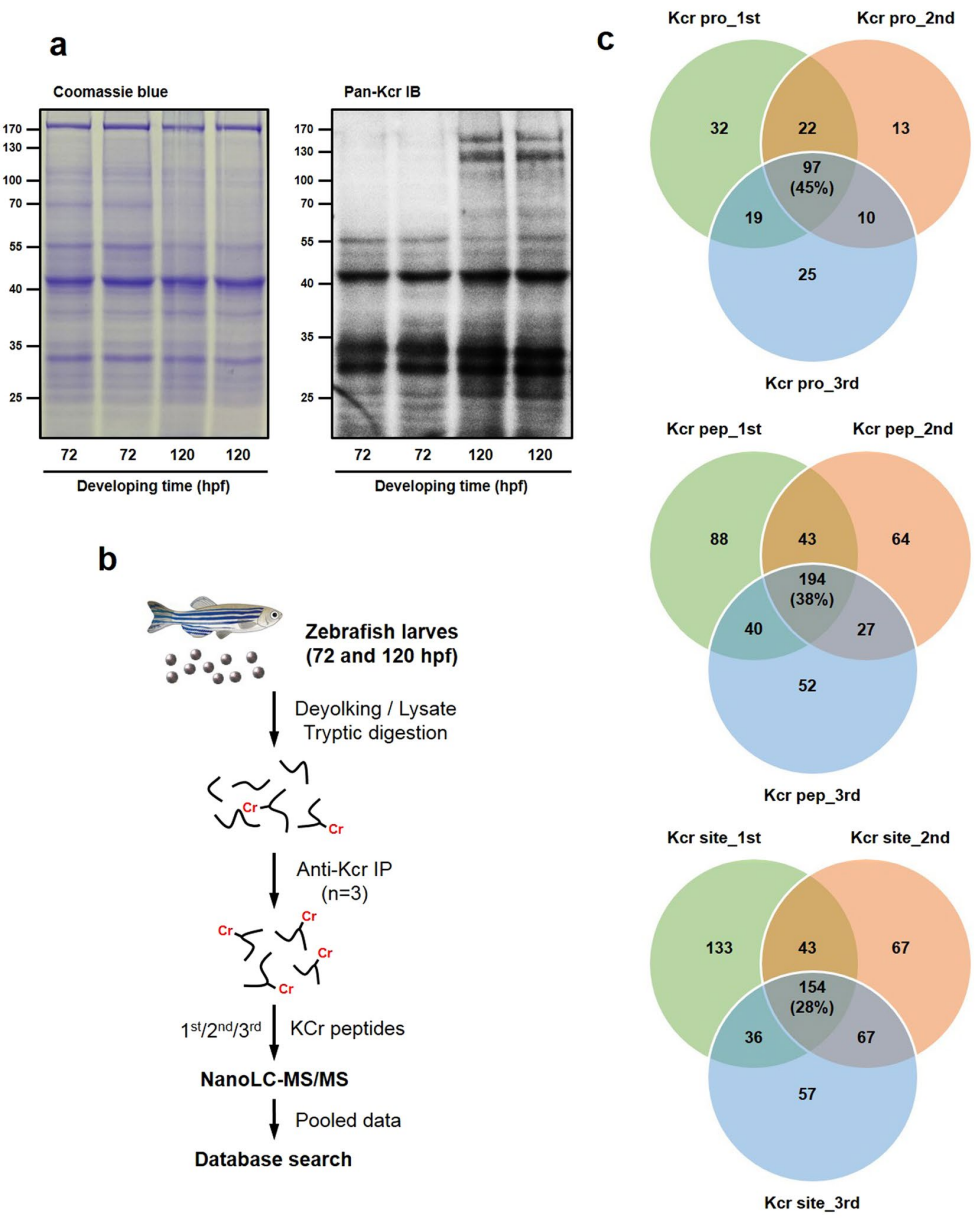
Experimental workflow for global proteomic analysis of lysine-crotonylated proteins. (**a**) Sodium dodecyl sulfate-polyacrylamide gel electrophoresis and crotonyl-lysine immunoblot. Lines represent 72 and 120 hpf, respectively. (**b**) Schematic representation of the sequential steps used for global profiling of lysine crotonylation in zebrafish larvae. (**c**) Overlap of crotonylated sites and proteins in immunoprecipitation experiments performed in triplicate.

We investigated lysine crotonylation (Kcr) modification using larvae at 72–120 h post-fertilization (hpf). This developmental stage was examined because all larvae organs are well-developed at this point. Purified proteins were examined by immunoblot assay with a specific pan-Kcr antibody (Fig. 1a). We detected multiple major protein bands with molecular weights greater than those expected for histones, indicating Kcr modifications on non-histone proteins. To obtain the global crotonylome in zebrafish larvae, proteins were prepared from 72 and 120 hpf larvae. Lysine-crotonylated peptides were immune-enriched with anti-crotonyl lysine antibody-conjugated agarose beads and identified by nano-LC-MS/MS (Fig. 1b). The obtained MS raw data were analyzed using MaxQuant software with the zebrafish database from UniProt (41,001 sequence). MaxQuant results were filtered by MaxQuant scores of more than 40, false discovery rate of less than 1% for both protein and peptide and site localization probability of greater than 0.75. For quality control validation of the MS data, we evaluated the mass error of all identified peptides. The distribution of mass error for precursor ions was close to zero and most values were less than 0.03 Da, indicating acceptable mass accuracy of the MS data (Fig. S1a). All identified Kcr peptides exhibited different abundances depending on their lengths (Fig. S1b). In this study, 557 Kcr sites in 218 proteins were identified in pooled larvae among 508 crotonylated peptides (Table S1). In all detected peptides, 154 Kcr sites, 194 Kcr peptides and 97 Kcr proteins were identified in individual triplicate experiments (Fig. 1c). Among our Kcr results from zebrafish embryos, Kcr proteins and sites converted to human were compared with recent studies to profile non-histone protein crotonylation in HeLa and H1299 cell lines, respectively^14,15^ (Fig. S1c). To compare crotonylation and acetylation in zebrafish, we used a previously acquired Kac dataset in zebrafish^26^ (Fig. S1d). Among the detected Kcr, only 67 (30.7%) Kcr proteins and 52 (9.3%) Kcr sites overlapped with Kac sites.

**Figure 2:**
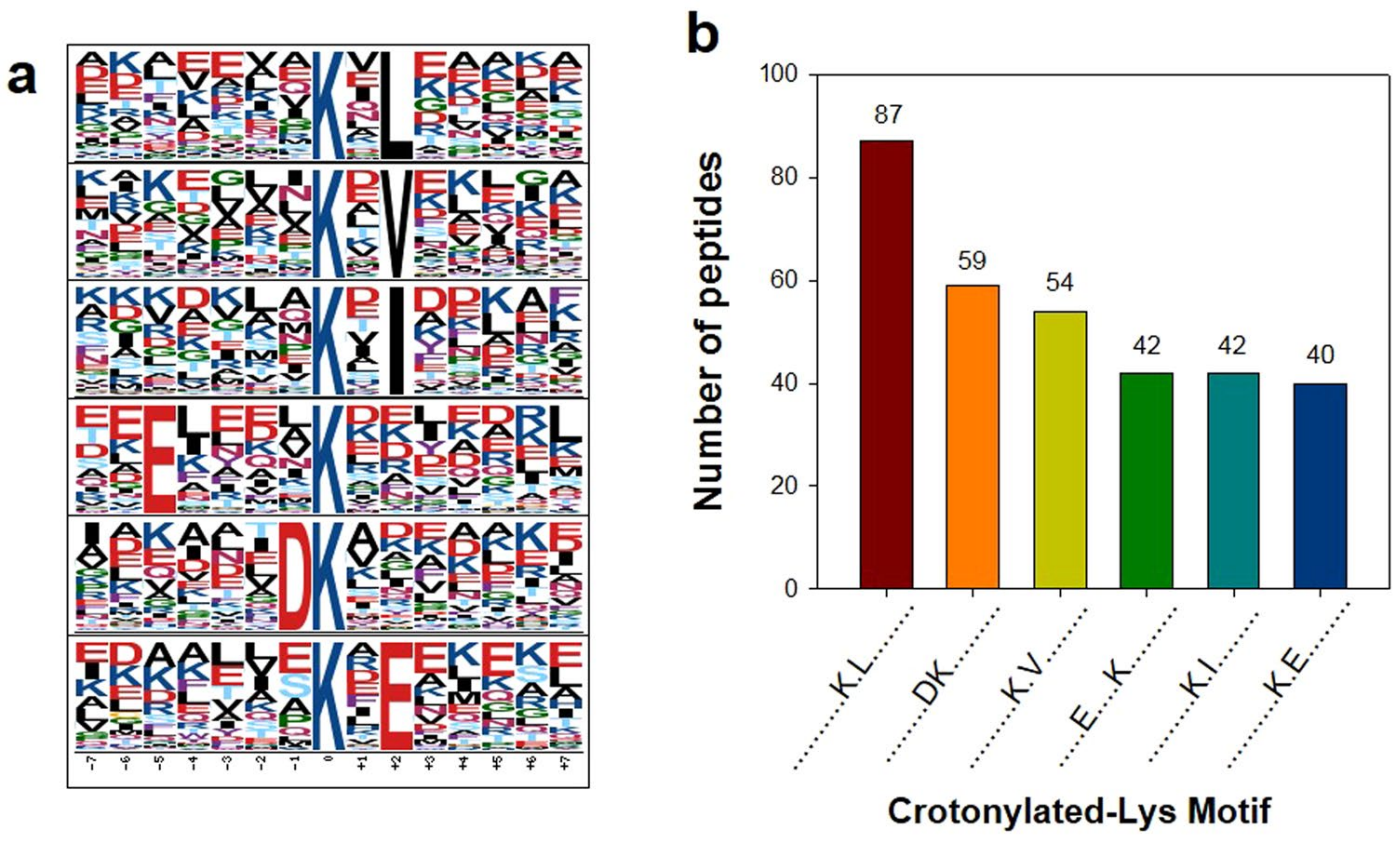
Motif analysis of all identified sites. (**a**) Crotonylation sequence motifs. (**b**) Number of identified peptides containing crotonylated lysines in each motif.

Our data set, including 484 surrounding sequences, was evaluated to identify site-specific sequence motifs from the −7 to the +7 positions surrounding the crotonylated lysine using the Motif-X program^27^. Of all surrounding sequences, 324 sequences were matched to a total of six definitively conserved motifs (Fig. 2a). The six motifs can be divided into two types: the first type includes hydrophobic residues at the +2 position relative to Kcr (Kcr-X-L, Kcr-X-V and Kcr-X-I), while the second type contains acidic residues at the −5, −1 and +2 positions relative to Kcr (E-X-X-X-Kcr, DKcr and Kcr-X-E). Approximately 56.6% of all motif peptides showed hydrophobic amino acid motifs and 43.5% showed acidic amino acid motifs (Fig. 2b). Kcr-X-L was the most common combination, accounting for 26.9% (87) of the motifs in zebrafish larvae.

### Functional enrichment of Kcr by bioinformatics

**Figure 3:**
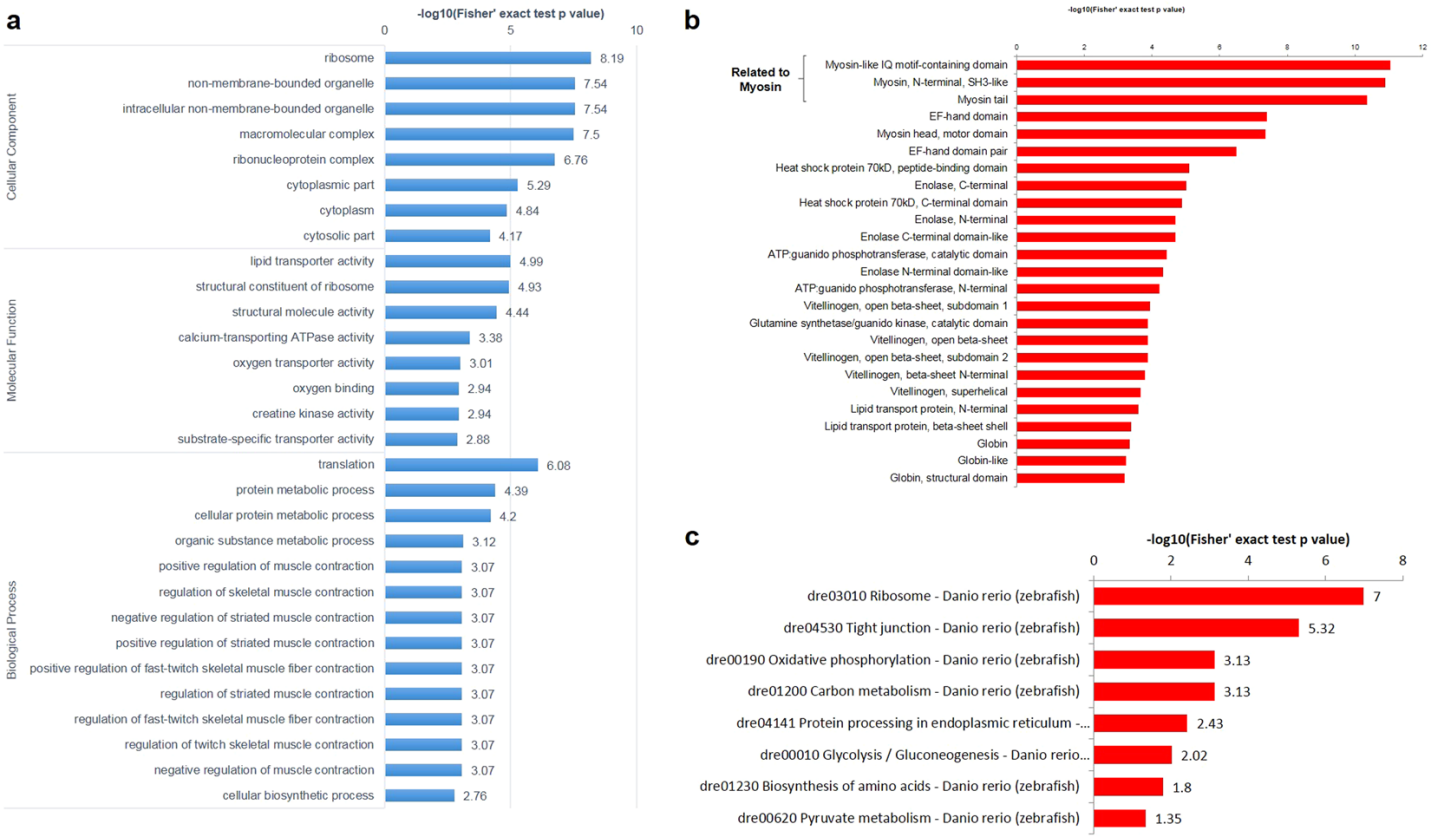
Enrichment analysis of crotonylated protein in zebrafish larvae. (**a**) GO enrichment. (**b**) Domain enrichment. (**c**) KEGG pathway enrichment analysis.

To comprehensively analyze the distribution and function of Kcr non-histone proteins, we performed GO functional annotation and enrichment analysis. Proteins were classified by GO annotation into three categories: biological process, cellular component and molecular function derived from the UniProt-GOA database (http://www.ebi.ac.uk/GOA/). Analyses of Kcr proteins and their subcellular localization are indicated in Fig. S2. Kcr proteins were in cells (37%), organelles (27%) and macromolecular complexes (27%) in cellular components. Molecular function analysis revealed protein functions in binding (47%), catalytic activity (27%), structural molecule activity (13%) and transporter activity (10%). The analysis of biological processes showed that Kcr proteins are primarily involved in cellular processes (28%), metabolic processes (22%) and single organism processes (19%). The identified Kcr proteins were localized in the cytosol (58%), mitochondria (11%), extracellular matrix (8%) and plasma membrane (6%). Figure 3a shows the preferred targets of Kcr proteins by GO enrichment analysis. Ribosomes, non-membrane-bound organelles and macromolecular complexes were significantly enriched among cellular components and lipid transporter activity, structural constituents of ribosomes and structural molecular activity were enriched in molecular function. Translation, metabolic process and diverse regulation of skeletal muscle contraction were strongly enriched in biological processes.

Protein domain enrichment analysis was performed such that domains related to myosin were given top ranks, which refer to GO analyses results (Fig. 3b). Kyoto Encyclopedia of Genes and Genomes (KEGG) pathway enrichment analysis was also performed to identify the metabolic pathways involving crotonylated proteins (Fig. 3c). Kcr occurs on many proteins involved in protein synthesis such as ribosome and protein processing. The cytoskeleton was involved in Kcr modification of tight junctions. Moreover, crotonylated proteins were identified in energy metabolism such as oxidative phosphorylation, carbon metabolism, glycolysis and glycogenesis.

### Evolutionarily conserved Kcr in zebrafish and humans

To analyze the conservation of homologous Kcr between zebrafish and humans, crotonylated proteins were analyzed with BLASTP^28^. We identified 213 (97.7%) Kcr proteins in zebrafish that significantly overlapped with 189 human proteins (Table S2). Moreover, sequences of amino acids upstream and downstream of Kcr sites in zebrafish larvae were analyzed with PSI-BlastP to identify conserved surrounding Kcr sites. We found that 428 zebrafish Kcr sites (76.8%) overlapped with 339 human surrounding sequences. Furthermore, 323 human Kcr sites contained the same lysine residues as 401 Kcr sites (72.0%) in zebrafish. These results indicate that Kcr sites and modifications are highly orthologous between human and zebrafish at the protein level.

Next, to compare potential cross-talk/interplay between Kcr and other PTMs such as Kac and lysine ubiquitination (Kubi), Kcr results were compared with the results in humans using the Kac and Kubi database from PhosphoSitePlus^29^. Of the total 323 Kcr sites converted to humans except for redundant sites, 95 Kac (29.4%) and 42 Kubi (13.0%) sites were detected in the same lysine residue. Kcr is expected to be involved in PTM cross-talk/interplay with competitive Kac rather than with Kubi.

**Figure 4:**
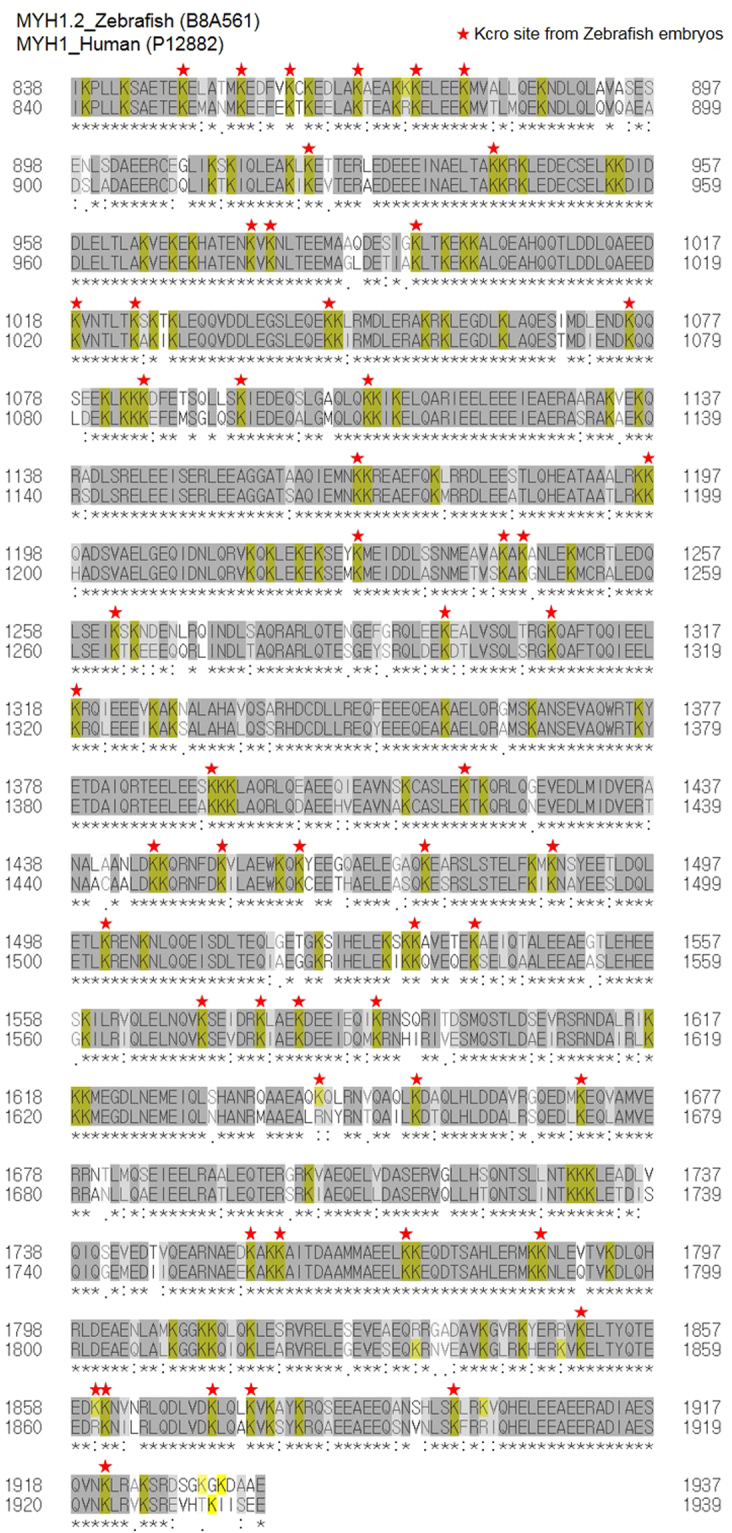
Sequence alignment of crotonylated myosin at coiled coil regions between zebrafish and humans.

According to the results of functional enrichment and BLASTP, Kcr in zebrafish is abundant in ribosomal proteins and myofilament proteins, including myosin, TM and troponin. A summary of crotonylated myofilament and ribosomal proteins among zebrafish and humans is presented in Tables 1 and 2, respectively. We identified 194 crotonylation sites in myofilament proteins in zebrafish larvae, including 156 Kcr sites on myosin and myosin light chain, 22 Kcr sites on TM and 16 troponin Kcr sites, except for duplicated proteins (Table 1). Remarkably, myosin1 was high conserved from zebrafish to human and contained 63 crotonylation sites in zebrafish larvae, accounting for approximately 11% of the total crotonylation sites without overlap between myhz1.1 (B8A568) and 1.2 (B8A561). Interestingly, 55 crotonylation sites in myosin were distributed intensively in the coiled coil motif (amino acids 841–1937) compared to the myosin motor (amino acids 87–780) (Fig. 4). Furthermore, myosin light chain, TM and troponin were largely conserved from zebrafish to humans and contained several crotonylation sites that are also highly similar to site-specific lysine residues in humans (Fig. S3).

**Table 1:** List of crotonylated myofilament proteins in zebrafish embryos.

**Zebrafish**			**Human**				
**UniProt ID**	**gene name**	**# Kcr sites**	**UniProt ID**	**gene name**	**protein name**	**Identities (%)**	**# lysine at similar location**
Q9I8U7	mylz3	4	P14649	MYL6B	Myosin light chain 6B	82.1	1
B8JKH7	mylz3	4					
Q6P0G6	myl1	4					
E9QG51	mylpfb	1	Q96A32	MYLPF	Myosin regulatory light chain 2, skeletal muscle isoform	82.1	5
O93409	mylpfa	5					
F1QJP3	myl10	2	P10916	MYL2	Myosin regulatory light chain 2, ventricular/cardiac muscle isoform	82.1	1
B8A568	myhz1.1	54	P12882	MYH1	Myosin-1	82.1	57
B8A561	myhz1.2	57					
Q6IQX1	myhz2	10					
B8A569	myhz1.3	1					
A2BGX6	myhc4	2					
X1WF87	myhb	1	Q9UKX2	MYH2	Myosin-2	82.1	4
F1QIR4	-	9					
E7FAD0	myhz1.1	1	Q9Y623	MYH4	Myosin-4	82.1	1
F1QJK4	myh9b	1	P35579	MYH9	Myosin-9	82.1	1
E7FBZ3	zgc:171719	1	P09493	TPM1	Tropomyosin alpha-1 chain	82.1	18
P13104	tpma	18					
Q6IQD7	tpm2	2					
Q6P0W3	tpm3	1	P06753	TPM3	Tropomyosin alpha-3 chain	82.1	1
F1QCC0	tnni2b.1	2	P48788	TNNI2	Troponin I, fast skeletal muscle	63.1	3
Q0D2W2	tnni2a.4	5				94.0	
Q6DHP2	tnni2b.2	1				82.1	
Q6IQ92	tnni1al	1	P19237	TNNI1	Troponin I, slow skeletal muscle	82.1	1
E7EXP0	tnnt3b	4	P45378	TNNT3	Troponin T, fast skeletal muscle	98.9	4
Q9I8U9	tnnt3a	3

**Table 2:** List of identified crotonylated ribosomal proteins in zebrafish embryos.

Zebrafish				Human						
Uniprot ID	Gene names	Modified sequence	Position	Uniprot ID	Gene names	Protein names	Identities (%)	Sequence identity	Position	Amino acid
Q7ZV05	rps11	_EAIDGTYIDK(cr)K_	59	P62280	RPS11	40S ribosomal protein S11	91.2	IEGTYIDKKCPFTGN	58	K
Q6PC90	rps12	_EAAK(cr)ALDK_	40	P25398	RPS12	40S ribosomal protein S12	97.0	RGIREAAKALDKRQA	40	K
F8W246	rps13	_FVTGNK(cr)ILR_	70	P62277	RPS13	40S ribosomal protein S13	99.1	VRFVTGNKILRILKS	70	K
Q6PBW7	rps19	_PGGVTVK(cr)DVNQQEFVR_	8	P39019	RPS19	40S ribosomal protein S19	88.3	MPG-VTVKDVNQQEF	7	K
Q6PBW7	rps19	_LK(cr)VPDWVDIVK_	30	P39019	RPS19	40S ribosomal protein S19	88.3	LKKSGKLKVPEWVDT	29	K
Q6PBW7	rps19	_VPDWVDIVK(cr)LAK_	39	P39019	RPS19	40S ribosomal protein S19	88.3	PEWVDTVKLAKHKEL	38	K
E9QDR0	rps2	_IK(cr)SLEEIYLYSLPIK_	62	P15880	RPS2	40S ribosomal protein S2	94.1	LVKDMKIKSLEEIYL	76	K
A8KB78	rps23	_WHDK(cr)QYK_	25	P62266	RPS23	40S ribosomal protein S23	98.6	RDQKWHDKQYKKAHL	25	K
A8KB78	rps23	_ANPFGGASHAK(cr)GIVLEK_	48	P62266	RPS23	40S ribosomal protein S23	98.6	FGGASHAKGIVLEKV	48	K
A8KB78	rps23	_GIVLEK(cr)VGVEAK_	54	P62266	RPS23	40S ribosomal protein S23	98.6	AKGIVLEKVGVEAKQ	54	K
B7ZD32	rps24	_ATVPK(cr)TEIR_	25	P62847	RPS24	40S ribosomal protein S24	89.8	PGKATVPKTEIREKL	37	K
Q6PBI5	rps25	_ATYDK(cr)LYK_	56	P62851	RPS25	40S ribosomal protein S25	91.6	FDKATYDKLCKEVPN	57	K
Q6PBI5	rps25	_AALQELLGK(cr)GLIK_	93	P62851	RPS25	40S ribosomal protein S25	91.6	ALQELLSKGLIKLVS	94	K
Q6PBI5	rps25	_GLIK(cr)LVSK_	97	P62851	RPS25	40S ribosomal protein S25	91.6	LLSKGLIKLVSKHRA	98	K
Q6DHL6	rps6	_LFNLSK(cr)EDDVR_	149	P62753	RPS6	40S ribosomal protein S6	95.6	RKLFNLSKEDDVRQY	149	K
A3KQ06	rps9	_MK(cr)LDYILGLK_	93	P46781	RPS9	40S ribosomal protein S9	95.6	VLDEGKMKLDYILGL	93	K
F8W4I2	rplp0	_GK(cr)AVVLMGK_	50	P05388	RPLP0	60S acidic ribosomal protein P0	96.8	IRMSLRGKAVVLMGK	50	K
Q90Z10	rpl13	_TK(cr)LIIFPR_	123	P26373	RPL13	60S ribosomal protein L13	86.7	RLKEYRSKLILFPRK	123	K
Q90Z10	rpl13	_EAAEQDVEK(cr)K_	209	P26373	RPL13	60S ribosomal protein L13	86.7	AAEQDVEKKK_____	209	K
Q1LYB7	rpl13a	_MVVPAALK(cr)IVR_	127	P40429	RPL13A	60S ribosomal protein L13a	87.9	MVVPAALKVVRLKPT	125	K
Q1LYB7	rpl13a	_NVESK(cr)IAVYTDVLK_	190	P40429	RPL13A	60S ribosomal protein L13a	87.9	AEKNVEKKIDKYTEV	188	K
Q1LYB7	rpl13a	_FNK(cr)VLIIDGR_	7	P40429	RPL13A	60S ribosomal protein L13a	87.9	—	-	-
E9QF69	rpl18	_IQNIPK(cr)LK_	97	Q07020	RPL18	60S ribosomal protein L18	84.8	VRVQEVPKLKVCALR	97	K
E9QF69	rpl18	_SDAPFNK(cr)VILR_	45	Q07020	RPL18	60S ribosomal protein L18	84.8	—	—	—
Q6P5L3	rpl19	_ILMEHIHK(cr)LK_	144	P84098	RPL19	60S ribosomal protein L19	93.9	ILMEHIHKLKADKAR	144	K
Q6P5L3	rpl19	_TLSK(cr)EDETK_	190	P84098	RPL19	60S ribosomal protein L19	94.0	EIIKTLSKEEETKK_	190	K
Q7ZV82	rpl27	_TVVNK(cr)DVFR_	98	P61353	RPL27	60S ribosomal protein L27	95.6	LDKTVVNKDVFRDPA	98	K
Q7ZWJ7	rpl34	_IVVK(cr)VLK_	105	P49207	RPL34	60S ribosomal protein L34	94.0	EEQKIVVKVLKAQAQ	105	K
Q6DGL9	rpl38	_QSLPPGLAVK(cr)ELK_	67	P63173	RPL38	60S ribosomal protein L38	100.0	LPPGLAVKELK____	67	K
Q7ZW95	rpl4	_SEEVQK(cr)AIR_	300	P36578	RPL4	60S ribosomal protein L4	85.3	—	—	—
Q6PBZ1	rpl7a	_AALAK(cr)LVEAIK_	217	P62424	RPL7A	60S ribosomal protein L7a	92.4	EDKGALAKLVEAIRT	217	K
Q6PBZ1	rpl7a	_AK(cr)ELATK_	259	P62424	RPL7A	60S ribosomal protein L7a	92.4	KLEKAKAKELATKLG	259	K
B0R193	ubb	_TITLEVEPSDTIENVK(cr)AK_	27	P62979	RPS27A	Ubiquitin-40S ribosomal protein S27a	97.5	SDTIENVKAKIQDKE	27	K
B0R193	ubb	_LIFAGK(cr)QLEDGR_	48	P62979	RPS27A	Ubiquitin-40S ribosomal protein S27a	97.5	QRLIFAGKQLEDGRT	48	K

Furthermore, 34 lysine sites on 21 ribosomal proteins were crotonylated in zebrafish larvae (Table 2). Crotonylated ribosomal proteins in zebrafish were found to share at least 85% homology and highly conserved site-specific lysine residues with humans. These results demonstrate that Kcr is evolutionarily well-conserved between zebrafish larvae and humans, at both the protein and amino acid levels.

## Discussion

Lysine crotonylation (Kcr) was reported as a new type of PTM in 2011 and serves as a powerful indicator of active cellular genes near histones^11^. Recent studies showed that Kcr levels can be regulated by crotonylating P300 and decrotonylating sirtuin 3^12,13^. Although increased histone crotonylation is related to acute kidney injury due to cell stress and crotonate availability^30^, previous studies on Kcr have focused on the histone regulation of epigenetics. Non-histone Kcr substrates have not been identified. Here, we evaluated Kcr modification of non-histone proteins in zebrafish larvae. We identified and validated 557 Kcr sites on 218 proteins by immunoprecipitation with MS-based proteomics in zebrafish. This is the first large-scale dataset for crotonylation of non-histone proteins in zebrafish larvae. Next, we compared the Kcr results in zebrafish larvae with those of recent Kcr studies in human cell lines. However, Kcr sites did not overlap significantly because 1) our Kcr results from zebrafish embryos and reference results from human cell lines are of different biological status and 2) the Kcr-antibodies used for enrichment may have been different. Thus, lysine crotonylation may function in a variety of species, from zebrafish to human. To investigate PTM crosstalk, we compared Kcr modifications with previously reported acetylation in zebrafish. Only 67 Kcr proteins (30.7%) and 52 Kcr sites (23.9%) overlapped with Kac in zebrafish. These results indicate that Kcr is non-competitive with Kac and engages with biological pathways and interactions that differ from those of Kac sites in zebrafish. However, when analyzing total 323 Kcr sites converted to the human and human Kac, Kubi dataset from PhosphoSitePlus, 95 of the 323 Kac and 42 Kubi were consistent. Based on these results, there may be a mutual relationship between Kcr and other PTMs.

We found six crotonylated motifs in zebrafish larvae that include specific hydrophobic (leucine, valine and isoleucine) or acidic (glutamate and aspartate) amino acid residues flanking the modified lysine residue. The motif results suggest an interaction with specific enzymes, such as kinases and acetylases. For example, acetylation of the KxGS motif can regulate tau assembly by HDAC6 and these sites are hypoacetylated in patients with Alzheimer’s disease^31^. Basophilic motifs such as RxxS are phosphorylated by protein kinase A and proline-directed motifs such as PxS and PxTPP are target sequences for mitogen-activated protein kinase^32^. Therefore, crotonylation-related proteins such as crotonylases and decrotonylases likely prefer hydrophobic or acidic flanking sequences. Because Kcr-X-E was also confirmed in Kac, Kcr-X-E may recognize an enzyme similar to Kac^33^. A recent study of Kcr on non-histone proteins showed that many acidic amino acid motifs such as Kcr-E, E-Kcr and Kcr-D were detected in H1299 and HeLa cells^14^.

Furthermore, we identified only 5 crotonylated sites in histones: H2A K241, H2B K6, H3 K123, H4 K60 and H4 K78. Our bioinformatics results for subcellular localization also suggest that 89% of proteins were crotonylated in the cytosol, mitochondria and extracellular matrix, but not in the nucleus. Therefore, our results indicate that Kcr is distributed in various subcellular locations. To further investigate the biological regulatory effects of Kcr in zebrafish embryos, we carried out GO and KEGG pathway analysis. The results suggest that Kcr serves as a diverse regulatory factor in cellular and metabolic processes.

Additionally, Kcr sites and proteins were evolutionarily conserved between humans and zebrafish. A total of 97.7% of Kcr proteins and 76.8% of Kcr sites in zebrafish significantly overlapped with humans. Our previous study showed that 69% of zebrafish phosphoproteins were conserved in humans^9^. In addition, 51.7% of zebrafish Kac sites overlapped with humans and 34.5% of Kac sites were identified as human Kac sites^26^. Particularly, our dataset revealed that crotonylation of ribosomal proteins and myofilament proteins was highly enriched and evolutionarily conserved. Thus, we focused on myofilament proteins and ribosomal proteins for Kcr.

Numerous studies have examined the correlation between myofilament proteins and PTMs, such as short-term phosphorylation at multiple sites in myosin light chain (MLC), troponin, TM and myosin binding protein-C, which is associated with modulation of contractility^34^. Previous studies on PTMs with myofilaments showed that increased phosphorylation of MLC2 is well-known to increase Ca^2+^ sensitivity^35^. Moreover, Tyr nitration and Cys *S*-nitrosylation of MLC1 is induced by oxidative stress or hypoxia-reoxygenation. As a result, nitrated and *S*-nitrosylated MLC1 may be prone to degradation by matrix metalloprotease-2^19^. Phosphorylation at Thr64 and Ser194 or 195 of human MLC1 is closely related to the stability of the myosin head^36^. Recently, Meishan *et al*. studied the relationship between myosin and PTMs in old age and found that modifications to myosin heavy chain type I and II (MYH1 and 2) in old age are associated with significant slowing of motility speed. They detected eight age-specific myosin PTMs: carbonylation of Pro79, Asn81, Asp900, Asp904 and Arg908; methylation of Glu1166; and deamidation of Gln1164 and Asn1168. Thus, these PTMs may be involved in disordered myosin organization and the slowing of motility^37^.

Other studies on TM and troponin showed that N-terminal acetylation of TM increased protein stability and strongly enhanced affinity to actin^38^. Acetylation enhances TM function, thereby regulating myosin activity^39^. In addition, TM can be phosphorylated by phosphoinositide 3-kinase, which activates myosin Mg^2+^ ATPase and remodeling of the actin cytoskeleton^40–42^. Phosphorylation of troponin I on Ser23 and 24 by protein kinase A has been shown to reduce myofilament Ca^2+^ sensitivity and is associated with heart failure^20^. Troponin T can be phosphorylated by several kinases such as protein kinase C, Ca^2+^/calmodulin-dependent protein kinase II and apoptosis signal-regulating kinase 1^43,44^. Consequently, phosphorylated troponin T at Ser209, 285 and Thr213, 294 by protein kinase C-α reduces tension, ATPase activity and Ca^2+^ sensitivity^45^.

Thus, Kcr of myofilament proteins may play an important role in regulating Ca^2+^ sensitivity, remodeling the actin cytoskeleton and modulating contractility. Furthermore, Kcr is significantly associated with heart failure, myocardial infarction and aging. Notably, Kcr of myosin is concentrated in myosin coiled coils, which are expected to contribute to intracellular transport.

Finally, we identified 34 crotonylated sites on 21 ribosomal proteins that are highly conserved between zebrafish and humans. Ribosomal proteins are among the major sources for protein synthesis and are responsible for translation. Since the 1970s, researchers have predicted that PTMs (such as acetylation) of ribosomal proteins are important for biological functions^46,47^. Recently, some studies revealed that the large ribosomal subunit L28 is substantially ubiquitinated during S phase in yeast and shows active ribosomal function during translation without targeting the protein for degradation^48^. In addition, protein N-terminal acetylation of ribosomal proteins by *N*-acetyltransferase is necessary to maintain protein synthesis in yeast^49^. Therefore, our data also indicate that Kcr of ribosomal proteins is important for the regulation of protein synthesis and ribosome assembly.

In conclusion, we determined the first large-scale crotonylome of zebrafish embryos. These crotonylated proteins and sites are widely distributed in non-histone proteins. Notably, our study revealed that Kcr is evolutionarily conserved between zebrafish and humans and is particularly enriched in ribosomal proteins and myofilament proteins such as myosin, TM and troponin. Therefore, our results provide a foundation for future studies of the effects of crotonylation on aging and heart failure.

## Methods

### Danio rerio cultures

*Danio rerio* (wild-type) were acquired from the Korean Zebrafish Organogenesis Mutant Bank (Daegu, South Korea). Zebrafish were maintained in a 14-h light/10-h dark cycle at 28.5 °C, with a recirculating filtration system using mechanical and biological filtration and fed with baby brine shrimp (Advanced Hatchery Technology, Inc., Salt Lake City, UT, USA) twice daily. The eggs were obtained by pair mating and kept at 28.5 °C in embryo media (0.3 mg/mL sea salt and 1 µg/mL methylene blue). Developmental stages are described as hours post fertilization (hpf) based on morphological features in standard embryogenesis^50^.

### Protein extraction

The eggs were grown until the early larval period after hatching (72–120 hpf) in embryo media (0.3 mg/mL of sea salt and 1 µg/mL of methylene blue) and individually collected 3 times. Manually dechorionated embryos were deyolked by pipetting as previously described and pooled^51^. Deyolked embryos were mixed in lysis buffer containing complete RIPA buffer, protease inhibitor cocktails and histone deacetylase inhibitors and the mixtures were sonicated on ice. The supernatants were separated after centrifugation at 14,000 × *g* for 10 min at 4 °C. For protein purification, embryonic proteins were precipitated in 10% trichloroacetic acid overnight at 4 °C and then centrifuged at 12,000 × *g* for 7 min at 4 °C. Precipitated pellets were washed with −20 °C acetone twice and then dissolved in 50 mM ammonium bicarbonate buffer. Re-suspended proteins were quantified using BCA assay kits.

### In-solution tryptic digestion

Before protein digestion, 10 mM DTT was added to reduce the protein lysates for 1 h at 37 °C and 20 mM iodoacetamide was used for alkylation for 45 min at room temperature (RT) in the dark. The alkylation reaction was quenched by incubation with 30 mM cysteine at RT for an additional 30 min. For trypsin digestion, the lysates were diluted with 100 mM TEAB in urea at a concentration of less than 2 M. Trypsin (Promega, Madison, WI, USA) was added to the solutions at a trypsin-to-protein ratio of 1:50 (w/w) for the first digestion at 37 °C for 16 h and 1:100 trypsin-to-protein mass ratio for a second 4-h digestion to complete the digestion cycle.

### Affinity enrichment for Kcr

To enrich lysine crotonylation (Kcr) peptides, digested peptides were dissolved in NETN buffer (100 mM NaCl, 1 mM EDTA, 50 mM Tris-HCl, 0.5% NP-40, pH 8.0) and incubated with pre-washed pan-crotonylation antibody-conjugated agarose beads (PTM Biolabs, Chicago, IL, USA) with gentle shaking at 4 °C overnight. To remove nonspecific peptides, the beads were washed with NETN buffer 4 times and with water twice. The enriched peptides were eluted with 0.1% trifluoracetic acid from the beads. The eluted peptides were dried with speed vacuum systems. The Kcr enriched peptides were desalted by C18 ZipTips (Millipore, Billerica, MA, USA) according to the manufacturer’s instructions, followed by LC-MS/MS analysis.

### LC-MS/MS analysis

Enriched peptides were dissolved in solvent A (water on 0.1% formic acid) and directly injected into a reversed-phase pre-column (Acclaim PepMap 100, Thermo Scientific, Waltham, MA, USA). Injected peptide samples were separated using a reversed-phase analytical column (Acclaim PepMap RSLC, Thermo Scientific) with gradient of 7–20% solvent B (0.1% formic acid in 98% acetonitrile) for 24 min, 20–35% for 8 min and to 80% for 5 min at a continuous flow rate of 300 nL/min on an EASY-nLC 1000 (Thermo Scientific). The eluted peptides were analyzed with a Q-Exactive Plus hybrid mass spectrometer (Thermo Scientific) with a nano-spray ionization source setting of 2.0 kV.

Entire peptides were detected in an Orbitrap at a resolution of 70,000 and were selected for MS/MS using 30% normalization collision energy. MS/MS samples were identified in the Orbitrap at a resolution of 17,500 with 20 data-dependent mode. MS data were acquired using the following parameters: threshold ion count of 5E3 in the MS survey scan with 15.0 s dynamic exclusion; automatic gain control of 5E4 ions; m/z scan range of 350–1800 for MS scans.

### Database search using MaxQuant

MS/MS data were analyzed using MaxQuant (v.1.4.1.2) against the UniProt *D. rerio* database (41,001 sequences) concatenated with a reverse decoy database. Protein and peptides were acquired using the following parameters: trypsin/P for cleavage enzyme allowing up to 4 missing cleavages; 10 ppm for precursor ions and 0.02 Da for fragment ions of mass error; carbamidomethylation on Cys for fixed modification and oxidation on Met, crotonylation on lysine and acetylation on the protein N-terminus for variable modifications. False discovery rate for protein, peptide and Kcr site were specified at 1%. The minimum peptide length was set to 7. For selected specific Kcr sites, site localization probability was set to >0.75. All other parameters in MaxQuant were used as default.

### Bioinformatics analysis for Gene Ontology annotation

Gene Ontology (GO) is a major bioinformatics initiative to unify the representation of gene and gene product attributes across all species. The GO annotation proteome was determined using the UniProt-GOA database (http://www.ebi.ac.uk/GOA/). Identified protein IDs were converted to UniProt ID and then mapped to GO IDs. If proteins were not annotated in the UniProt-GOA database, InterProScan software was used to annotate the protein’s GO function based on protein sequence alignment. Next, identified proteins were categorized using GO annotation based on three classification: biological process, cellular component and molecular function.

For subcellular localization, we used Wolfpsort, a subcellular localization predication software that predicts subcellular localization. Wolfpsort is an updated version of PSORT/PSORT II for predicting eukaryotic sequences.

To investigate the KEGG pathway, identified proteins annotated by the KEGG database. First, we used the KEGG online service tool KAAS to annotate the protein’s KEGG database. Next, we mapped the annotation results on the KEGG pathway database using the KEGG online service tool KEGG mapper.

### Bioinformatics analysis for enrichment of GO and KEGG pathway analysis

For three GO annotation categories, biological process, cellular component and molecular function, we used the Functional Annotation Tool of DAVID Bioinformatics Resources 6.7 to identify GO enrichments against the background of zebrafish. Additionally, to identify enriched pathways, the KEGG database was used with the Functional Annotation Tool of DAVID against the background of zebrafish. To test the enrichment of protein-containing UniProt entries against all UniProt proteins, we used a two-tailed Fisher’s exact test. Corrections for multiple hypothesis testing were performed using standard false discovery rate control methods. GO terms with a corrected p-value less than 0.05 were regarded as significant. Identified pathways were classified into hierarchical categories according to the KEGG website.

### Motif and homologous analysis

Motif-X software was used to analyze the model of sequences with amino acids in specific positions of modifier-15-mers (7 amino acids upstream and downstream of the site) in all protein sequences. All database protein sequences were used as background database parameters and other parameters were used as default.

To analyze the conservation of Kcr, homologous proteins and sites between zebrafish and humans were examined using BLASTP^28^. The detailed procedure for examining the conservation of proteins and modification sites was previously described^52^. BLASTP parameters for humans were obtained from the UniprotKB database and p-value < 0.001 was considered as high conservation. To analyze potential cross-talk among Kcr, Kac and lysine ubiquitination, Kcro results converted to human were compared using database sets downloaded from PhosphoSitePlus^29^.

### Immunoblot analysis

For SDS polyacrylamide gel electrophoresis, 20 μg of protein from each developmental phase was loaded in 10% gels. Separated gels were transferred onto polyvinylidene difluoride membranes on wetting blot systems and blocked with 5% bovine serum albumin with TBST buffer (20 mM Tris, 137 mM NaCl and 0.5% Tween-20 pH 7.4) for 5 h at RT. Membranes were incubated with anti-Kcr primary antibodies (PTM Biolabs, #PTM-501, 1:1000) overnight at 4 °C. After washing the membranes with TBST five times, they were incubated with anti-rabbit IgG horseradish peroxidase-linked secondary antibody (#7074, 1:2000; Cell Signaling Technology, Danvers, MA, USA) for 2 h at RT. Finally, the membranes were washed with TBST three times and target proteins were detected using ECL reagent with Image Quant LAS-4000 mini (GE Healthcare, Little Chalfont, UK).

## Electronic supplementary material

Supplemental information
