# Supplementary material for: First profiling of lysine crotonylation of myofilament proteins and ribosomal proteins in zebrafish embryos
Source: Sci Rep. 2018 Feb 26;8:3652. doi: 10.1038/s41598-018-22069-3 (PMC5827021; doi:10.1038/s41598-018-22069-3)
Supplement: Supplementary file 1 — Supplemental information [file 41598_2018_22069_MOESM1_ESM.pdf]

Supplemental information

First profiling of lysine crotonylation of myofilament proteins and ribosomal proteins in zebrafish embryos

Oh Kwang Kwon[#], Sun Joo Kim, Sangkyu Lee*

[1]BK21 Plus KNU Multi-Omics based Creative Drug Research Team, College of Pharmacy, Research Institute of Pharmaceutical Sciences, Kyungpook National University, Daegu 41566, Republic of Korea

*Correspondence: Sangkyu Lee, Ph.D., Professor, College of Pharmacy, Research Institute of Pharmaceutical Sciences, Kyungpook National University, 80 Daehak-ro, Buk-gu 41566, Republic of Korea. Tel.: +82-53-950 8571; Fax: +82-53-950-8557; E-mail: sangkyu@knu.ac.kr.

Supplementary Table Legends

Supplemental Table S1. List of all identified lysine crotonlyation in zebrafish embryos.

Supplemental Table S2. Crotonylated protein and sites homology between Zebrafish and human by BLASTP.

Supplementary Figure Legends

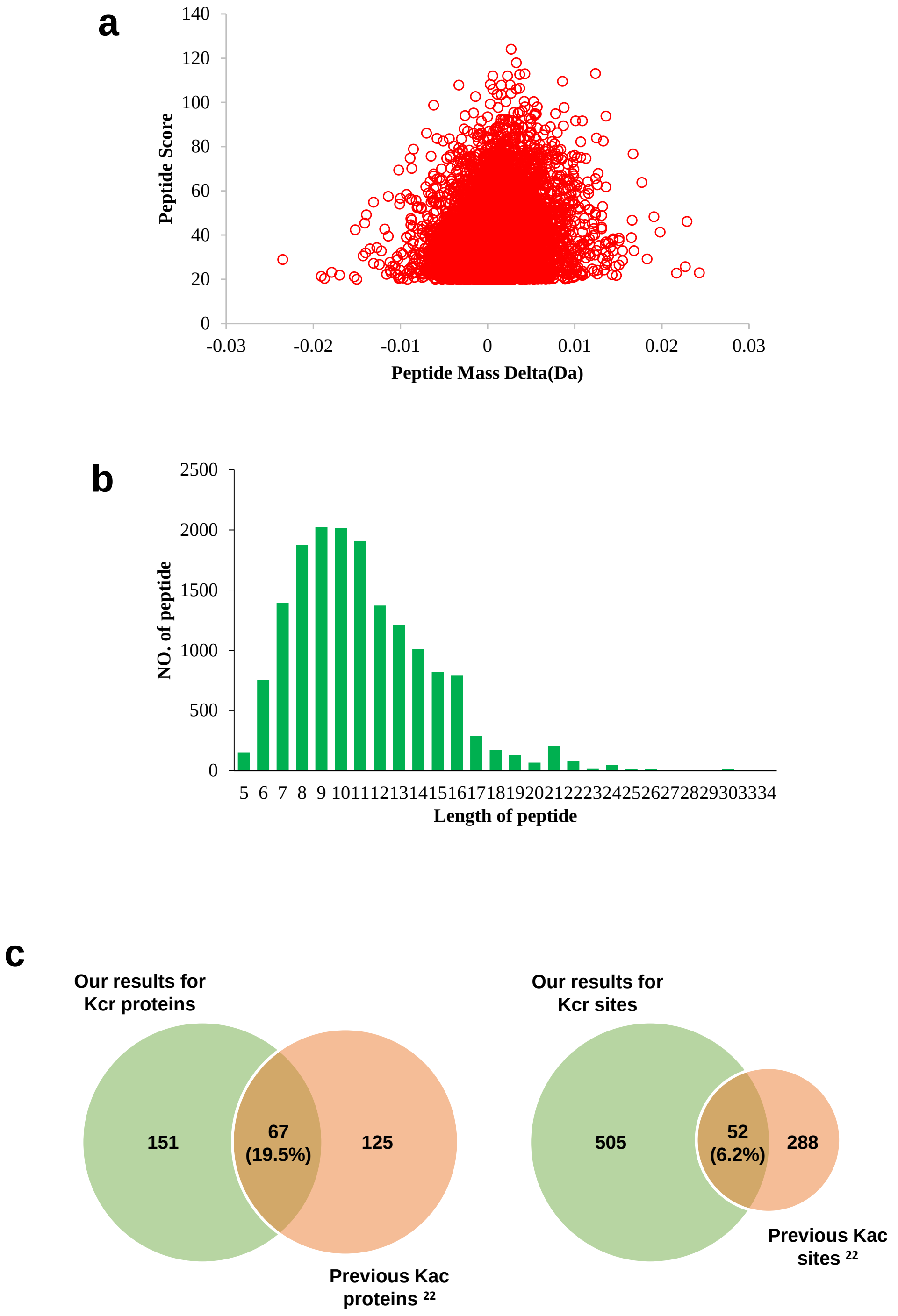

Supplementary Fig S1. QC validation of MS data and comparison between Kcr and Kac in zebrafish. (A) Distribution of mass error for precursor ions. (B) Peptide length distribution of identified crotonylated peptides. (C) Comparison of proteins and sites between current Kcr results and previous Kac results in zebrafish.

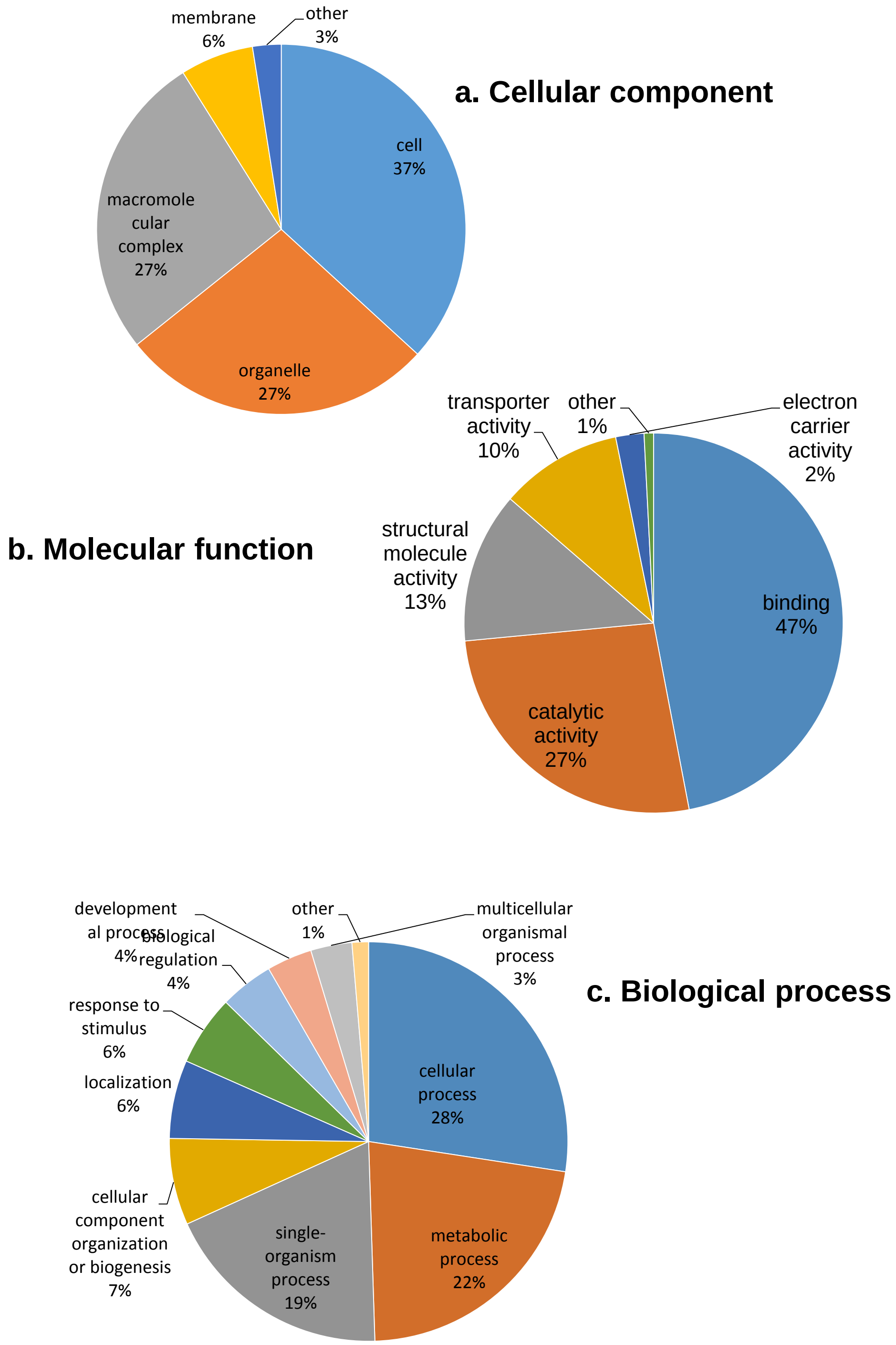
a. Cellular component
membrane 6%
other 3%
cell 37%
macromolecular complex 27%
organelle 27%
b. Molecular function
transporter activity 10%
other 1%
electron carrier activity 2%
structural molecule activity 13%
binding 47%
catalytic activity 27%
c. Biological process
developmental process 4%
biological regulation 4%
other 1%
multicellular organismal process 3%
response to stimulus 6%
localization 6%
cellular process 28%
cellular component organization or biogenesis 7%
single-organism process 19%
metabolic process 22%

d. Subcellular location

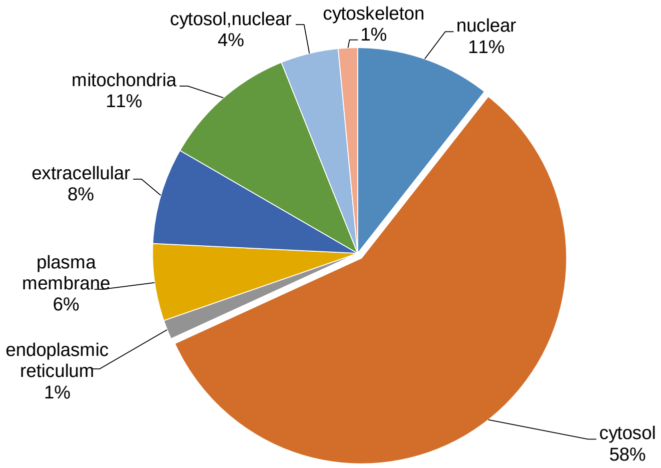

Supplementary Fig S2. GO-based annotation analysis of identified crotonylated proteins in zebrafish larvae. (A) Biological process (B), Molecular function, (C) Subcellular location, (D) Subcellular location.

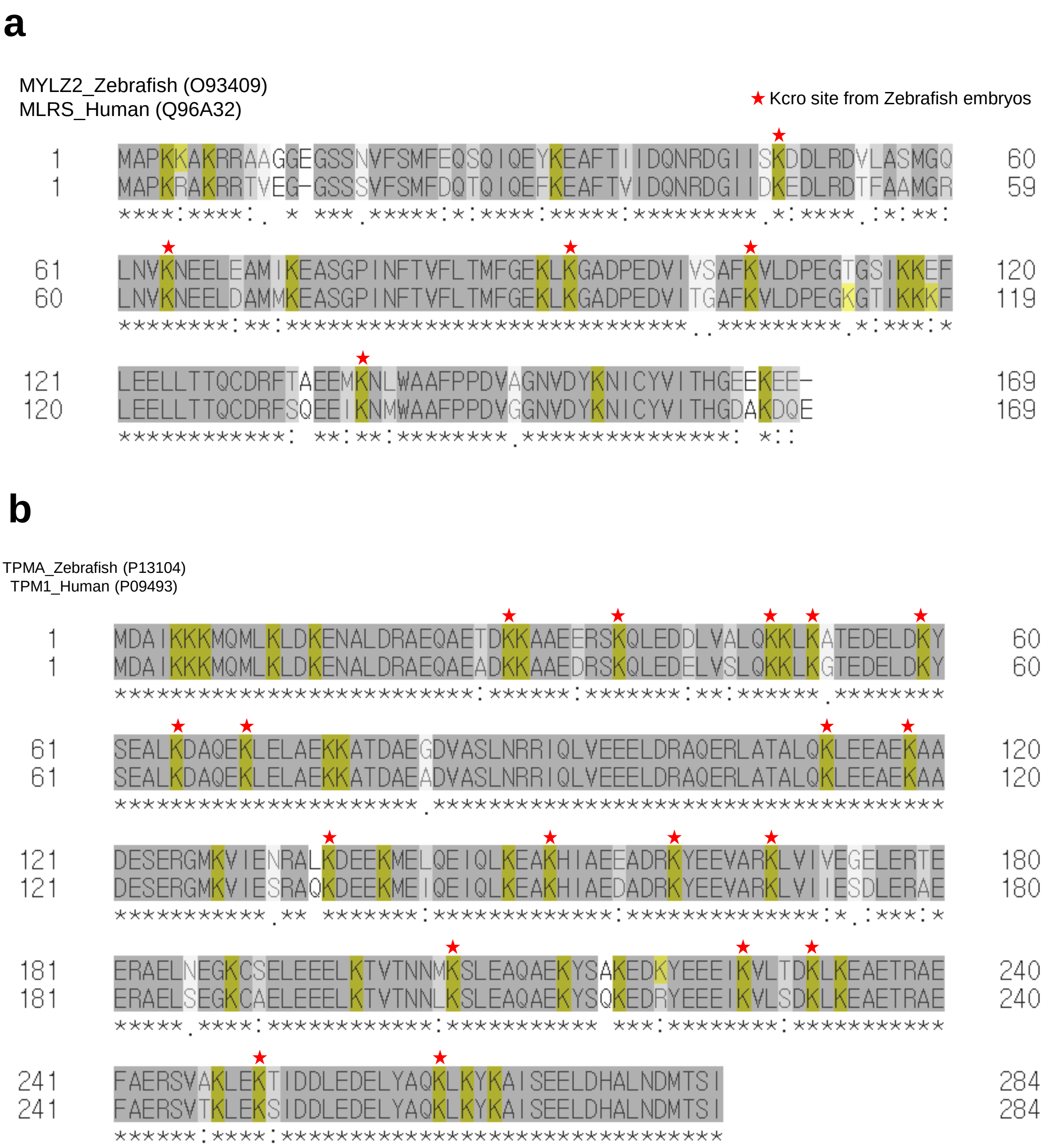
a
MYLZ2_Zebrafish (O93409)
MLRS_Human (Q96A32)
★ Kcro site from Zebrafish embryos
b
TPMA_Zebrafish (P13104)
TPM1_Human (P09493)

c

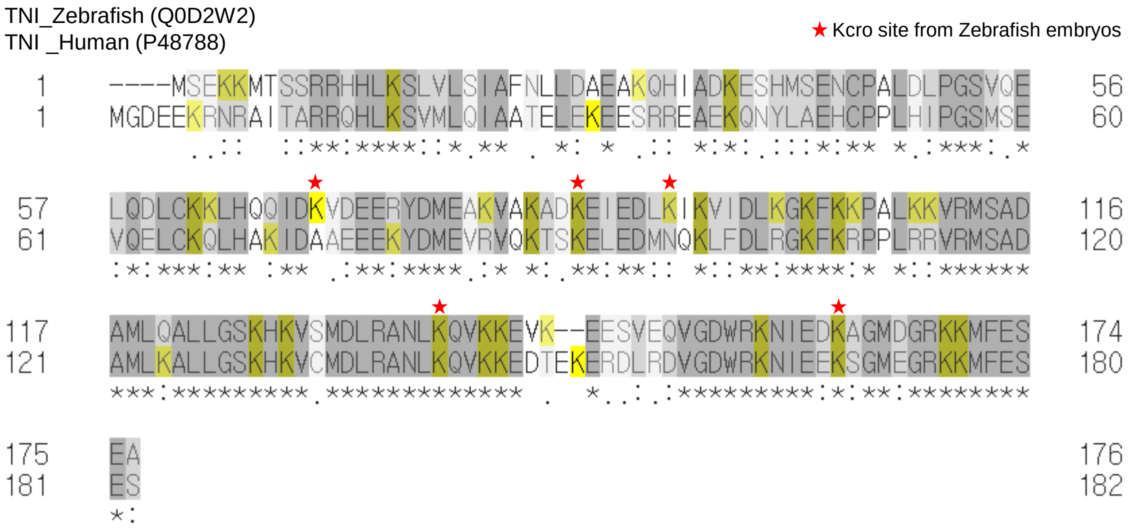

d

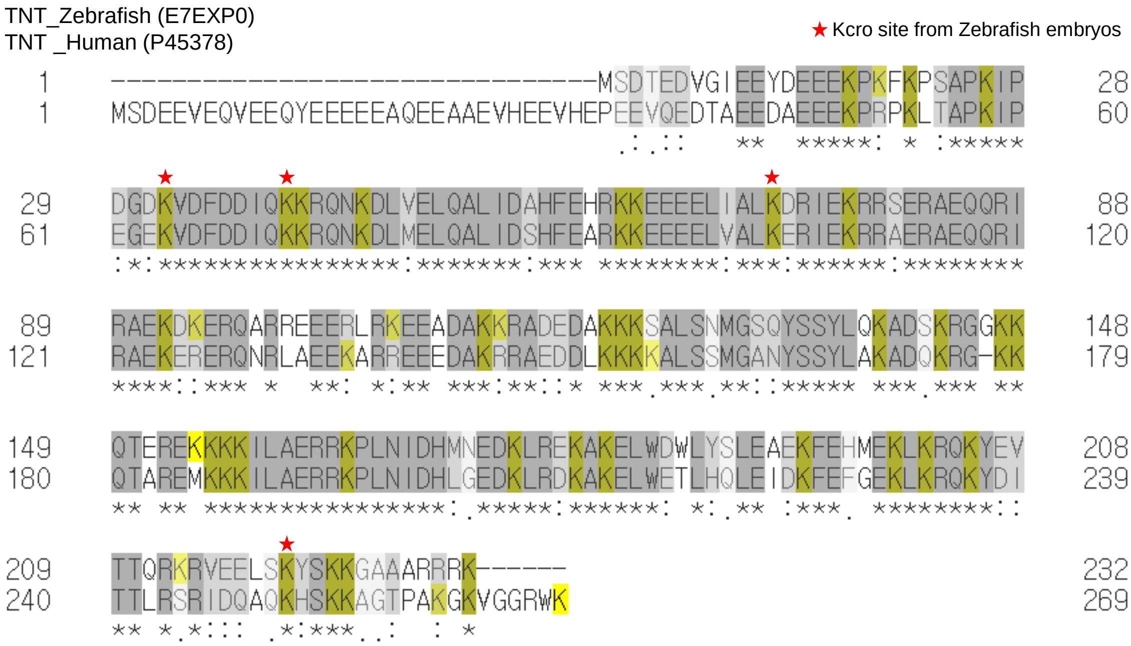

Supplementary Fig S3. Sequence alignment of crotonylated myosin between zebrafish and humans. (A) Crotonylated myosin light chain, (B) Tropomyosin (C) Troponin I, (D) Troponin T.

Uniprot ID	Gene names	Protein names	Modified sequence	Position	# modificiation	Surrounding sequence	Motif	Subcellular Location	Biological Process	Cellular Component	Molecular Function	KEGG KO No.	KEGG Gene	KEGG Pathway	IPR ID	Domain description
B8A568	myhz1.1	Uncharacterized protein	_GK(cr)QAFTQQIEELK(cr)R_	1307	2	VSQLTRGKQAFTQQI		nuclear		intracellular organelle; intracellular non-membrane-bounded organelle; cytoskeletal part; intracellular organelle part; macromolecular complex; intracellular part; cell; actin cytoskeleton; organelle part; non-membrane-bounded organelle; organelle; cytoskeleton; cell part; intracellular; myosin complex; protein complex;	ATP binding; motor activity; nucleoside phosphate binding; nucleoside binding; ion binding; hydrolase activity, acting on acid anhydrides; hydrolase activity; nucleotide binding; nucleoside-triphosphatase activity; organic cyclic compound binding; heterocyclic compound binding; ribonucleoside binding; carbohydrate derivative binding; pyrophosphatase activity; catalytic activity; ribonucleotide binding; binding; purine ribonucleoside binding; anion binding; purine ribonucleotide binding; adenyl ribonucleotide binding; adenyl nucleotide binding; purine nucleoside binding; hydrolase activity, acting on acid anhydrides, in phosphorus-containing anhydrides; purine nucleotide binding; small molecule binding; purine ribonucleoside triphosphate binding;	K10352	MYH; myosin heavy chain	dre04530 Tight junction - Danio rerio (zebrafish);	IPR002928; IPR004009; IPR001609; IPR027417; IPR027401;	Myosin tail; Myosin, N-terminal, SH3-like; Myosin head, motor domain; P-loop containing nucleoside triphosphate hydrolase; Myosin-like IQ motif-containing domain;
B8A568	myhz1.1	Uncharacterized protein	_GK(cr)QAFTQQIEELK(cr)R_	1318	2	TQQIEELKRQIEEEV		nuclear		intracellular organelle; intracellular non-membrane-bounded organelle; cytoskeletal part; intracellular organelle part; macromolecular complex; intracellular part; cell; actin cytoskeleton; organelle part; non-membrane-bounded organelle; organelle; cytoskeleton; cell part; intracellular; myosin complex; protein complex;	ATP binding; motor activity; nucleoside phosphate binding; nucleoside binding; ion binding; hydrolase activity, acting on acid anhydrides; hydrolase activity; nucleotide binding; nucleoside-triphosphatase activity; organic cyclic compound binding; heterocyclic compound binding; ribonucleoside binding; carbohydrate derivative binding; pyrophosphatase activity; catalytic activity; ribonucleotide binding; binding; purine ribonucleoside binding; anion binding; purine ribonucleotide binding; adenyl ribonucleotide binding; adenyl nucleotide binding; purine nucleoside binding; hydrolase activity, acting on acid anhydrides, in phosphorus-containing anhydrides; purine nucleotide binding; small molecule binding; purine ribonucleoside triphosphate binding;	K10352	MYH; myosin heavy chain	dre04530 Tight junction - Danio rerio (zebrafish);	IPR002928; IPR004009; IPR001609; IPR027417; IPR027401;	Myosin tail; Myosin, N-terminal, SH3-like; Myosin head, motor domain; P-loop containing nucleoside triphosphate hydrolase; Myosin-like IQ motif-containing domain;
B8A568	myhz1.1	Uncharacterized protein	_K(cr)LAEK(cr)DEEIEQIK_	1577	2	VKSEIDRKLAEKDEE		nuclear		intracellular organelle; intracellular non-membrane-bounded organelle; cytoskeletal part; intracellular organelle part; macromolecular complex; intracellular part; cell; actin cytoskeleton; organelle part; non-membrane-bounded organelle; organelle; cytoskeleton; cell part; intracellular; myosin complex; protein complex;	ATP binding; motor activity; nucleoside phosphate binding; nucleoside binding; ion binding; hydrolase activity, acting on acid anhydrides; hydrolase activity; nucleotide binding; nucleoside-triphosphatase activity; organic cyclic compound binding; heterocyclic compound binding; ribonucleoside binding; carbohydrate derivative binding; pyrophosphatase activity; catalytic activity; ribonucleotide binding; binding; purine ribonucleoside binding; anion binding; purine ribonucleotide binding; adenyl ribonucleotide binding; adenyl nucleotide binding; purine nucleoside binding; hydrolase activity, acting on acid anhydrides, in phosphorus-containing anhydrides; purine nucleotide binding; small molecule binding; purine ribonucleoside triphosphate binding;	K10352	MYH; myosin heavy chain	dre04530 Tight junction - Danio rerio (zebrafish);	IPR002928; IPR004009; IPR001609; IPR027417; IPR027401;	Myosin tail; Myosin, N-terminal, SH3-like; Myosin head, motor domain; P-loop containing nucleoside triphosphate hydrolase; Myosin-like IQ motif-containing domain;
B8A568	myhz1.1	Uncharacterized protein	_K(cr)LAEK(cr)DEEIEQIK_	1581	2	IDRKLAEKDEEIEQI	.......K.E.....	nuclear		intracellular organelle; intracellular non-membrane-bounded organelle; cytoskeletal part; intracellular organelle part; macromolecular complex; intracellular part; cell; actin cytoskeleton; organelle part; non-membrane-bounded organelle; organelle; cytoskeleton; cell part; intracellular; myosin complex; protein complex;	ATP binding; motor activity; nucleoside phosphate binding; nucleoside binding; ion binding; hydrolase activity, acting on acid anhydrides; hydrolase activity; nucleotide binding; nucleoside-triphosphatase activity; organic cyclic compound binding; heterocyclic compound binding; ribonucleoside binding; carbohydrate derivative binding; pyrophosphatase activity; catalytic activity; ribonucleotide binding; binding; purine ribonucleoside binding; anion binding; purine ribonucleotide binding; adenyl ribonucleotide binding; adenyl nucleotide binding; purine nucleoside binding; hydrolase activity, acting on acid anhydrides, in phosphorus-containing anhydrides; purine nucleotide binding; small molecule binding; purine ribonucleoside triphosphate binding;	K10352	MYH; myosin heavy chain	dre04530 Tight junction - Danio rerio (zebrafish);	IPR002928; IPR004009; IPR001609; IPR027417; IPR027401;	Myosin tail; Myosin, N-terminal, SH3-like; Myosin head, motor domain; P-loop containing nucleoside triphosphate hydrolase; Myosin-like IQ motif-containing domain;
B8A568	myhz1.1	Uncharacterized protein	_LQDLVDK(cr)LQLK(cr)VK_	1872	2	RLQDLVDKLQLKVKA	......DK.......	nuclear		intracellular organelle; intracellular non-membrane-bounded organelle; cytoskeletal part; intracellular organelle part; macromolecular complex; intracellular part; cell; actin cytoskeleton; organelle part; non-membrane-bounded organelle; organelle; cytoskeleton; cell part; intracellular; myosin complex; protein complex;	ATP binding; motor activity; nucleoside phosphate binding; nucleoside binding; ion binding; hydrolase activity, acting on acid anhydrides; hydrolase activity; nucleotide binding; nucleoside-triphosphatase activity; organic cyclic compound binding; heterocyclic compound binding; ribonucleoside binding; carbohydrate derivative binding; pyrophosphatase activity; catalytic activity; ribonucleotide binding; binding; purine ribonucleoside binding; anion binding; purine ribonucleotide binding; adenyl ribonucleotide binding; adenyl nucleotide binding; purine nucleoside binding; hydrolase activity, acting on acid anhydrides, in phosphorus-containing anhydrides; purine nucleotide binding; small molecule binding; purine ribonucleoside triphosphate binding;	K10352	MYH; myosin heavy chain	dre04530 Tight junction - Danio rerio (zebrafish);	IPR002928; IPR004009; IPR001609; IPR027417; IPR027401;	Myosin tail; Myosin, N-terminal, SH3-like; Myosin head, motor domain; P-loop containing nucleoside triphosphate hydrolase; Myosin-like IQ motif-containing domain;
B8A568	myhz1.1	Uncharacterized protein	_LQDLVDK(cr)LQLK(cr)VK_	1876	2	LVDKLQLKVKAYKRQ		nuclear		intracellular organelle; intracellular non-membrane-bounded organelle; cytoskeletal part; intracellular organelle part; macromolecular complex; intracellular part; cell; actin cytoskeleton; organelle part; non-membrane-bounded organelle; organelle; cytoskeleton; cell part; intracellular; myosin complex; protein complex;	ATP binding; motor activity; nucleoside phosphate binding; nucleoside binding; ion binding; hydrolase activity, acting on acid anhydrides; hydrolase activity; nucleotide binding; nucleoside-triphosphatase activity; organic cyclic compound binding; heterocyclic compound binding; ribonucleoside binding; carbohydrate derivative binding; pyrophosphatase activity; catalytic activity; ribonucleotide binding; binding; purine ribonucleoside binding; anion binding; purine ribonucleotide binding; adenyl ribonucleotide binding; adenyl nucleotide binding; purine nucleoside binding; hydrolase activity, acting on acid anhydrides, in phosphorus-containing anhydrides; purine nucleotide binding; small molecule binding; purine ribonucleoside triphosphate binding;	K10352	MYH; myosin heavy chain	dre04530 Tight junction - Danio rerio (zebrafish);	IPR002928; IPR004009; IPR001609; IPR027417; IPR027401;	Myosin tail; Myosin, N-terminal, SH3-like; Myosin head, motor domain; P-loop containing nucleoside triphosphate hydrolase; Myosin-like IQ motif-containing domain;
B8A561	myhz1.2	Uncharacterized protein	_LAEK(cr)DEEIEQIK(cr)R_	1581	2	IDRKLAEKDEEIEQI	.......K.E.....	nuclear		intracellular organelle; intracellular non-membrane-bounded organelle; cytoskeletal part; intracellular organelle part; macromolecular complex; intracellular part; cell; actin cytoskeleton; organelle part; non-membrane-bounded organelle; organelle; cytoskeleton; cell part; intracellular; myosin complex; protein complex;	ATP binding; motor activity; nucleoside phosphate binding; nucleoside binding; ion binding; hydrolase activity, acting on acid anhydrides; hydrolase activity; nucleotide binding; nucleoside-triphosphatase activity; organic cyclic compound binding; heterocyclic compound binding; ribonucleoside binding; carbohydrate derivative binding; pyrophosphatase activity; catalytic activity; ribonucleotide binding; binding; purine ribonucleoside binding; anion binding; purine ribonucleotide binding; adenyl ribonucleotide binding; adenyl nucleotide binding; purine nucleoside binding; hydrolase activity, acting on acid anhydrides, in phosphorus-containing anhydrides; purine nucleotide binding; small molecule binding; purine ribonucleoside triphosphate binding;	K10352	MYH; myosin heavy chain	dre04530 Tight junction - Danio rerio (zebrafish);	IPR002928; IPR004009; IPR001609; IPR027417; IPR027401;	Myosin tail; Myosin, N-terminal, SH3-like; Myosin head, motor domain; P-loop containing nucleoside triphosphate hydrolase; Myosin-like IQ motif-containing domain;

B8A561	myhz1.2	Uncharacterized protein	_LAEK(cr)DEEIEQIK(cr)R_	1589	2	DEEIEQIKRNSQRIT	..E....K.......	nuclear		intracellular organelle; intracellular non-membrane-bounded organelle; cytoskeletal part; intracellular organelle part; macromolecular complex; intracellular part; cell; actin cytoskeleton; organelle part; non-membrane-bounded organelle; organelle; cytoskeleton; cell part; intracellular; myosin complex; protein complex;	ATP binding; motor activity; nucleoside phosphate binding; nucleoside binding; ion binding; hydrolase activity, acting on acid anhydrides; hydrolase activity; nucleotide binding; nucleoside-triphosphatase activity; organic cyclic compound binding; heterocyclic compound binding; ribonucleoside binding; carbohydrate derivative binding; pyrophosphatase activity; catalytic activity; ribonucleotide binding; binding; purine ribonucleoside binding; anion binding; purine ribonucleotide binding; adenyl ribonucleotide binding; adenyl nucleotide binding; purine nucleoside binding; hydrolase activity, acting on acid anhydrides, in phosphorus-containing anhydrides; purine nucleotide binding; small molecule binding; purine ribonucleoside triphosphate binding;	K10352	MYH; myosin heavy chain	dre04530 Tight junction - Danio rerio (zebrafish);	IPR002928; IPR004009; IPR001609; IPR027417; IPR027401;	Myosin tail; Myosin, N-terminal, SH3-like; Myosin head, motor domain; P-loop containing nucleoside triphosphate hydrolase; Myosin-like IQ motif-containing domain;
B8A561	myhz1.2	Uncharacterized protein	_K(cr)LAEK(cr)DEEIEQIK_	1577	2	VKSEIDRKLAEKDEE		nuclear		intracellular organelle; intracellular non-membrane-bounded organelle; cytoskeletal part; intracellular organelle part; macromolecular complex; intracellular part; cell; actin cytoskeleton; organelle part; non-membrane-bounded organelle; organelle; cytoskeleton; cell part; intracellular; myosin complex; protein complex;	ATP binding; motor activity; nucleoside phosphate binding; nucleoside binding; ion binding; hydrolase activity, acting on acid anhydrides; hydrolase activity; nucleotide binding; nucleoside-triphosphatase activity; organic cyclic compound binding; heterocyclic compound binding; ribonucleoside binding; carbohydrate derivative binding; pyrophosphatase activity; catalytic activity; ribonucleotide binding; binding; purine ribonucleoside binding; anion binding; purine ribonucleotide binding; adenyl ribonucleotide binding; adenyl nucleotide binding; purine nucleoside binding; hydrolase activity, acting on acid anhydrides, in phosphorus-containing anhydrides; purine nucleotide binding; small molecule binding; purine ribonucleoside triphosphate binding;	K10352	MYH; myosin heavy chain	dre04530 Tight junction - Danio rerio (zebrafish);	IPR002928; IPR004009; IPR001609; IPR027417; IPR027401;	Myosin tail; Myosin, N-terminal, SH3-like; Myosin head, motor domain; P-loop containing nucleoside triphosphate hydrolase; Myosin-like IQ motif-containing domain;
B8A561	myhz1.2	Uncharacterized protein	_K(cr)LAEK(cr)DEEIEQIK_	1581	2	IDRKLAEKDEEIEQI	.......K.E.....	nuclear		intracellular organelle; intracellular non-membrane-bounded organelle; cytoskeletal part; intracellular organelle part; macromolecular complex; intracellular part; cell; actin cytoskeleton; organelle part; non-membrane-bounded organelle; organelle; cytoskeleton; cell part; intracellular; myosin complex; protein complex;	ATP binding; motor activity; nucleoside phosphate binding; nucleoside binding; ion binding; hydrolase activity, acting on acid anhydrides; hydrolase activity; nucleotide binding; nucleoside-triphosphatase activity; organic cyclic compound binding; heterocyclic compound binding; ribonucleoside binding; carbohydrate derivative binding; pyrophosphatase activity; catalytic activity; ribonucleotide binding; binding; purine ribonucleoside binding; anion binding; purine ribonucleotide binding; adenyl ribonucleotide binding; adenyl nucleotide binding; purine nucleoside binding; hydrolase activity, acting on acid anhydrides, in phosphorus-containing anhydrides; purine nucleotide binding; small molecule binding; purine ribonucleoside triphosphate binding;	K10352	MYH; myosin heavy chain	dre04530 Tight junction - Danio rerio (zebrafish);	IPR002928; IPR004009; IPR001609; IPR027417; IPR027401;	Myosin tail; Myosin, N-terminal, SH3-like; Myosin head, motor domain; P-loop containing nucleoside triphosphate hydrolase; Myosin-like IQ motif-containing domain;
B8A561	myhz1.2	Uncharacterized protein	_GK(cr)QAFTQQIEELK(cr)R_	1307	2	VSQLTRGKQAFTQQI		nuclear		intracellular organelle; intracellular non-membrane-bounded organelle; cytoskeletal part; intracellular organelle part; macromolecular complex; intracellular part; cell; actin cytoskeleton; organelle part; non-membrane-bounded organelle; organelle; cytoskeleton; cell part; intracellular; myosin complex; protein complex;	ATP binding; motor activity; nucleoside phosphate binding; nucleoside binding; ion binding; hydrolase activity, acting on acid anhydrides; hydrolase activity; nucleotide binding; nucleoside-triphosphatase activity; organic cyclic compound binding; heterocyclic compound binding; ribonucleoside binding; carbohydrate derivative binding; pyrophosphatase activity; catalytic activity; ribonucleotide binding; binding; purine ribonucleoside binding; anion binding; purine ribonucleotide binding; adenyl ribonucleotide binding; adenyl nucleotide binding; purine nucleoside binding; hydrolase activity, acting on acid anhydrides, in phosphorus-containing anhydrides; purine nucleotide binding; small molecule binding; purine ribonucleoside triphosphate binding;	K10352	MYH; myosin heavy chain	dre04530 Tight junction - Danio rerio (zebrafish);	IPR002928; IPR004009; IPR001609; IPR027417; IPR027401;	Myosin tail; Myosin, N-terminal, SH3-like; Myosin head, motor domain; P-loop containing nucleoside triphosphate hydrolase; Myosin-like IQ motif-containing domain;
B8A561	myhz1.2	Uncharacterized protein	_GK(cr)QAFTQQIEELK(cr)R_	1318	2	TQQIEELKRQIEEEV		nuclear		intracellular organelle; intracellular non-membrane-bounded organelle; cytoskeletal part; intracellular organelle part; macromolecular complex; intracellular part; cell; actin cytoskeleton; organelle part; non-membrane-bounded organelle; organelle; cytoskeleton; cell part; intracellular; myosin complex; protein complex;	ATP binding; motor activity; nucleoside phosphate binding; nucleoside binding; ion binding; hydrolase activity, acting on acid anhydrides; hydrolase activity; nucleotide binding; nucleoside-triphosphatase activity; organic cyclic compound binding; heterocyclic compound binding; ribonucleoside binding; carbohydrate derivative binding; pyrophosphatase activity; catalytic activity; ribonucleotide binding; binding; purine ribonucleoside binding; anion binding; purine ribonucleotide binding; adenyl ribonucleotide binding; adenyl nucleotide binding; purine nucleoside binding; hydrolase activity, acting on acid anhydrides, in phosphorus-containing anhydrides; purine nucleotide binding; small molecule binding; purine ribonucleoside triphosphate binding;	K10352	MYH; myosin heavy chain	dre04530 Tight junction - Danio rerio (zebrafish);	IPR002928; IPR004009; IPR001609; IPR027417; IPR027401;	Myosin tail; Myosin, N-terminal, SH3-like; Myosin head, motor domain; P-loop containing nucleoside triphosphate hydrolase; Myosin-like IQ motif-containing domain;
E9QJI6	eno3	Enolase	_(ac)SISK(cr)IHAR_	5	1	__MSISKIHAREILD		cytosol	single-organism carbohydrate catabolic process; glycolytic process; single-organism catabolic process; small molecule metabolic process; single-organism cellular process; single-organism process; carbohydrate metabolic process; primary metabolic process; cellular metabolic process; organic substance metabolic process; carbohydrate catabolic process; single-organism carbohydrate metabolic process; organic substance catabolic process; metabolic process; pyruvate metabolic process; generation of precursor metabolites and energy; single-organism metabolic process; carboxylic acid metabolic process; monocarboxylic acid metabolic process; organic acid metabolic process; cellular process; catabolic process; oxoacid metabolic process;	catalytic complex; cell part; intracellular; cytoplasm; macromolecular complex; intracellular part; cytosolic part; cell; cytosol; protein complex; phosphopyruvate hydratase complex; cytoplasmic part;	magnesium ion binding; phosphopyruvate hydratase activity; catalytic activity; binding; ion binding; metal ion binding; cation binding; lyase activity; hydro-lyase activity; carbon-oxygen lyase activity;				IPR029065; IPR029017; IPR020810; IPR020811;	Enolase C-terminal domain-like; Enolase N-terminal domain-like; Enolase, C-terminal; Enolase, N-terminal;

O42363	apoa1	Apolipoprotein A-I	_AAALVYLNQVK(cr)DQAEK_	44	1	LVYLNQVKDQAEKAL		extracellular	cholesterol metabolic process; lipid transport; organic hydroxy compound metabolic process; alcohol metabolic process; small molecule metabolic process; steroid metabolic process; organic cyclic compound metabolic process; single-organism process; organic substance transport; lipid localization; primary metabolic process; sterol metabolic process; lipoprotein metabolic process; establishment of localization; organic substance metabolic process; transport; metabolic process; single-organism localization; single-organism metabolic process; macromolecule metabolic process; protein metabolic process; localization; macromolecule localization; single-organism transport; lipid metabolic process;	macromolecular complex; extracellular region part; extracellular space; high-density lipoprotein particle; protein-lipid complex; extracellular region; plasma lipoprotein particle;	lipid binding; binding;	K08757	APOA1; apolipoprotein A-I	dre03320 PPAR signaling pathway - Danio rerio (zebrafish);		
Q6P5L2	dbi	Uncharacterized protein	_AAEEVK(cr)QLK_	14	1	QKAAEEVKQLKAKPT	.......K.L.....	cytosol			fatty-acyl-CoA binding; binding; ion binding; anion binding; cofactor binding; sulfur compound binding; coenzyme binding;	K08762	DBI; diazepam-binding inhibitor (GABA receptor modulator, acyl-CoA-binding protein)	dre03320 PPAR signaling pathway - Danio rerio (zebrafish);	IPR000582; IPR014352;	Acyl-CoA-binding protein, ACBP; FERM/acyl-CoA-binding protein, 3-helical bundle;
E9QGA9	gapdh	Glyceraldehyde-3-phosphate dehydrogenase	_AAFLTK(cr)K_	24	1	TRAAFLTKKVEIVAI	.......K.V.....	cytosol			organic cyclic compound binding; heterocyclic compound binding; oxidoreductase activity, acting on the aldehyde or oxo group of donors, NAD or NADP as acceptor; nucleoside phosphate binding; catalytic activity; binding; oxidoreductase activity, acting on the aldehyde or oxo group of donors; nucleotide binding; small molecule binding; oxidoreductase activity; glyceraldehyde-3-phosphate dehydrogenase (NAD+) (phosphorylating) activity;				IPR020829; IPR016040; IPR020828;	Glyceraldehyde 3-phosphate dehydrogenase, catalytic domain; NAD(P)-binding domain; Glyceraldehyde 3-phosphate dehydrogenase, NAD(P) binding domain;
B0UY61	neb	Uncharacterized protein	_AAK(cr)EAYK_	5231	1	AHEIRAAKEAYKNIS	..E....K.......	nuclear				K18267			IPR001452;	SH3 domain;
Q6PBZ1	rpl7a	Uncharacterized protein	_AALAK(cr)LVEAIK_	217	1	EDKAALAKLVEAIKT	.......K.V.....	plasma membrane	cellular component biogenesis; ribosome biogenesis; ribonucleoprotein complex biogenesis; cellular component organization or biogenesis;	ribosome; organelle; intracellular organelle; intracellular non-membrane-bounded organelle; cell part; intracellular; cytoplasm; macromolecular complex; intracellular part; cell; ribonucleoprotein complex; cytoplasmic part; non-membrane-bounded organelle;		K02936	RP-L7Ae; large subunit ribosomal protein L7Ae	dre03010 Ribosome - Danio rerio (zebrafish);	IPR029064; IPR004038;	50S ribosomal protein L30e-like; Ribosomal protein L7Ae/L30e/S12e/Gadd45;
Q6XG62	icn	Protein S100	_AALDK(cr)IFK_	58	1	TDKAALDKIFKDLDA	......DK.......	cytosol			cation binding; metal ion binding; calcium ion binding; binding; ion binding;				IPR011992; IPR002048; IPR013787;	EF-hand domain pair; EF-hand domain; S100/CaBP-9k-type, calcium binding, subdomain;
Q6PBI5	rps25	40S ribosomal protein S25	_AALQELLGK(cr)GLIK_	93	1	ALQELLGKGLIKLVS	.......K.L.....	nuclear		ribosome; organelle; intracellular organelle; intracellular non-membrane-bounded organelle; cell part; intracellular; cytoplasm; macromolecular complex; intracellular part; cell; ribonucleoprotein complex; cytoplasmic part; non-membrane-bounded organelle;		K02975	RP-S25e; small subunit ribosomal protein S25e	dre03010 Ribosome - Danio rerio (zebrafish);		
A5WWJ4	matn1	Uncharacterized protein	_AALVK(cr)AVSK_	103	1	KTKAALVKAVSKIEP	.......K.V.....	extracellular			cation binding; metal ion binding; calcium ion binding; binding; ion binding;				IPR019466; IPR000742; IPR002035;	Matrilin, coiled-coil trimerisation domain; Epidermal growth factor-like domain; von Willebrand factor, type A;
F1R5A5	krt5	Uncharacterized protein	_AAVENEFVLLK(cr)K_	263	1	ENEFVLLKKDVDAAY	..E....K.......	nuclear		intracellular organelle; intracellular non-membrane-bounded organelle; cytoskeletal part; intracellular organelle part; macromolecular complex; intracellular part; cell; keratin filament; organelle part; non-membrane-bounded organelle; organelle; cytoskeleton; intermediate filament cytoskeleton; cell part; intracellular; intermediate filament; protein complex;	structural molecule activity;	K07605				

Q6PC77	atp5h	ATP synthase, H+ transporting, mitochondrial F0 complex, subunit d	_AAVK(cr)AIDWLAFAER_	9	1	AGRRAAVKAIDWLAF	.......K.I.....	cytosol	nucleoside metabolic process; purine nucleoside metabolic process; purine ribonucleoside monophosphate metabolic process; nucleotide metabolic process; single-organism process; purine ribonucleoside biosynthetic process; nucleoside biosynthetic process; biosynthetic process; ribonucleoside monophosphate biosynthetic process; cellular metabolic process; phosphate-containing compound metabolic process; establishment of localization; ribose phosphate biosynthetic process; metabolic process; carbohydrate derivative biosynthetic process; nucleoside phosphate metabolic process; ribose phosphate metabolic process; purine nucleoside triphosphate biosynthetic process; single-organism transport; heterocycle biosynthetic process; cation transmembrane transport; purine ribonucleoside triphosphate biosynthetic process; small molecule metabolic process; ATP biosynthetic process; single-organism cellular process; purine ribonucleoside triphosphate metabolic process; organic cyclic compound metabolic process; purine-containing compound metabolic process; energy coupled proton transport, down electrochemical gradient; ribonucleoside triphosphate metabolic process; purine	intracellular organelle; membrane; mitochondrial part; organelle membrane; organelle envelope; intracellular organelle part; cytoplasm; macromolecular complex; intracellular part; mitochondrial proton-transporting ATP synthase complex, coupling factor F(o); cell; mitochondrial membrane part; proton-transporting ATP synthase complex; mitochondrion; organelle part; intracellular membrane-bounded organelle; organelle; mitochondrial envelope; mitochondrial proton-transporting ATP synthase complex; membrane part; cell part; intracellular; proton-transporting two-sector ATPase complex; envelope; organelle inner membrane; proton-transporting ATP synthase complex, coupling factor F(o); protein complex; membrane-bounded organelle; mitochondrial inner membrane; proton-transporting two-sector ATPase complex, proton-transporting domain; mitochondrial membrane; cytoplasmic part;	ion transmembrane transporter activity; substrate-specific transporter activity; monovalent inorganic cation transmembrane transporter activity; inorganic cation transmembrane transporter activity; hydrogen ion transmembrane transporter activity; cation transmembrane transporter activity; substrate-specific transmembrane transporter activity; transporter activity; transmembrane transporter activity;	K02138	ATPeF0D; F-type H+-transportin g ATPase subunit d	dre01100 Metabolic pathways - Danio rerio (zebrafish); dre00190 Oxidative phosphorylation - Danio rerio (zebrafish);		
Q7ZT21	hbae1	Embryonic alpha globin e1	_AAVK(cr)TLWAK_	12	1	AKDKAAVKTLWAKIA	.......K.L......	cytosol		cell part; intracellular; hemoglobin complex; cytoplasm; macromolecular complex; intracellular part; cytosolic part; protein complex; cell; cytosol; cytoplasmic part;	organic cyclic compound binding; heterocyclic compound binding; heme binding; iron ion binding; transition metal ion binding; oxygen binding; binding; ion binding; substrate-specific transporter activity; metal ion binding; cation binding; oxygen transporter activity; tetrapyrrole binding; transporter activity;	K13826			IPR009050; IPR000971; IPR012292;	Globin-like; Globin; Globin, structural domain;
Q6P0G6	myl1	Uncharacterized protein	_ADAVDLSGVK(cr)LDFTQDQM(ox)EDYR_	40	1	AVDLSGVKLDFTQDQ		cytosol			cation binding; metal ion binding; calcium ion binding; binding; ion binding;	K05738			IPR002048; IPR011992;	EF-hand domain; EF-hand domain pair;
Q6P0G6	myl1	Uncharacterized protein	_ADAVDLSGVK(cr)LDFTQDQMEDYR_	40	1	AVDLSGVKLDFTQDQ		cytosol			cation binding; metal ion binding; calcium ion binding; binding; ion binding;	K05738			IPR002048; IPR011992;	EF-hand domain; EF-hand domain pair;
B8A568	myhz1.1	Uncharacterized protein	_ADIAESQVNK(cr)LR_	1921	1	IAESQVNKLRAKSRD	..E....K.......	nuclear		intracellular organelle; intracellular non-membrane-bounded organelle; cytoskeletal part; intracellular organelle part; macromolecular complex; intracellular part; cell; actin cytoskeleton; organelle part; non-membrane-bounded organelle; organelle; cytoskeleton; cell part; intracellular; myosin complex; protein complex;	ATP binding; motor activity; nucleoside phosphate binding; nucleoside binding; ion binding; hydrolase activity, acting on acid anhydrides; hydrolase activity; nucleotide binding; nucleoside-triphosphatase activity; organic cyclic compound binding; heterocyclic compound binding; ribonucleoside binding; carbohydrate derivative binding; pyrophosphatase activity; catalytic activity; ribonucleotide binding; binding; purine ribonucleoside binding; anion binding; purine ribonucleotide binding; adenyl ribonucleotide binding; adenyl nucleotide binding; purine nucleoside binding; hydrolase activity, acting on acid anhydrides, in phosphorus-containing anhydrides; purine nucleotide binding; small molecule binding; purine ribonucleoside triphosphate binding;	K10352	MYH; myosin heavy chain	dre04530 Tight junction - Danio rerio (zebrafish);	IPR002928; IPR004009; IPR001609; IPR027417; IPR027401;	Myosin tail; Myosin, N-terminal, SH3-like; Myosin head, motor domain; P-loop containing nucleoside triphosphate hydrolase; Myosin-like IQ motif-containing domain;
B8A561	myhz1.2	Uncharacterized protein	_ADIAESQVNK(cr)LR_	1921	1	IAESQVNKLRAKSRD	..E....K.......	nuclear		intracellular organelle; intracellular non-membrane-bounded organelle; cytoskeletal part; intracellular organelle part; macromolecular complex; intracellular part; cell; actin cytoskeleton; organelle part; non-membrane-bounded organelle; organelle; cytoskeleton; cell part; intracellular; myosin complex; protein complex;	ATP binding; motor activity; nucleoside phosphate binding; nucleoside binding; ion binding; hydrolase activity, acting on acid anhydrides; hydrolase activity; nucleotide binding; nucleoside-triphosphatase activity; organic cyclic compound binding; heterocyclic compound binding; ribonucleoside binding; carbohydrate derivative binding; pyrophosphatase activity; catalytic activity; ribonucleotide binding; binding; purine ribonucleoside binding; anion binding; purine ribonucleotide binding; adenyl ribonucleotide binding; adenyl nucleotide binding; purine nucleoside binding; hydrolase activity, acting on acid anhydrides, in phosphorus-containing anhydrides; purine nucleotide binding; small molecule binding; purine ribonucleoside triphosphate binding;	K10352	MYH; myosin heavy chain	dre04530 Tight junction - Danio rerio (zebrafish);	IPR002928; IPR004009; IPR001609; IPR027417; IPR027401;	Myosin tail; Myosin, N-terminal, SH3-like; Myosin head, motor domain; P-loop containing nucleoside triphosphate hydrolase; Myosin-like IQ motif-containing domain;
Q0D2W2	tnni2a.4	Fast muscle troponin I	_ADK(cr)EIEDLK_	87	1	AKVAKADKEIEDLKI	......DK.......	mitochondria								
Q0D2W2	tnni2a.4	Fast muscle troponin I	_ADKEIEDLK(cr)IK_	93	1	DKEIEDLKIKVIDLK	..E....K.......	mitochondria								
F1QZU7	aldh2.2	Uncharacterized protein (Fragment)	_ADVDK(cr)AVK_	77	1	GDKADVDKAVKAARD	......DK.......	cytosol			oxidoreductase activity, acting on the aldehyde or oxo group of donors, NAD or NADP as acceptor; catalytic activity; oxidoreductase activity; oxidoreductase activity, acting on the aldehyde or oxo group of donors;				IPR016161; IPR015590; IPR016162; IPR016163;	Aldehyde/histidinol dehydrogenase; Aldehyde dehydrogenase domain; Aldehyde dehydrogenase, N-terminal; Aldehyde dehydrogenase, C-terminal;

Q68EH2	ak1	Adenylate kinase isoenzyme 1	_ADVSK(cr)GYLIDGYPR_	88	1	IAKADVSKGYLIDGY		cytosol	nucleoside metabolic process; purine nucleoside metabolic process; cellular aromatic compound metabolic process; purine ribonucleoside monophosphate metabolic process; ribonucleotide metabolic process; nucleotide metabolic process; single-organism process; cellular metabolic process; phosphate-containing compound metabolic process; organic substance metabolic process; metabolic process; heterocycle metabolic process; nucleobase-containing compound metabolic process; nucleoside phosphate metabolic process; carbohydrate derivative metabolic process; ribose phosphate metabolic process; cellular nitrogen compound metabolic process; nucleobase-containing small molecule metabolic process; purine nucleoside triphosphate metabolic process; cellular process; ribonucleoside metabolic process; ATP metabolic process; small molecule metabolic process; single-organism cellular process; purine ribonucleoside triphosphate metabolic process; nitrogen compound metabolic process; organic cyclic compound metabolic process; purine-containing compound metabolic process; nucleoside triphosphate metabolic process; primary metabolic process; ribonucleoside triphosphate metabolic process; purine nucleotide metabolic	intracellular part; cell; cell part; intracellular; cytoplasm;	ATP binding; nucleoside phosphate binding; adenylate kinase activity; nucleoside binding; ion binding; nucleobase-containing compound kinase activity; nucleotide binding; nucleotide kinase activity; phosphotransferase activity, phosphate group as acceptor; organic cyclic compound binding; heterocyclic compound binding; ribonucleoside binding; carbohydrate derivative binding; kinase activity; ribonucleotide binding; catalytic activity; binding; purine ribonucleoside binding; purine ribonucleotide binding; anion binding; adenyl ribonucleotide binding; adenyl nucleotide binding; purine nucleoside binding; transferase activity; purine nucleotide binding; small molecule binding; purine ribonucleoside triphosphate binding; transferase activity, transferring phosphorus-containing groups;	K00939	adk; adenylate kinase [EC:2.7.4.3]	dre01100 Metabolic pathways - Danio rerio (zebrafish); dre00230 Purine metabolism - Danio rerio (zebrafish);	IPR027417;	P-loop containing nucleoside triphosphate hydrolase;
Q6PC53	ppiab	Peptidyl-prolyl cis-trans isomerase	_ADVVPK(cr)TAENFR_	31	1	LRADVVPKTAENFRA		cytosol	cellular protein metabolic process; protein peptidyl-prolyl isomerization; peptidyl-proline modification; primary metabolic process; cellular metabolic process; organic substance metabolic process; protein folding; peptidyl-amino acid modification; metabolic process; cellular macromolecule metabolic process; cellular protein modification process; protein metabolic process; macromolecule metabolic process; macromolecule modification; protein modification process; cellular process;		peptidyl-prolyl cis-trans isomerase activity; isomerase activity; catalytic activity; cis-trans isomerase activity;	K03767			IPR002130; IPR029000;	Cyclophilin-type peptidyl-prolyl cis-trans isomerase domain; Cyclophilin-like domain;
E7EZ16	-	Uncharacterized protein	_AEADKNDK(cr)AVK_	633	1	AEADKNDKAVKDLVI	......DK.......	cytosol	protein folding; metabolic process; response to stress; cellular protein metabolic process; cellular macromolecule metabolic process; macromolecule metabolic process; protein metabolic process; response to stimulus; primary metabolic process; cellular process; cellular metabolic process; organic substance metabolic process;		ATP binding; nucleoside phosphate binding; nucleoside binding; ion binding; nucleotide binding; organic cyclic compound binding; heterocyclic compound binding; ribonucleoside binding; carbohydrate derivative binding; ribonucleotide binding; binding; purine ribonucleoside binding; anion binding; purine ribonucleotide binding; adenyl ribonucleotide binding; adenyl nucleotide binding; purine nucleoside binding; purine nucleotide binding; small molecule binding; purine ribonucleoside triphosphate binding;	K04079	htpG; molecular chaperone HtpG	dre04914 Progesterone-mediated oocyte maturation - Danio rerio (zebrafish); dre04141 Protein processing in endoplasmic reticulum - Danio rerio (zebrafish); dre04621 NOD-like receptor signaling pathway - Danio rerio (zebrafish);	IPR020568; IPR020575; IPR003594;	Ribosomal protein S5 domain 2-type fold; Heat shock protein Hsp90, N-terminal; Histidine kinase-like ATPase, C-terminal domain;
F1QK60	krt4	Uncharacterized protein	_AEAESWYK(cr)QK_	306	1	AEAESWYKQKFEEMQ		nuclear		intracellular organelle; intracellular non-membrane-bounded organelle; cytoskeletal part; intracellular organelle part; macromolecular complex; intracellular part; cell; keratin filament; organelle part; non-membrane-bounded organelle; organelle; cytoskeleton; intermediate filament cytoskeleton; cell part; intracellular; intermediate filament; protein complex;	structural molecule activity;	K07605				
Q6NWK4	atp6v1e1b	Atp6v1e1 protein	_AEEEFNIEK(cr)GR_	42	1	EEEFNIEKGRLVQTQ	..E....K.......	cytosol	ion transmembrane transport; cation transmembrane transport; inorganic ion transmembrane transport; single-organism cellular process; energy coupled proton transmembrane transport, against electrochemical gradient; single-organism process; hydrogen transport; proton transport; establishment of localization; transport; transmembrane transport; single-organism localization; cation transport; ion transport; ATP hydrolysis coupled proton transport; hydrogen ion transmembrane transport; localization; cellular process; monovalent inorganic cation transport; inorganic cation transmembrane transport; single-organism transport;	macromolecular complex; membrane; proton-transporting two-sector ATPase complex, catalytic domain; protein complex; membrane part; proton-transporting two-sector ATPase complex;	active transmembrane transporter activity; ATPase activity, coupled to transmembrane movement of ions; ion transmembrane transporter activity; primary active transmembrane transporter activity; ATPase activity, coupled to transmembrane movement of ions, rotational mechanism; hydrolase activity, acting on acid anhydrides; substrate-specific transporter activity; monovalent inorganic cation transmembrane transporter activity; hydrolase activity; nucleoside-triphosphatase activity; hydrogen ion transmembrane transporter activity; substrate-specific transmembrane transporter activity; proton-transporting ATPase activity, rotational mechanism; ATPase activity; P-P-bond-hydrolysis-driven transmembrane transporter activity; pyrophosphatase activity; cation-transporting ATPase activity; ATPase activity, coupled to transmembrane movement of substances; catalytic activity; ATPase activity, coupled to movement of substances; ATPase activity, coupled; hydrogen-exporting ATPase activity; hydrolase activity, acting on acid anhydrides, catalyzing transmembrane movement of substances; inorganic cation transmembrane transporter activity; hydrolase activity, acting on acid anhydrides, in phosphorus-containing anhydrides; cation transmembrane transporter activity; transporter activity; transmembrane transporter activity;	K02150	ATPeV1E; V-type H+-transporting ATPase subunit E	dre01100 Metabolic pathways - Danio rerio (zebrafish); dre04145 Phagosome - Danio rerio (zebrafish); dre00190 Oxidative phosphorylation - Danio rerio (zebrafish);		
Q804G7	anxa4	Annexin	_AEIDMLDIK(cr)AEFLK_	286	1	EIDMLDIKAEFLKMY	.......K.E.....	cytosol			calcium-dependent phospholipid binding; binding; phospholipid binding; ion binding; lipid binding; metal ion binding; cation binding; anion binding; calcium ion binding;	K17093				
F1QJK4	myh9b	Uncharacterized protein	_AEMEDLVSSK(cr)DDVGK_	1512	1	MEDLVSSKDDVGKSV		nuclear		intracellular organelle; intracellular non-membrane-bounded organelle; cytoskeletal part; intracellular organelle part; macromolecular complex; intracellular part; cell; actin cytoskeleton; organelle part; non-membrane-bounded organelle; organelle; cytoskeleton; cell part; intracellular; myosin complex; protein complex;	ATP binding; motor activity; nucleoside phosphate binding; nucleoside binding; ion binding; hydrolase activity, acting on acid anhydrides; hydrolase activity; nucleotide binding; nucleoside-triphosphatase activity; organic cyclic compound binding; heterocyclic compound binding; ribonucleoside binding; carbohydrate derivative binding; pyrophosphatase activity; catalytic activity; ribonucleotide binding; binding; purine ribonucleoside binding; anion binding; purine ribonucleotide binding; adenyl ribonucleotide binding; adenyl nucleotide binding; purine nucleoside binding; hydrolase activity, acting on acid anhydrides, in phosphorus-containing anhydrides; purine nucleotide binding; small molecule binding; purine ribonucleoside triphosphate binding;	K10352	MYH; myosin heavy chain	dre04530 Tight junction - Danio rerio (zebrafish);	IPR002928; IPR004009; IPR027417; IPR001609; IPR027401;	Myosin tail; Myosin, N-terminal, SH3-like; P-loop containing nucleoside triphosphate hydrolase; Myosin head, motor domain; Myosin-like IQ motif-containing domain;

P13104	tpma	Tropomyosin alpha-1 chain	_AEQAETDK(cr)K_	29	1	AEQAETDKKAAEERS	......DK.......	cytosol		cytoskeleton; organelle; intracellular organelle; intracellular non-membrane-bounded organelle; cell part; intracellular; cytoplasm; intracellular part; cell; non-membrane-bounded organelle;		K10373	TPM1; tropomyosin 1	dre04260 Cardiac muscle contraction - Danio rerio (zebrafish); dre04261 Adrenergic signaling in cardiomyocytes - Danio rerio [illegible]		
Q6IQD7	tpm2	Uncharacterized protein	_AEVAEAK(cr)SGDLEEELK_	189	1	RAEVAEAKSGDLEEE	..E....K.......	cytosol				K10374	TPM2; tropomyosin 2	dre04260 Cardiac muscle contraction - Danio rerio (zebrafish); dre04261 Adrenergic signaling in cardiomyocytes - Danio rerio [illegible]		
Q6IMW7	pvalb4	Parvalbumin 4	_AFAIIDQDK(cr)SGFIEEEELK_	55	1	FAIIDQDKSGFIEEE	......DK.......	cytosol			cation binding; metal ion binding; calcium ion binding; binding; ion binding;				IPR011992; IPR002048;	EF-hand domain pair; EF-hand domain;
Q803M8	ywhaqb	Tryosine 3-monooxygenase/tryptophan 5-monooxygenase activation protein, zeta polypeptide	_AFDISK(cr)TEMQPTHPIR_	157	1	QKAFDISKTEMQPTH	.......K.E.....	cytosol			monooxygenase activity; catalytic activity; oxidoreductase activity;	K16197	YWHAB_Q_Z; 14-3-3 protein beta/theta/zeta	dre04110 Cell cycle - Danio rerio (zebrafish); dre04114 Oocyte meiosis - Danio rerio (zebrafish);	IPR023410;	14-3-3 domain;
O42363	apoa1	Apolipoprotein A-I	_AFESNIEETK(cr)SK_	180	1	ESNIEETKSKVVPMV		extracellular	cholesterol metabolic process; lipid transport; organic hydroxy compound metabolic process; alcohol metabolic process; small molecule metabolic process; steroid metabolic process; organic cyclic compound metabolic process; single-organism process; organic substance transport; lipid localization; primary metabolic process; sterol metabolic process; lipoprotein metabolic process; establishment of localization; organic substance metabolic process; transport; metabolic process; single-organism localization; single-organism metabolic process; macromolecule metabolic process; protein metabolic process; localization; macromolecule localization; single-organism transport; lipid metabolic process;	macromolecular complex; extracellular region part; extracellular space; high-density lipoprotein particle; protein-lipid complex; extracellular region; plasma lipoprotein particle;	lipid binding; binding;	K08757	APOA1; apolipoprotein A-I	dre03320 PPAR signaling pathway - Danio rerio (zebrafish);		
F1Q6U4	hbae3	Uncharacterized protein	_AFFDK(cr)VAPK_	17	1	NVKAFFDKVAPKAEE	......DK.......	cytosol		cell part; intracellular; hemoglobin complex; cytoplasm; macromolecular complex; intracellular part; cytosolic part; protein complex; cell; cytosol; cytoplasmic part;	organic cyclic compound binding; heterocyclic compound binding; heme binding; iron ion binding; transition metal ion binding; oxygen binding; binding; ion binding; substrate-specific transporter activity; metal ion binding; cation binding; oxygen transporter activity; tetrapyrrole binding; transporter activity;				IPR009050; IPR000971; IPR012292;	Globin-like; Globin; Globin, structural domain;
Q9I8V0	pvalb2	Parvalbumin-2	_AFFVIDQDK(cr)SGFIEEDELK_	55	1	FFVIDQDKSGFIEED	......DK.......	cytosol			cation binding; metal ion binding; calcium ion binding; binding; ion binding;				IPR002048; IPR011992;	EF-hand domain; EF-hand domain pair;
Q804W0	pvalb1	Parvalbumin 1	_AFLSAGDSDGDGK(cr)IGAEEFAALVK_	97	1	GDSDGDGKIGAEEFA		cytosol			cation binding; metal ion binding; calcium ion binding; binding; ion binding;				IPR011992; IPR002048;	EF-hand domain pair; EF-hand domain;
Q9I8V0	pvalb2	Parvalbumin-2	_AFLSAGDSDGDGK(cr)IGVDEFALLVK_	97	1	GDSDGDGKIGVDEFA		cytosol			cation binding; metal ion binding; calcium ion binding; binding; ion binding;				IPR002048; IPR011992;	EF-hand domain; EF-hand domain pair;
E7F3J2	hnrnpua	Uncharacterized protein	_AFTVSK(cr)DELK_	345	1	GKAFTVSKDELKGKP	.......K.E.....	nuclear		organelle; intracellular membrane-bounded organelle; intracellular organelle; nucleus; cell part; intracellular; intracellular part; membrane-bounded organelle; cell;	organic cyclic compound binding; heterocyclic compound binding; binding; nucleic acid binding;	K12888	HNRNPU; heterogeneous nuclear ribonucleoprotein U	dre03040 Spliceosome - Danio rerio (zebrafish);	IPR003034; IPR027417; IPR001870; IPR003877;	SAP domain; P-loop containing nucleoside triphosphate hydrolase; B30.2/SPRY domain; SPRY domain;
B8A561	myhz1.2	Uncharacterized protein	_AITDAAM(ox)M(ox)AEELK(cr)K_	1772	1	AMMAEELKKEQDTSA	.......K.E.....	nuclear		intracellular organelle; intracellular non-membrane-bounded organelle; cytoskeletal part; intracellular organelle part; macromolecular complex; intracellular part; cell; actin cytoskeleton; organelle part; non-membrane-bounded organelle; organelle; cytoskeleton; cell part; intracellular; myosin complex; protein complex;	ATP binding; motor activity; nucleoside phosphate binding; nucleoside binding; ion binding; hydrolase activity, acting on acid anhydrides; hydrolase activity; nucleotide binding; nucleoside-triphosphatase activity; organic cyclic compound binding; heterocyclic compound binding; ribonucleoside binding; carbohydrate derivative binding; pyrophosphatase activity; catalytic activity; ribonucleotide binding; binding; purine ribonucleoside binding; anion binding; purine ribonucleotide binding; adenyl ribonucleotide binding; adenyl nucleotide binding; purine nucleoside binding; hydrolase activity, acting on acid anhydrides, in phosphorus-containing anhydrides; purine nucleotide binding; small molecule binding; purine ribonucleoside triphosphate binding;	K10352	MYH; myosin heavy chain	dre04530 Tight junction - Danio rerio (zebrafish);	IPR002928; IPR004009; IPR001609; IPR027417; IPR027401;	Myosin tail; Myosin, N-terminal, SH3-like; Myosin head, motor domain; P-loop containing nucleoside triphosphate hydrolase; Myosin-like IQ motif-containing domain;
B8A568	myhz1.1	Uncharacterized protein	_AITDAAMMAEELK(cr)K_	1772	1	AMMAEELKKEQDTSA	.......K.E.....	nuclear		intracellular organelle; intracellular non-membrane-bounded organelle; cytoskeletal part; intracellular organelle part; macromolecular complex; intracellular part; cell; actin cytoskeleton; organelle part; non-membrane-bounded organelle; organelle; cytoskeleton; cell part; intracellular; myosin complex; protein complex;	ATP binding; motor activity; nucleoside phosphate binding; nucleoside binding; ion binding; hydrolase activity, acting on acid anhydrides; hydrolase activity; nucleotide binding; nucleoside-triphosphatase activity; organic cyclic compound binding; heterocyclic compound binding; ribonucleoside binding; carbohydrate derivative binding; pyrophosphatase activity; catalytic activity; ribonucleotide binding; binding; purine ribonucleoside binding; anion binding; purine ribonucleotide binding; adenyl ribonucleotide binding; adenyl nucleotide binding; purine nucleoside binding; hydrolase activity, acting on acid anhydrides, in phosphorus-containing anhydrides; purine nucleotide binding; small molecule binding; purine ribonucleoside triphosphate binding;	K10352	MYH; myosin heavy chain	dre04530 Tight junction - Danio rerio (zebrafish);	IPR002928; IPR004009; IPR001609; IPR027417; IPR027401;	Myosin tail; Myosin, N-terminal, SH3-like; Myosin head, motor domain; P-loop containing nucleoside triphosphate hydrolase; Myosin-like IQ motif-containing domain;
B8A561	myhz1.2	Uncharacterized protein	_AITDAAMMAEELK(cr)K_	1772	1	AMMAEELKKEQDTSA	.......K.E.....	nuclear		intracellular organelle; intracellular non-membrane-bounded organelle; cytoskeletal part; intracellular organelle part; macromolecular complex; intracellular part; cell; actin cytoskeleton; organelle part; non-membrane-bounded organelle; organelle; cytoskeleton; cell part; intracellular; myosin complex; protein complex;	ATP binding; motor activity; nucleoside phosphate binding; nucleoside binding; ion binding; hydrolase activity, acting on acid anhydrides; hydrolase activity; nucleotide binding; nucleoside-triphosphatase activity; organic cyclic compound binding; heterocyclic compound binding; ribonucleoside binding; carbohydrate derivative binding; pyrophosphatase activity; catalytic activity; ribonucleotide binding; binding; purine ribonucleoside binding; anion binding; purine ribonucleotide binding; adenyl ribonucleotide binding; adenyl nucleotide binding; purine nucleoside binding; hydrolase activity, acting on acid anhydrides, in phosphorus-containing anhydrides; purine nucleotide binding; small molecule binding; purine ribonucleoside triphosphate binding;	K10352	MYH; myosin heavy chain	dre04530 Tight junction - Danio rerio (zebrafish);	IPR002928; IPR004009; IPR001609; IPR027417; IPR027401;	Myosin tail; Myosin, N-terminal, SH3-like; Myosin head, motor domain; P-loop containing nucleoside triphosphate hydrolase; Myosin-like IQ motif-containing domain;

A5WWJ4	matn1	Uncharacterized protein	_AK(cr)ALGFK_	393	1	GDAAKKAKALGFKMY	.......K.L.....	extracellular			cation binding; metal ion binding; calcium ion binding; binding; ion binding;				IPR019466; IPR000742; IPR002035;	Matrilin, coiled-coil trimerisation domain; Epidermal growth factor-like domain; von Willebrand factor, type A;
B8A568	myhz1.1	Uncharacterized protein	_AK(cr)ANLEK_	1244	1	MEAVAKAKANLEKMC		nuclear		intracellular organelle; intracellular non-membrane-bounded organelle; cytoskeletal part; intracellular organelle part; macromolecular complex; intracellular part; cell; actin cytoskeleton; organelle part; non-membrane-bounded organelle; organelle; cytoskeleton; cell part; intracellular; myosin complex; protein complex;	ATP binding; motor activity; nucleoside phosphate binding; nucleoside binding; ion binding; hydrolase activity, acting on acid anhydrides; hydrolase activity; nucleotide binding; nucleoside-triphosphatase activity; organic cyclic compound binding; heterocyclic compound binding; ribonucleoside binding; carbohydrate derivative binding; pyrophosphatase activity; catalytic activity; ribonucleotide binding; binding; purine ribonucleoside binding; anion binding; purine ribonucleotide binding; adenyl ribonucleotide binding; adenyl nucleotide binding; purine nucleoside binding; hydrolase activity, acting on acid anhydrides, in phosphorus-containing anhydrides; purine nucleotide binding; small molecule binding; purine ribonucleoside triphosphate binding;	K10352	MYH; myosin heavy chain	dre04530 Tight junction - Danio rerio (zebrafish);	IPR002928; IPR004009; IPR001609; IPR027417; IPR027401;	Myosin tail; Myosin, N-terminal, SH3-like; Myosin head, motor domain; P-loop containing nucleoside triphosphate hydrolase; Myosin-like IQ motif-containing domain;
B8A561	myhz1.2	Uncharacterized protein	_AK(cr)ANLEK_	1244	1	MEAVAKAKANLEKMC		nuclear		intracellular organelle; intracellular non-membrane-bounded organelle; cytoskeletal part; intracellular organelle part; macromolecular complex; intracellular part; cell; actin cytoskeleton; organelle part; non-membrane-bounded organelle; organelle; cytoskeleton; cell part; intracellular; myosin complex; protein complex;	ATP binding; motor activity; nucleoside phosphate binding; nucleoside binding; ion binding; hydrolase activity, acting on acid anhydrides; hydrolase activity; nucleotide binding; nucleoside-triphosphatase activity; organic cyclic compound binding; heterocyclic compound binding; ribonucleoside binding; carbohydrate derivative binding; pyrophosphatase activity; catalytic activity; ribonucleotide binding; binding; purine ribonucleoside binding; anion binding; purine ribonucleotide binding; adenyl ribonucleotide binding; adenyl nucleotide binding; purine nucleoside binding; hydrolase activity, acting on acid anhydrides, in phosphorus-containing anhydrides; purine nucleotide binding; small molecule binding; purine ribonucleoside triphosphate binding;	K10352	MYH; myosin heavy chain	dre04530 Tight junction - Danio rerio (zebrafish);	IPR002928; IPR004009; IPR001609; IPR027417; IPR027401;	Myosin tail; Myosin, N-terminal, SH3-like; Myosin head, motor domain; P-loop containing nucleoside triphosphate hydrolase; Myosin-like IQ motif-containing domain;
Q6PBZ1	rpl7a	Uncharacterized protein	_AK(cr)ELATK_	259	1	KLEKAKAKELATKLG	.......K.L.....	plasma membrane	cellular component biogenesis; ribosome biogenesis; ribonucleoprotein complex biogenesis; cellular component organization or biogenesis;	ribosome; organelle; intracellular organelle; intracellular non-membrane-bounded organelle; cell part; intracellular; cytoplasm; macromolecular complex; intracellular part; cell; ribonucleoprotein complex; cytoplasmic part; non-membrane-bounded organelle;		K02936	RP-L7Ae; large subunit ribosomal protein L7Ae	dre03010 Ribosome - Danio rerio (zebrafish);	IPR029064; IPR004038;	50S ribosomal protein L30e-like; Ribosomal protein L7Ae/L30e/S12e/ Gadd45;
Q6YI49	uchl1	Ubiquitin carboxyl-terminal hydrolase	_AK(cr)ELEQNK_	126	1	MSPAERAKELEQNKA	.......K.L.....	cytosol	modification-dependent macromolecule catabolic process; cellular macromolecule catabolic process; proteolysis involved in cellular protein catabolic process; cellular protein catabolic process; modification-dependent protein catabolic process; cellular protein metabolic process; ubiquitin-dependent protein catabolic process; primary metabolic process; cellular metabolic process; organic substance metabolic process; organic substance catabolic process; metabolic process; protein catabolic process; macromolecule catabolic process; cellular macromolecule metabolic process; cellular catabolic process; proteolysis; protein metabolic process; macromolecule metabolic process; cellular process; catabolic process;	cell; intracellular; cell part;		K05611			IPR001578;	Peptidase C12, ubiquitin carboxyl-terminal hydrolase;
Q5TZ29	apobb.1	Uncharacterized protein	_AK(cr)QLLNLLGNR_	2161	1	SKILEKAKQLLNLLG	.......K.L.....	endoplasmic reticulum	response to chemical; response to stimulus;		lipid transporter activity; substrate-specific transporter activity; transporter activity;				IPR011030; IPR015817; IPR015818; IPR001747; IPR015819; IPR015816; IPR016024; IPR015255; IPR009454;	Vitellinogen, superhelical; Vitellinogen, open beta-sheet, subdomain 1; Vitellinogen, open beta-sheet, subdomain 2; Lipid transport protein, N-terminal; Lipid transport protein, beta-sheet shell; Vitellinogen, beta-sheet N-terminal; Armadillo-type fold; Vitellinogen, open beta-sheet; Lipid transport, open beta-sheet;
P79735	ran	GTP-binding nuclear protein Ran	_AK(cr)SIVFHR_	133	1	KDRKVKAKSIVFHRK	.......K.I.....	cytosol	regulation of cellular process; single organism signaling; cellular response to stimulus; intracellular transport; intracellular signal transduction; single-organism cellular process; single-organism process; nuclear transport; organic substance transport; small GTPase mediated signal transduction; signaling; response to stimulus; protein transport; establishment of localization; cellular localization; transport; establishment of protein localization; signal transduction; cellular macromolecule localization; regulation of biological process; establishment of localization in cell; cell communication; intracellular protein transport; nucleocytoplasmic transport; cytoplasmic transport; localization; cellular protein localization; macromolecule localization; protein localization; cellular process; biological regulation;	organelle; intracellular membrane-bounded organelle; intracellular organelle; nucleus; cell part; intracellular; intracellular part; membrane-bounded organelle; cell;	nucleoside phosphate binding; nucleoside binding; ion binding; hydrolase activity, acting on acid anhydrides; hydrolase activity; nucleotide binding; nucleoside-triphosphatase activity; organic cyclic compound binding; heterocyclic compound binding; ribonucleoside binding; carbohydrate derivative binding; pyrophosphatase activity; catalytic activity; ribonucleotide binding; binding; GTPase activity; purine ribonucleoside binding; anion binding; purine ribonucleotide binding; purine nucleoside binding; guanyl ribonucleotide binding; hydrolase activity, acting on acid anhydrides, in phosphorus-containing anhydrides; guanyl nucleotide binding; GTP binding; purine nucleotide binding; small molecule binding; purine ribonucleoside triphosphate binding;	K07936	RAN; GTP-binding nuclear protein Ran	dre03013 RNA transport - Danio rerio (zebrafish); dre03008 Ribosome biogenesis in eukaryotes - Danio rerio (zebrafish);	IPR005225; IPR027417;	Small GTP-binding protein domain; P-loop containing nucleoside triphosphate hydrolase;

E9QEQ1	apoc2	Uncharacterized protein	_AK(cr)TIYSDTVR_	69	1	YKLEEKAKTIYSDTV	.......K.I.....	extracellular	single-organism process; lipoprotein metabolic process; establishment of localization; organic substance metabolic process; metabolic process; single-organism localization; positive regulation of metabolic process; positive regulation of molecular function; protein metabolic process; macromolecule metabolic process; lipoprotein catabolic process; macromolecule localization; lipid metabolic process; single-organism transport; lipid transport; lipid localization; organic substance transport; primary metabolic process; positive regulation of biological process; positive regulation of catalytic activity; transport; organic substance catabolic process; regulation of biological process; single-organism metabolic process; protein catabolic process; regulation of catalytic activity; macromolecule catabolic process; regulation of metabolic process; regulation of molecular function; localization; catabolic process; biological regulation;	macromolecular complex; extracellular region part; extracellular space; chylomicron; protein-lipid complex; extracellular region; plasma lipoprotein particle;	enzyme regulator activity; molecular function regulator; enzyme activator activity;				IPR023121;	ApoC-II domain;
Q6P027	rlbp1b	Cellular retinaldehyde-binding protein b	_AKDELNETDEK(cr)R_	58	1	ELNETDEKRTSAVKE		cytosol	behavior; single-multicellular organism process; response to light stimulus; visual behavior; response to abiotic stimulus; single-organism process; response to radiation; response to stimulus; optokinetic behavior; multicellular organismal process; single-organism behavior;	cell; intracellular; cell part;	transporter activity;				IPR001251; IPR011074;	CRAL-TRIO domain; CRAL/TRIO, N-terminal domain;
B8JKH7	mylz3	Uncharacterized protein	_ALGQNPTNK(cr)DVK_	71	1	LGQNPTNKDVKKILG	.......K.V.....	mitochondria			cation binding; metal ion binding; calcium ion binding; binding; ion binding;				IPR002048; IPR011992;	EF-hand domain; EF-hand domain pair;
Q9I8U7	mylz3	Fast skeletal muscle myosin light polypeptide 3	_ALGQNPTNK(cr)DVK_	47	1	LGQNPTNKDVKKILG	.......K.V.....	cytosol			cation binding; metal ion binding; calcium ion binding; binding; ion binding;				IPR011992; IPR002048;	EF-hand domain pair; EF-hand domain;
Q6P0G6	myl1	Uncharacterized protein	_ALGQNPTNK(cr)EVTK_	85	1	LGQNPTNKEVTKILG	.......K.V.....	cytosol			cation binding; metal ion binding; calcium ion binding; binding; ion binding;	K05738			IPR002048; IPR011992;	EF-hand domain; EF-hand domain pair;
P13104	tpma	Uncharacterized protein	_ALK(cr)DEEK_	136	1	VIENRALKDEEKMEL	..E....K.......	cytosol		cytoskeleton; organelle; intracellular organelle; intracellular non-membrane-bounded organelle; cell part; intracellular; cytoplasm; intracellular part; cell; non-membrane-bounded organelle;		K10373	TPM1; tropomyosin 1	dre04260 Cardiac muscle contraction - Danio rerio (zebrafish); dre04261 Adrenergic signaling in cardiomyocytes - Danio rerio [illegible]		
B8A568	myhz1.1	Uncharacterized protein	_ALQEAHQQTLDDLQAEEDK(cr)VNTLTK_	1018	1	DLQAEEDKVNTLTKS	......DK.......	nuclear		intracellular organelle; intracellular non-membrane-bounded organelle; cytoskeletal part; intracellular organelle part; macromolecular complex; intracellular part; cell; actin cytoskeleton; organelle part; non-membrane-bounded organelle; organelle; cytoskeleton; cell part; intracellular; myosin complex; protein complex;	ATP binding; motor activity; nucleoside phosphate binding; nucleoside binding; ion binding; hydrolase activity, acting on acid anhydrides; hydrolase activity; nucleotide binding; nucleoside-triphosphatase activity; organic cyclic compound binding; heterocyclic compound binding; ribonucleoside binding; carbohydrate derivative binding; pyrophosphatase activity; catalytic activity; ribonucleotide binding; binding; purine ribonucleoside binding; anion binding; purine ribonucleotide binding; adenyl ribonucleotide binding; adenyl nucleotide binding; purine nucleoside binding; hydrolase activity, acting on acid anhydrides, in phosphorus-containing anhydrides; purine nucleotide binding; small molecule binding; purine ribonucleoside triphosphate binding;	K10352	MYH; myosin heavy chain	dre04530 Tight junction - Danio rerio (zebrafish);	IPR002928; IPR004009; IPR001609; IPR027417; IPR027401;	Myosin tail; Myosin, N-terminal, SH3-like; Myosin head, motor domain; P-loop containing nucleoside triphosphate hydrolase; Myosin-like IQ motif-containing domain;
B8A561	myhz1.2	Uncharacterized protein	_ALQEAHQQTLDDLQAEEDK(cr)VNTLTK_	1018	1	DLQAEEDKVNTLTKS	......DK.......	nuclear		intracellular organelle; intracellular non-membrane-bounded organelle; cytoskeletal part; intracellular organelle part; macromolecular complex; intracellular part; cell; actin cytoskeleton; organelle part; non-membrane-bounded organelle; organelle; cytoskeleton; cell part; intracellular; myosin complex; protein complex;	ATP binding; motor activity; nucleoside phosphate binding; nucleoside binding; ion binding; hydrolase activity, acting on acid anhydrides; hydrolase activity; nucleotide binding; nucleoside-triphosphatase activity; organic cyclic compound binding; heterocyclic compound binding; ribonucleoside binding; carbohydrate derivative binding; pyrophosphatase activity; catalytic activity; ribonucleotide binding; binding; purine ribonucleoside binding; anion binding; purine ribonucleotide binding; adenyl ribonucleotide binding; adenyl nucleotide binding; purine nucleoside binding; hydrolase activity, acting on acid anhydrides, in phosphorus-containing anhydrides; purine nucleotide binding; small molecule binding; purine ribonucleoside triphosphate binding;	K10352	MYH; myosin heavy chain	dre04530 Tight junction - Danio rerio (zebrafish);	IPR002928; IPR004009; IPR001609; IPR027417; IPR027401;	Myosin tail; Myosin, N-terminal, SH3-like; Myosin head, motor domain; P-loop containing nucleoside triphosphate hydrolase; Myosin-like IQ motif-containing domain;
B8A568	myhz1.1	Uncharacterized protein	_ALQEAHQQTLDDLQAEEDKVNTLTK(cr)SK_	1024	1	DKVNTLTKSKTKLEQ		nuclear		intracellular organelle; intracellular non-membrane-bounded organelle; cytoskeletal part; intracellular organelle part; macromolecular complex; intracellular part; cell; actin cytoskeleton; organelle part; non-membrane-bounded organelle; organelle; cytoskeleton; cell part; intracellular; myosin complex; protein complex;	ATP binding; motor activity; nucleoside phosphate binding; nucleoside binding; ion binding; hydrolase activity, acting on acid anhydrides; hydrolase activity; nucleotide binding; nucleoside-triphosphatase activity; organic cyclic compound binding; heterocyclic compound binding; ribonucleoside binding; carbohydrate derivative binding; pyrophosphatase activity; catalytic activity; ribonucleotide binding; binding; purine ribonucleoside binding; anion binding; purine ribonucleotide binding; adenyl ribonucleotide binding; adenyl nucleotide binding; purine nucleoside binding; hydrolase activity, acting on acid anhydrides, in phosphorus-containing anhydrides; purine nucleotide binding; small molecule binding; purine ribonucleoside triphosphate binding;	K10352	MYH; myosin heavy chain	dre04530 Tight junction - Danio rerio (zebrafish);	IPR002928; IPR004009; IPR001609; IPR027417; IPR027401;	Myosin tail; Myosin, N-terminal, SH3-like; Myosin head, motor domain; P-loop containing nucleoside triphosphate hydrolase; Myosin-like IQ motif-containing domain;
B8A561	myhz1.2	Uncharacterized protein	_ALQEAHQQTLDDLQAEEDKVNTLTK(cr)SK_	1024	1	DKVNTLTKSKTKLEQ		nuclear		intracellular organelle; intracellular non-membrane-bounded organelle; cytoskeletal part; intracellular organelle part; macromolecular complex; intracellular part; cell; actin cytoskeleton; organelle part; non-membrane-bounded organelle; organelle; cytoskeleton; cell part; intracellular; myosin complex; protein complex;	ATP binding; motor activity; nucleoside phosphate binding; nucleoside binding; ion binding; hydrolase activity, acting on acid anhydrides; hydrolase activity; nucleotide binding; nucleoside-triphosphatase activity; organic cyclic compound binding; heterocyclic compound binding; ribonucleoside binding; carbohydrate derivative binding; pyrophosphatase activity; catalytic activity; ribonucleotide binding; binding; purine ribonucleoside binding; anion binding; purine ribonucleotide binding; adenyl ribonucleotide binding; adenyl nucleotide binding; purine nucleoside binding; hydrolase activity, acting on acid anhydrides, in phosphorus-containing anhydrides; purine nucleotide binding; small molecule binding; purine ribonucleoside triphosphate binding;	K10352	MYH; myosin heavy chain	dre04530 Tight junction - Danio rerio (zebrafish);	IPR002928; IPR004009; IPR001609; IPR027417; IPR027401;	Myosin tail; Myosin, N-terminal, SH3-like; Myosin head, motor domain; P-loop containing nucleoside triphosphate hydrolase; Myosin-like IQ motif-containing domain;
Q9I8V0	pvalb2	Parvalbumin-2	_ALTDAETK(cr)AFLSAGDSDGDGK_	84	1	ALTDAETKAFLSAGD		cytosol			cation binding; metal ion binding; calcium ion binding; binding; ion binding;				IPR002048; IPR011992;	EF-hand domain; EF-hand domain pair;

Q6IMW7	pvalb4	Parvalbumin 4	_ALTDGETK(cr)TFLK_	84	1	ALTDGETKTFLKAGD		cytosol			cation binding; metal ion binding; calcium ion binding; binding; ion binding;				IPR011992; IPR002048;	EF-hand domain pair; EF-hand domain;
E9QDY3	cyt1	Uncharacterized protein	_ANADLELK(cr)IR_	134	1	ANADLELKIRQFLDS		mitochondria		intracellular organelle; intracellular non-membrane-bounded organelle; cytoskeletal part; intracellular organelle part; macromolecular complex; intracellular part; cell; organelle part; non-membrane-bounded organelle; organelle; cytoskeleton; intermediate filament cytoskeleton; cell part; intracellular; intermediate filament; protein complex;	structural molecule activity;	K07604				
B8A568	myhz1.1	Uncharacterized protein	_ANALAANLDK(cr)K_	1446	1	ALAANLDKKQRNFDK	......DK.......	nuclear		intracellular organelle; intracellular non-membrane-bounded organelle; cytoskeletal part; intracellular organelle part; macromolecular complex; intracellular part; cell; actin cytoskeleton; organelle part; non-membrane-bounded organelle; organelle; cytoskeleton; cell part; intracellular; myosin complex; protein complex;	ATP binding; motor activity; nucleoside phosphate binding; nucleoside binding; ion binding; hydrolase activity, acting on acid anhydrides; hydrolase activity; nucleotide binding; nucleoside-triphosphatase activity; organic cyclic compound binding; heterocyclic compound binding; ribonucleoside binding; carbohydrate derivative binding; pyrophosphatase activity; catalytic activity; ribonucleotide binding; binding; purine ribonucleoside binding; anion binding; purine ribonucleotide binding; adenyl ribonucleotide binding; adenyl nucleotide binding; purine nucleoside binding; hydrolase activity, acting on acid anhydrides, in phosphorus-containing anhydrides; purine nucleotide binding; small molecule binding; purine ribonucleoside triphosphate binding;	K10352	MYH; myosin heavy chain	dre04530 Tight junction - Danio rerio (zebrafish);	IPR002928; IPR004009; IPR001609; IPR027417; IPR027401;	Myosin tail; Myosin, N-terminal, SH3-like; Myosin head, motor domain; P-loop containing nucleoside triphosphate hydrolase; Myosin-like IQ motif-containing domain;
B8A561	myhz1.2	Uncharacterized protein	_ANALAANLDK(cr)K_	1446	1	ALAANLDKKQRNFDK	......DK.......	nuclear		intracellular organelle; intracellular non-membrane-bounded organelle; cytoskeletal part; intracellular organelle part; macromolecular complex; intracellular part; cell; actin cytoskeleton; organelle part; non-membrane-bounded organelle; organelle; cytoskeleton; cell part; intracellular; myosin complex; protein complex;	ATP binding; motor activity; nucleoside phosphate binding; nucleoside binding; ion binding; hydrolase activity, acting on acid anhydrides; hydrolase activity; nucleotide binding; nucleoside-triphosphatase activity; organic cyclic compound binding; heterocyclic compound binding; ribonucleoside binding; carbohydrate derivative binding; pyrophosphatase activity; catalytic activity; ribonucleotide binding; binding; purine ribonucleoside binding; anion binding; purine ribonucleotide binding; adenyl ribonucleotide binding; adenyl nucleotide binding; purine nucleoside binding; hydrolase activity, acting on acid anhydrides, in phosphorus-containing anhydrides; purine nucleotide binding; small molecule binding; purine ribonucleoside triphosphate binding;	K10352	MYH; myosin heavy chain	dre04530 Tight junction - Danio rerio (zebrafish);	IPR002928; IPR004009; IPR001609; IPR027417; IPR027401;	Myosin tail; Myosin, N-terminal, SH3-like; Myosin head, motor domain; P-loop containing nucleoside triphosphate hydrolase; Myosin-like IQ motif-containing domain;
Q0D2W2	tnni2a.4	Troponin I, skeletal, fast 2b.2	_ANLK(cr)QVK_	138	1	MDLRANLKQVKKEVK	.......K.V.....	mitochondria								
A8KB78	rps23	Uncharacterized protein	_ANPFGGASHAK(cr)GIVLEK_	48	1	FGGASHAKGIVLEKV	.......K.I.....	cytosol	cellular protein metabolic process; biosynthetic process; primary metabolic process; cellular metabolic process; organic substance biosynthetic process; cellular biosynthetic process; organic substance metabolic process; metabolic process; cellular macromolecule biosynthetic process; cellular macromolecule metabolic process; gene expression; macromolecule metabolic process; protein metabolic process; macromolecule biosynthetic process; translation; cellular process;	intracellular organelle; ribosome; intracellular non-membrane-bounded organelle; small ribosomal subunit; cytoplasm; intracellular organelle part; macromolecular complex; intracellular part; cell; organelle part; non-membrane-bounded organelle; organelle; ribosomal subunit; cell part; intracellular; ribonucleoprotein complex; cytoplasmic part;	structural constituent of ribosome; structural molecule activity;	K02973	RP-S23e; small subunit ribosomal protein S23e	dre03010 Ribosome - Danio rerio (zebrafish);	IPR012340;	Nucleic acid-binding, OB-fold;
F1Q6U4	hbae3	Uncharacterized protein	_ANVK(cr)AFFDK_	12	1	AKDKANVKAFFDKVA		cytosol		cell part; intracellular; hemoglobin complex; cytoplasm; macromolecular complex; intracellular part; cytosolic part; protein complex; cell; cytosol; cytoplasmic part;	organic cyclic compound binding; heterocyclic compound binding; heme binding; iron ion binding; transition metal ion binding; oxygen binding; binding; ion binding; substrate-specific transporter activity; metal ion binding; cation binding; oxygen transporter activity; tetrapyrrole binding; transporter activity;				IPR009050; IPR000971; IPR012292;	Globin-like; Globin; Globin, structural domain;
Q642Z0	atp2a1	Calcium-transporting ATPase	_APVGNK(cr)M(ox)FVK_	511	1	SKAPVGNKMFVKGAP		plasma membrane	nucleoside metabolic process; purine nucleoside metabolic process; purine ribonucleoside monophosphate metabolic process; nucleotide metabolic process; single-organism process; purine ribonucleoside biosynthetic process; nucleoside biosynthetic process; biosynthetic process; ribonucleoside monophosphate biosynthetic process; cellular metabolic process; phosphate-containing compound metabolic process; ribose phosphate biosynthetic process; metabolic process; carbohydrate derivative biosynthetic process; nucleoside phosphate metabolic process; ribose phosphate metabolic process; purine nucleoside triphosphate biosynthetic process; heterocycle biosynthetic process; purine ribonucleoside triphosphate biosynthetic process; small molecule metabolic process; ATP biosynthetic process; single-organism cellular process; purine ribonucleoside triphosphate metabolic process; organic cyclic compound metabolic process; purine-containing compound metabolic process; ribonucleoside triphosphate metabolic process; purine ribonucleoside monophosphate biosynthetic process; purine nucleoside biosynthetic process; organophosphate biosynthetic process; ribonucleoside monophosphate	intrinsic component of membrane; membrane; integral component of membrane; membrane part;	ATPase activity, coupled to transmembrane movement of ions; nucleoside phosphate binding; primary active transmembrane transporter activity; substrate-specific transporter activity; hydrolase activity, acting on acid anhydrides; hydrolase activity; substrate-specific transmembrane transporter activity; ATPase activity; organic cyclic compound binding; heterocyclic compound binding; metal ion transmembrane transporter activity; pyrophosphatase activity; cation-transporting ATPase activity; catalytic activity; ribonucleotide binding; ATPase activity, coupled to movement of substances; binding; purine ribonucleoside binding; cation binding; hydrolase activity, acting on acid anhydrides, catalyzing transmembrane movement of substances; anion binding; adenyl ribonucleotide binding; adenyl nucleotide binding; inorganic cation transmembrane transporter activity; hydrolase activity, acting on acid anhydrides, in phosphorus-containing anhydrides; transporter activity; purine nucleotide binding; purine ribonucleoside triphosphate binding; active transmembrane transporter activity; ATP binding; ion transmembrane transporter activity; ion binding; nucleoside binding; nucleotide binding; nucleoside-triphosphatase activity; ribonucleoside binding; P-P-bond-hydrolysis-driven transmembrane transporter activity; ATPase activity, coupled to transmembrane movement of ions, phosphorylative mechanism; divalent inorganic cation transmembrane transporter activity; carbohydrate derivative binding; calcium-transporting ATPase activity; ATPase activity, coupled to transmembrane movement of substances; ATPase activity, coupled; metal ion binding; calcium ion transmembrane transporter activity; purine ribonucleotide binding; purine nucleoside binding; cation transmembrane transporter activity; transmembrane transporter activity; small molecule binding;	K05853	ATP2A; Ca2+ transporting ATPase, sarcoplasmic/endoplasmic reticulum [EC:3.6.3.8]	dre04020 Calcium signaling pathway - Danio rerio (zebrafish);	IPR023298; IPR004014; IPR023299; IPR008250; IPR006068; IPR023214;	P-type ATPase, transmembrane domain; Cation-transporting P-type ATPase, N-terminal; P-type ATPase, cytoplasmic domain N; P-type ATPase, A domain; Cation-transporting P-type ATPase, C-terminal; HAD-like domain;

Q642Z0	atp2a1	Calcium-transporting ATPase	_APVGNK(cr)MFVK_	511	1	SKAPVGNKMFVKGAP		plasma membrane	nucleoside metabolic process; purine nucleoside metabolic process; purine ribonucleoside monophosphate metabolic process; nucleotide metabolic process; single-organism process; purine ribonucleoside biosynthetic process; nucleoside biosynthetic process; biosynthetic process; ribonucleoside monophosphate biosynthetic process; cellular metabolic process; phosphate-containing compound metabolic process; ribose phosphate biosynthetic process; metabolic process; carbohydrate derivative biosynthetic process; nucleoside phosphate metabolic process; ribose phosphate metabolic process; purine nucleoside triphosphate biosynthetic process; heterocycle biosynthetic process; purine ribonucleoside triphosphate biosynthetic process; small molecule metabolic process; ATP biosynthetic process; single-organism cellular process; purine ribonucleoside triphosphate metabolic process; organic cyclic compound metabolic process; purine-containing compound metabolic process; ribonucleoside triphosphate metabolic process; purine ribonucleoside monophosphate biosynthetic process; purine nucleoside biosynthetic process; organophosphate biosynthetic process; ribonucleoside monophosphate	intrinsic component of membrane; membrane; integral component of membrane; membrane part;	ATPase activity, coupled to transmembrane movement of ions; nucleoside phosphate binding; primary active transmembrane transporter activity; substrate-specific transporter activity; hydrolase activity, acting on acid anhydrides; hydrolase activity; substrate-specific transmembrane transporter activity; ATPase activity; organic cyclic compound binding; heterocyclic compound binding; metal ion transmembrane transporter activity; pyrophosphatase activity; cation-transporting ATPase activity; catalytic activity; ribonucleotide binding; ATPase activity, coupled to movement of substances; binding; purine ribonucleoside binding; cation binding; hydrolase activity, acting on acid anhydrides, catalyzing transmembrane movement of substances; anion binding; adenyl ribonucleotide binding; adenyl nucleotide binding; inorganic cation transmembrane transporter activity; hydrolase activity, acting on acid anhydrides, in phosphorus-containing anhydrides; transporter activity; purine nucleotide binding; purine ribonucleoside triphosphate binding; active transmembrane transporter activity; ATP binding; ion transmembrane transporter activity; ion binding; nucleoside binding; nucleotide binding; nucleoside-triphosphatase activity; ribonucleoside binding; P-P-bond-hydrolysis-driven transmembrane transporter activity; ATPase activity, coupled to transmembrane movement of ions, phosphorylative mechanism; divalent inorganic cation transmembrane transporter activity; carbohydrate derivative binding; calcium-transporting ATPase activity; ATPase activity, coupled to transmembrane movement of substances; ATPase activity, coupled; metal ion binding; calcium ion transmembrane transporter activity; purine ribonucleotide binding; purine nucleoside binding; cation transmembrane transporter activity; transmembrane transporter activity; small molecule binding;	K05853	ATP2A; Ca2+ transporting ATPase, sarcoplasmic/endoplasmic reticulum [EC:3.6.3.8]	dre04020 Calcium signaling pathway - Danio rerio (zebrafish);	IPR023298; IPR004014; IPR023299; IPR008250; IPR006068; IPR023214;	P-type ATPase, transmembrane domain; Cation-transporting P-type ATPase, N-terminal; P-type ATPase, cytoplasmic domain N; P-type ATPase, A domain; Cation-transporting P-type ATPase, C-terminal; HAD-like domain;
Q5U3A4	atp2a1	Calcium-transporting ATPase	_APVGNK(cr)MFVK_	511	1	SKAPVGNKMFVKGAP		plasma membrane	regulation of biological quality; nucleoside metabolic process; purine nucleoside metabolic process; cellular divalent inorganic cation homeostasis; purine ribonucleoside monophosphate metabolic process; nucleotide metabolic process; positive regulation of muscle contraction; negative regulation of multicellular organismal process; single-organism process; purine ribonucleoside biosynthetic process; nucleoside biosynthetic process; biosynthetic process; ribonucleoside monophosphate biosynthetic process; cellular metabolic process; phosphate-containing compound metabolic process; response to external stimulus; ribose phosphate biosynthetic process; metabolic process; carbohydrate derivative biosynthetic process; positive regulation of multicellular organismal process; nucleoside phosphate metabolic process; regulation of skeletal muscle contraction; metal ion homeostasis; regulation of system process; ribose phosphate metabolic process; purine nucleoside triphosphate biosynthetic process; heterocycle biosynthetic process; purine ribonucleoside triphosphate biosynthetic process; ion homeostasis; calcium ion homeostasis; small molecule metabolic process; negative regulation of striated muscle contraction; inorganic ion	membrane; endoplasmic reticulum; nuclear outer membrane-endoplasmic reticulum membrane network; organelle membrane; myofibril; cytoplasm; intrinsic component of membrane; intracellular part; sarcoplasm; cell; I band; organelle part; non-membrane-bounded organelle; intracellular; cell part; membrane part; contractile fiber; membrane region; perinuclear region of cytoplasm; membrane-bounded organelle; endoplasmic reticulum membrane; intracellular organelle; bounding membrane of organelle; intracellular non-membrane-bounded organelle; H zone; intracellular organelle part; sarcoplasmic reticulum; endomembrane system; integral component of membrane; organelle; intracellular membrane-bounded organelle; endoplasmic reticulum part; endoplasmic reticulum-Golgi intermediate compartment; A band; sarcomere; contractile fiber part; cytoplasmic part;	ATPase activity, coupled to transmembrane movement of ions; nucleoside phosphate binding; primary active transmembrane transporter activity; substrate-specific transporter activity; hydrolase activity, acting on acid anhydrides; hydrolase activity; substrate-specific transmembrane transporter activity; ATPase activity; organic cyclic compound binding; heterocyclic compound binding; metal ion transmembrane transporter activity; pyrophosphatase activity; cation-transporting ATPase activity; catalytic activity; ribonucleotide binding; ATPase activity, coupled to movement of substances; binding; purine ribonucleoside binding; cation binding; hydrolase activity, acting on acid anhydrides, catalyzing transmembrane movement of substances; anion binding; adenyl ribonucleotide binding; adenyl nucleotide binding; inorganic cation transmembrane transporter activity; hydrolase activity, acting on acid anhydrides, in phosphorus-containing anhydrides; transporter activity; purine nucleotide binding; purine ribonucleoside triphosphate binding; active transmembrane transporter activity; ATP binding; ion transmembrane transporter activity; ion binding; nucleoside binding; nucleotide binding; nucleoside-triphosphatase activity; ribonucleoside binding; P-P-bond-hydrolysis-driven transmembrane transporter activity; ATPase activity, coupled to transmembrane movement of ions, phosphorylative mechanism; divalent inorganic cation transmembrane transporter activity; carbohydrate derivative binding; calcium-transporting ATPase activity; ATPase activity, coupled to transmembrane movement of substances; ATPase activity, coupled; calcium ion transmembrane transporter activity; metal ion binding; purine ribonucleotide binding; purine nucleoside binding; calcium ion binding; cation transmembrane transporter activity; transmembrane transporter activity; small molecule binding;	K05853	ATP2A; Ca2+ transporting ATPase, sarcoplasmic/endoplasmic reticulum [EC:3.6.3.8]	dre04020 Calcium signaling pathway - Danio rerio (zebrafish);	IPR004014; IPR023298; IPR008250; IPR023299; IPR006068; IPR023214;	Cation-transporting P-type ATPase, N-terminal; P-type ATPase, transmembrane domain; P-type ATPase, A domain; P-type ATPase, cytoplasmic domain N; Cation-transporting P-type ATPase, C-terminal; HAD-like domain;
F1QIR4	-	Uncharacterized protein	_AQLEFNQVK(cr)ADIER_	1572	1	QLEFNQVKADIERKL	..E....K.......	cytosol	cellular component biogenesis; muscle cell development; single-multicellular organism process; cellular component organization; muscle system process; cellular developmental process; cellular component morphogenesis; single-organism process; actin filament organization; cell development; anatomical structure morphogenesis; cell differentiation; actin cytoskeleton organization; muscle contraction; cellular component assembly; organelle assembly; macromolecular complex assembly; myofibril assembly; musculoskeletal movement; slow-twitch skeletal muscle fiber contraction; muscle structure development; cytoskeleton organization; anatomical structure formation involved in morphogenesis; cellular process; striated muscle myosin thick filament assembly; protein complex assembly; cellular protein complex assembly; skeletal muscle thin filament assembly; developmental process; cellular macromolecular complex assembly; striated muscle cell differentiation; actomyosin structure organization; system process; protein complex subunit organization; multicellular organismal movement; single-organism cellular process; cellular component assembly involved in morphogenesis; striated muscle contraction; myosin filament	intracellular organelle; intracellular non-membrane-bounded organelle; cytoskeletal part; intracellular organelle part; macromolecular complex; intracellular part; cell; actin cytoskeleton; organelle part; non-membrane-bounded organelle; organelle; cytoskeleton; cell part; intracellular; myosin complex; protein complex;	ATP binding; motor activity; nucleoside phosphate binding; nucleoside binding; ion binding; hydrolase activity, acting on acid anhydrides; hydrolase activity; nucleotide binding; nucleoside-triphosphatase activity; organic cyclic compound binding; heterocyclic compound binding; ribonucleoside binding; carbohydrate derivative binding; pyrophosphatase activity; catalytic activity; ribonucleotide binding; binding; purine ribonucleoside binding; anion binding; purine ribonucleotide binding; adenyl ribonucleotide binding; adenyl nucleotide binding; purine nucleoside binding; hydrolase activity, acting on acid anhydrides, in phosphorus-containing anhydrides; purine nucleotide binding; small molecule binding; purine ribonucleoside triphosphate binding;	K10352	MYH; myosin heavy chain	dre04530 Tight junction - Danio rerio (zebrafish);	IPR002928; IPR004009; IPR027417; IPR001609; IPR027401;	Myosin tail; Myosin, N-terminal, SH3-like; P-loop containing nucleoside triphosphate hydrolase; Myosin head, motor domain; Myosin-like IQ motif-containing domain;

E9QBV1	cltcb	Clathrin heavy chain	_AQLQK(cr)GLVK_	1118	1	LAKAQLQKGLVKEAI	.......K.L.....	cytosol	establishment of protein localization; transport; intracellular transport; cellular macromolecule localization; establishment of localization in cell; vesicle-mediated transport; intracellular protein transport; organic substance transport; protein localization; macromolecule localization; cellular protein localization; localization; protein transport; establishment of localization; cellular localization;	transport vesicle membrane; membrane; clathrin coat of trans-Golgi network vesicle; cytoplasmic vesicle; organelle membrane; membrane-bounded vesicle; cell periphery; cytoplasmic membrane-bounded vesicle; vesicle; Golgi-associated vesicle membrane; cytoplasm; cytoplasmic vesicle part; macromolecular complex; intracellular part; vesicle coat; cell; organelle part; coated vesicle membrane; trans-Golgi network transport vesicle; clathrin-coated vesicle; membrane part; cell part; intracellular; Golgi apparatus part; trans-Golgi network transport vesicle membrane; clathrin coat of coated pit; membrane-bounded organelle; intracellular organelle; bounding membrane of organelle; coated membrane; intracellular organelle part; Golgi membrane; vesicle membrane; endomembrane system; coated vesicle; clathrin-coated vesicle membrane; Golgi-associated vesicle; plasma membrane; cytoplasmic vesicle membrane; intracellular membrane-bounded organelle; organelle; clathrin vesicle coat; plasma membrane part; clathrin coat; membrane coat; Golgi apparatus; transport vesicle; coated pit; protein complex; cytoplasmic part;	structural molecule activity;	K04646	CLTC; clathrin heavy chain	dre04144 Endocytosis - Danio rerio (zebrafish); dre04142 Lysosome - Danio rerio (zebrafish);	IPR016025; IPR015348; IPR011990; IPR016024; IPR001473;	Clathrin, heavy chain, linker/propeller domain; Clathrin, heavy chain, linker, core motif; Tetratricopeptide-like helical domain; Armadillo-type fold; Clathrin, heavy chain, propeller, N-terminal;
Q6P3J5	eef2b	Eukaryotic translation elongation factor 2	_ARPFPDGLAEDIDK(cr)GDVSSR_	619	1	GLAEDIDKGDVSSRQ	......DK.......	cytosol			nucleoside phosphate binding; translation elongation factor activity; hydrolase activity, acting on acid anhydrides; hydrolase activity; organic cyclic compound binding; heterocyclic compound binding; pyrophosphatase activity; catalytic activity; ribonucleotide binding; binding; GTPase activity; purine ribonucleoside binding; anion binding; hydrolase activity, acting on acid anhydrides, in phosphorus-containing anhydrides; guanyl nucleotide binding; GTP binding; purine nucleotide binding; purine ribonucleoside triphosphate binding; RNA binding; ion binding; nucleoside binding; nucleotide binding; nucleoside-triphosphatase activity; ribonucleoside binding; translation factor activity, nucleic acid binding; carbohydrate derivative binding; nucleic acid binding; purine ribonucleotide binding; guanyl ribonucleotide binding; purine nucleoside binding; small molecule binding;	K03234			IPR027417; IPR000795; IPR005517; IPR005225; IPR004161; IPR000640; IPR020568; IPR014721; IPR009022; IPR009000;	P-loop containing nucleoside triphosphate hydrolase; Elongation factor, GTP-binding domain; Translation elongation factor EFG/EF2, domain IV; Small GTP-binding protein domain; Translation elongation factor EFTu/EF1A, domain 2; Translation elongation factor EFG, V domain; Ribosomal protein S5 domain 2-type fold; Ribosomal protein S5 domain 2-type fold, subgroup; Elongation factor G, III-V domain; Translation protein, beta-barrel domain;
A2BGU3	nono	Uncharacterized protein	_ASEIFINK(cr)DR_	105	1	ASEIFINKDRGFGFI	..E....K.......	cytosol			organic cyclic compound binding; heterocyclic compound binding; nucleoside phosphate binding; binding; nucleic acid binding; nucleotide binding; small molecule binding;				IPR012975; IPR000504; IPR012677;	NOPS; RNA recognition motif domain; Nucleotide-binding, alpha-beta plait;
F1QK60	krt4	Uncharacterized protein	_ASVENEFVLLK(cr)K_	220	1	ENEFVLLKKDVDAAY	..E....K.......	nuclear		intracellular organelle; intracellular non-membrane-bounded organelle; cytoskeletal part; intracellular organelle part; macromolecular complex; intracellular part; cell; keratin filament; organelle part; non-membrane-bounded organelle; organelle; cytoskeleton; intermediate filament cytoskeleton; cell part; intracellular; intermediate filament; protein complex;	structural molecule activity;	K07605				
P13104	tpma	Tropomyosin alpha-1 chain	_ATEDELDK(cr)YSEALK_	59	1	ATEDELDKYSEALKD	......DK.......	cytosol		cytoskeleton; organelle; intracellular organelle; intracellular non-membrane-bounded organelle; cell part; intracellular; cytoplasm; intracellular part; cell; non-membrane-bounded organelle;		K10373	TPM1; tropomyosin 1	dre04260 Cardiac muscle contraction - Danio rerio (zebrafish); dre04261 Adrenergic signaling in cardiomyocytes - Danio rerio (zebrafish);		
P13104	tpma	Tropomyosin alpha-1 chain	_ATEDELDKYSEALK(cr)DAQEK_	65	1	DKYSEALKDAQEKLE		cytosol		cytoskeleton; organelle; intracellular organelle; intracellular non-membrane-bounded organelle; cell part; intracellular; cytoplasm; intracellular part; cell; non-membrane-bounded organelle;		K10373	TPM1; tropomyosin 1	dre04260 Cardiac muscle contraction - Danio rerio (zebrafish); dre04261 Adrenergic signaling in cardiomyocytes - Danio rerio (zebrafish);		
O93548	bE1	Embryonic 1 beta-globin	_ATIQDIFAK(cr)ADYDVIGPQALAR_	18	1	TIQDIFAKADYDVIG		cytosol		cell part; intracellular; hemoglobin complex; cytoplasm; macromolecular complex; intracellular part; cytosolic part; protein complex; cell; cytosol; cytoplasmic part;	organic cyclic compound binding; heterocyclic compound binding; heme binding; iron ion binding; transition metal ion binding; oxygen binding; binding; ion binding; substrate-specific transporter activity; metal ion binding; cation binding; oxygen transporter activity; tetrapyrrole binding; transporter activity;				IPR009050; IPR000971; IPR012292;	Globin-like; Globin; Globin, structural domain;
E9QDY3	cyt1	Uncharacterized protein	_ATTETK(cr)TT_	448	1	SKATTETKTT_____		mitochondria		intracellular organelle; intracellular non-membrane-bounded organelle; cytoskeletal part; intracellular organelle part; macromolecular complex; intracellular part; cell; organelle part; non-membrane-bounded organelle; organelle; cytoskeleton; intermediate filament cytoskeleton; cell part; intracellular; intermediate filament; protein complex;	structural molecule activity;	K07604				

B7ZD32	rps24	40S ribosomal protein S24	_ATVPK(cr)TEIR_	25	1	PGKATVPKTEIREKL	.......K.E.....	cytosol	cellular protein metabolic process; biosynthetic process; primary metabolic process; cellular metabolic process; organic substance biosynthetic process; cellular biosynthetic process; organic substance metabolic process; metabolic process; cellular macromolecule biosynthetic process; cellular macromolecule metabolic process; gene expression; macromolecule metabolic process; protein metabolic process; macromolecule biosynthetic process; translation; cellular process;	ribosome; organelle; intracellular organelle; intracellular non-membrane-bounded organelle; cell part; intracellular; cytoplasm; macromolecular complex; intracellular part; cell; ribonucleoprotein complex; cytoplasmic part; non-membrane-bounded organelle;	organic cyclic compound binding; heterocyclic compound binding; nucleoside phosphate binding; binding; nucleotide binding; structural molecule activity; structural constituent of ribosome; small molecule binding;				IPR012678; IPR012677;	Ribosomal protein L23/L15e core domain; Nucleotide-binding, alpha-beta plait;
Q6PBI5	rps25	40S ribosomal protein S25	_ATYDK(cr)LYK_	56	1	FDKATYDKLYKEVPN	......DK.......	nuclear		ribosome; organelle; intracellular organelle; intracellular non-membrane-bounded organelle; cell part; intracellular; cytoplasm; macromolecular complex; intracellular part; cell; ribonucleoprotein complex; cytoplasmic part; non-membrane-bounded organelle;		K02975	RP-S25e; small subunit ribosomal protein S25e	dre03010 Ribosome - Danio rerio (zebrafish);		
Q6TH14	-	Enolase	_AVDHVNK(cr)DIAPK_	71	1	KAVDHVNKDIAPKLI	.......K.I.....	mitochondria	single-organism carbohydrate catabolic process; glycolytic process; single-organism catabolic process; small molecule metabolic process; single-organism cellular process; single-organism process; carbohydrate metabolic process; primary metabolic process; cellular metabolic process; organic substance metabolic process; carbohydrate catabolic process; single-organism carbohydrate metabolic process; organic substance catabolic process; metabolic process; pyruvate metabolic process; generation of precursor metabolites and energy; single-organism metabolic process; carboxylic acid metabolic process; monocarboxylic acid metabolic process; organic acid metabolic process; cellular process; catabolic process; oxoacid metabolic process;	catalytic complex; cell part; intracellular; cytoplasm; macromolecular complex; intracellular part; cytosolic part; cell; cytosol; protein complex; phosphopyruvate hydratase complex; cytoplasmic part;	magnesium ion binding; phosphopyruvate hydratase activity; catalytic activity; binding; ion binding; metal ion binding; cation binding; lyase activity; hydro-lyase activity; carbon-oxygen lyase activity;	K01689	ENO; enolase [EC:4.2.1.11]	dre00010 Glycolysis / Gluconeogenesis - Danio rerio (zebrafish); dre01230 Biosynthesis of amino acids - Danio rerio (zebrafish); dre01200 Carbon metabolism - Danio rerio (zebrafish); dre01100 Metabolic pathways - Danio rerio (zebrafish); dre03018 RNA degradation - Danio rerio (zebrafish);	IPR029065; IPR020810; IPR029017; IPR020811;	Enolase C-terminal domain-like; Enolase, C-terminal; Enolase N-terminal domain-like; Enolase, N-terminal;
Q6P3L3	hspa5	Heat shock protein 5	_AVEEK(cr)IEWLEAHQDADLEEFQAK_	599	1	IEKAVEEKIEWLEAH	.......K.E.....	extracellular	response to stress; response to stimulus;		ATP binding; nucleoside phosphate binding; nucleoside binding; ion binding; nucleotide binding; organic cyclic compound binding; heterocyclic compound binding; ribonucleoside binding; carbohydrate derivative binding; ribonucleotide binding; binding; purine ribonucleoside binding; anion binding; purine ribonucleotide binding; adenyl ribonucleotide binding; adenyl nucleotide binding; purine nucleoside binding; purine nucleotide binding; small molecule binding; purine ribonucleoside triphosphate binding;	K09490	HSPA5; heat shock 70kDa protein 5	dre03060 Protein export - Danio rerio (zebrafish); dre04141 Protein processing in endoplasmic reticulum - Danio rerio (zebrafish);	IPR029048; IPR029047;	Heat shock protein 70kD, C-terminal domain; Heat shock protein 70kD, peptide-binding domain;
B8A561	myhz1.2	Uncharacterized protein	_AVETEK(cr)AEIQTALEEAEGTLEHEESK_	1539	1	KKAVETEKAEIQTAL	.......K.E.....	nuclear		intracellular organelle; intracellular non-membrane-bounded organelle; cytoskeletal part; intracellular organelle part; macromolecular complex; intracellular part; cell; actin cytoskeleton; organelle part; non-membrane-bounded organelle; organelle; cytoskeleton; cell part; intracellular; myosin complex; protein complex;	ATP binding; motor activity; nucleoside phosphate binding; nucleoside binding; ion binding; hydrolase activity, acting on acid anhydrides; hydrolase activity; nucleotide binding; nucleoside-triphosphatase activity; organic cyclic compound binding; heterocyclic compound binding; ribonucleoside binding; carbohydrate derivative binding; pyrophosphatase activity; catalytic activity; ribonucleotide binding; binding; purine ribonucleoside binding; anion binding; purine ribonucleotide binding; adenyl ribonucleotide binding; adenyl nucleotide binding; purine nucleoside binding; hydrolase activity, acting on acid anhydrides, in phosphorus-containing anhydrides; purine nucleotide binding; small molecule binding; purine ribonucleoside triphosphate binding;	K10352	MYH; myosin heavy chain	dre04530 Tight junction - Danio rerio (zebrafish);	IPR002928; IPR004009; IPR001609; IPR027417; IPR027401;	Myosin tail; Myosin, N-terminal, SH3-like; Myosin head, motor domain; P-loop containing nucleoside triphosphate hydrolase; Myosin-like IQ motif-containing domain;
F1REG7	oxct1a	Uncharacterized protein (Fragment)	_AVFDVDK(cr)DK_	75	1	KAVFDVDKDKGLTLI	......DK.......	cytosol			CoA-transferase activity; transferase activity, transferring sulfur-containing groups; catalytic activity; transferase activity;					
Q6NV37	gyg1a	Glycogenin 1	_AVLHK(cr)IYDEVR_	53	1	PSRAVLHKIYDEVRL		cytosol			transferase activity, transferring glycosyl groups; catalytic activity; transferase activity;	K00750			IPR029044;	Nucleotide-diphospho-sugar transferases;
Q642Z0	atp2a1	Calcium-transporting ATPase	_AVNQDK(cr)K_	204	1	PRAVNQDKKNMLFSG	......DK.......	plasma membrane	nucleoside metabolic process; purine nucleoside metabolic process; purine ribonucleoside monophosphate metabolic process; nucleotide metabolic process; single-organism process; purine ribonucleoside biosynthetic process; nucleoside biosynthetic process; biosynthetic process; ribonucleoside monophosphate biosynthetic process; cellular metabolic process; phosphate-containing compound metabolic process; ribose phosphate biosynthetic process; metabolic process; carbohydrate derivative biosynthetic process; nucleoside phosphate metabolic process; ribose phosphate metabolic process; purine nucleoside triphosphate biosynthetic process; heterocycle biosynthetic process; purine ribonucleoside triphosphate biosynthetic process; small molecule metabolic process; ATP biosynthetic process; single-organism cellular process; purine ribonucleoside triphosphate metabolic process; organic cyclic compound metabolic process; purine-containing compound metabolic process; ribonucleoside triphosphate metabolic process; purine ribonucleoside monophosphate biosynthetic process; purine nucleoside biosynthetic process; organophosphate biosynthetic process; ribonucleoside monophosphate	intrinsic component of membrane; membrane; integral component of membrane; membrane part;	ATPase activity, coupled to transmembrane movement of ions; nucleoside phosphate binding; primary active transmembrane transporter activity; substrate-specific transporter activity; hydrolase activity, acting on acid anhydrides; hydrolase activity; substrate-specific transmembrane transporter activity; ATPase activity; organic cyclic compound binding; heterocyclic compound binding; metal ion transmembrane transporter activity; pyrophosphatase activity; cation-transporting ATPase activity; catalytic activity; ribonucleotide binding; ATPase activity, coupled to movement of substances; binding; purine ribonucleoside binding; cation binding; hydrolase activity, acting on acid anhydrides, catalyzing transmembrane movement of substances; anion binding; adenyl ribonucleotide binding; adenyl nucleotide binding; inorganic cation transmembrane transporter activity; hydrolase activity, acting on acid anhydrides, in phosphorus-containing anhydrides; transporter activity; purine nucleotide binding; purine ribonucleoside triphosphate binding; active transmembrane transporter activity; ATP binding; ion transmembrane transporter activity; ion binding; nucleoside binding; nucleotide binding; nucleoside-triphosphatase activity; ribonucleoside binding; P-P-bond-hydrolysis-driven transmembrane transporter activity; ATPase activity, coupled to transmembrane movement of ions, phosphorylative mechanism; divalent inorganic cation transmembrane transporter activity; carbohydrate derivative binding; calcium-transporting ATPase activity; ATPase activity, coupled to transmembrane movement of substances; ATPase activity, coupled; metal ion binding; calcium ion transmembrane transporter activity; purine ribonucleotide binding; purine nucleoside binding; cation transmembrane transporter activity; transmembrane transporter activity; small molecule binding;	K05853	ATP2A; Ca2+ transporting ATPase, sarcoplasmic/endoplasmic reticulum [EC:3.6.3.8]	dre04020 Calcium signaling pathway - Danio rerio (zebrafish);	IPR023298; IPR004014; IPR023299; IPR008250; IPR006068; IPR023214;	P-type ATPase, transmembrane domain; Cation-transporting P-type ATPase, N-terminal; P-type ATPase, cytoplasmic domain N; P-type ATPase, A domain; Cation-transporting P-type ATPase, C-terminal; HAD-like domain;

Q5U3A4	atp2a1	Calcium-transporting ATPase	_AVNQDK(cr)K_	204	1	PRAVNQDKKNMLFSG	......DK.......	plasma membrane	regulation of biological quality; nucleoside metabolic process; purine nucleoside metabolic process; cellular divalent inorganic cation homeostasis; purine ribonucleoside monophosphate metabolic process; nucleotide metabolic process; positive regulation of muscle contraction; negative regulation of multicellular organismal process; single-organism process; purine ribonucleoside biosynthetic process; nucleoside biosynthetic process; biosynthetic process; ribonucleoside monophosphate biosynthetic process; cellular metabolic process; phosphate-containing compound metabolic process; response to external stimulus; ribose phosphate biosynthetic process; metabolic process; carbohydrate derivative biosynthetic process; positive regulation of multicellular organismal process; nucleoside phosphate metabolic process; regulation of skeletal muscle contraction; metal ion homeostasis; regulation of system process; ribose phosphate metabolic process; purine nucleoside triphosphate biosynthetic process; heterocycle biosynthetic process; purine ribonucleoside triphosphate biosynthetic process; ion homeostasis; calcium ion homeostasis; small molecule metabolic process; negative regulation of striated muscle contraction; inorganic ion	membrane; endoplasmic reticulum; nuclear outer membrane-endoplasmic reticulum membrane network; organelle membrane; myofibril; cytoplasm; intrinsic component of membrane; intracellular part; sarcoplasm; cell; I band; organelle part; non-membrane-bounded organelle; intracellular; cell part; membrane part; contractile fiber; membrane region; perinuclear region of cytoplasm; membrane-bounded organelle; endoplasmic reticulum membrane; intracellular organelle; bounding membrane of organelle; intracellular non-membrane-bounded organelle; H zone; intracellular organelle part; sarcoplasmic reticulum; endomembrane system; integral component of membrane; organelle; intracellular membrane-bounded organelle; endoplasmic reticulum part; endoplasmic reticulum-Golgi intermediate compartment; A band; sarcomere; contractile fiber part; cytoplasmic part;	ATPase activity, coupled to transmembrane movement of ions; nucleoside phosphate binding; primary active transmembrane transporter activity; substrate-specific transporter activity; hydrolase activity, acting on acid anhydrides; hydrolase activity; substrate-specific transmembrane transporter activity; ATPase activity; organic cyclic compound binding; heterocyclic compound binding; metal ion transmembrane transporter activity; pyrophosphatase activity; cation-transporting ATPase activity; catalytic activity; ribonucleotide binding; ATPase activity, coupled to movement of substances; binding; purine ribonucleoside binding; cation binding; hydrolase activity, acting on acid anhydrides, catalyzing transmembrane movement of substances; anion binding; adenyl ribonucleotide binding; adenyl nucleotide binding; inorganic cation transmembrane transporter activity; hydrolase activity, acting on acid anhydrides, in phosphorus-containing anhydrides; transporter activity; purine nucleotide binding; purine ribonucleoside triphosphate binding; active transmembrane transporter activity; ATP binding; ion transmembrane transporter activity; ion binding; nucleoside binding; nucleotide binding; nucleoside-triphosphatase activity; ribonucleoside binding; P-P-bond-hydrolysis-driven transmembrane transporter activity; ATPase activity, coupled to transmembrane movement of ions, phosphorylative mechanism; divalent inorganic cation transmembrane transporter activity; carbohydrate derivative binding; calcium-transporting ATPase activity; ATPase activity, coupled to transmembrane movement of substances; ATPase activity, coupled; calcium ion transmembrane transporter activity; metal ion binding; purine ribonucleotide binding; purine nucleoside binding; calcium ion binding; cation transmembrane transporter activity; transmembrane transporter activity; small molecule binding;	K05853	ATP2A; Ca2+ transporting ATPase, sarcoplasmic/endoplasmic reticulum [EC:3.6.3.8]	dre04020 Calcium signaling pathway - Danio rerio (zebrafish);	IPR004014; IPR023298; IPR008250; IPR023299; IPR006068; IPR023214;	Cation-transporting P-type ATPase, N-terminal; P-type ATPase, transmembrane domain; P-type ATPase, A domain; P-type ATPase, cytoplasmic domain N; Cation-transporting P-type ATPase, C-terminal; HAD-like domain;
Q6PC77	atp5h	ATP synthase, H+ transporting, mitochondrial F0 complex, subunit d	_AVVAK(cr)AGMVDEFEK_	63	1	HYRAVVAKAGMVDEF		cytosol	nucleoside metabolic process; purine nucleoside metabolic process; purine ribonucleoside monophosphate metabolic process; nucleotide metabolic process; single-organism process; purine ribonucleoside biosynthetic process; nucleoside biosynthetic process; biosynthetic process; ribonucleoside monophosphate biosynthetic process; cellular metabolic process; phosphate-containing compound metabolic process; establishment of localization; ribose phosphate biosynthetic process; metabolic process; carbohydrate derivative biosynthetic process; nucleoside phosphate metabolic process; ribose phosphate metabolic process; purine nucleoside triphosphate biosynthetic process; single-organism transport; heterocycle biosynthetic process; cation transmembrane transport; purine ribonucleoside triphosphate biosynthetic process; small molecule metabolic process; ATP biosynthetic process; single-organism cellular process; purine ribonucleoside triphosphate metabolic process; organic cyclic compound metabolic process; purine-containing compound metabolic process; energy coupled proton transport, down electrochemical gradient; ribonucleoside triphosphate metabolic process; purine	intracellular organelle; membrane; mitochondrial part; organelle membrane; organelle envelope; intracellular organelle part; cytoplasm; macromolecular complex; intracellular part; mitochondrial proton-transporting ATP synthase complex, coupling factor F(o); cell; mitochondrial membrane part; proton-transporting ATP synthase complex; mitochondrion; organelle part; intracellular membrane-bounded organelle; organelle; mitochondrial envelope; mitochondrial proton-transporting ATP synthase complex; membrane part; cell part; intracellular; proton-transporting two-sector ATPase complex; envelope; organelle inner membrane; proton-transporting ATP synthase complex, coupling factor F(o); protein complex; membrane-bounded organelle; mitochondrial inner membrane; proton-transporting two-sector ATPase complex, proton-transporting domain; mitochondrial membrane; cytoplasmic part;	ion transmembrane transporter activity; substrate-specific transporter activity; monovalent inorganic cation transmembrane transporter activity; inorganic cation transmembrane transporter activity; hydrogen ion transmembrane transporter activity; cation transmembrane transporter activity; substrate-specific transmembrane transporter activity; transporter activity; transmembrane transporter activity;	K02138	ATPeF0D; F-type H+-transporting ATPase subunit d	dre01100 Metabolic pathways - Danio rerio (zebrafish); dre00190 Oxidative phosphorylation - Danio rerio (zebrafish);		
G1K2X0	ttnb	Uncharacterized protein (Fragment)	_AYHVEK(cr)R_	22276	1	VKAYHVEKREASKKA	.......K.E.....				ATP binding; nucleoside phosphate binding; phosphotransferase activity, alcohol group as acceptor; nucleoside binding; ion binding; protein tyrosine kinase activity; protein kinase activity; nucleotide binding; organic cyclic compound binding; heterocyclic compound binding; ribonucleoside binding; carbohydrate derivative binding; kinase activity; catalytic activity; ribonucleotide binding; binding; nucleic acid binding; purine ribonucleoside binding; anion binding; purine ribonucleotide binding; adenyl ribonucleotide binding; adenyl nucleotide binding; purine nucleoside binding; transferase activity; transferase activity, transferring phosphorus-containing groups; purine nucleotide binding; small molecule binding; purine ribonucleoside triphosphate binding;	K12567			IPR003598; IPR007110; IPR000719; IPR013783; IPR003599; IPR003961; IPR013098; IPR011009;	Immunoglobulin subtype 2; Immunoglobulin-like domain; Protein kinase domain; Immunoglobulin-like fold; Immunoglobulin subtype; Fibronectin, type III; Immunoglobulin I-set; Protein kinase-like domain;
Q6P5L2	dbi	Uncharacterized protein	_AYIAK(cr)VEELK_	77	1	AVKAYIAKVEELKGK	.......K.E.....	cytosol			fatty-acyl-CoA binding; binding; ion binding; anion binding; cofactor binding; sulfur compound binding; coenzyme binding;	K08762	DBI; diazepam-binding inhibitor (GABA receptor modulator, acyl-CoA-binding protein)	dre03320 PPAR signaling pathway - Danio rerio (zebrafish);	IPR000582; IPR014352;	Acyl-CoA-binding protein, ACBP; FERM/acyl-CoA-binding protein, 3-helical bundle;
E9QJ96	ywhaba	14-3-3 protein beta/alpha-A	_AYQDAFEISK(cr)K_	157	1	QDAFEISKKEMQPTH	.......K.E.....	cytosol							IPR023410;	14-3-3 domain;
B8A568	myhz1.1	Uncharacterized protein	_CASLEK(cr)TK_	1418	1	SKCASLEKTKQRLQG		nuclear		intracellular organelle; intracellular non-membrane-bounded organelle; cytoskeletal part; intracellular organelle part; macromolecular complex; intracellular part; cell; actin cytoskeleton; organelle part; non-membrane-bounded organelle; organelle; cytoskeleton; cell part; intracellular; myosin complex; protein complex;	ATP binding; motor activity; nucleoside phosphate binding; nucleoside binding; ion binding; hydrolase activity, acting on acid anhydrides; hydrolase activity; nucleotide binding; nucleoside-triphosphatase activity; organic cyclic compound binding; heterocyclic compound binding; ribonucleoside binding; carbohydrate derivative binding; pyrophosphatase activity; catalytic activity; ribonucleotide binding; binding; purine ribonucleoside binding; anion binding; purine ribonucleotide binding; adenyl ribonucleotide binding; adenyl nucleotide binding; purine nucleoside binding; hydrolase activity, acting on acid anhydrides, in phosphorus-containing anhydrides; purine nucleotide binding; small molecule binding; purine ribonucleoside triphosphate binding;	K10352	MYH; myosin heavy chain	dre04530 Tight junction - Danio rerio (zebrafish);	IPR002928; IPR004009; IPR001609; IPR027417; IPR027401;	Myosin tail; Myosin, N-terminal, SH3-like; Myosin head, motor domain; P-loop containing nucleoside triphosphate hydrolase; Myosin-like IQ motif-containing domain;

B8A561	myhz1.2	Uncharacterized protein	_CASLEK(cr)TK_	1418	1	SKCASLEKTKQRLQG		nuclear		intracellular organelle; intracellular non-membrane-bounded organelle; cytoskeletal part; intracellular organelle part; macromolecular complex; intracellular part; cell; actin cytoskeleton; organelle part; non-membrane-bounded organelle; organelle; cytoskeleton; cell part; intracellular; myosin complex; protein complex;	ATP binding; motor activity; nucleoside phosphate binding; nucleoside binding; ion binding; hydrolase activity, acting on acid anhydrides; hydrolase activity; nucleotide binding; nucleoside-triphosphatase activity; organic cyclic compound binding; heterocyclic compound binding; ribonucleoside binding; carbohydrate derivative binding; pyrophosphatase activity; catalytic activity; ribonucleotide binding; binding; purine ribonucleoside binding; anion binding; purine ribonucleotide binding; adenyl ribonucleotide binding; adenyl nucleotide binding; purine nucleoside binding; hydrolase activity, acting on acid anhydrides, in phosphorus-containing anhydrides; purine nucleotide binding; small molecule binding; purine ribonucleoside triphosphate binding;	K10352	MYH; myosin heavy chain	dre04530 Tight junction - Danio rerio (zebrafish);	IPR002928; IPR004009; IPR001609; IPR027417; IPR027401;	Myosin tail; Myosin, N-terminal, SH3-like; Myosin head, motor domain; P-loop containing nucleoside triphosphate hydrolase; Myosin-like IQ motif-containing domain;
Q68EH2	ak1	Adenylate kinase isoenzyme 1	_DAMIAK(cr)ADVSK_	83	1	IKDAMIAKADVSKGY		cytosol	nucleoside metabolic process; purine nucleoside metabolic process; cellular aromatic compound metabolic process; purine ribonucleoside monophosphate metabolic process; ribonucleotide metabolic process; nucleotide metabolic process; single-organism process; cellular metabolic process; phosphate-containing compound metabolic process; organic substance metabolic process; metabolic process; heterocycle metabolic process; nucleobase-containing compound metabolic process; nucleoside phosphate metabolic process; carbohydrate derivative metabolic process; ribose phosphate metabolic process; cellular nitrogen compound metabolic process; nucleobase-containing small molecule metabolic process; purine nucleoside triphosphate metabolic process; cellular process; ribonucleoside metabolic process; ATP metabolic process; small molecule metabolic process; single-organism cellular process; purine ribonucleoside triphosphate metabolic process; nitrogen compound metabolic process; organic cyclic compound metabolic process; purine-containing compound metabolic process; nucleoside triphosphate metabolic process; primary metabolic process; ribonucleoside triphosphate metabolic process; purine nucleotide metabolic [illegible]	intracellular part; cell; cell part; intracellular; cytoplasm;	ATP binding; nucleoside phosphate binding; adenylate kinase activity; nucleoside binding; ion binding; nucleobase-containing compound kinase activity; nucleotide binding; nucleotide kinase activity; phosphotransferase activity, phosphate group as acceptor; organic cyclic compound binding; heterocyclic compound binding; ribonucleoside binding; carbohydrate derivative binding; kinase activity; ribonucleotide binding; catalytic activity; binding; purine ribonucleoside binding; purine ribonucleotide binding; anion binding; adenyl ribonucleotide binding; adenyl nucleotide binding; purine nucleoside binding; transferase activity; purine nucleotide binding; small molecule binding; purine ribonucleoside triphosphate binding; transferase activity, transferring phosphorus-containing groups;	K00939	adk; adenylate kinase [EC:2.7.4.3]	dre01100 Metabolic pathways - Danio rerio (zebrafish); dre00230 Purine metabolism - Danio rerio (zebrafish);	IPR027417;	P-loop containing nucleoside triphosphate hydrolase;
P13104	tpma	Tropomyosin alpha-1 chain	_DAQEK(cr)LELAEK_	70	1	ALKDAQEKLELAEKK	.......K.E.....	cytosol		cytoskeleton; organelle; intracellular organelle; intracellular non-membrane-bounded organelle; cell part; intracellular; cytoplasm; intracellular part; cell; non-membrane-bounded organelle;		K10373	TPM1; tropomyosin 1	dre04260 Cardiac muscle contraction - Danio rerio (zebrafish); dre04261 Adrenergic signaling in cardiomyocytes - Danio rerio [illegible]		
E7F7C7	-	Histone H4	_DAVTYTEHAK(cr)R_	78	1	VTYTEHAKRKTVTAM		nuclear	cellular component biogenesis; cellular component organization; nucleosome organization; cellular macromolecular complex assembly; chromatin assembly or disassembly; protein complex subunit organization; macromolecular complex subunit organization; protein-DNA complex assembly; DNA packaging; nucleosome assembly; chromosome organization; cellular component assembly; macromolecular complex assembly; organelle organization; chromatin organization; protein complex biogenesis; chromatin assembly; cellular process; protein complex assembly; cellular component organization or biogenesis; protein-DNA complex subunit organization; DNA conformation change;	intracellular organelle; intracellular non-membrane-bounded organelle; intracellular organelle part; macromolecular complex; intracellular part; cell; organelle part; DNA bending complex; chromosomal part; nucleosome; non-membrane-bounded organelle; intracellular membrane-bounded organelle; organelle; nucleus; chromatin; cell part; intracellular; chromosome; membrane-bounded organelle; protein complex; protein-DNA complex; DNA packaging complex;	organic cyclic compound binding; heterocyclic compound binding; DNA binding; binding; nucleic acid binding;				IPR009072; IPR007125;	Histone-fold; Histone core;
Q5RHQ7	hnrnpub	Uncharacterized protein	_DEASK(cr)LLEK_	581	1	LQKDEASKLLEKYKE	.......K.L.....	nuclear		organelle; intracellular membrane-bounded organelle; intracellular organelle; nucleus; cell part; intracellular; macromolecular complex; intracellular part; ribonucleoprotein complex; membrane-bounded organelle; cell;	organic cyclic compound binding; heterocyclic compound binding; binding; nucleic acid binding;	K12888	HNRNPU; heterogeneous nuclear ribonucleoprotein U	dre03040 Spliceosome - Danio rerio (zebrafish);	IPR003034; IPR001870; IPR027417; IPR003877;	SAP domain; B30.2/SPRY domain; P-loop containing nucleoside triphosphate hydrolase; SPRY domain;
Q8JHI0	slc25a5	Solute carrier family 25 alpha, member 5	_DEGGK(cr)AFFK_	268	1	IARDEGGKAFFKGAW		cytosol	transport; transmembrane transport; single-organism localization; single-organism cellular process; single-organism process; localization; cellular process; single-organism transport; establishment of localization;	intracellular organelle; membrane; mitochondrial part; organelle membrane; organelle envelope; intracellular organelle part; cytoplasm; intrinsic component of membrane; intracellular part; cell; integral component of membrane; organelle part; mitochondrion; organelle; intracellular membrane-bounded organelle; mitochondrial envelope; intracellular; cell part; membrane part; envelope; organelle inner membrane; membrane-bounded organelle; mitochondrial inner membrane; mitochondrial membrane; cytoplasmic part;	transporter activity;	K05863	SLC25A4S; solute carrier family 25 (mitochondrial adenine nucleotide translocator), member 4/5/6/31	dre04020 Calcium signaling pathway - Danio rerio (zebrafish);	IPR023395;	Mitochondrial carrier domain;
Q6IQX1	myhz2	Myosin, heavy polypeptide 2, fast muscle specific	_DFETAQLLSK(cr)IEDEQSLGAQLQK_	1095	1	ETAQLLSKIEDEQSL	.......K.E.....	cytosol	response to stress; response to stimulus;	intracellular organelle; intracellular non-membrane-bounded organelle; cytoskeletal part; intracellular organelle part; macromolecular complex; intracellular part; cell; actin cytoskeleton; organelle part; non-membrane-bounded organelle; organelle; cytoskeleton; cell part; intracellular; myosin complex; protein complex;	ATP binding; motor activity; nucleoside phosphate binding; nucleoside binding; ion binding; hydrolase activity, acting on acid anhydrides; hydrolase activity; nucleotide binding; nucleoside-triphosphatase activity; organic cyclic compound binding; heterocyclic compound binding; ribonucleoside binding; carbohydrate derivative binding; pyrophosphatase activity; catalytic activity; ribonucleotide binding; binding; purine ribonucleoside binding; anion binding; purine ribonucleotide binding; adenyl ribonucleotide binding; adenyl nucleotide binding; purine nucleoside binding; hydrolase activity, acting on acid anhydrides, in phosphorus-containing anhydrides; purine nucleotide binding; small molecule binding; purine ribonucleoside triphosphate binding;	K10352	MYH; myosin heavy chain	dre04530 Tight junction - Danio rerio (zebrafish);	IPR002928; IPR004009; IPR001609; IPR027417; IPR027401;	Myosin tail; Myosin, N-terminal, SH3-like; Myosin head, motor domain; P-loop containing nucleoside triphosphate hydrolase; Myosin-like IQ motif-containing domain;

B8A568	myhz1.1	Uncharacterized protein	_DFETSQLLSK(cr)IEDEQSLGAQLQK_	1095	1	ETSQLLSKIEDEQSL	.......K.E.....	nuclear		intracellular organelle; intracellular non-membrane-bounded organelle; cytoskeletal part; intracellular organelle part; macromolecular complex; intracellular part; cell; actin cytoskeleton; organelle part; non-membrane-bounded organelle; organelle; cytoskeleton; cell part; intracellular; myosin complex; protein complex;	ATP binding; motor activity; nucleoside phosphate binding; nucleoside binding; ion binding; hydrolase activity, acting on acid anhydrides; hydrolase activity; nucleotide binding; nucleoside-triphosphatase activity; organic cyclic compound binding; heterocyclic compound binding; ribonucleoside binding; carbohydrate derivative binding; pyrophosphatase activity; catalytic activity; ribonucleotide binding; binding; purine ribonucleoside binding; anion binding; purine ribonucleotide binding; adenyl ribonucleotide binding; adenyl nucleotide binding; purine nucleoside binding; hydrolase activity, acting on acid anhydrides, in phosphorus-containing anhydrides; purine nucleotide binding; small molecule binding; purine ribonucleoside triphosphate binding;	K10352	MYH; myosin heavy chain	dre04530 Tight junction - Danio rerio (zebrafish);	IPR002928; IPR004009; IPR001609; IPR027417; IPR027401;	Myosin tail; Myosin, N-terminal, SH3-like; Myosin head, motor domain; P-loop containing nucleoside triphosphate hydrolase; Myosin-like IQ motif-containing domain;
B8A561	myhz1.2	Uncharacterized protein	_DFETSQLLSK(cr)IEDEQSLGAQLQK_	1095	1	ETSQLLSKIEDEQSL	.......K.E.....	nuclear		intracellular organelle; intracellular non-membrane-bounded organelle; cytoskeletal part; intracellular organelle part; macromolecular complex; intracellular part; cell; actin cytoskeleton; organelle part; non-membrane-bounded organelle; organelle; cytoskeleton; cell part; intracellular; myosin complex; protein complex;	ATP binding; motor activity; nucleoside phosphate binding; nucleoside binding; ion binding; hydrolase activity, acting on acid anhydrides; hydrolase activity; nucleotide binding; nucleoside-triphosphatase activity; organic cyclic compound binding; heterocyclic compound binding; ribonucleoside binding; carbohydrate derivative binding; pyrophosphatase activity; catalytic activity; ribonucleotide binding; binding; purine ribonucleoside binding; anion binding; purine ribonucleotide binding; adenyl ribonucleotide binding; adenyl nucleotide binding; purine nucleoside binding; hydrolase activity, acting on acid anhydrides, in phosphorus-containing anhydrides; purine nucleotide binding; small molecule binding; purine ribonucleoside triphosphate binding;	K10352	MYH; myosin heavy chain	dre04530 Tight junction - Danio rerio (zebrafish);	IPR002928; IPR004009; IPR001609; IPR027417; IPR027401;	Myosin tail; Myosin, N-terminal, SH3-like; Myosin head, motor domain; P-loop containing nucleoside triphosphate hydrolase; Myosin-like IQ motif-containing domain;
Q8JHI0	slc25a5	Solute carrier family 25 alpha, member 5	_DFLAGGIAAAISK(cr)TAVAPIER_	23	1	GIAAAISKTAVAPIE		cytosol	transport; transmembrane transport; single-organism localization; single-organism cellular process; single-organism process; localization; cellular process; single-organism transport; establishment of localization;	intracellular organelle; membrane; mitochondrial part; organelle membrane; organelle envelope; intracellular organelle part; cytoplasm; intrinsic component of membrane; intracellular part; cell; integral component of membrane; organelle part; mitochondrion; organelle; intracellular membrane-bounded organelle; mitochondrial envelope; intracellular; cell part; membrane part; envelope; organelle inner membrane; membrane-bounded organelle; mitochondrial inner membrane; mitochondrial membrane; cytoplasmic part;	transporter activity;	K05863	SLC25A4S; solute carrier family 25 (mitochondrial adenine nucleotide translocator), member 4/5/6/31	dre04020 Calcium signaling pathway - Danio rerio (zebrafish);	IPR023395;	Mitochondrial carrier domain;
B8JN01	-	Histone H2A	_DFLETVK(cr)ELR_	241	1	KDFLETVKELRKSQG	.......K.L.....	mitochondria	cellular component biogenesis; cellular component organization; nucleosome organization; cellular macromolecular complex assembly; chromatin assembly or disassembly; protein complex subunit organization; macromolecular complex subunit organization; protein-DNA complex assembly; DNA packaging; nucleosome assembly; chromosome organization; cellular component assembly; macromolecular complex assembly; organelle organization; chromatin organization; protein complex biogenesis; chromatin assembly; cellular process; protein complex assembly; cellular component organization or biogenesis; protein-DNA complex subunit organization; DNA conformation change;	intracellular organelle; intracellular non-membrane-bounded organelle; intracellular organelle part; macromolecular complex; intracellular part; cell; organelle part; DNA bending complex; chromosomal part; nucleosome; non-membrane-bounded organelle; intracellular membrane-bounded organelle; organelle; nucleus; chromatin; cell part; intracellular; chromosome; membrane-bounded organelle; protein complex; protein-DNA complex; DNA packaging complex;	organic cyclic compound binding; heterocyclic compound binding; DNA binding; binding; nucleic acid binding;				IPR002119; IPR009072; IPR007125;	Histone H2A; Histone-fold; Histone core;
B0S564	-	Protein disulfide-isomerase	_DFQDK(cr)MDQFK_	269	1	AAKDFQDKMDQFKKA	......DK.......	extracellular	regulation of cellular process; regulation of biological quality; small molecule metabolic process; cell redox homeostasis; single-organism cellular process; ether metabolic process; cellular homeostasis; single-organism process; organic substance metabolic process; homeostatic process; metabolic process; regulation of biological process; single-organism metabolic process; glycerol ether metabolic process; cellular process; biological regulation;	organelle; intracellular membrane-bounded organelle; intracellular organelle; endoplasmic reticulum; cell part; intracellular; cytoplasm; endomembrane system; macromolecular complex; intracellular part; membrane-bounded organelle; organelle; cell; protein complex; collagen trimer; cytoplasmic part;	protein disulfide oxidoreductase activity; disulfide oxidoreductase activity; oxidoreductase activity, acting on single donors with incorporation of molecular oxygen, incorporation of two atoms of oxygen; electron carrier activity; oxidoreductase activity, acting on single donors with incorporation of molecular oxygen; isomerase activity; catalytic activity; dioxygenase activity; oxidoreductase activity, acting on a sulfur group of donors; oxidoreductase activity;	K09580	PDIA1; protein disulfide-isomerase A1 [EC:5.3.4.1]	dre04141 Protein processing in endoplasmic reticulum - Danio rerio (zebrafish);	IPR005788; IPR013766; IPR012336;	Disulphide isomerase; Thioredoxin domain; Thioredoxin-like fold;
F1QJP3	myl10	Uncharacterized protein	_DGFIDK(cr)NDLR_	45	1	NRDGFIDKNDLRDTF	......DK.......	cytosol,nuclear			cation binding; metal ion binding; calcium ion binding; binding; ion binding;				IPR002048; IPR011992;	EF-hand domain; EF-hand domain pair;
O93409	mylpfa	Uncharacterized protein	_DGIISK(cr)DDLR_	48	1	NRDGIISKDDLRDVL		cytosol,nuclear			cation binding; metal ion binding; calcium ion binding; binding; ion binding;	K12758	MYLPF; fast skeletal myosin light chain 2	dre04510 Focal adhesion - Danio rerio (zebrafish); dre04530 Tight junction - Danio rerio (zebrafish); dre04810 Regulation of actin cytoskeleton - Danio rerio (zebrafish);	IPR011992; IPR002048;	EF-hand domain pair; EF-hand domain;
Q6TH14	-	Enolase	_DIAPK(cr)LIEK_	76	1	VNKDIAPKLIEKKFS	.......K.I.....	mitochondria	single-organism carbohydrate catabolic process; glycolytic process; single-organism catabolic process; small molecule metabolic process; single-organism cellular process; single-organism process; carbohydrate metabolic process; primary metabolic process; cellular metabolic process; organic substance metabolic process; carbohydrate catabolic process; single-organism carbohydrate metabolic process; organic substance catabolic process; metabolic process; pyruvate metabolic process; generation of precursor metabolites and energy; single-organism metabolic process; carboxylic acid metabolic process; monocarboxylic acid metabolic process; organic acid metabolic process; cellular process; catabolic process; oxoacid metabolic process;	catalytic complex; cell part; intracellular; cytoplasm; macromolecular complex; intracellular part; cytosolic part; cell; cytosol; protein complex; phosphopyruvate hydratase complex; cytoplasmic part;	magnesium ion binding; phosphopyruvate hydratase activity; catalytic activity; binding; ion binding; metal ion binding; cation binding; lyase activity; hydro-lyase activity; carbon-oxygen lyase activity;	K01689	ENO; enolase [EC:4.2.1.11]	dre00010 Glycolysis / Gluconeogenesis - Danio rerio (zebrafish); dre01230 Biosynthesis of amino acids - Danio rerio (zebrafish); dre01200 Carbon metabolism - Danio rerio (zebrafish); dre01100 Metabolic pathways - Danio rerio (zebrafish); dre03018 RNA degradation - Danio rerio (zebrafish);	IPR029065; IPR020810; IPR029017; IPR020811;	Enolase C-terminal domain-like; Enolase, C-terminal; Enolase N-terminal domain-like; Enolase, N-terminal;

Q7ZYX4	cct6a	Chaperonin containing TCP1, subunit 6A (Zeta 1)	_DK(cr)ALAVLEEVK_	129	1	GFEAAKDKALAVLEE	......DK.......	cytosol	protein folding; metabolic process; cellular protein metabolic process; cellular macromolecule metabolic process; macromolecule metabolic process; protein metabolic process; primary metabolic process; cellular process; cellular metabolic process; organic substance metabolic process;	intracellular part; cell; cell part; intracellular; cytoplasm;	ATP binding; nucleoside phosphate binding; nucleoside binding; ion binding; nucleotide binding; organic cyclic compound binding; heterocyclic compound binding; ribonucleoside binding; carbohydrate derivative binding; ribonucleotide binding; binding; purine ribonucleoside binding; anion binding; purine ribonucleotide binding; adenyl ribonucleotide binding; adenyl nucleotide binding; purine nucleoside binding; purine nucleotide binding; small molecule binding; purine ribonucleoside triphosphate binding;	K09498			IPR027410; IPR027409; IPR027413;	TCP-1-like chaperonin intermediate domain; GroEL-like apical domain; GroEL-like equatorial domain;
E9QD64	cct6a	Uncharacterized protein	_DK(cr)ALAVLEEVK_	84	1	IAKAAKDKALAVLEE	......DK.......	cytosol	protein folding; metabolic process; cellular protein metabolic process; cellular macromolecule metabolic process; macromolecule metabolic process; protein metabolic process; primary metabolic process; cellular process; cellular metabolic process; organic substance metabolic process;	intracellular part; cell; cell part; intracellular; cytoplasm;	ATP binding; nucleoside phosphate binding; nucleoside binding; ion binding; nucleotide binding; organic cyclic compound binding; heterocyclic compound binding; ribonucleoside binding; carbohydrate derivative binding; ribonucleotide binding; binding; purine ribonucleoside binding; anion binding; purine ribonucleotide binding; adenyl ribonucleotide binding; adenyl nucleotide binding; purine nucleoside binding; purine nucleotide binding; small molecule binding; purine ribonucleoside triphosphate binding;				IPR027410; IPR027409; IPR027413;	TCP-1-like chaperonin intermediate domain; GroEL-like apical domain; GroEL-like equatorial domain;
Q642Z0	atp2a1	Calcium-transporting ATPase	_DK(cr)IMAVIK_	544	1	LTGPVKDKIMAVIKE	......DK.......	plasma membrane	nucleoside metabolic process; purine nucleoside metabolic process; purine ribonucleoside monophosphate metabolic process; nucleotide metabolic process; single-organism process; purine ribonucleoside biosynthetic process; nucleoside biosynthetic process; biosynthetic process; ribonucleoside monophosphate biosynthetic process; cellular metabolic process; phosphate-containing compound metabolic process; ribose phosphate biosynthetic process; metabolic process; carbohydrate derivative biosynthetic process; nucleoside phosphate metabolic process; ribose phosphate metabolic process; purine nucleoside triphosphate biosynthetic process; heterocycle biosynthetic process; purine ribonucleoside triphosphate biosynthetic process; small molecule metabolic process; ATP biosynthetic process; single-organism cellular process; purine ribonucleoside triphosphate metabolic process; organic cyclic compound metabolic process; purine-containing compound metabolic process; ribonucleoside triphosphate metabolic process; purine ribonucleoside monophosphate biosynthetic process; purine nucleoside biosynthetic process; organophosphate biosynthetic process; ribonucleoside monophosphate	intrinsic component of membrane; membrane; integral component of membrane; membrane part;	ATPase activity, coupled to transmembrane movement of ions; nucleoside phosphate binding; primary active transmembrane transporter activity; substrate-specific transporter activity; hydrolase activity, acting on acid anhydrides; hydrolase activity; substrate-specific transmembrane transporter activity; ATPase activity; organic cyclic compound binding; heterocyclic compound binding; metal ion transmembrane transporter activity; pyrophosphatase activity; cation-transporting ATPase activity; catalytic activity; ribonucleotide binding; ATPase activity, coupled to movement of substances; binding; purine ribonucleoside binding; cation binding; hydrolase activity, acting on acid anhydrides, catalyzing transmembrane movement of substances; anion binding; adenyl ribonucleotide binding; adenyl nucleotide binding; inorganic cation transmembrane transporter activity; hydrolase activity, acting on acid anhydrides, in phosphorus-containing anhydrides; transporter activity; purine nucleotide binding; purine ribonucleoside triphosphate binding; active transmembrane transporter activity; ATP binding; ion transmembrane transporter activity; ion binding; nucleoside binding; nucleotide binding; nucleoside-triphosphatase activity; ribonucleoside binding; P-P-bond-hydrolysis-driven transmembrane transporter activity; ATPase activity, coupled to transmembrane movement of ions, phosphorylative mechanism; divalent inorganic cation transmembrane transporter activity; carbohydrate derivative binding; calcium-transporting ATPase activity; ATPase activity, coupled to transmembrane movement of substances; ATPase activity, coupled; metal ion binding; calcium ion transmembrane transporter activity; purine ribonucleotide binding; purine nucleoside binding; cation transmembrane transporter activity; transmembrane transporter activity; small molecule binding;	K05853	ATP2A; Ca2+ transporting ATPase, sarcoplasmic/endoplasmic reticulum [EC:3.6.3.8]	dre04020 Calcium signaling pathway - Danio rerio (zebrafish);	IPR023298; IPR004014; IPR023299; IPR008250; IPR006068; IPR023214;	P-type ATPase, transmembrane domain; Cation-transporting P-type ATPase, N-terminal; P-type ATPase, cytoplasmic domain N; P-type ATPase, A domain; Cation-transporting P-type ATPase, C-terminal; HAD-like domain;
Q5U3A4	atp2a1	Calcium-transporting ATPase	_DK(cr)IMAVIK_	544	1	LTGPVKDKIMAVIKE	......DK.......	plasma membrane	regulation of biological quality; nucleoside metabolic process; purine nucleoside metabolic process; cellular divalent inorganic cation homeostasis; purine ribonucleoside monophosphate metabolic process; nucleotide metabolic process; positive regulation of muscle contraction; negative regulation of multicellular organismal process; single-organism process; purine ribonucleoside biosynthetic process; nucleoside biosynthetic process; biosynthetic process; ribonucleoside monophosphate biosynthetic process; cellular metabolic process; phosphate-containing compound metabolic process; response to external stimulus; ribose phosphate biosynthetic process; metabolic process; carbohydrate derivative biosynthetic process; positive regulation of multicellular organismal process; nucleoside phosphate metabolic process; regulation of skeletal muscle contraction; metal ion homeostasis; regulation of system process; ribose phosphate metabolic process; purine nucleoside triphosphate biosynthetic process; heterocycle biosynthetic process; purine ribonucleoside triphosphate biosynthetic process; ion homeostasis; calcium ion homeostasis; small molecule metabolic process; negative regulation of striated muscle contraction; inorganic ion	membrane; endoplasmic reticulum; nuclear outer membrane-endoplasmic reticulum membrane network; organelle membrane; myofibril; cytoplasm; intrinsic component of membrane; intracellular part; sarcoplasm; cell; I band; organelle part; non-membrane-bounded organelle; intracellular; cell part; membrane part; contractile fiber; membrane region; perinuclear region of cytoplasm; membrane-bounded organelle; endoplasmic reticulum membrane; intracellular organelle; bounding membrane of organelle; intracellular non-membrane-bounded organelle; H zone; intracellular organelle part; sarcoplasmic reticulum; endomembrane system; integral component of membrane; organelle; intracellular membrane-bounded organelle; endoplasmic reticulum part; endoplasmic reticulum-Golgi intermediate compartment; A band; sarcomere; contractile fiber part; cytoplasmic part;	ATPase activity, coupled to transmembrane movement of ions; nucleoside phosphate binding; primary active transmembrane transporter activity; substrate-specific transporter activity; hydrolase activity, acting on acid anhydrides; hydrolase activity; substrate-specific transmembrane transporter activity; ATPase activity; organic cyclic compound binding; heterocyclic compound binding; metal ion transmembrane transporter activity; pyrophosphatase activity; cation-transporting ATPase activity; catalytic activity; ribonucleotide binding; ATPase activity, coupled to movement of substances; binding; purine ribonucleoside binding; cation binding; hydrolase activity, acting on acid anhydrides, catalyzing transmembrane movement of substances; anion binding; adenyl ribonucleotide binding; adenyl nucleotide binding; inorganic cation transmembrane transporter activity; hydrolase activity, acting on acid anhydrides, in phosphorus-containing anhydrides; transporter activity; purine nucleotide binding; purine ribonucleoside triphosphate binding; active transmembrane transporter activity; ATP binding; ion transmembrane transporter activity; ion binding; nucleoside binding; nucleotide binding; nucleoside-triphosphatase activity; ribonucleoside binding; P-P-bond-hydrolysis-driven transmembrane transporter activity; ATPase activity, coupled to transmembrane movement of ions, phosphorylative mechanism; divalent inorganic cation transmembrane transporter activity; carbohydrate derivative binding; calcium-transporting ATPase activity; ATPase activity, coupled to transmembrane movement of substances; ATPase activity, coupled; calcium ion transmembrane transporter activity; metal ion binding; purine ribonucleotide binding; purine nucleoside binding; calcium ion binding; cation transmembrane transporter activity; transmembrane transporter activity; small molecule binding;	K05853	ATP2A; Ca2+ transporting ATPase, sarcoplasmic/endoplasmic reticulum [EC:3.6.3.8]	dre04020 Calcium signaling pathway - Danio rerio (zebrafish);	IPR004014; IPR023298; IPR008250; IPR023299; IPR006068; IPR023214;	Cation-transporting P-type ATPase, N-terminal; P-type ATPase, transmembrane domain; P-type ATPase, A domain; P-type ATPase, cytoplasmic domain N; Cation-transporting P-type ATPase, C-terminal; HAD-like domain;

O42248	gnb2l1	Guanine nucleotide-binding protein subunit beta-2-like 1	_DK(cr)TIIMWK_	38	1	ILSASRDKTIIMWKL	......DK.......	cytosol,nuclear	positive regulation of embryonic development; regulation of cellular process; circulatory system development; morphogenesis of an epithelium; single-multicellular organism process; negative regulation of signal transduction; blood vessel development; regulation of signaling; single-organism process; negative regulation of biological process; negative regulation of cellular process; regulation of multicellular organismal process; gastrulation; anatomical structure morphogenesis; tissue morphogenesis; angiogenesis; regulation of cell communication; negative regulation of signaling; regulation of gastrulation; negative regulation of cell communication; positive regulation of multicellular organismal process; regulation of localization; regulation of protein localization; blood vessel morphogenesis; multicellular organismal development; embryo development; regulation of signal transduction; anatomical structure formation involved in morphogenesis; embryonic morphogenesis; developmental process; epithelium development; regulation of establishment or maintenance of cell polarity; tissue development; negative regulation of Wnt signaling pathway; convergent extension; system development; vasculature development;	intracellular part; cell; cell part; intracellular; cytoplasm;		K14753			IPR017986; IPR015943;	WD40-repeat-containing domain; WD40/YVTN repeat-like-containing domain;
E9QDY3	cyt1	Uncharacterized protein	_DLESWFQAK(cr)SESLNK_	297	1	LESWFQAKSESLNKE	.......K.E.....	mitochondria		intracellular organelle; intracellular non-membrane-bounded organelle; cytoskeletal part; intracellular organelle part; macromolecular complex; intracellular part; cell; organelle part; non-membrane-bounded organelle; organelle; cytoskeleton; intermediate filament cytoskeleton; cell part; intracellular; intermediate filament; protein complex;	structural molecule activity;	K07604				
Q6IQ92	tnni1al	Troponin 1	_DLHQK(cr)IDVVDEER_	73	1	LCRDLHQKIDVVDEE		mitochondria								
E7F2T3	si:ch1073-159d7.7	Uncharacterized protein	_DLIVK(cr)TVTASK_	53	1	NVRDLIVKTVTASKE	.......K.V.....	nuclear	cellular component biogenesis; cellular component organization; nucleosome organization; cellular macromolecular complex assembly; chromatin assembly or disassembly; protein complex subunit organization; macromolecular complex subunit organization; protein-DNA complex assembly; DNA packaging; nucleosome assembly; chromosome organization; cellular component assembly; macromolecular complex assembly; organelle organization; chromatin organization; protein complex biogenesis; chromatin assembly; cellular process; protein complex assembly; cellular component organization or biogenesis; protein-DNA complex subunit organization; DNA conformation change;	intracellular organelle; intracellular non-membrane-bounded organelle; intracellular organelle part; macromolecular complex; intracellular part; cell; organelle part; DNA bending complex; chromosomal part; nucleosome; non-membrane-bounded organelle; intracellular membrane-bounded organelle; organelle; nucleus; chromatin; cell part; intracellular; chromosome; membrane-bounded organelle; protein complex; protein-DNA complex; DNA packaging complex;	organic cyclic compound binding; heterocyclic compound binding; DNA binding; binding; nucleic acid binding;				IPR011991; IPR005818;	Winged helix-turn-helix DNA-binding domain; Linker histone H1/H5, domain H15;
E7EZ16	-	Uncharacterized protein	_DLK(cr)IDIIPNVQER_	71	1	LDSGKDLKIDIIPNV		cytosol	protein folding; metabolic process; response to stress; cellular protein metabolic process; cellular macromolecule metabolic process; macromolecule metabolic process; protein metabolic process; response to stimulus; primary metabolic process; cellular process; cellular metabolic process; organic substance metabolic process;		ATP binding; nucleoside phosphate binding; nucleoside binding; ion binding; nucleotide binding; organic cyclic compound binding; heterocyclic compound binding; ribonucleoside binding; carbohydrate derivative binding; ribonucleotide binding; binding; purine ribonucleoside binding; anion binding; purine ribonucleotide binding; adenyl ribonucleotide binding; adenyl nucleotide binding; purine nucleoside binding; purine nucleotide binding; small molecule binding; purine ribonucleoside triphosphate binding;	K04079	htpG; molecular chaperone HtpG	dre04914 Progesterone-mediated oocyte maturation - Danio rerio (zebrafish); dre04141 Protein processing in endoplasmic reticulum - Danio rerio (zebrafish); dre04621 NOD-like receptor signaling pathway - Danio rerio (zebrafish);	IPR020568; IPR020575; IPR003594;	Ribosomal protein S5 domain 2-type fold; Heat shock protein Hsp90, N-terminal; Histidine kinase-like ATPase, C-terminal domain;
Q9I8V1	actc1b	Actin, alpha 1, skeletal muscle	_DLTDYLMK(cr)ILTER_	193	1	DLTDYLMKILTERGY	.......K.L.....	cytoskeleton		cytoskeleton; organelle; intracellular organelle; intracellular non-membrane-bounded organelle; cell part; intracellular; cytoplasm; intracellular part; cell; non-membrane-bounded organelle;	ATP binding; nucleoside phosphate binding; nucleoside binding; ion binding; nucleotide binding; organic cyclic compound binding; heterocyclic compound binding; ribonucleoside binding; carbohydrate derivative binding; ribonucleotide binding; binding; purine ribonucleoside binding; anion binding; purine ribonucleotide binding; adenyl ribonucleotide binding; adenyl nucleotide binding; purine nucleoside binding; purine nucleotide binding; small molecule binding; purine ribonucleoside triphosphate binding;	K10354				

Q90474	hsp90a.1	Heat shock protein HSP 90-alpha 1	_DTQK(cr)HIYYITGETK_	482	1	SRMKDTQKHIYYITG	.......K.I.....	cytosol	cellular component biogenesis; regulation of nitric oxide biosynthetic process; cellular developmental process; positive regulation of biosynthetic process; cellular component morphogenesis; single-organism process; cellular metabolic process; protein folding; actin cytoskeleton organization; metabolic process; cellular component assembly; positive regulation of metabolic process; macromolecular complex assembly; positive regulation of nitrogen compound metabolic process; protein metabolic process; macromolecule metabolic process; positive regulation of nitric oxide biosynthetic process; striated muscle myosin thick filament assembly; skeletal muscle thin filament assembly; skeletal muscle myosin thick filament assembly; developmental process; actomyosin structure organization; protein complex subunit organization; regulation of cellular biosynthetic process; single-organism cellular process; positive regulation of cellular process; myosin filament organization; regulation of cellular metabolic process; myosin filament assembly; sarcomerogenesis; multicellular organismal process; single-organism organelle organization; organ development; regulation of biological process; striated muscle cell	intracellular organelle; intracellular non-membrane-bounded organelle; cytoplasmic vesicle; membrane-bounded vesicle; myofibril; melanosome; cytoplasmic membrane-bounded vesicle; vesicle; cytoplasm; intracellular part; Z disc; cell; I band; organelle part; pigment granule; non-membrane-bounded organelle; intracellular membrane-bounded organelle; organelle; cell part; intracellular; sarcomere; contractile fiber; perinuclear region of cytoplasm; contractile fiber part; membrane-bounded organelle; cytoplasmic part;	nitric-oxide synthase regulator activity; ATP binding; nucleoside phosphate binding; nucleoside binding; ion binding; nucleotide binding; molecular function regulator; organic cyclic compound binding; heterocyclic compound binding; ribonucleoside binding; carbohydrate derivative binding; enzyme regulator activity; ribonucleotide binding; binding; purine ribonucleoside binding; anion binding; purine ribonucleotide binding; adenyl ribonucleotide binding; adenyl nucleotide binding; purine nucleoside binding; purine nucleotide binding; small molecule binding; purine ribonucleoside triphosphate binding;	K04079	htpG; molecular chaperone HtpG	dre04914 Progesterone-mediated oocyte maturation - Danio rerio (zebrafish); dre04141 Protein processing in endoplasmic reticulum - Danio rerio (zebrafish); dre04621 NOD-like receptor signaling pathway - Danio rerio (zebrafish);	IPR020568; IPR020575; IPR003594;	Ribosomal protein S5 domain 2-type fold; Heat shock protein Hsp90, N-terminal; Histidine kinase-like ATPase, C-terminal domain;
Q90ZM2	sec61al1	Protein transport protein Sec61 subunit alpha-like 1	_DVAK(cr)QLK_	392	1	SSAKDVAKQLKEQQM	.......K.L.....	plasma membrane	developmental process; nervous system development; single-multicellular organism process; brain development; system development; single-organism process; organic substance transport; multicellular organismal process; protein transport; establishment of localization; establishment of protein localization; transport; organ development; habenula development; single-organism developmental process; diencephalon development; central nervous system development; head development; forebrain development; multicellular organismal development; protein localization; macromolecule localization; localization; anatomical structure development; epithalamus development;	membrane; intracellular organelle; endoplasmic reticulum; nuclear outer membrane-endoplasmic reticulum membrane network; bounding membrane of organelle; organelle membrane; cytoplasm; intracellular organelle part; intrinsic component of membrane; endomembrane system; intracellular part; cell; integral component of membrane; organelle part; intracellular membrane-bounded organelle; organelle; endoplasmic reticulum part; intracellular; cell part; membrane part; membrane region; membrane-bounded organelle; endoplasmic reticulum membrane; cytoplasmic part;		K10956	SEC61A; protein transport protein SEC61 subunit alpha	dre03060 Protein export - Danio rerio (zebrafish); dre04145 Phagosome - Danio rerio (zebrafish); dre04141 Protein processing in endoplasmic reticulum - Danio rerio (zebrafish);	IPR023201; IPR019561;	SecY subunit domain; Translocon Sec61/SecY, plug domain;
Q6NWF6	krt8	Keratin, type II cytoskeletal 8	_DVDEAYMNK(cr)VELEAK_	231	1	VDEAYMNKVELEAKL	..E....K.......	mitochondria		intracellular organelle; intracellular non-membrane-bounded organelle; cytoskeletal part; intracellular organelle part; cytoplasm; nuclear lumen; macromolecular complex; intracellular part; cell; organelle part; keratin filament; non-membrane-bounded organelle; membrane-enclosed lumen; intracellular membrane-bounded organelle; organelle; cytoskeleton; nucleoplasm; nucleus; intracellular organelle lumen; intermediate filament cytoskeleton; intracellular; cell part; nuclear matrix; intermediate filament; nuclear periphery; membrane-bounded organelle; protein complex; organelle lumen; nuclear part;	structural molecule activity;	K07605				
B0S564	-	Protein disulfide-isomerase	_DVESEDSK(cr)AFIK_	168	1	DVESEDSKAFIKTAE	..E....K.......	extracellular	regulation of cellular process; regulation of biological quality; small molecule metabolic process; cell redox homeostasis; single-organism cellular process; ether metabolic process; cellular homeostasis; single-organism process; organic substance metabolic process; homeostatic process; metabolic process; regulation of biological process; single-organism metabolic process; glycerol ether metabolic process; cellular process; biological regulation;	organelle; intracellular membrane-bounded organelle; intracellular organelle; endoplasmic reticulum; cell part; intracellular; cytoplasm; endomembrane system; macromolecular complex; intracellular part; membrane-bounded organelle; cell; protein complex; collagen trimer; cytoplasmic part;	protein disulfide oxidoreductase activity; disulfide oxidoreductase activity; oxidoreductase activity, acting on single donors with incorporation of molecular oxygen, incorporation of two atoms of oxygen; electron carrier activity; oxidoreductase activity, acting on single donors with incorporation of molecular oxygen; isomerase activity; catalytic activity; dioxygenase activity; oxidoreductase activity, acting on a sulfur group of donors; oxidoreductase activity;	K09580	PDIA1; protein disulfide-isomerase A1 [EC:5.3.4.1]	dre04141 Protein processing in endoplasmic reticulum - Danio rerio (zebrafish);	IPR005788; IPR013766; IPR012336;	Disulphide isomerase; Thioredoxin domain; Thioredoxin-like fold;
O93409	mylpfa	Mylz2 protein	_DVLASMGQLNVK(cr)NEELEAMIK_	64	1	SMGQLNVKNEELEAM	.......K.E.....	cytosol,nuclear			cation binding; metal ion binding; calcium ion binding; binding; ion binding;	K12758	MYLPF; fast skeletal myosin light chain 2	dre04510 Focal adhesion - Danio rerio (zebrafish); dre04530 Tight junction - Danio rerio (zebrafish); dre04810 Regulation of actin cytoskeleton - Danio rerio (zebrafish);	IPR011992; IPR002048;	EF-hand domain pair; EF-hand domain;

B3DFP9	apoa2	Uncharacterized protein	_DVVEELK(cr)K_	76	1	KDVVEELKKSPRVES		extracellular	developmental process; cellular component organization; single-multicellular organism process; morphogenesis of an epithelium; epithelium development; epiboly involved in gastrulation with mouth forming second; tissue development; organelle fission; epiboly; morphogenesis of an epithelial sheet; single-organism process; multicellular organismal process; gastrulation; anatomical structure morphogenesis; gastrulation with mouth forming second; tissue morphogenesis; organelle organization; single-organism developmental process; nuclear division; multicellular organismal development; embryo development; cellular process; anatomical structure development; embryonic morphogenesis; cellular component organization or biogenesis;							
Q90Z10	rpl13	60S ribosomal protein L13	_EAAEQDVEK(cr)K_	209	1	AAEQDVEKK_____		cytosol	regulation of cellular process; cellular protein metabolic process; biosynthetic process; primary metabolic process; organic substance biosynthetic process; cellular metabolic process; cellular biosynthetic process; organic substance metabolic process; metabolic process; regulation of biological process; cellular macromolecule biosynthetic process; cellular macromolecule metabolic process; gene expression; protein metabolic process; macromolecule metabolic process; translation; macromolecule biosynthetic process; cellular process; regulation of cell cycle; biological regulation;	ribosome; organelle; intracellular organelle; intracellular non-membrane-bounded organelle; cell part; intracellular; cytoplasm; macromolecular complex; intracellular part; cell; ribonucleoprotein complex; cytoplasmic part; non-membrane-bounded organelle;	structural constituent of ribosome; structural molecule activity;	K02873	RP-L13e; large subunit ribosomal protein L13e	dre03010 Ribosome - Danio rerio (zebrafish);		
Q6PC90	rps12	40S ribosomal protein S12	_EAAK(cr)ALDK_	40	1	RGIREAAKALDKRQA	.......K.L.....	cytosol	cellular protein metabolic process; biosynthetic process; primary metabolic process; cellular metabolic process; organic substance biosynthetic process; cellular biosynthetic process; organic substance metabolic process; metabolic process; cellular macromolecule biosynthetic process; cellular macromolecule metabolic process; gene expression; macromolecule metabolic process; protein metabolic process; macromolecule biosynthetic process; translation; cellular process;	ribosome; organelle; intracellular organelle; intracellular non-membrane-bounded organelle; cell part; intracellular; cytoplasm; macromolecular complex; intracellular part; cell; ribonucleoprotein complex; cytoplasmic part; non-membrane-bounded organelle;	structural constituent of ribosome; structural molecule activity;	K02951	RP-S12e; small subunit ribosomal protein S12e	dre03010 Ribosome - Danio rerio (zebrafish);	IPR029064; IPR004038;	50S ribosomal protein L30e-like; Ribosomal protein L7Ae/L30e/S12e/ Gadd45;
Q7T3L3	hsp90b1	Chaperone protein GP96	_EAESSPFVEK(cr)LLK_	557	1	ESSPFVEKLLKKGYE	.......K.L.....	endoplasmic reticulum	developmental process; single-multicellular organism process; response to stress; system development; cellular protein metabolic process; single-organism process; response to stimulus; primary metabolic process; otolith development; inner ear development; cellular metabolic process; multicellular organismal process; organic substance metabolic process; otolith tethering; protein folding; metabolic process; organ development; ear development; single-organism developmental process; cellular macromolecule metabolic process; protein metabolic process; macromolecule metabolic process; multicellular organismal development; sensory organ development; cellular process; anatomical structure development;		ATP binding; nucleoside phosphate binding; nucleoside binding; ion binding; nucleotide binding; organic cyclic compound binding; heterocyclic compound binding; ribonucleoside binding; carbohydrate derivative binding; ribonucleotide binding; binding; purine ribonucleoside binding; anion binding; purine ribonucleotide binding; adenyl ribonucleotide binding; adenyl nucleotide binding; purine nucleoside binding; purine nucleotide binding; small molecule binding; purine ribonucleoside triphosphate binding;	K09487	HSP90B; heat shock protein 90kDa beta	dre04141 Protein processing in endoplasmic reticulum - Danio rerio (zebrafish); dre04621 NOD-like receptor signaling pathway - Danio rerio (zebrafish);	IPR020568; IPR020575; IPR003594;	Ribosomal protein S5 domain 2-type fold; Heat shock protein Hsp90, N-terminal; Histidine kinase-like ATPase, C-terminal domain;
Q7ZV05	rps11	Ribosomal protein S11	_EAIDGTYIDK(cr)K_	59	1	IDGTYIDKKCPFTGN	......DK.......	cytosol	cellular protein metabolic process; biosynthetic process; primary metabolic process; cellular metabolic process; organic substance biosynthetic process; cellular biosynthetic process; organic substance metabolic process; metabolic process; cellular macromolecule biosynthetic process; cellular macromolecule metabolic process; gene expression; macromolecule metabolic process; protein metabolic process; macromolecule biosynthetic process; translation; cellular process;	ribosome; organelle; intracellular organelle; intracellular non-membrane-bounded organelle; cell part; intracellular; cytoplasm; macromolecular complex; intracellular part; cell; ribonucleoprotein complex; cytoplasmic part; non-membrane-bounded organelle;	structural constituent of ribosome; structural molecule activity;	K02949	RP-S11e; small subunit ribosomal protein S11e	dre03010 Ribosome - Danio rerio (zebrafish);	IPR012340;	Nucleic acid-binding, OB-fold;
P13104	tpma	Uncharacterized protein	_EAK(cr)HIAEEADR_	152	1	EIQLKEAKHIAEEAD	.......K.I.....	cytosol		cytoskeleton; organelle; intracellular organelle; intracellular non-membrane-bounded organelle; cell part; intracellular; cytoplasm; intracellular part; cell; non-membrane-bounded organelle;		K10373	TPM1; tropomyosin 1	dre04260 Cardiac muscle contraction - Danio rerio (zebrafish); dre04261 Adrenergic signaling in cardiomyocytes - Danio rerio (zebrafish);		
B8A569	myhz1.3	Uncharacterized protein	_EALAK(cr)AEAK_	867	1	KCKEALAKAEAKKKE	.......K.E.....	nuclear		intracellular organelle; intracellular non-membrane-bounded organelle; cytoskeletal part; intracellular organelle part; macromolecular complex; intracellular part; cell; actin cytoskeleton; organelle part; non-membrane-bounded organelle; organelle; cytoskeleton; cell part; intracellular; myosin complex; protein complex;	ATP binding; motor activity; nucleoside phosphate binding; nucleoside binding; ion binding; hydrolase activity, acting on acid anhydrides; hydrolase activity; nucleotide binding; nucleoside-triphosphatase activity; organic cyclic compound binding; heterocyclic compound binding; ribonucleoside binding; carbohydrate derivative binding; pyrophosphatase activity; catalytic activity; ribonucleotide binding; binding; purine ribonucleoside binding; anion binding; purine ribonucleotide binding; adenyl ribonucleotide binding; adenyl nucleotide binding; purine nucleoside binding; hydrolase activity, acting on acid anhydrides, in phosphorus-containing anhydrides; purine nucleotide binding; small molecule binding; purine ribonucleoside triphosphate binding;				IPR002928; IPR004009; IPR027417; IPR001609; IPR027401;	Myosin tail; Myosin, N-terminal, SH3-like; P-loop containing nucleoside triphosphate hydrolase; Myosin head, motor domain; Myosin-like IQ motif-containing domain;

F1R0W9	dctn1a	Uncharacterized protein	_EALEAK(cr)DR_	239	1	AREALEAKDRYMEEM	..E....K.......	cytosol				K04648			IPR000938; IPR022157;	CAP Gly-rich domain; Dynein associated protein;
Q6IQX1	myhz2	Myosin, heavy polypeptide 2, fast muscle specific	_EDDVHPM(ox)NPPK(cr)FDK_	85	1	VHPMNPPKFDKIEDM		cytosol	response to stress; response to stimulus;	intracellular organelle; intracellular non-membrane-bounded organelle; cytoskeletal part; intracellular organelle part; macromolecular complex; intracellular part; cell; actin cytoskeleton; organelle part; non-membrane-bounded organelle; organelle; cytoskeleton; cell part; intracellular; myosin complex; protein complex;	ATP binding; motor activity; nucleoside phosphate binding; nucleoside binding; ion binding; hydrolase activity, acting on acid anhydrides; hydrolase activity; nucleotide binding; nucleoside-triphosphatase activity; organic cyclic compound binding; heterocyclic compound binding; ribonucleoside binding; carbohydrate derivative binding; pyrophosphatase activity; catalytic activity; ribonucleotide binding; binding; purine ribonucleoside binding; anion binding; purine ribonucleotide binding; adenyl ribonucleotide binding; adenyl nucleotide binding; purine nucleoside binding; hydrolase activity, acting on acid anhydrides, in phosphorus-containing anhydrides; purine nucleotide binding; small molecule binding; purine ribonucleoside triphosphate binding;	K10352	MYH; myosin heavy chain	dre04530 Tight junction - Danio rerio (zebrafish);	IPR002928; IPR004009; IPR001609; IPR027417; IPR027401;	Myosin tail; Myosin, N-terminal, SH3-like; Myosin head, motor domain; P-loop containing nucleoside triphosphate hydrolase; Myosin-like IQ motif-containing domain;
B8A568	myhz1.1	Uncharacterized protein	_EDFVK(cr)CK_	860	1	TMKEDFVKCKEDLAK		nuclear		intracellular organelle; intracellular non-membrane-bounded organelle; cytoskeletal part; intracellular organelle part; macromolecular complex; intracellular part; cell; actin cytoskeleton; organelle part; non-membrane-bounded organelle; organelle; cytoskeleton; cell part; intracellular; myosin complex; protein complex;	ATP binding; motor activity; nucleoside phosphate binding; nucleoside binding; ion binding; hydrolase activity, acting on acid anhydrides; hydrolase activity; nucleotide binding; nucleoside-triphosphatase activity; organic cyclic compound binding; heterocyclic compound binding; ribonucleoside binding; carbohydrate derivative binding; pyrophosphatase activity; catalytic activity; ribonucleotide binding; binding; purine ribonucleoside binding; anion binding; purine ribonucleotide binding; adenyl ribonucleotide binding; adenyl nucleotide binding; purine nucleoside binding; hydrolase activity, acting on acid anhydrides, in phosphorus-containing anhydrides; purine nucleotide binding; small molecule binding; purine ribonucleoside triphosphate binding;	K10352	MYH; myosin heavy chain	dre04530 Tight junction - Danio rerio (zebrafish);	IPR002928; IPR004009; IPR001609; IPR027417; IPR027401;	Myosin tail; Myosin, N-terminal, SH3-like; Myosin head, motor domain; P-loop containing nucleoside triphosphate hydrolase; Myosin-like IQ motif-containing domain;
Q6P0W3	tpm3	Uncharacterized protein	EDKYEEEIK(cr)ILTDK	226	1	DKYEEEIKILTDKLK	.......K.L.....	cytosol								
P13104	tpma	Uncharacterized protein	_EDKYEEEIK(cr)VLTDK_	226	1	DKYEEEIKVLTDKLK	.......K.L.....	cytosol		cytoskeleton; organelle; intracellular organelle; intracellular non-membrane-bounded organelle; cell part; intracellular; cytoplasm; intracellular part; cell; non-membrane-bounded organelle;		K10373	TPM1; tropomyosin 1	dre04260 Cardiac muscle contraction - Danio rerio (zebrafish); dre04261 Adrenergic signaling in cardiomyocytes - Danio rerio (zebrafish);		
B8A568	myhz1.1	Uncharacterized protein	_EDLAK(cr)AEAK_	867	1	KCKEDLAKAEAKKKE	.......K.E.....	nuclear		intracellular organelle; intracellular non-membrane-bounded organelle; cytoskeletal part; intracellular organelle part; macromolecular complex; intracellular part; cell; actin cytoskeleton; organelle part; non-membrane-bounded organelle; organelle; cytoskeleton; cell part; intracellular; myosin complex; protein complex;	ATP binding; motor activity; nucleoside phosphate binding; nucleoside binding; ion binding; hydrolase activity, acting on acid anhydrides; hydrolase activity; nucleotide binding; nucleoside-triphosphatase activity; organic cyclic compound binding; heterocyclic compound binding; ribonucleoside binding; carbohydrate derivative binding; pyrophosphatase activity; catalytic activity; ribonucleotide binding; binding; purine ribonucleoside binding; anion binding; purine ribonucleotide binding; adenyl ribonucleotide binding; adenyl nucleotide binding; purine nucleoside binding; hydrolase activity, acting on acid anhydrides, in phosphorus-containing anhydrides; purine nucleotide binding; small molecule binding; purine ribonucleoside triphosphate binding;	K10352	MYH; myosin heavy chain	dre04530 Tight junction - Danio rerio (zebrafish);	IPR002928; IPR004009; IPR001609; IPR027417; IPR027401;	Myosin tail; Myosin, N-terminal, SH3-like; Myosin head, motor domain; P-loop containing nucleoside triphosphate hydrolase; Myosin-like IQ motif-containing domain;
B8A561	myhz1.2	Uncharacterized protein	_EDLAK(cr)AEAK_	867	1	KCKEDLAKAEAKKKE	.......K.E.....	nuclear		intracellular organelle; intracellular non-membrane-bounded organelle; cytoskeletal part; intracellular organelle part; macromolecular complex; intracellular part; cell; actin cytoskeleton; organelle part; non-membrane-bounded organelle; organelle; cytoskeleton; cell part; intracellular; myosin complex; protein complex;	ATP binding; motor activity; nucleoside phosphate binding; nucleoside binding; ion binding; hydrolase activity, acting on acid anhydrides; hydrolase activity; nucleotide binding; nucleoside-triphosphatase activity; organic cyclic compound binding; heterocyclic compound binding; ribonucleoside binding; carbohydrate derivative binding; pyrophosphatase activity; catalytic activity; ribonucleotide binding; binding; purine ribonucleoside binding; anion binding; purine ribonucleotide binding; adenyl ribonucleotide binding; adenyl nucleotide binding; purine nucleoside binding; hydrolase activity, acting on acid anhydrides, in phosphorus-containing anhydrides; purine nucleotide binding; small molecule binding; purine ribonucleoside triphosphate binding;	K10352	MYH; myosin heavy chain	dre04530 Tight junction - Danio rerio (zebrafish);	IPR002928; IPR004009; IPR001609; IPR027417; IPR027401;	Myosin tail; Myosin, N-terminal, SH3-like; Myosin head, motor domain; P-loop containing nucleoside triphosphate hydrolase; Myosin-like IQ motif-containing domain;
E7EXP0	tnnt3b	Uncharacterized protein	EEEELIALK(cr)DR	72	1	EEELIALKDRIEKRR	..E....K.......	nuclear				K12046				
Q6NYR4	hspa8	Heat shock cognate 71 kDa protein	_EEFEHQQK(cr)ELEK_	597	1	EEFEHQQKELEKVCN	.......K.L.....	cytosol	response to stress; response to stimulus;		ATP binding; nucleoside phosphate binding; nucleoside binding; ion binding; nucleotide binding; organic cyclic compound binding; heterocyclic compound binding; ribonucleoside binding; carbohydrate derivative binding; ribonucleotide binding; binding; purine ribonucleoside binding; anion binding; purine ribonucleotide binding; adenyl ribonucleotide binding; adenyl nucleotide binding; purine nucleoside binding; purine nucleotide binding; small molecule binding; purine ribonucleoside triphosphate binding;	K03283	HSPA1_8; heat shock 70kDa protein 1/8	dre04010 MAPK signaling pathway - Danio rerio (zebrafish); dre04141 Protein processing in endoplasmic reticulum - Danio rerio (zebrafish); dre04144 Endocytosis - Danio rerio (zebrafish); dre03040 Spliceosome - Danio rerio (zebrafish);	IPR029048; IPR029047;	Heat shock protein 70kD, C-terminal domain; Heat shock protein 70kD, peptide-binding domain;
Q90473	hspa8	Heat shock cognate 71 kDa protein	_EEFEHQQK(cr)ELEK_	597	1	EEFEHQQKELEKVCN	.......K.L.....	cytosol	developmental process; response to wounding; tissue development; response to stress; wound healing; tissue regeneration; single-organism developmental process; fin regeneration; single-organism process; regeneration; developmental growth; response to stimulus; growth; anatomical structure development;		ATP binding; nucleoside phosphate binding; nucleoside binding; ion binding; nucleotide binding; organic cyclic compound binding; heterocyclic compound binding; ribonucleoside binding; carbohydrate derivative binding; ribonucleotide binding; binding; purine ribonucleoside binding; anion binding; purine ribonucleotide binding; adenyl ribonucleotide binding; adenyl nucleotide binding; purine nucleoside binding; purine nucleotide binding; small molecule binding; purine ribonucleoside triphosphate binding;	K03283	HSPA1_8; heat shock 70kDa protein 1/8	dre04010 MAPK signaling pathway - Danio rerio (zebrafish); dre04141 Protein processing in endoplasmic reticulum - Danio rerio (zebrafish); dre04144 Endocytosis - Danio rerio (zebrafish); dre03040 Spliceosome - Danio rerio (zebrafish);	IPR029048; IPR029047;	Heat shock protein 70kD, C-terminal domain; Heat shock protein 70kD, peptide-binding domain;
Q5TZ29	apobb.1	Uncharacterized protein	_EEIQK(cr)FLK_	2362	1	DIREEIQKFLKHASS	.......K.L.....	endoplasmic reticulum	response to chemical; response to stimulus;		lipid transporter activity; substrate-specific transporter activity; transporter activity;				IPR011030; IPR015817; IPR015818; IPR001747; IPR015819; IPR015816; IPR016024; IPR015255; IPR009454;	Vitellinogen, superhelical; Vitellinogen, open beta-sheet, subdomain 1; Vitellinogen, open beta-sheet, subdomain 2; Lipid transport protein, N-terminal; Lipid transport protein, beta-sheet shell; Vitellinogen, beta-sheet N-terminal; Armadillo-type fold; Vitellinogen, open beta-sheet; Lipid transport, open beta-sheet;

B8A568	myhz1.1	Uncharacterized protein	_EEK(cr)VVKEDDVHPM(ox)NPPK_	71	1	TLDTKEEKVVKEDDV	.......K.V.....	nuclear		intracellular organelle; intracellular non-membrane-bounded organelle; cytoskeletal part; intracellular organelle part; macromolecular complex; intracellular part; cell; actin cytoskeleton; organelle part; non-membrane-bounded organelle; organelle; cytoskeleton; cell part; intracellular; myosin complex; protein complex;	ATP binding; motor activity; nucleoside phosphate binding; nucleoside binding; ion binding; hydrolase activity, acting on acid anhydrides; hydrolase activity; nucleotide binding; nucleoside-triphosphatase activity; organic cyclic compound binding; heterocyclic compound binding; ribonucleoside binding; carbohydrate derivative binding; pyrophosphatase activity; catalytic activity; ribonucleotide binding; binding; purine ribonucleoside binding; anion binding; purine ribonucleotide binding; adenyl ribonucleotide binding; adenyl nucleotide binding; purine nucleoside binding; hydrolase activity, acting on acid anhydrides, in phosphorus-containing anhydrides; purine nucleotide binding; small molecule binding; purine ribonucleoside triphosphate binding;	K10352	MYH; myosin heavy chain	dre04530 Tight junction - Danio rerio (zebrafish);	IPR002928; IPR004009; IPR001609; IPR027417; IPR027401;	Myosin tail; Myosin, N-terminal, SH3-like; Myosin head, motor domain; P-loop containing nucleoside triphosphate hydrolase; Myosin-like IQ motif-containing domain;
Q6TGV6	psma5	Proteasome subunit alpha type	_EELEDVIK(cr)DI_	239	1	EELEDVIKDI_____		cytosol,nuclear	modification-dependent macromolecule catabolic process; cellular macromolecule catabolic process; proteolysis involved in cellular protein catabolic process; cellular protein catabolic process; modification-dependent protein catabolic process; cellular protein metabolic process; ubiquitin-dependent protein catabolic process; primary metabolic process; cellular metabolic process; organic substance metabolic process; organic substance catabolic process; metabolic process; protein catabolic process; macromolecule catabolic process; cellular macromolecule metabolic process; cellular catabolic process; proteolysis; protein metabolic process; macromolecule metabolic process; cellular process; catabolic process;	intracellular organelle; proteasome core complex, alpha-subunit complex; proteasome complex; cytoplasm; macromolecular complex; intracellular part; cell; intracellular membrane-bounded organelle; organelle; nucleus; intracellular; cell part; proteasome core complex; membrane-bounded organelle; protein complex;	endopeptidase activity; threonine-type endopeptidase activity; peptidase activity; catalytic activity; threonine-type peptidase activity; peptidase activity, acting on L-amino acid peptides; hydrolase activity;	K02729	PSMA5; 20S proteasome subunit alpha 5 [EC:3.4.25.1]	dre03050 Proteasome - Danio rerio (zebrafish);	IPR000426; IPR029055;	Proteasome alpha-subunit, N-terminal domain; Nucleophile aminohydrolases, N-terminal;
E9QDY3	cyt1	Uncharacterized protein	_EELIFLK(cr)K_	238	1	KEELIFLKKNHEEEL	..E....K.......	mitochondria		intracellular organelle; intracellular non-membrane-bounded organelle; cytoskeletal part; intracellular organelle part; macromolecular complex; intracellular part; cell; organelle part; non-membrane-bounded organelle; organelle; cytoskeleton; intermediate filament cytoskeleton; cell part; intracellular; intermediate filament; protein complex;	structural molecule activity;	K07604				
Q7T3L3	hsp90b1	Chaperone protein GP96	_EELVK(cr)NLGTIAK_	161	1	MTKEELVKNLGTIAK	.......K.L.....	endoplasmic reticulum	developmental process; single-multicellular organism process; response to stress; system development; cellular protein metabolic process; single-organism process; response to stimulus; primary metabolic process; otolith development; inner ear development; cellular metabolic process; multicellular organismal process; organic substance metabolic process; otolith tethering; protein folding; metabolic process; organ development; ear development; single-organism developmental process; cellular macromolecule metabolic process; protein metabolic process; macromolecule metabolic process; multicellular organismal development; sensory organ development; cellular process; anatomical structure development;		ATP binding; nucleoside phosphate binding; nucleoside binding; ion binding; nucleotide binding; organic cyclic compound binding; heterocyclic compound binding; ribonucleoside binding; carbohydrate derivative binding; ribonucleotide binding; binding; purine ribonucleoside binding; anion binding; purine ribonucleotide binding; adenyl ribonucleotide binding; adenyl nucleotide binding; purine nucleoside binding; purine nucleotide binding; small molecule binding; purine ribonucleoside triphosphate binding;	K09487	HSP90B; heat shock protein 90kDa beta	dre04141 Protein processing in endoplasmic reticulum - Danio rerio (zebrafish); dre04621 NOD-like receptor signaling pathway - Danio rerio (zebrafish);	IPR020568; IPR020575; IPR003594;	Ribosomal protein S5 domain 2-type fold; Heat shock protein Hsp90, N-terminal; Histidine kinase-like ATPase, C-terminal domain;
Q6P3J5	eef2b	Eukaryotic translation elongation factor 2	_EETAK(cr)LIEK_	314	1	FKKEETAKLIEKLDI	.......K.I.....	cytosol			nucleoside phosphate binding; translation elongation factor activity; hydrolase activity, acting on acid anhydrides; hydrolase activity; organic cyclic compound binding; heterocyclic compound binding; pyrophosphatase activity; catalytic activity; ribonucleotide binding; binding; GTPase activity; purine ribonucleoside binding; anion binding; hydrolase activity, acting on acid anhydrides, in phosphorus-containing anhydrides; guanyl nucleotide binding; GTP binding; purine nucleotide binding; purine ribonucleoside triphosphate binding; RNA binding; ion binding; nucleoside binding; nucleotide binding; nucleoside-triphosphatase activity; ribonucleoside binding; translation factor activity, nucleic acid binding; carbohydrate derivative binding; nucleic acid binding; purine ribonucleotide binding; guanyl ribonucleotide binding; purine nucleoside binding; small molecule binding;	K03234			IPR027417; IPR000795; IPR005517; IPR005225; IPR004161; IPR000640; IPR020568; IPR014721; IPR009022; IPR009000;	P-loop containing nucleoside triphosphate hydrolase; Elongation factor, GTP-binding domain; Translation elongation factor EFG/EF2, domain IV; Small GTP-binding protein domain; Translation elongation factor EFTu/EF1A, domain 2; Translation elongation factor EFG, V domain; Ribosomal protein S5 domain 2-type fold; Ribosomal protein S5 domain 2-type fold, subgroup; Elongation factor G, III-V domain; Translation protein, beta-barrel domain;
E9QFX0	apoc1	Uncharacterized protein	_EIAEDLADK(cr)TK_	47	1	IAEDLADKTKTAFQN	......DK.......	extracellular	metabolic process; macromolecule metabolic process; protein metabolic process; primary metabolic process; lipoprotein metabolic process; organic substance metabolic process;	extracellular region;						
A2BFV8	anxa1a	Annexin	_EIGDIK(cr)QVYK_	148	1	NKEIGDIKQVYKQEY	.......K.V.....	cytosol			calcium-dependent phospholipid binding; binding; phospholipid binding; ion binding; lipid binding; metal ion binding; cation binding; anion binding; calcium ion binding;					

Q642Z0	atp2a1	Calcium-transporting ATPase	_EIVPGDIVEVSVGDK(cr)VPADIR_	158	1	VEVSVGDKVPADIRI	......DK.......	plasma membrane	nucleoside metabolic process; purine nucleoside metabolic process; purine ribonucleoside monophosphate metabolic process; nucleotide metabolic process; single-organism process; purine ribonucleoside biosynthetic process; nucleoside biosynthetic process; biosynthetic process; ribonucleoside monophosphate biosynthetic process; cellular metabolic process; phosphate-containing compound metabolic process; ribose phosphate biosynthetic process; metabolic process; carbohydrate derivative biosynthetic process; nucleoside phosphate metabolic process; ribose phosphate metabolic process; purine nucleoside triphosphate biosynthetic process; heterocycle biosynthetic process; purine ribonucleoside triphosphate biosynthetic process; small molecule metabolic process; ATP biosynthetic process; single-organism cellular process; purine ribonucleoside triphosphate metabolic process; organic cyclic compound metabolic process; purine-containing compound metabolic process; ribonucleoside triphosphate metabolic process; purine ribonucleoside monophosphate biosynthetic process; purine nucleoside biosynthetic process; organophosphate biosynthetic process; ribonucleoside monophosphate	intrinsic component of membrane; membrane; integral component of membrane; membrane part;	ATPase activity, coupled to transmembrane movement of ions; nucleoside phosphate binding; primary active transmembrane transporter activity; substrate-specific transporter activity; hydrolase activity, acting on acid anhydrides; hydrolase activity; substrate-specific transmembrane transporter activity; ATPase activity; organic cyclic compound binding; heterocyclic compound binding; metal ion transmembrane transporter activity; pyrophosphatase activity; cation-transporting ATPase activity; catalytic activity; ribonucleotide binding; ATPase activity, coupled to movement of substances; binding; purine ribonucleoside binding; cation binding; hydrolase activity, acting on acid anhydrides, catalyzing transmembrane movement of substances; anion binding; adenyl ribonucleotide binding; adenyl nucleotide binding; inorganic cation transmembrane transporter activity; hydrolase activity, acting on acid anhydrides, in phosphorus-containing anhydrides; transporter activity; purine nucleotide binding; purine ribonucleoside triphosphate binding; active transmembrane transporter activity; ATP binding; ion transmembrane transporter activity; ion binding; nucleoside binding; nucleotide binding; nucleoside-triphosphatase activity; ribonucleoside binding; P-P-bond-hydrolysis-driven transmembrane transporter activity; ATPase activity, coupled to transmembrane movement of ions, phosphorylative mechanism; divalent inorganic cation transmembrane transporter activity; carbohydrate derivative binding; calcium-transporting ATPase activity; ATPase activity, coupled to transmembrane movement of substances; ATPase activity, coupled; metal ion binding; calcium ion transmembrane transporter activity; purine ribonucleotide binding; purine nucleoside binding; cation transmembrane transporter activity; transmembrane transporter activity; small molecule binding;	K05853	ATP2A; Ca2+ transporting ATPase, sarcoplasmic/endoplasmic reticulum [EC:3.6.3.8]	dre04020 Calcium signaling pathway - Danio rerio (zebrafish);	IPR023298; IPR004014; IPR023299; IPR008250; IPR006068; IPR023214;	P-type ATPase, transmembrane domain; Cation-transporting P-type ATPase, N-terminal; P-type ATPase, cytoplasmic domain N; P-type ATPase, A domain; Cation-transporting P-type ATPase, C-terminal; HAD-like domain;
B0S758	ttna	Uncharacterized protein	_EK(cr)ELESGRPEGEIVGR_	7294	1	IEFLKKEKELESGRP	.......K.L.....	cytosol,nuclear							IPR003598; IPR007110; IPR013783; IPR003599; IPR013098;	Immunoglobulin subtype 2; Immunoglobulin-like domain; Immunoglobulin-like fold; Immunoglobulin subtype; Immunoglobulin I-set;
B8A5D3	nars	Uncharacterized protein	_EK(cr)NLEEAK_	101	1	EDAERREKNLEEAKK	.......K.L.....	cytosol	cellular aromatic compound metabolic process; cellular protein metabolic process; single-organism process; biosynthetic process; asparaginyl-tRNA aminoacylation; tRNA aminoacylation; cellular metabolic process; organic substance metabolic process; metabolic process; cellular amino acid metabolic process; heterocycle metabolic process; nucleobase-containing compound metabolic process; cellular nitrogen compound metabolic process; protein metabolic process; macromolecule metabolic process; tRNA aminoacylation for protein translation; macromolecule biosynthetic process; translation; cellular process; small molecule metabolic process; single-organism cellular process; nitrogen compound metabolic process; organic cyclic compound metabolic process; primary metabolic process; organic substance biosynthetic process; cellular biosynthetic process; RNA metabolic process; tRNA metabolic process; cellular macromolecule biosynthetic process; nucleic acid metabolic process; single-organism metabolic process; amino acid activation; cellular macromolecule metabolic process; carboxylic acid metabolic process; gene expression; organic acid metabolic process; ncRNA metabolic process; oxoacid metabolic process; organonitrogen compound	intracellular part; cell; cell part; intracellular; cytoplasm;	ligase activity, forming aminoacyl-tRNA and related compounds; ATP binding; nucleoside phosphate binding; ligase activity, forming carbon-oxygen bonds; nucleoside binding; ion binding; ligase activity; nucleotide binding; organic cyclic compound binding; heterocyclic compound binding; ribonucleoside binding; carbohydrate derivative binding; ribonucleotide binding; catalytic activity; binding; nucleic acid binding; purine ribonucleoside binding; purine ribonucleotide binding; anion binding; adenyl ribonucleotide binding; adenyl nucleotide binding; purine nucleoside binding; asparagine-tRNA ligase activity; aminoacyl-tRNA ligase activity; purine nucleotide binding; small molecule binding; purine ribonucleoside triphosphate binding;	K01893	NARS; asparaginyl-tRNA synthetase [EC:6.1.1.22]	dre00970 Aminoacyl-tRNA biosynthesis - Danio rerio (zebrafish);	IPR012340; IPR004364; IPR004365; IPR006195;	Nucleic acid-binding, OB-fold; Aminoacyl-tRNA synthetase, class II (D/K/N); OB-fold nucleic acid binding domain, AA-tRNA synthetase-type; Aminoacyl-tRNA synthetase, class II;
E7EZ16	-	Uncharacterized protein	_EK(cr)YIDQEELNK_	259	1	KKKKIKEKYIDQEEL	.......K.I.....	cytosol	protein folding; metabolic process; response to stress; cellular protein metabolic process; cellular macromolecule metabolic process; macromolecule metabolic process; protein metabolic process; response to stimulus; primary metabolic process; cellular process; cellular metabolic process; organic substance metabolic process;		ATP binding; nucleoside phosphate binding; nucleoside binding; ion binding; nucleotide binding; organic cyclic compound binding; heterocyclic compound binding; ribonucleoside binding; carbohydrate derivative binding; ribonucleotide binding; binding; purine ribonucleoside binding; anion binding; purine ribonucleotide binding; adenyl ribonucleotide binding; adenyl nucleotide binding; purine nucleoside binding; purine nucleotide binding; small molecule binding; purine ribonucleoside triphosphate binding;	K04079	htpG; molecular chaperone HtpG	dre04914 Progesterone-mediated oocyte maturation - Danio rerio (zebrafish); dre04141 Protein processing in endoplasmic reticulum - Danio rerio (zebrafish); dre04621 NOD-like receptor signaling pathway - Danio rerio (zebrafish);	IPR020568; IPR020575; IPR003594;	Ribosomal protein S5 domain 2-type fold; Heat shock protein Hsp90, N-terminal; Histidine kinase-like ATPase, C-terminal domain;
B8A568	myhz1.1	Uncharacterized protein	_ELATMK(cr)EDFVK_	855	1	EKELATMKEDFVKCK	..E....K.......	nuclear		intracellular organelle; intracellular non-membrane-bounded organelle; cytoskeletal part; intracellular organelle part; macromolecular complex; intracellular part; cell; actin cytoskeleton; organelle part; non-membrane-bounded organelle; organelle; cytoskeleton; cell part; intracellular; myosin complex; protein complex;	ATP binding; motor activity; nucleoside phosphate binding; nucleoside binding; ion binding; hydrolase activity, acting on acid anhydrides; hydrolase activity; nucleotide binding; nucleoside-triphosphatase activity; organic cyclic compound binding; heterocyclic compound binding; ribonucleoside binding; carbohydrate derivative binding; pyrophosphatase activity; catalytic activity; ribonucleotide binding; binding; purine ribonucleoside binding; anion binding; purine ribonucleotide binding; adenyl ribonucleotide binding; adenyl nucleotide binding; purine nucleoside binding; hydrolase activity, acting on acid anhydrides, in phosphorus-containing anhydrides; purine nucleotide binding; small molecule binding; purine ribonucleoside triphosphate binding;	K10352	MYH; myosin heavy chain	dre04530 Tight junction - Danio rerio (zebrafish);	IPR002928; IPR004009; IPR001609; IPR027417; IPR027401;	Myosin tail; Myosin, N-terminal, SH3-like; Myosin head, motor domain; P-loop containing nucleoside triphosphate hydrolase; Myosin-like IQ motif-containing domain;

B8A561	myhz1.2	Uncharacterized protein	_ELATMK(cr)EDFVK_	855	1	EKELATMKEDFVKCK	..E....K.......	nuclear		intracellular organelle; intracellular non-membrane-bounded organelle; cytoskeletal part; intracellular organelle part; macromolecular complex; intracellular part; cell; actin cytoskeleton; organelle part; non-membrane-bounded organelle; organelle; cytoskeleton; cell part; intracellular; myosin complex; protein complex;	ATP binding; motor activity; nucleoside phosphate binding; nucleoside binding; ion binding; hydrolase activity, acting on acid anhydrides; hydrolase activity; nucleotide binding; nucleoside-triphosphatase activity; organic cyclic compound binding; heterocyclic compound binding; ribonucleoside binding; carbohydrate derivative binding; pyrophosphatase activity; catalytic activity; ribonucleotide binding; binding; purine ribonucleoside binding; anion binding; purine ribonucleotide binding; adenyl ribonucleotide binding; adenyl nucleotide binding; purine nucleoside binding; hydrolase activity, acting on acid anhydrides, in phosphorus-containing anhydrides; purine nucleotide binding; small molecule binding; purine ribonucleoside triphosphate binding;	K10352	MYH; myosin heavy chain	dre04530 Tight junction - Danio rerio (zebrafish);	IPR002928; IPR004009; IPR001609; IPR027417; IPR027401;	Myosin tail; Myosin, N-terminal, SH3-like; Myosin head, motor domain; P-loop containing nucleoside triphosphate hydrolase; Myosin-like IQ motif-containing domain;
B8A568	myhz1.1	Uncharacterized protein	_ELEEK(cr)MVALLQEK_	878	1	KKKELEEKMVALLQE	.......K.V.....	nuclear		intracellular organelle; intracellular non-membrane-bounded organelle; cytoskeletal part; intracellular organelle part; macromolecular complex; intracellular part; cell; actin cytoskeleton; organelle part; non-membrane-bounded organelle; organelle; cytoskeleton; cell part; intracellular; myosin complex; protein complex;	ATP binding; motor activity; nucleoside phosphate binding; nucleoside binding; ion binding; hydrolase activity, acting on acid anhydrides; hydrolase activity; nucleotide binding; nucleoside-triphosphatase activity; organic cyclic compound binding; heterocyclic compound binding; ribonucleoside binding; carbohydrate derivative binding; pyrophosphatase activity; catalytic activity; ribonucleotide binding; binding; purine ribonucleoside binding; anion binding; purine ribonucleotide binding; adenyl ribonucleotide binding; adenyl nucleotide binding; purine nucleoside binding; hydrolase activity, acting on acid anhydrides, in phosphorus-containing anhydrides; purine nucleotide binding; small molecule binding; purine ribonucleoside triphosphate binding;	K10352	MYH; myosin heavy chain	dre04530 Tight junction - Danio rerio (zebrafish);	IPR002928; IPR004009; IPR001609; IPR027417; IPR027401;	Myosin tail; Myosin, N-terminal, SH3-like; Myosin head, motor domain; P-loop containing nucleoside triphosphate hydrolase; Myosin-like IQ motif-containing domain;
B8A561	myhz1.2	Uncharacterized protein	_ELEEK(cr)MVALLQEK_	878	1	KKKELEEKMVALLQE	.......K.V.....	nuclear		intracellular organelle; intracellular non-membrane-bounded organelle; cytoskeletal part; intracellular organelle part; macromolecular complex; intracellular part; cell; actin cytoskeleton; organelle part; non-membrane-bounded organelle; organelle; cytoskeleton; cell part; intracellular; myosin complex; protein complex;	ATP binding; motor activity; nucleoside phosphate binding; nucleoside binding; ion binding; hydrolase activity, acting on acid anhydrides; hydrolase activity; nucleotide binding; nucleoside-triphosphatase activity; organic cyclic compound binding; heterocyclic compound binding; ribonucleoside binding; carbohydrate derivative binding; pyrophosphatase activity; catalytic activity; ribonucleotide binding; binding; purine ribonucleoside binding; anion binding; purine ribonucleotide binding; adenyl ribonucleotide binding; adenyl nucleotide binding; purine nucleoside binding; hydrolase activity, acting on acid anhydrides, in phosphorus-containing anhydrides; purine nucleotide binding; small molecule binding; purine ribonucleoside triphosphate binding;	K10352	MYH; myosin heavy chain	dre04530 Tight junction - Danio rerio (zebrafish);	IPR002928; IPR004009; IPR001609; IPR027417; IPR027401;	Myosin tail; Myosin, N-terminal, SH3-like; Myosin head, motor domain; P-loop containing nucleoside triphosphate hydrolase; Myosin-like IQ motif-containing domain;
E7FAD0	myhz1.1	Uncharacterized protein	_ELEEK(cr)MVSLLQEK_	878	1	KKKELEEKMVSLLQE	.......K.V.....	cytosol		intracellular organelle; intracellular non-membrane-bounded organelle; cytoskeletal part; intracellular organelle part; macromolecular complex; intracellular part; cell; actin cytoskeleton; organelle part; non-membrane-bounded organelle; organelle; cytoskeleton; cell part; intracellular; myosin complex; protein complex;	ATP binding; motor activity; nucleoside phosphate binding; nucleoside binding; ion binding; hydrolase activity, acting on acid anhydrides; hydrolase activity; nucleotide binding; nucleoside-triphosphatase activity; organic cyclic compound binding; heterocyclic compound binding; ribonucleoside binding; carbohydrate derivative binding; pyrophosphatase activity; catalytic activity; ribonucleotide binding; binding; purine ribonucleoside binding; anion binding; purine ribonucleotide binding; adenyl ribonucleotide binding; adenyl nucleotide binding; purine nucleoside binding; hydrolase activity, acting on acid anhydrides, in phosphorus-containing anhydrides; purine nucleotide binding; small molecule binding; purine ribonucleoside triphosphate binding;	K10352	MYH; myosin heavy chain	dre04530 Tight junction - Danio rerio (zebrafish);	IPR002928; IPR004009; IPR001609; IPR027417; IPR027401;	Myosin tail; Myosin, N-terminal, SH3-like; Myosin head, motor domain; P-loop containing nucleoside triphosphate hydrolase; Myosin-like IQ motif-containing domain;
Q6DHP2	tnni2b.2	Troponin I, skeletal, fast 2b.2	_ELHQK(cr)IDVVDEER_	67	1	LCKELHQKIDVVDEE		mitochondria								
Q6NUW5	anp32e	Acidic leucine-rich nuclear phosphoprotein 32 family member E	_ELSTLEALQNLK(cr)NLK_	113	1	LEALQNLKNLKSLDL	.......K.L.....	nuclear		organelle; intracellular membrane-bounded organelle; intracellular organelle; nucleus; cell part; intracellular; cytoplasm; intracellular part; membrane-bounded organelle; cell;					IPR003603;	U2A'/phosphoprotein 32 family A, C-terminal;
E7FES0	-	Uncharacterized protein	_ELTELGEQVK(cr)PHFEGIFK_	240	1	TELGEQVKPHFEGIF		extracellular	lipid transport; single-organism process; lipid localization; organic substance transport; primary metabolic process; lipoprotein metabolic process; organic substance metabolic process; establishment of localization; transport; metabolic process; single-organism localization; protein metabolic process; macromolecule metabolic process; localization; macromolecule localization; single-organism transport;	extracellular region;	lipid binding; binding;					
B8A568	myhz1.1	Uncharacterized protein	_ELTYQTEEDK(cr)K_	1860	1	TYQTEEDKKNVNRLQ	......DK.......	nuclear		intracellular organelle; intracellular non-membrane-bounded organelle; cytoskeletal part; intracellular organelle part; macromolecular complex; intracellular part; cell; actin cytoskeleton; organelle part; non-membrane-bounded organelle; organelle; cytoskeleton; cell part; intracellular; myosin complex; protein complex;	ATP binding; motor activity; nucleoside phosphate binding; nucleoside binding; ion binding; hydrolase activity, acting on acid anhydrides; hydrolase activity; nucleotide binding; nucleoside-triphosphatase activity; organic cyclic compound binding; heterocyclic compound binding; ribonucleoside binding; carbohydrate derivative binding; pyrophosphatase activity; catalytic activity; ribonucleotide binding; binding; purine ribonucleoside binding; anion binding; purine ribonucleotide binding; adenyl ribonucleotide binding; adenyl nucleotide binding; purine nucleoside binding; hydrolase activity, acting on acid anhydrides, in phosphorus-containing anhydrides; purine nucleotide binding; small molecule binding; purine ribonucleoside triphosphate binding;	K10352	MYH; myosin heavy chain	dre04530 Tight junction - Danio rerio (zebrafish);	IPR002928; IPR004009; IPR001609; IPR027417; IPR027401;	Myosin tail; Myosin, N-terminal, SH3-like; Myosin head, motor domain; P-loop containing nucleoside triphosphate hydrolase; Myosin-like IQ motif-containing domain;
B8A561	myhz1.2	Uncharacterized protein	_ELTYQTEEDK(cr)K_	1860	1	TYQTEEDKKNVNRLQ	......DK.......	nuclear		intracellular organelle; intracellular non-membrane-bounded organelle; cytoskeletal part; intracellular organelle part; macromolecular complex; intracellular part; cell; actin cytoskeleton; organelle part; non-membrane-bounded organelle; organelle; cytoskeleton; cell part; intracellular; myosin complex; protein complex;	ATP binding; motor activity; nucleoside phosphate binding; nucleoside binding; ion binding; hydrolase activity, acting on acid anhydrides; hydrolase activity; nucleotide binding; nucleoside-triphosphatase activity; organic cyclic compound binding; heterocyclic compound binding; ribonucleoside binding; carbohydrate derivative binding; pyrophosphatase activity; catalytic activity; ribonucleotide binding; binding; purine ribonucleoside binding; anion binding; purine ribonucleotide binding; adenyl ribonucleotide binding; adenyl nucleotide binding; purine nucleoside binding; hydrolase activity, acting on acid anhydrides, in phosphorus-containing anhydrides; purine nucleotide binding; small molecule binding; purine ribonucleoside triphosphate binding;	K10352	MYH; myosin heavy chain	dre04530 Tight junction - Danio rerio (zebrafish);	IPR002928; IPR004009; IPR001609; IPR027417; IPR027401;	Myosin tail; Myosin, N-terminal, SH3-like; Myosin head, motor domain; P-loop containing nucleoside triphosphate hydrolase; Myosin-like IQ motif-containing domain;

B8A568	myhz1.1	Uncharacterized protein	_ELTYQTEEDKK(cr)NVNR_	1861	1	YQTEEDKKNVNRLQD	.......K.V.....	nuclear		intracellular organelle; intracellular non-membrane-bounded organelle; cytoskeletal part; intracellular organelle part; macromolecular complex; intracellular part; cell; actin cytoskeleton; organelle part; non-membrane-bounded organelle; organelle; cytoskeleton; cell part; intracellular; myosin complex; protein complex;	ATP binding; motor activity; nucleoside phosphate binding; nucleoside binding; ion binding; hydrolase activity, acting on acid anhydrides; hydrolase activity; nucleotide binding; nucleoside-triphosphatase activity; organic cyclic compound binding; heterocyclic compound binding; ribonucleoside binding; carbohydrate derivative binding; pyrophosphatase activity; catalytic activity; ribonucleotide binding; binding; purine ribonucleoside binding; anion binding; purine ribonucleotide binding; adenyl ribonucleotide binding; adenyl nucleotide binding; purine nucleoside binding; hydrolase activity, acting on acid anhydrides, in phosphorus-containing anhydrides; purine nucleotide binding; small molecule binding; purine ribonucleoside triphosphate binding;	K10352	MYH; myosin heavy chain	dre04530 Tight junction - Danio rerio (zebrafish);	IPR002928; IPR004009; IPR001609; IPR027417; IPR027401;	Myosin tail; Myosin, N-terminal, SH3-like; Myosin head, motor domain; P-loop containing nucleoside triphosphate hydrolase; Myosin-like IQ motif-containing domain;
B8A561	myhz1.2	Uncharacterized protein	_ELTYQTEEDKK(cr)NVNR_	1861	1	YQTEEDKKNVNRLQD	.......K.V.....	nuclear		intracellular organelle; intracellular non-membrane-bounded organelle; cytoskeletal part; intracellular organelle part; macromolecular complex; intracellular part; cell; actin cytoskeleton; organelle part; non-membrane-bounded organelle; organelle; cytoskeleton; cell part; intracellular; myosin complex; protein complex;	ATP binding; motor activity; nucleoside phosphate binding; nucleoside binding; ion binding; hydrolase activity, acting on acid anhydrides; hydrolase activity; nucleotide binding; nucleoside-triphosphatase activity; organic cyclic compound binding; heterocyclic compound binding; ribonucleoside binding; carbohydrate derivative binding; pyrophosphatase activity; catalytic activity; ribonucleotide binding; binding; purine ribonucleoside binding; anion binding; purine ribonucleotide binding; adenyl ribonucleotide binding; adenyl nucleotide binding; purine nucleoside binding; hydrolase activity, acting on acid anhydrides, in phosphorus-containing anhydrides; purine nucleotide binding; small molecule binding; purine ribonucleoside triphosphate binding;	K10352	MYH; myosin heavy chain	dre04530 Tight junction - Danio rerio (zebrafish);	IPR002928; IPR004009; IPR001609; IPR027417; IPR027401;	Myosin tail; Myosin, N-terminal, SH3-like; Myosin head, motor domain; P-loop containing nucleoside triphosphate hydrolase; Myosin-like IQ motif-containing domain;
B3DFP9	apoa2	Uncharacterized protein	_ELVDK(cr)YDGLR_	32	1	PDKELVDKYDGLRTV	......DK.......	extracellular	developmental process; cellular component organization; single-multicellular organism process; morphogenesis of an epithelium; epithelium development; epiboly involved in gastrulation with mouth forming second; tissue development; organelle fission; epiboly; morphogenesis of an epithelial sheet; single-organism process; multicellular organismal process; gastrulation; anatomical structure morphogenesis; gastrulation with mouth forming second; tissue morphogenesis; organelle organization; single-organism developmental process; nuclear division; multicellular organismal development; embryo development; cellular process; anatomical structure development; embryonic morphogenesis; cellular component organization or biogenesis;							
E7EZ16	-	Uncharacterized protein	_EMLQQSK(cr)ILK_	383	1	REMLQQSKILKVIRK	.......K.L.....	cytosol	protein folding; metabolic process; response to stress; cellular protein metabolic process; cellular macromolecule metabolic process; macromolecule metabolic process; protein metabolic process; response to stimulus; primary metabolic process; cellular process; cellular metabolic process; organic substance metabolic process;		ATP binding; nucleoside phosphate binding; nucleoside binding; ion binding; nucleotide binding; organic cyclic compound binding; heterocyclic compound binding; ribonucleoside binding; carbohydrate derivative binding; ribonucleotide binding; binding; purine ribonucleoside binding; anion binding; purine ribonucleotide binding; adenyl ribonucleotide binding; adenyl nucleotide binding; purine nucleoside binding; purine nucleotide binding; small molecule binding; purine ribonucleoside triphosphate binding;	K04079	htpG; molecular chaperone HtpG	dre04914 Progesterone-mediated oocyte maturation - Danio rerio (zebrafish); dre04141 Protein processing in endoplasmic reticulum - Danio rerio (zebrafish); dre04621 NOD-like receptor signaling pathway - Danio rerio (zebrafish);	IPR020568; IPR020575; IPR003594;	Ribosomal protein S5 domain 2-type fold; Heat shock protein Hsp90, N-terminal; Histidine kinase-like ATPase, C-terminal domain;
Q6NYR4	hspa8	Heat shock cognate 71 kDa protein	_ENK(cr)ITITNDK_	500	1	KSTGKENKITITNDK		cytosol	response to stress; response to stimulus;		ATP binding; nucleoside phosphate binding; nucleoside binding; ion binding; nucleotide binding; organic cyclic compound binding; heterocyclic compound binding; ribonucleoside binding; carbohydrate derivative binding; ribonucleotide binding; binding; purine ribonucleoside binding; anion binding; purine ribonucleotide binding; adenyl ribonucleotide binding; adenyl nucleotide binding; purine nucleoside binding; purine nucleotide binding; small molecule binding; purine ribonucleoside triphosphate binding;	K03283	HSPA1_8; heat shock 70kDa protein 1/8	dre04010 MAPK signaling pathway - Danio rerio (zebrafish); dre04141 Protein processing in endoplasmic reticulum - Danio rerio (zebrafish); dre04144 Endocytosis - Danio rerio (zebrafish); dre03040 Spliceosome - Danio rerio (zebrafish);	IPR029048; IPR029047;	Heat shock protein 70kD, C-terminal domain; Heat shock protein 70kD, peptide-binding domain;
B8A561	myhz1.2	Uncharacterized protein	_ENLGK(cr)LM(ox)TNLR_	659	1	QFRENLGKLMTNLRS		nuclear		intracellular organelle; intracellular non-membrane-bounded organelle; cytoskeletal part; intracellular organelle part; macromolecular complex; intracellular part; cell; actin cytoskeleton; organelle part; non-membrane-bounded organelle; organelle; cytoskeleton; cell part; intracellular; myosin complex; protein complex;	ATP binding; motor activity; nucleoside phosphate binding; nucleoside binding; ion binding; hydrolase activity, acting on acid anhydrides; hydrolase activity; nucleotide binding; nucleoside-triphosphatase activity; organic cyclic compound binding; heterocyclic compound binding; ribonucleoside binding; carbohydrate derivative binding; pyrophosphatase activity; catalytic activity; ribonucleotide binding; binding; purine ribonucleoside binding; anion binding; purine ribonucleotide binding; adenyl ribonucleotide binding; adenyl nucleotide binding; purine nucleoside binding; hydrolase activity, acting on acid anhydrides, in phosphorus-containing anhydrides; purine nucleotide binding; small molecule binding; purine ribonucleoside triphosphate binding;	K10352	MYH; myosin heavy chain	dre04530 Tight junction - Danio rerio (zebrafish);	IPR002928; IPR004009; IPR001609; IPR027417; IPR027401;	Myosin tail; Myosin, N-terminal, SH3-like; Myosin head, motor domain; P-loop containing nucleoside triphosphate hydrolase; Myosin-like IQ motif-containing domain;
B8A568	myhz1.1	Uncharacterized protein	_ENLGK(cr)LMTNLR_	659	1	QFRENLGKLMTNLRS		nuclear		intracellular organelle; intracellular non-membrane-bounded organelle; cytoskeletal part; intracellular organelle part; macromolecular complex; intracellular part; cell; actin cytoskeleton; organelle part; non-membrane-bounded organelle; organelle; cytoskeleton; cell part; intracellular; myosin complex; protein complex;	ATP binding; motor activity; nucleoside phosphate binding; nucleoside binding; ion binding; hydrolase activity, acting on acid anhydrides; hydrolase activity; nucleotide binding; nucleoside-triphosphatase activity; organic cyclic compound binding; heterocyclic compound binding; ribonucleoside binding; carbohydrate derivative binding; pyrophosphatase activity; catalytic activity; ribonucleotide binding; binding; purine ribonucleoside binding; anion binding; purine ribonucleotide binding; adenyl ribonucleotide binding; adenyl nucleotide binding; purine nucleoside binding; hydrolase activity, acting on acid anhydrides, in phosphorus-containing anhydrides; purine nucleotide binding; small molecule binding; purine ribonucleoside triphosphate binding;	K10352	MYH; myosin heavy chain	dre04530 Tight junction - Danio rerio (zebrafish);	IPR002928; IPR004009; IPR001609; IPR027417; IPR027401;	Myosin tail; Myosin, N-terminal, SH3-like; Myosin head, motor domain; P-loop containing nucleoside triphosphate hydrolase; Myosin-like IQ motif-containing domain;

B8A561	myhz1.2	Uncharacterized protein	_ENLGK(cr)LMTNLR_	659	1	QFRENLGKLMTNLRS		nuclear		intracellular organelle; intracellular non-membrane-bounded organelle; cytoskeletal part; intracellular organelle part; macromolecular complex; intracellular part; cell; actin cytoskeleton; organelle part; non-membrane-bounded organelle; organelle; cytoskeleton; cell part; intracellular; myosin complex; protein complex;	ATP binding; motor activity; nucleoside phosphate binding; nucleoside binding; ion binding; hydrolase activity, acting on acid anhydrides; hydrolase activity; nucleotide binding; nucleoside-triphosphatase activity; organic cyclic compound binding; heterocyclic compound binding; ribonucleoside binding; carbohydrate derivative binding; pyrophosphatase activity; catalytic activity; ribonucleotide binding; binding; purine ribonucleoside binding; anion binding; purine ribonucleotide binding; adenyl ribonucleotide binding; adenyl nucleotide binding; purine nucleoside binding; hydrolase activity, acting on acid anhydrides, in phosphorus-containing anhydrides; purine nucleotide binding; small molecule binding; purine ribonucleoside triphosphate binding;	K10352	MYH; myosin heavy chain	dre04530 Tight junction - Danio rerio (zebrafish);	IPR002928; IPR004009; IPR001609; IPR027417; IPR027401;	Myosin tail; Myosin, N-terminal, SH3-like; Myosin head, motor domain; P-loop containing nucleoside triphosphate hydrolase; Myosin-like IQ motif-containing domain;
O42363	apoa1	Apolipoprotein A-I	_EQLVK(cr)AVEEAR_	218	1	EYKEQLVKAVEEARE	.......K.V.....	extracellular	cholesterol metabolic process; lipid transport; organic hydroxy compound metabolic process; alcohol metabolic process; small molecule metabolic process; steroid metabolic process; organic cyclic compound metabolic process; single-organism process; organic substance transport; lipid localization; primary metabolic process; sterol metabolic process; lipoprotein metabolic process; establishment of localization; organic substance metabolic process; transport; metabolic process; single-organism localization; single-organism metabolic process; macromolecule metabolic process; protein metabolic process; localization; macromolecule localization; single-organism transport; lipid metabolic process;	macromolecular complex; extracellular region part; extracellular space; high-density lipoprotein particle; protein-lipid complex; extracellular region; plasma lipoprotein particle;	lipid binding; binding;	K08757	APOA1; apolipoprotein A-I	dre03320 PPAR signaling pathway - Danio rerio (zebrafish);		
Q804W0	pvalb1	Parvalbumin 1	_ETK(cr)AFLSAGDSDGDGK_	84	1	VLTDKETKAFLSAGD		cytosol			cation binding; metal ion binding; calcium ion binding; binding; ion binding;				IPR011992; IPR002048;	EF-hand domain pair; EF-hand domain;
Q7T3L3	hsp90b1	Chaperone protein GP96	_ETLQQHK(cr)LLK_	455	1	RETLQQHKLLKVIRK	.......K.L.....	endoplasmic reticulum	developmental process; single-multicellular organism process; response to stress; system development; cellular protein metabolic process; single-organism process; response to stimulus; primary metabolic process; otolith development; inner ear development; cellular metabolic process; multicellular organismal process; organic substance metabolic process; otolith tethering; protein folding; metabolic process; organ development; ear development; single-organism developmental process; cellular macromolecule metabolic process; protein metabolic process; macromolecule metabolic process; multicellular organismal development; sensory organ development; cellular process; anatomical structure development;		ATP binding; nucleoside phosphate binding; nucleoside binding; ion binding; nucleotide binding; organic cyclic compound binding; heterocyclic compound binding; ribonucleoside binding; carbohydrate derivative binding; ribonucleotide binding; binding; purine ribonucleoside binding; anion binding; purine ribonucleotide binding; adenyl ribonucleotide binding; adenyl nucleotide binding; purine nucleoside binding; purine nucleotide binding; small molecule binding; purine ribonucleoside triphosphate binding;	K09487	HSP90B; heat shock protein 90kDa beta	dre04141 Protein processing in endoplasmic reticulum - Danio rerio (zebrafish); dre04621 NOD-like receptor signaling pathway - Danio rerio (zebrafish);	IPR020568; IPR020575; IPR003594;	Ribosomal protein S5 domain 2-type fold; Heat shock protein Hsp90, N-terminal; Histidine kinase-like ATPase, C-terminal domain;
H0WE86	mdh1aa	Uncharacterized protein	_EVIPTDK(cr)VEVGFK_	90	1	REVIPTDKVEVGFKD	......DK.......	extracellular	dicarboxylic acid metabolic process; metabolic process; single-organism metabolic process; malate metabolic process; small molecule metabolic process; single-organism cellular process; carboxylic acid metabolic process; single-organism process; organic acid metabolic process; cellular process; cellular metabolic process; oxoacid metabolic process; organic substance metabolic process;		organic cyclic compound binding; heterocyclic compound binding; oxidoreductase activity, acting on CH-OH group of donors; nucleoside phosphate binding; catalytic activity; binding; nucleotide binding; malate dehydrogenase activity; small molecule binding; oxidoreductase activity;				IPR016040; IPR001236;	NAD(P)-binding domain; Lactate/malate dehydrogenase, N-terminal;
Q5TZ29	apobb.1	Uncharacterized protein	_EVLMK(cr)VIFDK_	518	1	EGREVLMKVIFDKAA	.......K.I.....	endoplasmic reticulum	response to chemical; response to stimulus;		lipid transporter activity; substrate-specific transporter activity; transporter activity;				IPR011030; IPR015817; IPR015818; IPR001747; IPR015819; IPR015816; IPR016024; IPR015255; IPR009454;	Vitellinogen, superhelical; Vitellinogen, open beta-sheet, subdomain 1; Vitellinogen, open beta-sheet, subdomain 2; Lipid transport protein, N-terminal; Lipid transport protein, beta-sheet shell; Vitellinogen, beta-sheet N-terminal; Armadillo-type fold; Vitellinogen, open beta-sheet; Lipid transport, open beta-sheet;
B3DFN3	lmnb2	Lamin B2	_EVSGIK(cr)SLYEAELADAR_	73	1	TREVSGIKSLYEAEL	.......K.L.....	nuclear		intracellular organelle; intracellular non-membrane-bounded organelle; cytoskeletal part; intracellular organelle part; macromolecular complex; intracellular part; cell; organelle part; non-membrane-bounded organelle; organelle; cytoskeleton; intermediate filament cytoskeleton; cell part; intracellular; intermediate filament; protein complex;	structural molecule activity;	K07611			IPR001322;	Lamin Tail Domain;
Q6P0G6	myl1	Uncharacterized protein	_EVTK(cr)ILGNPTADDMVNKR_	89	1	PTNKEVTKILGNPTA	.......K.L.....	cytosol			cation binding; metal ion binding; calcium ion binding; binding; ion binding;	K05738			IPR002048; IPR011992;	EF-hand domain; EF-hand domain pair;

Q5U3A4	atp2a1	Calcium-transporting ATPase	_EYEPEM(ox)GK(cr)VYR_	128	1	EYEPEMGKVYRSDRK	..E....K.......	plasma membrane	regulation of biological quality; nucleoside metabolic process; purine nucleoside metabolic process; cellular divalent inorganic cation homeostasis; purine ribonucleoside monophosphate metabolic process; nucleotide metabolic process; positive regulation of muscle contraction; negative regulation of multicellular organismal process; single-organism process; purine ribonucleoside biosynthetic process; nucleoside biosynthetic process; biosynthetic process; ribonucleoside monophosphate biosynthetic process; cellular metabolic process; phosphate-containing compound metabolic process; response to external stimulus; ribose phosphate biosynthetic process; metabolic process; carbohydrate derivative biosynthetic process; positive regulation of multicellular organismal process; nucleoside phosphate metabolic process; regulation of skeletal muscle contraction; metal ion homeostasis; regulation of system process; ribose phosphate metabolic process; purine nucleoside triphosphate biosynthetic process; heterocycle biosynthetic process; purine ribonucleoside triphosphate biosynthetic process; ion homeostasis; calcium ion homeostasis; small molecule metabolic process; negative regulation of striated muscle contraction; inorganic ion	membrane; endoplasmic reticulum; nuclear outer membrane-endoplasmic reticulum membrane network; organelle membrane; myofibril; cytoplasm; intrinsic component of membrane; intracellular part; sarcoplasm; cell; I band; organelle part; non-membrane-bounded organelle; intracellular; cell part; membrane part; contractile fiber; membrane region; perinuclear region of cytoplasm; membrane-bounded organelle; endoplasmic reticulum membrane; intracellular organelle; bounding membrane of organelle; intracellular non-membrane-bounded organelle; H zone; intracellular organelle part; sarcoplasmic reticulum; endomembrane system; integral component of membrane; organelle; intracellular membrane-bounded organelle; endoplasmic reticulum part; endoplasmic reticulum-Golgi intermediate compartment; A band; sarcomere; contractile fiber part; cytoplasmic part;	ATPase activity, coupled to transmembrane movement of ions; nucleoside phosphate binding; primary active transmembrane transporter activity; substrate-specific transporter activity; hydrolase activity, acting on acid anhydrides; hydrolase activity; substrate-specific transmembrane transporter activity; ATPase activity; organic cyclic compound binding; heterocyclic compound binding; metal ion transmembrane transporter activity; pyrophosphatase activity; cation-transporting ATPase activity; catalytic activity; ribonucleotide binding; ATPase activity, coupled to movement of substances; binding; purine ribonucleoside binding; cation binding; hydrolase activity, acting on acid anhydrides, catalyzing transmembrane movement of substances; anion binding; adenyl ribonucleotide binding; adenyl nucleotide binding; inorganic cation transmembrane transporter activity; hydrolase activity, acting on acid anhydrides, in phosphorus-containing anhydrides; transporter activity; purine nucleotide binding; purine ribonucleoside triphosphate binding; active transmembrane transporter activity; ATP binding; ion transmembrane transporter activity; ion binding; nucleoside binding; nucleotide binding; nucleoside-triphosphatase activity; ribonucleoside binding; P-P-bond-hydrolysis-driven transmembrane transporter activity; ATPase activity, coupled to transmembrane movement of ions, phosphorylative mechanism; divalent inorganic cation transmembrane transporter activity; carbohydrate derivative binding; calcium-transporting ATPase activity; ATPase activity, coupled to transmembrane movement of substances; ATPase activity, coupled; calcium ion transmembrane transporter activity; metal ion binding; purine ribonucleotide binding; purine nucleoside binding; calcium ion binding; cation transmembrane transporter activity; transmembrane transporter activity; small molecule binding;	K05853	ATP2A; Ca2+ transporting ATPase, sarcoplasmic/endoplasmic reticulum [EC:3.6.3.8]	dre04020 Calcium signaling pathway - Danio rerio (zebrafish);	IPR004014; IPR023298; IPR008250; IPR023299; IPR006068; IPR023214;	Cation-transporting P-type ATPase, N-terminal; P-type ATPase, transmembrane domain; P-type ATPase, A domain; P-type ATPase, cytoplasmic domain N; Cation-transporting P-type ATPase, C-terminal; HAD-like domain;
Q642Z0	atp2a1	Calcium-transporting ATPase	_EYEPEMGK(cr)VYR_	128	1	EYEPEMGKVYRSDRK	..E....K.......	plasma membrane	nucleoside metabolic process; purine nucleoside metabolic process; purine ribonucleoside monophosphate metabolic process; nucleotide metabolic process; single-organism process; purine ribonucleoside biosynthetic process; nucleoside biosynthetic process; biosynthetic process; ribonucleoside monophosphate biosynthetic process; cellular metabolic process; phosphate-containing compound metabolic process; ribose phosphate biosynthetic process; metabolic process; carbohydrate derivative biosynthetic process; nucleoside phosphate metabolic process; ribose phosphate metabolic process; purine nucleoside triphosphate biosynthetic process; heterocycle biosynthetic process; purine ribonucleoside triphosphate biosynthetic process; small molecule metabolic process; ATP biosynthetic process; single-organism cellular process; purine ribonucleoside triphosphate metabolic process; organic cyclic compound metabolic process; purine-containing compound metabolic process; ribonucleoside triphosphate metabolic process; purine ribonucleoside monophosphate biosynthetic process; purine nucleoside biosynthetic process; organophosphate biosynthetic process; ribonucleoside monophosphate	intrinsic component of membrane; membrane; integral component of membrane; membrane part;	ATPase activity, coupled to transmembrane movement of ions; nucleoside phosphate binding; primary active transmembrane transporter activity; substrate-specific transporter activity; hydrolase activity, acting on acid anhydrides; hydrolase activity; substrate-specific transmembrane transporter activity; ATPase activity; organic cyclic compound binding; heterocyclic compound binding; metal ion transmembrane transporter activity; pyrophosphatase activity; cation-transporting ATPase activity; catalytic activity; ribonucleotide binding; ATPase activity, coupled to movement of substances; binding; purine ribonucleoside binding; cation binding; hydrolase activity, acting on acid anhydrides, catalyzing transmembrane movement of substances; anion binding; adenyl ribonucleotide binding; adenyl nucleotide binding; inorganic cation transmembrane transporter activity; hydrolase activity, acting on acid anhydrides, in phosphorus-containing anhydrides; transporter activity; purine nucleotide binding; purine ribonucleoside triphosphate binding; active transmembrane transporter activity; ATP binding; ion transmembrane transporter activity; ion binding; nucleoside binding; nucleotide binding; nucleoside-triphosphatase activity; ribonucleoside binding; P-P-bond-hydrolysis-driven transmembrane transporter activity; ATPase activity, coupled to transmembrane movement of ions, phosphorylative mechanism; divalent inorganic cation transmembrane transporter activity; carbohydrate derivative binding; calcium-transporting ATPase activity; ATPase activity, coupled to transmembrane movement of substances; ATPase activity, coupled; metal ion binding; calcium ion transmembrane transporter activity; purine ribonucleotide binding; purine nucleoside binding; cation transmembrane transporter activity; transmembrane transporter activity; small molecule binding;	K05853	ATP2A; Ca2+ transporting ATPase, sarcoplasmic/endoplasmic reticulum [EC:3.6.3.8]	dre04020 Calcium signaling pathway - Danio rerio (zebrafish);	IPR023298; IPR004014; IPR023299; IPR008250; IPR006068; IPR023214;	P-type ATPase, transmembrane domain; Cation-transporting P-type ATPase, N-terminal; P-type ATPase, cytoplasmic domain N; P-type ATPase, A domain; Cation-transporting P-type ATPase, C-terminal; HAD-like domain;
F1QUR3	-	Protein disulfide-isomerase (Fragment)	_FAHTNNEDLLK(cr)K_	149	1	TNNEDLLKKHGIDGE		cytosol	regulation of cellular process; regulation of biological quality; small molecule metabolic process; cell redox homeostasis; single-organism cellular process; ether metabolic process; cellular homeostasis; single-organism process; organic substance metabolic process; homeostatic process; metabolic process; regulation of biological process; single-organism metabolic process; glycerol ether metabolic process; cellular process; biological regulation;	organelle; intracellular membrane-bounded organelle; intracellular organelle; endoplasmic reticulum; cell part; intracellular; cytoplasm; endomembrane system; intracellular part; membrane-bounded organelle; cell; cytoplasmic part;	protein disulfide oxidoreductase activity; oxidoreductase activity, acting on a sulfur group of donors; disulfide oxidoreductase activity; electron carrier activity; isomerase activity; catalytic activity; oxidoreductase activity;	K08056	PDIA3; protein disulfide isomerase family A, member 3 [EC:5.3.4.1]	dre04141 Protein processing in endoplasmic reticulum - Danio rerio (zebrafish);	IPR005788; IPR013766; IPR012336;	Disulphide isomerase; Thioredoxin domain; Thioredoxin-like fold;
F1QEW2	si:dkey-183i3.5	Uncharacterized protein (Fragment)	_FASFIDK(cr)AR_	191	1	HNAVLRAKISMFTNP		nuclear		intracellular organelle; intracellular non-membrane-bounded organelle; cytoskeletal part; intracellular organelle part; macromolecular complex; intracellular part; cell; organelle part; non-membrane-bounded organelle; organelle; cytoskeleton; intermediate filament cytoskeleton; cell part; intracellular; intermediate filament; protein complex;	structural molecule activity;					

Q7T3L3	hsp90b1	Chaperone protein GP96	_FDESDK(cr)AK_	603	1	VKFDESDKAKEKREA	......DK.......	endoplasmic reticulum	developmental process; single-multicellular organism process; response to stress; system development; cellular protein metabolic process; single-organism process; response to stimulus; primary metabolic process; otolith development; inner ear development; cellular metabolic process; multicellular organismal process; organic substance metabolic process; otolith tethering; protein folding; metabolic process; organ development; ear development; single-organism developmental process; cellular macromolecule metabolic process; protein metabolic process; macromolecule metabolic process; multicellular organismal development; sensory organ development; cellular process; anatomical structure development;		ATP binding; nucleoside phosphate binding; nucleoside binding; ion binding; nucleotide binding; organic cyclic compound binding; heterocyclic compound binding; ribonucleoside binding; carbohydrate derivative binding; ribonucleotide binding; binding; purine ribonucleoside binding; anion binding; purine ribonucleotide binding; adenyl ribonucleotide binding; adenyl nucleotide binding; purine nucleoside binding; purine nucleotide binding; small molecule binding; purine ribonucleoside triphosphate binding;	K09487	HSP90B; heat shock protein 90kDa beta	dre04141 Protein processing in endoplasmic reticulum - Danio rerio (zebrafish); dre04621 NOD-like receptor signaling pathway - Danio rerio (zebrafish);	IPR020568; IPR020575; IPR003594;	Ribosomal protein S5 domain 2-type fold; Heat shock protein Hsp90, N-terminal; Histidine kinase-like ATPase, C-terminal domain;
E7FEZ9	atox1	Copper chaperone Atox1	_FDIDLPNK(cr)K_	37	1	FDIDLPNKKVFIESD	.......K.V.....	cytosol	transport; single-organism localization; cation transport; metal ion transport; ion transport; single-organism process; localization; single-organism transport; establishment of localization;		metal ion binding; cation binding; binding; ion binding;	K07213			IPR006121;	Heavy metal-associated domain, HMA;
Q6PC53	ppiab	Peptidyl-prolyl cis-trans isomerase	_FEDENFTLK(cr)HGGK_	91	1	EDENFTLKHGGKGTL	..E....K.......	cytosol	cellular protein metabolic process; protein peptidyl-prolyl isomerization; peptidyl-proline modification; primary metabolic process; cellular metabolic process; organic substance metabolic process; protein folding; peptidyl-amino acid modification; metabolic process; cellular macromolecule metabolic process; cellular protein modification process; protein metabolic process; macromolecule metabolic process; macromolecule modification; protein modification process; cellular process;		peptidyl-prolyl cis-trans isomerase activity; isomerase activity; catalytic activity; cis-trans isomerase activity;	K03767			IPR002130; IPR029000;	Cyclophilin-type peptidyl-prolyl cis-trans isomerase domain; Cyclophilin-like domain;
Q92005	eef1a	Elongation factor 1-alpha	_FEEITK(cr)EVSAYIK_	172	1	ARFEEITKEVSAYIK	.......K.V.....	cytosol		intracellular part; cell; cell part; intracellular; cytoplasm;	nucleoside phosphate binding; translation elongation factor activity; hydrolase activity, acting on acid anhydrides; hydrolase activity; organic cyclic compound binding; heterocyclic compound binding; pyrophosphatase activity; catalytic activity; ribonucleotide binding; binding; GTPase activity; purine ribonucleoside binding; anion binding; hydrolase activity, acting on acid anhydrides, in phosphorus-containing anhydrides; guanyl nucleotide binding; GTP binding; purine nucleotide binding; purine ribonucleoside triphosphate binding; RNA binding; ion binding; nucleoside binding; nucleotide binding; nucleoside-triphosphatase activity; ribonucleoside binding; translation factor activity, nucleic acid binding; carbohydrate derivative binding; nucleic acid binding; purine ribonucleotide binding; guanyl ribonucleotide binding; purine nucleoside binding; small molecule binding;	K03231	EEF1A; elongation factor 1-alpha	dre03013 RNA transport - Danio rerio (zebrafish);	IPR004160; IPR004161; IPR009001; IPR027417; IPR000795; IPR009000;	Translation elongation factor EFTu/EF1A, C-terminal; Translation elongation factor EFTu/EF1A, domain 2; Translation elongation factor EF1A/initiation factor IF2gamma, C-terminal; P-loop containing nucleoside triphosphate hydrolase; Elongation factor, GTP-binding domain; Translation protein, beta-barrel domain;
Q92005	eef1a	Elongation factor 1-alpha	_FEK(cr)EAAEMGK_	44	1	RTIEKFEKEAAEMGK		cytosol		intracellular part; cell; cell part; intracellular; cytoplasm;	nucleoside phosphate binding; translation elongation factor activity; hydrolase activity, acting on acid anhydrides; hydrolase activity; organic cyclic compound binding; heterocyclic compound binding; pyrophosphatase activity; catalytic activity; ribonucleotide binding; binding; GTPase activity; purine ribonucleoside binding; anion binding; hydrolase activity, acting on acid anhydrides, in phosphorus-containing anhydrides; guanyl nucleotide binding; GTP binding; purine nucleotide binding; purine ribonucleoside triphosphate binding; RNA binding; ion binding; nucleoside binding; nucleotide binding; nucleoside-triphosphatase activity; ribonucleoside binding; translation factor activity, nucleic acid binding; carbohydrate derivative binding; nucleic acid binding; purine ribonucleotide binding; guanyl ribonucleotide binding; purine nucleoside binding; small molecule binding;	K03231	EEF1A; elongation factor 1-alpha	dre03013 RNA transport - Danio rerio (zebrafish);	IPR004160; IPR004161; IPR009001; IPR027417; IPR000795; IPR009000;	Translation elongation factor EFTu/EF1A, C-terminal; Translation elongation factor EFTu/EF1A, domain 2; Translation elongation factor EF1A/initiation factor IF2gamma, C-terminal; P-loop containing nucleoside triphosphate hydrolase; Elongation factor, GTP-binding domain; Translation protein, beta-barrel domain;
B8A568	myhz1.1	Uncharacterized protein	_FGHTK(cr)VFFK_	764	1	EYRFGHTKVFFKAGL		nuclear		intracellular organelle; intracellular non-membrane-bounded organelle; cytoskeletal part; intracellular organelle part; macromolecular complex; intracellular part; cell; actin cytoskeleton; organelle part; non-membrane-bounded organelle; organelle; cytoskeleton; cell part; intracellular; myosin complex; protein complex;	ATP binding; motor activity; nucleoside phosphate binding; nucleoside binding; ion binding; hydrolase activity, acting on acid anhydrides; hydrolase activity; nucleotide binding; nucleoside-triphosphatase activity; organic cyclic compound binding; heterocyclic compound binding; ribonucleoside binding; carbohydrate derivative binding; pyrophosphatase activity; catalytic activity; ribonucleotide binding; binding; purine ribonucleoside binding; anion binding; purine ribonucleotide binding; adenyl ribonucleotide binding; adenyl nucleotide binding; purine nucleoside binding; hydrolase activity, acting on acid anhydrides, in phosphorus-containing anhydrides; purine nucleotide binding; small molecule binding; purine ribonucleoside triphosphate binding;	K10352	MYH; myosin heavy chain	dre04530 Tight junction - Danio rerio (zebrafish);	IPR002928; IPR004009; IPR001609; IPR027417; IPR027401;	Myosin tail; Myosin, N-terminal, SH3-like; Myosin head, motor domain; P-loop containing nucleoside triphosphate hydrolase; Myosin-like IQ motif-containing domain;

B8A561	myhz1.2	Uncharacterized protein	_FGHTK(cr)VFFK_	764	1	EYRFGHTKVFFKAGL		nuclear		intracellular organelle; intracellular non-membrane-bounded organelle; cytoskeletal part; intracellular organelle part; macromolecular complex; intracellular part; cell; actin cytoskeleton; organelle part; non-membrane-bounded organelle; organelle; cytoskeleton; cell part; intracellular; myosin complex; protein complex;	ATP binding; motor activity; nucleoside phosphate binding; nucleoside binding; ion binding; hydrolase activity, acting on acid anhydrides; hydrolase activity; nucleotide binding; nucleoside-triphosphatase activity; organic cyclic compound binding; heterocyclic compound binding; ribonucleoside binding; carbohydrate derivative binding; pyrophosphatase activity; catalytic activity; ribonucleotide binding; binding; purine ribonucleoside binding; anion binding; purine ribonucleotide binding; adenyl ribonucleotide binding; adenyl nucleotide binding; purine nucleoside binding; hydrolase activity, acting on acid anhydrides, in phosphorus-containing anhydrides; purine nucleotide binding; small molecule binding; purine ribonucleoside triphosphate binding;	K10352	MYH; myosin heavy chain	dre04530 Tight junction - Danio rerio (zebrafish);	IPR002928; IPR004009; IPR001609; IPR027417; IPR027401;	Myosin tail; Myosin, N-terminal, SH3-like; Myosin head, motor domain; P-loop containing nucleoside triphosphate hydrolase; Myosin-like IQ motif-containing domain;
E9QFX0	apoc1	Uncharacterized protein	_FGTQMK(cr)EIAEDLADK_	38	1	TKFGTQMKEIAEDLA	.......K.I.....	extracellular	metabolic process; macromolecule metabolic process; protein metabolic process; primary metabolic process; lipoprotein metabolic process; organic substance metabolic process;	extracellular region;						
E7F4R9	-	Isocitrate dehydrogenase [NADP]	_FK(cr)DIFQDIFEK_	259	1	KAYDGRFKDIFQDIF	.......K.I.....	mitochondria	tricarboxylic acid metabolic process; oxidation-reduction process; small molecule metabolic process; tricarboxylic acid cycle; single-organism cellular process; single-organism process; isocitrate metabolic process; primary metabolic process; cellular respiration; cellular metabolic process; organic substance metabolic process; citrate metabolic process; metabolic process; generation of precursor metabolites and energy; aerobic respiration; single-organism metabolic process; energy derivation by oxidation of organic compounds; carboxylic acid metabolic process; organic acid metabolic process; cellular process; oxoacid metabolic process;		magnesium ion binding; nucleoside phosphate binding; ion binding; isocitrate dehydrogenase activity; nucleotide binding; cofactor binding; oxidoreductase activity; oxidoreductase activity, acting on CH-OH group of donors; heterocyclic compound binding; organic cyclic compound binding; oxidoreductase activity, acting on the CH-OH group of donors, NAD or NADP as acceptor; catalytic activity; NAD binding; binding; isocitrate dehydrogenase (NADP+) activity; metal ion binding; cation binding; small molecule binding; coenzyme binding;	K00031	IDH1; isocitrate dehydrogenase [EC:1.1.1.42]	dre01210 2-Oxocarboxylic acid metabolism - Danio rerio (zebrafish); dre01230 Biosynthesis of amino acids - Danio rerio (zebrafish); dre01200 Carbon metabolism - Danio rerio (zebrafish); dre01100 Metabolic pathways - Danio rerio (zebrafish); dre00480 Glutathione metabolism - Danio rerio (zebrafish); dre04146 Peroxisome - Danio rerio (zebrafish); dre00020 Citrate cycle (TCA cycle) - Danio rerio (zebrafish);	IPR024084;	Isopropylmalate dehydrogenase-like domain;
D1GJ56	actn3a	Actn3a	_FLDIPK(cr)MLDADDIVNTPKPDEK_	229	1	EKFLDIPKMLDADDI	.......K.L.....	cytosol	cellular component biogenesis; cellular component organization; actin crosslink formation; protein complex subunit organization; single-organism cellular process; actin filament organization; single-organism process; actin filament bundle assembly; actin filament bundle organization; macromolecular complex subunit organization; single-organism organelle organization; actin cytoskeleton organization; cellular component assembly; organelle organization; actin filament-based process; cytoskeleton organization; cellular process; cellular component organization or biogenesis;		cation binding; metal ion binding; calcium ion binding; binding; ion binding;	K05699	ACTN; actinin alpha	dre04510 Focal adhesion - Danio rerio (zebrafish); dre04530 Tight junction - Danio rerio (zebrafish); dre04810 Regulation of actin cytoskeleton - Danio rerio (zebrafish); dre04520 Adherens junction - Danio rerio (zebrafish);	IPR014837; IPR011992; IPR002048; IPR001715;	EF-hand, Ca insensitive; EF-hand domain pair; EF-hand domain; Calponin homology domain;
Q503V2	apoea	Uncharacterized protein	_FNEDLQLLVTK(cr)LR_	107	1	DLQLLVTKLRTHMEE		extracellular	lipid transport; single-organism process; lipid localization; organic substance transport; primary metabolic process; lipoprotein metabolic process; organic substance metabolic process; establishment of localization; transport; metabolic process; single-organism localization; protein metabolic process; macromolecule metabolic process; localization; macromolecule localization; single-organism transport;	extracellular region;	lipid binding; binding;	K04524				
Q08BA1	atp5a1	ATP synthase subunit alpha	_FNEGTEEK(cr)K_	238	1	FNEGTEEKKKLYCIY	..E....K.......	mitochondria	nucleoside metabolic process; purine nucleoside metabolic process; purine ribonucleoside monophosphate metabolic process; nucleotide metabolic process; energy coupled proton transmembrane transport, against electrochemical gradient; single-organism process; biosynthetic process; nucleoside biosynthetic process; purine ribonucleoside biosynthetic process; ribonucleoside monophosphate biosynthetic process; cellular metabolic process; phosphate-containing compound metabolic process; establishment of localization; ribose phosphate biosynthetic process; metabolic process; carbohydrate derivative biosynthetic process; nucleoside phosphate metabolic process; ribose phosphate metabolic process; purine nucleoside triphosphate biosynthetic process; single-organism transport; heterocycle biosynthetic process; purine ribonucleoside triphosphate biosynthetic process; cation transmembrane transport; small molecule metabolic process; ATP biosynthetic process; single-organism cellular process; purine-containing compound metabolic process; organic cyclic compound metabolic process; purine ribonucleoside triphosphate metabolic process; energy coupled proton transport, down electrochemical	proton-transporting ATP synthase complex, catalytic core F(1); membrane; membrane part; cell part; intracellular; proton-transporting two-sector ATPase complex; macromolecular complex; proton-transporting two-sector ATPase complex, catalytic domain; intracellular part; protein complex; cell; proton-transporting ATP synthase complex;	ATPase activity, coupled to transmembrane movement of ions; nucleoside phosphate binding; primary active transmembrane transporter activity; substrate-specific transporter activity; hydrolase activity, acting on acid anhydrides; ATPase activity, coupled to transmembrane movement of ions, rotational mechanism; monovalent inorganic cation transmembrane transporter activity; hydrolase activity; hydrogen ion transmembrane transporter activity; substrate-specific transmembrane transporter activity; proton-transporting ATP synthase activity, rotational mechanism; ATPase activity; organic cyclic compound binding; heterocyclic compound binding; cation-transporting ATPase activity; pyrophosphatase activity; catalytic activity; ribonucleotide binding; ATPase activity, coupled to movement of substances; binding; purine ribonucleoside binding; hydrogen-exporting ATPase activity; hydrolase activity, acting on acid anhydrides, catalyzing transmembrane movement of substances; anion binding; adenyl ribonucleotide binding; adenyl nucleotide binding; inorganic cation transmembrane transporter activity; hydrolase activity, acting on acid anhydrides, in phosphorus-containing anhydrides; transporter activity; purine nucleotide binding; purine ribonucleoside triphosphate binding; active transmembrane transporter activity; ATP binding; ion transmembrane transporter activity; ion binding; nucleoside binding; nucleotide binding; nucleoside-triphosphatase activity; proton-transporting ATPase activity, rotational mechanism; ribonucleoside binding; P-P-bond-hydrolysis-driven transmembrane transporter activity; carbohydrate derivative binding; ATPase activity, coupled to transmembrane movement of substances; ATPase activity, coupled; purine ribonucleotide binding; purine nucleoside binding; cation transmembrane transporter activity; transmembrane transporter activity; small molecule binding;	K02132	ATPeF1A; F-type H+-transporting ATPase subunit alpha	dre01100 Metabolic pathways - Danio rerio (zebrafish); dre00190 Oxidative phosphorylation - Danio rerio (zebrafish);	IPR000793; IPR004100; IPR000194; IPR027417; IPR023366;	ATPase, F1/V1/A1 complex, alpha/beta subunit, C-terminal; ATPase, F1 complex alpha/beta subunit, N-terminal domain; ATPase, F1/V1/A1 complex, alpha/beta subunit, nucleotide-binding domain; P-loop containing nucleoside triphosphate hydrolase; ATP synthase subunit alpha-like domain;
A2CE73	khdrbs1a	Uncharacterized protein	_FNFVGK(cr)ILGPQGSTIK_	88	1	PRFNFVGKILGPQGS	.......K.L.....	cytosol			organic cyclic compound binding; heterocyclic compound binding; RNA binding; binding; nucleic acid binding;				IPR004087; IPR004088;	K Homology domain; K Homology domain, type 1;

Q1LYB7	rpl13a	OTTDARP00000009674	_FNK(cr)VLIIDGR_	7	1	MADRFNKVLIIDGRG		cytosol	cellular protein metabolic process; biosynthetic process; primary metabolic process; cellular metabolic process; organic substance biosynthetic process; cellular biosynthetic process; organic substance metabolic process; metabolic process; cellular macromolecule biosynthetic process; cellular macromolecule metabolic process; gene expression; macromolecule metabolic process; protein metabolic process; macromolecule biosynthetic process; translation; cellular process;	intracellular organelle; ribosome; intracellular non-membrane-bounded organelle; cytoplasm; intracellular organelle part; macromolecular complex; intracellular part; cell; organelle part; non-membrane-bounded organelle; organelle; large ribosomal subunit; ribosomal subunit; cell part; intracellular; ribonucleoprotein complex; cytoplasmic part;	structural constituent of ribosome; structural molecule activity;	K02872	RP-L13Ae; large subunit ribosomal protein L13Ae	dre03010 Ribosome - Danio rerio (zebrafish);	IPR023564;	Ribosomal protein L13 domain;
B3DFN3	lmnb2	Lamin B2	_FSPK(cr)FVLK_	486	1	IVYKFSPKFVLKAGQ	.......K.V.....	nuclear		intracellular organelle; intracellular non-membrane-bounded organelle; cytoskeletal part; intracellular organelle part; macromolecular complex; intracellular part; cell; organelle part; non-membrane-bounded organelle; organelle; cytoskeleton; intermediate filament cytoskeleton; cell part; intracellular; intermediate filament; protein complex;	structural molecule activity;	K07611			IPR001322;	Lamin Tail Domain;
O93409	mylpfa	Mylz2 protein	_FTAEEMK(cr)NLWAAFPPDVAGNVDYK_	138	1	RFTAEEMKNLWAAFP	.......K.L.....	cytosol,nuclear			cation binding; metal ion binding; calcium ion binding; binding; ion binding;	K12758	MYLPF; fast skeletal myosin light chain 2	dre04510 Focal adhesion - Danio rerio (zebrafish); dre04530 Tight junction - Danio rerio (zebrafish); dre04810 Regulation of actin cytoskeleton - Danio rerio (zebrafish);	IPR011992; IPR002048;	EF-hand domain pair; EF-hand domain;
Q6TNV0	cox4i1	Cytochrome c oxidase subunit IV isoform 1	_FVQQLSADQK(cr)SLK_	60	1	QQLSADQKSLKEKEK	.......K.L.....	mitochondria	response to oxygen levels; response to decreased oxygen levels; response to stimulus; response to stress; response to abiotic stimulus; response to hypoxia;		ion transmembrane transporter activity; substrate-specific transporter activity; monovalent inorganic cation transmembrane transporter activity; oxidoreductase activity, acting on a heme group of donors, oxygen as acceptor; oxidoreductase activity, acting on a heme group of donors; hydrogen ion transmembrane transporter activity; heme-copper terminal oxidase activity; oxidoreductase activity; substrate-specific transmembrane transporter activity; electron carrier activity; cytochrome-c oxidase activity; catalytic activity; inorganic cation transmembrane transporter activity; cation transmembrane transporter activity; transporter activity; transmembrane transporter activity;	K02263	COX4; cytochrome c oxidase subunit 4	dre04260 Cardiac muscle contraction - Danio rerio (zebrafish); dre01100 Metabolic pathways - Danio rerio (zebrafish); dre00190 Oxidative phosphorylation - Danio rerio (zebrafish);		
F8W246	rps13	Uncharacterized protein	_FVTGNK(cr)ILR_	70	1	VRFVTGNKILRILKS	.......K.L.....	mitochondria	cellular protein metabolic process; biosynthetic process; primary metabolic process; cellular metabolic process; organic substance biosynthetic process; cellular biosynthetic process; organic substance metabolic process; metabolic process; cellular macromolecule biosynthetic process; cellular macromolecule metabolic process; gene expression; macromolecule metabolic process; protein metabolic process; macromolecule biosynthetic process; translation; cellular process;	ribosome; organelle; intracellular organelle; intracellular non-membrane-bounded organelle; cell part; intracellular; cytoplasm; macromolecular complex; intracellular part; cell; ribonucleoprotein complex; cytoplasmic part; non-membrane-bounded organelle;	structural constituent of ribosome; structural molecule activity;				IPR012606; IPR009068;	Ribosomal protein S13/S15, N-terminal; S15/NS1, RNA-binding;
E9QG51	mylpfb	Uncharacterized protein	_GADPEDVIVSAFK(cr)VLDPEATGTIK_	85	1	DVIVSAFKVLDPEAT	.......K.L.....	cytosol			cation binding; metal ion binding; calcium ion binding; binding; ion binding;				IPR011992; IPR002048;	EF-hand domain pair; EF-hand domain;
O93409	mylpfa	Mylz2 protein	_GADPEDVIVSAFK(cr)VLDPEGTGSIK_	106	1	DVIVSAFKVLDPEGT	.......K.L.....	cytosol,nuclear			cation binding; metal ion binding; calcium ion binding; binding; ion binding;	K12758	MYLPF; fast skeletal myosin light chain 2	dre04510 Focal adhesion - Danio rerio (zebrafish); dre04530 Tight junction - Danio rerio (zebrafish); dre04810 Regulation of actin cytoskeleton - Danio rerio (zebrafish);	IPR011992; IPR002048;	EF-hand domain pair; EF-hand domain;
Q6AZA0	acat1	Acetyl-CoA acetyltransferase, mitochondrial	_GAIDK(cr)AGIPVEEVK_	71	1	AIKGAIDKAGIPVEE	......DK.......	mitochondria		organelle; intracellular membrane-bounded organelle; intracellular organelle; cell part; intracellular; cytoplasm; intracellular part; membrane-bounded organelle; cell; mitochondrion; cytoplasmic part;	transferase activity, transferring acyl groups; catalytic activity; binding; ion binding; metal ion binding; cation binding; acetyl-CoA C-acyltransferase activity; C-acyltransferase activity; acetyl-CoA C-acetyltransferase activity; transferase activity; transferase activity, transferring acyl groups other than amino-acyl groups; acetyltransferase activity; C-acetyltransferase activity;	K00626	E2.3.1.9; acetyl-CoA C-acetyltransferase [EC:2.3.1.9]	dre01200 Carbon metabolism - Danio rerio (zebrafish); dre00900 Terpenoid backbone biosynthesis - Danio rerio (zebrafish); dre01212 Fatty acid metabolism - Danio rerio (zebrafish); dre00630 Glyoxylate and dicarboxylate metabolism - Danio rerio (zebrafish); dre00280 Valine, leucine and isoleucine degradation - Danio rerio (zebrafish); dre00640 Propanoate metabolism - Danio rerio (zebrafish); dre01100 Metabolic pathways - Danio rerio (zebrafish); dre00310 Lysine degradation - Danio rerio (zebrafish); dre00620 Pyruvate metabolism - Danio rerio (zebrafish); dre00072 Synthesis and degradation of ketone bodies - Danio rerio (zebrafish); dre00380 Tryptophan metabolism - Danio rerio (zebrafish); dre00071 Fatty acid degradation - Danio rerio (zebrafish); dre00650 Butanoate metabolism - Danio rerio (zebrafish);	IPR016039; IPR020616; IPR020617;	Thiolase-like; Thiolase, N-terminal; Thiolase, C-terminal;
F1Q560	shank3b	Uncharacterized protein	_GANK(cr)EIK_	354	1	LLFRGANKEIKNYNN	.......K.I.....	nuclear				K15009			IPR011511; IPR001452; IPR013761; IPR020683; IPR001660; IPR001478;	Variant SH3 domain; SH3 domain; Sterile alpha motif/pointed domain; Ankyrin repeat-containing domain; Sterile alpha motif domain; PDZ domain;

E9QGA9	gapdh	Glyceraldehyde-3-phosphate dehydrogenase	_GASQNIIPASTGAAK(cr)AVGK_	213	1	PASTGAAKAVGKVIP	.......K.V.....	cytosol			organic cyclic compound binding; heterocyclic compound binding; oxidoreductase activity, acting on the aldehyde or oxo group of donors, NAD or NADP as acceptor; nucleoside phosphate binding; catalytic activity; binding; oxidoreductase activity, acting on the aldehyde or oxo group of donors; nucleotide binding; small molecule binding; oxidoreductase activity; glyceraldehyde-3-phosphate dehydrogenase (NAD+) (phosphorylating) activity;				IPR020829; IPR016040; IPR020828;	Glyceraldehyde 3-phosphate dehydrogenase, catalytic domain; NAD(P)-binding domain; Glyceraldehyde 3-phosphate dehydrogenase, NAD(P) binding domain;
Q6P3L9	glud1b	Glutamate dehydrogenase 1	_GATIVENK(cr)LVEDLK_	68	1	GATIVENKLVEDLKT	.......K.V.....	cytosol	small molecule metabolic process; single-organism cellular process; nitrogen compound metabolic process; single-organism process; primary metabolic process; cellular metabolic process; organic substance metabolic process; metabolic process; cellular amino acid metabolic process; single-organism metabolic process; carboxylic acid metabolic process; organic acid metabolic process; cellular process; oxoacid metabolic process; organonitrogen compound metabolic process;		organic cyclic compound binding; heterocyclic compound binding; nucleoside phosphate binding; catalytic activity; oxidoreductase activity, acting on the CH-NH2 group of donors, NAD or NADP as acceptor; binding; nucleotide binding; oxidoreductase activity, acting on the CH-NH2 group of donors; oxidoreductase activity; small molecule binding;	K00261	GLUD1_2; glutamate dehydrogenase (NAD(P)+) [EC:1.4.1.3]	dre00330 Arginine and proline metabolism - Danio rerio (zebrafish); dre01100 Metabolic pathways - Danio rerio (zebrafish); dre00910 Nitrogen metabolism - Danio rerio (zebrafish); dre00250 Alanine, aspartate and glutamate metabolism - Danio rerio (zebrafish); dre00471 D-Glutamine and D-glutamate metabolism - Danio rerio (zebrafish);	IPR006096; IPR016040; IPR006097;	Glutamate/phenylalanine/leucine/valine dehydrogenase, C-terminal; NAD(P)-binding domain; Glutamate/phenylalanine/leucine/valine dehydrogenase, dimerisation domain;
F6NLI6	ppib	Peptidyl-prolyl cis-trans isomerase	_GDGTGGK(cr)SIYGDR_	108	1	RGDGTGGKSIYGDRF	.......K.I.....	extracellular				K03768			IPR002130; IPR029000;	Cyclophilin-type peptidyl-prolyl cis-trans isomerase domain; Cyclophilin-like domain;
I3ISQ0	lgals2b	Galectin	_GDVK(cr)IISVK_	118	1	VHVKGDVKIISVKAK	.......K.I.....	cytosol			carbohydrate binding; binding;	K06830			IPR001079; IPR013320;	Galectin, carbohydrate recognition domain; Concanavalin A-like lectin/glucanase, subgroup;
Q6NWJ5	tuba8l4	Tubulin alpha 6	_GDVVPK(cr)DVNSAIATIK_	326	1	YRGDVVPKDVNSAIA	.......K.V.....	cytosol	cellular component biogenesis; cellular component organization; cellular macromolecular complex assembly; protein complex subunit organization; single-organism cellular process; single-organism process; microtubule-based process; macromolecular complex subunit organization; microtubule-based movement; cellular component assembly; macromolecular complex assembly; protein polymerization; movement of cell or subcellular component; protein complex biogenesis; cellular process; protein complex assembly; cellular protein complex assembly; cellular component organization or biogenesis;	intracellular organelle; microtubule; intracellular non-membrane-bounded organelle; cytoskeletal part; intracellular organelle part; cytoplasm; macromolecular complex; intracellular part; cell; organelle part; non-membrane-bounded organelle; organelle; cytoskeleton; microtubule cytoskeleton; intracellular; cell part; protein complex;	nucleoside phosphate binding; nucleoside binding; ion binding; hydrolase activity, acting on acid anhydrides; hydrolase activity; nucleotide binding; nucleoside-triphosphatase activity; organic cyclic compound binding; heterocyclic compound binding; pyrophosphatase activity; catalytic activity; ribonucleotide binding; ribonucleoside binding; carbohydrate derivative binding; binding; GTPase activity; purine ribonucleoside binding; anion binding; purine ribonucleotide binding; purine nucleoside binding; guanyl ribonucleotide binding; hydrolase activity, acting on acid anhydrides, in phosphorus-containing anhydrides; guanyl nucleotide binding; structural molecule activity; GTP binding; purine nucleotide binding; small molecule binding; purine ribonucleoside triphosphate binding;	K07374	TUBA; tubulin alpha	dre04145 Phagosome - Danio rerio (zebrafish); dre04540 Gap junction - Danio rerio (zebrafish);	IPR003008; IPR023123; IPR008280; IPR018316;	Tubulin/FtsZ, GTPase domain; Tubulin, C-terminal; Tubulin/FtsZ, C-terminal; Tubulin/FtsZ, 2-layer sandwich domain;
F1QJP3	myl10	Uncharacterized protein	_GEEIK(cr)YHLMSQADK_	119	1	ILKGEEIKYHLMSQA		cytosol,nuclear			cation binding; metal ion binding; calcium ion binding; binding; ion binding;				IPR002048; IPR011992;	EF-hand domain; EF-hand domain pair;
F1QK60	krt4	Uncharacterized protein	_GELAVK(cr)DAK_	370	1	ERGELAVKDAKLRIR		nuclear		intracellular organelle; intracellular non-membrane-bounded organelle; cytoskeletal part; intracellular organelle part; macromolecular complex; intracellular part; cell; keratin filament; organelle part; non-membrane-bounded organelle; organelle; cytoskeleton; intermediate filament cytoskeleton; cell part; intracellular; intermediate filament; protein complex;	structural molecule activity;	K07605				
H0WEH3	khsrp	Uncharacterized protein	_GGETIK(cr)QLQER_	198	1	GKGGETIKQLQERAG	.......K.L.....	cytosol			organic cyclic compound binding; heterocyclic compound binding; RNA binding; binding; nucleic acid binding;				IPR009019; IPR004087; IPR004088;	K homology domain, prokaryotic type; K Homology domain; K Homology domain, type 1;
Q6PC12	eno1a	Enolase 1, (Alpha)	_GGK(cr)YDLDFK_	256	1	SEFYKGGKYDLDFKS		cytosol	single-organism carbohydrate catabolic process; glycolytic process; single-organism catabolic process; small molecule metabolic process; single-organism cellular process; single-organism process; carbohydrate metabolic process; primary metabolic process; cellular metabolic process; organic substance metabolic process; carbohydrate catabolic process; single-organism carbohydrate metabolic process; organic substance catabolic process; metabolic process; pyruvate metabolic process; generation of precursor metabolites and energy; single-organism metabolic process; carboxylic acid metabolic process; monocarboxylic acid metabolic process; organic acid metabolic process; cellular process; catabolic process; oxoacid metabolic process;	catalytic complex; cell part; intracellular; cytoplasm; macromolecular complex; intracellular part; cytosolic part; cell; cytosol; protein complex; phosphopyruvate hydratase complex; cytoplasmic part;	magnesium ion binding; phosphopyruvate hydratase activity; catalytic activity; binding; ion binding; metal ion binding; cation binding; lyase activity; hydro-lyase activity; carbon-oxygen lyase activity;	K01689	ENO; enolase [EC:4.2.1.11]	dre00010 Glycolysis / Gluconeogenesis - Danio rerio (zebrafish); dre01230 Biosynthesis of amino acids - Danio rerio (zebrafish); dre01200 Carbon metabolism - Danio rerio (zebrafish); dre01100 Metabolic pathways - Danio rerio (zebrafish); dre03018 RNA degradation - Danio rerio (zebrafish);	IPR029065; IPR029017; IPR020810; IPR020811;	Enolase C-terminal domain-like; Enolase N-terminal domain-like; Enolase, C-terminal; Enolase, N-terminal;

Q7T306	ckmb	Uncharacterized protein	_GGNMK(cr)EVFK_	246	1	MQKGGNMKEVFKRFC	.......K.V.....	cytosol			ATP binding; nucleoside phosphate binding; nucleoside binding; ion binding; nucleotide binding; organic cyclic compound binding; heterocyclic compound binding; ribonucleoside binding; carbohydrate derivative binding; kinase activity; catalytic activity; ribonucleotide binding; binding; purine ribonucleoside binding; anion binding; purine ribonucleotide binding; adenyl ribonucleotide binding; adenyl nucleotide binding; purine nucleoside binding; transferase activity; transferase activity, transferring phosphorus-containing groups; purine nucleotide binding; small molecule binding; purine ribonucleoside triphosphate binding;				IPR022413; IPR022414; IPR014746;	ATP:guanido phosphotransferase, N-terminal; ATP:guanido phosphotransferase, catalytic domain; Glutamine synthetase/guanido kinase, catalytic domain;
A2BHA3	ckma	Uncharacterized protein	_GGNMK(cr)EVFK_	247	1	MQKGGNMKEVFKRFC	.......K.V.....	cytosol			ATP binding; nucleoside phosphate binding; nucleoside binding; ion binding; nucleotide binding; organic cyclic compound binding; heterocyclic compound binding; ribonucleoside binding; carbohydrate derivative binding; kinase activity; catalytic activity; ribonucleotide binding; binding; purine ribonucleoside binding; anion binding; purine ribonucleotide binding; adenyl ribonucleotide binding; adenyl nucleotide binding; purine nucleoside binding; transferase activity; transferase activity, transferring phosphorus-containing groups; purine nucleotide binding; small molecule binding; purine ribonucleoside triphosphate binding;	K00933	E2.7.3.2; creatine kinase [EC:2.7.3.2]	dre00330 Arginine and proline metabolism - Danio rerio (zebrafish); dre01100 Metabolic pathways - Danio rerio (zebrafish);	IPR022413; IPR022414; IPR014746;	ATP:guanido phosphotransferase, N-terminal; ATP:guanido phosphotransferase, catalytic domain; Glutamine synthetase/guanido kinase, catalytic domain;
Q7T306	ckmb	Uncharacterized protein	_GGVHVK(cr)LPK_	297	1	LRGGVHVKLPKLSTH		cytosol			ATP binding; nucleoside phosphate binding; nucleoside binding; ion binding; nucleotide binding; organic cyclic compound binding; heterocyclic compound binding; ribonucleoside binding; carbohydrate derivative binding; kinase activity; catalytic activity; ribonucleotide binding; binding; purine ribonucleoside binding; anion binding; purine ribonucleotide binding; adenyl ribonucleotide binding; adenyl nucleotide binding; purine nucleoside binding; transferase activity; transferase activity, transferring phosphorus-containing groups; purine nucleotide binding; small molecule binding; purine ribonucleoside triphosphate binding;				IPR022413; IPR022414; IPR014746;	ATP:guanido phosphotransferase, N-terminal; ATP:guanido phosphotransferase, catalytic domain; Glutamine synthetase/guanido kinase, catalytic domain;
A2BHA3	ckma	Uncharacterized protein	_GGVHVK(cr)LPK_	298	1	LRGGVHVKLPKLSTH		cytosol			ATP binding; nucleoside phosphate binding; nucleoside binding; ion binding; nucleotide binding; organic cyclic compound binding; heterocyclic compound binding; ribonucleoside binding; carbohydrate derivative binding; kinase activity; catalytic activity; ribonucleotide binding; binding; purine ribonucleoside binding; anion binding; purine ribonucleotide binding; adenyl ribonucleotide binding; adenyl nucleotide binding; purine nucleoside binding; transferase activity; transferase activity, transferring phosphorus-containing groups; purine nucleotide binding; small molecule binding; purine ribonucleoside triphosphate binding;	K00933	E2.7.3.2; creatine kinase [EC:2.7.3.2]	dre00330 Arginine and proline metabolism - Danio rerio (zebrafish); dre01100 Metabolic pathways - Danio rerio (zebrafish);	IPR022413; IPR022414; IPR014746;	ATP:guanido phosphotransferase, N-terminal; ATP:guanido phosphotransferase, catalytic domain; Glutamine synthetase/guanido kinase, catalytic domain;
Q08BA1	atp5a1	ATP synthase subunit alpha	_GHLDK(cr)MEPSK_	497	1	GVRGHLDKMEPSKIT	......DK.......	mitochondria	nucleoside metabolic process; purine nucleoside metabolic process; purine ribonucleoside monophosphate metabolic process; nucleotide metabolic process; energy coupled proton transmembrane transport, against electrochemical gradient; single-organism process; biosynthetic process; nucleoside biosynthetic process; purine ribonucleoside biosynthetic process; ribonucleoside monophosphate biosynthetic process; cellular metabolic process; phosphate-containing compound metabolic process; establishment of localization; ribose phosphate biosynthetic process; metabolic process; carbohydrate derivative biosynthetic process; nucleoside phosphate metabolic process; ribose phosphate metabolic process; purine nucleoside triphosphate biosynthetic process; single-organism transport; heterocycle biosynthetic process; purine ribonucleoside triphosphate biosynthetic process; cation transmembrane transport; small molecule metabolic process; ATP biosynthetic process; single-organism cellular process; purine-containing compound metabolic process; organic cyclic compound metabolic process; purine ribonucleoside triphosphate metabolic process; energy coupled proton transport, down electrochemical	proton-transporting ATP synthase complex, catalytic core F(1); membrane; membrane part; cell part; intracellular; proton-transporting two-sector ATPase complex; macromolecular complex; proton-transporting two-sector ATPase complex, catalytic domain; intracellular part; protein complex; cell; proton-transporting ATP synthase complex;	ATPase activity, coupled to transmembrane movement of ions; nucleoside phosphate binding; primary active transmembrane transporter activity; substrate-specific transporter activity; hydrolase activity, acting on acid anhydrides; ATPase activity, coupled to transmembrane movement of ions, rotational mechanism; monovalent inorganic cation transmembrane transporter activity; hydrolase activity; hydrogen ion transmembrane transporter activity; substrate-specific transmembrane transporter activity; proton-transporting ATP synthase activity, rotational mechanism; ATPase activity; organic cyclic compound binding; heterocyclic compound binding; cation-transporting ATPase activity; pyrophosphatase activity; catalytic activity; ribonucleotide binding; ATPase activity, coupled to movement of substances; binding; purine ribonucleoside binding; hydrogen-exporting ATPase activity; hydrolase activity, acting on acid anhydrides, catalyzing transmembrane movement of substances; anion binding; adenyl ribonucleotide binding; adenyl nucleotide binding; inorganic cation transmembrane transporter activity; hydrolase activity, acting on acid anhydrides, in phosphorus-containing anhydrides; transporter activity; purine nucleotide binding; purine ribonucleoside triphosphate binding; active transmembrane transporter activity; ATP binding; ion transmembrane transporter activity; ion binding; nucleoside binding; nucleotide binding; nucleoside-triphosphatase activity; proton-transporting ATPase activity, rotational mechanism; ribonucleoside binding; P-P-bond-hydrolysis-driven transmembrane transporter activity; carbohydrate derivative binding; ATPase activity, coupled to transmembrane movement of substances; ATPase activity, coupled; purine ribonucleotide binding; purine nucleoside binding; cation transmembrane transporter activity; transmembrane transporter activity; small molecule binding;	K02132	ATPeF1A; F-type H+-transporting ATPase subunit alpha	dre01100 Metabolic pathways - Danio rerio (zebrafish); dre00190 Oxidative phosphorylation - Danio rerio (zebrafish);	IPR000793; IPR004100; IPR000194; IPR027417; IPR023366;	ATPase, F1/V1/A1 complex, alpha/beta subunit, C-terminal; ATPase, F1 complex alpha/beta subunit, N-terminal domain; ATPase, F1/V1/A1 complex, alpha/beta subunit, nucleotide-binding domain; P-loop containing nucleoside triphosphate hydrolase; ATP synthase subunit alpha-like domain;
Q7ZYX4	cct6a	Chaperonin containing TCP1, subunit 6A (Zeta 1)	_GIDPFSLDALAK(cr)EGIVALR_	307	1	FSLDALAKEGIVALR		cytosol	protein folding; metabolic process; cellular protein metabolic process; cellular macromolecule metabolic process; macromolecule metabolic process; protein metabolic process; primary metabolic process; cellular process; cellular metabolic process; organic substance metabolic process;	intracellular part; cell; cell part; intracellular; cytoplasm;	ATP binding; nucleoside phosphate binding; nucleoside binding; ion binding; nucleotide binding; organic cyclic compound binding; heterocyclic compound binding; ribonucleoside binding; carbohydrate derivative binding; ribonucleotide binding; binding; purine ribonucleoside binding; anion binding; purine ribonucleotide binding; adenyl ribonucleotide binding; adenyl nucleotide binding; purine nucleoside binding; purine nucleotide binding; small molecule binding; purine ribonucleoside triphosphate binding;	K09498			IPR027410; IPR027409; IPR027413;	TCP-1-like chaperonin intermediate domain; GroEL-like apical domain; GroEL-like equatorial domain;
A8KB78	rps23	Uncharacterized protein	_GIVLEK(cr)VGVEAK_	54	1	AKGIVLEKVGVEAKQ		cytosol	cellular protein metabolic process; biosynthetic process; primary metabolic process; cellular metabolic process; organic substance biosynthetic process; cellular biosynthetic process; organic substance metabolic process; metabolic process; cellular macromolecule biosynthetic process; cellular macromolecule metabolic process; gene expression; macromolecule metabolic process; protein metabolic process; macromolecule biosynthetic process; translation; cellular process;	intracellular organelle; ribosome; intracellular non-membrane-bounded organelle; small ribosomal subunit; cytoplasm; intracellular organelle part; macromolecular complex; intracellular part; cell; organelle part; non-membrane-bounded organelle; organelle; ribosomal subunit; cell part; intracellular; ribonucleoprotein complex; cytoplasmic part;	structural constituent of ribosome; structural molecule activity;	K02973	RP-S23e; small subunit ribosomal protein S23e	dre03010 Ribosome - Danio rerio (zebrafish);	IPR012340;	Nucleic acid-binding, OB-fold;

F8W4I2	rplp0	60S acidic ribosomal protein P0	_GK(cr)AVVLMGK_	50	1	IRLSLRGKAVVLMGK	.......K.V.....	cytosol	cellular component biogenesis; ribosome biogenesis; ribonucleoprotein complex biogenesis; cellular component organization or biogenesis;	ribosome; organelle; intracellular organelle; intracellular non-membrane-bounded organelle; cell part; intracellular; cytoplasm; macromolecular complex; intracellular part; cell; ribonucleoprotein complex; cytoplasmic part; non-membrane-bounded organelle;						
Q0D276	zgc:153846	Uncharacterized protein	_GK(cr)VIFYEDR_	3	1	_____MGKVIFYEDR		extracellular							IPR011024; IPR001064;	Gamma-crystallin-related; Beta/gamma crystallin;
Q8AY63	ckbb	Brain-subtype creatine kinase	_GK(cr)YYALK_	172	1	LDGDLKGKYYALKDM		cytosol			ATP binding; nucleoside phosphate binding; creatine kinase activity; nucleoside binding; ion binding; phosphotransferase activity, nitrogenous group as acceptor; nucleotide binding; organic cyclic compound binding; heterocyclic compound binding; ribonucleoside binding; carbohydrate derivative binding; kinase activity; catalytic activity; ribonucleotide binding; binding; purine ribonucleoside binding; anion binding; purine ribonucleotide binding; adenyl ribonucleotide binding; adenyl nucleotide binding; purine nucleoside binding; transferase activity; transferase activity, transferring phosphorus-containing groups; purine nucleotide binding; small molecule binding; purine ribonucleoside triphosphate binding;	K00933	E2.7.3.2; creatine kinase [EC:2.7.3.2]	dre00330 Arginine and proline metabolism - Danio rerio (zebrafish); dre01100 Metabolic pathways - Danio rerio (zebrafish);	IPR022413; IPR014746; IPR022414;	ATP:guanido phosphotransferase, N-terminal; Glutamine synthetase/guanido kinase, catalytic domain; ATP:guanido phosphotransferase, catalytic domain;
Q7T306	ckmb	Uncharacterized protein	_GK(cr)YYPLK_	171	1	LDGEFKGKYYPLKDM		cytosol			ATP binding; nucleoside phosphate binding; nucleoside binding; ion binding; nucleotide binding; organic cyclic compound binding; heterocyclic compound binding; ribonucleoside binding; carbohydrate derivative binding; kinase activity; catalytic activity; ribonucleotide binding; binding; purine ribonucleoside binding; anion binding; purine ribonucleotide binding; adenyl ribonucleotide binding; adenyl nucleotide binding; purine nucleoside binding; transferase activity; transferase activity, transferring phosphorus-containing groups; purine nucleotide binding; small molecule binding; purine ribonucleoside triphosphate binding;				IPR022413; IPR022414; IPR014746;	ATP:guanido phosphotransferase, N-terminal; ATP:guanido phosphotransferase, catalytic domain; Glutamine synthetase/guanido kinase, catalytic domain;
A2BHA3	ckma	Uncharacterized protein	_GK(cr)YYPLK_	172	1	LDGEFKGKYYPLKSM		cytosol			ATP binding; nucleoside phosphate binding; nucleoside binding; ion binding; nucleotide binding; organic cyclic compound binding; heterocyclic compound binding; ribonucleoside binding; carbohydrate derivative binding; kinase activity; catalytic activity; ribonucleotide binding; binding; purine ribonucleoside binding; anion binding; purine ribonucleotide binding; adenyl ribonucleotide binding; adenyl nucleotide binding; purine nucleoside binding; transferase activity; transferase activity, transferring phosphorus-containing groups; purine nucleotide binding; small molecule binding; purine ribonucleoside triphosphate binding;	K00933	E2.7.3.2; creatine kinase [EC:2.7.3.2]	dre00330 Arginine and proline metabolism - Danio rerio (zebrafish); dre01100 Metabolic pathways - Danio rerio (zebrafish);	IPR022413; IPR022414; IPR014746;	ATP:guanido phosphotransferase, N-terminal; ATP:guanido phosphotransferase, catalytic domain; Glutamine synthetase/guanido kinase, catalytic domain;
O93548	bE1	Embryonic 1 beta-globin	_GLELAVK(cr)NM(ox)DNIK_	77	1	KGLELAVKNMDNIKA		cytosol		cell part; intracellular; hemoglobin complex; cytoplasm; macromolecular complex; intracellular part; cytosolic part; protein complex; cell; cytosol; cytoplasmic part;	organic cyclic compound binding; heterocyclic compound binding; heme binding; iron ion binding; transition metal ion binding; oxygen binding; binding; ion binding; substrate-specific transporter activity; metal ion binding; cation binding; oxygen transporter activity; tetrapyrrole binding; transporter activity;				IPR009050; IPR000971; IPR012292;	Globin-like; Globin; Globin, structural domain;
F6NT71	hbbe1.1	Uncharacterized protein	_GLELAVK(cr)NMDNIK_	18	1	KGLELAVKNMDNIKA		cytosol							IPR009050; IPR000971; IPR012292;	Globin-like; Globin; Globin, structural domain;
O93548	bE1	Embryonic 1 beta-globin	_GLELAVK(cr)NMDNIK_	18	1	TIQDIFAKADYDVIG		cytosol		cell part; intracellular; hemoglobin complex; cytoplasm; macromolecular complex; intracellular part; cytosolic part; protein complex; cell; cytosol; cytoplasmic part;	organic cyclic compound binding; heterocyclic compound binding; heme binding; iron ion binding; transition metal ion binding; oxygen binding; binding; ion binding; substrate-specific transporter activity; metal ion binding; cation binding; oxygen transporter activity; tetrapyrrole binding; transporter activity;				IPR009050; IPR000971; IPR012292;	Globin-like; Globin; Globin, structural domain;
Q6PBI5	rps25	40S ribosomal protein S25	_GLIK(cr)LVSK_	97	1	LLGKGLIKLVSKHRA	.......K.V.....	nuclear		ribosome; organelle; intracellular organelle; intracellular non-membrane-bounded organelle; cell part; intracellular; cytoplasm; macromolecular complex; intracellular part; cell; ribonucleoprotein complex; cytoplasmic part; non-membrane-bounded organelle;		K02975	RP-S25e; small subunit ribosomal protein S25e	dre03010 Ribosome - Danio rerio (zebrafish);		
Q4VBU7	cox5aa	Cox5aa protein	_GMNTLIGYDLVPEPK(cr)ILDAALR_	78	1	YDLVPEPKILDAALR	.......K.L.....	mitochondria	nervous system development; developmental process; single-multicellular organism process; cell differentiation in spinal cord; spinal cord development; generation of neurons; embryonic organ development; system development; cellular developmental process; single-organism cellular process; neurogenesis; single-organism process; multicellular organismal process; central nervous system neuron differentiation; neuron differentiation; cell differentiation; organ development; ventral spinal cord development; central nervous system development; single-organism developmental process; multicellular organismal development; spinal cord motor neuron differentiation; embryo development; cellular process; anatomical structure development;	membrane; intracellular organelle; mitochondrial part; organelle membrane; organelle envelope; intracellular organelle part; cytoplasm; intracellular part; cell; organelle part; mitochondrion; mitochondrial envelope; intracellular membrane-bounded organelle; organelle; cell part; intracellular; envelope; organelle inner membrane; membrane-bounded organelle; mitochondrial inner membrane; mitochondrial membrane; cytoplasmic part;	ion transmembrane transporter activity; substrate-specific transporter activity; monovalent inorganic cation transmembrane transporter activity; oxidoreductase activity, acting on a heme group of donors, oxygen as acceptor; oxidoreductase activity, acting on a heme group of donors; hydrogen ion transmembrane transporter activity; heme-copper terminal oxidase activity; oxidoreductase activity; substrate-specific transmembrane transporter activity; electron carrier activity; cytochrome-c oxidase activity; catalytic activity; inorganic cation transmembrane transporter activity; cation transmembrane transporter activity; transporter activity; transmembrane transporter activity;	K02264	COX5A; cytochrome c oxidase subunit 5a	dre04260 Cardiac muscle contraction - Danio rerio (zebrafish); dre01100 Metabolic pathways - Danio rerio (zebrafish); dre00190 Oxidative phosphorylation - Danio rerio (zebrafish);		
E7F225	zgc:163061	Uncharacterized protein	_GMVTK(cr)GVLK_	82	1	AIKGMVTKGVLKQVK	.......K.V.....	nuclear	cellular component biogenesis; cellular component organization; nucleosome organization; cellular macromolecular complex assembly; chromatin assembly or disassembly; protein complex subunit organization; macromolecular complex subunit organization; protein-DNA complex assembly; DNA packaging; nucleosome assembly; chromosome organization; cellular component assembly; macromolecular complex assembly; organelle organization; chromatin organization; protein complex biogenesis; chromatin assembly; cellular process; protein complex assembly; cellular component organization or biogenesis; protein-DNA complex subunit organization; DNA conformation change;	intracellular organelle; intracellular non-membrane-bounded organelle; intracellular organelle part; macromolecular complex; intracellular part; cell; organelle part; DNA bending complex; chromosomal part; nucleosome; non-membrane-bounded organelle; intracellular membrane-bounded organelle; organelle; nucleus; chromatin; cell part; intracellular; chromosome; membrane-bounded organelle; protein complex; protein-DNA complex; DNA packaging complex;	organic cyclic compound binding; heterocyclic compound binding; DNA binding; binding; nucleic acid binding;	K11275			IPR011991; IPR005818;	Winged helix-turn-helix DNA-binding domain; Linker histone H1/H5, domain H15;

B0S564	-	Protein disulfide-isomerase	_GNPK(cr)EYSAGR_	112	1	GGEKGNPKEYSAGRQ	..E....K.......	extracellular	regulation of cellular process; regulation of biological quality; small molecule metabolic process; cell redox homeostasis; single-organism cellular process; ether metabolic process; cellular homeostasis; single-organism process; organic substance metabolic process; homeostatic process; metabolic process; regulation of biological process; single-organism metabolic process; glycerol ether metabolic process; cellular process; biological regulation;	organelle; intracellular membrane-bounded organelle; intracellular organelle; endoplasmic reticulum; cell part; intracellular; cytoplasm; endomembrane system; macromolecular complex; intracellular part; membrane-bounded organelle; cell; protein complex; collagen trimer; cytoplasmic part;	protein disulfide oxidoreductase activity; disulfide oxidoreductase activity; oxidoreductase activity, acting on single donors with incorporation of molecular oxygen, incorporation of two atoms of oxygen; electron carrier activity; oxidoreductase activity, acting on single donors with incorporation of molecular oxygen; isomerase activity; catalytic activity; dioxygenase activity; oxidoreductase activity, acting on a sulfur group of donors; oxidoreductase activity;	K09580	PDIA1; protein disulfide-isomerase A1 [EC:5.3.4.1]	dre04141 Protein processing in endoplasmic reticulum - Danio rerio (zebrafish);	IPR005788; IPR013766; IPR012336;	Disulphide isomerase; Thioredoxin domain; Thioredoxin-like fold;
B8A568	myhz1.1	Uncharacterized protein	_GQEDMK(cr)EQVAMVER_	1670	1	VRGQEDMKEQVAMVE		nuclear		intracellular organelle; intracellular non-membrane-bounded organelle; cytoskeletal part; intracellular organelle part; macromolecular complex; intracellular part; cell; actin cytoskeleton; organelle part; non-membrane-bounded organelle; organelle; cytoskeleton; cell part; intracellular; myosin complex; protein complex;	ATP binding; motor activity; nucleoside phosphate binding; nucleoside binding; ion binding; hydrolase activity, acting on acid anhydrides; hydrolase activity; nucleotide binding; nucleoside-triphosphatase activity; organic cyclic compound binding; heterocyclic compound binding; ribonucleoside binding; carbohydrate derivative binding; pyrophosphatase activity; catalytic activity; ribonucleotide binding; binding; purine ribonucleoside binding; anion binding; purine ribonucleotide binding; adenyl ribonucleotide binding; adenyl nucleotide binding; purine nucleoside binding; hydrolase activity, acting on acid anhydrides, in phosphorus-containing anhydrides; purine nucleotide binding; small molecule binding; purine ribonucleoside triphosphate binding;	K10352	MYH; myosin heavy chain	dre04530 Tight junction - Danio rerio (zebrafish);	IPR002928; IPR004009; IPR001609; IPR027417; IPR027401;	Myosin tail; Myosin, N-terminal, SH3-like; Myosin head, motor domain; P-loop containing nucleoside triphosphate hydrolase; Myosin-like IQ motif-containing domain;
B8A561	myhz1.2	Uncharacterized protein	_GQEDMK(cr)EQVAMVER_	1670	1	VRGQEDMKEQVAMVE		nuclear		intracellular organelle; intracellular non-membrane-bounded organelle; cytoskeletal part; intracellular organelle part; macromolecular complex; intracellular part; cell; actin cytoskeleton; organelle part; non-membrane-bounded organelle; organelle; cytoskeleton; cell part; intracellular; myosin complex; protein complex;	ATP binding; motor activity; nucleoside phosphate binding; nucleoside binding; ion binding; hydrolase activity, acting on acid anhydrides; hydrolase activity; nucleotide binding; nucleoside-triphosphatase activity; organic cyclic compound binding; heterocyclic compound binding; ribonucleoside binding; carbohydrate derivative binding; pyrophosphatase activity; catalytic activity; ribonucleotide binding; binding; purine ribonucleoside binding; anion binding; purine ribonucleotide binding; adenyl ribonucleotide binding; adenyl nucleotide binding; purine nucleoside binding; hydrolase activity, acting on acid anhydrides, in phosphorus-containing anhydrides; purine nucleotide binding; small molecule binding; purine ribonucleoside triphosphate binding;	K10352	MYH; myosin heavy chain	dre04530 Tight junction - Danio rerio (zebrafish);	IPR002928; IPR004009; IPR001609; IPR027417; IPR027401;	Myosin tail; Myosin, N-terminal, SH3-like; Myosin head, motor domain; P-loop containing nucleoside triphosphate hydrolase; Myosin-like IQ motif-containing domain;
A8WGC6	atp5b	ATP synthase subunit beta	_GQK(cr)VLDTGAPIR_	113	1	EGLVRGQKVLDTGAP	.......K.L.....	mitochondria	nucleoside metabolic process; purine nucleoside metabolic process; purine ribonucleoside monophosphate metabolic process; nucleotide metabolic process; energy coupled proton transmembrane transport, against electrochemical gradient; single-organism process; biosynthetic process; nucleoside biosynthetic process; purine ribonucleoside biosynthetic process; ribonucleoside monophosphate biosynthetic process; cellular metabolic process; phosphate-containing compound metabolic process; establishment of localization; ribose phosphate biosynthetic process; metabolic process; carbohydrate derivative biosynthetic process; nucleoside phosphate metabolic process; ribose phosphate metabolic process; purine nucleoside triphosphate biosynthetic process; single-organism transport; heterocycle biosynthetic process; purine ribonucleoside triphosphate biosynthetic process; cation transmembrane transport; small molecule metabolic process; ATP biosynthetic process; single-organism cellular process; purine-containing compound metabolic process; organic cyclic compound metabolic process; purine ribonucleoside triphosphate metabolic process; energy coupled proton transport, down electrochemical	proton-transporting ATP synthase complex, catalytic core F(1); membrane; membrane part; cell part; intracellular; proton-transporting two-sector ATPase complex; macromolecular complex; proton-transporting two-sector ATPase complex, catalytic domain; intracellular part; protein complex; cell; proton-transporting ATP synthase complex;	ATPase activity, coupled to transmembrane movement of ions; nucleoside phosphate binding; primary active transmembrane transporter activity; substrate-specific transporter activity; hydrolase activity, acting on acid anhydrides; ATPase activity, coupled to transmembrane movement of ions, rotational mechanism; monovalent inorganic cation transmembrane transporter activity; hydrolase activity; hydrogen ion transmembrane transporter activity; substrate-specific transmembrane transporter activity; proton-transporting ATP synthase activity, rotational mechanism; ATPase activity; organic cyclic compound binding; heterocyclic compound binding; cation-transporting ATPase activity; pyrophosphatase activity; catalytic activity; ribonucleotide binding; ATPase activity, coupled to movement of substances; binding; purine ribonucleoside binding; hydrogen-exporting ATPase activity; hydrolase activity, acting on acid anhydrides, catalyzing transmembrane movement of substances; anion binding; adenyl ribonucleotide binding; adenyl nucleotide binding; inorganic cation transmembrane transporter activity; hydrolase activity, acting on acid anhydrides, in phosphorus-containing anhydrides; hydrogen-exporting ATPase activity, phosphorylative mechanism; transporter activity; purine nucleotide binding; purine ribonucleoside triphosphate binding; active transmembrane transporter activity; ATP binding; ion transmembrane transporter activity; ion binding; nucleoside binding; nucleotide binding; nucleoside-triphosphatase activity; ribonucleoside binding; ATPase activity, coupled to transmembrane movement of ions, phosphorylative mechanism; P-P-bond-hydrolysis-driven transmembrane transporter activity; carbohydrate derivative binding; ATPase activity, coupled to transmembrane movement of substances; ATPase activity, coupled; purine ribonucleotide binding; purine nucleoside binding; cation transmembrane transporter activity; transmembrane transporter activity; small molecule binding;	K02133	ATPeF1B; F-type H+-transporting ATPase subunit beta [EC:3.6.3.14]	dre01100 Metabolic pathways - Danio rerio (zebrafish); dre00190 Oxidative phosphorylation - Danio rerio (zebrafish);	IPR000793; IPR004100; IPR000194; IPR027417; IPR024034;	ATPase, F1/V1/A1 complex, alpha/beta subunit, C-terminal; ATPase, F1 complex alpha/beta subunit, N-terminal domain; ATPase, F1/V1/A1 complex, alpha/beta subunit, nucleotide-binding domain; P-loop containing nucleoside triphosphate hydrolase; ATPase, F1 complex beta subunit/V1 complex, C-terminal;
B8A568	myhz1.1	Uncharacterized protein	_GQTVPQVYNSVSALSK(cr)SIYER_	433	1	NSVSALSKSIYERMF	.......K.I.....	nuclear		intracellular organelle; intracellular non-membrane-bounded organelle; cytoskeletal part; intracellular organelle part; macromolecular complex; intracellular part; cell; actin cytoskeleton; organelle part; non-membrane-bounded organelle; organelle; cytoskeleton; cell part; intracellular; myosin complex; protein complex;	ATP binding; motor activity; nucleoside phosphate binding; nucleoside binding; ion binding; hydrolase activity, acting on acid anhydrides; hydrolase activity; nucleotide binding; nucleoside-triphosphatase activity; organic cyclic compound binding; heterocyclic compound binding; ribonucleoside binding; carbohydrate derivative binding; pyrophosphatase activity; catalytic activity; ribonucleotide binding; binding; purine ribonucleoside binding; anion binding; purine ribonucleotide binding; adenyl ribonucleotide binding; adenyl nucleotide binding; purine nucleoside binding; hydrolase activity, acting on acid anhydrides, in phosphorus-containing anhydrides; purine nucleotide binding; small molecule binding; purine ribonucleoside triphosphate binding;	K10352	MYH; myosin heavy chain	dre04530 Tight junction - Danio rerio (zebrafish);	IPR002928; IPR004009; IPR001609; IPR027417; IPR027401;	Myosin tail; Myosin, N-terminal, SH3-like; Myosin head, motor domain; P-loop containing nucleoside triphosphate hydrolase; Myosin-like IQ motif-containing domain;

F1RBK3	-	Pyruvate kinase	_GSGTEEVK(cr)LVK_	132	1	GSGTEEVKLVKGNII	.......K.V.....	cytosol	single-organism carbohydrate catabolic process; glycolytic process; single-organism catabolic process; small molecule metabolic process; single-organism cellular process; single-organism process; carbohydrate metabolic process; primary metabolic process; cellular metabolic process; organic substance metabolic process; carbohydrate catabolic process; single-organism carbohydrate metabolic process; organic substance catabolic process; metabolic process; pyruvate metabolic process; generation of precursor metabolites and energy; single-organism metabolic process; carboxylic acid metabolic process; monocarboxylic acid metabolic process; organic acid metabolic process; cellular process; catabolic process; oxoacid metabolic process;		magnesium ion binding; pyruvate kinase activity; kinase activity; phosphotransferase activity, alcohol group as acceptor; catalytic activity; binding; ion binding; potassium ion binding; metal ion binding; cation binding; alkali metal ion binding; transferase activity; transferase activity, transferring phosphorus-containing groups;	K00873	PK; pyruvate kinase [EC:2.7.1.40]	dre00010 Glycolysis / Gluconeogenesis - Danio rerio (zebrafish); dre01230 Biosynthesis of amino acids - Danio rerio (zebrafish); dre01200 Carbon metabolism - Danio rerio (zebrafish); dre01100 Metabolic pathways - Danio rerio (zebrafish); dre00620 Pyruvate metabolism - Danio rerio (zebrafish); dre00230 Purine metabolism - Danio rerio (zebrafish);	IPR015794; IPR011037; IPR015813; IPR015806; IPR015795; IPR015793;	Pyruvate kinase, alpha/beta; Pyruvate kinase-like, insert domain; Pyruvate/Phosphoenolpyruvate kinase-like domain; Pyruvate kinase, beta-barrel insert domain; Pyruvate kinase, C-terminal; Pyruvate kinase, barrel;
Q6NYR4	hspa8	Heat shock cognate 71 kDa protein	_GTLDPVEK(cr)ALR_	319	1	GTLDPVEKALRDAKM	.......K.L.....	cytosol	response to stress; response to stimulus;		ATP binding; nucleoside phosphate binding; nucleoside binding; ion binding; nucleotide binding; organic cyclic compound binding; heterocyclic compound binding; ribonucleoside binding; carbohydrate derivative binding; ribonucleotide binding; binding; purine ribonucleoside binding; anion binding; purine ribonucleotide binding; adenyl ribonucleotide binding; adenyl nucleotide binding; purine nucleoside binding; purine nucleotide binding; small molecule binding; purine ribonucleoside triphosphate binding;	K03283	HSPA1_8; heat shock 70kDa protein 1/8	dre04010 MAPK signaling pathway - Danio rerio (zebrafish); dre04141 Protein processing in endoplasmic reticulum - Danio rerio (zebrafish); dre04144 Endocytosis - Danio rerio (zebrafish); dre03040 Spliceosome - Danio rerio (zebrafish);	IPR029048; IPR029047;	Heat shock protein 70kD, C-terminal domain; Heat shock protein 70kD, peptide-binding domain;
Q90473	hspa8	Heat shock cognate 71 kDa protein	_GTLDPVEK(cr)ALR_	319	1	GTLDPVEKALRDAKM	.......K.L.....	cytosol	developmental process; response to wounding; tissue development; response to stress; wound healing; tissue regeneration; single-organism developmental process; fin regeneration; single-organism process; regeneration; developmental growth; response to stimulus; growth; anatomical structure development;		ATP binding; nucleoside phosphate binding; nucleoside binding; ion binding; nucleotide binding; organic cyclic compound binding; heterocyclic compound binding; ribonucleoside binding; carbohydrate derivative binding; ribonucleotide binding; binding; purine ribonucleoside binding; anion binding; purine ribonucleotide binding; adenyl ribonucleotide binding; adenyl nucleotide binding; purine nucleoside binding; purine nucleotide binding; small molecule binding; purine ribonucleoside triphosphate binding;	K03283	HSPA1_8; heat shock 70kDa protein 1/8	dre04010 MAPK signaling pathway - Danio rerio (zebrafish); dre04141 Protein processing in endoplasmic reticulum - Danio rerio (zebrafish); dre04144 Endocytosis - Danio rerio (zebrafish); dre03040 Spliceosome - Danio rerio (zebrafish);	IPR029048; IPR029047;	Heat shock protein 70kD, C-terminal domain; Heat shock protein 70kD, peptide-binding domain;
E9QBQ4	aldob	Fructose-bisphosphate aldolase	_GVLFPK(cr)VIK_	96	1	DKGVLFPKVIKDKGI	.......K.I.....	cytosol	single-organism carbohydrate catabolic process; glycolytic process; single-organism catabolic process; small molecule metabolic process; single-organism cellular process; single-organism process; carbohydrate metabolic process; primary metabolic process; cellular metabolic process; organic substance metabolic process; carbohydrate catabolic process; single-organism carbohydrate metabolic process; organic substance catabolic process; metabolic process; pyruvate metabolic process; generation of precursor metabolites and energy; single-organism metabolic process; carboxylic acid metabolic process; monocarboxylic acid metabolic process; organic acid metabolic process; cellular process; catabolic process; oxoacid metabolic process;		lyase activity; fructose-bisphosphate aldolase activity; aldehyde-lyase activity; catalytic activity; carbon-carbon lyase activity;				IPR013785;	Aldolase-type TIM barrel;
E7F225	zgc:163061	Uncharacterized protein	_GVLK(cr)QVK_	86	1	MVTKGVLKQVKGTGA	.......K.V.....	nuclear	cellular component biogenesis; cellular component organization; nucleosome organization; cellular macromolecular complex assembly; chromatin assembly or disassembly; protein complex subunit organization; macromolecular complex subunit organization; protein-DNA complex assembly; DNA packaging; nucleosome assembly; chromosome organization; cellular component assembly; macromolecular complex assembly; organelle organization; chromatin organization; protein complex biogenesis; chromatin assembly; cellular process; protein complex assembly; cellular component organization or biogenesis; protein-DNA complex subunit organization; DNA conformation change;	intracellular organelle; intracellular non-membrane-bounded organelle; intracellular organelle part; macromolecular complex; intracellular part; cell; organelle part; DNA bending complex; chromosomal part; nucleosome; non-membrane-bounded organelle; intracellular membrane-bounded organelle; organelle; nucleus; chromatin; cell part; intracellular; chromosome; membrane-bounded organelle; protein complex; protein-DNA complex; DNA packaging complex;	organic cyclic compound binding; heterocyclic compound binding; DNA binding; binding; nucleic acid binding;	K11275			IPR011991; IPR005818;	Winged helix-turn-helix DNA-binding domain; Linker histone H1/H5, domain H15;
E7F7C7	-	Histone H4	_GVLK(cr)VFLENVIR_	60	1	EETRGVLKVFLENVI		nuclear	cellular component biogenesis; cellular component organization; nucleosome organization; cellular macromolecular complex assembly; chromatin assembly or disassembly; protein complex subunit organization; macromolecular complex subunit organization; protein-DNA complex assembly; DNA packaging; nucleosome assembly; chromosome organization; cellular component assembly; macromolecular complex assembly; organelle organization; chromatin organization; protein complex biogenesis; chromatin assembly; cellular process; protein complex assembly; cellular component organization or biogenesis; protein-DNA complex subunit organization; DNA conformation change;	intracellular organelle; intracellular non-membrane-bounded organelle; intracellular organelle part; macromolecular complex; intracellular part; cell; organelle part; DNA bending complex; chromosomal part; nucleosome; non-membrane-bounded organelle; intracellular membrane-bounded organelle; organelle; nucleus; chromatin; cell part; intracellular; chromosome; membrane-bounded organelle; protein complex; protein-DNA complex; DNA packaging complex;	organic cyclic compound binding; heterocyclic compound binding; DNA binding; binding; nucleic acid binding;				IPR009072; IPR007125;	Histone-fold; Histone core;

A8WGC6	atp5b	ATP synthase subunit beta	_GVQK(cr)ILQDYK_	415	1	DVARGVQKILQDYKS	.......K.L.....	mitochondria	nucleoside metabolic process; purine nucleoside metabolic process; purine ribonucleoside monophosphate metabolic process; nucleotide metabolic process; energy coupled proton transmembrane transport, against electrochemical gradient; single-organism process; biosynthetic process; nucleoside biosynthetic process; purine ribonucleoside biosynthetic process; ribonucleoside monophosphate biosynthetic process; cellular metabolic process; phosphate-containing compound metabolic process; establishment of localization; ribose phosphate biosynthetic process; metabolic process; carbohydrate derivative biosynthetic process; nucleoside phosphate metabolic process; ribose phosphate metabolic process; purine nucleoside triphosphate biosynthetic process; single-organism transport; heterocycle biosynthetic process; purine ribonucleoside triphosphate biosynthetic process; cation transmembrane transport; small molecule metabolic process; ATP biosynthetic process; single-organism cellular process; purine-containing compound metabolic process; organic cyclic compound metabolic process; purine ribonucleoside triphosphate metabolic process; energy coupled proton transport, down electrochemical	proton-transporting ATP synthase complex, catalytic core F(1); membrane; membrane part; cell part; intracellular; proton-transporting two-sector ATPase complex; macromolecular complex; proton-transporting two-sector ATPase complex, catalytic domain; intracellular part; protein complex; cell; proton-transporting ATP synthase complex;	ATPase activity, coupled to transmembrane movement of ions; nucleoside phosphate binding; primary active transmembrane transporter activity; substrate-specific transporter activity; hydrolase activity, acting on acid anhydrides; ATPase activity, coupled to transmembrane movement of ions, rotational mechanism; monovalent inorganic cation transmembrane transporter activity; hydrolase activity; hydrogen ion transmembrane transporter activity; substrate-specific transmembrane transporter activity; proton-transporting ATP synthase activity, rotational mechanism; ATPase activity; organic cyclic compound binding; heterocyclic compound binding; cation-transporting ATPase activity; pyrophosphatase activity; catalytic activity; ribonucleotide binding; ATPase activity, coupled to movement of substances; binding; purine ribonucleoside binding; hydrogen-exporting ATPase activity; hydrolase activity, acting on acid anhydrides, catalyzing transmembrane movement of substances; anion binding; adenyl ribonucleotide binding; adenyl nucleotide binding; inorganic cation transmembrane transporter activity; hydrolase activity, acting on acid anhydrides, in phosphorus-containing anhydrides; hydrogen-exporting ATPase activity, phosphorylative mechanism; transporter activity; purine nucleotide binding; purine ribonucleoside triphosphate binding; active transmembrane transporter activity; ATP binding; ion transmembrane transporter activity; ion binding; nucleoside binding; nucleotide binding; nucleoside-triphosphatase activity; ribonucleoside binding; ATPase activity, coupled to transmembrane movement of ions, phosphorylative mechanism; P-P-bond-hydrolysis-driven transmembrane transporter activity; carbohydrate derivative binding; ATPase activity, coupled to transmembrane movement of substances; ATPase activity, coupled; purine ribonucleotide binding; purine nucleoside binding; cation transmembrane transporter activity; transmembrane transporter activity; small molecule binding;	K02133	ATPeF1B; F-type H+-transporting ATPase subunit beta [EC:3.6.3.14]	dre01100 Metabolic pathways - Danio rerio (zebrafish); dre00190 Oxidative phosphorylation - Danio rerio (zebrafish);	IPR000793; IPR004100; IPR000194; IPR027417; IPR024034;	ATPase, F1/V1/A1 complex, alpha/beta subunit, C-terminal; ATPase, F1 complex alpha/beta subunit, N-terminal domain; ATPase, F1/V1/A1 complex, alpha/beta subunit, nucleotide-binding domain; P-loop containing nucleoside triphosphate hydrolase; ATPase, F1 complex beta subunit/V1 complex, C-terminal;
G1K2X0	ttnb	Uncharacterized protein (Fragment)	_GVTISK(cr)VK_	27496	1	QRGVTISKVKIAPFE					ATP binding; nucleoside phosphate binding; phosphotransferase activity, alcohol group as acceptor; nucleoside binding; ion binding; protein tyrosine kinase activity; protein kinase activity; nucleotide binding; organic cyclic compound binding; heterocyclic compound binding; ribonucleoside binding; carbohydrate derivative binding; kinase activity; catalytic activity; ribonucleotide binding; binding; nucleic acid binding; purine ribonucleoside binding; anion binding; purine ribonucleotide binding; adenyl ribonucleotide binding; adenyl nucleotide binding; purine nucleoside binding; transferase activity; transferase activity, transferring phosphorus-containing groups; purine nucleotide binding; small molecule binding; purine ribonucleoside triphosphate binding;	K12567			IPR003598; IPR007110; IPR000719; IPR013783; IPR003599; IPR003961; IPR013098; IPR011009;	Immunoglobulin subtype 2; Immunoglobulin-like domain; Protein kinase domain; Immunoglobulin-like fold; Immunoglobulin subtype; Fibronectin, type III; Immunoglobulin I-set; Protein kinase-like domain;
B8A568	myhz1.1	Uncharacterized protein	_HATENK(cr)VK_	976	1	EKHATENKVKNLTEE		nuclear		intracellular organelle; intracellular non-membrane-bounded organelle; cytoskeletal part; intracellular organelle part; macromolecular complex; intracellular part; cell; actin cytoskeleton; organelle part; non-membrane-bounded organelle; organelle; cytoskeleton; cell part; intracellular; myosin complex; protein complex;	ATP binding; motor activity; nucleoside phosphate binding; nucleoside binding; ion binding; hydrolase activity, acting on acid anhydrides; hydrolase activity; nucleotide binding; nucleoside-triphosphatase activity; organic cyclic compound binding; heterocyclic compound binding; ribonucleoside binding; carbohydrate derivative binding; pyrophosphatase activity; catalytic activity; ribonucleotide binding; binding; purine ribonucleoside binding; anion binding; purine ribonucleotide binding; adenyl ribonucleotide binding; adenyl nucleotide binding; purine nucleoside binding; hydrolase activity, acting on acid anhydrides, in phosphorus-containing anhydrides; purine nucleotide binding; small molecule binding; purine ribonucleoside triphosphate binding;	K10352	MYH; myosin heavy chain	dre04530 Tight junction - Danio rerio (zebrafish);	IPR002928; IPR004009; IPR001609; IPR027417; IPR027401;	Myosin tail; Myosin, N-terminal, SH3-like; Myosin head, motor domain; P-loop containing nucleoside triphosphate hydrolase; Myosin-like IQ motif-containing domain;
B8A561	myhz1.2	Uncharacterized protein	_HATENK(cr)VK_	976	1	EKHATENKVKNLTEE		nuclear		intracellular organelle; intracellular non-membrane-bounded organelle; cytoskeletal part; intracellular organelle part; macromolecular complex; intracellular part; cell; actin cytoskeleton; organelle part; non-membrane-bounded organelle; organelle; cytoskeleton; cell part; intracellular; myosin complex; protein complex;	ATP binding; motor activity; nucleoside phosphate binding; nucleoside binding; ion binding; hydrolase activity, acting on acid anhydrides; hydrolase activity; nucleotide binding; nucleoside-triphosphatase activity; organic cyclic compound binding; heterocyclic compound binding; ribonucleoside binding; carbohydrate derivative binding; pyrophosphatase activity; catalytic activity; ribonucleotide binding; binding; purine ribonucleoside binding; anion binding; purine ribonucleotide binding; adenyl ribonucleotide binding; adenyl nucleotide binding; purine nucleoside binding; hydrolase activity, acting on acid anhydrides, in phosphorus-containing anhydrides; purine nucleotide binding; small molecule binding; purine ribonucleoside triphosphate binding;	K10352	MYH; myosin heavy chain	dre04530 Tight junction - Danio rerio (zebrafish);	IPR002928; IPR004009; IPR001609; IPR027417; IPR027401;	Myosin tail; Myosin, N-terminal, SH3-like; Myosin head, motor domain; P-loop containing nucleoside triphosphate hydrolase; Myosin-like IQ motif-containing domain;
Q8AY63	ckbb	Brain-subtype creatine kinase	_HEK(cr)FGEILK_	307	1	PNVSKHEKFGEILKR		cytosol			ATP binding; nucleoside phosphate binding; creatine kinase activity; nucleoside binding; ion binding; phosphotransferase activity, nitrogenous group as acceptor; nucleotide binding; organic cyclic compound binding; heterocyclic compound binding; ribonucleoside binding; carbohydrate derivative binding; kinase activity; catalytic activity; ribonucleotide binding; binding; purine ribonucleoside binding; anion binding; purine ribonucleotide binding; adenyl ribonucleotide binding; adenyl nucleotide binding; purine nucleoside binding; transferase activity; transferase activity, transferring phosphorus-containing groups; purine nucleotide binding; small molecule binding; purine ribonucleoside triphosphate binding;	K00933	E2.7.3.2; creatine kinase [EC:2.7.3.2]	dre00330 Arginine and proline metabolism - Danio rerio (zebrafish); dre01100 Metabolic pathways - Danio rerio (zebrafish);	IPR022413; IPR014746; IPR022414;	ATP:guanido phosphotransferase, N-terminal; Glutamine synthetase/guanido kinase, catalytic domain; ATP:guanido phosphotransferase, catalytic domain;
A2BHA3	ckma	Uncharacterized protein	_HNNHMAK(cr)VLTK_	32	1	KHNNHMAKVLTKEMY	.......K.L.....	cytosol			ATP binding; nucleoside phosphate binding; nucleoside binding; ion binding; nucleotide binding; organic cyclic compound binding; heterocyclic compound binding; ribonucleoside binding; carbohydrate derivative binding; kinase activity; catalytic activity; ribonucleotide binding; binding; purine ribonucleoside binding; anion binding; purine ribonucleotide binding; adenyl ribonucleotide binding; adenyl nucleotide binding; purine nucleoside binding; transferase activity; transferase activity, transferring phosphorus-containing groups; purine nucleotide binding; small molecule binding; purine ribonucleoside triphosphate binding;	K00933	E2.7.3.2; creatine kinase [EC:2.7.3.2]	dre00330 Arginine and proline metabolism - Danio rerio (zebrafish); dre01100 Metabolic pathways - Danio rerio (zebrafish);	IPR022413; IPR022414; IPR014746;	ATP:guanido phosphotransferase, N-terminal; ATP:guanido phosphotransferase, catalytic domain; Glutamine synthetase/guanido kinase, catalytic domain;

F1Q9K1	smarcc1a	Uncharacterized protein	_HQGTITEDK(cr)SK_	196	1	QGTITEDKSKATHII	......DK.......	nuclear		organelle; intracellular membrane-bounded organelle; intracellular organelle; nucleus; cell part; intracellular; intracellular part; membrane-bounded organelle; cell;	organic cyclic compound binding; heterocyclic compound binding; DNA binding; binding; nucleic acid binding; macromolecular complex binding; chromatin binding;	K11649			IPR001005; IPR011991; IPR009057; IPR007526; IPR017884; IPR000953; IPR001357;	SANT/Myb domain; Winged helix-turn-helix DNA-binding domain; Homeodomain-like; SWIRM domain; SANT domain; Chromo domain/shadow; BRCT domain;
F6NLI6	ppib	Peptidyl-prolyl cis-trans isomerase	_HVVFGK(cr)ILEGMDVVR_	163	1	GKHVVFGKILEGMDV	.......K.L.....	extracellular				K03768			IPR002130; IPR029000;	Cyclophilin-type peptidyl-prolyl cis-trans isomerase domain; Cyclophilin-like domain;
B8JKN7	ppiaa	Uncharacterized protein	_HVVFGQVVEGLDVIK(cr)K_	80	1	VEGLDVIKKVEGFGS	.......K.V.....	extracellular	cellular protein metabolic process; protein peptidyl-prolyl isomerization; peptidyl-proline modification; primary metabolic process; cellular metabolic process; organic substance metabolic process; protein folding; peptidyl-amino acid modification; metabolic process; cellular macromolecule metabolic process; cellular protein modification process; protein metabolic process; macromolecule metabolic process; macromolecule modification; protein modification process; cellular process;		peptidyl-prolyl cis-trans isomerase activity; isomerase activity; catalytic activity; cis-trans isomerase activity;				IPR002130; IPR029000;	Cyclophilin-type peptidyl-prolyl cis-trans isomerase domain; Cyclophilin-like domain;
B3DFP9	apoa2	Uncharacterized protein	_IAGGLASELEPVVDK(cr)AR_	101	1	ELEPVVDKARLNALG	......DK.......	extracellular	developmental process; cellular component organization; single-multicellular organism process; morphogenesis of an epithelium; epithelium development; epiboly involved in gastrulation with mouth forming second; tissue development; organelle fission; epiboly; morphogenesis of an epithelial sheet; single-organism process; multicellular organismal process; gastrulation; anatomical structure morphogenesis; gastrulation with mouth forming second; tissue morphogenesis; organelle organization; single-organism developmental process; nuclear division; multicellular organismal development; embryo development; cellular process; anatomical structure development; embryonic morphogenesis; cellular component organization or biogenesis;							
Q6IQV0	zgc:86598	Uncharacterized protein	_IAK(cr)VLGTEDLYDYIDK_	247	1	DQLVRIAKVLGTEDL	.......K.L.....	nuclear			ATP binding; nucleoside phosphate binding; phosphotransferase activity, alcohol group as acceptor; nucleoside binding; ion binding; protein kinase activity; nucleotide binding; organic cyclic compound binding; heterocyclic compound binding; ribonucleoside binding; carbohydrate derivative binding; kinase activity; catalytic activity; ribonucleotide binding; binding; purine ribonucleoside binding; protein serine/threonine kinase activity; anion binding; purine ribonucleotide binding; adenyl ribonucleotide binding; adenyl nucleotide binding; purine nucleoside binding; transferase activity; transferase activity, transferring phosphorus-containing groups; purine nucleotide binding; small molecule binding; purine ribonucleoside triphosphate binding;	K03097	CSNK2A; casein kinase II subunit alpha [EC:2.7.11.1]	dre04310 Wnt signaling pathway - Danio rerio (zebrafish); dre05168 Herpes simplex infection - Danio rerio (zebrafish); dre04530 Tight junction - Danio rerio (zebrafish); dre03008 Ribosome biogenesis in eukaryotes - Danio rerio (zebrafish); dre04520 Adherens junction - Danio rerio (zebrafish);	IPR000719; IPR002290; IPR011009;	Protein kinase domain; Serine/threonine/dual specificity protein kinase, catalytic domain; Protein kinase-like domain;
A3KPR3	histh1l	Uncharacterized protein	_IALK(cr)ALVK_	75	1	SRVKIALKALVKRGA	.......K.L.....	nuclear	cellular component biogenesis; cellular component organization; nucleosome organization; cellular macromolecular complex assembly; chromatin assembly or disassembly; protein complex subunit organization; macromolecular complex subunit organization; protein-DNA complex assembly; DNA packaging; nucleosome assembly; chromosome organization; cellular component assembly; macromolecular complex assembly; organelle organization; chromatin organization; protein complex biogenesis; chromatin assembly; cellular process; protein complex assembly; cellular component organization or biogenesis; protein-DNA complex subunit organization; DNA conformation change;	intracellular organelle; intracellular non-membrane-bounded organelle; intracellular organelle part; macromolecular complex; intracellular part; cell; organelle part; DNA bending complex; chromosomal part; nucleosome; non-membrane-bounded organelle; intracellular membrane-bounded organelle; organelle; nucleus; chromatin; cell part; intracellular; chromosome; membrane-bounded organelle; protein complex; protein-DNA complex; DNA packaging complex;	organic cyclic compound binding; heterocyclic compound binding; DNA binding; binding; nucleic acid binding;	K11275			IPR011991; IPR005818;	Winged helix-turn-helix DNA-binding domain; Linker histone H1/H5, domain H15;
B8JKH7	mylz3	Uncharacterized protein	_IDFEAFLPMLK(cr)TVDANQK_	100	1	EAFLPMLKTVDANQK	.......K.V.....	mitochondria			cation binding; metal ion binding; calcium ion binding; binding; ion binding;				IPR002048; IPR011992;	EF-hand domain; EF-hand domain pair;
Q9I8U7	mylz3	Fast skeletal muscle myosin light polypeptide 3	_IDFEAFLPMLK(cr)TVDANQK_	76	1	EAFLPMLKTVDANQK	.......K.V.....	cytosol			cation binding; metal ion binding; calcium ion binding; binding; ion binding;				IPR011992; IPR002048;	EF-hand domain pair; EF-hand domain;

Q6TH14	-	Enolase	_IDK(cr)FMLELDGTENK_	126	1	CKAGAAEKGVPLYRH	.......K.V.....	mitochondria	single-organism carbohydrate catabolic process; glycolytic process; single-organism catabolic process; small molecule metabolic process; single-organism cellular process; single-organism process; carbohydrate metabolic process; primary metabolic process; cellular metabolic process; organic substance metabolic process; carbohydrate catabolic process; single-organism carbohydrate metabolic process; organic substance catabolic process; metabolic process; pyruvate metabolic process; generation of precursor metabolites and energy; single-organism metabolic process; carboxylic acid metabolic process; monocarboxylic acid metabolic process; organic acid metabolic process; cellular process; catabolic process; oxoacid metabolic process;	catalytic complex; cell part; intracellular; cytoplasm; macromolecular complex; intracellular part; cytosolic part; cell; cytosol; protein complex; phosphopyruvate hydratase complex; cytoplasmic part;	magnesium ion binding; phosphopyruvate hydratase activity; catalytic activity; binding; ion binding; metal ion binding; cation binding; lyase activity; hydro-lyase activity; carbon-oxygen lyase activity;	K01689	ENO; enolase [EC:4.2.1.11]	dre00010 Glycolysis / Gluconeogenesis - Danio rerio (zebrafish); dre01230 Biosynthesis of amino acids - Danio rerio (zebrafish); dre01200 Carbon metabolism - Danio rerio (zebrafish); dre01100 Metabolic pathways - Danio rerio (zebrafish); dre03018 RNA degradation - Danio rerio (zebrafish);	IPR029065; IPR020810; IPR029017; IPR020811;	Enolase C-terminal domain-like; Enolase, C-terminal; Enolase N-terminal domain-like; Enolase, N-terminal;
Q6IQX1	myhz2	Myosin, heavy polypeptide 2, fast muscle specific	_IEDEQSLGAQLQK(cr)K_	1108	1	SLGAQLQKKIKELQA	.......K.I.....	cytosol	response to stress; response to stimulus;	intracellular organelle; intracellular non-membrane-bounded organelle; cytoskeletal part; intracellular organelle part; macromolecular complex; intracellular part; cell; actin cytoskeleton; organelle part; non-membrane-bounded organelle; organelle; cytoskeleton; cell part; intracellular; myosin complex; protein complex;	ATP binding; motor activity; nucleoside phosphate binding; nucleoside binding; ion binding; hydrolase activity, acting on acid anhydrides; hydrolase activity; nucleotide binding; nucleoside-triphosphatase activity; organic cyclic compound binding; heterocyclic compound binding; ribonucleoside binding; carbohydrate derivative binding; pyrophosphatase activity; catalytic activity; ribonucleotide binding; binding; purine ribonucleoside binding; anion binding; purine ribonucleotide binding; adenyl ribonucleotide binding; adenyl nucleotide binding; purine nucleoside binding; hydrolase activity, acting on acid anhydrides, in phosphorus-containing anhydrides; purine nucleotide binding; small molecule binding; purine ribonucleoside triphosphate binding;	K10352	MYH; myosin heavy chain	dre04530 Tight junction - Danio rerio (zebrafish);	IPR002928; IPR004009; IPR001609; IPR027417; IPR027401;	Myosin tail; Myosin, N-terminal, SH3-like; Myosin head, motor domain; P-loop containing nucleoside triphosphate hydrolase; Myosin-like IQ motif-containing domain;
B8A561	myhz1.2	Uncharacterized protein	_IEDEQSLGAQLQK(cr)K_	1108	1	SLGAQLQKKIKELQA	.......K.I.....	nuclear		intracellular organelle; intracellular non-membrane-bounded organelle; cytoskeletal part; intracellular organelle part; macromolecular complex; intracellular part; cell; actin cytoskeleton; organelle part; non-membrane-bounded organelle; organelle; cytoskeleton; cell part; intracellular; myosin complex; protein complex;	ATP binding; motor activity; nucleoside phosphate binding; nucleoside binding; ion binding; hydrolase activity, acting on acid anhydrides; hydrolase activity; nucleotide binding; nucleoside-triphosphatase activity; organic cyclic compound binding; heterocyclic compound binding; ribonucleoside binding; carbohydrate derivative binding; pyrophosphatase activity; catalytic activity; ribonucleotide binding; binding; purine ribonucleoside binding; anion binding; purine ribonucleotide binding; adenyl ribonucleotide binding; adenyl nucleotide binding; purine nucleoside binding; hydrolase activity, acting on acid anhydrides, in phosphorus-containing anhydrides; purine nucleotide binding; small molecule binding; purine ribonucleoside triphosphate binding;	K10352	MYH; myosin heavy chain	dre04530 Tight junction - Danio rerio (zebrafish);	IPR002928; IPR004009; IPR001609; IPR027417; IPR027401;	Myosin tail; Myosin, N-terminal, SH3-like; Myosin head, motor domain; P-loop containing nucleoside triphosphate hydrolase; Myosin-like IQ motif-containing domain;
Q6TH14	-	Enolase	_IEEELGDK(cr)AK_	420	1	IEEELGDKAKFAGKD	......DK.......	mitochondria	single-organism carbohydrate catabolic process; glycolytic process; single-organism catabolic process; small molecule metabolic process; single-organism cellular process; single-organism process; carbohydrate metabolic process; primary metabolic process; cellular metabolic process; organic substance metabolic process; carbohydrate catabolic process; single-organism carbohydrate metabolic process; organic substance catabolic process; metabolic process; pyruvate metabolic process; generation of precursor metabolites and energy; single-organism metabolic process; carboxylic acid metabolic process; monocarboxylic acid metabolic process; organic acid metabolic process; cellular process; catabolic process; oxoacid metabolic process;	catalytic complex; cell part; intracellular; cytoplasm; macromolecular complex; intracellular part; cytosolic part; cell; cytosol; protein complex; phosphopyruvate hydratase complex; cytoplasmic part;	magnesium ion binding; phosphopyruvate hydratase activity; catalytic activity; binding; ion binding; metal ion binding; cation binding; lyase activity; hydro-lyase activity; carbon-oxygen lyase activity;	K01689	ENO; enolase [EC:4.2.1.11]	dre00010 Glycolysis / Gluconeogenesis - Danio rerio (zebrafish); dre01230 Biosynthesis of amino acids - Danio rerio (zebrafish); dre01200 Carbon metabolism - Danio rerio (zebrafish); dre01100 Metabolic pathways - Danio rerio (zebrafish); dre03018 RNA degradation - Danio rerio (zebrafish);	IPR029065; IPR020810; IPR029017; IPR020811;	Enolase C-terminal domain-like; Enolase, C-terminal; Enolase N-terminal domain-like; Enolase, N-terminal;
Q6PC12	eno1a	Enolase 1, (Alpha)	_IEEELGDK(cr)AR_	420	1	IEEELGDKARFAGKN	......DK.......	cytosol	single-organism carbohydrate catabolic process; glycolytic process; single-organism catabolic process; small molecule metabolic process; single-organism cellular process; single-organism process; carbohydrate metabolic process; primary metabolic process; cellular metabolic process; organic substance metabolic process; carbohydrate catabolic process; single-organism carbohydrate metabolic process; organic substance catabolic process; metabolic process; pyruvate metabolic process; generation of precursor metabolites and energy; single-organism metabolic process; carboxylic acid metabolic process; monocarboxylic acid metabolic process; organic acid metabolic process; cellular process; catabolic process; oxoacid metabolic process;	catalytic complex; cell part; intracellular; cytoplasm; macromolecular complex; intracellular part; cytosolic part; cell; cytosol; protein complex; phosphopyruvate hydratase complex; cytoplasmic part;	magnesium ion binding; phosphopyruvate hydratase activity; catalytic activity; binding; ion binding; metal ion binding; cation binding; lyase activity; hydro-lyase activity; carbon-oxygen lyase activity;	K01689	ENO; enolase [EC:4.2.1.11]	dre00010 Glycolysis / Gluconeogenesis - Danio rerio (zebrafish); dre01230 Biosynthesis of amino acids - Danio rerio (zebrafish); dre01200 Carbon metabolism - Danio rerio (zebrafish); dre01100 Metabolic pathways - Danio rerio (zebrafish); dre03018 RNA degradation - Danio rerio (zebrafish);	IPR029065; IPR029017; IPR020810; IPR020811;	Enolase C-terminal domain-like; Enolase N-terminal domain-like; Enolase, C-terminal; Enolase, N-terminal;
Q804W0	pvalb1	Parvalbumin 1	_IGAEEFAALVK(cr)A_	108	1	EEFAALVKA______		cytosol			cation binding; metal ion binding; calcium ion binding; binding; ion binding;				IPR011992; IPR002048;	EF-hand domain pair; EF-hand domain;
Q9I8V0	pvalb2	Parvalbumin-2	_IGVDEFALLVK(cr)A_	108	1	DEFALLVKA______		cytosol			cation binding; metal ion binding; calcium ion binding; binding; ion binding;				IPR002048; IPR011992;	EF-hand domain; EF-hand domain pair;

Q90ZM2	sec61al1	Protein transport protein Sec61 subunit alpha-like 1	_IIEVGDTPK(cr)DR_	107	1	IEVGDTPKDRALFNG		plasma membrane	developmental process; nervous system development; single-multicellular organism process; brain development; system development; single-organism process; organic substance transport; multicellular organismal process; protein transport; establishment of localization; establishment of protein localization; transport; organ development; habenula development; single-organism developmental process; diencephalon development; central nervous system development; head development; forebrain development; multicellular organismal development; protein localization; macromolecule localization; localization; anatomical structure development; epithalamus development;	membrane; intracellular organelle; endoplasmic reticulum; nuclear outer membrane-endoplasmic reticulum membrane network; bounding membrane of organelle; organelle membrane; cytoplasm; intracellular organelle part; intrinsic component of membrane; endomembrane system; intracellular part; cell; integral component of membrane; organelle part; intracellular membrane-bounded organelle; organelle; endoplasmic reticulum part; intracellular; cell part; membrane part; membrane region; membrane-bounded organelle; endoplasmic reticulum membrane; cytoplasmic part;		K10956	SEC61A; protein transport protein SEC61 subunit alpha	dre03060 Protein export - Danio rerio (zebrafish); dre04145 Phagosome - Danio rerio (zebrafish); dre04141 Protein processing in endoplasmic reticulum - Danio rerio (zebrafish);	IPR023201; IPR019561;	SecY subunit domain; Translocon Sec61/SecY, plug domain;
F1RBK3	-	Pyruvate kinase	_IISK(cr)LENHEGVR_	269	1	KDIRIISKLENHEGV	.......K.E.....	cytosol	single-organism carbohydrate catabolic process; glycolytic process; single-organism catabolic process; small molecule metabolic process; single-organism cellular process; single-organism process; carbohydrate metabolic process; primary metabolic process; cellular metabolic process; organic substance metabolic process; carbohydrate catabolic process; single-organism carbohydrate metabolic process; organic substance catabolic process; metabolic process; pyruvate metabolic process; generation of precursor metabolites and energy; single-organism metabolic process; carboxylic acid metabolic process; monocarboxylic acid metabolic process; organic acid metabolic process; cellular process; catabolic process; oxoacid metabolic process;		magnesium ion binding; pyruvate kinase activity; kinase activity; phosphotransferase activity, alcohol group as acceptor; catalytic activity; binding; ion binding; potassium ion binding; metal ion binding; cation binding; alkali metal ion binding; transferase activity; transferase activity, transferring phosphorus-containing groups;	K00873	PK; pyruvate kinase [EC:2.7.1.40]	dre00010 Glycolysis / Gluconeogenesis - Danio rerio (zebrafish); dre01230 Biosynthesis of amino acids - Danio rerio (zebrafish); dre01200 Carbon metabolism - Danio rerio (zebrafish); dre01100 Metabolic pathways - Danio rerio (zebrafish); dre00620 Pyruvate metabolism - Danio rerio (zebrafish); dre00230 Purine metabolism - Danio rerio (zebrafish);	IPR015794; IPR011037; IPR015813; IPR015806; IPR015795; IPR015793;	Pyruvate kinase, alpha/beta; Pyruvate kinase-like, insert domain; Pyruvate/Phosphoenolpyruvate kinase-like domain; Pyruvate kinase, beta-barrel insert domain; Pyruvate kinase, C-terminal; Pyruvate kinase, barrel;
B3DFN3	lmnb2	Lamin B2	_IK(cr)DLEAQYNK_	130	1	AAALVRIKDLEAQYN	.......K.L.....	nuclear		intracellular organelle; intracellular non-membrane-bounded organelle; cytoskeletal part; intracellular organelle part; macromolecular complex; intracellular part; cell; organelle part; non-membrane-bounded organelle; organelle; cytoskeleton; intermediate filament cytoskeleton; cell part; intracellular; intermediate filament; protein complex;	structural molecule activity;	K07611			IPR001322;	Lamin Tail Domain;
F1QIR4	-	Uncharacterized protein	_IK(cr)ELTYQTEEDRK_	1851	1	RKYERRIKELTYQTE	.......K.L.....	cytosol	cellular component biogenesis; muscle cell development; single-multicellular organism process; cellular component organization; muscle system process; cellular developmental process; cellular component morphogenesis; single-organism process; actin filament organization; cell development; anatomical structure morphogenesis; cell differentiation; actin cytoskeleton organization; muscle contraction; cellular component assembly; organelle assembly; macromolecular complex assembly; myofibril assembly; musculoskeletal movement; slow-twitch skeletal muscle fiber contraction; muscle structure development; cytoskeleton organization; anatomical structure formation involved in morphogenesis; cellular process; striated muscle myosin thick filament assembly; protein complex assembly; cellular protein complex assembly; skeletal muscle thin filament assembly; developmental process; cellular macromolecular complex assembly; striated muscle cell differentiation; actomyosin structure organization; system process; protein complex subunit organization; multicellular organismal movement; single-organism cellular process; cellular component assembly involved in morphogenesis; striated muscle contraction; myosin filament	intracellular organelle; intracellular non-membrane-bounded organelle; cytoskeletal part; intracellular organelle part; macromolecular complex; intracellular part; cell; actin cytoskeleton; organelle part; non-membrane-bounded organelle; organelle; cytoskeleton; cell part; intracellular; myosin complex; protein complex;	ATP binding; motor activity; nucleoside phosphate binding; nucleoside binding; ion binding; hydrolase activity, acting on acid anhydrides; hydrolase activity; nucleotide binding; nucleoside-triphosphatase activity; organic cyclic compound binding; heterocyclic compound binding; ribonucleoside binding; carbohydrate derivative binding; pyrophosphatase activity; catalytic activity; ribonucleotide binding; binding; purine ribonucleoside binding; anion binding; purine ribonucleotide binding; adenyl ribonucleotide binding; adenyl nucleotide binding; purine nucleoside binding; hydrolase activity, acting on acid anhydrides, in phosphorus-containing anhydrides; purine nucleotide binding; small molecule binding; purine ribonucleoside triphosphate binding;	K10352	MYH; myosin heavy chain	dre04530 Tight junction - Danio rerio (zebrafish);	IPR002928; IPR004009; IPR027417; IPR001609; IPR027401;	Myosin tail; Myosin, N-terminal, SH3-like; P-loop containing nucleoside triphosphate hydrolase; Myosin head, motor domain; Myosin-like IQ motif-containing domain;
Q9I8V1	actc1b	Actin, alpha 1, skeletal muscle	_IK(cr)IIAPPER_	330	1	APSTMKIKIIAPPER	.......K.I.....	cytoskeleton		cytoskeleton; organelle; intracellular organelle; intracellular non-membrane-bounded organelle; cell part; intracellular; cytoplasm; intracellular part; cell; non-membrane-bounded organelle;	ATP binding; nucleoside phosphate binding; nucleoside binding; ion binding; nucleotide binding; organic cyclic compound binding; heterocyclic compound binding; ribonucleoside binding; carbohydrate derivative binding; ribonucleotide binding; binding; purine ribonucleoside binding; anion binding; purine ribonucleotide binding; adenyl ribonucleotide binding; adenyl nucleotide binding; purine nucleoside binding; purine nucleotide binding; small molecule binding; purine ribonucleoside triphosphate binding;	K10354				

Q6P3J5	eef2b	Eukaryotic translation elongation factor 2	_IK(cr)PVLMMNK_	152	1	QAIAERIKPVLMMNK	.......K.V.....	cytosol			nucleoside phosphate binding; translation elongation factor activity; hydrolase activity, acting on acid anhydrides; hydrolase activity; organic cyclic compound binding; heterocyclic compound binding; pyrophosphatase activity; catalytic activity; ribonucleotide binding; binding; GTPase activity; purine ribonucleoside binding; anion binding; hydrolase activity, acting on acid anhydrides, in phosphorus-containing anhydrides; guanyl nucleotide binding; GTP binding; purine nucleotide binding; purine ribonucleoside triphosphate binding; RNA binding; ion binding; nucleoside binding; nucleotide binding; nucleoside-triphosphatase activity; ribonucleoside binding; translation factor activity, nucleic acid binding; carbohydrate derivative binding; nucleic acid binding; purine ribonucleotide binding; guanyl ribonucleotide binding; purine nucleoside binding; small molecule binding;	K03234			IPR027417; IPR000795; IPR005517; IPR005225; IPR004161; IPR000640; IPR020568; IPR014721; IPR009022; IPR009000;	P-loop containing nucleoside triphosphate hydrolase; Elongation factor, GTP-binding domain; Translation elongation factor EFG/EF2, domain IV; Small GTP-binding protein domain; Translation elongation factor EFTu/EF1A, domain 2; Translation elongation factor EFG, V domain; Ribosomal protein S5 domain 2-type fold; Ribosomal protein S5 domain 2-type fold, subgroup; Elongation factor G, III-V domain; Translation protein, beta-barrel domain;
E9QDR0	rps2	Uncharacterized protein	_IK(cr)SLEEIYLYSLPIK_	62	1	LVKDMKIKSLEEIYL	.......K.L.....	nuclear	cellular protein metabolic process; biosynthetic process; primary metabolic process; cellular metabolic process; organic substance biosynthetic process; cellular biosynthetic process; organic substance metabolic process; metabolic process; cellular macromolecule biosynthetic process; cellular macromolecule metabolic process; gene expression; macromolecule metabolic process; protein metabolic process; macromolecule biosynthetic process; translation; cellular process;	ribosome; organelle; intracellular organelle; intracellular non-membrane-bounded organelle; cell part; intracellular; cytoplasm; macromolecular complex; intracellular part; cell; ribonucleoprotein complex; cytoplasmic part; non-membrane-bounded organelle;	organic cyclic compound binding; heterocyclic compound binding; RNA binding; binding; nucleic acid binding; structural molecule activity; structural constituent of ribosome;				IPR014720; IPR013810;	Double-stranded RNA-binding domain; Ribosomal protein S5, N-terminal;
A3KPI6	si:ch211-288g17.3	Uncharacterized protein	_IK(cr)VVVVK_	247	1	PGTGRKIKVVVVKKV	.......K.V.....	nuclear			organic cyclic compound binding; heterocyclic compound binding; DNA binding; binding; nucleic acid binding;					
B8JKH7	mylz3	Uncharacterized protein	_ILGDPSADDM(ox)ANK(cr)R_	88	1	SADDMANKRIDFEAF	.......K.I.....	mitochondria			cation binding; metal ion binding; calcium ion binding; binding; ion binding;				IPR002048; IPR011992;	EF-hand domain; EF-hand domain pair;
Q6P0G6	myl1	Uncharacterized protein	_ILGNPTADDMVNK(cr)R_	102	1	TADDMVNKRVDFEGF	.......K.V.....	cytosol			cation binding; metal ion binding; calcium ion binding; binding; ion binding;	K05738			IPR002048; IPR011992;	EF-hand domain; EF-hand domain pair;
Q6P5L3	rpl19	60S ribosomal protein L19	_ILMEHIHK(cr)LK_	144	1	ILMEHIHKLKADKAR		cytosol	cellular protein metabolic process; biosynthetic process; primary metabolic process; cellular metabolic process; organic substance biosynthetic process; cellular biosynthetic process; organic substance metabolic process; metabolic process; cellular macromolecule biosynthetic process; cellular macromolecule metabolic process; gene expression; macromolecule metabolic process; protein metabolic process; macromolecule biosynthetic process; translation; cellular process;	ribosome; organelle; intracellular organelle; intracellular non-membrane-bounded organelle; cell part; intracellular; cytoplasm; macromolecular complex; intracellular part; cell; ribonucleoprotein complex; cytoplasmic part; non-membrane-bounded organelle;	structural constituent of ribosome; structural molecule activity;	K02885	RP-L19e; large subunit ribosomal protein L19e	dre03010 Ribosome - Danio rerio (zebrafish);	IPR000196; IPR015974; IPR015972;	Ribosomal protein L19/L19e domain; Ribosomal protein L19/L19e, domain 3; Ribosomal protein L19/L19e, domain 1;
F1QHC4	lmnb1	Uncharacterized protein	_INELEGALSHEK(cr)DLSR_	333	1	EGALSHEKDLSRRLL	.......K.L.....	nuclear		intracellular organelle; intracellular non-membrane-bounded organelle; cytoskeletal part; intracellular organelle part; macromolecular complex; intracellular part; cell; organelle part; non-membrane-bounded organelle; organelle; cytoskeleton; intermediate filament cytoskeleton; cell part; intracellular; intermediate filament; protein complex;	structural molecule activity;	K07611			IPR001322;	Lamin Tail Domain;
Q5TZ29	apobb.1	Uncharacterized protein	_IPALPEFTFPK(cr)K_	1208	1	LPEFTFPKKLFLNAE	.......K.L.....	endoplasmic reticulum	response to chemical; response to stimulus;		lipid transporter activity; substrate-specific transporter activity; transporter activity;				IPR011030; IPR015817; IPR015818; IPR001747; IPR015819; IPR015816; IPR016024; IPR015255; IPR009454;	Vitellinogen, superhelical; Vitellinogen, open beta-sheet, subdomain 1; Vitellinogen, open beta-sheet, subdomain 2; Lipid transport protein, N-terminal; Lipid transport protein, beta-sheet shell; Vitellinogen, beta-sheet N-terminal; Armadillo-type fold; Vitellinogen, open beta-sheet; Lipid transport, open beta-sheet;
E7EXP0	tnnt3b	Uncharacterized protein	_IPDGDK(cr)VDFDDIQK_	32	1	PKIPDGDKVDFDDIQ	......DK.......	nuclear				K12046				
E7EXP0	tnnt3b	Uncharacterized protein	_IPDGDKVDFDDIQK(cr)K_	40	1	VDFDDIQKKRQNKDL		nuclear				K12046				
F1R1J9	ahnak	Uncharacterized protein	_IPK(cr)TDIDIK_	1275	1	KVKGSKFKIPFSGPT		nuclear			catalytic activity; oxidoreductase activity;				IPR001478; IPR011254;	PDZ domain; Prismane-like;

E9QF69	rpl18	60S ribosomal protein L18	_IQNIPK(cr)LK_	97	1	VRIQNIPKLKVCALK		mitochondria	cellular protein metabolic process; biosynthetic process; primary metabolic process; cellular metabolic process; organic substance biosynthetic process; cellular biosynthetic process; organic substance metabolic process; metabolic process; cellular macromolecule biosynthetic process; cellular macromolecule metabolic process; gene expression; macromolecule metabolic process; protein metabolic process; macromolecule biosynthetic process; translation; cellular process;	ribosome; organelle; intracellular organelle; intracellular non-membrane-bounded organelle; cell part; intracellular; cytoplasm; macromolecular complex; intracellular part; cell; ribonucleoprotein complex; cytoplasmic part; non-membrane-bounded organelle;	structural constituent of ribosome; structural molecule activity;				IPR021131;	Ribosomal protein L18e/L15P;
Q6PFT7	cotl1	Coactosin-like 1	_ISTDK(cr)ALVK_	98	1	RAKISTDKALVKDAV	......DK.......	cytosol,nuclear		cell; intracellular; cell part;					IPR002108; IPR029006;	Actin-depolymerising factor homology domain; ADF-H/Gelsolin-like domain;
F1QXV8	pgk1	Phosphoglycerate kinase	_ITLPVDFITADK(cr)FDEK_	291	1	VDFITADKFDEKATT	......DK.......	cytosol	single-organism carbohydrate catabolic process; glycolytic process; single-organism catabolic process; small molecule metabolic process; single-organism cellular process; single-organism process; carbohydrate metabolic process; primary metabolic process; cellular metabolic process; organic substance metabolic process; carbohydrate catabolic process; single-organism carbohydrate metabolic process; organic substance catabolic process; metabolic process; pyruvate metabolic process; generation of precursor metabolites and energy; single-organism metabolic process; carboxylic acid metabolic process; monocarboxylic acid metabolic process; organic acid metabolic process; cellular process; catabolic process; oxoacid metabolic process;		phosphotransferase activity, carboxyl group as acceptor; kinase activity; catalytic activity; transferase activity; phosphoglycerate kinase activity; transferase activity, transferring phosphorus-containing groups;	K00927	PGK; phosphoglycerate kinase [EC:2.7.2.3]	dre00010 Glycolysis / Gluconeogenesis - Danio rerio (zebrafish); dre01230 Biosynthesis of amino acids - Danio rerio (zebrafish); dre01200 Carbon metabolism - Danio rerio (zebrafish); dre01100 Metabolic pathways - Danio rerio (zebrafish);	IPR015824; IPR015901;	Phosphoglycerate kinase, N-terminal; Phosphoglycerate kinase, C-terminal;
F1R2S5	vtg5	Uncharacterized protein (Fragment)	_IVTTYAK(cr)K_	1277	1	KIVTTYAKKVCKHIL	.......K.V.....	plasma membrane			lipid transporter activity; substrate-specific transporter activity; transporter activity;				IPR015816; IPR015258; IPR011030; IPR015819; IPR015817; IPR015818; IPR001747; IPR015255;	Vitellinogen, beta-sheet N-terminal; Vitellinogen, beta-sheet shell; Vitellinogen, superhelical; Lipid transport protein, beta-sheet shell; Vitellinogen, open beta-sheet, subdomain 1; Vitellinogen, open beta-sheet, subdomain 2; Lipid transport protein, N-terminal; Vitellinogen, open beta-sheet;
Q7ZWJ7	rpl34	60S ribosomal protein L34	_IVVK(cr)VLK_	105	1	EEQKIVVKVLKAQAQ	.......K.L.....	nuclear	cellular protein metabolic process; biosynthetic process; primary metabolic process; cellular metabolic process; organic substance biosynthetic process; cellular biosynthetic process; organic substance metabolic process; metabolic process; cellular macromolecule biosynthetic process; cellular macromolecule metabolic process; gene expression; macromolecule metabolic process; protein metabolic process; macromolecule biosynthetic process; translation; cellular process;	ribosome; organelle; intracellular organelle; intracellular non-membrane-bounded organelle; cell part; intracellular; cytoplasm; macromolecular complex; intracellular part; cell; ribonucleoprotein complex; cytoplasmic part; non-membrane-bounded organelle;	structural constituent of ribosome; structural molecule activity;	K02915	RP-L34e; large subunit ribosomal protein L34e	dre03010 Ribosome - Danio rerio (zebrafish);		
Q804W0	pvalb1	Parvalbumin 1	_K(cr)AFAIIDQDNSGFIEEEELK_	46	1	KSADEVKKAFAIIDQ		cytosol			cation binding; metal ion binding; calcium ion binding; binding; ion binding;				IPR011992; IPR002048;	EF-hand domain pair; EF-hand domain;
Q9I8V0	pvalb2	Parvalbumin-2	_K(cr)AFFVIDQDK_	46	1	KSPDDIKKAFFVIDQ		cytosol			cation binding; metal ion binding; calcium ion binding; binding; ion binding;				IPR002048; IPR011992;	EF-hand domain; EF-hand domain pair;
B8A568	myhz1.1	Uncharacterized protein	_K(cr)AITDAAMMAEELKK_	1759	1	NAEDKAKKAITDAAM	.......K.I.....	nuclear		intracellular organelle; intracellular non-membrane-bounded organelle; cytoskeletal part; intracellular organelle part; macromolecular complex; intracellular part; cell; actin cytoskeleton; organelle part; non-membrane-bounded organelle; organelle; cytoskeleton; cell part; intracellular; myosin complex; protein complex;	ATP binding; motor activity; nucleoside phosphate binding; nucleoside binding; ion binding; hydrolase activity, acting on acid anhydrides; hydrolase activity; nucleotide binding; nucleoside-triphosphatase activity; organic cyclic compound binding; heterocyclic compound binding; ribonucleoside binding; carbohydrate derivative binding; pyrophosphatase activity; catalytic activity; ribonucleotide binding; binding; purine ribonucleoside binding; anion binding; purine ribonucleotide binding; adenyl ribonucleotide binding; adenyl nucleotide binding; purine nucleoside binding; hydrolase activity, acting on acid anhydrides, in phosphorus-containing anhydrides; purine nucleotide binding; small molecule binding; purine ribonucleoside triphosphate binding;	K10352	MYH; myosin heavy chain	dre04530 Tight junction - Danio rerio (zebrafish);	IPR002928; IPR004009; IPR001609; IPR027417; IPR027401;	Myosin tail; Myosin, N-terminal, SH3-like; Myosin head, motor domain; P-loop containing nucleoside triphosphate hydrolase; Myosin-like IQ motif-containing domain;
B8A561	myhz1.2	Uncharacterized protein	_K(cr)AITDAAMMAEELKK_	1759	1	NAEDKAKKAITDAAM	.......K.I.....	nuclear		intracellular organelle; intracellular non-membrane-bounded organelle; cytoskeletal part; intracellular organelle part; macromolecular complex; intracellular part; cell; actin cytoskeleton; organelle part; non-membrane-bounded organelle; organelle; cytoskeleton; cell part; intracellular; myosin complex; protein complex;	ATP binding; motor activity; nucleoside phosphate binding; nucleoside binding; ion binding; hydrolase activity, acting on acid anhydrides; hydrolase activity; nucleotide binding; nucleoside-triphosphatase activity; organic cyclic compound binding; heterocyclic compound binding; ribonucleoside binding; carbohydrate derivative binding; pyrophosphatase activity; catalytic activity; ribonucleotide binding; binding; purine ribonucleoside binding; anion binding; purine ribonucleotide binding; adenyl ribonucleotide binding; adenyl nucleotide binding; purine nucleoside binding; hydrolase activity, acting on acid anhydrides, in phosphorus-containing anhydrides; purine nucleotide binding; small molecule binding; purine ribonucleoside triphosphate binding;	K10352	MYH; myosin heavy chain	dre04530 Tight junction - Danio rerio (zebrafish);	IPR002928; IPR004009; IPR001609; IPR027417; IPR027401;	Myosin tail; Myosin, N-terminal, SH3-like; Myosin head, motor domain; P-loop containing nucleoside triphosphate hydrolase; Myosin-like IQ motif-containing domain;

B8A561	myhz1.2	Uncharacterized protein	_K(cr)AVETEK_	1533	1	HELEKSKKAVETEKA	.......K.V.....	nuclear		intracellular organelle; intracellular non-membrane-bounded organelle; cytoskeletal part; intracellular organelle part; macromolecular complex; intracellular part; cell; actin cytoskeleton; organelle part; non-membrane-bounded organelle; organelle; cytoskeleton; cell part; intracellular; myosin complex; protein complex;	ATP binding; motor activity; nucleoside phosphate binding; nucleoside binding; ion binding; hydrolase activity, acting on acid anhydrides; hydrolase activity; nucleotide binding; nucleoside-triphosphatase activity; organic cyclic compound binding; heterocyclic compound binding; ribonucleoside binding; carbohydrate derivative binding; pyrophosphatase activity; catalytic activity; ribonucleotide binding; binding; purine ribonucleoside binding; anion binding; purine ribonucleotide binding; adenyl ribonucleotide binding; adenyl nucleotide binding; purine nucleoside binding; hydrolase activity, acting on acid anhydrides, in phosphorus-containing anhydrides; purine nucleotide binding; small molecule binding; purine ribonucleoside triphosphate binding;	K10352	MYH; myosin heavy chain	dre04530 Tight junction - Danio rerio (zebrafish);	IPR002928; IPR004009; IPR001609; IPR027417; IPR027401;	Myosin tail; Myosin, N-terminal, SH3-like; Myosin head, motor domain; P-loop containing nucleoside triphosphate hydrolase; Myosin-like IQ motif-containing domain;
B8A568	myhz1.1	Uncharacterized protein	_K(cr)DFETSQLLSK_	1085	1	SEEKLKKKDFETSQL	..E....K.......	nuclear		intracellular organelle; intracellular non-membrane-bounded organelle; cytoskeletal part; intracellular organelle part; macromolecular complex; intracellular part; cell; actin cytoskeleton; organelle part; non-membrane-bounded organelle; organelle; cytoskeleton; cell part; intracellular; myosin complex; protein complex;	ATP binding; motor activity; nucleoside phosphate binding; nucleoside binding; ion binding; hydrolase activity, acting on acid anhydrides; hydrolase activity; nucleotide binding; nucleoside-triphosphatase activity; organic cyclic compound binding; heterocyclic compound binding; ribonucleoside binding; carbohydrate derivative binding; pyrophosphatase activity; catalytic activity; ribonucleotide binding; binding; purine ribonucleoside binding; anion binding; purine ribonucleotide binding; adenyl ribonucleotide binding; adenyl nucleotide binding; purine nucleoside binding; hydrolase activity, acting on acid anhydrides, in phosphorus-containing anhydrides; purine nucleotide binding; small molecule binding; purine ribonucleoside triphosphate binding;	K10352	MYH; myosin heavy chain	dre04530 Tight junction - Danio rerio (zebrafish);	IPR002928; IPR004009; IPR001609; IPR027417; IPR027401;	Myosin tail; Myosin, N-terminal, SH3-like; Myosin head, motor domain; P-loop containing nucleoside triphosphate hydrolase; Myosin-like IQ motif-containing domain;
B8A561	myhz1.2	Uncharacterized protein	_K(cr)DFETSQLLSK_	1085	1	SEEKLKKKDFETSQL	..E....K.......	nuclear		intracellular organelle; intracellular non-membrane-bounded organelle; cytoskeletal part; intracellular organelle part; macromolecular complex; intracellular part; cell; actin cytoskeleton; organelle part; non-membrane-bounded organelle; organelle; cytoskeleton; cell part; intracellular; myosin complex; protein complex;	ATP binding; motor activity; nucleoside phosphate binding; nucleoside binding; ion binding; hydrolase activity, acting on acid anhydrides; hydrolase activity; nucleotide binding; nucleoside-triphosphatase activity; organic cyclic compound binding; heterocyclic compound binding; ribonucleoside binding; carbohydrate derivative binding; pyrophosphatase activity; catalytic activity; ribonucleotide binding; binding; purine ribonucleoside binding; anion binding; purine ribonucleotide binding; adenyl ribonucleotide binding; adenyl nucleotide binding; purine nucleoside binding; hydrolase activity, acting on acid anhydrides, in phosphorus-containing anhydrides; purine nucleotide binding; small molecule binding; purine ribonucleoside triphosphate binding;	K10352	MYH; myosin heavy chain	dre04530 Tight junction - Danio rerio (zebrafish);	IPR002928; IPR004009; IPR001609; IPR027417; IPR027401;	Myosin tail; Myosin, N-terminal, SH3-like; Myosin head, motor domain; P-loop containing nucleoside triphosphate hydrolase; Myosin-like IQ motif-containing domain;
E9QDY3	cyt1	Uncharacterized protein	_K(cr)DLESWFQAK_	288	1	AVSAKNRKDLESWFQ	.......K.L.....	mitochondria		intracellular organelle; intracellular non-membrane-bounded organelle; cytoskeletal part; intracellular organelle part; macromolecular complex; intracellular part; cell; organelle part; non-membrane-bounded organelle; organelle; cytoskeleton; intermediate filament cytoskeleton; cell part; intracellular; intermediate filament; protein complex;	structural molecule activity;	K07604				
E7FES0	-	Uncharacterized protein	_K(cr)ELTELGEQVKPHFEGIFK_	230	1	VKSGELRKELTELGE	.......K.L.....	extracellular	lipid transport; single-organism process; lipid localization; organic substance transport; primary metabolic process; lipoprotein metabolic process; organic substance metabolic process; establishment of localization; transport; metabolic process; single-organism localization; protein metabolic process; macromolecule metabolic process; localization; macromolecule localization; single-organism transport;	extracellular region;	lipid binding; binding;					
Q8AX99	actn3b	Actn3b	_K(cr)HEAFESDLAAHQDR_	447	1	EIRALMRKHEAFESD	.......K.E.....	cytosol	cellular component biogenesis; cellular component organization; actin crosslink formation; protein complex subunit organization; single-organism cellular process; actin filament organization; single-organism process; actin filament bundle assembly; actin filament bundle organization; macromolecular complex subunit organization; single-organism organelle organization; actin cytoskeleton organization; cellular component assembly; organelle organization; actin filament-based process; cytoskeleton organization; cellular process; cellular component organization or biogenesis;		cation binding; metal ion binding; calcium ion binding; binding; ion binding;	K05699	ACTN; actinin alpha	dre04510 Focal adhesion - Danio rerio (zebrafish); dre04530 Tight junction - Danio rerio (zebrafish); dre04810 Regulation of actin cytoskeleton - Danio rerio (zebrafish); dre04520 Adherens junction - Danio rerio (zebrafish);	IPR014837; IPR002048; IPR011992; IPR001715;	EF-hand, Ca insensitive; EF-hand domain; EF-hand domain pair; Calponin homology domain;
D1GJ56	actn3a	Actn3a	_K(cr)HEAFESDLAAHQDR_	445	1	EIRALMRKHEAFESD	.......K.E.....	cytosol	cellular component biogenesis; cellular component organization; actin crosslink formation; protein complex subunit organization; single-organism cellular process; actin filament organization; single-organism process; actin filament bundle assembly; actin filament bundle organization; macromolecular complex subunit organization; single-organism organelle organization; actin cytoskeleton organization; cellular component assembly; organelle organization; actin filament-based process; cytoskeleton organization; cellular process; cellular component organization or biogenesis;		cation binding; metal ion binding; calcium ion binding; binding; ion binding;	K05699	ACTN; actinin alpha	dre04510 Focal adhesion - Danio rerio (zebrafish); dre04530 Tight junction - Danio rerio (zebrafish); dre04810 Regulation of actin cytoskeleton - Danio rerio (zebrafish); dre04520 Adherens junction - Danio rerio (zebrafish);	IPR014837; IPR011992; IPR002048; IPR001715;	EF-hand, Ca insensitive; EF-hand domain pair; EF-hand domain; Calponin homology domain;

F1R446	spna2	Uncharacterized protein	_K(cr)HEDFDK_	1489	1	SVEALIKKHEDFDKA	..E....K.......	cytosol,nucle ar	developmental process; nervous system development; cellular component organization; single-multicellular organism process; developmental maturation; generation of neurons; system development; cellular developmental process; neuron development; single-organism cellular process; single-organism membrane organization; single-organism process; neurogenesis; neuronal ion channel clustering; multicellular organismal process; cell development; neuron differentiation; cell differentiation; single-organism developmental process; clustering of voltage-gated sodium channels; membrane organization; cell maturation; multicellular organismal development; neuron maturation; cellular process; anatomical structure development; cellular component organization or biogenesis;	axon; cell projection; cell part; main axon; neuron projection; paranode region of axon; cell projection part; cell; neuron part; axon part; node of Ranvier;	cation binding; metal ion binding; calcium ion binding; binding; ion binding;	K06114			IPR014837; IPR013315; IPR001452; IPR002048; IPR011992;	EF-hand, Ca insensitive; Spectrin alpha chain, SH3 domain; SH3 domain; EF-hand domain; EF-hand domain pair;
F1QS28	hnrnpa0a	Uncharacterized protein	_K(cr)IFIGGLK_	102	1	EALAKVKKIFIGGLK		nuclear			organic cyclic compound binding; heterocyclic compound binding; nucleoside phosphate binding; binding; nucleic acid binding; nucleotide binding; small molecule binding;	K12894			IPR000504; IPR012677;	RNA recognition motif domain; Nucleotide-binding, alpha-beta plait;
Q9I8U7	mylz3	Fast skeletal muscle myosin light polypeptide 3	_K(cr)ILGDPSADDM(ox)ANK_	51	1	PTNKDVKKILGDPSA	.......K.L.....	cytosol			cation binding; metal ion binding; calcium ion binding; binding; ion binding;				IPR011992; IPR002048;	EF-hand domain pair; EF-hand domain;
B8JKH7	mylz3	Uncharacterized protein	_K(cr)ILGDPSADDMANK_	75	1	PTNKDVKKILGDPSA	.......K.L.....	mitochondria			cation binding; metal ion binding; calcium ion binding; binding; ion binding;				IPR002048; IPR011992;	EF-hand domain; EF-hand domain pair;
Q9I8U7	mylz3	Fast skeletal muscle myosin light polypeptide 3	_K(cr)ILGDPSADDMANK_	51	1	PTNKDVKKILGDPSA	.......K.L.....	cytosol			cation binding; metal ion binding; calcium ion binding; binding; ion binding;				IPR011992; IPR002048;	EF-hand domain pair; EF-hand domain;
Q5TZ29	apobb.1	Uncharacterized protein	_K(cr)LFLNAEGAAK_	1209	1	PEFTFPKKLFLNAEG		endoplasmic reticulum	response to chemical; response to stimulus;		lipid transporter activity; substrate-specific transporter activity; transporter activity;				IPR011030; IPR015817; IPR015818; IPR001747; IPR015819; IPR015816; IPR016024; IPR015255; IPR009454;	Vitellinogen, superhelical; Vitellinogen, open beta-sheet, subdomain 1; Vitellinogen, open beta-sheet, subdomain 2; Lipid transport protein, N-terminal; Lipid transport protein, beta-sheet shell; Vitellinogen, beta-sheet N-terminal; Armadillo-type fold; Vitellinogen, open beta-sheet; Lipid transport, open beta-sheet;
Q6NWF6	krt8	Lamin B2	_K(cr)LLEGEEDR_	417	1	IEIATYRKLLEGEED	.......K.L.....	mitochondria		intracellular organelle; intracellular non-membrane-bounded organelle; cytoskeletal part; intracellular organelle part; cytoplasm; nuclear lumen; macromolecular complex; intracellular part; cell; organelle part; keratin filament; non-membrane-bounded organelle; membrane-enclosed lumen; intracellular membrane-bounded organelle; organelle; cytoskeleton; nucleoplasm; nucleus; intracellular organelle lumen; intermediate filament cytoskeleton; intracellular; cell part; nuclear matrix; intermediate filament; nuclear periphery; membrane-bounded organelle; protein complex; organelle lumen; nuclear part;	structural molecule activity;	K07605				
F1QIR4	-	Uncharacterized protein	_K(cr)LSEKDEEMEQAK_	1578	1	VKADIERKLSEKDEE		cytosol	cellular component biogenesis; muscle cell development; single-multicellular organism process; cellular component organization; muscle system process; cellular developmental process; cellular component morphogenesis; single-organism process; actin filament organization; cell development; anatomical structure morphogenesis; cell differentiation; actin cytoskeleton organization; muscle contraction; cellular component assembly; organelle assembly; macromolecular complex assembly; myofibril assembly; musculoskeletal movement; slow-twitch skeletal muscle fiber contraction; muscle structure development; cytoskeleton organization; anatomical structure formation involved in morphogenesis; cellular process; striated muscle myosin thick filament assembly; protein complex assembly; cellular protein complex assembly; skeletal muscle thin filament assembly; developmental process; cellular macromolecular complex assembly; striated muscle cell differentiation; actomyosin structure organization; system process; protein complex subunit organization; multicellular organismal movement; single-organism cellular process; cellular component assembly involved in morphogenesis; striated muscle contraction; myosin filament	intracellular organelle; intracellular non-membrane-bounded organelle; cytoskeletal part; intracellular organelle part; macromolecular complex; intracellular part; cell; actin cytoskeleton; organelle part; non-membrane-bounded organelle; organelle; cytoskeleton; cell part; intracellular; myosin complex; protein complex;	ATP binding; motor activity; nucleoside phosphate binding; nucleoside binding; ion binding; hydrolase activity, acting on acid anhydrides; hydrolase activity; nucleotide binding; nucleoside-triphosphatase activity; organic cyclic compound binding; heterocyclic compound binding; ribonucleoside binding; carbohydrate derivative binding; pyrophosphatase activity; catalytic activity; ribonucleotide binding; binding; purine ribonucleoside binding; anion binding; purine ribonucleotide binding; adenyl ribonucleotide binding; adenyl nucleotide binding; purine nucleoside binding; hydrolase activity, acting on acid anhydrides, in phosphorus-containing anhydrides; purine nucleotide binding; small molecule binding; purine ribonucleoside triphosphate binding;	K10352	MYH; myosin heavy chain	dre04530 Tight junction - Danio rerio (zebrafish);	IPR002928; IPR004009; IPR027417; IPR001609; IPR027401;	Myosin tail; Myosin, N-terminal, SH3-like; P-loop containing nucleoside triphosphate hydrolase; Myosin head, motor domain; Myosin-like IQ motif-containing domain;

Q6IQD7	tpm2	Uncharacterized protein	_K(cr)LVILEGELER_	168	1	KYEEVARKLVILEGE	.......K.V.....	cytosol				K10374	TPM2; tropomyosin 2	dre04260 Cardiac muscle contraction - Danio rerio (zebrafish); dre04261 Adrenergic signaling in cardiomyocytes - Danio rerio (zebrafish);		
P13104	tpma	Uncharacterized protein	_K(cr)LVIVEGELER_	168	1	KYEEVARKLVIVEGE	.......K.V.....	cytosol		cytoskeleton; organelle; intracellular organelle; intracellular non-membrane-bounded organelle; cell part; intracellular; cytoplasm; intracellular part; cell; non-membrane-bounded organelle;		K10373	TPM1; tropomyosin 1	dre04260 Cardiac muscle contraction - Danio rerio (zebrafish); dre04261 Adrenergic signaling in cardiomyocytes - Danio rerio (zebrafish);		
E7FAQ8	hnrnpa1a	Uncharacterized protein	_K(cr)MFVGGIK_	107	1	GAHSTVKKMFVGGIK		nuclear			organic cyclic compound binding; heterocyclic compound binding; nucleoside phosphate binding; binding; nucleic acid binding; nucleotide binding; small molecule binding;	K12741	HNRNPA1_3; heterogeneous nuclear ribonucleoprotein A1/A3	dre03040 Spliceosome - Danio rerio (zebrafish);	IPR000504; IPR012677;	RNA recognition motif domain; Nucleotide-binding, alpha-beta plait;
B8A561	myhz1.2	Uncharacterized protein	_K(cr)NLEVTVK_	1786	1	AHLERMKKNLEVTVK	.......K.L.....	nuclear		intracellular organelle; intracellular non-membrane-bounded organelle; cytoskeletal part; intracellular organelle part; macromolecular complex; intracellular part; cell; actin cytoskeleton; organelle part; non-membrane-bounded organelle; organelle; cytoskeleton; cell part; intracellular; myosin complex; protein complex;	ATP binding; motor activity; nucleoside phosphate binding; nucleoside binding; ion binding; hydrolase activity, acting on acid anhydrides; hydrolase activity; nucleotide binding; nucleoside-triphosphatase activity; organic cyclic compound binding; heterocyclic compound binding; ribonucleoside binding; carbohydrate derivative binding; pyrophosphatase activity; catalytic activity; ribonucleotide binding; binding; purine ribonucleoside binding; anion binding; purine ribonucleotide binding; adenyl ribonucleotide binding; adenyl nucleotide binding; purine nucleoside binding; hydrolase activity, acting on acid anhydrides, in phosphorus-containing anhydrides; purine nucleotide binding; small molecule binding; purine ribonucleoside triphosphate binding;	K10352	MYH; myosin heavy chain	dre04530 Tight junction - Danio rerio (zebrafish);	IPR002928; IPR004009; IPR001609; IPR027417; IPR027401;	Myosin tail; Myosin, N-terminal, SH3-like; Myosin head, motor domain; P-loop containing nucleoside triphosphate hydrolase; Myosin-like IQ motif-containing domain;
B8A568	myhz1.1	Uncharacterized protein	_K(cr)QADSVAELGEQIDNLQR_	1197	1	TAAALRKKQADSVAE		nuclear		intracellular organelle; intracellular non-membrane-bounded organelle; cytoskeletal part; intracellular organelle part; macromolecular complex; intracellular part; cell; actin cytoskeleton; organelle part; non-membrane-bounded organelle; organelle; cytoskeleton; cell part; intracellular; myosin complex; protein complex;	ATP binding; motor activity; nucleoside phosphate binding; nucleoside binding; ion binding; hydrolase activity, acting on acid anhydrides; hydrolase activity; nucleotide binding; nucleoside-triphosphatase activity; organic cyclic compound binding; heterocyclic compound binding; ribonucleoside binding; carbohydrate derivative binding; pyrophosphatase activity; catalytic activity; ribonucleotide binding; binding; purine ribonucleoside binding; anion binding; purine ribonucleotide binding; adenyl ribonucleotide binding; adenyl nucleotide binding; purine nucleoside binding; hydrolase activity, acting on acid anhydrides, in phosphorus-containing anhydrides; purine nucleotide binding; small molecule binding; purine ribonucleoside triphosphate binding;	K10352	MYH; myosin heavy chain	dre04530 Tight junction - Danio rerio (zebrafish);	IPR002928; IPR004009; IPR001609; IPR027417; IPR027401;	Myosin tail; Myosin, N-terminal, SH3-like; Myosin head, motor domain; P-loop containing nucleoside triphosphate hydrolase; Myosin-like IQ motif-containing domain;
B8A561	myhz1.2	Uncharacterized protein	_K(cr)QADSVAELGEQIDNLQR_	1197	1	TAAALRKKQADSVAE		nuclear		intracellular organelle; intracellular non-membrane-bounded organelle; cytoskeletal part; intracellular organelle part; macromolecular complex; intracellular part; cell; actin cytoskeleton; organelle part; non-membrane-bounded organelle; organelle; cytoskeleton; cell part; intracellular; myosin complex; protein complex;	ATP binding; motor activity; nucleoside phosphate binding; nucleoside binding; ion binding; hydrolase activity, acting on acid anhydrides; hydrolase activity; nucleotide binding; nucleoside-triphosphatase activity; organic cyclic compound binding; heterocyclic compound binding; ribonucleoside binding; carbohydrate derivative binding; pyrophosphatase activity; catalytic activity; ribonucleotide binding; binding; purine ribonucleoside binding; anion binding; purine ribonucleotide binding; adenyl ribonucleotide binding; adenyl nucleotide binding; purine nucleoside binding; hydrolase activity, acting on acid anhydrides, in phosphorus-containing anhydrides; purine nucleotide binding; small molecule binding; purine ribonucleoside triphosphate binding;	K10352	MYH; myosin heavy chain	dre04530 Tight junction - Danio rerio (zebrafish);	IPR002928; IPR004009; IPR001609; IPR027417; IPR027401;	Myosin tail; Myosin, N-terminal, SH3-like; Myosin head, motor domain; P-loop containing nucleoside triphosphate hydrolase; Myosin-like IQ motif-containing domain;
F1QIR4	-	Uncharacterized protein	_K(cr)QLEQEK_	1534	1	HELEKIRKQLEQEKA	.......K.L.....	cytosol	cellular component biogenesis; muscle cell development; single-multicellular organism process; cellular component organization; muscle system process; cellular developmental process; cellular component morphogenesis; single-organism process; actin filament organization; cell development; anatomical structure morphogenesis; cell differentiation; actin cytoskeleton organization; muscle contraction; cellular component assembly; organelle assembly; macromolecular complex assembly; myofibril assembly; musculoskeletal movement; slow-twitch skeletal muscle fiber contraction; muscle structure development; cytoskeleton organization; anatomical structure formation involved in morphogenesis; cellular process; striated muscle myosin thick filament assembly; protein complex assembly; cellular protein complex assembly; skeletal muscle thin filament assembly; developmental process; cellular macromolecular complex assembly; striated muscle cell differentiation; actomyosin structure organization; system process; protein complex subunit organization; multicellular organismal movement; single-organism cellular process; cellular component assembly involved in morphogenesis; striated muscle contraction; myosin filament	intracellular organelle; intracellular non-membrane-bounded organelle; cytoskeletal part; intracellular organelle part; macromolecular complex; intracellular part; cell; actin cytoskeleton; organelle part; non-membrane-bounded organelle; organelle; cytoskeleton; cell part; intracellular; myosin complex; protein complex;	ATP binding; motor activity; nucleoside phosphate binding; nucleoside binding; ion binding; hydrolase activity, acting on acid anhydrides; hydrolase activity; nucleotide binding; nucleoside-triphosphatase activity; organic cyclic compound binding; heterocyclic compound binding; ribonucleoside binding; carbohydrate derivative binding; pyrophosphatase activity; catalytic activity; ribonucleotide binding; binding; purine ribonucleoside binding; anion binding; purine ribonucleotide binding; adenyl ribonucleotide binding; adenyl nucleotide binding; purine nucleoside binding; hydrolase activity, acting on acid anhydrides, in phosphorus-containing anhydrides; purine nucleotide binding; small molecule binding; purine ribonucleoside triphosphate binding;	K10352	MYH; myosin heavy chain	dre04530 Tight junction - Danio rerio (zebrafish);	IPR002928; IPR004009; IPR027417; IPR001609; IPR027401;	Myosin tail; Myosin, N-terminal, SH3-like; P-loop containing nucleoside triphosphate hydrolase; Myosin head, motor domain; Myosin-like IQ motif-containing domain;
B3DFN3	lmnb2	Lamin B2	_K(cr)SMFDEEVR_	203	1	TEELEFRKSMFDEEV	..E....K......	nuclear		intracellular organelle; intracellular non-membrane-bounded organelle; cytoskeletal part; intracellular organelle part; macromolecular complex; intracellular part; cell; organelle part; non-membrane-bounded organelle; organelle; cytoskeleton; intermediate filament cytoskeleton; cell part; intracellular; intermediate filament; protein complex;	structural molecule activity;	K07611			IPR001322;	Lamin Tail Domain;

Q8JHI0	slc25a5	Solute carrier family 25 alpha, member 5	_K(cr)VFLDGVDK_	97	1	AFKDKYKKVFLDGVD		cytosol	transport; transmembrane transport; single-organism localization; single-organism cellular process; single-organism process; localization; cellular process; single-organism transport; establishment of localization;	intracellular organelle; membrane; mitochondrial part; organelle membrane; organelle envelope; intracellular organelle part; cytoplasm; intrinsic component of membrane; intracellular part; cell; integral component of membrane; organelle part; mitochondrion; organelle; intracellular membrane-bounded organelle; mitochondrial envelope; intracellular; cell part; membrane part; envelope; organelle inner membrane; membrane-bounded organelle; mitochondrial inner membrane; mitochondrial membrane; cytoplasmic part;	transporter activity;	K05863	SLC25A4S; solute carrier family 25 (mitochondrial adenine nucleotide translocator), member 4/5/6/31	dre04020 Calcium signaling pathway - Danio rerio (zebrafish);	IPR023395;	Mitochondrial carrier domain;
Q7ZTS4	krt18	Keratin, type I cytoskeletal 18	_K(cr)VIDDTNLNR_	191	1	ADITGLRKVIDDTNL	.......K.I.....	mitochondria		intracellular organelle; intracellular non-membrane-bounded organelle; cytoskeletal part; intracellular organelle part; macromolecular complex; intracellular part; cell; organelle part; non-membrane-bounded organelle; organelle; cytoskeleton; intermediate filament cytoskeleton; cell part; intracellular; intermediate filament; protein complex;	structural molecule activity;	K07604				
E9QDY3	cyt1	Uncharacterized protein	_K(cr)VLDELTM(ox)TR_	211	1	ADIVGLRKVLDELTM	.......K.L.....	mitochondria		intracellular organelle; intracellular non-membrane-bounded organelle; cytoskeletal part; intracellular organelle part; macromolecular complex; intracellular part; cell; organelle part; non-membrane-bounded organelle; organelle; cytoskeleton; intermediate filament cytoskeleton; cell part; intracellular; intermediate filament; protein complex;	structural molecule activity;	K07604				
E9QDY3	cyt1	Uncharacterized protein	_K(cr)VLDELTMTR_	211	1	ADIVGLRKVLDELTM	.......K.L.....	mitochondria		intracellular organelle; intracellular non-membrane-bounded organelle; cytoskeletal part; intracellular organelle part; macromolecular complex; intracellular part; cell; organelle part; non-membrane-bounded organelle; organelle; cytoskeleton; intermediate filament cytoskeleton; cell part; intracellular; intermediate filament; protein complex;	structural molecule activity;	K07604				
P13104	tpma	Uncharacterized protein	_K(cr)YEEVAR_	161	1	IAEEADRKYEEVARK	..E....K.......	cytosol		cytoskeleton; organelle; intracellular organelle; intracellular non-membrane-bounded organelle; cell part; intracellular; cytoplasm; intracellular part; cell; non-membrane-bounded organelle;		K10373	TPM1; tropomyosin 1	dre04260 Cardiac muscle contraction - Danio rerio (zebrafish); dre04261 Adrenergic signaling in cardiomyocytes - Danio rerio [illegible]		
Q6IQX1	myhz2	Myosin, heavy polypeptide 2, fast muscle specific	_KK(cr)DFETAQLLSK_	1085	1	SEEKIKKKDFETAQL	..E....K.......	cytosol	response to stress; response to stimulus;	intracellular organelle; intracellular non-membrane-bounded organelle; cytoskeletal part; intracellular organelle part; macromolecular complex; intracellular part; cell; actin cytoskeleton; organelle part; non-membrane-bounded organelle; organelle; cytoskeleton; cell part; intracellular; myosin complex; protein complex;	ATP binding; motor activity; nucleoside phosphate binding; nucleoside binding; ion binding; hydrolase activity, acting on acid anhydrides; hydrolase activity; nucleotide binding; nucleoside-triphosphatase activity; organic cyclic compound binding; heterocyclic compound binding; ribonucleoside binding; carbohydrate derivative binding; pyrophosphatase activity; catalytic activity; ribonucleotide binding; binding; purine ribonucleoside binding; anion binding; purine ribonucleotide binding; adenyl ribonucleotide binding; adenyl nucleotide binding; purine nucleoside binding; hydrolase activity, acting on acid anhydrides, in phosphorus-containing anhydrides; purine nucleotide binding; small molecule binding; purine ribonucleoside triphosphate binding;	K10352	MYH; myosin heavy chain	dre04530 Tight junction - Danio rerio (zebrafish);	IPR002928; IPR004009; IPR001609; IPR027417; IPR027401;	Myosin tail; Myosin, N-terminal, SH3-like; Myosin head, motor domain; P-loop containing nucleoside triphosphate hydrolase; Myosin-like IQ motif-containing domain;
B8A568	myhz1.1	Uncharacterized protein	_KK(cr)ELEEK_	873	1	AKAEAKKKELEEKMV	.......K.L.....	nuclear		intracellular organelle; intracellular non-membrane-bounded organelle; cytoskeletal part; intracellular organelle part; macromolecular complex; intracellular part; cell; actin cytoskeleton; organelle part; non-membrane-bounded organelle; organelle; cytoskeleton; cell part; intracellular; myosin complex; protein complex;	ATP binding; motor activity; nucleoside phosphate binding; nucleoside binding; ion binding; hydrolase activity, acting on acid anhydrides; hydrolase activity; nucleotide binding; nucleoside-triphosphatase activity; organic cyclic compound binding; heterocyclic compound binding; ribonucleoside binding; carbohydrate derivative binding; pyrophosphatase activity; catalytic activity; ribonucleotide binding; binding; purine ribonucleoside binding; anion binding; purine ribonucleotide binding; adenyl ribonucleotide binding; adenyl nucleotide binding; purine nucleoside binding; hydrolase activity, acting on acid anhydrides, in phosphorus-containing anhydrides; purine nucleotide binding; small molecule binding; purine ribonucleoside triphosphate binding;	K10352	MYH; myosin heavy chain	dre04530 Tight junction - Danio rerio (zebrafish);	IPR002928; IPR004009; IPR001609; IPR027417; IPR027401;	Myosin tail; Myosin, N-terminal, SH3-like; Myosin head, motor domain; P-loop containing nucleoside triphosphate hydrolase; Myosin-like IQ motif-containing domain;
B8A561	myhz1.2	Uncharacterized protein	_KK(cr)ELEEK_	873	1	AKAEAKKKELEEKMV	.......K.L.....	nuclear		intracellular organelle; intracellular non-membrane-bounded organelle; cytoskeletal part; intracellular organelle part; macromolecular complex; intracellular part; cell; actin cytoskeleton; organelle part; non-membrane-bounded organelle; organelle; cytoskeleton; cell part; intracellular; myosin complex; protein complex;	ATP binding; motor activity; nucleoside phosphate binding; nucleoside binding; ion binding; hydrolase activity, acting on acid anhydrides; hydrolase activity; nucleotide binding; nucleoside-triphosphatase activity; organic cyclic compound binding; heterocyclic compound binding; ribonucleoside binding; carbohydrate derivative binding; pyrophosphatase activity; catalytic activity; ribonucleotide binding; binding; purine ribonucleoside binding; anion binding; purine ribonucleotide binding; adenyl ribonucleotide binding; adenyl nucleotide binding; purine nucleoside binding; hydrolase activity, acting on acid anhydrides, in phosphorus-containing anhydrides; purine nucleotide binding; small molecule binding; purine ribonucleoside triphosphate binding;	K10352	MYH; myosin heavy chain	dre04530 Tight junction - Danio rerio (zebrafish);	IPR002928; IPR004009; IPR001609; IPR027417; IPR027401;	Myosin tail; Myosin, N-terminal, SH3-like; Myosin head, motor domain; P-loop containing nucleoside triphosphate hydrolase; Myosin-like IQ motif-containing domain;
E7EXP0	tnnt3b	Uncharacterized protein	KKEEEELIALK(cr)DR	72	1	EEELIALKDRIEKRR	..E....K.......	nuclear				K12046				

Q92005	eef1a	Elongation factor 1-alpha	_KLEDNPK(cr)ALK_	392	1	KKLEDNPKALKSGDA	.......K.L.....	cytosol		intracellular part; cell; cell part; intracellular; cytoplasm;	nucleoside phosphate binding; translation elongation factor activity; hydrolase activity, acting on acid anhydrides; hydrolase activity; organic cyclic compound binding; heterocyclic compound binding; pyrophosphatase activity; catalytic activity; ribonucleotide binding; binding; GTPase activity; purine ribonucleoside binding; anion binding; hydrolase activity, acting on acid anhydrides, in phosphorus-containing anhydrides; guanyl nucleotide binding; GTP binding; purine nucleotide binding; purine ribonucleoside triphosphate binding; RNA binding; ion binding; nucleoside binding; nucleotide binding; nucleoside-triphosphatase activity; ribonucleoside binding; translation factor activity, nucleic acid binding; carbohydrate derivative binding; nucleic acid binding; purine ribonucleotide binding; guanyl ribonucleotide binding; purine nucleoside binding; small molecule binding;	K03231	EEF1A; elongation factor 1-alpha	dre03013 RNA transport - Danio rerio (zebrafish);	IPR004160; IPR004161; IPR009001; IPR027417; IPR000795; IPR009000;	Translation elongation factor EFTu/EF1A, C-terminal; Translation elongation factor EFTu/EF1A, domain 2; Translation elongation factor EF1A/initiation factor IF2gamma, C-terminal; P-loop containing nucleoside triphosphate hydrolase; Elongation factor, GTP-binding domain; Translation protein, beta-barrel domain;
F1QIR4	-	Uncharacterized protein	_KLSEK(cr)DEEM(ox)EQAK_	1582	1	IERKLSEKDEEMEQA	.......K.E.....	cytosol	cellular component biogenesis; muscle cell development; single-multicellular organism process; cellular component organization; muscle system process; cellular developmental process; cellular component morphogenesis; single-organism process; actin filament organization; cell development; anatomical structure morphogenesis; cell differentiation; actin cytoskeleton organization; muscle contraction; cellular component assembly; organelle assembly; macromolecular complex assembly; myofibril assembly; musculoskeletal movement; slow-twitch skeletal muscle fiber contraction; muscle structure development; cytoskeleton organization; anatomical structure formation involved in morphogenesis; cellular process; striated muscle myosin thick filament assembly; protein complex assembly; cellular protein complex assembly; skeletal muscle thin filament assembly; developmental process; cellular macromolecular complex assembly; striated muscle cell differentiation; actomyosin structure organization; system process; protein complex subunit organization; multicellular organismal movement; single-organism cellular process; cellular component assembly involved in morphogenesis; striated muscle contraction; myosin filament	intracellular organelle; intracellular non-membrane-bounded organelle; cytoskeletal part; intracellular organelle part; macromolecular complex; intracellular part; cell; actin cytoskeleton; organelle part; non-membrane-bounded organelle; organelle; cytoskeleton; cell part; intracellular; myosin complex; protein complex;	ATP binding; motor activity; nucleoside phosphate binding; nucleoside binding; ion binding; hydrolase activity, acting on acid anhydrides; hydrolase activity; nucleotide binding; nucleoside-triphosphatase activity; organic cyclic compound binding; heterocyclic compound binding; ribonucleoside binding; carbohydrate derivative binding; pyrophosphatase activity; catalytic activity; ribonucleotide binding; binding; purine ribonucleoside binding; anion binding; purine ribonucleotide binding; adenyl ribonucleotide binding; adenyl nucleotide binding; purine nucleoside binding; hydrolase activity, acting on acid anhydrides, in phosphorus-containing anhydrides; purine nucleotide binding; small molecule binding; purine ribonucleoside triphosphate binding;	K10352	MYH; myosin heavy chain	dre04530 Tight junction - Danio rerio (zebrafish);	IPR002928; IPR004009; IPR027417; IPR001609; IPR027401;	Myosin tail; Myosin, N-terminal, SH3-like; P-loop containing nucleoside triphosphate hydrolase; Myosin head, motor domain; Myosin-like IQ motif-containing domain;
F1QIR4	-	Uncharacterized protein	_KLSEK(cr)DEEMEQAK_	1582	1	IERKLSEKDEEMEQA	.......K.E.....	cytosol	cellular component biogenesis; muscle cell development; single-multicellular organism process; cellular component organization; muscle system process; cellular developmental process; cellular component morphogenesis; single-organism process; actin filament organization; cell development; anatomical structure morphogenesis; cell differentiation; actin cytoskeleton organization; muscle contraction; cellular component assembly; organelle assembly; macromolecular complex assembly; myofibril assembly; musculoskeletal movement; slow-twitch skeletal muscle fiber contraction; muscle structure development; cytoskeleton organization; anatomical structure formation involved in morphogenesis; cellular process; striated muscle myosin thick filament assembly; protein complex assembly; cellular protein complex assembly; skeletal muscle thin filament assembly; developmental process; cellular macromolecular complex assembly; striated muscle cell differentiation; actomyosin structure organization; system process; protein complex subunit organization; multicellular organismal movement; single-organism cellular process; cellular component assembly involved in morphogenesis; striated muscle contraction; myosin filament	intracellular organelle; intracellular non-membrane-bounded organelle; cytoskeletal part; intracellular organelle part; macromolecular complex; intracellular part; cell; actin cytoskeleton; organelle part; non-membrane-bounded organelle; organelle; cytoskeleton; cell part; intracellular; myosin complex; protein complex;	ATP binding; motor activity; nucleoside phosphate binding; nucleoside binding; ion binding; hydrolase activity, acting on acid anhydrides; hydrolase activity; nucleotide binding; nucleoside-triphosphatase activity; organic cyclic compound binding; heterocyclic compound binding; ribonucleoside binding; carbohydrate derivative binding; pyrophosphatase activity; catalytic activity; ribonucleotide binding; binding; purine ribonucleoside binding; anion binding; purine ribonucleotide binding; adenyl ribonucleotide binding; adenyl nucleotide binding; purine nucleoside binding; hydrolase activity, acting on acid anhydrides, in phosphorus-containing anhydrides; purine nucleotide binding; small molecule binding; purine ribonucleoside triphosphate binding;	K10352	MYH; myosin heavy chain	dre04530 Tight junction - Danio rerio (zebrafish);	IPR002928; IPR004009; IPR027417; IPR001609; IPR027401;	Myosin tail; Myosin, N-terminal, SH3-like; P-loop containing nucleoside triphosphate hydrolase; Myosin head, motor domain; Myosin-like IQ motif-containing domain;
Q8JHI0	slc25a5	Solute carrier family 25 alpha, member 5	_LAADVGK(cr)AGAER_	147	1	RLAADVGKAGAEREF		cytosol	transport; transmembrane transport; single-organism localization; single-organism cellular process; single-organism process; localization; cellular process; single-organism transport; establishment of localization;	intracellular organelle; membrane; mitochondrial part; organelle membrane; organelle envelope; intracellular organelle part; cytoplasm; intrinsic component of membrane; intracellular part; cell; integral component of membrane; organelle part; mitochondrion; organelle; intracellular membrane-bounded organelle; mitochondrial envelope; intracellular; cell part; membrane part; envelope; organelle inner membrane; membrane-bounded organelle; mitochondrial inner membrane; mitochondrial membrane; cytoplasmic part;	transporter activity;	K05863	SLC25A4S; solute carrier family 25 (mitochondrial adenine nucleotide translocator), member 4/5/6/31	dre04020 Calcium signaling pathway - Danio rerio (zebrafish);	IPR023395;	Mitochondrial carrier domain;

B8A568	myhz1.1	Uncharacterized protein	_LAEK(cr)DEEIEQIKR_	1581	1	IDRKLAEKDEEIEQI	.......K.E.....	nuclear		intracellular organelle; intracellular non-membrane-bounded organelle; cytoskeletal part; intracellular organelle part; macromolecular complex; intracellular part; cell; actin cytoskeleton; organelle part; non-membrane-bounded organelle; organelle; cytoskeleton; cell part; intracellular; myosin complex; protein complex;	ATP binding; motor activity; nucleoside phosphate binding; nucleoside binding; ion binding; hydrolase activity, acting on acid anhydrides; hydrolase activity; nucleotide binding; nucleoside-triphosphatase activity; organic cyclic compound binding; heterocyclic compound binding; ribonucleoside binding; carbohydrate derivative binding; pyrophosphatase activity; catalytic activity; ribonucleotide binding; binding; purine ribonucleoside binding; anion binding; purine ribonucleotide binding; adenyl ribonucleotide binding; adenyl nucleotide binding; purine nucleoside binding; hydrolase activity, acting on acid anhydrides, in phosphorus-containing anhydrides; purine nucleotide binding; small molecule binding; purine ribonucleoside triphosphate binding;	K10352	MYH; myosin heavy chain	dre04530 Tight junction - Danio rerio (zebrafish);	IPR002928; IPR004009; IPR001609; IPR027417; IPR027401;	Myosin tail; Myosin, N-terminal, SH3-like; Myosin head, motor domain; P-loop containing nucleoside triphosphate hydrolase; Myosin-like IQ motif-containing domain;
Q6IQX1	myhz2	Myosin, heavy polypeptide 2, fast muscle specific	_LAEK(cr)DEEMEQIK_	1581	1	IDRKLAEKDEEMEQI	.......K.E.....	cytosol	response to stress; response to stimulus;	intracellular organelle; intracellular non-membrane-bounded organelle; cytoskeletal part; intracellular organelle part; macromolecular complex; intracellular part; cell; actin cytoskeleton; organelle part; non-membrane-bounded organelle; organelle; cytoskeleton; cell part; intracellular; myosin complex; protein complex;	ATP binding; motor activity; nucleoside phosphate binding; nucleoside binding; ion binding; hydrolase activity, acting on acid anhydrides; hydrolase activity; nucleotide binding; nucleoside-triphosphatase activity; organic cyclic compound binding; heterocyclic compound binding; ribonucleoside binding; carbohydrate derivative binding; pyrophosphatase activity; catalytic activity; ribonucleotide binding; binding; purine ribonucleoside binding; anion binding; purine ribonucleotide binding; adenyl ribonucleotide binding; adenyl nucleotide binding; purine nucleoside binding; hydrolase activity, acting on acid anhydrides, in phosphorus-containing anhydrides; purine nucleotide binding; small molecule binding; purine ribonucleoside triphosphate binding;	K10352	MYH; myosin heavy chain	dre04530 Tight junction - Danio rerio (zebrafish);	IPR002928; IPR004009; IPR001609; IPR027417; IPR027401;	Myosin tail; Myosin, N-terminal, SH3-like; Myosin head, motor domain; P-loop containing nucleoside triphosphate hydrolase; Myosin-like IQ motif-containing domain;
B8A568	myhz1.1	Uncharacterized protein	_LAEKDEEIEQIK(cr)R_	1589	1	DEEIEQIKRNSQRIT	..E....K.......	nuclear		intracellular organelle; intracellular non-membrane-bounded organelle; cytoskeletal part; intracellular organelle part; macromolecular complex; intracellular part; cell; actin cytoskeleton; organelle part; non-membrane-bounded organelle; organelle; cytoskeleton; cell part; intracellular; myosin complex; protein complex;	ATP binding; motor activity; nucleoside phosphate binding; nucleoside binding; ion binding; hydrolase activity, acting on acid anhydrides; hydrolase activity; nucleotide binding; nucleoside-triphosphatase activity; organic cyclic compound binding; heterocyclic compound binding; ribonucleoside binding; carbohydrate derivative binding; pyrophosphatase activity; catalytic activity; ribonucleotide binding; binding; purine ribonucleoside binding; anion binding; purine ribonucleotide binding; adenyl ribonucleotide binding; adenyl nucleotide binding; purine nucleoside binding; hydrolase activity, acting on acid anhydrides, in phosphorus-containing anhydrides; purine nucleotide binding; small molecule binding; purine ribonucleoside triphosphate binding;	K10352	MYH; myosin heavy chain	dre04530 Tight junction - Danio rerio (zebrafish);	IPR002928; IPR004009; IPR001609; IPR027417; IPR027401;	Myosin tail; Myosin, N-terminal, SH3-like; Myosin head, motor domain; P-loop containing nucleoside triphosphate hydrolase; Myosin-like IQ motif-containing domain;
Q6IQX1	myhz2	Myosin, heavy polypeptide 2, fast muscle specific	_LAEKDEEMEQIK(cr)R_	1589	1	DEEMEQIKRNSQRVT	..E....K.......	cytosol	response to stress; response to stimulus;	intracellular organelle; intracellular non-membrane-bounded organelle; cytoskeletal part; intracellular organelle part; macromolecular complex; intracellular part; cell; actin cytoskeleton; organelle part; non-membrane-bounded organelle; organelle; cytoskeleton; cell part; intracellular; myosin complex; protein complex;	ATP binding; motor activity; nucleoside phosphate binding; nucleoside binding; ion binding; hydrolase activity, acting on acid anhydrides; hydrolase activity; nucleotide binding; nucleoside-triphosphatase activity; organic cyclic compound binding; heterocyclic compound binding; ribonucleoside binding; carbohydrate derivative binding; pyrophosphatase activity; catalytic activity; ribonucleotide binding; binding; purine ribonucleoside binding; anion binding; purine ribonucleotide binding; adenyl ribonucleotide binding; adenyl nucleotide binding; purine nucleoside binding; hydrolase activity, acting on acid anhydrides, in phosphorus-containing anhydrides; purine nucleotide binding; small molecule binding; purine ribonucleoside triphosphate binding;	K10352	MYH; myosin heavy chain	dre04530 Tight junction - Danio rerio (zebrafish);	IPR002928; IPR004009; IPR001609; IPR027417; IPR027401;	Myosin tail; Myosin, N-terminal, SH3-like; Myosin head, motor domain; P-loop containing nucleoside triphosphate hydrolase; Myosin-like IQ motif-containing domain;
E7F225	zgc:163061	Uncharacterized protein	_LAIK(cr)GMVTK_	77	1	SRVKLAIKGMVTKGV		nuclear	cellular component biogenesis; cellular component organization; nucleosome organization; cellular macromolecular complex assembly; chromatin assembly or disassembly; protein complex subunit organization; macromolecular complex subunit organization; protein-DNA complex assembly; DNA packaging; nucleosome assembly; chromosome organization; cellular component assembly; macromolecular complex assembly; organelle organization; chromatin organization; protein complex biogenesis; chromatin assembly; cellular process; protein complex assembly; cellular component organization or biogenesis; protein-DNA complex subunit organization; DNA conformation change;	intracellular organelle; intracellular non-membrane-bounded organelle; intracellular organelle part; macromolecular complex; intracellular part; cell; organelle part; DNA bending complex; chromosomal part; nucleosome; non-membrane-bounded organelle; intracellular membrane-bounded organelle; organelle; nucleus; chromatin; cell part; intracellular; chromosome; membrane-bounded organelle; protein complex; protein-DNA complex; DNA packaging complex;	organic cyclic compound binding; heterocyclic compound binding; DNA binding; binding; nucleic acid binding;	K11275			IPR011991; IPR005818;	Winged helix-turn-helix DNA-binding domain; Linker histone H1/H5, domain H15;
F1QA12	-	Uncharacterized protein (Fragment)	_LAK(cr)EQAEK_	539	1	AEKERLAKEQAEKEK		extracellular		membrane;					IPR007943;	Aspartyl beta-hydroxylase/Triadin domain;
B8A568	myhz1.1	Uncharacterized protein	_LAQESIMDLENDK(cr)QQSEEK_	1075	1	IMDLENDKQQSEEKL	......DK.......	nuclear		intracellular organelle; intracellular non-membrane-bounded organelle; cytoskeletal part; intracellular organelle part; macromolecular complex; intracellular part; cell; actin cytoskeleton; organelle part; non-membrane-bounded organelle; organelle; cytoskeleton; cell part; intracellular; myosin complex; protein complex;	ATP binding; motor activity; nucleoside phosphate binding; nucleoside binding; ion binding; hydrolase activity, acting on acid anhydrides; hydrolase activity; nucleotide binding; nucleoside-triphosphatase activity; organic cyclic compound binding; heterocyclic compound binding; ribonucleoside binding; carbohydrate derivative binding; pyrophosphatase activity; catalytic activity; ribonucleotide binding; binding; purine ribonucleoside binding; anion binding; purine ribonucleotide binding; adenyl ribonucleotide binding; adenyl nucleotide binding; purine nucleoside binding; hydrolase activity, acting on acid anhydrides, in phosphorus-containing anhydrides; purine nucleotide binding; small molecule binding; purine ribonucleoside triphosphate binding;	K10352	MYH; myosin heavy chain	dre04530 Tight junction - Danio rerio (zebrafish);	IPR002928; IPR004009; IPR001609; IPR027417; IPR027401;	Myosin tail; Myosin, N-terminal, SH3-like; Myosin head, motor domain; P-loop containing nucleoside triphosphate hydrolase; Myosin-like IQ motif-containing domain;
B8A561	myhz1.2	Uncharacterized protein	_LAQESIMDLENDK(cr)QQSEEK_	1075	1	IMDLENDKQQSEEKL	......DK.......	nuclear		intracellular organelle; intracellular non-membrane-bounded organelle; cytoskeletal part; intracellular organelle part; macromolecular complex; intracellular part; cell; actin cytoskeleton; organelle part; non-membrane-bounded organelle; organelle; cytoskeleton; cell part; intracellular; myosin complex; protein complex;	ATP binding; motor activity; nucleoside phosphate binding; nucleoside binding; ion binding; hydrolase activity, acting on acid anhydrides; hydrolase activity; nucleotide binding; nucleoside-triphosphatase activity; organic cyclic compound binding; heterocyclic compound binding; ribonucleoside binding; carbohydrate derivative binding; pyrophosphatase activity; catalytic activity; ribonucleotide binding; binding; purine ribonucleoside binding; anion binding; purine ribonucleotide binding; adenyl ribonucleotide binding; adenyl nucleotide binding; purine nucleoside binding; hydrolase activity, acting on acid anhydrides, in phosphorus-containing anhydrides; purine nucleotide binding; small molecule binding; purine ribonucleoside triphosphate binding;	K10352	MYH; myosin heavy chain	dre04530 Tight junction - Danio rerio (zebrafish);	IPR002928; IPR004009; IPR001609; IPR027417; IPR027401;	Myosin tail; Myosin, N-terminal, SH3-like; Myosin head, motor domain; P-loop containing nucleoside triphosphate hydrolase; Myosin-like IQ motif-containing domain;

P13104	tpma	Uncharacterized protein	_LATALQK(cr)LEEAEK_	112	1	RLATALQKLEEAEKA	.......K.E.....	cytosol		cytoskeleton; organelle; intracellular organelle; intracellular non-membrane-bounded organelle; cell part; intracellular; cytoplasm; intracellular part; cell; non-membrane-bounded organelle;		K10373	TPM1; tropomyosin 1	dre04260 Cardiac muscle contraction - Danio rerio (zebrafish); dre04261 Adrenergic signaling in cardiomyocytes - Danio rerio (zebrafish);		
Q6P3J5	eef2b	Eukaryotic translation elongation factor 2	_LDIK(cr)LDTEDKDK_	322	1	LIEKLDIKLDTEDKD	..E....K.......	cytosol			nucleoside phosphate binding; translation elongation factor activity; hydrolase activity, acting on acid anhydrides; hydrolase activity; organic cyclic compound binding; heterocyclic compound binding; pyrophosphatase activity; catalytic activity; ribonucleotide binding; binding; GTPase activity; purine ribonucleoside binding; anion binding; hydrolase activity, acting on acid anhydrides, in phosphorus-containing anhydrides; guanyl nucleotide binding; GTP binding; purine nucleotide binding; purine ribonucleoside triphosphate binding; RNA binding; ion binding; nucleoside binding; nucleotide binding; nucleoside-triphosphatase activity; ribonucleoside binding; translation factor activity, nucleic acid binding; carbohydrate derivative binding; nucleic acid binding; purine ribonucleotide binding; guanyl ribonucleotide binding; purine nucleoside binding; small molecule binding;	K03234			IPR027417; IPR000795; IPR005517; IPR005225; IPR004161; IPR000640; IPR020568; IPR014721; IPR009022; IPR009000;	P-loop containing nucleoside triphosphate hydrolase; Elongation factor, GTP-binding domain; Translation elongation factor EFG/EF2, domain IV; Small GTP-binding protein domain; Translation elongation factor EFTu/EF1A, domain 2; Translation elongation factor EFG, V domain; Ribosomal protein S5 domain 2-type fold; Ribosomal protein S5 domain 2-type fold, subgroup; Elongation factor G, III-V domain; Translation protein, beta-barrel domain;
B8A568	myhz1.1	Uncharacterized protein	_LEDEEEINAELTAK(cr)K_	941	1	INAELTAKKRKLEDE		nuclear		intracellular organelle; intracellular non-membrane-bounded organelle; cytoskeletal part; intracellular organelle part; macromolecular complex; intracellular part; cell; actin cytoskeleton; organelle part; non-membrane-bounded organelle; organelle; cytoskeleton; cell part; intracellular; myosin complex; protein complex;	ATP binding; motor activity; nucleoside phosphate binding; nucleoside binding; ion binding; hydrolase activity, acting on acid anhydrides; hydrolase activity; nucleotide binding; nucleoside-triphosphatase activity; organic cyclic compound binding; heterocyclic compound binding; ribonucleoside binding; carbohydrate derivative binding; pyrophosphatase activity; catalytic activity; ribonucleotide binding; binding; purine ribonucleoside binding; anion binding; purine ribonucleotide binding; adenyl ribonucleotide binding; adenyl nucleotide binding; purine nucleoside binding; hydrolase activity, acting on acid anhydrides, in phosphorus-containing anhydrides; purine nucleotide binding; small molecule binding; purine ribonucleoside triphosphate binding;	K10352	MYH; myosin heavy chain	dre04530 Tight junction - Danio rerio (zebrafish);	IPR002928; IPR004009; IPR001609; IPR027417; IPR027401;	Myosin tail; Myosin, N-terminal, SH3-like; Myosin head, motor domain; P-loop containing nucleoside triphosphate hydrolase; Myosin-like IQ motif-containing domain;
B8A561	myhz1.2	Uncharacterized protein	_LEDEEEINAELTAK(cr)K_	941	1	INAELTAKKRKLEDE		nuclear		intracellular organelle; intracellular non-membrane-bounded organelle; cytoskeletal part; intracellular organelle part; macromolecular complex; intracellular part; cell; actin cytoskeleton; organelle part; non-membrane-bounded organelle; organelle; cytoskeleton; cell part; intracellular; myosin complex; protein complex;	ATP binding; motor activity; nucleoside phosphate binding; nucleoside binding; ion binding; hydrolase activity, acting on acid anhydrides; hydrolase activity; nucleotide binding; nucleoside-triphosphatase activity; organic cyclic compound binding; heterocyclic compound binding; ribonucleoside binding; carbohydrate derivative binding; pyrophosphatase activity; catalytic activity; ribonucleotide binding; binding; purine ribonucleoside binding; anion binding; purine ribonucleotide binding; adenyl ribonucleotide binding; adenyl nucleotide binding; purine nucleoside binding; hydrolase activity, acting on acid anhydrides, in phosphorus-containing anhydrides; purine nucleotide binding; small molecule binding; purine ribonucleoside triphosphate binding;	K10352	MYH; myosin heavy chain	dre04530 Tight junction - Danio rerio (zebrafish);	IPR002928; IPR004009; IPR001609; IPR027417; IPR027401;	Myosin tail; Myosin, N-terminal, SH3-like; Myosin head, motor domain; P-loop containing nucleoside triphosphate hydrolase; Myosin-like IQ motif-containing domain;
P13104	tpma	Tropomyosin alpha-1 chain	_LEEAEK(cr)AADESER_	118	1	QKLEEAEKAADESER		cytosol		cytoskeleton; organelle; intracellular organelle; intracellular non-membrane-bounded organelle; cell part; intracellular; cytoplasm; intracellular part; cell; non-membrane-bounded organelle;		K10373	TPM1; tropomyosin 1	dre04260 Cardiac muscle contraction - Danio rerio (zebrafish); dre04261 Adrenergic signaling in cardiomyocytes - Danio rerio (zebrafish);		
B8A568	myhz1.1	Uncharacterized protein	_LEEAGGATAAQIEMNK(cr)K_	1167	1	AAQIEMNKKREAEFQ		nuclear		intracellular organelle; intracellular non-membrane-bounded organelle; cytoskeletal part; intracellular organelle part; macromolecular complex; intracellular part; cell; actin cytoskeleton; organelle part; non-membrane-bounded organelle; organelle; cytoskeleton; cell part; intracellular; myosin complex; protein complex;	ATP binding; motor activity; nucleoside phosphate binding; nucleoside binding; ion binding; hydrolase activity, acting on acid anhydrides; hydrolase activity; nucleotide binding; nucleoside-triphosphatase activity; organic cyclic compound binding; heterocyclic compound binding; ribonucleoside binding; carbohydrate derivative binding; pyrophosphatase activity; catalytic activity; ribonucleotide binding; binding; purine ribonucleoside binding; anion binding; purine ribonucleotide binding; adenyl ribonucleotide binding; adenyl nucleotide binding; purine nucleoside binding; hydrolase activity, acting on acid anhydrides, in phosphorus-containing anhydrides; purine nucleotide binding; small molecule binding; purine ribonucleoside triphosphate binding;	K10352	MYH; myosin heavy chain	dre04530 Tight junction - Danio rerio (zebrafish);	IPR002928; IPR004009; IPR001609; IPR027417; IPR027401;	Myosin tail; Myosin, N-terminal, SH3-like; Myosin head, motor domain; P-loop containing nucleoside triphosphate hydrolase; Myosin-like IQ motif-containing domain;
B8A561	myhz1.2	Uncharacterized protein	_LEEAGGATAAQIEMNK(cr)K_	1167	1	AAQIEMNKKREAEFQ		nuclear		intracellular organelle; intracellular non-membrane-bounded organelle; cytoskeletal part; intracellular organelle part; macromolecular complex; intracellular part; cell; actin cytoskeleton; organelle part; non-membrane-bounded organelle; organelle; cytoskeleton; cell part; intracellular; myosin complex; protein complex;	ATP binding; motor activity; nucleoside phosphate binding; nucleoside binding; ion binding; hydrolase activity, acting on acid anhydrides; hydrolase activity; nucleotide binding; nucleoside-triphosphatase activity; organic cyclic compound binding; heterocyclic compound binding; ribonucleoside binding; carbohydrate derivative binding; pyrophosphatase activity; catalytic activity; ribonucleotide binding; binding; purine ribonucleoside binding; anion binding; purine ribonucleotide binding; adenyl ribonucleotide binding; adenyl nucleotide binding; purine nucleoside binding; hydrolase activity, acting on acid anhydrides, in phosphorus-containing anhydrides; purine nucleotide binding; small molecule binding; purine ribonucleoside triphosphate binding;	K10352	MYH; myosin heavy chain	dre04530 Tight junction - Danio rerio (zebrafish);	IPR002928; IPR004009; IPR001609; IPR027417; IPR027401;	Myosin tail; Myosin, N-terminal, SH3-like; Myosin head, motor domain; P-loop containing nucleoside triphosphate hydrolase; Myosin-like IQ motif-containing domain;

Q7ZUI4	zgc:56493	Thioredoxin	_LEEMVK(cr)QHK_	104	1	TKLEEMVKQHKN___		cytosol	regulation of cellular process; regulation of biological quality; small molecule metabolic process; cell redox homeostasis; single-organism cellular process; ether metabolic process; cellular homeostasis; single-organism process; organic substance metabolic process; homeostatic process; metabolic process; regulation of biological process; single-organism metabolic process; glycerol ether metabolic process; cellular process; biological regulation;		protein disulfide oxidoreductase activity; oxidoreductase activity, acting on a sulfur group of donors; disulfide oxidoreductase activity; electron carrier activity; catalytic activity; oxidoreductase activity;	K03671			IPR012336; IPR013766;	Thioredoxin-like fold; Thioredoxin domain;
F1QK60	krt4	Uncharacterized protein	_LEGELK(cr)NMQGLVEDFK_	189	1	MKLEGELKNMQGLVE		nuclear		intracellular organelle; intracellular non-membrane-bounded organelle; cytoskeletal part; intracellular organelle part; macromolecular complex; intracellular part; cell; keratin filament; organelle part; non-membrane-bounded organelle; organelle; cytoskeleton; intermediate filament cytoskeleton; cell part; intracellular; intermediate filament; protein complex;	structural molecule activity;	K07605				
Q29RA2	zgc:136908	Uncharacterized protein	_LEILQIHTK(cr)NMK_	388	1	EILQIHTKNMKLSED		cytosol			ATP binding; nucleoside phosphate binding; nucleoside binding; ion binding; hydrolase activity, acting on acid anhydrides; hydrolase activity; nucleotide binding; nucleoside-triphosphatase activity; organic cyclic compound binding; heterocyclic compound binding; ribonucleoside binding; carbohydrate derivative binding; pyrophosphatase activity; catalytic activity; ribonucleotide binding; binding; purine ribonucleoside binding; anion binding; purine ribonucleotide binding; adenyl ribonucleotide binding; adenyl nucleotide binding; purine nucleoside binding; hydrolase activity, acting on acid anhydrides, in phosphorus-containing anhydrides; purine nucleotide binding; small molecule binding; purine ribonucleoside triphosphate binding;				IPR029067; IPR003959; IPR003593; IPR009010; IPR004201; IPR027417; IPR003338;	CDC48 domain 2-like; ATPase, AAA-type, core; AAA+ ATPase domain; Aspartate decarboxylase-like domain; CDC48, domain 2; P-loop containing nucleoside triphosphate hydrolase; CDC48, N-terminal subdomain;
P13104	tpma	Tropomyosin alpha-1 chain	_LEK(cr)TIDDLEDELYAQK_	251	1	RSVAKLEKTIDDLED	.......K.I.....	cytosol		cytoskeleton; organelle; intracellular organelle; intracellular non-membrane-bounded organelle; cell part; intracellular; cytoplasm; intracellular part; cell; non-membrane-bounded organelle;		K10373	TPM1; tropomyosin 1	dre04260 Cardiac muscle contraction - Danio rerio (zebrafish); dre04261 Adrenergic signaling in cardiomyocytes - Danio rerio [illegible]		
E7FBZ3	zgc:171719	Uncharacterized protein	_LEK(cr)TIDDLEEK_	251	1	RSVAKLEKTIDDLEE	.......K.I.....	cytosol								
B8A568	myhz1.1	Uncharacterized protein	_LEQQVDDLEGSLEQEK(cr)K_	1044	1	EGSLEQEKKLRMDLE	.......K.L.....	nuclear		intracellular organelle; intracellular non-membrane-bounded organelle; cytoskeletal part; intracellular organelle part; macromolecular complex; intracellular part; cell; actin cytoskeleton; organelle part; non-membrane-bounded organelle; organelle; cytoskeleton; cell part; intracellular; myosin complex; protein complex;	ATP binding; motor activity; nucleoside phosphate binding; nucleoside binding; ion binding; hydrolase activity, acting on acid anhydrides; hydrolase activity; nucleotide binding; nucleoside-triphosphatase activity; organic cyclic compound binding; heterocyclic compound binding; ribonucleoside binding; carbohydrate derivative binding; pyrophosphatase activity; catalytic activity; ribonucleotide binding; binding; purine ribonucleoside binding; anion binding; purine ribonucleotide binding; adenyl ribonucleotide binding; adenyl nucleotide binding; purine nucleoside binding; hydrolase activity, acting on acid anhydrides, in phosphorus-containing anhydrides; purine nucleotide binding; small molecule binding; purine ribonucleoside triphosphate binding;	K10352	MYH; myosin heavy chain	dre04530 Tight junction - Danio rerio (zebrafish);	IPR002928; IPR004009; IPR001609; IPR027417; IPR027401;	Myosin tail; Myosin, N-terminal, SH3-like; Myosin head, motor domain; P-loop containing nucleoside triphosphate hydrolase; Myosin-like IQ motif-containing domain;
B8A561	myhz1.2	Uncharacterized protein	_LEQQVDDLEGSLEQEK(cr)K_	1044	1	EGSLEQEKKLRMDLE	.......K.L.....	nuclear		intracellular organelle; intracellular non-membrane-bounded organelle; cytoskeletal part; intracellular organelle part; macromolecular complex; intracellular part; cell; actin cytoskeleton; organelle part; non-membrane-bounded organelle; organelle; cytoskeleton; cell part; intracellular; myosin complex; protein complex;	ATP binding; motor activity; nucleoside phosphate binding; nucleoside binding; ion binding; hydrolase activity, acting on acid anhydrides; hydrolase activity; nucleotide binding; nucleoside-triphosphatase activity; organic cyclic compound binding; heterocyclic compound binding; ribonucleoside binding; carbohydrate derivative binding; pyrophosphatase activity; catalytic activity; ribonucleotide binding; binding; purine ribonucleoside binding; anion binding; purine ribonucleotide binding; adenyl ribonucleotide binding; adenyl nucleotide binding; purine nucleoside binding; hydrolase activity, acting on acid anhydrides, in phosphorus-containing anhydrides; purine nucleotide binding; small molecule binding; purine ribonucleoside triphosphate binding;	K10352	MYH; myosin heavy chain	dre04530 Tight junction - Danio rerio (zebrafish);	IPR002928; IPR004009; IPR001609; IPR027417; IPR027401;	Myosin tail; Myosin, N-terminal, SH3-like; Myosin head, motor domain; P-loop containing nucleoside triphosphate hydrolase; Myosin-like IQ motif-containing domain;
Q6DHL6	rps6	40S ribosomal protein S6	_LFNLSK(cr)EDDVR_	149	1	RKLFNLSKEDDVRQY		cytosol	cellular component biogenesis; ribonucleoprotein complex biogenesis; cellular aromatic compound metabolic process; cellular protein metabolic process; biosynthetic process; cellular metabolic process; organic substance metabolic process; metabolic process; heterocycle metabolic process; nucleobase-containing compound metabolic process; rRNA processing; cellular nitrogen compound metabolic process; protein metabolic process; macromolecule metabolic process; translation; macromolecule biosynthetic process; cellular process; ncRNA processing; rRNA metabolic process; ribosomal small subunit biogenesis; ribosome biogenesis; organic cyclic compound metabolic process; nitrogen compound metabolic process; primary metabolic process; organic substance biosynthetic process; cellular biosynthetic process; RNA metabolic process; nucleic acid metabolic process; cellular macromolecule biosynthetic process; cellular macromolecule metabolic process; gene expression; ncRNA metabolic process; cellular component organization or biogenesis;	intracellular organelle; ribosome; intracellular non-membrane-bounded organelle; small ribosomal subunit; cytoplasm; intracellular organelle part; macromolecular complex; intracellular part; cell; cytosol; organelle part; non-membrane-bounded organelle; organelle; intracellular; ribosomal subunit; cell part; cytosolic part; ribonucleoprotein complex; cytosolic small ribosomal subunit; cytoplasmic part; cytosolic ribosome;	structural constituent of ribosome; structural molecule activity;	K02991	RP-S6e; small subunit ribosomal protein S6e	dre04910 Insulin signaling pathway - Danio rerio (zebrafish); dre03010 Ribosome - Danio rerio (zebrafish); dre04150 mTOR signaling pathway - Danio rerio (zebrafish);		

F1QXV8	pgk1	Phosphoglycerate kinase	_LHLDK(cr)VDVK_	11	1	SNKLHLDKVDVKGKR	......DK.......	cytosol	single-organism carbohydrate catabolic process; glycolytic process; single-organism catabolic process; small molecule metabolic process; single-organism cellular process; single-organism process; carbohydrate metabolic process; primary metabolic process; cellular metabolic process; organic substance metabolic process; carbohydrate catabolic process; single-organism carbohydrate metabolic process; organic substance catabolic process; metabolic process; pyruvate metabolic process; generation of precursor metabolites and energy; single-organism metabolic process; carboxylic acid metabolic process; monocarboxylic acid metabolic process; organic acid metabolic process; cellular process; catabolic process; oxoacid metabolic process;		phosphotransferase activity, carboxyl group as acceptor; kinase activity; catalytic activity; transferase activity; phosphoglycerate kinase activity; transferase activity, transferring phosphorus-containing groups;	K00927	PGK; phosphoglycerate kinase [EC:2.7.2.3]	dre00010 Glycolysis / Gluconeogenesis - Danio rerio (zebrafish); dre01230 Biosynthesis of amino acids - Danio rerio (zebrafish); dre01200 Carbon metabolism - Danio rerio (zebrafish); dre01100 Metabolic pathways - Danio rerio (zebrafish);	IPR015824; IPR015901;	Phosphoglycerate kinase, N-terminal; Phosphoglycerate kinase, C-terminal;
Q0D2W2	tnni2a.4	Fast muscle troponin I	_LHQQIDK(cr)VDEER_	70	1	KLHQQIDKVDEERYD	......DK.......	mitochondria								
Q6NXC1	rbm4.3	Uncharacterized protein	_LHVANVEK(cr)GTDDELR_	87	1	LHVANVEKGTDDELR		cytosol			organic cyclic compound binding; heterocyclic compound binding; nucleoside phosphate binding; binding; nucleic acid binding; nucleotide binding; small molecule binding;				IPR000504; IPR012677;	RNA recognition motif domain; Nucleotide-binding, alpha-[illegible]
B0R193	ubb	Uncharacterized protein	_LIFAGK(cr)QLEDGR_	48	1	QRLIFAGKQLEDGRT	.......K.L.....	cytosol,nuclear							IPR029071; IPR000626;	Ubiquitin-related domain; Ubiquitin-like;
Q08BA1	atp5a1	ATP synthase subunit alpha	_LIK(cr)EGDIVK_	125	1	FGNDKLIKEGDIVKR		mitochondria	nucleoside metabolic process; purine nucleoside metabolic process; purine ribonucleoside monophosphate metabolic process; nucleotide metabolic process; energy coupled proton transmembrane transport, against electrochemical gradient; single-organism process; biosynthetic process; nucleoside biosynthetic process; purine ribonucleoside biosynthetic process; ribonucleoside monophosphate biosynthetic process; cellular metabolic process; phosphate-containing compound metabolic process; establishment of localization; ribose phosphate biosynthetic process; metabolic process; carbohydrate derivative biosynthetic process; nucleoside phosphate metabolic process; ribose phosphate metabolic process; purine nucleoside triphosphate biosynthetic process; single-organism transport; heterocycle biosynthetic process; purine ribonucleoside triphosphate biosynthetic process; cation transmembrane transport; small molecule metabolic process; ATP biosynthetic process; single-organism cellular process; purine-containing compound metabolic process; organic cyclic compound metabolic process; purine ribonucleoside triphosphate metabolic process; energy coupled proton transport, down electrochemical	proton-transporting ATP synthase complex, catalytic core F(1); membrane; membrane part; cell part; intracellular; proton-transporting two-sector ATPase complex; macromolecular complex; proton-transporting two-sector ATPase complex, catalytic domain; intracellular part; protein complex; cell; proton-transporting ATP synthase complex;	ATPase activity, coupled to transmembrane movement of ions; nucleoside phosphate binding; primary active transmembrane transporter activity; substrate-specific transporter activity; hydrolase activity, acting on acid anhydrides; ATPase activity, coupled to transmembrane movement of ions, rotational mechanism; monovalent inorganic cation transmembrane transporter activity; hydrolase activity; hydrogen ion transmembrane transporter activity; substrate-specific transmembrane transporter activity; proton-transporting ATP synthase activity, rotational mechanism; ATPase activity; organic cyclic compound binding; heterocyclic compound binding; cation-transporting ATPase activity; pyrophosphatase activity; catalytic activity; ribonucleotide binding; ATPase activity, coupled to movement of substances; binding; purine ribonucleoside binding; hydrogen-exporting ATPase activity; hydrolase activity, acting on acid anhydrides, catalyzing transmembrane movement of substances; anion binding; adenyl ribonucleotide binding; adenyl nucleotide binding; inorganic cation transmembrane transporter activity; hydrolase activity, acting on acid anhydrides, in phosphorus-containing anhydrides; transporter activity; purine nucleotide binding; purine ribonucleoside triphosphate binding; active transmembrane transporter activity; ATP binding; ion transmembrane transporter activity; ion binding; nucleoside binding; nucleotide binding; nucleoside-triphosphatase activity; proton-transporting ATPase activity, rotational mechanism; ribonucleoside binding; P-P-bond-hydrolysis-driven transmembrane transporter activity; carbohydrate derivative binding; ATPase activity, coupled to transmembrane movement of substances; ATPase activity, coupled; purine ribonucleotide binding; purine nucleoside binding; cation transmembrane transporter activity; transmembrane transporter activity; small molecule binding;	K02132	ATPeF1A; F-type H+-transporting ATPase subunit alpha	dre01100 Metabolic pathways - Danio rerio (zebrafish); dre00190 Oxidative phosphorylation - Danio rerio (zebrafish);	IPR000793; IPR004100; IPR000194; IPR027417; IPR023366;	ATPase, F1/V1/A1 complex, alpha/beta subunit, C-terminal; ATPase, F1 complex alpha/beta subunit, N-terminal domain; ATPase, F1/V1/A1 complex, alpha/beta subunit, nucleotide-binding domain; P-loop containing nucleoside triphosphate hydrolase; ATP synthase subunit alpha-like domain;
P13104	tpma	Tropomyosin alpha-1 chain	_LK(cr)ATEDELDK_	51	1	VALQKKLKATEDELD		cytosol		cytoskeleton; organelle; intracellular organelle; intracellular non-membrane-bounded organelle; cell part; intracellular; cytoplasm; intracellular part; cell; non-membrane-bounded organelle;		K10373	TPM1; tropomyosin 1	dre04260 Cardiac muscle contraction - Danio rerio (zebrafish); dre04261 Adrenergic signaling in cardiomyocytes - Danio rerio [illegible]		
P13104	tpma	Tropomyosin alpha-1 chain	_LK(cr)ATEDELDKYSEALK_	51	1	VALQKKLKATEDELD		cytosol		cytoskeleton; organelle; intracellular organelle; intracellular non-membrane-bounded organelle; cell part; intracellular; cytoplasm; intracellular part; cell; non-membrane-bounded organelle;		K10373	TPM1; tropomyosin 1	dre04260 Cardiac muscle contraction - Danio rerio (zebrafish); dre04261 Adrenergic signaling in cardiomyocytes - Danio rerio [illegible]		
Q6AZA0	acat1	Acetyl-CoA acetyltransferase, mitochondrial	_LK(cr)AVFLK_	261	1	FSKVPKLKAVFLKEN	.......K.V.....	mitochondria		organelle; intracellular membrane-bounded organelle; intracellular organelle; cell part; intracellular; cytoplasm; intracellular part; membrane-bounded organelle; cell; mitochondrion; cytoplasmic part;	transferase activity, transferring acyl groups; catalytic activity; binding; ion binding; metal ion binding; cation binding; acetyl-CoA C-acyltransferase activity; C-acyltransferase activity; acetyl-CoA C-acetyltransferase activity; transferase activity; transferase activity, transferring acyl groups other than amino-acyl groups; acetyltransferase activity; C-acetyltransferase activity;	K00626	E2.3.1.9; acetyl-CoA C-acetyltransferase [EC:2.3.1.9]	dre01200 Carbon metabolism - Danio rerio (zebrafish); dre00900 Terpenoid backbone biosynthesis - Danio rerio (zebrafish); dre01212 Fatty acid metabolism - Danio rerio (zebrafish); dre00630 Glyoxylate and dicarboxylate metabolism - Danio rerio (zebrafish); dre00280 Valine, leucine and isoleucine degradation - Danio rerio (zebrafish); dre00640 Propanoate metabolism - Danio rerio (zebrafish); dre01100 Metabolic pathways - Danio rerio (zebrafish); dre00310 Lysine degradation - Danio rerio (zebrafish); dre00620 Pyruvate metabolism - Danio rerio (zebrafish); dre00072 Synthesis and degradation of ketone bodies - Danio rerio (zebrafish); dre00380 Tryptophan metabolism - Danio rerio (zebrafish); dre00071 Fatty acid degradation - Danio rerio (zebrafish); dre00650 Butanoate metabolism - Danio rerio (zebrafish);	IPR016039; IPR020616; IPR020617;	Thiolase-like; Thiolase, N-terminal; Thiolase, C-terminal;

E9QIC9	lmnb1	Uncharacterized protein	_LK(cr)DVEALLNAK_	137	1	TSSQNRLKDVEALLN	.......K.V......	mitochondria		intracellular organelle; intracellular non-membrane-bounded organelle; cytoskeletal part; intracellular organelle part; macromolecular complex; intracellular part; cell; organelle part; non-membrane-bounded organelle; organelle; cytoskeleton; intermediate filament cytoskeleton; cell part; intracellular; intermediate filament; protein complex;	structural molecule activity;					
F1QIR4	-	Uncharacterized protein	_LK(cr)EAYAK_	863	1	KEEFTKLKEAYAKSE	..E....K.......	cytosol	cellular component biogenesis; muscle cell development; single-multicellular organism process; cellular component organization; muscle system process; cellular developmental process; cellular component morphogenesis; single-organism process; actin filament organization; cell development; anatomical structure morphogenesis; cell differentiation; actin cytoskeleton organization; muscle contraction; cellular component assembly; organelle assembly; macromolecular complex assembly; myofibril assembly; musculoskeletal movement; slow-twitch skeletal muscle fiber contraction; muscle structure development; cytoskeleton organization; anatomical structure formation involved in morphogenesis; cellular process; striated muscle myosin thick filament assembly; protein complex assembly; cellular protein complex assembly; skeletal muscle thin filament assembly; developmental process; cellular macromolecular complex assembly; striated muscle cell differentiation; actomyosin structure organization; system process; protein complex subunit organization; multicellular organismal movement; single-organism cellular process; cellular component assembly involved in morphogenesis; striated muscle contraction; myosin filament [illegible]	intracellular organelle; intracellular non-membrane-bounded organelle; cytoskeletal part; intracellular organelle part; macromolecular complex; intracellular part; cell; actin cytoskeleton; organelle part; non-membrane-bounded organelle; organelle; cytoskeleton; cell part; intracellular; myosin complex; protein complex;	ATP binding; motor activity; nucleoside phosphate binding; nucleoside binding; ion binding; hydrolase activity, acting on acid anhydrides; hydrolase activity; nucleotide binding; nucleoside-triphosphatase activity; organic cyclic compound binding; heterocyclic compound binding; ribonucleoside binding; carbohydrate derivative binding; pyrophosphatase activity; catalytic activity; ribonucleotide binding; binding; purine ribonucleoside binding; anion binding; purine ribonucleotide binding; adenyl ribonucleotide binding; adenyl nucleotide binding; purine nucleoside binding; hydrolase activity, acting on acid anhydrides, in phosphorus-containing anhydrides; purine nucleotide binding; small molecule binding; purine ribonucleoside triphosphate binding;	K10352	MYH; myosin heavy chain	dre04530 Tight junction - Danio rerio (zebrafish);	IPR002928; IPR004009; IPR027417; IPR001609; IPR027401;	Myosin tail; Myosin, N-terminal, SH3-like; P-loop containing nucleoside triphosphate hydrolase; Myosin head, motor domain; Myosin-like IQ motif-containing domain;
B8A568	myhz1.1	Uncharacterized protein	_LK(cr)ETTERLEDEEEINAELTAK_	922	1	IQLEAKLKETTERLE		nuclear		intracellular organelle; intracellular non-membrane-bounded organelle; cytoskeletal part; intracellular organelle part; macromolecular complex; intracellular part; cell; actin cytoskeleton; organelle part; non-membrane-bounded organelle; organelle; cytoskeleton; cell part; intracellular; myosin complex; protein complex;	ATP binding; motor activity; nucleoside phosphate binding; nucleoside binding; ion binding; hydrolase activity, acting on acid anhydrides; hydrolase activity; nucleotide binding; nucleoside-triphosphatase activity; organic cyclic compound binding; heterocyclic compound binding; ribonucleoside binding; carbohydrate derivative binding; pyrophosphatase activity; catalytic activity; ribonucleotide binding; binding; purine ribonucleoside binding; anion binding; purine ribonucleotide binding; adenyl ribonucleotide binding; adenyl nucleotide binding; purine nucleoside binding; hydrolase activity, acting on acid anhydrides, in phosphorus-containing anhydrides; purine nucleotide binding; small molecule binding; purine ribonucleoside triphosphate binding;	K10352	MYH; myosin heavy chain	dre04530 Tight junction - Danio rerio (zebrafish);	IPR002928; IPR004009; IPR001609; IPR027417; IPR027401;	Myosin tail; Myosin, N-terminal, SH3-like; Myosin head, motor domain; P-loop containing nucleoside triphosphate hydrolase; Myosin-like IQ motif-containing domain;
O93409	mylpfa	Uncharacterized protein	_LK(cr)GADPEDVIVSAFK_	93	1	TMFGEKLKGADPEDV		cytosol,nucle ar			cation binding; metal ion binding; calcium ion binding; binding; ion binding;	K12758	MYLPF; fast skeletal myosin light chain 2	dre04510 Focal adhesion - Danio rerio (zebrafish); dre04530 Tight junction - Danio rerio (zebrafish); dre04810 Regulation of actin cytoskeleton - Danio rerio (zebrafish);	IPR011992; IPR002048;	EF-hand domain pair; EF-hand domain;
Q6PBW7	rps19	Ribosomal protein S19	_LK(cr)VPDWVDIVK_	30	1	LKKSGKLKVPDWVDI		cytosol	cellular protein metabolic process; biosynthetic process; primary metabolic process; cellular metabolic process; organic substance biosynthetic process; cellular biosynthetic process; organic substance metabolic process; metabolic process; cellular macromolecule biosynthetic process; cellular macromolecule metabolic process; gene expression; macromolecule metabolic process; protein metabolic process; macromolecule biosynthetic process; translation; cellular process;	ribosome; organelle; intracellular organelle; intracellular non-membrane-bounded organelle; cell part; intracellular; cytoplasm; macromolecular complex; intracellular part; cell; ribonucleoprotein complex; cytoplasmic part; non-membrane-bounded organelle;	structural constituent of ribosome; structural molecule activity;	K02966	RP-S19e; small subunit ribosomal protein S19e	dre03010 Ribosome - Danio rerio (zebrafish);		
Q08CK7	igf2bp1	Insulin-like growth factor 2 mRNA-binding protein 1	_LKEENFFGPK(cr)EEVK_	485	1	EENFFGPKEEVKLET	.......K.E......	cytosol,nucle ar	regulation of cellular process; regulation of gene expression; regulation of cellular biosynthetic process; regulation of primary metabolic process; regulation of macromolecule metabolic process; posttranscriptional regulation of gene expression; regulation of macromolecule biosynthetic process; regulation of cellular metabolic process; regulation of biological process; regulation of cellular protein metabolic process; regulation of translation; regulation of protein metabolic process; regulation of biosynthetic process; regulation of metabolic process; biological regulation; regulation of cellular macromolecule biosynthetic process;	organelle; intracellular membrane-bounded organelle; intracellular organelle; nucleus; CRD-mediated mRNA stability complex; cell part; intracellular; cytoplasm; macromolecular complex; intracellular part; membrane-bounded organelle; protein complex; cell; cytoplasmic part;	organic cyclic compound binding; heterocyclic compound binding; RNA binding; mRNA binding; poly(A) RNA binding; nucleoside phosphate binding; mRNA 3'-UTR binding; binding; nucleic acid binding; nucleotide binding; small molecule binding;	K17391			IPR004087; IPR004088; IPR000504; IPR012677;	K Homology domain; K Homology domain, type 1; RNA recognition motif domain; Nucleotide-binding, alpha-beta plait;
Q7ZTS4	krt18	Keratin, type I cytoskeletal 18	_LLDGGDFK(cr)LQDALEEQK_	394	1	LLDGGDFKLQDALEE		mitochondria		intracellular organelle; intracellular non-membrane-bounded organelle; cytoskeletal part; intracellular organelle part; macromolecular complex; intracellular part; cell; organelle part; non-membrane-bounded organelle; organelle; cytoskeleton; intermediate filament cytoskeleton; cell part; intracellular; intermediate filament; protein complex;	structural molecule activity;	K07604				

Q8UVG7	fabp3	Fatty acid binding protein 3, muscle and heart	_LLHVQK(cr)WDGK_	97	1	GKLLHVQKWDGKETT		cytosol			lipid binding; binding; transporter activity;	K08752	FABP3; fatty acid-binding protein 3, muscle and heart	dre03320 PPAR signaling pathway - Danio rerio (zebrafish);	IPR000463; IPR012674; IPR000566; IPR011038;	Cytosolic fatty-acid binding; Calycin; Lipocalin/cytosolic fatty-acid binding domain; Calycin-like;
Q7T306	ckmb	Uncharacterized protein	_LMVEMEK(cr)K_	364	1	KLMVEMEKKLEKGES	.......K.L.....	cytosol			ATP binding; nucleoside phosphate binding; nucleoside binding; ion binding; nucleotide binding; organic cyclic compound binding; heterocyclic compound binding; ribonucleoside binding; carbohydrate derivative binding; kinase activity; catalytic activity; ribonucleotide binding; binding; purine ribonucleoside binding; anion binding; purine ribonucleotide binding; adenyl ribonucleotide binding; adenyl nucleotide binding; purine nucleoside binding; transferase activity; transferase activity, transferring phosphorus-containing groups; purine nucleotide binding; small molecule binding; purine ribonucleoside triphosphate binding;				IPR022413; IPR022414; IPR014746;	ATP:guanido phosphotransferase, N-terminal; ATP:guanido phosphotransferase, catalytic domain; Glutamine synthetase/guanido kinase, catalytic domain;
A2BHA3	ckma	Uncharacterized protein	_LMVEMEK(cr)K_	365	1	KLMVEMEKKLEKGES	.......K.L.....	cytosol			ATP binding; nucleoside phosphate binding; nucleoside binding; ion binding; nucleotide binding; organic cyclic compound binding; heterocyclic compound binding; ribonucleoside binding; carbohydrate derivative binding; kinase activity; catalytic activity; ribonucleotide binding; binding; purine ribonucleoside binding; anion binding; purine ribonucleotide binding; adenyl ribonucleotide binding; adenyl nucleotide binding; purine nucleoside binding; transferase activity; transferase activity, transferring phosphorus-containing groups; purine nucleotide binding; small molecule binding; purine ribonucleoside triphosphate binding;	K00933	E2.7.3.2; creatine kinase [EC:2.7.3.2]	dre00330 Arginine and proline metabolism - Danio rerio (zebrafish); dre01100 Metabolic pathways - Danio rerio (zebrafish);	IPR022413; IPR022414; IPR014746;	ATP:guanido phosphotransferase, N-terminal; ATP:guanido phosphotransferase, catalytic domain; Glutamine synthetase/guanido kinase, catalytic domain;
B8JIS1	atp5f1	Uncharacterized protein	_LNADK(cr)IAK_	127	1	ADKLNADKIAKAQEV	......DK.......	mitochondria	nucleoside metabolic process; purine nucleoside metabolic process; purine ribonucleoside monophosphate metabolic process; nucleotide metabolic process; single-organism process; purine ribonucleoside biosynthetic process; nucleoside biosynthetic process; biosynthetic process; ribonucleoside monophosphate biosynthetic process; cellular metabolic process; phosphate-containing compound metabolic process; establishment of localization; ribose phosphate biosynthetic process; metabolic process; carbohydrate derivative biosynthetic process; nucleoside phosphate metabolic process; ribose phosphate metabolic process; purine nucleoside triphosphate biosynthetic process; single-organism transport; heterocycle biosynthetic process; cation transmembrane transport; purine ribonucleoside triphosphate biosynthetic process; small molecule metabolic process; ATP biosynthetic process; single-organism cellular process; purine ribonucleoside triphosphate metabolic process; organic cyclic compound metabolic process; purine-containing compound metabolic process; energy coupled proton transport, down electrochemical gradient; ribonucleoside triphosphate metabolic process; purine	intracellular organelle; membrane; mitochondrial part; organelle membrane; organelle envelope; intracellular organelle part; cytoplasm; macromolecular complex; intracellular part; mitochondrial proton-transporting ATP synthase complex, coupling factor F(o); cell; mitochondrial membrane part; proton-transporting ATP synthase complex; mitochondrion; organelle part; intracellular membrane-bounded organelle; organelle; mitochondrial envelope; mitochondrial proton-transporting ATP synthase complex; membrane part; cell part; intracellular; proton-transporting two-sector ATPase complex; envelope; organelle inner membrane; proton-transporting ATP synthase complex, coupling factor F(o); protein complex; membrane-bounded organelle; mitochondrial inner membrane; proton-transporting two-sector ATPase complex, proton-transporting domain; mitochondrial membrane; cytoplasmic part;	ion transmembrane transporter activity; substrate-specific transporter activity; monovalent inorganic cation transmembrane transporter activity; inorganic cation transmembrane transporter activity; hydrogen ion transmembrane transporter activity; cation transmembrane transporter activity; substrate-specific transmembrane transporter activity; transporter activity; transmembrane transporter activity;	K02127	ATPeF0B; F-type H+-transporting ATPase subunit b	dre01100 Metabolic pathways - Danio rerio (zebrafish); dre00190 Oxidative phosphorylation - Danio rerio (zebrafish);		
B0UY94	eif5a2	Eukaryotic translation initiation factor 5A	_LPEGDLGK(cr)EIESK_	122	1	LPEGDLGKEIESKF_		cytosol	positive regulation of cellular protein metabolic process; positive regulation of protein complex disassembly; posttranscriptional regulation of gene expression; regulation of translational termination; positive regulation of biosynthetic process; single-organism process; biosynthetic process; cellular metabolic process; positive regulation of cellular component organization; metabolic process; positive regulation of metabolic process; protein metabolic process; macromolecule metabolic process; translation; regulation of gene expression; regulation of cellular biosynthetic process; positive regulation of translational termination; positive regulation of translation; regulation of primary metabolic process; single-organism cellular process; positive regulation of cellular process; regulation of cellular metabolic process; regulation of cellular protein metabolic process; regulation of biological process; single-organism metabolic process; positive regulation of macromolecule biosynthetic process; cellular macromolecule metabolic process; regulation of metabolic process; positive regulation of macromolecule metabolic process; cellular protein modification process; regulation of protein complex disassembly; protein modification process; regulation of cellular		organic cyclic compound binding; heterocyclic compound binding; ribosome binding; RNA binding; translation factor activity, nucleic acid binding; translation elongation factor activity; binding; nucleic acid binding; macromolecular complex binding; translation initiation factor activity; ribonucleoprotein complex binding;				IPR008991; IPR012340; IPR014722; IPR020189;	Translation protein SH3-like domain; Nucleic acid-binding, OB-fold; Ribosomal protein L2 domain 2; Translation elongation factor, IF5A C-terminal;

Q1LWN2	vtg1	Uncharacterized protein	_LPIIVTTYAK(cr)K_	1274	1	IIVTTYAKKLGKHIL	.......K.L.....	plasma membrane			lipid transporter activity; substrate-specific transporter activity; transporter activity;				IPR011030; IPR015817; IPR015818; IPR001747; IPR015258; IPR015816; IPR015819; IPR015255;	Vitellinogen, superhelical; Vitellinogen, open beta-sheet, subdomain 1; Vitellinogen, open beta-sheet, subdomain 2; Lipid transport protein, N-terminal; Vitellinogen, beta-sheet shell; Vitellinogen, beta-sheet N-terminal; Lipid transport protein, beta-sheet shell; Vitellinogen, open beta-sheet;
Q8AY63	ckbb	Brain-subtype creatine kinase	_LPNVSK(cr)HEK_	304	1	VKLPNVSKHEKFGEI	.......K.E.....	cytosol			ATP binding; nucleoside phosphate binding; creatine kinase activity; nucleoside binding; ion binding; phosphotransferase activity, nitrogenous group as acceptor; nucleotide binding; organic cyclic compound binding; heterocyclic compound binding; ribonucleoside binding; carbohydrate derivative binding; kinase activity; catalytic activity; ribonucleotide binding; binding; purine ribonucleoside binding; anion binding; purine ribonucleotide binding; adenyl ribonucleotide binding; adenyl nucleotide binding; purine nucleoside binding; transferase activity; transferase activity, transferring phosphorus-containing groups; purine nucleotide binding; small molecule binding; purine ribonucleoside triphosphate binding;	K00933	E2.7.3.2; creatine kinase [EC:2.7.3.2]	dre00330 Arginine and proline metabolism - Danio rerio (zebrafish); dre01100 Metabolic pathways - Danio rerio (zebrafish);	IPR022413; IPR014746; IPR022414;	ATP:guanido phosphotransferase, N-terminal; Glutamine synthetase/guanido kinase, catalytic domain; ATP:guanido phosphotransferase, catalytic domain;
Q803E5	etf1b	Eukaryotic translation termination factor 1	_LQAK(cr)VLK_	249	1	FDPRLQAKVLKLVDI	.......K.L.....	cytosol		intracellular part; cell; cell part; intracellular; cytoplasm;	organic cyclic compound binding; translation termination factor activity; heterocyclic compound binding; RNA binding; translation factor activity, nucleic acid binding; binding; nucleic acid binding; translation release factor activity, codon specific; translation release factor activity;	K03265	ETF1; peptide chain release factor subunit 1	dre03015 mRNA surveillance pathway - Danio rerio (zebrafish);	IPR005141; IPR029064; IPR005142; IPR005140; IPR024049;	eRF1 domain 2; 50S ribosomal protein L30e-like; eRF1 domain 3; eRF1 domain 1/Pelota-like; Peptide Chain Release Factor eRF1/aRF1, N-terminal;
B8JHR2	tpt1	Translationally-controlled tumor protein homolog	_LQETSYDK(cr)K_	72	1	LQETSYDKKSYTAYI	......DK.......	cytosol							IPR011323; IPR011057;	Mss4/translationally controlled tumour-associated TCTP; Mss4-like;
Q92051	cahz	Carbonic anhydrase	_LQK(cr)VLDALDDIK_	158	1	AANPRLQKVLDALDD	.......K.L.....	cytosol	gas transport; response to osmotic stress; response to stress; response to abiotic stimulus; small molecule metabolic process; single-organism cellular process; single-organism process; organic substance transport; response to salt stress; response to stimulus; cellular metabolic process; hypotonic response; establishment of localization; transport; metabolic process; one-carbon compound transport; one-carbon metabolic process; single-organism localization; carbon dioxide transport; single-organism metabolic process; localization; cellular process; hypotonic salinity response; single-organism transport;		transition metal ion binding; catalytic activity; binding; ion binding; lyase activity; metal ion binding; cation binding; hydro-lyase activity; zinc ion binding; carbonate dehydratase activity; carbon-oxygen lyase activity;	K18245	CA2; carbonic anhydrase 2 [EC:4.2.1.1]	dre00910 Nitrogen metabolism - Danio rerio (zebrafish);	IPR001148;	Alpha carbonic anhydrase;
Q7T306	ckmb	Uncharacterized protein	_LSTHAK(cr)FEEILTR_	306	1	PKLSTHAKFEEILTR	.......K.E.....	cytosol			ATP binding; nucleoside phosphate binding; nucleoside binding; ion binding; nucleotide binding; organic cyclic compound binding; heterocyclic compound binding; ribonucleoside binding; carbohydrate derivative binding; kinase activity; catalytic activity; ribonucleotide binding; binding; purine ribonucleoside binding; anion binding; purine ribonucleotide binding; adenyl ribonucleotide binding; adenyl nucleotide binding; purine nucleoside binding; transferase activity; transferase activity, transferring phosphorus-containing groups; purine nucleotide binding; small molecule binding; purine ribonucleoside triphosphate binding;				IPR022413; IPR022414; IPR014746;	ATP:guanido phosphotransferase, N-terminal; ATP:guanido phosphotransferase, catalytic domain; Glutamine synthetase/guanido kinase, catalytic domain;
A2BHA3	ckma	Uncharacterized protein	_LSTHAK(cr)FEEILTR_	307	1	PKLSTHAKFEEILTR	.......K.E.....	cytosol			ATP binding; nucleoside phosphate binding; nucleoside binding; ion binding; nucleotide binding; organic cyclic compound binding; heterocyclic compound binding; ribonucleoside binding; carbohydrate derivative binding; kinase activity; catalytic activity; ribonucleotide binding; binding; purine ribonucleoside binding; anion binding; purine ribonucleotide binding; adenyl ribonucleotide binding; adenyl nucleotide binding; purine nucleoside binding; transferase activity; transferase activity, transferring phosphorus-containing groups; purine nucleotide binding; small molecule binding; purine ribonucleoside triphosphate binding;	K00933	E2.7.3.2; creatine kinase [EC:2.7.3.2]	dre00330 Arginine and proline metabolism - Danio rerio (zebrafish); dre01100 Metabolic pathways - Danio rerio (zebrafish);	IPR022413; IPR022414; IPR014746;	ATP:guanido phosphotransferase, N-terminal; ATP:guanido phosphotransferase, catalytic domain; Glutamine synthetase/guanido kinase, catalytic domain;
A2BHA3	ckma	Uncharacterized protein	_LSVEALSSLDGEFK(cr)GK_	170	1	SSLDGEFKGKYYPLK		cytosol			ATP binding; nucleoside phosphate binding; nucleoside binding; ion binding; nucleotide binding; organic cyclic compound binding; heterocyclic compound binding; ribonucleoside binding; carbohydrate derivative binding; kinase activity; catalytic activity; ribonucleotide binding; binding; purine ribonucleoside binding; anion binding; purine ribonucleotide binding; adenyl ribonucleotide binding; adenyl nucleotide binding; purine nucleoside binding; transferase activity; transferase activity, transferring phosphorus-containing groups; purine nucleotide binding; small molecule binding; purine ribonucleoside triphosphate binding;	K00933	E2.7.3.2; creatine kinase [EC:2.7.3.2]	dre00330 Arginine and proline metabolism - Danio rerio (zebrafish); dre01100 Metabolic pathways - Danio rerio (zebrafish);	IPR022413; IPR022414; IPR014746;	ATP:guanido phosphotransferase, N-terminal; ATP:guanido phosphotransferase, catalytic domain; Glutamine synthetase/guanido kinase, catalytic domain;
Q6XG62	icn	Protein S100 (Fragment)	_LTLSK(cr)GELK_	32	1	GDKLTLSKGELKELL	.......K.E.....	cytosol			cation binding; metal ion binding; calcium ion binding; binding; ion binding;				IPR011992; IPR002048; IPR013787;	EF-hand domain pair; EF-hand domain; S100/CaBP-9k-type, calcium binding, subdomain;

Q7T334	mdh2	Malate dehydrogenase	_LVADAMTELK(cr)GSIK_	323	1	ADAMTELKGSIKKGE		mitochondria	tricarboxylic acid metabolic process; dicarboxylic acid metabolic process; oxidation-reduction process; small molecule metabolic process; tricarboxylic acid cycle; single-organism cellular process; single-organism process; primary metabolic process; carbohydrate metabolic process; cellular respiration; cellular metabolic process; organic substance metabolic process; citrate metabolic process; metabolic process; generation of precursor metabolites and energy; cellular carbohydrate metabolic process; aerobic respiration; single-organism metabolic process; energy derivation by oxidation of organic compounds; malate metabolic process; carboxylic acid metabolic process; organic acid metabolic process; cellular process; oxoacid metabolic process;		organic cyclic compound binding; heterocyclic compound binding; oxidoreductase activity, acting on CH-OH group of donors; oxidoreductase activity, acting on the CH-OH group of donors, NAD or NADP as acceptor; nucleoside phosphate binding; catalytic activity; binding; nucleotide binding; malate dehydrogenase activity; L-malate dehydrogenase activity; small molecule binding; oxidoreductase activity;	K00026	MDH2; malate dehydrogenase [EC:1.1.1.37]	dre01200 Carbon metabolism - Danio rerio (zebrafish); dre01100 Metabolic pathways - Danio rerio (zebrafish); dre00620 Pyruvate metabolism - Danio rerio (zebrafish); dre00630 Glyoxylate and dicarboxylate metabolism - Danio rerio (zebrafish); dre00020 Citrate cycle (TCA cycle) - Danio rerio (zebrafish); dre00270 Cysteine and methionine metabolism - Danio rerio (zebrafish);	IPR022383; IPR016040; IPR001236; IPR015955;	Lactate/malate dehydrogenase, C-terminal; NAD(P)-binding domain; Lactate/malate dehydrogenase, N-terminal; Lactate dehydrogenase/glycoside hydrolase, family 4, C-terminal;
F8W3N3	fabp7a	Uncharacterized protein	_LVDSQNFDEYMK(cr)SLGVGFATR_	22	1	QNFDEYMKSLGVGFA	.......K.L.....	cytosol			lipid binding; binding; transporter activity;				IPR000463; IPR012674; IPR000566; IPR011038;	Cytosolic fatty-acid binding; Calycin; Lipocalin/cytosolic fatty-acid binding domain; Calycin-like;
Q1MTC4	vtg2	Uncharacterized protein	_LVPEFAHDK(cr)TYVYK_	28	1	VPEFAHDKTYVYKYE	......DK.......	extracellular	response to organic cyclic compound; response to lipid; response to chemical; response to estrogen; response to oxygen-containing compound; response to steroid hormone; response to endogenous stimulus; response to alcohol; response to estradiol; response to organic substance; response to hormone; response to stimulus;		lipid transporter activity; substrate-specific transporter activity; transporter activity;				IPR011030; IPR015817; IPR015818; IPR001747; IPR015816; IPR015258; IPR015819; IPR001846; IPR015255;	Vitellinogen, superhelical; Vitellinogen, open beta-sheet, subdomain 1; Vitellinogen, open beta-sheet, subdomain 2; Lipid transport protein, N-terminal; Vitellinogen, beta-sheet N-terminal; Vitellinogen, beta-sheet shell; Lipid transport protein, beta-sheet shell; von Willebrand factor, type D domain; Vitellinogen, open beta-sheet;
A2CE73	khdrbs1a	Uncharacterized protein	_M(ox)AHAMDEVK(cr)K_	165	1	AHAMDEVKKFLMPVE		cytosol			organic cyclic compound binding; heterocyclic compound binding; RNA binding; binding; nucleic acid binding;				IPR004087; IPR004088;	K Homology domain; K Homology domain, type 1;
B8A561	myhz1.2	Uncharacterized protein	_M(ox)EIDDLSSNM(ox)EAVAK(cr)AK_	1242	1	SNMEAVAKAKANLEK		nuclear		intracellular organelle; intracellular non-membrane-bounded organelle; cytoskeletal part; intracellular organelle part; macromolecular complex; intracellular part; cell; actin cytoskeleton; organelle part; non-membrane-bounded organelle; organelle; cytoskeleton; cell part; intracellular; myosin complex; protein complex;	ATP binding; motor activity; nucleoside phosphate binding; nucleoside binding; ion binding; hydrolase activity, acting on acid anhydrides; hydrolase activity; nucleotide binding; nucleoside-triphosphatase activity; organic cyclic compound binding; heterocyclic compound binding; ribonucleoside binding; carbohydrate derivative binding; pyrophosphatase activity; catalytic activity; ribonucleotide binding; binding; purine ribonucleoside binding; anion binding; purine ribonucleotide binding; adenyl ribonucleotide binding; adenyl nucleotide binding; purine nucleoside binding; hydrolase activity, acting on acid anhydrides, in phosphorus-containing anhydrides; purine nucleotide binding; small molecule binding; purine ribonucleoside triphosphate binding;	K10352	MYH; myosin heavy chain	dre04530 Tight junction - Danio rerio (zebrafish);	IPR002928; IPR004009; IPR001609; IPR027417; IPR027401;	Myosin tail; Myosin, N-terminal, SH3-like; Myosin head, motor domain; P-loop containing nucleoside triphosphate hydrolase; Myosin-like IQ motif-containing domain;
Q6PI52	calm1a	Calmodulin	_M(ox)K(cr)DTDSEEEIR_	78	1	TMMARKMKDTDSEEE		cytosol,nuclear			cation binding; metal ion binding; calcium ion binding; binding; ion binding;	K02183	CALM; calmodulin	dre04020 Calcium signaling pathway - Danio rerio (zebrafish); dre04916 Melanogenesis - Danio rerio (zebrafish); dre04070 Phosphatidylinositol signaling system - Danio rerio (zebrafish); dre04912 GnRH signaling pathway - Danio rerio (zebrafish); dre04910 Insulin signaling pathway - Danio rerio (zebrafish); dre04261 Adrenergic signaling in cardiomyocytes - Danio rerio (zebrafish); dre04114 Oocyte meiosis - Danio rerio (zebrafish); dre04744 Phototransduction - Danio rerio (zebrafish); dre04270 Vascular smooth muscle contraction - Danio rerio	IPR002048; IPR011992;	EF-hand domain; EF-hand domain pair;
B8A561	myhz1.2	Uncharacterized protein	_M(ox)K(cr)NSYEETLDQLETLK_	1487	1	STELFKMKNSYEETL	..E....K.......	nuclear		intracellular organelle; intracellular non-membrane-bounded organelle; cytoskeletal part; intracellular organelle part; macromolecular complex; intracellular part; cell; actin cytoskeleton; organelle part; non-membrane-bounded organelle; organelle; cytoskeleton; cell part; intracellular; myosin complex; protein complex;	ATP binding; motor activity; nucleoside phosphate binding; nucleoside binding; ion binding; hydrolase activity, acting on acid anhydrides; hydrolase activity; nucleotide binding; nucleoside-triphosphatase activity; organic cyclic compound binding; heterocyclic compound binding; ribonucleoside binding; carbohydrate derivative binding; pyrophosphatase activity; catalytic activity; ribonucleotide binding; binding; purine ribonucleoside binding; anion binding; purine ribonucleotide binding; adenyl ribonucleotide binding; adenyl nucleotide binding; purine nucleoside binding; hydrolase activity, acting on acid anhydrides, in phosphorus-containing anhydrides; purine nucleotide binding; small molecule binding; purine ribonucleoside triphosphate binding;	K10352	MYH; myosin heavy chain	dre04530 Tight junction - Danio rerio (zebrafish);	IPR002928; IPR004009; IPR001609; IPR027417; IPR027401;	Myosin tail; Myosin, N-terminal, SH3-like; Myosin head, motor domain; P-loop containing nucleoside triphosphate hydrolase; Myosin-like IQ motif-containing domain;

B8A561	myhz1.2	Uncharacterized protein	_M(ox)QGSLEDQIIAANPLLEAYGNAK(cr)TVR_	238	1	LEAYGNAKTVRNDNS	.......K.V.....	nuclear		intracellular organelle; intracellular non-membrane-bounded organelle; cytoskeletal part; intracellular organelle part; macromolecular complex; intracellular part; cell; actin cytoskeleton; organelle part; non-membrane-bounded organelle; organelle; cytoskeleton; cell part; intracellular; myosin complex; protein complex;	ATP binding; motor activity; nucleoside phosphate binding; nucleoside binding; ion binding; hydrolase activity, acting on acid anhydrides; hydrolase activity; nucleotide binding; nucleoside-triphosphatase activity; organic cyclic compound binding; heterocyclic compound binding; ribonucleoside binding; carbohydrate derivative binding; pyrophosphatase activity; catalytic activity; ribonucleotide binding; binding; purine ribonucleoside binding; anion binding; purine ribonucleotide binding; adenyl ribonucleotide binding; adenyl nucleotide binding; purine nucleoside binding; hydrolase activity, acting on acid anhydrides, in phosphorus-containing anhydrides; purine nucleotide binding; small molecule binding; purine ribonucleoside triphosphate binding;	K10352	MYH; myosin heavy chain	dre04530 Tight junction - Danio rerio (zebrafish);	IPR002928; IPR004009; IPR001609; IPR027417; IPR027401;	Myosin tail; Myosin, N-terminal, SH3-like; Myosin head, motor domain; P-loop containing nucleoside triphosphate hydrolase; Myosin-like IQ motif-containing domain;
Q7ZVI7	actba	Actin, cytoplasmic 1	_M(ox)QK(cr)EITSLAPSTMK_	315	1	GIADRMQKEITSLAP	.......K.I.....	cytoskeleton		cytoskeleton; organelle; intracellular organelle; intracellular non-membrane-bounded organelle; cell part; intracellular; cytoplasm; intracellular part; cell; non-membrane-bounded organelle;	ATP binding; nucleoside phosphate binding; nucleoside binding; ion binding; nucleotide binding; organic cyclic compound binding; heterocyclic compound binding; ribonucleoside binding; carbohydrate derivative binding; ribonucleotide binding; binding; purine ribonucleoside binding; anion binding; purine ribonucleotide binding; adenyl ribonucleotide binding; adenyl nucleotide binding; purine nucleoside binding; purine nucleotide binding; small molecule binding; purine ribonucleoside triphosphate binding;	K05692	ACTB_G1; actin beta/gamm a 1	dre04510 Focal adhesion - Danio rerio (zebrafish); dre04145 Phagosome - Danio rerio (zebrafish); dre04530 Tight junction - Danio rerio (zebrafish); dre05132 Salmonella infection - Danio rerio (zebrafish); dre04810 Regulation of actin cytoskeleton - Danio rerio (zebrafish); dre04520 Adherens junction - Danio rerio (zebrafish);		
A2CE73	khdrbs1a	Uncharacterized protein	_MAHAMDEVK(cr)K_	165	1	AHAMDEVKKFLMPVE		cytosol			organic cyclic compound binding; heterocyclic compound binding; RNA binding; binding; nucleic acid binding;				IPR004087; IPR004088;	K Homology domain; K Homology domain, type 1;
B8A568	myhz1.1	Uncharacterized protein	_MEIDDLSSNM(ox)EAVAK(cr)AK_	1242	1	SNMEAVAKAKANLEK		nuclear		intracellular organelle; intracellular non-membrane-bounded organelle; cytoskeletal part; intracellular organelle part; macromolecular complex; intracellular part; cell; actin cytoskeleton; organelle part; non-membrane-bounded organelle; organelle; cytoskeleton; cell part; intracellular; myosin complex; protein complex;	ATP binding; motor activity; nucleoside phosphate binding; nucleoside binding; ion binding; hydrolase activity, acting on acid anhydrides; hydrolase activity; nucleotide binding; nucleoside-triphosphatase activity; organic cyclic compound binding; heterocyclic compound binding; ribonucleoside binding; carbohydrate derivative binding; pyrophosphatase activity; catalytic activity; ribonucleotide binding; binding; purine ribonucleoside binding; anion binding; purine ribonucleotide binding; adenyl ribonucleotide binding; adenyl nucleotide binding; purine nucleoside binding; hydrolase activity, acting on acid anhydrides, in phosphorus-containing anhydrides; purine nucleotide binding; small molecule binding; purine ribonucleoside triphosphate binding;	K10352	MYH; myosin heavy chain	dre04530 Tight junction - Danio rerio (zebrafish);	IPR002928; IPR004009; IPR001609; IPR027417; IPR027401;	Myosin tail; Myosin, N-terminal, SH3-like; Myosin head, motor domain; P-loop containing nucleoside triphosphate hydrolase; Myosin-like IQ motif-containing domain;
B8A561	myhz1.2	Uncharacterized protein	_MEIDDLSSNM(ox)EAVAK(cr)AK_	1242	1	SNMEAVAKAKANLEK		nuclear		intracellular organelle; intracellular non-membrane-bounded organelle; cytoskeletal part; intracellular organelle part; macromolecular complex; intracellular part; cell; actin cytoskeleton; organelle part; non-membrane-bounded organelle; organelle; cytoskeleton; cell part; intracellular; myosin complex; protein complex;	ATP binding; motor activity; nucleoside phosphate binding; nucleoside binding; ion binding; hydrolase activity, acting on acid anhydrides; hydrolase activity; nucleotide binding; nucleoside-triphosphatase activity; organic cyclic compound binding; heterocyclic compound binding; ribonucleoside binding; carbohydrate derivative binding; pyrophosphatase activity; catalytic activity; ribonucleotide binding; binding; purine ribonucleoside binding; anion binding; purine ribonucleotide binding; adenyl ribonucleotide binding; adenyl nucleotide binding; purine nucleoside binding; hydrolase activity, acting on acid anhydrides, in phosphorus-containing anhydrides; purine nucleotide binding; small molecule binding; purine ribonucleoside triphosphate binding;	K10352	MYH; myosin heavy chain	dre04530 Tight junction - Danio rerio (zebrafish);	IPR002928; IPR004009; IPR001609; IPR027417; IPR027401;	Myosin tail; Myosin, N-terminal, SH3-like; Myosin head, motor domain; P-loop containing nucleoside triphosphate hydrolase; Myosin-like IQ motif-containing domain;
Q6PI52	calm1a	Calmodulin	_MK(cr)DTDSEEEIR_	78	1	TMMARKMKDTDSEEE		cytosol,nucle ar			cation binding; metal ion binding; calcium ion binding; binding; ion binding;	K02183	CALM; calmodulin	dre04020 Calcium signaling pathway - Danio rerio (zebrafish); dre04916 Melanogenesis - Danio rerio (zebrafish); dre04070 Phosphatidylinositol signaling system - Danio rerio (zebrafish); dre04912 GnRH signaling pathway - Danio rerio (zebrafish); dre04910 Insulin signaling pathway - Danio rerio (zebrafish); dre04261 Adrenergic signaling in cardiomyocytes - Danio rerio (zebrafish); dre04114 Oocyte meiosis - Danio rerio (zebrafish); dre04744 Phototransduction - Danio rerio (zebrafish); dre04270 Vascular smooth muscle contraction - Danio rerio [illegible]	IPR002048; IPR011992;	EF-hand domain; EF-hand domain pair;
Q6NYR4	hspa8	Heat shock cognate 71 kDa protein	_MK(cr)EIAEAYLGK_	128	1	SMVLTKMKEIAEAYL	.......K.I.....	cytosol	response to stress; response to stimulus;		ATP binding; nucleoside phosphate binding; nucleoside binding; ion binding; nucleotide binding; organic cyclic compound binding; heterocyclic compound binding; ribonucleoside binding; carbohydrate derivative binding; ribonucleotide binding; binding; purine ribonucleoside binding; anion binding; purine ribonucleotide binding; adenyl ribonucleotide binding; adenyl nucleotide binding; purine nucleoside binding; purine nucleotide binding; small molecule binding; purine ribonucleoside triphosphate binding;	K03283	HSPA1_8; heat shock 70kDa protein 1/8	dre04010 MAPK signaling pathway - Danio rerio (zebrafish); dre04141 Protein processing in endoplasmic reticulum - Danio rerio (zebrafish); dre04144 Endocytosis - Danio rerio (zebrafish); dre03040 Spliceosome - Danio rerio [illegible]	IPR029048; IPR029047;	Heat shock protein 70kD, C-terminal domain; Heat shock protein 70kD, peptide-binding domain;
Q90473	hspa8	Heat shock cognate 71 kDa protein	_MK(cr)EIAEAYLGK_	128	1	SMVLTKMKEIAEAYL	.......K.I.....	cytosol	developmental process; response to wounding; tissue development; response to stress; wound healing; tissue regeneration; single-organism developmental process; fin regeneration; single-organism process; regeneration; developmental growth; response to stimulus; growth; anatomical structure development;		ATP binding; nucleoside phosphate binding; nucleoside binding; ion binding; nucleotide binding; organic cyclic compound binding; heterocyclic compound binding; ribonucleoside binding; carbohydrate derivative binding; ribonucleotide binding; binding; purine ribonucleoside binding; anion binding; purine ribonucleotide binding; adenyl ribonucleotide binding; adenyl nucleotide binding; purine nucleoside binding; purine nucleotide binding; small molecule binding; purine ribonucleoside triphosphate binding;	K03283	HSPA1_8; heat shock 70kDa protein 1/8	dre04010 MAPK signaling pathway - Danio rerio (zebrafish); dre04141 Protein processing in endoplasmic reticulum - Danio rerio (zebrafish); dre04144 Endocytosis - Danio rerio (zebrafish); dre03040 Spliceosome - Danio rerio [illegible]	IPR029048; IPR029047;	Heat shock protein 70kD, C-terminal domain; Heat shock protein 70kD, peptide-binding domain;
Q6PH46	cct4	T-complex protein 1 subunit delta	_MK(cr)IMVIK_	315	1	LHFLNKMKIMVIKDI		plasma membrane	protein folding; metabolic process; response to stress; response to abiotic stimulus; response to hypoxia; response to oxygen levels; cellular protein metabolic process; cellular macromolecule metabolic process; response to decreased oxygen levels; macromolecule metabolic process; protein metabolic process; response to stimulus; primary metabolic process; cellular process; cellular metabolic process; organic substance metabolic process;	intracellular part; cell; cell part; intracellular; cytoplasm;	ATP binding; nucleoside phosphate binding; nucleoside binding; ion binding; nucleotide binding; organic cyclic compound binding; heterocyclic compound binding; ribonucleoside binding; carbohydrate derivative binding; ribonucleotide binding; binding; purine ribonucleoside binding; anion binding; purine ribonucleotide binding; adenyl ribonucleotide binding; adenyl nucleotide binding; purine nucleoside binding; purine nucleotide binding; small molecule binding; purine ribonucleoside triphosphate binding;	K09496			IPR027410; IPR027409; IPR027413;	TCP-1-like chaperonin intermediate domain; GroEL-like apical domain; GroEL-like equatorial domain;

A3KQ06	rps9	Uncharacterized protein	_MK(cr)LDYILGLK_	93	1	VLDEGKMKLDYILGL		cytosol	cellular protein metabolic process; biosynthetic process; primary metabolic process; cellular metabolic process; organic substance biosynthetic process; cellular biosynthetic process; organic substance metabolic process; metabolic process; cellular macromolecule biosynthetic process; cellular macromolecule metabolic process; gene expression; macromolecule metabolic process; protein metabolic process; macromolecule biosynthetic process; translation; cellular process;	intracellular organelle; ribosome; intracellular non-membrane-bounded organelle; small ribosomal subunit; cytoplasm; intracellular organelle part; macromolecular complex; intracellular part; cell; organelle part; non-membrane-bounded organelle; organelle; ribosomal subunit; cell part; intracellular; ribonucleoprotein complex; cytoplasmic part;	organic cyclic compound binding; heterocyclic compound binding; RNA binding; binding; nucleic acid binding; structural molecule activity; structural constituent of ribosome; rRNA binding;				IPR001912; IPR002942;	Ribosomal protein S4/S9, N-terminal; RNA-binding S4 domain;
B8A568	myhz1.1	Uncharacterized protein	_MK(cr)NSYEETLDQLETLK_	1487	1	STELFKMKNSYEETL	..E....K......	nuclear		intracellular organelle; intracellular non-membrane-bounded organelle; cytoskeletal part; intracellular organelle part; macromolecular complex; intracellular part; cell; actin cytoskeleton; organelle part; non-membrane-bounded organelle; organelle; cytoskeleton; cell part; intracellular; myosin complex; protein complex;	ATP binding; motor activity; nucleoside phosphate binding; nucleoside binding; ion binding; hydrolase activity, acting on acid anhydrides; hydrolase activity; nucleotide binding; nucleoside-triphosphatase activity; organic cyclic compound binding; heterocyclic compound binding; ribonucleoside binding; carbohydrate derivative binding; pyrophosphatase activity; catalytic activity; ribonucleotide binding; binding; purine ribonucleoside binding; anion binding; purine ribonucleotide binding; adenyl ribonucleotide binding; adenyl nucleotide binding; purine nucleoside binding; hydrolase activity, acting on acid anhydrides, in phosphorus-containing anhydrides; purine nucleotide binding; small molecule binding; purine ribonucleoside triphosphate binding;	K10352	MYH; myosin heavy chain	dre04530 Tight junction - Danio rerio (zebrafish);	IPR002928; IPR004009; IPR001609; IPR027417; IPR027401;	Myosin tail; Myosin, N-terminal, SH3-like; Myosin head, motor domain; P-loop containing nucleoside triphosphate hydrolase; Myosin-like IQ motif-containing domain;
B8A561	myhz1.2	Uncharacterized protein	_MK(cr)NSYEETLDQLETLK_	1487	1	STELFKMKNSYEETL	..E....K......	nuclear		intracellular organelle; intracellular non-membrane-bounded organelle; cytoskeletal part; intracellular organelle part; macromolecular complex; intracellular part; cell; actin cytoskeleton; organelle part; non-membrane-bounded organelle; organelle; cytoskeleton; cell part; intracellular; myosin complex; protein complex;	ATP binding; motor activity; nucleoside phosphate binding; nucleoside binding; ion binding; hydrolase activity, acting on acid anhydrides; hydrolase activity; nucleotide binding; nucleoside-triphosphatase activity; organic cyclic compound binding; heterocyclic compound binding; ribonucleoside binding; carbohydrate derivative binding; pyrophosphatase activity; catalytic activity; ribonucleotide binding; binding; purine ribonucleoside binding; anion binding; purine ribonucleotide binding; adenyl ribonucleotide binding; adenyl nucleotide binding; purine nucleoside binding; hydrolase activity, acting on acid anhydrides, in phosphorus-containing anhydrides; purine nucleotide binding; small molecule binding; purine ribonucleoside triphosphate binding;	K10352	MYH; myosin heavy chain	dre04530 Tight junction - Danio rerio (zebrafish);	IPR002928; IPR004009; IPR001609; IPR027417; IPR027401;	Myosin tail; Myosin, N-terminal, SH3-like; Myosin head, motor domain; P-loop containing nucleoside triphosphate hydrolase; Myosin-like IQ motif-containing domain;
Q15I85	crygEM2-4	Crygm2d3 protein	_MK(cr)VTFFEDR_	2	1	MKVTFFEDR______		extracellular							IPR011024; IPR001064;	Gamma-crystallin-related; Beta/gamma crystallin;
Q9I8V1	actc1b	Actin, alpha 1, skeletal muscle	_MQK(cr)EITALAPSTMK_	317	1	GIADRMQKEITALAP	.......K.I.....	cytoskeleton		cytoskeleton; organelle; intracellular organelle; intracellular non-membrane-bounded organelle; cell part; intracellular; cytoplasm; intracellular part; cell; non-membrane-bounded organelle;	ATP binding; nucleoside phosphate binding; nucleoside binding; ion binding; nucleotide binding; organic cyclic compound binding; heterocyclic compound binding; ribonucleoside binding; carbohydrate derivative binding; ribonucleotide binding; binding; purine ribonucleoside binding; anion binding; purine ribonucleotide binding; adenyl ribonucleotide binding; adenyl nucleotide binding; purine nucleoside binding; purine nucleotide binding; small molecule binding; purine ribonucleoside triphosphate binding;	K10354				
Q7ZVI7	actba	Actin, cytoplasmic 1	_MQK(cr)EITSLAPSTMK_	315	1	GIADRMQKEITSLAP	.......K.I.....	cytoskeleton		cytoskeleton; organelle; intracellular organelle; intracellular non-membrane-bounded organelle; cell part; intracellular; cytoplasm; intracellular part; cell; non-membrane-bounded organelle;	ATP binding; nucleoside phosphate binding; nucleoside binding; ion binding; nucleotide binding; organic cyclic compound binding; heterocyclic compound binding; ribonucleoside binding; carbohydrate derivative binding; ribonucleotide binding; binding; purine ribonucleoside binding; anion binding; purine ribonucleotide binding; adenyl ribonucleotide binding; adenyl nucleotide binding; purine nucleoside binding; purine nucleotide binding; small molecule binding; purine ribonucleoside triphosphate binding;	K05692	ACTB_G1; actin beta/gamma 1	dre04510 Focal adhesion - Danio rerio (zebrafish); dre04145 Phagosome - Danio rerio (zebrafish); dre04530 Tight junction - Danio rerio (zebrafish); dre05132 Salmonella infection - Danio rerio (zebrafish); dre04810 Regulation of actin cytoskeleton - Danio rerio (zebrafish); dre04520 Adherens junction - Danio rerio (zebrafish);		
Q6PE34	zgc:65894	Uncharacterized protein	_MSMK(cr)EVDEQMLNVQNK_	324	1	FRGRMSMKEVDEQML	.......K.V.....	cytosol	cellular component biogenesis; cellular component organization; cellular macromolecular complex assembly; protein complex subunit organization; single-organism cellular process; single-organism process; microtubule-based process; macromolecular complex subunit organization; microtubule-based movement; cellular component assembly; macromolecular complex assembly; protein polymerization; movement of cell or subcellular component; protein complex biogenesis; cellular process; protein complex assembly; cellular protein complex assembly; cellular component organization or biogenesis;	intracellular organelle; microtubule; intracellular non-membrane-bounded organelle; cytoskeletal part; intracellular organelle part; cytoplasm; macromolecular complex; intracellular part; cell; organelle part; non-membrane-bounded organelle; organelle; cytoskeleton; microtubule cytoskeleton; intracellular; cell part; protein complex;	nucleoside phosphate binding; nucleoside binding; ion binding; hydrolase activity, acting on acid anhydrides; hydrolase activity; nucleotide binding; nucleoside-triphosphatase activity; organic cyclic compound binding; heterocyclic compound binding; ribonucleoside binding; carbohydrate derivative binding; pyrophosphatase activity; catalytic activity; ribonucleotide binding; binding; GTPase activity; purine ribonucleoside binding; anion binding; purine ribonucleotide binding; purine nucleoside binding; guanyl ribonucleotide binding; hydrolase activity, acting on acid anhydrides, in phosphorus-containing anhydrides; guanyl nucleotide binding; structural molecule activity; GTP binding; purine nucleotide binding; small molecule binding; purine ribonucleoside triphosphate binding;	K07375	TUBB; tubulin beta	dre04145 Phagosome - Danio rerio (zebrafish); dre04540 Gap junction - Danio rerio (zebrafish);	IPR003008; IPR023123; IPR018316; IPR008280;	Tubulin/FtsZ, GTPase domain; Tubulin, C-terminal; Tubulin/FtsZ, 2-layer sandwich domain; Tubulin/FtsZ, C-terminal;
Q1LYB7	rpl13a	OTTDARP00000009674	_MVVPAALK(cr)IVR_	127	1	MVVPAALKIVRLKPT	.......K.V.....	cytosol	cellular protein metabolic process; biosynthetic process; primary metabolic process; cellular metabolic process; organic substance biosynthetic process; cellular biosynthetic process; organic substance metabolic process; metabolic process; cellular macromolecule biosynthetic process; cellular macromolecule metabolic process; gene expression; macromolecule metabolic process; protein metabolic process; macromolecule biosynthetic process; translation; cellular process;	intracellular organelle; ribosome; intracellular non-membrane-bounded organelle; cytoplasm; intracellular organelle part; macromolecular complex; intracellular part; cell; organelle part; non-membrane-bounded organelle; organelle; large ribosomal subunit; ribosomal subunit; cell part; intracellular; ribonucleoprotein complex; cytoplasmic part;	structural constituent of ribosome; structural molecule activity;	K02872	RP-L13Ae; large subunit ribosomal protein L13Ae	dre03010 Ribosome - Danio rerio (zebrafish);	IPR023564;	Ribosomal protein L13 domain;

B8A568	myhz1.1	Uncharacterized protein	_NAEDK(cr)AK_	1756	1	EARNAEDKAKKAITD	......DK.......	nuclear		intracellular organelle; intracellular non-membrane-bounded organelle; cytoskeletal part; intracellular organelle part; macromolecular complex; intracellular part; cell; actin cytoskeleton; organelle part; non-membrane-bounded organelle; organelle; cytoskeleton; cell part; intracellular; myosin complex; protein complex;	ATP binding; motor activity; nucleoside phosphate binding; nucleoside binding; ion binding; hydrolase activity, acting on acid anhydrides; hydrolase activity; nucleotide binding; nucleoside-triphosphatase activity; organic cyclic compound binding; heterocyclic compound binding; ribonucleoside binding; carbohydrate derivative binding; pyrophosphatase activity; catalytic activity; ribonucleotide binding; binding; purine ribonucleoside binding; anion binding; purine ribonucleotide binding; adenyl ribonucleotide binding; adenyl nucleotide binding; purine nucleoside binding; hydrolase activity, acting on acid anhydrides, in phosphorus-containing anhydrides; purine nucleotide binding; small molecule binding; purine ribonucleoside triphosphate binding;	K10352	MYH; myosin heavy chain	dre04530 Tight junction - Danio rerio (zebrafish);	IPR002928; IPR004009; IPR001609; IPR027417; IPR027401;	Myosin tail; Myosin, N-terminal, SH3-like; Myosin head, motor domain; P-loop containing nucleoside triphosphate hydrolase; Myosin-like IQ motif-containing domain;
B8A561	myhz1.2	Uncharacterized protein	_NAEDK(cr)AK_	1756	1	EARNAEDKAKKAITD	......DK.......	nuclear		intracellular organelle; intracellular non-membrane-bounded organelle; cytoskeletal part; intracellular organelle part; macromolecular complex; intracellular part; cell; actin cytoskeleton; organelle part; non-membrane-bounded organelle; organelle; cytoskeleton; cell part; intracellular; myosin complex; protein complex;	ATP binding; motor activity; nucleoside phosphate binding; nucleoside binding; ion binding; hydrolase activity, acting on acid anhydrides; hydrolase activity; nucleotide binding; nucleoside-triphosphatase activity; organic cyclic compound binding; heterocyclic compound binding; ribonucleoside binding; carbohydrate derivative binding; pyrophosphatase activity; catalytic activity; ribonucleotide binding; binding; purine ribonucleoside binding; anion binding; purine ribonucleotide binding; adenyl ribonucleotide binding; adenyl nucleotide binding; purine nucleoside binding; hydrolase activity, acting on acid anhydrides, in phosphorus-containing anhydrides; purine nucleotide binding; small molecule binding; purine ribonucleoside triphosphate binding;	K10352	MYH; myosin heavy chain	dre04530 Tight junction - Danio rerio (zebrafish);	IPR002928; IPR004009; IPR001609; IPR027417; IPR027401;	Myosin tail; Myosin, N-terminal, SH3-like; Myosin head, motor domain; P-loop containing nucleoside triphosphate hydrolase; Myosin-like IQ motif-containing domain;
Q642Z0	atp2a1	Calcium-transporting ATPase	_NAESAIEALK(cr)EYEPEMGK_	120	1	ESAIEALKEYEPEMG		plasma membrane	nucleoside metabolic process; purine nucleoside metabolic process; purine ribonucleoside monophosphate metabolic process; nucleotide metabolic process; single-organism process; purine ribonucleoside biosynthetic process; nucleoside biosynthetic process; biosynthetic process; ribonucleoside monophosphate biosynthetic process; cellular metabolic process; phosphate-containing compound metabolic process; ribose phosphate biosynthetic process; metabolic process; carbohydrate derivative biosynthetic process; nucleoside phosphate metabolic process; ribose phosphate metabolic process; purine nucleoside triphosphate biosynthetic process; heterocycle biosynthetic process; purine ribonucleoside triphosphate biosynthetic process; small molecule metabolic process; ATP biosynthetic process; single-organism cellular process; purine ribonucleoside triphosphate metabolic process; organic cyclic compound metabolic process; purine-containing compound metabolic process; ribonucleoside triphosphate metabolic process; purine ribonucleoside monophosphate biosynthetic process; purine nucleoside biosynthetic process; organophosphate biosynthetic process; ribonucleoside monophosphate	intrinsic component of membrane; membrane; integral component of membrane; membrane part;	ATPase activity, coupled to transmembrane movement of ions; nucleoside phosphate binding; primary active transmembrane transporter activity; substrate-specific transporter activity; hydrolase activity, acting on acid anhydrides; hydrolase activity; substrate-specific transmembrane transporter activity; ATPase activity; organic cyclic compound binding; heterocyclic compound binding; metal ion transmembrane transporter activity; pyrophosphatase activity; cation-transporting ATPase activity; catalytic activity; ribonucleotide binding; ATPase activity, coupled to movement of substances; binding; purine ribonucleoside binding; cation binding; hydrolase activity, acting on acid anhydrides, catalyzing transmembrane movement of substances; anion binding; adenyl ribonucleotide binding; adenyl nucleotide binding; inorganic cation transmembrane transporter activity; hydrolase activity, acting on acid anhydrides, in phosphorus-containing anhydrides; transporter activity; purine nucleotide binding; purine ribonucleoside triphosphate binding; active transmembrane transporter activity; ATP binding; ion transmembrane transporter activity; ion binding; nucleoside binding; nucleotide binding; nucleoside-triphosphatase activity; ribonucleoside binding; P-P-bond-hydrolysis-driven transmembrane transporter activity; ATPase activity, coupled to transmembrane movement of ions, phosphorylative mechanism; divalent inorganic cation transmembrane transporter activity; carbohydrate derivative binding; calcium-transporting ATPase activity; ATPase activity, coupled to transmembrane movement of substances; ATPase activity, coupled; metal ion binding; calcium ion transmembrane transporter activity; purine ribonucleotide binding; purine nucleoside binding; cation transmembrane transporter activity; transmembrane transporter activity; small molecule binding;	K05853	ATP2A; Ca2+ transporting ATPase, sarcoplasmic/endoplasmic reticulum [EC:3.6.3.8]	dre04020 Calcium signaling pathway - Danio rerio (zebrafish);	IPR023298; IPR004014; IPR023299; IPR008250; IPR006068; IPR023214;	P-type ATPase, transmembrane domain; Cation-transporting P-type ATPase, N-terminal; P-type ATPase, cytoplasmic domain N; P-type ATPase, A domain; Cation-transporting P-type ATPase, C-terminal; HAD-like domain;
F1QFC0	hspa9	Uncharacterized protein	_NAK(cr)VLENAEGAR_	79	1	VMDGKNAKVLENAEG	.......K.L.....	mitochondria	protein folding; metabolic process; cellular protein metabolic process; cellular macromolecule metabolic process; macromolecule metabolic process; protein metabolic process; primary metabolic process; cellular process; cellular metabolic process; organic substance metabolic process;		ATP binding; nucleoside phosphate binding; nucleoside binding; ion binding; nucleotide binding; organic cyclic compound binding; heterocyclic compound binding; ribonucleoside binding; carbohydrate derivative binding; ribonucleotide binding; binding; purine ribonucleoside binding; anion binding; purine ribonucleotide binding; adenyl ribonucleotide binding; adenyl nucleotide binding; purine nucleoside binding; purine nucleotide binding; small molecule binding; purine ribonucleoside triphosphate binding;	K04043	dnaK; molecular chaperone DnaK	dre03018 RNA degradation - Danio rerio (zebrafish);	IPR029048; IPR029047;	Heat shock protein 70kD, C-terminal domain; Heat shock protein 70kD, peptide-binding domain;
Q29RA2	zgc:136908	Uncharacterized protein	_NAPAIIFIDELDAIAPK(cr)R_	314	1	ELDAIAPKREKTHGE	.......K.E.....	cytosol			ATP binding; nucleoside phosphate binding; nucleoside binding; ion binding; hydrolase activity, acting on acid anhydrides; hydrolase activity; nucleotide binding; nucleoside-triphosphatase activity; organic cyclic compound binding; heterocyclic compound binding; ribonucleoside binding; carbohydrate derivative binding; pyrophosphatase activity; catalytic activity; ribonucleotide binding; binding; purine ribonucleoside binding; anion binding; purine ribonucleotide binding; adenyl ribonucleotide binding; adenyl nucleotide binding; purine nucleoside binding; hydrolase activity, acting on acid anhydrides, in phosphorus-containing anhydrides; purine nucleotide binding; small molecule binding; purine ribonucleoside triphosphate binding;				IPR029067; IPR003959; IPR003593; IPR009010; IPR004201; IPR027417; IPR003338;	CDC48 domain 2-like; ATPase, AAA-type, core; AAA+ ATPase domain; Aspartate decarboxylase-like domain; CDC48, domain 2; P-loop containing nucleoside triphosphate hydrolase; CDC48, N-terminal subdomain;
B8A568	myhz1.1	Uncharacterized protein	_NFDK(cr)VLAEWK_	1453	1	KKQRNFDKVLAEWKQ	......DK.......	nuclear		intracellular organelle; intracellular non-membrane-bounded organelle; cytoskeletal part; intracellular organelle part; macromolecular complex; intracellular part; cell; actin cytoskeleton; organelle part; non-membrane-bounded organelle; organelle; cytoskeleton; cell part; intracellular; myosin complex; protein complex;	ATP binding; motor activity; nucleoside phosphate binding; nucleoside binding; ion binding; hydrolase activity, acting on acid anhydrides; hydrolase activity; nucleotide binding; nucleoside-triphosphatase activity; organic cyclic compound binding; heterocyclic compound binding; ribonucleoside binding; carbohydrate derivative binding; pyrophosphatase activity; catalytic activity; ribonucleotide binding; binding; purine ribonucleoside binding; anion binding; purine ribonucleotide binding; adenyl ribonucleotide binding; adenyl nucleotide binding; purine nucleoside binding; hydrolase activity, acting on acid anhydrides, in phosphorus-containing anhydrides; purine nucleotide binding; small molecule binding; purine ribonucleoside triphosphate binding;	K10352	MYH; myosin heavy chain	dre04530 Tight junction - Danio rerio (zebrafish);	IPR002928; IPR004009; IPR001609; IPR027417; IPR027401;	Myosin tail; Myosin, N-terminal, SH3-like; Myosin head, motor domain; P-loop containing nucleoside triphosphate hydrolase; Myosin-like IQ motif-containing domain;

B8A561	myhz1.2	Uncharacterized protein	_NFDK(cr)VLAEWK_	1453	1	KKQRNFDKVLAEWKQ	......DK.......	nuclear		intracellular organelle; intracellular non-membrane-bounded organelle; cytoskeletal part; intracellular organelle part; macromolecular complex; intracellular part; cell; actin cytoskeleton; organelle part; non-membrane-bounded organelle; organelle; cytoskeleton; cell part; intracellular; myosin complex; protein complex;	ATP binding; motor activity; nucleoside phosphate binding; nucleoside binding; ion binding; hydrolase activity, acting on acid anhydrides; hydrolase activity; nucleotide binding; nucleoside-triphosphatase activity; organic cyclic compound binding; heterocyclic compound binding; ribonucleoside binding; carbohydrate derivative binding; pyrophosphatase activity; catalytic activity; ribonucleotide binding; binding; purine ribonucleoside binding; anion binding; purine ribonucleotide binding; adenyl ribonucleotide binding; adenyl nucleotide binding; purine nucleoside binding; hydrolase activity, acting on acid anhydrides, in phosphorus-containing anhydrides; purine nucleotide binding; small molecule binding; purine ribonucleoside triphosphate binding;	K10352	MYH; myosin heavy chain	dre04530 Tight junction - Danio rerio (zebrafish);	IPR002928; IPR004009; IPR001609; IPR027417; IPR027401;	Myosin tail; Myosin, N-terminal, SH3-like; Myosin head, motor domain; P-loop containing nucleoside triphosphate hydrolase; Myosin-like IQ motif-containing domain;
Q9I8V0	pvalb2	Parvalbumin-2	_NFFAK(cr)VGLSAK_	33	1	NYKNFFAKVGLSAKS		cytosol			cation binding; metal ion binding; calcium ion binding; binding; ion binding;				IPR002048; IPR011992;	EF-hand domain; EF-hand domain pair;
Q0D2W2	tnni2a.4	Uncharacterized protein	_NIEDK(cr)AGMDGR_	162	1	WRKNIEDKAGMDGRK	......DK.......	mitochondria								
Q71N41	gamt	Guanidinoacetate N-methyltransferase	_NK(cr)YDNIDK_	179	1	WGELLKNKYDNIDKM	..E....K.......	cytosol	cellular amino acid biosynthetic process; organic acid biosynthetic process; single-organism biosynthetic process; small molecule metabolic process; single-organism cellular process; cellular modified amino acid biosynthetic process; nitrogen compound metabolic process; single-organism process; biosynthetic process; creatine metabolic process; alpha-amino acid metabolic process; primary metabolic process; organonitrogen compound biosynthetic process; cellular metabolic process; organic substance biosynthetic process; cellular biosynthetic process; organic substance metabolic process; carboxylic acid biosynthetic process; metabolic process; cellular amino acid metabolic process; cellular modified amino acid metabolic process; single-organism metabolic process; alpha-amino acid biosynthetic process; carboxylic acid metabolic process; small molecule biosynthetic process; organic acid metabolic process; cellular process; oxoacid metabolic process; creatine biosynthetic process; organonitrogen compound metabolic process;		transferase activity, transferring one-carbon groups; S-adenosylmethionine-dependent methyltransferase activity; methyltransferase activity; guanidinoacetate N-methyltransferase activity; catalytic activity; transferase activity;	K00542	GAMT; guanidinoacetate N-methyltransferase [EC:2.1.1.2]	dre00330 Arginine and proline metabolism - Danio rerio (zebrafish); dre01100 Metabolic pathways - Danio rerio (zebrafish); dre00260 Glycine, serine and threonine metabolism - Danio rerio (zebrafish);	IPR026480; IPR029063;	Arginine N-methyltransferase 2-like domain; S-adenosyl-L-methionine-dependent methyltransferase-like;
I3ITD9	pabpc1b	Uncharacterized protein	_NLDK(cr)SIDNK_	108	1	IFIKNLDKSIDNKAL	......DK.......	cytosol,nuclear			organic cyclic compound binding; heterocyclic compound binding; nucleoside phosphate binding; binding; nucleic acid binding; nucleotide binding; small molecule binding;				IPR000504; IPR012677;	RNA recognition motif domain; Nucleotide-binding, alpha-beta plait;
Q7T334	mdh2	Malate dehydrogenase	_NLGLGK(cr)LSAFEEK_	306	1	EKNLGLGKLSAFEEK		mitochondria	tricarboxylic acid metabolic process; dicarboxylic acid metabolic process; oxidation-reduction process; small molecule metabolic process; tricarboxylic acid cycle; single-organism cellular process; single-organism process; primary metabolic process; carbohydrate metabolic process; cellular respiration; cellular metabolic process; organic substance metabolic process; citrate metabolic process; metabolic process; generation of precursor metabolites and energy; cellular carbohydrate metabolic process; aerobic respiration; single-organism metabolic process; energy derivation by oxidation of organic compounds; malate metabolic process; carboxylic acid metabolic process; organic acid metabolic process; cellular process; oxoacid metabolic process;		organic cyclic compound binding; heterocyclic compound binding; oxidoreductase activity, acting on CH-OH group of donors; oxidoreductase activity, acting on the CH-OH group of donors, NAD or NADP as acceptor; nucleoside phosphate binding; catalytic activity; binding; nucleotide binding; malate dehydrogenase activity; L-malate dehydrogenase activity; small molecule binding; oxidoreductase activity;	K00026	MDH2; malate dehydrogenase [EC:1.1.1.37]	dre01200 Carbon metabolism - Danio rerio (zebrafish); dre01100 Metabolic pathways - Danio rerio (zebrafish); dre00620 Pyruvate metabolism - Danio rerio (zebrafish); dre00630 Glyoxylate and dicarboxylate metabolism - Danio rerio (zebrafish); dre00020 Citrate cycle (TCA cycle) - Danio rerio (zebrafish); dre00270 Cysteine and methionine metabolism - Danio rerio (zebrafish);	IPR022383; IPR016040; IPR001236; IPR015955;	Lactate/malate dehydrogenase, C-terminal; NAD(P)-binding domain; Lactate/malate dehydrogenase, N-terminal; Lactate dehydrogenase/glycoside hydrolase, family 4, C-terminal;
E9QF19	pgk1	Phosphoglycerate kinase	_NLLGK(cr)DVQFLK_	100	1	ELKNLLGKDVQFLKD	.......K.V.....	cytosol	single-organism carbohydrate catabolic process; glycolytic process; single-organism catabolic process; small molecule metabolic process; single-organism cellular process; single-organism process; carbohydrate metabolic process; primary metabolic process; cellular metabolic process; organic substance metabolic process; carbohydrate catabolic process; single-organism carbohydrate metabolic process; organic substance catabolic process; metabolic process; pyruvate metabolic process; generation of precursor metabolites and energy; single-organism metabolic process; carboxylic acid metabolic process; monocarboxylic acid metabolic process; organic acid metabolic process; cellular process; catabolic process; oxoacid metabolic process;		phosphotransferase activity, carboxyl group as acceptor; kinase activity; catalytic activity; transferase activity; phosphoglycerate kinase activity; transferase activity, transferring phosphorus-containing groups;				IPR015824;	Phosphoglycerate kinase, N-terminal;
F1R431	-	Uncharacterized protein	_NLQK(cr)TVK_	603	1	QTSKNLQKTVKLPRL	.......K.V.....	cytosol				K05768	GSN; gelsolin	dre04810 Regulation of actin cytoskeleton - Danio rerio (zebrafish);	IPR029006; IPR007123;	ADF-H/Gelsolin-like domain; Gelsolin-like domain;

A2BGX6	myhc4	Uncharacterized protein	_NLTEEM(ox)ASQDESIAK(cr)LTK_	993	1	SQDESIAKLTKEKKA		cytosol		intracellular organelle; intracellular non-membrane-bounded organelle; cytoskeletal part; intracellular organelle part; macromolecular complex; intracellular part; cell; actin cytoskeleton; organelle part; non-membrane-bounded organelle; organelle; cytoskeleton; cell part; intracellular; myosin complex; protein complex;	ATP binding; motor activity; nucleoside phosphate binding; nucleoside binding; ion binding; hydrolase activity, acting on acid anhydrides; hydrolase activity; nucleotide binding; nucleoside-triphosphatase activity; organic cyclic compound binding; heterocyclic compound binding; ribonucleoside binding; carbohydrate derivative binding; pyrophosphatase activity; catalytic activity; ribonucleotide binding; binding; purine ribonucleoside binding; anion binding; purine ribonucleotide binding; adenyl ribonucleotide binding; adenyl nucleotide binding; purine nucleoside binding; hydrolase activity, acting on acid anhydrides, in phosphorus-containing anhydrides; purine nucleotide binding; small molecule binding; purine ribonucleoside triphosphate binding;	K10352	MYH; myosin heavy chain	dre04530 Tight junction - Danio rerio (zebrafish);	IPR002928; IPR004009; IPR027417; IPR001609; IPR027401;	Myosin tail; Myosin, N-terminal, SH3-like; P-loop containing nucleoside triphosphate hydrolase; Myosin head, motor domain; Myosin-like IQ motif-containing domain;
B8A568	myhz1.1	Uncharacterized protein	_NLTEEMAAQDESIGK(cr)LTK_	993	1	AQDESIGKLTKEKKA		nuclear		intracellular organelle; intracellular non-membrane-bounded organelle; cytoskeletal part; intracellular organelle part; macromolecular complex; intracellular part; cell; actin cytoskeleton; organelle part; non-membrane-bounded organelle; organelle; cytoskeleton; cell part; intracellular; myosin complex; protein complex;	ATP binding; motor activity; nucleoside phosphate binding; nucleoside binding; ion binding; hydrolase activity, acting on acid anhydrides; hydrolase activity; nucleotide binding; nucleoside-triphosphatase activity; organic cyclic compound binding; heterocyclic compound binding; ribonucleoside binding; carbohydrate derivative binding; pyrophosphatase activity; catalytic activity; ribonucleotide binding; binding; purine ribonucleoside binding; anion binding; purine ribonucleotide binding; adenyl ribonucleotide binding; adenyl nucleotide binding; purine nucleoside binding; hydrolase activity, acting on acid anhydrides, in phosphorus-containing anhydrides; purine nucleotide binding; small molecule binding; purine ribonucleoside triphosphate binding;	K10352	MYH; myosin heavy chain	dre04530 Tight junction - Danio rerio (zebrafish);	IPR002928; IPR004009; IPR001609; IPR027417; IPR027401;	Myosin tail; Myosin, N-terminal, SH3-like; Myosin head, motor domain; P-loop containing nucleoside triphosphate hydrolase; Myosin-like IQ motif-containing domain;
B8A561	myhz1.2	Uncharacterized protein	_NLTEEMAAQDESIGK(cr)LTK_	993	1	AQDESIGKLTKEKKA		nuclear		intracellular organelle; intracellular non-membrane-bounded organelle; cytoskeletal part; intracellular organelle part; macromolecular complex; intracellular part; cell; actin cytoskeleton; organelle part; non-membrane-bounded organelle; organelle; cytoskeleton; cell part; intracellular; myosin complex; protein complex;	ATP binding; motor activity; nucleoside phosphate binding; nucleoside binding; ion binding; hydrolase activity, acting on acid anhydrides; hydrolase activity; nucleotide binding; nucleoside-triphosphatase activity; organic cyclic compound binding; heterocyclic compound binding; ribonucleoside binding; carbohydrate derivative binding; pyrophosphatase activity; catalytic activity; ribonucleotide binding; binding; purine ribonucleoside binding; anion binding; purine ribonucleotide binding; adenyl ribonucleotide binding; adenyl nucleotide binding; purine nucleoside binding; hydrolase activity, acting on acid anhydrides, in phosphorus-containing anhydrides; purine nucleotide binding; small molecule binding; purine ribonucleoside triphosphate binding;	K10352	MYH; myosin heavy chain	dre04530 Tight junction - Danio rerio (zebrafish);	IPR002928; IPR004009; IPR001609; IPR027417; IPR027401;	Myosin tail; Myosin, N-terminal, SH3-like; Myosin head, motor domain; P-loop containing nucleoside triphosphate hydrolase; Myosin-like IQ motif-containing domain;
Q6IQX1	myhz2	Uncharacterized protein (Fragment)	_NLTEEMASQDESIAK(cr)LTK_	993	1	SQDESIAKLTKEKKA		cytosol	response to stress; response to stimulus;	intracellular organelle; intracellular non-membrane-bounded organelle; cytoskeletal part; intracellular organelle part; macromolecular complex; intracellular part; cell; actin cytoskeleton; organelle part; non-membrane-bounded organelle; organelle; cytoskeleton; cell part; intracellular; myosin complex; protein complex;	ATP binding; motor activity; nucleoside phosphate binding; nucleoside binding; ion binding; hydrolase activity, acting on acid anhydrides; hydrolase activity; nucleotide binding; nucleoside-triphosphatase activity; organic cyclic compound binding; heterocyclic compound binding; ribonucleoside binding; carbohydrate derivative binding; pyrophosphatase activity; catalytic activity; ribonucleotide binding; binding; purine ribonucleoside binding; anion binding; purine ribonucleotide binding; adenyl ribonucleotide binding; adenyl nucleotide binding; purine nucleoside binding; hydrolase activity, acting on acid anhydrides, in phosphorus-containing anhydrides; purine nucleotide binding; small molecule binding; purine ribonucleoside triphosphate binding;	K10352	MYH; myosin heavy chain	dre04530 Tight junction - Danio rerio (zebrafish);	IPR002928; IPR004009; IPR001609; IPR027417; IPR027401;	Myosin tail; Myosin, N-terminal, SH3-like; Myosin head, motor domain; P-loop containing nucleoside triphosphate hydrolase; Myosin-like IQ motif-containing domain;
Q6NYR4	hspa8	Heat shock cognate 71 kDa protein	_NQVAM(ox)NPTNTVFDAK(cr)R_	71	1	TNTVFDAKRLIGRRF	.......K.L.....	cytosol	response to stress; response to stimulus;		ATP binding; nucleoside phosphate binding; nucleoside binding; ion binding; nucleotide binding; organic cyclic compound binding; heterocyclic compound binding; ribonucleoside binding; carbohydrate derivative binding; ribonucleotide binding; binding; purine ribonucleoside binding; anion binding; purine ribonucleotide binding; adenyl ribonucleotide binding; adenyl nucleotide binding; purine nucleoside binding; purine nucleotide binding; small molecule binding; purine ribonucleoside triphosphate binding;	K03283	HSPA1_8; heat shock 70kDa protein 1/8	dre04010 MAPK signaling pathway - Danio rerio (zebrafish); dre04141 Protein processing in endoplasmic reticulum - Danio rerio (zebrafish); dre04144 Endocytosis - Danio rerio (zebrafish); dre03040 Spliceosome - Danio rerio (zebrafish);	IPR029048; IPR029047;	Heat shock protein 70kD, C-terminal domain; Heat shock protein 70kD, peptide-binding domain;
B8A568	myhz1.1	Uncharacterized protein	_NSYEETLDQLETLK(cr)R_	1501	1	LDQLETLKRENKNLQ	.......K.E.....	nuclear		intracellular organelle; intracellular non-membrane-bounded organelle; cytoskeletal part; intracellular organelle part; macromolecular complex; intracellular part; cell; actin cytoskeleton; organelle part; non-membrane-bounded organelle; organelle; cytoskeleton; cell part; intracellular; myosin complex; protein complex;	ATP binding; motor activity; nucleoside phosphate binding; nucleoside binding; ion binding; hydrolase activity, acting on acid anhydrides; hydrolase activity; nucleotide binding; nucleoside-triphosphatase activity; organic cyclic compound binding; heterocyclic compound binding; ribonucleoside binding; carbohydrate derivative binding; pyrophosphatase activity; catalytic activity; ribonucleotide binding; binding; purine ribonucleoside binding; anion binding; purine ribonucleotide binding; adenyl ribonucleotide binding; adenyl nucleotide binding; purine nucleoside binding; hydrolase activity, acting on acid anhydrides, in phosphorus-containing anhydrides; purine nucleotide binding; small molecule binding; purine ribonucleoside triphosphate binding;	K10352	MYH; myosin heavy chain	dre04530 Tight junction - Danio rerio (zebrafish);	IPR002928; IPR004009; IPR001609; IPR027417; IPR027401;	Myosin tail; Myosin, N-terminal, SH3-like; Myosin head, motor domain; P-loop containing nucleoside triphosphate hydrolase; Myosin-like IQ motif-containing domain;
B8A561	myhz1.2	Uncharacterized protein	_NSYEETLDQLETLK(cr)R_	1501	1	LDQLETLKRENKNLQ	.......K.E.....	nuclear		intracellular organelle; intracellular non-membrane-bounded organelle; cytoskeletal part; intracellular organelle part; macromolecular complex; intracellular part; cell; actin cytoskeleton; organelle part; non-membrane-bounded organelle; organelle; cytoskeleton; cell part; intracellular; myosin complex; protein complex;	ATP binding; motor activity; nucleoside phosphate binding; nucleoside binding; ion binding; hydrolase activity, acting on acid anhydrides; hydrolase activity; nucleotide binding; nucleoside-triphosphatase activity; organic cyclic compound binding; heterocyclic compound binding; ribonucleoside binding; carbohydrate derivative binding; pyrophosphatase activity; catalytic activity; ribonucleotide binding; binding; purine ribonucleoside binding; anion binding; purine ribonucleotide binding; adenyl ribonucleotide binding; adenyl nucleotide binding; purine nucleoside binding; hydrolase activity, acting on acid anhydrides, in phosphorus-containing anhydrides; purine nucleotide binding; small molecule binding; purine ribonucleoside triphosphate binding;	K10352	MYH; myosin heavy chain	dre04530 Tight junction - Danio rerio (zebrafish);	IPR002928; IPR004009; IPR001609; IPR027417; IPR027401;	Myosin tail; Myosin, N-terminal, SH3-like; Myosin head, motor domain; P-loop containing nucleoside triphosphate hydrolase; Myosin-like IQ motif-containing domain;

F1QIR4	-	Uncharacterized protein	_NSYEEVLDQLETMK(cr)R_	1502	1	LDQLETMKRENKNLQ	.......K.E.....	cytosol	cellular component biogenesis; muscle cell development; single-multicellular organism process; cellular component organization; muscle system process; cellular developmental process; cellular component morphogenesis; single-organism process; actin filament organization; cell development; anatomical structure morphogenesis; cell differentiation; actin cytoskeleton organization; muscle contraction; cellular component assembly; organelle assembly; macromolecular complex assembly; myofibril assembly; musculoskeletal movement; slow-twitch skeletal muscle fiber contraction; muscle structure development; cytoskeleton organization; anatomical structure formation involved in morphogenesis; cellular process; striated muscle myosin thick filament assembly; protein complex assembly; cellular protein complex assembly; skeletal muscle thin filament assembly; developmental process; cellular macromolecular complex assembly; striated muscle cell differentiation; actomyosin structure organization; system process; protein complex subunit organization; multicellular organismal movement; single-organism cellular process; cellular component assembly involved in morphogenesis; striated muscle contraction; myosin filament	intracellular organelle; intracellular non-membrane-bounded organelle; cytoskeletal part; intracellular organelle part; macromolecular complex; intracellular part; cell; actin cytoskeleton; organelle part; non-membrane-bounded organelle; organelle; cytoskeleton; cell part; intracellular; myosin complex; protein complex;	ATP binding; motor activity; nucleoside phosphate binding; nucleoside binding; ion binding; hydrolase activity, acting on acid anhydrides; hydrolase activity; nucleotide binding; nucleoside-triphosphatase activity; organic cyclic compound binding; heterocyclic compound binding; ribonucleoside binding; carbohydrate derivative binding; pyrophosphatase activity; catalytic activity; ribonucleotide binding; binding; purine ribonucleoside binding; anion binding; purine ribonucleotide binding; adenyl ribonucleotide binding; adenyl nucleotide binding; purine nucleoside binding; hydrolase activity, acting on acid anhydrides, in phosphorus-containing anhydrides; purine nucleotide binding; small molecule binding; purine ribonucleoside triphosphate binding;	K10352	MYH; myosin heavy chain	dre04530 Tight junction - Danio rerio (zebrafish);	IPR002928; IPR004009; IPR027417; IPR001609; IPR027401;	Myosin tail; Myosin, N-terminal, SH3-like; P-loop containing nucleoside triphosphate hydrolase; Myosin head, motor domain; Myosin-like IQ motif-containing domain;
E7F4R9	-	Isocitrate dehydrogenase [NADP]	_NTILK(cr)AYDGR_	252	1	STKNTILKAYDGRFK		mitochondria	tricarboxylic acid metabolic process; oxidation-reduction process; small molecule metabolic process; tricarboxylic acid cycle; single-organism cellular process; single-organism process; isocitrate metabolic process; primary metabolic process; cellular respiration; cellular metabolic process; organic substance metabolic process; citrate metabolic process; metabolic process; generation of precursor metabolites and energy; aerobic respiration; single-organism metabolic process; energy derivation by oxidation of organic compounds; carboxylic acid metabolic process; organic acid metabolic process; cellular process; oxoacid metabolic process;		magnesium ion binding; nucleoside phosphate binding; ion binding; isocitrate dehydrogenase activity; nucleotide binding; cofactor binding; oxidoreductase activity; oxidoreductase activity, acting on CH-OH group of donors; heterocyclic compound binding; organic cyclic compound binding; oxidoreductase activity, acting on the CH-OH group of donors, NAD or NADP as acceptor; catalytic activity; NAD binding; binding; isocitrate dehydrogenase (NADP+) activity; metal ion binding; cation binding; small molecule binding; coenzyme binding;	K00031	IDH1; isocitrate dehydrogenase [EC:1.1.1.42]	dre01210 2-Oxocarboxylic acid metabolism - Danio rerio (zebrafish); dre01230 Biosynthesis of amino acids - Danio rerio (zebrafish); dre01200 Carbon metabolism - Danio rerio (zebrafish); dre01100 Metabolic pathways - Danio rerio (zebrafish); dre00480 Glutathione metabolism - Danio rerio (zebrafish); dre04146 Peroxisome - Danio rerio (zebrafish); dre00020 Citrate cycle (TCA cycle) - Danio rerio (zebrafish);	IPR024084;	Isopropylmalate dehydrogenase-like domain;
Q1LYB7	rpl13a	OTTDARP00000009674	_NVESK(cr)IAVYTDVLK_	190	1	AEKNVESKIAVYTDV		cytosol	cellular protein metabolic process; biosynthetic process; primary metabolic process; cellular metabolic process; organic substance biosynthetic process; cellular biosynthetic process; organic substance metabolic process; metabolic process; cellular macromolecule biosynthetic process; cellular macromolecule metabolic process; gene expression; macromolecule metabolic process; protein metabolic process; macromolecule biosynthetic process; translation; cellular process;	intracellular organelle; ribosome; intracellular non-membrane-bounded organelle; cytoplasm; intracellular organelle part; macromolecular complex; intracellular part; cell; organelle part; non-membrane-bounded organelle; organelle; large ribosomal subunit; ribosomal subunit; cell part; intracellular; ribonucleoprotein complex; cytoplasmic part;	structural constituent of ribosome; structural molecule activity;	K02872	RP-L13Ae; large subunit ribosomal protein L13Ae	dre03010 Ribosome - Danio rerio (zebrafish);	IPR023564;	Ribosomal protein L13 domain;
B8A561	myhz1.2	Uncharacterized protein	_NVQAQLK(cr)DAQLHLDDAVR_	1653	1	RNVQAQLKDAQLHLD		nuclear		intracellular organelle; intracellular non-membrane-bounded organelle; cytoskeletal part; intracellular organelle part; macromolecular complex; intracellular part; cell; actin cytoskeleton; organelle part; non-membrane-bounded organelle; organelle; cytoskeleton; cell part; intracellular; myosin complex; protein complex;	ATP binding; motor activity; nucleoside phosphate binding; nucleoside binding; ion binding; hydrolase activity, acting on acid anhydrides; hydrolase activity; nucleotide binding; nucleoside-triphosphatase activity; organic cyclic compound binding; heterocyclic compound binding; ribonucleoside binding; carbohydrate derivative binding; pyrophosphatase activity; catalytic activity; ribonucleotide binding; binding; purine ribonucleoside binding; anion binding; purine ribonucleotide binding; adenyl ribonucleotide binding; adenyl nucleotide binding; purine nucleoside binding; hydrolase activity, acting on acid anhydrides, in phosphorus-containing anhydrides; purine nucleotide binding; small molecule binding; purine ribonucleoside triphosphate binding;	K10352	MYH; myosin heavy chain	dre04530 Tight junction - Danio rerio (zebrafish);	IPR002928; IPR004009; IPR001609; IPR027417; IPR027401;	Myosin tail; Myosin, N-terminal, SH3-like; Myosin head, motor domain; P-loop containing nucleoside triphosphate hydrolase; Myosin-like IQ motif-containing domain;
Q803X7	nap1l1	Nap1l1 protein	_NVTLK(cr)TIK_	263	1	KGKNVTLKTIKKKQK	.......K.I.....	nuclear	cellular component biogenesis; cellular component organization; nucleosome organization; cellular macromolecular complex assembly; chromatin assembly or disassembly; protein complex subunit organization; macromolecular complex subunit organization; protein-DNA complex assembly; DNA packaging; nucleosome assembly; chromosome organization; cellular component assembly; macromolecular complex assembly; organelle organization; chromatin organization; protein complex biogenesis; chromatin assembly; cellular process; protein complex assembly; cellular component organization or biogenesis; protein-DNA complex subunit organization; DNA conformation change;	organelle; intracellular membrane-bounded organelle; intracellular organelle; nucleus; cell part; intracellular; intracellular part; membrane-bounded organelle; cell;		K11279				

Q05AK8	si:ch73-368j24.9	Histone H2B	_PEPAK(cr)TAPK_	6	1	__MPEPAKTAPKKGS		nuclear	cellular component biogenesis; cellular component organization; nucleosome organization; cellular macromolecular complex assembly; chromatin assembly or disassembly; protein complex subunit organization; macromolecular complex subunit organization; protein-DNA complex assembly; DNA packaging; nucleosome assembly; chromosome organization; cellular component assembly; macromolecular complex assembly; organelle organization; chromatin organization; protein complex biogenesis; chromatin assembly; cellular process; protein complex assembly; cellular component organization or biogenesis; protein-DNA complex subunit organization; DNA conformation change;	intracellular organelle; intracellular non-membrane-bounded organelle; intracellular organelle part; macromolecular complex; intracellular part; cell; organelle part; DNA bending complex; chromosomal part; nucleosome; non-membrane-bounded organelle; intracellular membrane-bounded organelle; organelle; nucleus; chromatin; cell part; intracellular; chromosome; membrane-bounded organelle; protein complex; protein-DNA complex; DNA packaging complex;	organic cyclic compound binding; heterocyclic compound binding; DNA binding; binding; nucleic acid binding;	K11252			IPR009072; IPR007125;	Histone-fold; Histone core;
Q6PBW7	rps19	Ribosomal protein S19	_PGGVTVK(cr)DVNQQEFVR_	8	1	MPGGVTVKDVNQQEF	.......K.V.....	cytosol	cellular protein metabolic process; biosynthetic process; primary metabolic process; cellular metabolic process; organic substance biosynthetic process; cellular biosynthetic process; organic substance metabolic process; metabolic process; cellular macromolecule biosynthetic process; cellular macromolecule metabolic process; gene expression; macromolecule metabolic process; protein metabolic process; macromolecule biosynthetic process; translation; cellular process;	ribosome; organelle; intracellular organelle; intracellular non-membrane-bounded organelle; cell part; intracellular; cytoplasm; macromolecular complex; intracellular part; cell; ribonucleoprotein complex; cytoplasmic part; non-membrane-bounded organelle;	structural constituent of ribosome; structural molecule activity;	K02966	RP-S19e; small subunit ribosomal protein S19e	dre03010 Ribosome - Danio rerio (zebrafish);		
F1QTL9	hnrnpa0b	Uncharacterized protein	_PHVVDGK(cr)NVEVK_	76	1	RPHVVDGKNVEVKRA	.......K.V.....	nuclear			organic cyclic compound binding; heterocyclic compound binding; nucleoside phosphate binding; binding; nucleic acid binding; nucleotide binding; small molecule binding;	K12894			IPR000504; IPR012677;	RNA recognition motif domain; Nucleotide-binding, alpha-[illegible]
Q6PH15	slc1a2b	Amino acid transporter	_PK(cr)HVEVR_	3	1	_____MPKHVEVRMH		plasma membrane		membrane;	organic acid transmembrane transporter activity; dicarboxylic acid transmembrane transporter activity; solute:cation symporter activity; active transmembrane transporter activity; secondary active transmembrane transporter activity; ion transmembrane transporter activity; symporter activity; solute:sodium symporter activity; substrate-specific transporter activity; monovalent inorganic cation transmembrane transporter activity; substrate-specific transmembrane transporter activity; metal ion transmembrane transporter activity; sodium:dicarboxylate symporter activity; anion:cation symporter activity; organic acid:sodium symporter activity; inorganic cation transmembrane transporter activity; sodium ion transmembrane transporter activity; organic anion transmembrane transporter activity; cation transmembrane transporter activity; anion transmembrane transporter activity; transporter activity; transmembrane transporter activity; carboxylic acid transmembrane transporter activity;	K05613				
Q6NYR4	hspa8	Heat shock cognate 71 kDa protein	_PK(cr)VQVEYK_	102	1	INDNSRPKVQVEYKG		cytosol	response to stress; response to stimulus;		ATP binding; nucleoside phosphate binding; nucleoside binding; ion binding; nucleotide binding; organic cyclic compound binding; heterocyclic compound binding; ribonucleoside binding; carbohydrate derivative binding; ribonucleotide binding; binding; purine ribonucleoside binding; anion binding; purine ribonucleotide binding; adenyl ribonucleotide binding; adenyl nucleotide binding; purine nucleoside binding; purine nucleotide binding; small molecule binding; purine ribonucleoside triphosphate binding;	K03283	HSPA1_8; heat shock 70kDa protein 1/8	dre04010 MAPK signaling pathway - Danio rerio (zebrafish); dre04141 Protein processing in endoplasmic reticulum - Danio rerio (zebrafish); dre04144 Endocytosis - Danio rerio (zebrafish); dre03040 Spliceosome - Danio rerio [illegible]	IPR029048; IPR029047;	Heat shock protein 70kD, C-terminal domain; Heat shock protein 70kD, peptide-binding domain;
B8A568	myhz1.1	Uncharacterized protein	_QAAEAQK(cr)QLR_	1643	1	RQAAEAQKQLRNVQA	.......K.L.....	nuclear		intracellular organelle; intracellular non-membrane-bounded organelle; cytoskeletal part; intracellular organelle part; macromolecular complex; intracellular part; cell; actin cytoskeleton; organelle part; non-membrane-bounded organelle; organelle; cytoskeleton; cell part; intracellular; myosin complex; protein complex;	ATP binding; motor activity; nucleoside phosphate binding; nucleoside binding; ion binding; hydrolase activity, acting on acid anhydrides; hydrolase activity; nucleotide binding; nucleoside-triphosphatase activity; organic cyclic compound binding; heterocyclic compound binding; ribonucleoside binding; carbohydrate derivative binding; pyrophosphatase activity; catalytic activity; ribonucleotide binding; binding; purine ribonucleoside binding; anion binding; purine ribonucleotide binding; adenyl ribonucleotide binding; adenyl nucleotide binding; purine nucleoside binding; hydrolase activity, acting on acid anhydrides, in phosphorus-containing anhydrides; purine nucleotide binding; small molecule binding; purine ribonucleoside triphosphate binding;	K10352	MYH; myosin heavy chain	dre04530 Tight junction - Danio rerio (zebrafish);	IPR002928; IPR004009; IPR001609; IPR027417; IPR027401;	Myosin tail; Myosin, N-terminal, SH3-like; Myosin head, motor domain; P-loop containing nucleoside triphosphate hydrolase; Myosin-like IQ motif-containing domain;
B8A561	myhz1.2	Uncharacterized protein	_QAAEAQK(cr)QLR_	1643	1	RQAAEAQKQLRNVQA	.......K.L.....	nuclear		intracellular organelle; intracellular non-membrane-bounded organelle; cytoskeletal part; intracellular organelle part; macromolecular complex; intracellular part; cell; actin cytoskeleton; organelle part; non-membrane-bounded organelle; organelle; cytoskeleton; cell part; intracellular; myosin complex; protein complex;	ATP binding; motor activity; nucleoside phosphate binding; nucleoside binding; ion binding; hydrolase activity, acting on acid anhydrides; hydrolase activity; nucleotide binding; nucleoside-triphosphatase activity; organic cyclic compound binding; heterocyclic compound binding; ribonucleoside binding; carbohydrate derivative binding; pyrophosphatase activity; catalytic activity; ribonucleotide binding; binding; purine ribonucleoside binding; anion binding; purine ribonucleotide binding; adenyl ribonucleotide binding; adenyl nucleotide binding; purine nucleoside binding; hydrolase activity, acting on acid anhydrides, in phosphorus-containing anhydrides; purine nucleotide binding; small molecule binding; purine ribonucleoside triphosphate binding;	K10352	MYH; myosin heavy chain	dre04530 Tight junction - Danio rerio (zebrafish);	IPR002928; IPR004009; IPR001609; IPR027417; IPR027401;	Myosin tail; Myosin, N-terminal, SH3-like; Myosin head, motor domain; P-loop containing nucleoside triphosphate hydrolase; Myosin-like IQ motif-containing domain;
B8A568	myhz1.1	Uncharacterized protein	_QAFTQQIEELK(cr)R_	1318	1	TQQIEELKRQIEEEV		nuclear		intracellular organelle; intracellular non-membrane-bounded organelle; cytoskeletal part; intracellular organelle part; macromolecular complex; intracellular part; cell; actin cytoskeleton; organelle part; non-membrane-bounded organelle; organelle; cytoskeleton; cell part; intracellular; myosin complex; protein complex;	ATP binding; motor activity; nucleoside phosphate binding; nucleoside binding; ion binding; hydrolase activity, acting on acid anhydrides; hydrolase activity; nucleotide binding; nucleoside-triphosphatase activity; organic cyclic compound binding; heterocyclic compound binding; ribonucleoside binding; carbohydrate derivative binding; pyrophosphatase activity; catalytic activity; ribonucleotide binding; binding; purine ribonucleoside binding; anion binding; purine ribonucleotide binding; adenyl ribonucleotide binding; adenyl nucleotide binding; purine nucleoside binding; hydrolase activity, acting on acid anhydrides, in phosphorus-containing anhydrides; purine nucleotide binding; small molecule binding; purine ribonucleoside triphosphate binding;	K10352	MYH; myosin heavy chain	dre04530 Tight junction - Danio rerio (zebrafish);	IPR002928; IPR004009; IPR001609; IPR027417; IPR027401;	Myosin tail; Myosin, N-terminal, SH3-like; Myosin head, motor domain; P-loop containing nucleoside triphosphate hydrolase; Myosin-like IQ motif-containing domain;

B8A561	myhz1.2	Uncharacterized protein	_QAFTQQIEELK(cr)R_	1318	1	TQQIEELKRQIEEEV		nuclear		intracellular organelle; intracellular non-membrane-bounded organelle; cytoskeletal part; intracellular organelle part; macromolecular complex; intracellular part; cell; actin cytoskeleton; organelle part; non-membrane-bounded organelle; organelle; cytoskeleton; cell part; intracellular; myosin complex; protein complex;	ATP binding; motor activity; nucleoside phosphate binding; nucleoside binding; ion binding; hydrolase activity, acting on acid anhydrides; hydrolase activity; nucleotide binding; nucleoside-triphosphatase activity; organic cyclic compound binding; heterocyclic compound binding; ribonucleoside binding; carbohydrate derivative binding; pyrophosphatase activity; catalytic activity; ribonucleotide binding; binding; purine ribonucleoside binding; anion binding; purine ribonucleotide binding; adenyl ribonucleotide binding; adenyl nucleotide binding; purine nucleoside binding; hydrolase activity, acting on acid anhydrides, in phosphorus-containing anhydrides; purine nucleotide binding; small molecule binding; purine ribonucleoside triphosphate binding;	K10352	MYH; myosin heavy chain	dre04530 Tight junction - Danio rerio (zebrafish);	IPR002928; IPR004009; IPR001609; IPR027417; IPR027401;	Myosin tail; Myosin, N-terminal, SH3-like; Myosin head, motor domain; P-loop containing nucleoside triphosphate hydrolase; Myosin-like IQ motif-containing domain;
Q6PE25	eef1g	Elongation factor 1-gamma	_QATEQAK(cr)EEVK_	132	1	KQATEQAKEEVKRVL	.......K.E.....	nuclear		cell part; intracellular; cytoplasm; macromolecular complex; intracellular part; protein complex; cell; cytoplasmic part; eukaryotic translation elongation factor 1 complex;	organic cyclic compound binding; heterocyclic compound binding; RNA binding; translation factor activity, nucleic acid binding; translation elongation factor activity; binding; nucleic acid binding;	K03233			IPR004046; IPR010987; IPR001662; IPR004045; IPR012336;	Glutathione S-transferase, C-terminal; Glutathione S-transferase, C-terminal-like; Translation elongation factor EF1B, gamma chain, conserved; Glutathione S-transferase, N-terminal; Thioredoxin-like fold;
Q5TZ29	apobb.1	Uncharacterized protein	_QFVANFDQEK(cr)FTEDVK_	2123	1	VANFDQEKFTEDVKN		endoplasmic reticulum	response to chemical; response to stimulus;		lipid transporter activity; substrate-specific transporter activity; transporter activity;				IPR011030; IPR015817; IPR015818; IPR001747; IPR015819; IPR015816; IPR016024; IPR015255; IPR009454;	Vitellinogen, superhelical; Vitellinogen, open beta-sheet, subdomain 1; Vitellinogen, open beta-sheet, subdomain 2; Lipid transport protein, N-terminal; Lipid transport protein, beta-sheet shell; Vitellinogen, beta-sheet N-terminal; Armadillo-type fold; Vitellinogen, open beta-sheet; Lipid transport, open beta-sheet;
Q6NZU0	hspa4b	Heat shock protein 4	_QIYIDK(cr)LAELK_	676	1	PKQIYIDKLAELKNL	......DK.......	cytosol	response to stress; response to stimulus;		ATP binding; nucleoside phosphate binding; nucleoside binding; ion binding; nucleotide binding; organic cyclic compound binding; heterocyclic compound binding; ribonucleoside binding; carbohydrate derivative binding; ribonucleotide binding; binding; purine ribonucleoside binding; anion binding; purine ribonucleotide binding; adenyl ribonucleotide binding; adenyl nucleotide binding; purine nucleoside binding; purine nucleotide binding; small molecule binding; purine ribonucleoside triphosphate binding;	K09489			IPR029048; IPR029047;	Heat shock protein 70kD, C-terminal domain; Heat shock protein 70kD, peptide-binding domain;
Q7ZV77	psma4	Proteasome subunit alpha type	_QK(cr)EVEELIK_	231	1	KIKVLKQKEVEELIK	.......K.V.....	mitochondria	modification-dependent macromolecule catabolic process; cellular macromolecule catabolic process; proteolysis involved in cellular protein catabolic process; cellular protein catabolic process; modification-dependent protein catabolic process; cellular protein metabolic process; ubiquitin-dependent protein catabolic process; primary metabolic process; cellular metabolic process; organic substance metabolic process; organic substance catabolic process; metabolic process; protein catabolic process; macromolecule catabolic process; cellular macromolecule metabolic process; cellular catabolic process; proteolysis; protein metabolic process; macromolecule metabolic process; cellular process; catabolic process;	intracellular organelle; proteasome core complex, alpha-subunit complex; proteasome complex; cytoplasm; macromolecular complex; intracellular part; cell; intracellular membrane-bounded organelle; organelle; nucleus; intracellular; cell part; proteasome core complex; membrane-bounded organelle; protein complex;	endopeptidase activity; threonine-type endopeptidase activity; peptidase activity; catalytic activity; threonine-type peptidase activity; peptidase activity, acting on L-amino acid peptides; hydrolase activity;	K02728	PSMA4; 20S proteasome subunit alpha 3 [EC:3.4.25.1]	dre03050 Proteasome - Danio rerio (zebrafish);	IPR029055; IPR000426;	Nucleophile aminohydrolases, N-terminal; Proteasome alpha-subunit, N-terminal domain;
F1QK60	krt4	Uncharacterized protein	_QK(cr)FEEMQSTAGQYGDDLR_	308	1	AESWYKQKFEEMQST	.......K.E.....	nuclear		intracellular organelle; intracellular non-membrane-bounded organelle; cytoskeletal part; intracellular organelle part; macromolecular complex; intracellular part; cell; keratin filament; organelle part; non-membrane-bounded organelle; organelle; cytoskeleton; intermediate filament cytoskeleton; cell part; intracellular; intermediate filament; protein complex;	structural molecule activity;	K07605				

Q6DRD1	atp5o	ATP synthase oligomycin sensitivity conferral protein	_QK(cr)TFVDALTK_	87	1	VKRSVKQKTFVDALT		cytosol	nucleoside metabolic process; purine nucleoside metabolic process; purine ribonucleoside monophosphate metabolic process; nucleotide metabolic process; single-organism process; purine ribonucleoside biosynthetic process; nucleoside biosynthetic process; biosynthetic process; ribonucleoside monophosphate biosynthetic process; cellular metabolic process; phosphate-containing compound metabolic process; establishment of localization; ribose phosphate biosynthetic process; metabolic process; carbohydrate derivative biosynthetic process; nucleoside phosphate metabolic process; ribose phosphate metabolic process; purine nucleoside triphosphate biosynthetic process; single-organism transport; heterocycle biosynthetic process; cation transmembrane transport; purine ribonucleoside triphosphate biosynthetic process; small molecule metabolic process; ATP biosynthetic process; single-organism cellular process; purine ribonucleoside triphosphate metabolic process; organic cyclic compound metabolic process; purine-containing compound metabolic process; energy coupled proton transport, down electrochemical gradient; ribonucleoside triphosphate metabolic process; purine		active transmembrane transporter activity; ATPase activity, coupled to transmembrane movement of ions; ion transmembrane transporter activity; primary active transmembrane transporter activity; ATPase activity, coupled to transmembrane movement of ions, rotational mechanism; hydrolase activity, acting on acid anhydrides; substrate-specific transporter activity; monovalent inorganic cation transmembrane transporter activity; hydrolase activity; nucleoside-triphosphatase activity; hydrogen ion transmembrane transporter activity; substrate-specific transmembrane transporter activity; proton-transporting ATP synthase activity, rotational mechanism; ATPase activity; P-P-bond-hydrolysis-driven transmembrane transporter activity; pyrophosphatase activity; cation-transporting ATPase activity; ATPase activity, coupled to transmembrane movement of substances; catalytic activity; ATPase activity, coupled to movement of substances; ATPase activity, coupled; hydrolase activity, acting on acid anhydrides, catalyzing transmembrane movement of substances; inorganic cation transmembrane transporter activity; hydrolase activity, acting on acid anhydrides, in phosphorus-containing anhydrides; cation transmembrane transporter activity; transporter activity; transmembrane transporter activity;	K02137	ATPeF0O; F-type H+-transporting ATPase subunit O	dre01100 Metabolic pathways - Danio rerio (zebrafish); dre00190 Oxidative phosphorylation - Danio rerio (zebrafish);	IPR026015;	F1F0 ATP synthase OSCP/delta subunit, N-terminal domain;
B8A568	myhz1.1	Uncharacterized protein	_QK(cr)YEEGQAELEGAQK_	1461	1	VLAEWKQKYEEGQAE	.......K.E.....	nuclear		intracellular organelle; intracellular non-membrane-bounded organelle; cytoskeletal part; intracellular organelle part; macromolecular complex; intracellular part; cell; actin cytoskeleton; organelle part; non-membrane-bounded organelle; organelle; cytoskeleton; cell part; intracellular; myosin complex; protein complex;	ATP binding; motor activity; nucleoside phosphate binding; nucleoside binding; ion binding; hydrolase activity, acting on acid anhydrides; hydrolase activity; nucleotide binding; nucleoside-triphosphatase activity; organic cyclic compound binding; heterocyclic compound binding; ribonucleoside binding; carbohydrate derivative binding; pyrophosphatase activity; catalytic activity; ribonucleotide binding; binding; purine ribonucleoside binding; anion binding; purine ribonucleotide binding; adenyl ribonucleotide binding; adenyl nucleotide binding; purine nucleoside binding; hydrolase activity, acting on acid anhydrides, in phosphorus-containing anhydrides; purine nucleotide binding; small molecule binding; purine ribonucleoside triphosphate binding;	K10352	MYH; myosin heavy chain	dre04530 Tight junction - Danio rerio (zebrafish);	IPR002928; IPR004009; IPR001609; IPR027417; IPR027401;	Myosin tail; Myosin, N-terminal, SH3-like; Myosin head, motor domain; P-loop containing nucleoside triphosphate hydrolase; Myosin-like IQ motif-containing domain;
B8A561	myhz1.2	Uncharacterized protein	_QK(cr)YEEGQAELEGAQK_	1461	1	VLAEWKQKYEEGQAE	.......K.E.....	nuclear		intracellular organelle; intracellular non-membrane-bounded organelle; cytoskeletal part; intracellular organelle part; macromolecular complex; intracellular part; cell; actin cytoskeleton; organelle part; non-membrane-bounded organelle; organelle; cytoskeleton; cell part; intracellular; myosin complex; protein complex;	ATP binding; motor activity; nucleoside phosphate binding; nucleoside binding; ion binding; hydrolase activity, acting on acid anhydrides; hydrolase activity; nucleotide binding; nucleoside-triphosphatase activity; organic cyclic compound binding; heterocyclic compound binding; ribonucleoside binding; carbohydrate derivative binding; pyrophosphatase activity; catalytic activity; ribonucleotide binding; binding; purine ribonucleoside binding; anion binding; purine ribonucleotide binding; adenyl ribonucleotide binding; adenyl nucleotide binding; purine nucleoside binding; hydrolase activity, acting on acid anhydrides, in phosphorus-containing anhydrides; purine nucleotide binding; small molecule binding; purine ribonucleoside triphosphate binding;	K10352	MYH; myosin heavy chain	dre04530 Tight junction - Danio rerio (zebrafish);	IPR002928; IPR004009; IPR001609; IPR027417; IPR027401;	Myosin tail; Myosin, N-terminal, SH3-like; Myosin head, motor domain; P-loop containing nucleoside triphosphate hydrolase; Myosin-like IQ motif-containing domain;
F1QK60	krt4	Uncharacterized protein	_QLDGLGNEK(cr)MK_	181	1	LDGLGNEKMKLEGEL		nuclear		intracellular organelle; intracellular non-membrane-bounded organelle; cytoskeletal part; intracellular organelle part; macromolecular complex; intracellular part; cell; keratin filament; organelle part; non-membrane-bounded organelle; organelle; cytoskeleton; intermediate filament cytoskeleton; cell part; intracellular; intermediate filament; protein complex;	structural molecule activity;	K07605				
P13104	tpma	Tropomyosin alpha-1 chain	_QLEDDLVALQK(cr)K_	48	1	DDLVALQKKLKATED	.......K.L.....	cytosol		cytoskeleton; organelle; intracellular organelle; intracellular non-membrane-bounded organelle; cell part; intracellular; cytoplasm; intracellular part; cell; non-membrane-bounded organelle;		K10373	TPM1; tropomyosin 1	dre04260 Cardiac muscle contraction - Danio rerio (zebrafish); dre04261 Adrenergic signaling in cardiomyocytes - Danio rerio [illegible]		
B8A561	myhz1.2	Uncharacterized protein	_QLEEK(cr)EALVSQLTR_	1296	1	FGRQLEEKEALVSQL		nuclear		intracellular organelle; intracellular non-membrane-bounded organelle; cytoskeletal part; intracellular organelle part; macromolecular complex; intracellular part; cell; actin cytoskeleton; organelle part; non-membrane-bounded organelle; organelle; cytoskeleton; cell part; intracellular; myosin complex; protein complex;	ATP binding; motor activity; nucleoside phosphate binding; nucleoside binding; ion binding; hydrolase activity, acting on acid anhydrides; hydrolase activity; nucleotide binding; nucleoside-triphosphatase activity; organic cyclic compound binding; heterocyclic compound binding; ribonucleoside binding; carbohydrate derivative binding; pyrophosphatase activity; catalytic activity; ribonucleotide binding; binding; purine ribonucleoside binding; anion binding; purine ribonucleotide binding; adenyl ribonucleotide binding; adenyl nucleotide binding; purine nucleoside binding; hydrolase activity, acting on acid anhydrides, in phosphorus-containing anhydrides; purine nucleotide binding; small molecule binding; purine ribonucleoside triphosphate binding;	K10352	MYH; myosin heavy chain	dre04530 Tight junction - Danio rerio (zebrafish);	IPR002928; IPR004009; IPR001609; IPR027417; IPR027401;	Myosin tail; Myosin, N-terminal, SH3-like; Myosin head, motor domain; P-loop containing nucleoside triphosphate hydrolase; Myosin-like IQ motif-containing domain;
Q9I8U9	tnnt3a	Fast skeletal muscle troponin T	_QLNIDHLNEDK(cr)LR	173	1	IDHLNEDKLRDKAQE	......DK.......	nuclear								
B8A568	myhz1.1	Uncharacterized protein	_QSEEAEEQANSHLSK(cr)LR_	1897	1	QANSHLSKLRKVQHE		nuclear		intracellular organelle; intracellular non-membrane-bounded organelle; cytoskeletal part; intracellular organelle part; macromolecular complex; intracellular part; cell; actin cytoskeleton; organelle part; non-membrane-bounded organelle; organelle; cytoskeleton; cell part; intracellular; myosin complex; protein complex;	ATP binding; motor activity; nucleoside phosphate binding; nucleoside binding; ion binding; hydrolase activity, acting on acid anhydrides; hydrolase activity; nucleotide binding; nucleoside-triphosphatase activity; organic cyclic compound binding; heterocyclic compound binding; ribonucleoside binding; carbohydrate derivative binding; pyrophosphatase activity; catalytic activity; ribonucleotide binding; binding; purine ribonucleoside binding; anion binding; purine ribonucleotide binding; adenyl ribonucleotide binding; adenyl nucleotide binding; purine nucleoside binding; hydrolase activity, acting on acid anhydrides, in phosphorus-containing anhydrides; purine nucleotide binding; small molecule binding; purine ribonucleoside triphosphate binding;	K10352	MYH; myosin heavy chain	dre04530 Tight junction - Danio rerio (zebrafish);	IPR002928; IPR004009; IPR001609; IPR027417; IPR027401;	Myosin tail; Myosin, N-terminal, SH3-like; Myosin head, motor domain; P-loop containing nucleoside triphosphate hydrolase; Myosin-like IQ motif-containing domain;

B8A561	myhz1.2	Uncharacterized protein	_QSEEAEEQANSHLSK(cr)LR_	1897	1	QANSHLSKLRKVQHE		nuclear		intracellular organelle; intracellular non-membrane-bounded organelle; cytoskeletal part; intracellular organelle part; macromolecular complex; intracellular part; cell; actin cytoskeleton; organelle part; non-membrane-bounded organelle; organelle; cytoskeleton; cell part; intracellular; myosin complex; protein complex;	ATP binding; motor activity; nucleoside phosphate binding; nucleoside binding; ion binding; hydrolase activity, acting on acid anhydrides; hydrolase activity; nucleotide binding; nucleoside-triphosphatase activity; organic cyclic compound binding; heterocyclic compound binding; ribonucleoside binding; carbohydrate derivative binding; pyrophosphatase activity; catalytic activity; ribonucleotide binding; binding; purine ribonucleoside binding; anion binding; purine ribonucleotide binding; adenyl ribonucleotide binding; adenyl nucleotide binding; purine nucleoside binding; hydrolase activity, acting on acid anhydrides, in phosphorus-containing anhydrides; purine nucleotide binding; small molecule binding; purine ribonucleoside triphosphate binding;	K10352	MYH; myosin heavy chain	dre04530 Tight junction - Danio rerio (zebrafish);	IPR002928; IPR004009; IPR001609; IPR027417; IPR027401;	Myosin tail; Myosin, N-terminal, SH3-like; Myosin head, motor domain; P-loop containing nucleoside triphosphate hydrolase; Myosin-like IQ motif-containing domain;
Q6DGL9	rpl38	Ribosomal protein L38	_QSLPPGLAVK(cr)ELK_	67	1	LPPGLAVKELK____		nuclear	developmental process; single-multicellular organism process; cellular protein metabolic process; single-organism process; biosynthetic process; primary metabolic process; organic substance biosynthetic process; cellular metabolic process; multicellular organismal process; cellular biosynthetic process; organic substance metabolic process; metabolic process; cellular macromolecule biosynthetic process; single-organism developmental process; cellular macromolecule metabolic process; gene expression; protein metabolic process; macromolecule metabolic process; multicellular organismal development; translation; macromolecule biosynthetic process; embryo development; cellular process; anatomical structure development;	ribosome; organelle; intracellular organelle; intracellular non-membrane-bounded organelle; cell part; intracellular; cytoplasm; macromolecular complex; intracellular part; cell; ribonucleoprotein complex; cytoplasmic part; non-membrane-bounded organelle;	structural constituent of ribosome; structural molecule activity;	K02923	RP-L38e; large subunit ribosomal protein L38e	dre03010 Ribosome - Danio rerio (zebrafish);		
Q08CK7	igf2bp1	Insulin-like growth factor 2 mRNA-binding protein 1	_QTQSK(cr)IDVHR_	231	1	ITKQTQSKIDVHRKE		cytosol,nuclear	regulation of cellular process; regulation of gene expression; regulation of cellular biosynthetic process; regulation of primary metabolic process; regulation of macromolecule metabolic process; posttranscriptional regulation of gene expression; regulation of macromolecule biosynthetic process; regulation of cellular metabolic process; regulation of biological process; regulation of cellular protein metabolic process; regulation of translation; regulation of protein metabolic process; regulation of biosynthetic process; regulation of metabolic process; biological regulation; regulation of cellular macromolecule biosynthetic process;	organelle; intracellular membrane-bounded organelle; intracellular organelle; nucleus; CRD-mediated mRNA stability complex; cell part; intracellular; cytoplasm; macromolecular complex; intracellular part; membrane-bounded organelle; protein complex; cell; cytoplasmic part;	organic cyclic compound binding; heterocyclic compound binding; RNA binding; mRNA binding; poly(A) RNA binding; nucleoside phosphate binding; mRNA 3'-UTR binding; binding; nucleic acid binding; nucleotide binding; small molecule binding;	K17391			IPR004087; IPR004088; IPR000504; IPR012677;	K Homology domain; K Homology domain, type 1; RNA recognition motif domain; Nucleotide-binding, alpha-beta plait;
Q92005	eef1a	Elongation factor 1-alpha	_QTVAVGVIK(cr)SVEK_	439	1	TVAVGVIKSVEKKIG	.......K.V.....	cytosol		intracellular part; cell; cell part; intracellular; cytoplasm;	nucleoside phosphate binding; translation elongation factor activity; hydrolase activity, acting on acid anhydrides; hydrolase activity; organic cyclic compound binding; heterocyclic compound binding; pyrophosphatase activity; catalytic activity; ribonucleotide binding; binding; GTPase activity; purine ribonucleoside binding; anion binding; hydrolase activity, acting on acid anhydrides, in phosphorus-containing anhydrides; guanyl nucleotide binding; GTP binding; purine nucleotide binding; purine ribonucleoside triphosphate binding; RNA binding; ion binding; nucleoside binding; nucleotide binding; nucleoside-triphosphatase activity; ribonucleoside binding; translation factor activity, nucleic acid binding; carbohydrate derivative binding; nucleic acid binding; purine ribonucleotide binding; guanyl ribonucleotide binding; purine nucleoside binding; small molecule binding;	K03231	EEF1A; elongation factor 1-alpha	dre03013 RNA transport - Danio rerio (zebrafish);	IPR004160; IPR004161; IPR009001; IPR027417; IPR000795; IPR009000;	Translation elongation factor EFTu/EF1A, C-terminal; Translation elongation factor EFTu/EF1A, domain 2; Translation elongation factor EF1A/initiation factor IF2gamma, C-terminal; P-loop containing nucleoside triphosphate hydrolase; Elongation factor, GTP-binding domain; Translation protein, beta-barrel domain;
F1QFC0	hspa9	Uncharacterized protein	_RFDDAEVQK(cr)DLK_	138	1	FDDAEVQKDLKNVPY	.......K.L.....	mitochondria	protein folding; metabolic process; cellular protein metabolic process; cellular macromolecule metabolic process; macromolecule metabolic process; protein metabolic process; primary metabolic process; cellular process; cellular metabolic process; organic substance metabolic process;		ATP binding; nucleoside phosphate binding; nucleoside binding; ion binding; nucleotide binding; organic cyclic compound binding; heterocyclic compound binding; ribonucleoside binding; carbohydrate derivative binding; ribonucleotide binding; binding; purine ribonucleoside binding; anion binding; purine ribonucleotide binding; adenyl ribonucleotide binding; adenyl nucleotide binding; purine nucleoside binding; purine nucleotide binding; small molecule binding; purine ribonucleoside triphosphate binding;	K04043	dnaK; molecular chaperone DnaK	dre03018 RNA degradation - Danio rerio (zebrafish);	IPR029048; IPR029047;	Heat shock protein 70kD, C-terminal domain; Heat shock protein 70kD, peptide-binding domain;
Q9I8U7	mylz3	Fast skeletal muscle myosin light polypeptide 3	_RIDFEAFLPMLK(cr)TVDANQK_	76	1	EAFLPMLKTVDANQK	.......K.V.....	cytosol			cation binding; metal ion binding; calcium ion binding; binding; ion binding;				IPR011992; IPR002048;	EF-hand domain pair; EF-hand domain;
Q804W0	pvalb1	Parvalbumin 4	_SADEVK(cr)K_	45	1	AKSADEVKKAFAIID		cytosol			cation binding; metal ion binding; calcium ion binding; binding; ion binding;				IPR011992; IPR002048;	EF-hand domain pair; EF-hand domain;
B8A561	myhz1.2	Uncharacterized protein	_SAETEK(cr)ELATM(ox)K_	849	1	LKSAETEKELATMKE	.......K.L.....	nuclear		intracellular organelle; intracellular non-membrane-bounded organelle; cytoskeletal part; intracellular organelle part; macromolecular complex; intracellular part; cell; actin cytoskeleton; organelle part; non-membrane-bounded organelle; organelle; cytoskeleton; cell part; intracellular; myosin complex; protein complex;	ATP binding; motor activity; nucleoside phosphate binding; nucleoside binding; ion binding; hydrolase activity, acting on acid anhydrides; hydrolase activity; nucleotide binding; nucleoside-triphosphatase activity; organic cyclic compound binding; heterocyclic compound binding; ribonucleoside binding; carbohydrate derivative binding; pyrophosphatase activity; catalytic activity; ribonucleotide binding; binding; purine ribonucleoside binding; anion binding; purine ribonucleotide binding; adenyl ribonucleotide binding; adenyl nucleotide binding; purine nucleoside binding; hydrolase activity, acting on acid anhydrides, in phosphorus-containing anhydrides; purine nucleotide binding; small molecule binding; purine ribonucleoside triphosphate binding;	K10352	MYH; myosin heavy chain	dre04530 Tight junction - Danio rerio (zebrafish);	IPR002928; IPR004009; IPR001609; IPR027417; IPR027401;	Myosin tail; Myosin, N-terminal, SH3-like; Myosin head, motor domain; P-loop containing nucleoside triphosphate hydrolase; Myosin-like IQ motif-containing domain;

B8A568	myhz1.1	Uncharacterized protein	_SAETEK(cr)ELATMK_	849	1	LKSAETEKELATMKE	.......K.L.....	nuclear		intracellular organelle; intracellular non-membrane-bounded organelle; cytoskeletal part; intracellular organelle part; macromolecular complex; intracellular part; cell; actin cytoskeleton; organelle part; non-membrane-bounded organelle; organelle; cytoskeleton; cell part; intracellular; myosin complex; protein complex;	ATP binding; motor activity; nucleoside phosphate binding; nucleoside binding; ion binding; hydrolase activity, acting on acid anhydrides; hydrolase activity; nucleotide binding; nucleoside-triphosphatase activity; organic cyclic compound binding; heterocyclic compound binding; ribonucleoside binding; carbohydrate derivative binding; pyrophosphatase activity; catalytic activity; ribonucleotide binding; binding; purine ribonucleoside binding; anion binding; purine ribonucleotide binding; adenyl ribonucleotide binding; adenyl nucleotide binding; purine nucleoside binding; hydrolase activity, acting on acid anhydrides, in phosphorus-containing anhydrides; purine nucleotide binding; small molecule binding; purine ribonucleoside triphosphate binding;	K10352	MYH; myosin heavy chain	dre04530 Tight junction - Danio rerio (zebrafish);	IPR002928; IPR004009; IPR001609; IPR027417; IPR027401;	Myosin tail; Myosin, N-terminal, SH3-like; Myosin head, motor domain; P-loop containing nucleoside triphosphate hydrolase; Myosin-like IQ motif-containing domain;
B8A561	myhz1.2	Uncharacterized protein	_SAETEK(cr)ELATMK_	849	1	LKSAETEKELATMKE	.......K.L.....	nuclear		intracellular organelle; intracellular non-membrane-bounded organelle; cytoskeletal part; intracellular organelle part; macromolecular complex; intracellular part; cell; actin cytoskeleton; organelle part; non-membrane-bounded organelle; organelle; cytoskeleton; cell part; intracellular; myosin complex; protein complex;	ATP binding; motor activity; nucleoside phosphate binding; nucleoside binding; ion binding; hydrolase activity, acting on acid anhydrides; hydrolase activity; nucleotide binding; nucleoside-triphosphatase activity; organic cyclic compound binding; heterocyclic compound binding; ribonucleoside binding; carbohydrate derivative binding; pyrophosphatase activity; catalytic activity; ribonucleotide binding; binding; purine ribonucleoside binding; anion binding; purine ribonucleotide binding; adenyl ribonucleotide binding; adenyl nucleotide binding; purine nucleoside binding; hydrolase activity, acting on acid anhydrides, in phosphorus-containing anhydrides; purine nucleotide binding; small molecule binding; purine ribonucleoside triphosphate binding;	K10352	MYH; myosin heavy chain	dre04530 Tight junction - Danio rerio (zebrafish);	IPR002928; IPR004009; IPR001609; IPR027417; IPR027401;	Myosin tail; Myosin, N-terminal, SH3-like; Myosin head, motor domain; P-loop containing nucleoside triphosphate hydrolase; Myosin-like IQ motif-containing domain;
Q8AWD0	vdac2	Uncharacterized protein	_SAK(cr)DIFNK_	15	1	ADLGKSAKDIFNKGY	.......K.I.....	cytosol	developmental process; response to wounding; tissue development; response to stress; wound healing; tissue regeneration; single-organism developmental process; fin regeneration; single-organism process; regeneration; developmental growth; response to stimulus; growth; anatomical structure development;	membrane; intracellular organelle; outer membrane; mitochondrial part; bounding membrane of organelle; organelle membrane; mitochondrial outer membrane; cytoplasm; intracellular organelle part; organelle envelope; intracellular part; cell; organelle part; mitochondrion; organelle; mitochondrial envelope; intracellular membrane-bounded organelle; intracellular; cell part; envelope; organelle outer membrane; membrane-bounded organelle; mitochondrial membrane; cytoplasmic part;	ion transmembrane transporter activity; passive transmembrane transporter activity; anion channel activity; substrate-specific transporter activity; channel activity; substrate-specific transmembrane transporter activity; voltage-gated ion channel activity; voltage-gated anion channel activity; substrate-specific channel activity; ion channel activity; gated channel activity; voltage-gated channel activity; transmembrane transporter activity; transporter activity; anion transmembrane transporter activity;	K15040	VDAC2; voltage-dependent anion channel protein 2	dre04020 Calcium signaling pathway - Danio rerio (zebrafish);	IPR023614;	Porin domain;
B0R198	vdac3	Uncharacterized protein	_SAK(cr)DIFSK_	40	1	ADLGKSAKDIFSKGY	.......K.I.....	cytosol		membrane; intracellular organelle; outer membrane; mitochondrial part; bounding membrane of organelle; organelle membrane; mitochondrial outer membrane; cytoplasm; intracellular organelle part; organelle envelope; intracellular part; cell; organelle part; mitochondrion; organelle; mitochondrial envelope; intracellular membrane-bounded organelle; intracellular; cell part; envelope; organelle outer membrane; membrane-bounded organelle; mitochondrial membrane; cytoplasmic part;	ion transmembrane transporter activity; passive transmembrane transporter activity; anion channel activity; substrate-specific transporter activity; channel activity; substrate-specific transmembrane transporter activity; voltage-gated ion channel activity; voltage-gated anion channel activity; substrate-specific channel activity; ion channel activity; gated channel activity; voltage-gated channel activity; transmembrane transporter activity; transporter activity; anion transmembrane transporter activity;				IPR023614;	Porin domain;
E9QF69	rpl18	60S ribosomal protein L18	_SDAPFNK(cr)VILR_	45	1	RSDAPFNKVILRRLF	.......K.I.....	mitochondria	cellular protein metabolic process; biosynthetic process; primary metabolic process; cellular metabolic process; organic substance biosynthetic process; cellular biosynthetic process; organic substance metabolic process; metabolic process; cellular macromolecule biosynthetic process; cellular macromolecule metabolic process; gene expression; macromolecule metabolic process; protein metabolic process; macromolecule biosynthetic process; translation; cellular process;	ribosome; organelle; intracellular organelle; intracellular non-membrane-bounded organelle; cell part; intracellular; cytoplasm; macromolecular complex; intracellular part; cell; ribonucleoprotein complex; cytoplasmic part; non-membrane-bounded organelle;	structural constituent of ribosome; structural molecule activity;				IPR021131;	Ribosomal protein L18e/L15P;
F1QCC0	tnni2b.1	Uncharacterized protein	_SDK(cr)EIEDLK_	87	1	TKVAKSDKEIEDLKI	......DK.......	mitochondria								
F1QCC0	tnni2b.1	Uncharacterized protein	_SDKEIEDLK(cr)IK_	93	1	DKEIEDLKIKVQDLK	..E....K.......	mitochondria								
Q7ZW95	rpl4	Ribosomal protein L4	_SEEVQK(cr)AIR_	300	1	IKSEEVQKAIRPANR	.......K.I.....	cytosol	cellular protein metabolic process; biosynthetic process; primary metabolic process; cellular metabolic process; organic substance biosynthetic process; cellular biosynthetic process; organic substance metabolic process; metabolic process; cellular macromolecule biosynthetic process; cellular macromolecule metabolic process; gene expression; macromolecule metabolic process; protein metabolic process; macromolecule biosynthetic process; translation; cellular process;	ribosome; organelle; intracellular organelle; intracellular non-membrane-bounded organelle; cell part; intracellular; cytoplasm; macromolecular complex; intracellular part; cell; ribonucleoprotein complex; cytoplasmic part; non-membrane-bounded organelle;	structural constituent of ribosome; structural molecule activity;	K02930	RP-L4e; large subunit ribosomal protein L4e	dre03010 Ribosome - Danio rerio (zebrafish);	IPR023574; IPR025755;	Ribosomal protein L4 domain; 60S ribosomal protein L4, C-terminal domain;
E9QDY3	cyt1	Uncharacterized protein	_SEITEVK(cr)R_	323	1	RSEITEVKRTLQSLE	..E....K.......	mitochondria		intracellular organelle; intracellular non-membrane-bounded organelle; cytoskeletal part; intracellular organelle part; macromolecular complex; intracellular part; cell; organelle part; non-membrane-bounded organelle; organelle; cytoskeleton; intermediate filament cytoskeleton; cell part; intracellular; intermediate filament; protein complex;	structural molecule activity;	K07604				
E9QDY3	cyt1	Uncharacterized protein	_SESLNK(cr)EVAVSTETLQTSR_	303	1	AKSESLNKEVAVSTE	.......K.V.....	mitochondria		intracellular organelle; intracellular non-membrane-bounded organelle; cytoskeletal part; intracellular organelle part; macromolecular complex; intracellular part; cell; organelle part; non-membrane-bounded organelle; organelle; cytoskeleton; intermediate filament cytoskeleton; cell part; intracellular; intermediate filament; protein complex;	structural molecule activity;	K07604				
F1QTL9	hnrnpa0b	Uncharacterized protein	_SEVITDK(cr)DTGK_	134	1	KSEVITDKDTGKKRG	......DK.......	nuclear			organic cyclic compound binding; heterocyclic compound binding; nucleoside phosphate binding; binding; nucleic acid binding; nucleotide binding; small molecule binding;	K12894			IPR000504; IPR012677;	RNA recognition motif domain; Nucleotide-binding, alpha-[illegible]

B8A568	myhz1.1	Uncharacterized protein	_SEYK(cr)MEIDDLSSNMEAVAK_	1227	1	EKEKSEYKMEIDDLS	..E....K.......	nuclear		intracellular organelle; intracellular non-membrane-bounded organelle; cytoskeletal part; intracellular organelle part; macromolecular complex; intracellular part; cell; actin cytoskeleton; organelle part; non-membrane-bounded organelle; organelle; cytoskeleton; cell part; intracellular; myosin complex; protein complex;	ATP binding; motor activity; nucleoside phosphate binding; nucleoside binding; ion binding; hydrolase activity, acting on acid anhydrides; hydrolase activity; nucleotide binding; nucleoside-triphosphatase activity; organic cyclic compound binding; heterocyclic compound binding; ribonucleoside binding; carbohydrate derivative binding; pyrophosphatase activity; catalytic activity; ribonucleotide binding; binding; purine ribonucleoside binding; anion binding; purine ribonucleotide binding; adenyl ribonucleotide binding; adenyl nucleotide binding; purine nucleoside binding; hydrolase activity, acting on acid anhydrides, in phosphorus-containing anhydrides; purine nucleotide binding; small molecule binding; purine ribonucleoside triphosphate binding;	K10352	MYH; myosin heavy chain	dre04530 Tight junction - Danio rerio (zebrafish);	IPR002928; IPR004009; IPR001609; IPR027417; IPR027401;	Myosin tail; Myosin, N-terminal, SH3-like; Myosin head, motor domain; P-loop containing nucleoside triphosphate hydrolase; Myosin-like IQ motif-containing domain;
B8A561	myhz1.2	Uncharacterized protein	_SEYK(cr)MEIDDLSSNMEAVAK_	1227	1	EKEKSEYKMEIDDLS	..E....K.......	nuclear		intracellular organelle; intracellular non-membrane-bounded organelle; cytoskeletal part; intracellular organelle part; macromolecular complex; intracellular part; cell; actin cytoskeleton; organelle part; non-membrane-bounded organelle; organelle; cytoskeleton; cell part; intracellular; myosin complex; protein complex;	ATP binding; motor activity; nucleoside phosphate binding; nucleoside binding; ion binding; hydrolase activity, acting on acid anhydrides; hydrolase activity; nucleotide binding; nucleoside-triphosphatase activity; organic cyclic compound binding; heterocyclic compound binding; ribonucleoside binding; carbohydrate derivative binding; pyrophosphatase activity; catalytic activity; ribonucleotide binding; binding; purine ribonucleoside binding; anion binding; purine ribonucleotide binding; adenyl ribonucleotide binding; adenyl nucleotide binding; purine nucleoside binding; hydrolase activity, acting on acid anhydrides, in phosphorus-containing anhydrides; purine nucleotide binding; small molecule binding; purine ribonucleoside triphosphate binding;	K10352	MYH; myosin heavy chain	dre04530 Tight junction - Danio rerio (zebrafish);	IPR002928; IPR004009; IPR001609; IPR027417; IPR027401;	Myosin tail; Myosin, N-terminal, SH3-like; Myosin head, motor domain; P-loop containing nucleoside triphosphate hydrolase; Myosin-like IQ motif-containing domain;
Q804W0	pvalb1	Parvalbumin 1	_SFFAK(cr)VGLSAK_	33	1	DYKSFFAKVGLSAKS		cytosol			cation binding; metal ion binding; calcium ion binding; binding; ion binding;				IPR011992; IPR002048;	EF-hand domain pair; EF-hand domain;
Q5RH26	atp5c1	ATP synthase subunit gamma	_SIK(cr)NIQK_	34	1	TIRLKSIKNIQKITK	.......K.I.....	mitochondria	nucleoside metabolic process; purine nucleoside metabolic process; purine ribonucleoside monophosphate metabolic process; nucleotide metabolic process; single-organism process; purine ribonucleoside biosynthetic process; nucleoside biosynthetic process; biosynthetic process; ribonucleoside monophosphate biosynthetic process; cellular metabolic process; phosphate-containing compound metabolic process; establishment of localization; ribose phosphate biosynthetic process; metabolic process; carbohydrate derivative biosynthetic process; nucleoside phosphate metabolic process; ribose phosphate metabolic process; purine nucleoside triphosphate biosynthetic process; single-organism transport; heterocycle biosynthetic process; cation transmembrane transport; purine ribonucleoside triphosphate biosynthetic process; small molecule metabolic process; ATP biosynthetic process; single-organism cellular process; purine ribonucleoside triphosphate metabolic process; organic cyclic compound metabolic process; purine-containing compound metabolic process; energy coupled proton transport, down electrochemical gradient; ribonucleoside triphosphate metabolic process; purine	proton-transporting ATP synthase complex, catalytic core F(1); membrane; membrane part; cell part; intracellular; proton-transporting two-sector ATPase complex; macromolecular complex; proton-transporting two-sector ATPase complex, catalytic domain; intracellular part; protein complex; cell; proton-transporting ATP synthase complex;	active transmembrane transporter activity; ATPase activity, coupled to transmembrane movement of ions; ion transmembrane transporter activity; primary active transmembrane transporter activity; ATPase activity, coupled to transmembrane movement of ions, rotational mechanism; hydrolase activity, acting on acid anhydrides; substrate-specific transporter activity; monovalent inorganic cation transmembrane transporter activity; hydrolase activity; nucleoside-triphosphatase activity; hydrogen ion transmembrane transporter activity; proton-transporting ATPase activity, rotational mechanism; substrate-specific transmembrane transporter activity; proton-transporting ATP synthase activity, rotational mechanism; ATPase activity; P-P-bond-hydrolysis-driven transmembrane transporter activity; pyrophosphatase activity; cation-transporting ATPase activity; ATPase activity, coupled to transmembrane movement of substances; catalytic activity; ATPase activity, coupled to movement of substances; ATPase activity, coupled; hydrogen-exporting ATPase activity; hydrolase activity, acting on acid anhydrides, catalyzing transmembrane movement of substances; inorganic cation transmembrane transporter activity; hydrolase activity, acting on acid anhydrides, in phosphorus-containing anhydrides; cation transmembrane transporter activity; transporter activity; transmembrane transporter activity;	K02136	ATPeF1G; F-type H+-transporting ATPase subunit gamma	dre01100 Metabolic pathways - Danio rerio (zebrafish); dre00190 Oxidative phosphorylation - Danio rerio (zebrafish);	IPR023633;	ATPase, F1 complex, gamma subunit domain;
Q58EE9	gfap	Glial fibrillary acidic protein	_SK(cr)FADLTDAANR_	272	1	TEEWYRSKFADLTDA	..E....K.......	mitochondria		intracellular organelle; intracellular non-membrane-bounded organelle; cytoskeletal part; intracellular organelle part; cytoplasm; type III intermediate filament; macromolecular complex; intracellular part; cell; organelle part; non-membrane-bounded organelle; organelle; cytoskeleton; intermediate filament cytoskeleton; intracellular; cell part; intermediate filament; protein complex;	structural molecule activity;	K05640			IPR006821;	Intermediate filament head, DNA-binding domain;
P13104	tpma	Tropomyosin alpha-1 chain	_SK(cr)QLEDDLVALQK_	37	1	KAAEERSKQLEDDLV	.......K.L.....	cytosol		cytoskeleton; organelle; intracellular organelle; intracellular non-membrane-bounded organelle; cell part; intracellular; cytoplasm; intracellular part; cell; non-membrane-bounded organelle;		K10373	TPM1; tropomyosin 1	dre04260 Cardiac muscle contraction - Danio rerio (zebrafish); dre04261 Adrenergic signaling in cardiomyocytes - Danio rerio (zebrafish);		
Q5TZ29	apobb.1	Uncharacterized protein	_SK(cr)VEEILSELK_	2104	1	EYFAIKSKVEEILSE	.......K.E.....	endoplasmic reticulum	response to chemical; response to stimulus;		lipid transporter activity; substrate-specific transporter activity; transporter activity;				IPR011030; IPR015817; IPR015818; IPR001747; IPR015819; IPR015816; IPR016024; IPR015255; IPR009454;	Vitellinogen, superhelical; Vitellinogen, open beta-sheet, subdomain 1; Vitellinogen, open beta-sheet, subdomain 2; Lipid transport protein, N-terminal; Lipid transport protein, beta-sheet shell; Vitellinogen, beta-sheet N-terminal; Armadillo-type fold; Vitellinogen, open beta-sheet; Lipid transport, open beta-sheet;

Q1ECX9	pdia4	Protein disulfide-isomerase A4	_SK(cr)VLEVAK_	434	1	ATQFWRSKVLEVAKD	.......K.L.....	extracellular	regulation of cellular process; regulation of biological quality; small molecule metabolic process; cell redox homeostasis; cellular protein metabolic process; single-organism cellular process; ether metabolic process; cellular homeostasis; single-organism process; primary metabolic process; cellular metabolic process; organic substance metabolic process; protein folding; homeostatic process; metabolic process; regulation of biological process; single-organism metabolic process; cellular macromolecule metabolic process; glycerol ether metabolic process; protein metabolic process; macromolecule metabolic process; cellular process; biological regulation;	endoplasmic reticulum lumen; intracellular organelle; endoplasmic reticulum; cytoplasm; intracellular organelle part; endomembrane system; intracellular part; cell; organelle part; organelle; intracellular membrane-bounded organelle; membrane-enclosed lumen; intracellular organelle lumen; endoplasmic reticulum part; cell part; intracellular; membrane-bounded organelle; cytoplasmic part; organelle lumen;	protein disulfide oxidoreductase activity; disulfide oxidoreductase activity; electron carrier activity; intramolecular oxidoreductase activity, transposing S-S bonds; isomerase activity; catalytic activity; oxidoreductase activity, acting on a sulfur group of donors; intramolecular oxidoreductase activity; protein disulfide isomerase activity; oxidoreductase activity;	K09582	PDIA4; protein disulfide-isomerase A4 [EC:5.3.4.1]	dre04141 Protein processing in endoplasmic reticulum - Danio rerio (zebrafish);	IPR005788; IPR013766; IPR012336;	Disulphide isomerase; Thioredoxin domain; Thioredoxin-like fold;
O42363	apoa1	Apolipoprotein A-I	_SK(cr)VVPMVEAVR_	182	1	NIEETKSKVVPMVEA	.......K.V.....	extracellular	cholesterol metabolic process; lipid transport; organic hydroxy compound metabolic process; alcohol metabolic process; small molecule metabolic process; steroid metabolic process; organic cyclic compound metabolic process; single-organism process; organic substance transport; lipid localization; primary metabolic process; sterol metabolic process; lipoprotein metabolic process; establishment of localization; organic substance metabolic process; transport; metabolic process; single-organism localization; single-organism metabolic process; macromolecule metabolic process; protein metabolic process; localization; macromolecule localization; single-organism transport; lipid metabolic process;	macromolecular complex; extracellular region part; extracellular space; high-density lipoprotein particle; protein-lipid complex; extracellular region; plasma lipoprotein particle;	lipid binding; binding;	K08757	APOA1; apolipoprotein A-I	dre03320 PPAR signaling pathway - Danio rerio (zebrafish);		
B8A568	myhz1.1	Uncharacterized protein	_SSVK(cr)LLATLYPPVVEETGGGK_	619	1	LYQKSSVKLLATLYP	.......K.L.....	nuclear		intracellular organelle; intracellular non-membrane-bounded organelle; cytoskeletal part; intracellular organelle part; macromolecular complex; intracellular part; cell; actin cytoskeleton; organelle part; non-membrane-bounded organelle; organelle; cytoskeleton; cell part; intracellular; myosin complex; protein complex;	ATP binding; motor activity; nucleoside phosphate binding; nucleoside binding; ion binding; hydrolase activity, acting on acid anhydrides; hydrolase activity; nucleotide binding; nucleoside-triphosphatase activity; organic cyclic compound binding; heterocyclic compound binding; ribonucleoside binding; carbohydrate derivative binding; pyrophosphatase activity; catalytic activity; ribonucleotide binding; binding; purine ribonucleoside binding; anion binding; purine ribonucleotide binding; adenyl ribonucleotide binding; adenyl nucleotide binding; purine nucleoside binding; hydrolase activity, acting on acid anhydrides, in phosphorus-containing anhydrides; purine nucleotide binding; small molecule binding; purine ribonucleoside triphosphate binding;	K10352	MYH; myosin heavy chain	dre04530 Tight junction - Danio rerio (zebrafish);	IPR002928; IPR004009; IPR001609; IPR027417; IPR027401;	Myosin tail; Myosin, N-terminal, SH3-like; Myosin head, motor domain; P-loop containing nucleoside triphosphate hydrolase; Myosin-like IQ motif-containing domain;
B8A561	myhz1.2	Uncharacterized protein	_SSVK(cr)LLATLYPPVVEETGGGK_	619	1	LYQKSSVKLLATLYP	.......K.L.....	nuclear		intracellular organelle; intracellular non-membrane-bounded organelle; cytoskeletal part; intracellular organelle part; macromolecular complex; intracellular part; cell; actin cytoskeleton; organelle part; non-membrane-bounded organelle; organelle; cytoskeleton; cell part; intracellular; myosin complex; protein complex;	ATP binding; motor activity; nucleoside phosphate binding; nucleoside binding; ion binding; hydrolase activity, acting on acid anhydrides; hydrolase activity; nucleotide binding; nucleoside-triphosphatase activity; organic cyclic compound binding; heterocyclic compound binding; ribonucleoside binding; carbohydrate derivative binding; pyrophosphatase activity; catalytic activity; ribonucleotide binding; binding; purine ribonucleoside binding; anion binding; purine ribonucleotide binding; adenyl ribonucleotide binding; adenyl nucleotide binding; purine nucleoside binding; hydrolase activity, acting on acid anhydrides, in phosphorus-containing anhydrides; purine nucleotide binding; small molecule binding; purine ribonucleoside triphosphate binding;	K10352	MYH; myosin heavy chain	dre04530 Tight junction - Danio rerio (zebrafish);	IPR002928; IPR004009; IPR001609; IPR027417; IPR027401;	Myosin tail; Myosin, N-terminal, SH3-like; Myosin head, motor domain; P-loop containing nucleoside triphosphate hydrolase; Myosin-like IQ motif-containing domain;
Q6P3J5	eef2b	Eukaryotic translation elongation factor 2	_STLTDSLVSK(cr)AGIIASAR_	42	1	LTDSLVSKAGIIASA		cytosol			nucleoside phosphate binding; translation elongation factor activity; hydrolase activity, acting on acid anhydrides; hydrolase activity; organic cyclic compound binding; heterocyclic compound binding; pyrophosphatase activity; catalytic activity; ribonucleotide binding; binding; GTPase activity; purine ribonucleoside binding; anion binding; hydrolase activity, acting on acid anhydrides, in phosphorus-containing anhydrides; guanyl nucleotide binding; GTP binding; purine nucleotide binding; purine ribonucleoside triphosphate binding; RNA binding; ion binding; nucleoside binding; nucleotide binding; nucleoside-triphosphatase activity; ribonucleoside binding; translation factor activity, nucleic acid binding; carbohydrate derivative binding; nucleic acid binding; purine ribonucleotide binding; guanyl ribonucleotide binding; purine nucleoside binding; small molecule binding;	K03234			IPR027417; IPR000795; IPR005517; IPR005225; IPR004161; IPR000640; IPR020568; IPR014721; IPR009022; IPR009000;	P-loop containing nucleoside triphosphate hydrolase; Elongation factor, GTP-binding domain; Translation elongation factor EFG/EF2, domain IV; Small GTP-binding protein domain; Translation elongation factor EFTu/EF1A, domain 2; Translation elongation factor EFG, V domain; Ribosomal protein S5 domain 2-type fold; Ribosomal protein S5 domain 2-type fold, subgroup; Elongation factor G, III-V domain; Translation protein, beta-barrel domain;

Q90473	hspa8	Heat shock cognate 71 kDa protein	_STVEDEK(cr)LK_	557	1	KSTVEDEKLKGKISD		cytosol	developmental process; response to wounding; tissue development; response to stress; wound healing; tissue regeneration; single-organism developmental process; fin regeneration; single-organism process; regeneration; developmental growth; response to stimulus; growth; anatomical structure development;		ATP binding; nucleoside phosphate binding; nucleoside binding; ion binding; nucleotide binding; organic cyclic compound binding; heterocyclic compound binding; ribonucleoside binding; carbohydrate derivative binding; ribonucleotide binding; binding; purine ribonucleoside binding; anion binding; purine ribonucleotide binding; adenyl ribonucleotide binding; adenyl nucleotide binding; purine nucleoside binding; purine nucleotide binding; small molecule binding; purine ribonucleoside triphosphate binding;	K03283	HSPA1_8; heat shock 70kDa protein 1/8	dre04010 MAPK signaling pathway - Danio rerio (zebrafish); dre04141 Protein processing in endoplasmic reticulum - Danio rerio (zebrafish); dre04144 Endocytosis - Danio rerio (zebrafish); dre03040 Spliceosome - Danio rerio (zebrafish);	IPR029048; IPR029047;	Heat shock protein 70kD, C-terminal domain; Heat shock protein 70kD, peptide-binding domain;
Q6GQM9	eno2	Eno2 protein	_TAIDK(cr)AGFTDK_	233	1	LIKTAIDKAGFTDKV	......DK.......	cytoskeleton	single-organism carbohydrate catabolic process; glycolytic process; single-organism catabolic process; small molecule metabolic process; single-organism cellular process; single-organism process; carbohydrate metabolic process; primary metabolic process; cellular metabolic process; organic substance metabolic process; carbohydrate catabolic process; single-organism carbohydrate metabolic process; organic substance catabolic process; metabolic process; pyruvate metabolic process; generation of precursor metabolites and energy; single-organism metabolic process; carboxylic acid metabolic process; monocarboxylic acid metabolic process; organic acid metabolic process; cellular process; catabolic process; oxoacid metabolic process;	catalytic complex; cell part; intracellular; cytoplasm; macromolecular complex; intracellular part; cytosolic part; cell; cytosol; protein complex; phosphopyruvate hydratase complex; cytoplasmic part;	magnesium ion binding; phosphopyruvate hydratase activity; catalytic activity; binding; ion binding; metal ion binding; cation binding; lyase activity; hydro-lyase activity; carbon-oxygen lyase activity;	K01689	ENO; enolase [EC:4.2.1.11]	dre00010 Glycolysis / Gluconeogenesis - Danio rerio (zebrafish); dre01230 Biosynthesis of amino acids - Danio rerio (zebrafish); dre01200 Carbon metabolism - Danio rerio (zebrafish); dre01100 Metabolic pathways - Danio rerio (zebrafish); dre03018 RNA degradation - Danio rerio (zebrafish);	IPR029065; IPR020810; IPR029017; IPR020811;	Enolase C-terminal domain-like; Enolase, C-terminal; Enolase N-terminal domain-like; Enolase, N-terminal;
E9QBF0	tpi1b	Triosephosphate isomerase	_TASPQQAQEVHDK(cr)LR_	194	1	QAQEVHDKLRQW___		cytosol	carbohydrate biosynthetic process; glycolytic process; hexose biosynthetic process; single-organism process; biosynthetic process; monosaccharide biosynthetic process; cellular metabolic process; organic substance metabolic process; glucose metabolic process; pyruvate metabolic process; metabolic process; gluconeogenesis; generation of precursor metabolites and energy; monosaccharide metabolic process; monocarboxylic acid metabolic process; cellular process; single-organism carbohydrate catabolic process; single-organism biosynthetic process; single-organism catabolic process; small molecule metabolic process; single-organism cellular process; carbohydrate metabolic process; primary metabolic process; organic substance biosynthetic process; carbohydrate catabolic process; single-organism carbohydrate metabolic process; organic substance catabolic process; single-organism metabolic process; carboxylic acid metabolic process; organic acid metabolic process; catabolic process; oxoacid metabolic process; hexose metabolic process;		isomerase activity; intramolecular oxidoreductase activity; triose-phosphate isomerase activity; catalytic activity; intramolecular oxidoreductase activity, interconverting aldoses and ketoses;				IPR013785;	Aldolase-type TIM barrel;
A2BHA3	ckma	Uncharacterized protein	_TDLNFENLK(cr)GGDDLDPNYVLSSR_	116	1	DLNFENLKGGDDLDP		cytosol			ATP binding; nucleoside phosphate binding; nucleoside binding; ion binding; nucleotide binding; organic cyclic compound binding; heterocyclic compound binding; ribonucleoside binding; carbohydrate derivative binding; kinase activity; catalytic activity; ribonucleotide binding; binding; purine ribonucleoside binding; anion binding; purine ribonucleotide binding; adenyl ribonucleotide binding; adenyl nucleotide binding; purine nucleoside binding; transferase activity; transferase activity, transferring phosphorus-containing groups; purine nucleotide binding; small molecule binding; purine ribonucleoside triphosphate binding;	K00933	E2.7.3.2; creatine kinase [EC:2.7.3.2]	dre00330 Arginine and proline metabolism - Danio rerio (zebrafish); dre01100 Metabolic pathways - Danio rerio (zebrafish);	IPR022413; IPR022414; IPR014746;	ATP:guanido phosphotransferase, N-terminal; ATP:guanido phosphotransferase, catalytic domain; Glutamine synthetase/guanido kinase, catalytic domain;
F1QIR4	-	Uncharacterized protein	_TEELEDAK(cr)K_	1393	1	TEELEDAKKKLAQRL	..E....K.......	cytosol	cellular component biogenesis; muscle cell development; single-multicellular organism process; cellular component organization; muscle system process; cellular developmental process; cellular component morphogenesis; single-organism process; actin filament organization; cell development; anatomical structure morphogenesis; cell differentiation; actin cytoskeleton organization; muscle contraction; cellular component assembly; organelle assembly; macromolecular complex assembly; myofibril assembly; musculoskeletal movement; slow-twitch skeletal muscle fiber contraction; muscle structure development; cytoskeleton organization; anatomical structure formation involved in morphogenesis; cellular process; striated muscle myosin thick filament assembly; protein complex assembly; cellular protein complex assembly; skeletal muscle thin filament assembly; developmental process; cellular macromolecular complex assembly; striated muscle cell differentiation; actomyosin structure organization; system process; protein complex subunit organization; multicellular organismal movement; single-organism cellular process; cellular component assembly involved in morphogenesis; striated muscle contraction; myosin filament	intracellular organelle; intracellular non-membrane-bounded organelle; cytoskeletal part; intracellular organelle part; macromolecular complex; intracellular part; cell; actin cytoskeleton; organelle part; non-membrane-bounded organelle; organelle; cytoskeleton; cell part; intracellular; myosin complex; protein complex;	ATP binding; motor activity; nucleoside phosphate binding; nucleoside binding; ion binding; hydrolase activity, acting on acid anhydrides; hydrolase activity; nucleotide binding; nucleoside-triphosphatase activity; organic cyclic compound binding; heterocyclic compound binding; ribonucleoside binding; carbohydrate derivative binding; pyrophosphatase activity; catalytic activity; ribonucleotide binding; binding; purine ribonucleoside binding; anion binding; purine ribonucleotide binding; adenyl ribonucleotide binding; adenyl nucleotide binding; purine nucleoside binding; hydrolase activity, acting on acid anhydrides, in phosphorus-containing anhydrides; purine nucleotide binding; small molecule binding; purine ribonucleoside triphosphate binding;	K10352	MYH; myosin heavy chain	dre04530 Tight junction - Danio rerio (zebrafish);	IPR002928; IPR004009; IPR027417; IPR001609; IPR027401;	Myosin tail; Myosin, N-terminal, SH3-like; P-loop containing nucleoside triphosphate hydrolase; Myosin head, motor domain; Myosin-like IQ motif-containing domain;

X1WF87	myhb	Uncharacterized protein	_TEELEEAK(cr)K_	1392	1	TEELEEAKKKLAQRL	..E....K.......	cytosol				K10352	MYH; myosin heavy chain	dre04530 Tight junction - Danio rerio (zebrafish);	IPR002928; IPR004009; IPR027417; IPR001609; IPR027401;	Myosin tail; Myosin, N-terminal, SH3-like; P-loop containing nucleoside triphosphate hydrolase; Myosin head, motor domain; Myosin-like IQ motif-containing domain;
B8A568	myhz1.1	Uncharacterized protein	_TEELEESK(cr)K_	1392	1	TEELEESKKKLAQRL	..E....K.......	nuclear		intracellular organelle; intracellular non-membrane-bounded organelle; cytoskeletal part; intracellular organelle part; macromolecular complex; intracellular part; cell; actin cytoskeleton; organelle part; non-membrane-bounded organelle; organelle; cytoskeleton; cell part; intracellular; myosin complex; protein complex;	ATP binding; motor activity; nucleoside phosphate binding; nucleoside binding; ion binding; hydrolase activity, acting on acid anhydrides; hydrolase activity; nucleotide binding; nucleoside-triphosphatase activity; organic cyclic compound binding; heterocyclic compound binding; ribonucleoside binding; carbohydrate derivative binding; pyrophosphatase activity; catalytic activity; ribonucleotide binding; binding; purine ribonucleoside binding; anion binding; purine ribonucleotide binding; adenyl ribonucleotide binding; adenyl nucleotide binding; purine nucleoside binding; hydrolase activity, acting on acid anhydrides, in phosphorus-containing anhydrides; purine nucleotide binding; small molecule binding; purine ribonucleoside triphosphate binding;	K10352	MYH; myosin heavy chain	dre04530 Tight junction - Danio rerio (zebrafish);	IPR002928; IPR004009; IPR001609; IPR027417; IPR027401;	Myosin tail; Myosin, N-terminal, SH3-like; Myosin head, motor domain; P-loop containing nucleoside triphosphate hydrolase; Myosin-like IQ motif-containing domain;
B8A561	myhz1.2	Uncharacterized protein	_TEELEESK(cr)K_	1392	1	TEELEESKKKLAQRL	..E....K.......	nuclear		intracellular organelle; intracellular non-membrane-bounded organelle; cytoskeletal part; intracellular organelle part; macromolecular complex; intracellular part; cell; actin cytoskeleton; organelle part; non-membrane-bounded organelle; organelle; cytoskeleton; cell part; intracellular; myosin complex; protein complex;	ATP binding; motor activity; nucleoside phosphate binding; nucleoside binding; ion binding; hydrolase activity, acting on acid anhydrides; hydrolase activity; nucleotide binding; nucleoside-triphosphatase activity; organic cyclic compound binding; heterocyclic compound binding; ribonucleoside binding; carbohydrate derivative binding; pyrophosphatase activity; catalytic activity; ribonucleotide binding; binding; purine ribonucleoside binding; anion binding; purine ribonucleotide binding; adenyl ribonucleotide binding; adenyl nucleotide binding; purine nucleoside binding; hydrolase activity, acting on acid anhydrides, in phosphorus-containing anhydrides; purine nucleotide binding; small molecule binding; purine ribonucleoside triphosphate binding;	K10352	MYH; myosin heavy chain	dre04530 Tight junction - Danio rerio (zebrafish);	IPR002928; IPR004009; IPR001609; IPR027417; IPR027401;	Myosin tail; Myosin, N-terminal, SH3-like; Myosin head, motor domain; P-loop containing nucleoside triphosphate hydrolase; Myosin-like IQ motif-containing domain;
E7EYE1	si:dkey-78p20.4	Uncharacterized protein	_TELNYEILEK(cr)GHVR_	826	1	LNYEILEKGHVRFWV		nuclear							IPR003598; IPR007110; IPR013783; IPR003599; IPR003961; IPR013098;	Immunoglobulin subtype 2; Immunoglobulin-like domain; Immunoglobulin-like fold; Immunoglobulin subtype; Fibronectin, type III; Immunoglobulin I-set;
Q6IMW7	pvalb4	Parvalbumin 4	_TFLK(cr)AGDSDGDGK_	88	1	GETKTFLKAGDSDGD		cytosol			cation binding; metal ion binding; calcium ion binding; binding; ion binding;				IPR011992; IPR002048;	EF-hand domain pair; EF-hand domain;
Q503V2	apoea	Uncharacterized protein	_THMEEAK(cr)DR_	116	1	RTHMEEAKDRVTEYT		extracellular	lipid transport; single-organism process; lipid localization; organic substance transport; primary metabolic process; lipoprotein metabolic process; organic substance metabolic process; establishment of localization; transport; metabolic process; single-organism localization; protein metabolic process; macromolecule metabolic process; localization; macromolecule localization; single-organism transport;	extracellular region;	lipid binding; binding;	K04524				
Q68EH0	gspt1	Uncharacterized protein	_TIAIGK(cr)VLK_	555	1	GKTIAIGKVLKLVPE	.......K.L......	cytosol			nucleoside phosphate binding; nucleoside binding; ion binding; hydrolase activity, acting on acid anhydrides; hydrolase activity; nucleotide binding; nucleoside-triphosphatase activity; organic cyclic compound binding; heterocyclic compound binding; ribonucleoside binding; carbohydrate derivative binding; pyrophosphatase activity; catalytic activity; ribonucleotide binding; binding; GTPase activity; purine ribonucleoside binding; anion binding; purine ribonucleotide binding; purine nucleoside binding; guanyl ribonucleotide binding; hydrolase activity, acting on acid anhydrides, in phosphorus-containing anhydrides; guanyl nucleotide binding; GTP binding; purine nucleotide binding; small molecule binding; purine ribonucleoside triphosphate binding;	K03267	ERF3; peptide chain release factor subunit 3	dre03015 mRNA surveillance pathway - Danio rerio (zebrafish);	IPR004160; IPR004161; IPR027417; IPR009001; IPR000795; IPR009818; IPR009000;	Translation elongation factor EFTu/EF1A, C-terminal; Translation elongation factor EFTu/EF1A, domain 2; P-loop containing nucleoside triphosphate hydrolase; Translation elongation factor EF1A/initiation factor IF2gamma, C-terminal; Elongation factor, GTP-binding domain; Ataxin-2, C-terminal; Translation protein, beta-barrel domain;
P13104	tpma	Tropomyosin alpha-1 chain	_TIDDLEDELYAQK(cr)LK_	264	1	EDELYAQKLKYKAIS	..E....K.......	cytosol		cytoskeleton; organelle; intracellular organelle; intracellular non-membrane-bounded organelle; cell part; intracellular; cytoplasm; intracellular part; cell; non-membrane-bounded organelle;		K10373	TPM1; tropomyosin 1	dre04260 Cardiac muscle contraction - Danio rerio (zebrafish); dre04261 Adrenergic signaling in cardiomyocytes - Danio rerio (zebrafish);		

Q92005	eef1a	Elongation factor 1-alpha	_TIEK(cr)FEK_	41	1	IDKRTIEKFEKEAAE	.......K.E.....	cytosol		intracellular part; cell; cell part; intracellular; cytoplasm;	nucleoside phosphate binding; translation elongation factor activity; hydrolase activity, acting on acid anhydrides; hydrolase activity; organic cyclic compound binding; heterocyclic compound binding; pyrophosphatase activity; catalytic activity; ribonucleotide binding; binding; GTPase activity; purine ribonucleoside binding; anion binding; hydrolase activity, acting on acid anhydrides, in phosphorus-containing anhydrides; guanyl nucleotide binding; GTP binding; purine nucleotide binding; purine ribonucleoside triphosphate binding; RNA binding; ion binding; nucleoside binding; nucleotide binding; nucleoside-triphosphatase activity; ribonucleoside binding; translation factor activity, nucleic acid binding; carbohydrate derivative binding; nucleic acid binding; purine ribonucleotide binding; guanyl ribonucleotide binding; purine nucleoside binding; small molecule binding;	K03231	EEF1A; elongation factor 1-alpha	dre03013 RNA transport - Danio rerio (zebrafish);	IPR004160; IPR004161; IPR009001; IPR027417; IPR000795; IPR009000;	Translation elongation factor EFTu/EF1A, C-terminal; Translation elongation factor EFTu/EF1A, domain 2; Translation elongation factor EF1A/initiation factor IF2gamma, C-terminal; P-loop containing nucleoside triphosphate hydrolase; Elongation factor, GTP-binding domain; Translation protein, beta-barrel domain;
F1QIR4	-	Uncharacterized protein	_TINDFTMQK(cr)AK_	1280	1	INDFTMQKAKLQTEN		cytosol	cellular component biogenesis; muscle cell development; single-multicellular organism process; cellular component organization; muscle system process; cellular developmental process; cellular component morphogenesis; single-organism process; actin filament organization; cell development; anatomical structure morphogenesis; cell differentiation; actin cytoskeleton organization; muscle contraction; cellular component assembly; organelle assembly; macromolecular complex assembly; myofibril assembly; musculoskeletal movement; slow-twitch skeletal muscle fiber contraction; muscle structure development; cytoskeleton organization; anatomical structure formation involved in morphogenesis; cellular process; striated muscle myosin thick filament assembly; protein complex assembly; cellular protein complex assembly; skeletal muscle thin filament assembly; developmental process; cellular macromolecular complex assembly; striated muscle cell differentiation; actomyosin structure organization; system process; protein complex subunit organization; multicellular organismal movement; single-organism cellular process; cellular component assembly involved in morphogenesis; striated muscle contraction; myosin filament [illegible]	intracellular organelle; intracellular non-membrane-bounded organelle; cytoskeletal part; intracellular organelle part; macromolecular complex; intracellular part; cell; actin cytoskeleton; organelle part; non-membrane-bounded organelle; organelle; cytoskeleton; cell part; intracellular; myosin complex; protein complex;	ATP binding; motor activity; nucleoside phosphate binding; nucleoside binding; ion binding; hydrolase activity, acting on acid anhydrides; hydrolase activity; nucleotide binding; nucleoside-triphosphatase activity; organic cyclic compound binding; heterocyclic compound binding; ribonucleoside binding; carbohydrate derivative binding; pyrophosphatase activity; catalytic activity; ribonucleotide binding; binding; purine ribonucleoside binding; anion binding; purine ribonucleotide binding; adenyl ribonucleotide binding; adenyl nucleotide binding; purine nucleoside binding; hydrolase activity, acting on acid anhydrides, in phosphorus-containing anhydrides; purine nucleotide binding; small molecule binding; purine ribonucleoside triphosphate binding;	K10352	MYH; myosin heavy chain	dre04530 Tight junction - Danio rerio (zebrafish);	IPR002928; IPR004009; IPR027417; IPR001609; IPR027401;	Myosin tail; Myosin, N-terminal, SH3-like; P-loop containing nucleoside triphosphate hydrolase; Myosin head, motor domain; Myosin-like IQ motif-containing domain;
B0R193	ubb	Uncharacterized protein	_TITLEVEPSDTIENVK(cr)AK_	27	1	SDTIENVKAKIQDKE		cytosol,nuclear							IPR029071; IPR000626;	Ubiquitin-related domain; Ubiquitin-like;
B8A4A7	sncga	Gamma-synuclein	TK(cr)AGVEEAAAK	23	1	VAAAEKTKAGVEEAA		cytosol								
F1R8P5	-	Uncharacterized protein	TK(cr)AGVEEAALK	23	1	VAAAEKTKAGVEEAA		cytosol								
E9QIC5	srsf1a	Serine/arginine-rich-splicing factor 1A	_TK(cr)DVEDVFYK_	29	1	LPPDIRTKDVEDVFY	.......K.V.....	extracellular			organic cyclic compound binding; heterocyclic compound binding; nucleoside phosphate binding; binding; nucleic acid binding; nucleotide binding; small molecule binding;				IPR000504; IPR012677;	RNA recognition motif domain; Nucleotide-binding, alpha- [illegible]
Q6ZM50	capza1b	Si:dkey-16k6.1	_TK(cr)IDWNK_	268	1	QLPVTRTKIDWNKIL		cytosol	cellular component organization; single-organism organelle organization; actin cytoskeleton organization; organelle organization; actin filament-based process; single-organism cellular process; single-organism process; cytoskeleton organization; cellular process; cellular component organization or biogenesis;	intracellular organelle; intracellular non-membrane-bounded organelle; cytoskeletal part; intracellular organelle part; F-actin capping protein complex; macromolecular complex; intracellular part; cell; actin cytoskeleton; organelle part; non-membrane-bounded organelle; organelle; cytoskeleton; cell part; intracellular; protein complex;		K10364				
Q90Z10	rpl13	60S ribosomal protein L13	_TK(cr)LIIFPR_	123	1	RLKEYRTKLIIFPRK	.......K.I.....	cytosol	regulation of cellular process; cellular protein metabolic process; biosynthetic process; primary metabolic process; organic substance biosynthetic process; cellular metabolic process; cellular biosynthetic process; organic substance metabolic process; metabolic process; regulation of biological process; cellular macromolecule biosynthetic process; cellular macromolecule metabolic process; gene expression; protein metabolic process; macromolecule metabolic process; translation; macromolecule biosynthetic process; cellular process; regulation of cell cycle; biological regulation;	ribosome; organelle; intracellular organelle; intracellular non-membrane-bounded organelle; cell part; intracellular; cytoplasm; macromolecular complex; intracellular part; cell; ribonucleoprotein complex; cytoplasmic part; non-membrane-bounded organelle;	structural constituent of ribosome; structural molecule activity;	K02873	RP-L13e; large subunit ribosomal protein L13e	dre03010 Ribosome - Danio rerio (zebrafish);		
B0S564	-	Protein disulfide-isomerase	_TLDGFTK(cr)FLESGGK_	466	1	RTLDGFTKFLESGGK	.......K.L.....	extracellular	regulation of cellular process; regulation of biological quality; small molecule metabolic process; cell redox homeostasis; single-organism cellular process; ether metabolic process; cellular homeostasis; single-organism process; organic substance metabolic process; homeostatic process; metabolic process; regulation of biological process; single-organism metabolic process; glycerol ether metabolic process; cellular process; biological regulation;	organelle; intracellular membrane-bounded organelle; intracellular organelle; endoplasmic reticulum; cell part; intracellular; cytoplasm; endomembrane system; macromolecular complex; intracellular part; membrane-bounded organelle; cell; protein complex; collagen trimer; cytoplasmic part;	protein disulfide oxidoreductase activity; disulfide oxidoreductase activity; oxidoreductase activity, acting on single donors with incorporation of molecular oxygen, incorporation of two atoms of oxygen; electron carrier activity; oxidoreductase activity, acting on single donors with incorporation of molecular oxygen; isomerase activity; catalytic activity; dioxygenase activity; oxidoreductase activity, acting on a sulfur group of donors; oxidoreductase activity;	K09580	PDIA1; protein disulfide-isomerase A1 [EC:5.3.4.1]	dre04141 Protein processing in endoplasmic reticulum - Danio rerio (zebrafish);	IPR005788; IPR013766; IPR012336;	Disulphide isomerase; Thioredoxin domain; Thioredoxin-like fold;

B8A568	myhz1.1	Uncharacterized protein	_TLEDQLSEIK(cr)SK_	1262	1	EDQLSEIKSKNDENL		nuclear		intracellular organelle; intracellular non-membrane-bounded organelle; cytoskeletal part; intracellular organelle part; macromolecular complex; intracellular part; cell; actin cytoskeleton; organelle part; non-membrane-bounded organelle; organelle; cytoskeleton; cell part; intracellular; myosin complex; protein complex;	ATP binding; motor activity; nucleoside phosphate binding; nucleoside binding; ion binding; hydrolase activity, acting on acid anhydrides; hydrolase activity; nucleotide binding; nucleoside-triphosphatase activity; organic cyclic compound binding; heterocyclic compound binding; ribonucleoside binding; carbohydrate derivative binding; pyrophosphatase activity; catalytic activity; ribonucleotide binding; binding; purine ribonucleoside binding; anion binding; purine ribonucleotide binding; adenyl ribonucleotide binding; adenyl nucleotide binding; purine nucleoside binding; hydrolase activity, acting on acid anhydrides, in phosphorus-containing anhydrides; purine nucleotide binding; small molecule binding; purine ribonucleoside triphosphate binding;	K10352	MYH; myosin heavy chain	dre04530 Tight junction - Danio rerio (zebrafish);	IPR002928; IPR004009; IPR001609; IPR027417; IPR027401;	Myosin tail; Myosin, N-terminal, SH3-like; Myosin head, motor domain; P-loop containing nucleoside triphosphate hydrolase; Myosin-like IQ motif-containing domain;
B8A561	myhz1.2	Uncharacterized protein	_TLEDQLSEIK(cr)SK_	1262	1	EDQLSEIKSKNDENL		nuclear		intracellular organelle; intracellular non-membrane-bounded organelle; cytoskeletal part; intracellular organelle part; macromolecular complex; intracellular part; cell; actin cytoskeleton; organelle part; non-membrane-bounded organelle; organelle; cytoskeleton; cell part; intracellular; myosin complex; protein complex;	ATP binding; motor activity; nucleoside phosphate binding; nucleoside binding; ion binding; hydrolase activity, acting on acid anhydrides; hydrolase activity; nucleotide binding; nucleoside-triphosphatase activity; organic cyclic compound binding; heterocyclic compound binding; ribonucleoside binding; carbohydrate derivative binding; pyrophosphatase activity; catalytic activity; ribonucleotide binding; binding; purine ribonucleoside binding; anion binding; purine ribonucleotide binding; adenyl ribonucleotide binding; adenyl nucleotide binding; purine nucleoside binding; hydrolase activity, acting on acid anhydrides, in phosphorus-containing anhydrides; purine nucleotide binding; small molecule binding; purine ribonucleoside triphosphate binding;	K10352	MYH; myosin heavy chain	dre04530 Tight junction - Danio rerio (zebrafish);	IPR002928; IPR004009; IPR001609; IPR027417; IPR027401;	Myosin tail; Myosin, N-terminal, SH3-like; Myosin head, motor domain; P-loop containing nucleoside triphosphate hydrolase; Myosin-like IQ motif-containing domain;
Q9I8U9	tnnt3a	Fast skeletal muscle troponin T	_TLESEK(cr)FEHMER_	192	1	IKTLESEKFEHMERL	.......K.E.....	nuclear								
Q6P5L3	rpl19	60S ribosomal protein L19	_TLSK(cr)EDETK_	190	1	EIIKTLSKEDETKK_		cytosol	cellular protein metabolic process; biosynthetic process; primary metabolic process; cellular metabolic process; organic substance biosynthetic process; cellular biosynthetic process; organic substance metabolic process; metabolic process; cellular macromolecule biosynthetic process; cellular macromolecule metabolic process; gene expression; macromolecule metabolic process; protein metabolic process; macromolecule biosynthetic process; translation; cellular process;	ribosome; organelle; intracellular organelle; intracellular non-membrane-bounded organelle; cell part; intracellular; cytoplasm; macromolecular complex; intracellular part; cell; ribonucleoprotein complex; cytoplasmic part; non-membrane-bounded organelle;	structural constituent of ribosome; structural molecule activity;	K02885	RP-L19e; large subunit ribosomal protein L19e	dre03010 Ribosome - Danio rerio (zebrafish);	IPR000196; IPR015974; IPR015972;	Ribosomal protein L19/L19e domain; Ribosomal protein L19/L19e, domain 3; Ribosomal protein L19/L19e, domain 1;
E7FF12	-	Uncharacterized protein	_TMATEK(cr)LLK_	163	1	MRTMATEKLLKAMPA	.......K.L.....	cytosol,nuclear	cellular component biogenesis; vesicle coating; cellular component organization; cellular macromolecular complex assembly; protein complex subunit organization; vesicle-mediated transport; vesicle organization; clathrin coat assembly; establishment of localization; macromolecular complex subunit organization; transport; cellular component assembly; organelle organization; macromolecular complex assembly; membrane budding; membrane organization; localization; protein complex biogenesis; cellular process; protein complex assembly; cellular component organization or biogenesis; cellular protein complex assembly;	membrane; membrane part; cell part; intracellular; coated membrane; cytoplasm; clathrin coat; macromolecular complex; membrane coat; intracellular part; protein complex; cell; cytoplasmic part;	1-phosphatidylinositol binding; binding; phospholipid binding; phosphatidylinositol binding; ion binding; lipid binding; anion binding;				IPR013809; IPR011417; IPR008942; IPR014712;	Epsin-like, N-terminal; AP180 N-terminal homology (ANTH) domain; ENTH/VHS; Clathrin adaptor, phosphoinositide-binding, GAT-like;
Q7ZV77	psma4	Proteasome subunit alpha type	_TMDVSK(cr)LSAEK_	205	1	NKTMDVSKLSAEKVE		mitochondria	modification-dependent macromolecule catabolic process; cellular macromolecule catabolic process; proteolysis involved in cellular protein catabolic process; cellular protein catabolic process; modification-dependent protein catabolic process; cellular protein metabolic process; ubiquitin-dependent protein catabolic process; primary metabolic process; cellular metabolic process; organic substance metabolic process; organic substance catabolic process; macromolecule catabolic process; metabolic process; protein catabolic process; macromolecule catabolic process; cellular macromolecule metabolic process; cellular catabolic process; proteolysis; protein metabolic process; macromolecule metabolic process; cellular process; catabolic process;	intracellular organelle; proteasome core complex, alpha-subunit complex; proteasome complex; cytoplasm; macromolecular complex; intracellular part; cell; intracellular membrane-bounded organelle; organelle; nucleus; intracellular; cell part; proteasome core complex; membrane-bounded organelle; protein complex;	endopeptidase activity; threonine-type endopeptidase activity; peptidase activity; catalytic activity; threonine-type peptidase activity; peptidase activity, acting on L-amino acid peptides; hydrolase activity;	K02728	PSMA4; 20S proteasome subunit alpha 3 [EC:3.4.25.1]	dre03050 Proteasome - Danio rerio (zebrafish);	IPR029055; IPR000426;	Nucleophile aminohydrolases, N-terminal; Proteasome alpha-subunit, N-terminal domain;
Q6TNU6	dldh	Dihydrolipoyl dehydrogenase	_TNADTDGLVK(cr)ILSHK_	438	1	ADTDGLVKILSHKDT	.......K.L.....	mitochondria	regulation of cellular process; homeostatic process; regulation of biological quality; regulation of biological process; cell redox homeostasis; single-organism cellular process; cellular homeostasis; single-organism process; cellular process; biological regulation;	intracellular part; cell; cell part; intracellular; cytoplasm;	dihydrolipoyl dehydrogenase activity; oxidoreductase activity, acting on NAD(P)H; nucleoside phosphate binding; flavin adenine dinucleotide binding; ion binding; nucleotide binding; cofactor binding; oxidoreductase activity, acting on a sulfur group of donors, NAD(P) as acceptor; oxidoreductase activity; heterocyclic compound binding; organic cyclic compound binding; catalytic activity; binding; oxidoreductase activity, acting on a sulfur group of donors; anion binding; small molecule binding; coenzyme binding;	K00382	DLD; dihydrolipoamide dehydrogenase [EC:1.8.1.4]	dre00010 Glycolysis / Gluconeogenesis - Danio rerio (zebrafish); dre01200 Carbon metabolism - Danio rerio (zebrafish); dre01100 Metabolic pathways - Danio rerio (zebrafish); dre00620 Pyruvate metabolism - Danio rerio (zebrafish); dre00260 Glycine, serine and threonine metabolism - Danio rerio (zebrafish); dre00020 Citrate cycle (TCA cycle) - Danio rerio (zebrafish); dre00280 Valine, leucine and isoleucine degradation - Danio rerio (zebrafish);	IPR016156; IPR004099; IPR023753;	FAD/NAD-linked reductase, dimerisation domain; Pyridine nucleotide-disulphide oxidoreductase, dimerisation domain; Pyridine nucleotide-disulphide oxidoreductase, FAD/NAD(P)-binding domain;

E7F4R9	-	Isocitrate dehydrogenase [NADP]	_TNLDK(cr)ALGK_	443	1	AIKTNLDKALGK___		mitochondria	tricarboxylic acid metabolic process; oxidation-reduction process; small molecule metabolic process; tricarboxylic acid cycle; single-organism cellular process; single-organism process; isocitrate metabolic process; primary metabolic process; cellular respiration; cellular metabolic process; organic substance metabolic process; citrate metabolic process; metabolic process; generation of precursor metabolites and energy; aerobic respiration; single-organism metabolic process; energy derivation by oxidation of organic compounds; carboxylic acid metabolic process; organic acid metabolic process; cellular process; oxoacid metabolic process;		magnesium ion binding; nucleoside phosphate binding; ion binding; isocitrate dehydrogenase activity; nucleotide binding; cofactor binding; oxidoreductase activity; oxidoreductase activity, acting on CH-OH group of donors; heterocyclic compound binding; organic cyclic compound binding; oxidoreductase activity, acting on the CH-OH group of donors, NAD or NADP as acceptor; catalytic activity; NAD binding; binding; isocitrate dehydrogenase (NADP+) activity; metal ion binding; cation binding; small molecule binding; coenzyme binding;	K00031	IDH1; isocitrate dehydrogenase [EC:1.1.1.42]	dre01210 2-Oxocarboxylic acid metabolism - Danio rerio (zebrafish); dre01230 Biosynthesis of amino acids - Danio rerio (zebrafish); dre01200 Carbon metabolism - Danio rerio (zebrafish); dre01100 Metabolic pathways - Danio rerio (zebrafish); dre00480 Glutathione metabolism - Danio rerio (zebrafish); dre04146 Peroxisome - Danio rerio (zebrafish); dre00020 Citrate cycle (TCA cycle) - Danio rerio (zebrafish);	IPR024084;	Isopropylmalate dehydrogenase-like domain;
E9QGC9	myoz1b	Uncharacterized protein	_TPK(cr)DVMLEELSLMK_	51	1	GTKIKTPKDVMLEEL	.......K.V.....	nuclear								
Q5U3A4	atp2a1	Calcium-transporting ATPase	_TPLQQK(cr)LDEFGEQLSK_	252	1	EKTPLQQKLDEFGEQ		plasma membrane	regulation of biological quality; nucleoside metabolic process; purine nucleoside metabolic process; cellular divalent inorganic cation homeostasis; purine ribonucleoside monophosphate metabolic process; nucleotide metabolic process; positive regulation of muscle contraction; negative regulation of multicellular organismal process; single-organism process; purine ribonucleoside biosynthetic process; nucleoside biosynthetic process; biosynthetic process; ribonucleoside monophosphate biosynthetic process; cellular metabolic process; phosphate-containing compound metabolic process; response to external stimulus; ribose phosphate biosynthetic process; metabolic process; carbohydrate derivative biosynthetic process; positive regulation of multicellular organismal process; nucleoside phosphate metabolic process; regulation of skeletal muscle contraction; metal ion homeostasis; regulation of system process; ribose phosphate metabolic process; purine nucleoside triphosphate biosynthetic process; heterocycle biosynthetic process; purine ribonucleoside triphosphate biosynthetic process; ion homeostasis; calcium ion homeostasis; small molecule metabolic process; negative regulation of striated muscle contraction; inorganic ion	membrane; endoplasmic reticulum; nuclear outer membrane-endoplasmic reticulum membrane network; organelle membrane; myofibril; cytoplasm; intrinsic component of membrane; intracellular part; sarcoplasm; cell; I band; organelle part; non-membrane-bounded organelle; intracellular; cell part; membrane part; contractile fiber; membrane region; perinuclear region of cytoplasm; membrane-bounded organelle; endoplasmic reticulum membrane; intracellular organelle; bounding membrane of organelle; intracellular non-membrane-bounded organelle; H zone; intracellular organelle part; sarcoplasmic reticulum; endomembrane system; integral component of membrane; organelle; intracellular membrane-bounded organelle; endoplasmic reticulum part; endoplasmic reticulum-Golgi intermediate compartment; A band; sarcomere; contractile fiber part; cytoplasmic part;	ATPase activity, coupled to transmembrane movement of ions; nucleoside phosphate binding; primary active transmembrane transporter activity; substrate-specific transporter activity; hydrolase activity, acting on acid anhydrides; hydrolase activity; substrate-specific transmembrane transporter activity; ATPase activity; organic cyclic compound binding; heterocyclic compound binding; metal ion transmembrane transporter activity; pyrophosphatase activity; cation-transporting ATPase activity; catalytic activity; ribonucleotide binding; ATPase activity, coupled to movement of substances; binding; purine ribonucleoside binding; cation binding; hydrolase activity, acting on acid anhydrides, catalyzing transmembrane movement of substances; anion binding; adenyl ribonucleotide binding; adenyl nucleotide binding; inorganic cation transmembrane transporter activity; hydrolase activity, acting on acid anhydrides, in phosphorus-containing anhydrides; transporter activity; purine nucleotide binding; purine ribonucleoside triphosphate binding; active transmembrane transporter activity; ATP binding; ion transmembrane transporter activity; ion binding; nucleoside binding; nucleotide binding; nucleoside-triphosphatase activity; ribonucleoside binding; P-P-bond-hydrolysis-driven transmembrane transporter activity; ATPase activity, coupled to transmembrane movement of ions, phosphorylative mechanism; divalent inorganic cation transmembrane transporter activity; carbohydrate derivative binding; calcium-transporting ATPase activity; ATPase activity, coupled to transmembrane movement of substances; ATPase activity, coupled; calcium ion transmembrane transporter activity; metal ion binding; purine ribonucleotide binding; purine nucleoside binding; calcium ion binding; cation transmembrane transporter activity; transmembrane transporter activity; small molecule binding;	K05853	ATP2A; Ca2+ transporting ATPase, sarcoplasmic/endoplasmic reticulum [EC:3.6.3.8]	dre04020 Calcium signaling pathway - Danio rerio (zebrafish);	IPR004014; IPR023298; IPR008250; IPR023299; IPR006068; IPR023214;	Cation-transporting P-type ATPase, N-terminal; P-type ATPase, transmembrane domain; P-type ATPase, A domain; P-type ATPase, cytoplasmic domain N; Cation-transporting P-type ATPase, C-terminal; HAD-like domain;
I3ISK4	gnb3b	Uncharacterized protein	_TPVIK(cr)TVK_	132	1	NLKTPVIKTVKELDA	.......K.V.....	cytosol							IPR017986; IPR015943; IPR001632;	WD40-repeat-containing domain; WD40/YVTN repeat-like-containing domain; G-protein, beta subunit;
Q6XG62	icn	Protein S100	_TTDK(cr)AALDK_	53	1	IFGKTTDKAALDKIF	......DK.......	cytosol			cation binding; metal ion binding; calcium ion binding; binding; ion binding;				IPR011992; IPR002048; IPR013787;	EF-hand domain pair; EF-hand domain; S100/CaBP-9k-type, calcium binding, subdomain;
F1R8T2	-	Protein S100 (Fragment)	_TTDK(cr)ASLDNIFK_	53	1	VFGKTTDKASLDNIF	......DK.......	cytosol			cation binding; metal ion binding; calcium ion binding; binding; ion binding;				IPR002048; IPR011992; IPR013787;	EF-hand domain; EF-hand domain pair; S100/CaBP-9k-type, calcium binding, subdomain;
Q9I8U7	mylz3	Fast skeletal muscle myosin light polypeptide 3	_TVDANQK(cr)GTYDDYVEGLR_	83	1	KTVDANQKGTYDDYV		cytosol			cation binding; metal ion binding; calcium ion binding; binding; ion binding;				IPR011992; IPR002048;	EF-hand domain pair; EF-hand domain;
Q6IQX1	myhz2	Myosin, heavy polypeptide 2, fast muscle specific	_TVEDQLSEIK(cr)SK_	1262	1	EDQLSEIKSKNDENL		cytosol	response to stress; response to stimulus;	intracellular organelle; intracellular non-membrane-bounded organelle; cytoskeletal part; intracellular organelle part; macromolecular complex; intracellular part; cell; actin cytoskeleton; organelle part; non-membrane-bounded organelle; organelle; cytoskeleton; cell part; intracellular; myosin complex; protein complex;	ATP binding; motor activity; nucleoside phosphate binding; nucleoside binding; ion binding; hydrolase activity, acting on acid anhydrides; hydrolase activity; nucleotide binding; nucleoside-triphosphatase activity; organic cyclic compound binding; heterocyclic compound binding; ribonucleoside binding; carbohydrate derivative binding; pyrophosphatase activity; catalytic activity; ribonucleotide binding; binding; purine ribonucleoside binding; anion binding; purine ribonucleotide binding; adenyl ribonucleotide binding; adenyl nucleotide binding; purine nucleoside binding; hydrolase activity, acting on acid anhydrides, in phosphorus-containing anhydrides; purine nucleotide binding; small molecule binding; purine ribonucleoside triphosphate binding;	K10352	MYH; myosin heavy chain	dre04530 Tight junction - Danio rerio (zebrafish);	IPR002928; IPR004009; IPR001609; IPR027417; IPR027401;	Myosin tail; Myosin, N-terminal, SH3-like; Myosin head, motor domain; P-loop containing nucleoside triphosphate hydrolase; Myosin-like IQ motif-containing domain;
F1R8W4	desma	Uncharacterized protein	_TVLIK(cr)TIETR_	452	1	SKKTVLIKTIETRDG	.......K.I.....	mitochondria		intracellular organelle; intracellular non-membrane-bounded organelle; cytoskeletal part; intracellular organelle part; macromolecular complex; intracellular part; cell; organelle part; non-membrane-bounded organelle; organelle; cytoskeleton; intermediate filament cytoskeleton; cell part; intracellular; intermediate filament; protein complex;	structural molecule activity;	K07610			IPR006821;	Intermediate filament head, DNA-binding domain;
F6NT71	hbbe1.1	Uncharacterized protein	_TVLK(cr)GLELAVK_	11	1	AHGKTVLKGLELAVK	.......K.L.....	cytosol							IPR009050; IPR000971; IPR012292;	Globin-like; Globin; Globin, structural domain;
O93548	bE1	Embryonic 1 beta-globin	_TVLK(cr)GLELAVK_	70	1	AHGKTVLKGLELAVK	.......K.L.....	cytosol		cell part; intracellular; hemoglobin complex; cytoplasm; macromolecular complex; intracellular part; cytosolic part; protein complex; cell; cytosol; cytoplasmic part;	organic cyclic compound binding; heterocyclic compound binding; heme binding; iron ion binding; transition metal ion binding; oxygen binding; binding; ion binding; substrate-specific transporter activity; metal ion binding; cation binding; oxygen transporter activity; tetrapyrrole binding; transporter activity;				IPR009050; IPR000971; IPR012292;	Globin-like; Globin; Globin, structural domain;

P13104	tpma	Tropomyosin alpha-1 chain	_TVTNNMK(cr)SLEAQAEK_	205	1	KTVTNNMKSLEAQAE	.......K.L.....	cytosol		cytoskeleton; organelle; intracellular organelle; intracellular non-membrane-bounded organelle; cell part; intracellular; cytoplasm; intracellular part; cell; non-membrane-bounded organelle;		K10373	TPM1; tropomyosin 1	dre04260 Cardiac muscle contraction - Danio rerio (zebrafish); dre04261 Adrenergic signaling in cardiomyocytes - Danio rerio [illegible]		
Q7ZV82	rpl27	60S ribosomal protein L27	_TVVNK(cr)DVFR_	98	1	LDKTVVNKDVFRDPA	.......K.V.....	cytosol	cellular protein metabolic process; biosynthetic process; primary metabolic process; cellular metabolic process; organic substance biosynthetic process; cellular biosynthetic process; organic substance metabolic process; metabolic process; cellular macromolecule biosynthetic process; cellular macromolecule metabolic process; gene expression; macromolecule metabolic process; protein metabolic process; macromolecule biosynthetic process; translation; cellular process;	ribosome; organelle; intracellular organelle; intracellular non-membrane-bounded organelle; cell part; intracellular; cytoplasm; macromolecular complex; intracellular part; cell; ribonucleoprotein complex; cytoplasmic part; non-membrane-bounded organelle;	structural constituent of ribosome; structural molecule activity;	K02901	RP-L27e; large subunit ribosomal protein L27e	dre03010 Ribosome - Danio rerio (zebrafish);	IPR008991; IPR014722; IPR005824;	Translation protein SH3-like domain; Ribosomal protein L2 domain 2; KOW;
B8A561	myhz1.2	Uncharacterized protein	_VAK(cr)EDDVHPM(ox)NPPK_	74	1	TKEERVAKEDDVHPM	..E....K.......	nuclear		intracellular organelle; intracellular non-membrane-bounded organelle; cytoskeletal part; intracellular organelle part; macromolecular complex; intracellular part; cell; actin cytoskeleton; organelle part; non-membrane-bounded organelle; organelle; cytoskeleton; cell part; intracellular; myosin complex; protein complex;	ATP binding; motor activity; nucleoside phosphate binding; nucleoside binding; ion binding; hydrolase activity, acting on acid anhydrides; hydrolase activity; nucleotide binding; nucleoside-triphosphatase activity; organic cyclic compound binding; heterocyclic compound binding; ribonucleoside binding; carbohydrate derivative binding; pyrophosphatase activity; catalytic activity; ribonucleotide binding; binding; purine ribonucleoside binding; anion binding; purine ribonucleotide binding; adenyl ribonucleotide binding; adenyl nucleotide binding; purine nucleoside binding; hydrolase activity, acting on acid anhydrides, in phosphorus-containing anhydrides; purine nucleotide binding; small molecule binding; purine ribonucleoside triphosphate binding;	K10352	MYH; myosin heavy chain	dre04530 Tight junction - Danio rerio (zebrafish);	IPR002928; IPR004009; IPR001609; IPR027417; IPR027401;	Myosin tail; Myosin, N-terminal, SH3-like; Myosin head, motor domain; P-loop containing nucleoside triphosphate hydrolase; Myosin-like IQ motif-containing domain;
B8A561	myhz1.2	Uncharacterized protein	_VAK(cr)EDDVHPMNPPK_	74	1	TKEERVAKEDDVHPM	..E....K.......	nuclear		intracellular organelle; intracellular non-membrane-bounded organelle; cytoskeletal part; intracellular organelle part; macromolecular complex; intracellular part; cell; actin cytoskeleton; organelle part; non-membrane-bounded organelle; organelle; cytoskeleton; cell part; intracellular; myosin complex; protein complex;	ATP binding; motor activity; nucleoside phosphate binding; nucleoside binding; ion binding; hydrolase activity, acting on acid anhydrides; hydrolase activity; nucleotide binding; nucleoside-triphosphatase activity; organic cyclic compound binding; heterocyclic compound binding; ribonucleoside binding; carbohydrate derivative binding; pyrophosphatase activity; catalytic activity; ribonucleotide binding; binding; purine ribonucleoside binding; anion binding; purine ribonucleotide binding; adenyl ribonucleotide binding; adenyl nucleotide binding; purine nucleoside binding; hydrolase activity, acting on acid anhydrides, in phosphorus-containing anhydrides; purine nucleotide binding; small molecule binding; purine ribonucleoside triphosphate binding;	K10352	MYH; myosin heavy chain	dre04530 Tight junction - Danio rerio (zebrafish);	IPR002928; IPR004009; IPR001609; IPR027417; IPR027401;	Myosin tail; Myosin, N-terminal, SH3-like; Myosin head, motor domain; P-loop containing nucleoside triphosphate hydrolase; Myosin-like IQ motif-containing domain;
F1Q6U4	hbae3	Uncharacterized protein	_VAPK(cr)AEEIGR_	21	1	FFDKVAPKAEEIGRE	.......K.E.....	cytosol		cell part; intracellular; hemoglobin complex; cytoplasm; macromolecular complex; intracellular part; cytosolic part; protein complex; cell; cytosol; cytoplasmic part;	organic cyclic compound binding; heterocyclic compound binding; heme binding; iron ion binding; transition metal ion binding; oxygen binding; binding; ion binding; substrate-specific transporter activity; metal ion binding; cation binding; oxygen transporter activity; tetrapyrrole binding; transporter activity;				IPR009050; IPR000971; IPR012292;	Globin-like; Globin; Globin, structural domain;
Q7SXN2	hnrpdl	Heterogeneous nuclear ribonucleoprotein D-like	_VAQPK(cr)EVYR_	193	1	EIKVAQPKEVYRQQQ	.......K.V.....	nuclear		macromolecular complex; intracellular part; ribonucleoprotein complex; cell; cell part; intracellular;	organic cyclic compound binding; heterocyclic compound binding; nucleoside phosphate binding; nucleic acid binding; nucleotide binding; small molecule binding;	K13044			IPR000504; IPR012677;	RNA recognition motif domain; Nucleotide-binding, alpha-[illegible]
Q9I8U9	tnnt3a	Fast skeletal muscle troponin T	VDFDDIQK(cr)K	38	1	VDFDDIQKKRHNKDT		nuclear								
E7EXP0	tnnt3b	Uncharacterized protein	VEELSK(cr)YSK	220	1	KRVEELSKYSKKGAA		nuclear				K12046				
A5WWJ4	matn1	Uncharacterized protein	_VFLAK(cr)VIDGLSVGPDATR_	63	1	QVKVFLAKVIDGLSV	.......K.I.....	extracellular			cation binding; metal ion binding; calcium ion binding; binding; ion binding;				IPR019466; IPR000742; IPR002035;	Matrilin, coiled-coil trimerisation domain; Epidermal growth factor-like domain; von Willebrand factor, type A;
Q803M8	ywhaqb	Tryosine 3-monooxygenase/tryptophan 5-monooxygenase activation protein, zeta polypeptide	_VFYLK(cr)MK_	120	1	ESKVFYLKMKGDYYR		cytosol			monooxygenase activity; catalytic activity; oxidoreductase activity;	K16197	YWHAB_Q_Z; 14-3-3 protein beta/theta/zeta	dre04110 Cell cycle - Danio rerio (zebrafish); dre04114 Oocyte meiosis - Danio rerio (zebrafish);	IPR023410;	14-3-3 domain;
E9QJ96	ywhaba	14-3-3 protein beta/alpha-A	VFYLK(cr)MK	120	1	ESKVFYLKMKGDYYR		cytosol							IPR023410;	14-3-3 domain;
Q9I8V0	pvalb2	Parvalbumin-2	_VGLSAK(cr)SPDDIK_	39	1	AKVGLSAKSPDDIKK		cytosol			cation binding; metal ion binding; calcium ion binding; binding; ion binding;				IPR002048; IPR011992;	EF-hand domain; EF-hand domain pair;
G1K2X0	ttnb	Uncharacterized protein (Fragment)	_VHEK(cr)NLR_	25335	1	EWTKVHEKNLRVTEH	.......K.L.....				ATP binding; nucleoside phosphate binding; phosphotransferase activity, alcohol group as acceptor; nucleoside binding; ion binding; protein tyrosine kinase activity; protein kinase activity; nucleotide binding; organic cyclic compound binding; heterocyclic compound binding; ribonucleoside binding; carbohydrate derivative binding; kinase activity; catalytic activity; ribonucleotide binding; binding; nucleic acid binding; purine ribonucleoside binding; anion binding; purine ribonucleotide binding; adenyl ribonucleotide binding; adenyl nucleotide binding; purine nucleoside binding; transferase activity; transferase activity, transferring phosphorus-containing groups; purine nucleotide binding; small molecule binding; purine ribonucleoside triphosphate binding;	K12567			IPR003598; IPR007110; IPR000719; IPR013783; IPR003599; IPR003961; IPR013098; IPR011009;	Immunoglobulin subtype 2; Immunoglobulin-like domain; Protein kinase domain; Immunoglobulin-like fold; Immunoglobulin subtype; Fibronectin, type III; Immunoglobulin I-set; Protein kinase-like domain;
G1K2X0	ttnb	Uncharacterized protein (Fragment)	_VHHYVVEK(cr)R_	24446	1	VHHYVVEKREASRRT	.......K.E.....				ATP binding; nucleoside phosphate binding; phosphotransferase activity, alcohol group as acceptor; nucleoside binding; ion binding; protein tyrosine kinase activity; protein kinase activity; nucleotide binding; organic cyclic compound binding; heterocyclic compound binding; ribonucleoside binding; carbohydrate derivative binding; kinase activity; catalytic activity; ribonucleotide binding; binding; nucleic acid binding; purine ribonucleoside binding; anion binding; purine ribonucleotide binding; adenyl ribonucleotide binding; adenyl nucleotide binding; purine nucleoside binding; transferase activity; transferase activity, transferring phosphorus-containing groups; purine nucleotide binding; small molecule binding; purine ribonucleoside triphosphate binding;	K12567			IPR003598; IPR007110; IPR000719; IPR013783; IPR003599; IPR003961; IPR013098; IPR011009;	Immunoglobulin subtype 2; Immunoglobulin-like domain; Protein kinase domain; Immunoglobulin-like fold; Immunoglobulin subtype; Fibronectin, type III; Immunoglobulin I-set; Protein kinase-like domain;
F1RB19	ybx1	Uncharacterized protein	_VIATK(cr)VLGTVK_	38	1	DKKVIATKVLGTVKW	.......K.L.....	nuclear								

B0R198	vdac3	Uncharacterized protein	_VK(cr)ELGLSLNQK_	90	1	LETKYKVKELGLSLN	.......K.L.....	cytosol		membrane; intracellular organelle; outer membrane; mitochondrial part; bounding membrane of organelle; organelle membrane; mitochondrial outer membrane; cytoplasm; intracellular organelle part; organelle envelope; intracellular part; cell; organelle part; mitochondrion; organelle; mitochondrial envelope; intracellular membrane-bounded organelle; intracellular; cell part; envelope; organelle outer membrane; membrane-bounded organelle; mitochondrial membrane; cytoplasmic part;	ion transmembrane transporter activity; passive transmembrane transporter activity; anion channel activity; substrate-specific transporter activity; channel activity; substrate-specific transmembrane transporter activity; voltage-gated ion channel activity; voltage-gated anion channel activity; substrate-specific channel activity; ion channel activity; gated channel activity; voltage-gated channel activity; transmembrane transporter activity; transporter activity; anion transmembrane transporter activity;				IPR023614;	Porin domain;
B8A568	myhz1.1	Uncharacterized protein	_VK(cr)ELTYQTEEDKK_	1850	1	RKYERRVKELTYQTE	.......K.L.....	nuclear		intracellular organelle; intracellular non-membrane-bounded organelle; cytoskeletal part; intracellular organelle part; macromolecular complex; intracellular part; cell; actin cytoskeleton; organelle part; non-membrane-bounded organelle; organelle; cytoskeleton; cell part; intracellular; myosin complex; protein complex;	ATP binding; motor activity; nucleoside phosphate binding; nucleoside binding; ion binding; hydrolase activity, acting on acid anhydrides; hydrolase activity; nucleotide binding; nucleoside-triphosphatase activity; organic cyclic compound binding; heterocyclic compound binding; ribonucleoside binding; carbohydrate derivative binding; pyrophosphatase activity; catalytic activity; ribonucleotide binding; binding; purine ribonucleoside binding; anion binding; purine ribonucleotide binding; adenyl ribonucleotide binding; adenyl nucleotide binding; purine nucleoside binding; hydrolase activity, acting on acid anhydrides, in phosphorus-containing anhydrides; purine nucleotide binding; small molecule binding; purine ribonucleoside triphosphate binding;	K10352	MYH; myosin heavy chain	dre04530 Tight junction - Danio rerio (zebrafish);	IPR002928; IPR004009; IPR001609; IPR027417; IPR027401;	Myosin tail; Myosin, N-terminal, SH3-like; Myosin head, motor domain; P-loop containing nucleoside triphosphate hydrolase; Myosin-like IQ motif-containing domain;
B8A561	myhz1.2	Uncharacterized protein	_VK(cr)ELTYQTEEDKK_	1850	1	RKYERRVKELTYQTE	.......K.L.....	nuclear		intracellular organelle; intracellular non-membrane-bounded organelle; cytoskeletal part; intracellular organelle part; macromolecular complex; intracellular part; cell; actin cytoskeleton; organelle part; non-membrane-bounded organelle; organelle; cytoskeleton; cell part; intracellular; myosin complex; protein complex;	ATP binding; motor activity; nucleoside phosphate binding; nucleoside binding; ion binding; hydrolase activity, acting on acid anhydrides; hydrolase activity; nucleotide binding; nucleoside-triphosphatase activity; organic cyclic compound binding; heterocyclic compound binding; ribonucleoside binding; carbohydrate derivative binding; pyrophosphatase activity; catalytic activity; ribonucleotide binding; binding; purine ribonucleoside binding; anion binding; purine ribonucleotide binding; adenyl ribonucleotide binding; adenyl nucleotide binding; purine nucleoside binding; hydrolase activity, acting on acid anhydrides, in phosphorus-containing anhydrides; purine nucleotide binding; small molecule binding; purine ribonucleoside triphosphate binding;	K10352	MYH; myosin heavy chain	dre04530 Tight junction - Danio rerio (zebrafish);	IPR002928; IPR004009; IPR001609; IPR027417; IPR027401;	Myosin tail; Myosin, N-terminal, SH3-like; Myosin head, motor domain; P-loop containing nucleoside triphosphate hydrolase; Myosin-like IQ motif-containing domain;
G1K2X0	ttnb	Uncharacterized protein (Fragment)	_VK(cr)IIDVSK_	11960	1	PDPPTRVKIIDVSKG	.......K.I.....				ATP binding; nucleoside phosphate binding; phosphotransferase activity, alcohol group as acceptor; nucleoside binding; ion binding; protein tyrosine kinase activity; protein kinase activity; nucleotide binding; organic cyclic compound binding; heterocyclic compound binding; ribonucleoside binding; carbohydrate derivative binding; kinase activity; catalytic activity; ribonucleotide binding; binding; nucleic acid binding; purine ribonucleoside binding; anion binding; purine ribonucleotide binding; adenyl ribonucleotide binding; adenyl nucleotide binding; purine nucleoside binding; transferase activity; transferase activity, transferring phosphorus-containing groups; purine nucleotide binding; small molecule binding; purine ribonucleoside triphosphate binding;	K12567			IPR003598; IPR007110; IPR000719; IPR013783; IPR003599; IPR003961; IPR013098; IPR011009;	Immunoglobulin subtype 2; Immunoglobulin-like domain; Protein kinase domain; Immunoglobulin-like fold; Immunoglobulin subtype; Fibronectin, type III; Immunoglobulin I-set; Protein kinase-like domain;
Q6IQX1	myhz2	Uncharacterized protein	_VK(cr)NLTEEM(ox)ASQDESIAK_	978	1	HATENKVKNLTEEMA	.......K.L.....	cytosol	response to stress; response to stimulus;	intracellular organelle; intracellular non-membrane-bounded organelle; cytoskeletal part; intracellular organelle part; macromolecular complex; intracellular part; cell; actin cytoskeleton; organelle part; non-membrane-bounded organelle; organelle; cytoskeleton; cell part; intracellular; myosin complex; protein complex;	ATP binding; motor activity; nucleoside phosphate binding; nucleoside binding; ion binding; hydrolase activity, acting on acid anhydrides; hydrolase activity; nucleotide binding; nucleoside-triphosphatase activity; organic cyclic compound binding; heterocyclic compound binding; ribonucleoside binding; carbohydrate derivative binding; pyrophosphatase activity; catalytic activity; ribonucleotide binding; binding; purine ribonucleoside binding; anion binding; purine ribonucleotide binding; adenyl ribonucleotide binding; adenyl nucleotide binding; purine nucleoside binding; hydrolase activity, acting on acid anhydrides, in phosphorus-containing anhydrides; purine nucleotide binding; small molecule binding; purine ribonucleoside triphosphate binding;	K10352	MYH; myosin heavy chain	dre04530 Tight junction - Danio rerio (zebrafish);	IPR002928; IPR004009; IPR001609; IPR027417; IPR027401;	Myosin tail; Myosin, N-terminal, SH3-like; Myosin head, motor domain; P-loop containing nucleoside triphosphate hydrolase; Myosin-like IQ motif-containing domain;
B8A568	myhz1.1	Uncharacterized protein	_VK(cr)NLTEEMAAQDESIGK_	978	1	HATENKVKNLTEEMA	.......K.L.....	nuclear		intracellular organelle; intracellular non-membrane-bounded organelle; cytoskeletal part; intracellular organelle part; macromolecular complex; intracellular part; cell; actin cytoskeleton; organelle part; non-membrane-bounded organelle; organelle; cytoskeleton; cell part; intracellular; myosin complex; protein complex;	ATP binding; motor activity; nucleoside phosphate binding; nucleoside binding; ion binding; hydrolase activity, acting on acid anhydrides; hydrolase activity; nucleotide binding; nucleoside-triphosphatase activity; organic cyclic compound binding; heterocyclic compound binding; ribonucleoside binding; carbohydrate derivative binding; pyrophosphatase activity; catalytic activity; ribonucleotide binding; binding; purine ribonucleoside binding; anion binding; purine ribonucleotide binding; adenyl ribonucleotide binding; adenyl nucleotide binding; purine nucleoside binding; hydrolase activity, acting on acid anhydrides, in phosphorus-containing anhydrides; purine nucleotide binding; small molecule binding; purine ribonucleoside triphosphate binding;	K10352	MYH; myosin heavy chain	dre04530 Tight junction - Danio rerio (zebrafish);	IPR002928; IPR004009; IPR001609; IPR027417; IPR027401;	Myosin tail; Myosin, N-terminal, SH3-like; Myosin head, motor domain; P-loop containing nucleoside triphosphate hydrolase; Myosin-like IQ motif-containing domain;
B8A561	myhz1.2	Uncharacterized protein	_VK(cr)NLTEEMAAQDESIGK_	978	1	HATENKVKNLTEEMA	.......K.L.....	nuclear		intracellular organelle; intracellular non-membrane-bounded organelle; cytoskeletal part; intracellular organelle part; macromolecular complex; intracellular part; cell; actin cytoskeleton; organelle part; non-membrane-bounded organelle; organelle; cytoskeleton; cell part; intracellular; myosin complex; protein complex;	ATP binding; motor activity; nucleoside phosphate binding; nucleoside binding; ion binding; hydrolase activity, acting on acid anhydrides; hydrolase activity; nucleotide binding; nucleoside-triphosphatase activity; organic cyclic compound binding; heterocyclic compound binding; ribonucleoside binding; carbohydrate derivative binding; pyrophosphatase activity; catalytic activity; ribonucleotide binding; binding; purine ribonucleoside binding; anion binding; purine ribonucleotide binding; adenyl ribonucleotide binding; adenyl nucleotide binding; purine nucleoside binding; hydrolase activity, acting on acid anhydrides, in phosphorus-containing anhydrides; purine nucleotide binding; small molecule binding; purine ribonucleoside triphosphate binding;	K10352	MYH; myosin heavy chain	dre04530 Tight junction - Danio rerio (zebrafish);	IPR002928; IPR004009; IPR001609; IPR027417; IPR027401;	Myosin tail; Myosin, N-terminal, SH3-like; Myosin head, motor domain; P-loop containing nucleoside triphosphate hydrolase; Myosin-like IQ motif-containing domain;

Q6IQX1	myhz2	Uncharacterized protein (Fragment)	_VK(cr)NLTEEMASQDESIAK_	978	1	HATENKVKNLTEEMA	.......K.L.....	cytosol	response to stress; response to stimulus;	intracellular organelle; intracellular non-membrane-bounded organelle; cytoskeletal part; intracellular organelle part; macromolecular complex; intracellular part; cell; actin cytoskeleton; organelle part; non-membrane-bounded organelle; organelle; cytoskeleton; cell part; intracellular; myosin complex; protein complex;	ATP binding; motor activity; nucleoside phosphate binding; nucleoside binding; ion binding; hydrolase activity, acting on acid anhydrides; hydrolase activity; nucleotide binding; nucleoside-triphosphatase activity; organic cyclic compound binding; heterocyclic compound binding; ribonucleoside binding; carbohydrate derivative binding; pyrophosphatase activity; catalytic activity; ribonucleotide binding; binding; purine ribonucleoside binding; anion binding; purine ribonucleotide binding; adenyl ribonucleotide binding; adenyl nucleotide binding; purine nucleoside binding; hydrolase activity, acting on acid anhydrides, in phosphorus-containing anhydrides; purine nucleotide binding; small molecule binding; purine ribonucleoside triphosphate binding;	K10352	MYH; myosin heavy chain	dre04530 Tight junction - Danio rerio (zebrafish);	IPR002928; IPR004009; IPR001609; IPR027417; IPR027401;	Myosin tail; Myosin, N-terminal, SH3-like; Myosin head, motor domain; P-loop containing nucleoside triphosphate hydrolase; Myosin-like IQ motif-containing domain;
A2BGX6	myhc4	Uncharacterized protein	_VK(cr)NLTEEMASQDESIAK_	978	1	HATENKVKNLTEEMA	.......K.L.....	cytosol		intracellular organelle; intracellular non-membrane-bounded organelle; cytoskeletal part; intracellular organelle part; macromolecular complex; intracellular part; cell; actin cytoskeleton; organelle part; non-membrane-bounded organelle; organelle; cytoskeleton; cell part; intracellular; myosin complex; protein complex;	ATP binding; motor activity; nucleoside phosphate binding; nucleoside binding; ion binding; hydrolase activity, acting on acid anhydrides; hydrolase activity; nucleotide binding; nucleoside-triphosphatase activity; organic cyclic compound binding; heterocyclic compound binding; ribonucleoside binding; carbohydrate derivative binding; pyrophosphatase activity; catalytic activity; ribonucleotide binding; binding; purine ribonucleoside binding; anion binding; purine ribonucleotide binding; adenyl ribonucleotide binding; adenyl nucleotide binding; purine nucleoside binding; hydrolase activity, acting on acid anhydrides, in phosphorus-containing anhydrides; purine nucleotide binding; small molecule binding; purine ribonucleoside triphosphate binding;	K10352	MYH; myosin heavy chain	dre04530 Tight junction - Danio rerio (zebrafish);	IPR002928; IPR004009; IPR027417; IPR001609; IPR027401;	Myosin tail; Myosin, N-terminal, SH3-like; P-loop containing nucleoside triphosphate hydrolase; Myosin head, motor domain; Myosin-like IQ motif-containing domain;
Q5TZ29	apobb.1	Uncharacterized protein	_VK(cr)VVDVVK_	2320	1	IEKLEKVKVVDVVKK	.......K.V.....	endoplasmic reticulum	response to chemical; response to stimulus;		lipid transporter activity; substrate-specific transporter activity; transporter activity;				IPR011030; IPR015817; IPR015818; IPR001747; IPR015819; IPR015816; IPR016024; IPR015255; IPR009454;	Vitellinogen, superhelical; Vitellinogen, open beta-sheet, subdomain 1; Vitellinogen, open beta-sheet, subdomain 2; Lipid transport protein, N-terminal; Lipid transport protein, beta-sheet shell; Vitellinogen, beta-sheet N-terminal; Armadillo-type fold; Vitellinogen, open beta-sheet; Lipid transport, open beta-sheet;
B8A561	myhz1.2	Uncharacterized protein	_VLNASVIPEGQFIDNK(cr)K_	741	1	EGQFIDNKKASEKLL		nuclear		intracellular organelle; intracellular non-membrane-bounded organelle; cytoskeletal part; intracellular organelle part; macromolecular complex; intracellular part; cell; actin cytoskeleton; organelle part; non-membrane-bounded organelle; organelle; cytoskeleton; cell part; intracellular; myosin complex; protein complex;	ATP binding; motor activity; nucleoside phosphate binding; nucleoside binding; ion binding; hydrolase activity, acting on acid anhydrides; hydrolase activity; nucleotide binding; nucleoside-triphosphatase activity; organic cyclic compound binding; heterocyclic compound binding; ribonucleoside binding; carbohydrate derivative binding; pyrophosphatase activity; catalytic activity; ribonucleotide binding; binding; purine ribonucleoside binding; anion binding; purine ribonucleotide binding; adenyl ribonucleotide binding; adenyl nucleotide binding; purine nucleoside binding; hydrolase activity, acting on acid anhydrides, in phosphorus-containing anhydrides; purine nucleotide binding; small molecule binding; purine ribonucleoside triphosphate binding;	K10352	MYH; myosin heavy chain	dre04530 Tight junction - Danio rerio (zebrafish);	IPR002928; IPR004009; IPR001609; IPR027417; IPR027401;	Myosin tail; Myosin, N-terminal, SH3-like; Myosin head, motor domain; P-loop containing nucleoside triphosphate hydrolase; Myosin-like IQ motif-containing domain;
Q804W0	pvalb1	Parvalbumin 1	_VLTDK(cr)ETK_	81	1	NARVLTDKETKAFLS	......DK.......	cytosol			cation binding; metal ion binding; calcium ion binding; binding; ion binding;				IPR011992; IPR002048;	EF-hand domain pair; EF-hand domain;
P13104	tpma	Tropomyosin alpha-1 chain	_VLTDK(cr)LK_	231	1	EIKVLTDKLKEAETR	......DK.......	cytosol		cytoskeleton; organelle; intracellular organelle; intracellular non-membrane-bounded organelle; cell part; intracellular; cytoplasm; intracellular part; cell; non-membrane-bounded organelle;		K10373	TPM1; tropomyosin 1	dre04260 Cardiac muscle contraction - Danio rerio (zebrafish); dre04261 Adrenergic signaling in cardiomyocytes - Danio rerio (zebrafish);		
Q7T306	ckmb	Ckmb protein	_VLTK(cr)DIYNK_	35	1	HMSKVLTKDIYNKLR	.......K.I.....	cytosol			ATP binding; nucleoside phosphate binding; nucleoside binding; ion binding; nucleotide binding; organic cyclic compound binding; heterocyclic compound binding; ribonucleoside binding; carbohydrate derivative binding; kinase activity; catalytic activity; ribonucleotide binding; binding; purine ribonucleoside binding; anion binding; purine ribonucleotide binding; adenyl ribonucleotide binding; adenyl nucleotide binding; purine nucleoside binding; transferase activity; transferase activity, transferring phosphorus-containing groups; purine nucleotide binding; small molecule binding; purine ribonucleoside triphosphate binding;				IPR022413; IPR022414; IPR014746;	ATP:guanido phosphotransferase, N-terminal; ATP:guanido phosphotransferase, catalytic domain; Glutamine synthetase/guanido kinase, catalytic domain;
A2BHA3	ckma	Uncharacterized protein	_VLTK(cr)EMYGK_	36	1	HMAKVLTKEMYGKLR		cytosol			ATP binding; nucleoside phosphate binding; nucleoside binding; ion binding; nucleotide binding; organic cyclic compound binding; heterocyclic compound binding; ribonucleoside binding; carbohydrate derivative binding; kinase activity; catalytic activity; ribonucleotide binding; binding; purine ribonucleoside binding; anion binding; purine ribonucleotide binding; adenyl ribonucleotide binding; adenyl nucleotide binding; purine nucleoside binding; transferase activity; transferase activity, transferring phosphorus-containing groups; purine nucleotide binding; small molecule binding; purine ribonucleoside triphosphate binding;	K00933	E2.7.3.2; creatine kinase [EC:2.7.3.2]	dre00330 Arginine and proline metabolism - Danio rerio (zebrafish); dre01100 Metabolic pathways - Danio rerio (zebrafish);	IPR022413; IPR022414; IPR014746;	ATP:guanido phosphotransferase, N-terminal; ATP:guanido phosphotransferase, catalytic domain; Glutamine synthetase/guanido kinase, catalytic domain;
Q503C7	pygma	Alpha-1,4 glucan phosphorylase	_VLYPNDNFFEGK(cr)ELR_	290	1	NDNFFEGKELRLKQE	.......K.L.....	cytosol	metabolic process; carbohydrate metabolic process; primary metabolic process; organic substance metabolic process;		organic cyclic compound binding; heterocyclic compound binding; transferase activity, transferring hexosyl groups; catalytic activity; binding; ion binding; phosphorylase activity; anion binding; transferase activity, transferring glycosyl groups; cofactor binding; transferase activity; pyridoxal phosphate binding; glycogen phosphorylase activity;	K00688	E2.4.1.1; starch phosphorylase [EC:2.4.1.1]	dre01100 Metabolic pathways - Danio rerio (zebrafish); dre04910 Insulin signaling pathway - Danio rerio (zebrafish); dre00500 Starch and sucrose metabolism - Danio rerio (zebrafish);		
E9QD64	cct6a	Uncharacterized protein	_VMK(cr)IIDLK_	228	1	FIEDRVMKIIDLKNK	.......K.I.....	cytosol	protein folding; metabolic process; cellular protein metabolic process; cellular macromolecule metabolic process; macromolecule metabolic process; protein metabolic process; primary metabolic process; cellular process; cellular metabolic process; organic substance metabolic process;	intracellular part; cell; cell part; intracellular; cytoplasm;	ATP binding; nucleoside phosphate binding; nucleoside binding; ion binding; nucleotide binding; organic cyclic compound binding; heterocyclic compound binding; ribonucleoside binding; carbohydrate derivative binding; ribonucleotide binding; binding; purine ribonucleoside binding; anion binding; purine ribonucleotide binding; adenyl ribonucleotide binding; adenyl nucleotide binding; purine nucleoside binding; purine nucleotide binding; small molecule binding; purine ribonucleoside triphosphate binding;				IPR027410; IPR027409; IPR027413;	TCP-1-like chaperonin intermediate domain; GroEL-like apical domain; GroEL-like equatorial domain;

E9QBV1	cltcb	Clathrin heavy chain	_VNIPK(cr)VLR_	1347	1	WSRVNIPKVLRAAEQ	.......K.L.....	cytosol	establishment of protein localization; transport; intracellular transport; cellular macromolecule localization; establishment of localization in cell; vesicle-mediated transport; intracellular protein transport; organic substance transport; protein localization; macromolecule localization; cellular protein localization; localization; protein transport; establishment of localization; cellular localization;	transport vesicle membrane; membrane; clathrin coat of trans-Golgi network vesicle; cytoplasmic vesicle; organelle membrane; membrane-bounded vesicle; cell periphery; cytoplasmic membrane-bounded vesicle; vesicle; Golgi-associated vesicle membrane; cytoplasm; cytoplasmic vesicle part; macromolecular complex; intracellular part; vesicle coat; cell; organelle part; coated vesicle membrane; trans-Golgi network transport vesicle; clathrin-coated vesicle; membrane part; cell part; intracellular; Golgi apparatus part; trans-Golgi network transport vesicle membrane; clathrin coat of coated pit; membrane-bounded organelle; intracellular organelle; bounding membrane of organelle; coated membrane; intracellular organelle part; Golgi membrane; vesicle membrane; endomembrane system; coated vesicle; clathrin-coated vesicle membrane; Golgi-associated vesicle; plasma membrane; cytoplasmic vesicle membrane; intracellular membrane-bounded organelle; organelle; clathrin vesicle coat; plasma membrane part; clathrin coat; membrane coat; Golgi apparatus; transport vesicle; coated pit; protein complex; cytoplasmic part;	structural molecule activity;	K04646	CLTC; clathrin heavy chain	dre04144 Endocytosis - Danio rerio (zebrafish); dre04142 Lysosome - Danio rerio (zebrafish);	IPR016025; IPR015348; IPR011990; IPR016024; IPR001473;	Clathrin, heavy chain, linker/propeller domain; Clathrin, heavy chain, linker, core motif; Tetratricopeptide-like helical domain; Armadillo-type fold; Clathrin, heavy chain, propeller, N-terminal;
Q6IQK3	vamp3	Uncharacterized protein	_VNVDK(cr)VLER_	38	1	IMRVNVDKVLERDQK	......DK......	plasma membrane	transport; localization; vesicle-mediated transport; establishment of localization;	intrinsic component of membrane; membrane; integral component of membrane; membrane part;		K13505	VAMP3; vesicle-associated membrane protein 3	dre04145 Phagosome - Danio rerio (zebrafish); dre04130 SNARE interactions in vesicular transport - Danio rerio (zebrafish);	IPR001388;	Synaptobrevin;
Q6PBW7	rps19	Ribosomal protein S19	_VPDWVDIVK(cr)LAK_	39	1	PDWVDIVKLAKHKEL		cytosol	cellular protein metabolic process; biosynthetic process; primary metabolic process; cellular metabolic process; organic substance biosynthetic process; cellular biosynthetic process; organic substance metabolic process; metabolic process; cellular macromolecule biosynthetic process; cellular macromolecule metabolic process; gene expression; macromolecule metabolic process; protein metabolic process; macromolecule biosynthetic process; translation; cellular process;	ribosome; organelle; intracellular organelle; intracellular non-membrane-bounded organelle; cell part; intracellular; cytoplasm; macromolecular complex; intracellular part; cell; ribonucleoprotein complex; cytoplasmic part; non-membrane-bounded organelle;	structural constituent of ribosome; structural molecule activity;	K02966	RP-S19e; small subunit ribosomal protein S19e	dre03010 Ribosome - Danio rerio (zebrafish);		
Q6IQX1	myhz2	Uncharacterized protein	_VQLELNQVK(cr)GEIDR_	1571	1	QLELNQVKGEIDRKL	..E....K.......	cytosol	response to stress; response to stimulus;	intracellular organelle; intracellular non-membrane-bounded organelle; cytoskeletal part; intracellular organelle part; macromolecular complex; intracellular part; cell; actin cytoskeleton; organelle part; non-membrane-bounded organelle; organelle; cytoskeleton; cell part; intracellular; myosin complex; protein complex;	ATP binding; motor activity; nucleoside phosphate binding; nucleoside binding; ion binding; hydrolase activity, acting on acid anhydrides; hydrolase activity; nucleotide binding; nucleoside-triphosphatase activity; organic cyclic compound binding; heterocyclic compound binding; ribonucleoside binding; carbohydrate derivative binding; pyrophosphatase activity; catalytic activity; ribonucleotide binding; binding; purine ribonucleoside binding; anion binding; purine ribonucleotide binding; adenyl ribonucleotide binding; adenyl nucleotide binding; purine nucleoside binding; hydrolase activity, acting on acid anhydrides, in phosphorus-containing anhydrides; purine nucleotide binding; small molecule binding; purine ribonucleoside triphosphate binding;	K10352	MYH; myosin heavy chain	dre04530 Tight junction - Danio rerio (zebrafish);	IPR002928; IPR004009; IPR001609; IPR027417; IPR027401;	Myosin tail; Myosin, N-terminal, SH3-like; Myosin head, motor domain; P-loop containing nucleoside triphosphate hydrolase; Myosin-like IQ motif-containing domain;
B8A568	myhz1.1	Uncharacterized protein	_VQLELNQVK(cr)SEIDRK_	1571	1	QLELNQVKSEIDRKL	..E....K.......	nuclear		intracellular organelle; intracellular non-membrane-bounded organelle; cytoskeletal part; intracellular organelle part; macromolecular complex; intracellular part; cell; actin cytoskeleton; organelle part; non-membrane-bounded organelle; organelle; cytoskeleton; cell part; intracellular; myosin complex; protein complex;	ATP binding; motor activity; nucleoside phosphate binding; nucleoside binding; ion binding; hydrolase activity, acting on acid anhydrides; hydrolase activity; nucleotide binding; nucleoside-triphosphatase activity; organic cyclic compound binding; heterocyclic compound binding; ribonucleoside binding; carbohydrate derivative binding; pyrophosphatase activity; catalytic activity; ribonucleotide binding; binding; purine ribonucleoside binding; anion binding; purine ribonucleotide binding; adenyl ribonucleotide binding; adenyl nucleotide binding; purine nucleoside binding; hydrolase activity, acting on acid anhydrides, in phosphorus-containing anhydrides; purine nucleotide binding; small molecule binding; purine ribonucleoside triphosphate binding;	K10352	MYH; myosin heavy chain	dre04530 Tight junction - Danio rerio (zebrafish);	IPR002928; IPR004009; IPR001609; IPR027417; IPR027401;	Myosin tail; Myosin, N-terminal, SH3-like; Myosin head, motor domain; P-loop containing nucleoside triphosphate hydrolase; Myosin-like IQ motif-containing domain;
B8A561	myhz1.2	Uncharacterized protein	_VQLELNQVK(cr)SEIDRK_	1571	1	QLELNQVKSEIDRKL	..E....K.......	nuclear		intracellular organelle; intracellular non-membrane-bounded organelle; cytoskeletal part; intracellular organelle part; macromolecular complex; intracellular part; cell; actin cytoskeleton; organelle part; non-membrane-bounded organelle; organelle; cytoskeleton; cell part; intracellular; myosin complex; protein complex;	ATP binding; motor activity; nucleoside phosphate binding; nucleoside binding; ion binding; hydrolase activity, acting on acid anhydrides; hydrolase activity; nucleotide binding; nucleoside-triphosphatase activity; organic cyclic compound binding; heterocyclic compound binding; ribonucleoside binding; carbohydrate derivative binding; pyrophosphatase activity; catalytic activity; ribonucleotide binding; binding; purine ribonucleoside binding; anion binding; purine ribonucleotide binding; adenyl ribonucleotide binding; adenyl nucleotide binding; purine nucleoside binding; hydrolase activity, acting on acid anhydrides, in phosphorus-containing anhydrides; purine nucleotide binding; small molecule binding; purine ribonucleoside triphosphate binding;	K10352	MYH; myosin heavy chain	dre04530 Tight junction - Danio rerio (zebrafish);	IPR002928; IPR004009; IPR001609; IPR027417; IPR027401;	Myosin tail; Myosin, N-terminal, SH3-like; Myosin head, motor domain; P-loop containing nucleoside triphosphate hydrolase; Myosin-like IQ motif-containing domain;
B3DFN3	lmnb2	Lamin B2	_VSEK(cr)EEVTTR_	61	1	LQFKVSEKEEVTTRE	.......K.E.....	nuclear		intracellular organelle; intracellular non-membrane-bounded organelle; cytoskeletal part; intracellular organelle part; macromolecular complex; intracellular part; cell; organelle part; non-membrane-bounded organelle; organelle; cytoskeleton; intermediate filament cytoskeleton; cell part; intracellular; intermediate filament; protein complex;	structural molecule activity;	K07611			IPR001322;	Lamin Tail Domain;

Q08CK7	igf2bp1	Insulin-like growth factor 2 mRNA-binding protein 1	_VTAEDLVK(cr)TFEDYK_	20	1	VTAEDLVKTFEDYKI		cytosol,nuclear	regulation of cellular process; regulation of gene expression; regulation of cellular biosynthetic process; regulation of primary metabolic process; regulation of macromolecule metabolic process; posttranscriptional regulation of gene expression; regulation of macromolecule biosynthetic process; regulation of cellular metabolic process; regulation of biological process; regulation of cellular protein metabolic process; regulation of translation; regulation of protein metabolic process; regulation of biosynthetic process; regulation of metabolic process; biological regulation; regulation of cellular macromolecule biosynthetic process;	organelle; intracellular membrane-bounded organelle; intracellular organelle; nucleus; CRD-mediated mRNA stability complex; cell part; intracellular; cytoplasm; macromolecular complex; intracellular part; membrane-bounded organelle; protein complex; cell; cytoplasmic part;	organic cyclic compound binding; heterocyclic compound binding; RNA binding; mRNA binding; poly(A) RNA binding; nucleoside phosphate binding; mRNA 3'-UTR binding; binding; nucleic acid binding; nucleotide binding; small molecule binding;	K17391	IPR004087; IPR004088; IPR000504; IPR012677;	K Homology domain; K Homology domain, type 1; RNA recognition motif domain; Nucleotide-binding, alpha-beta plait;
Q4QRF4	si:dkeyp-46h3.6	Histone H3.2	_VTIM(ox)PK(cr)DIQLAR_	123	1	KRVTIMPKDIQLARR	.......K.I.....	nuclear	cellular component biogenesis; cellular component organization; nucleosome organization; cellular macromolecular complex assembly; chromatin assembly or disassembly; protein complex subunit organization; macromolecular complex subunit organization; protein-DNA complex assembly; DNA packaging; nucleosome assembly; chromosome organization; cellular component assembly; macromolecular complex assembly; organelle organization; chromatin organization; protein complex biogenesis; chromatin assembly; cellular process; protein complex assembly; cellular component organization or biogenesis; protein-DNA complex subunit organization; DNA conformation change;	intracellular organelle; intracellular non-membrane-bounded organelle; intracellular organelle part; macromolecular complex; intracellular part; cell; organelle part; DNA bending complex; chromosomal part; nucleosome; non-membrane-bounded organelle; intracellular membrane-bounded organelle; organelle; nucleus; chromatin; cell part; intracellular; chromosome; membrane-bounded organelle; protein complex; protein-DNA complex; DNA packaging complex;	organic cyclic compound binding; heterocyclic compound binding; DNA binding; binding; nucleic acid binding;	K11253	IPR009072; IPR007125;	Histone-fold; Histone core;
Q4QRF4	si:dkeyp-46h3.6	Histone H3.2	_VTIMPK(cr)DIQLAR_	123	1	KRVTIMPKDIQLARR	.......K.I.....	nuclear	cellular component biogenesis; cellular component organization; nucleosome organization; cellular macromolecular complex assembly; chromatin assembly or disassembly; protein complex subunit organization; macromolecular complex subunit organization; protein-DNA complex assembly; DNA packaging; nucleosome assembly; chromosome organization; cellular component assembly; macromolecular complex assembly; organelle organization; chromatin organization; protein complex biogenesis; chromatin assembly; cellular process; protein complex assembly; cellular component organization or biogenesis; protein-DNA complex subunit organization; DNA conformation change;	intracellular organelle; intracellular non-membrane-bounded organelle; intracellular organelle part; macromolecular complex; intracellular part; cell; organelle part; DNA bending complex; chromosomal part; nucleosome; non-membrane-bounded organelle; intracellular membrane-bounded organelle; organelle; nucleus; chromatin; cell part; intracellular; chromosome; membrane-bounded organelle; protein complex; protein-DNA complex; DNA packaging complex;	organic cyclic compound binding; heterocyclic compound binding; DNA binding; binding; nucleic acid binding;	K11253	IPR009072; IPR007125;	Histone-fold; Histone core;
Q6PC53	ppiab	Peptidyl-prolyl cis-trans isomerase	_VVDGLDVVDAIEK(cr)K_	144	1	DVVDAIEKKGSSSGK		cytosol	cellular protein metabolic process; protein peptidyl-prolyl isomerization; peptidyl-proline modification; primary metabolic process; cellular metabolic process; organic substance metabolic process; protein folding; peptidyl-amino acid modification; metabolic process; cellular macromolecule metabolic process; cellular protein modification process; protein metabolic process; macromolecule metabolic process; macromolecule modification; protein modification process; cellular process;		peptidyl-prolyl cis-trans isomerase activity; isomerase activity; catalytic activity; cis-trans isomerase activity;	K03767	IPR002130; IPR029000;	Cyclophilin-type peptidyl-prolyl cis-trans isomerase domain; Cyclophilin-like domain;
Q5TZ29	apobb.1	Uncharacterized protein	_VVDVVK(cr)K_	2326	1	VKVVDVVKKFSDIVD		endoplasmic reticulum	response to chemical; response to stimulus;		lipid transporter activity; substrate-specific transporter activity; transporter activity;		IPR011030; IPR015817; IPR015818; IPR001747; IPR015819; IPR015816; IPR016024; IPR015255; IPR009454;	Vitellinogen, superhelical; Vitellinogen, open beta-sheet, subdomain 1; Vitellinogen, open beta-sheet, subdomain 2; Lipid transport protein, N-terminal; Lipid transport protein, beta-sheet shell; Vitellinogen, beta-sheet N-terminal; Armadillo-type fold; Vitellinogen, open beta-sheet; Lipid transport, [illegible]

Q5TZ29	apobb.1	Uncharacterized protein	_VVELIK(cr)K_	2203	1	KKVVELIKKFNIEET		endoplasmic reticulum	response to chemical; response to stimulus;		lipid transporter activity; substrate-specific transporter activity; transporter activity;				IPR011030; IPR015817; IPR015818; IPR001747; IPR015819; IPR015816; IPR016024; IPR015255; IPR009454;	Vitellinogen, superhelical; Vitellinogen, open beta-sheet, subdomain 1; Vitellinogen, open beta-sheet, subdomain 2; Lipid transport protein, N-terminal; Lipid transport protein, beta-sheet shell; Vitellinogen, beta-sheet N-terminal; Armadillo-type fold; Vitellinogen, open beta-sheet; Lipid transport, open beta-sheet;
A8KB78	rps23	Uncharacterized protein	_WHDK(cr)QYK_	25	1	REQKWHDKQYKKAHL	......DK.......	cytosol	cellular protein metabolic process; biosynthetic process; primary metabolic process; cellular metabolic process; organic substance biosynthetic process; cellular biosynthetic process; organic substance metabolic process; metabolic process; cellular macromolecule biosynthetic process; cellular macromolecule metabolic process; gene expression; macromolecule metabolic process; protein metabolic process; macromolecule biosynthetic process; translation; cellular process;	intracellular organelle; ribosome; intracellular non-membrane-bounded organelle; small ribosomal subunit; cytoplasm; intracellular organelle part; macromolecular complex; intracellular part; cell; organelle part; non-membrane-bounded organelle; organelle; ribosomal subunit; cell part; intracellular; ribonucleoprotein complex; cytoplasmic part;	structural constituent of ribosome; structural molecule activity;	K02973	RP-S23e; small subunit ribosomal protein S23e	dre03010 Ribosome - Danio rerio (zebrafish);	IPR012340;	Nucleic acid-binding, OB-fold;
B8A568	myhz1.1	Uncharacterized protein	_YEEGQAELEGAQK(cr)EAR_	1474	1	AELEGAQKEARSLST		nuclear		intracellular organelle; intracellular non-membrane-bounded organelle; cytoskeletal part; intracellular organelle part; macromolecular complex; intracellular part; cell; actin cytoskeleton; organelle part; non-membrane-bounded organelle; organelle; cytoskeleton; cell part; intracellular; myosin complex; protein complex;	ATP binding; motor activity; nucleoside phosphate binding; nucleoside binding; ion binding; hydrolase activity, acting on acid anhydrides; hydrolase activity; nucleotide binding; nucleoside-triphosphatase activity; organic cyclic compound binding; heterocyclic compound binding; ribonucleoside binding; carbohydrate derivative binding; pyrophosphatase activity; catalytic activity; ribonucleotide binding; binding; purine ribonucleoside binding; anion binding; purine ribonucleotide binding; adenyl ribonucleotide binding; adenyl nucleotide binding; purine nucleoside binding; hydrolase activity, acting on acid anhydrides, in phosphorus-containing anhydrides; purine nucleotide binding; small molecule binding; purine ribonucleoside triphosphate binding;	K10352	MYH; myosin heavy chain	dre04530 Tight junction - Danio rerio (zebrafish);	IPR002928; IPR004009; IPR001609; IPR027417; IPR027401;	Myosin tail; Myosin, N-terminal, SH3-like; Myosin head, motor domain; P-loop containing nucleoside triphosphate hydrolase; Myosin-like IQ motif-containing domain;
B8A561	myhz1.2	Uncharacterized protein	_YEEGQAELEGAQK(cr)EAR_	1474	1	AELEGAQKEARSLST		nuclear		intracellular organelle; intracellular non-membrane-bounded organelle; cytoskeletal part; intracellular organelle part; macromolecular complex; intracellular part; cell; actin cytoskeleton; organelle part; non-membrane-bounded organelle; organelle; cytoskeleton; cell part; intracellular; myosin complex; protein complex;	ATP binding; motor activity; nucleoside phosphate binding; nucleoside binding; ion binding; hydrolase activity, acting on acid anhydrides; hydrolase activity; nucleotide binding; nucleoside-triphosphatase activity; organic cyclic compound binding; heterocyclic compound binding; ribonucleoside binding; carbohydrate derivative binding; pyrophosphatase activity; catalytic activity; ribonucleotide binding; binding; purine ribonucleoside binding; anion binding; purine ribonucleotide binding; adenyl ribonucleotide binding; adenyl nucleotide binding; purine nucleoside binding; hydrolase activity, acting on acid anhydrides, in phosphorus-containing anhydrides; purine nucleotide binding; small molecule binding; purine ribonucleoside triphosphate binding;	K10352	MYH; myosin heavy chain	dre04530 Tight junction - Danio rerio (zebrafish);	IPR002928; IPR004009; IPR001609; IPR027417; IPR027401;	Myosin tail; Myosin, N-terminal, SH3-like; Myosin head, motor domain; P-loop containing nucleoside triphosphate hydrolase; Myosin-like IQ motif-containing domain;
E7EYX7	cirbpb	Uncharacterized protein	_YGTIAK(cr)VDVIR_	33	1	SKYGTIAKVDVIRDR		nuclear			organic cyclic compound binding; heterocyclic compound binding; nucleoside phosphate binding; binding; nucleic acid binding; nucleotide binding; small molecule binding;	K13195			IPR000504; IPR012677;	RNA recognition motif domain; Nucleotide-binding, alpha-[illegible]
Q8AX99	actn3b	Actn3b	_YLDIPK(cr)MLDAEDIVNTPKPDEK_	231	1	EKYLDIPKMLDAEDI	.......K.L.....	cytosol	cellular component biogenesis; cellular component organization; actin crosslink formation; protein complex subunit organization; single-organism cellular process; actin filament organization; single-organism process; actin filament bundle assembly; actin filament bundle organization; macromolecular complex subunit organization; single-organism organelle organization; actin cytoskeleton organization; cellular component assembly; organelle organization; actin filament-based process; cytoskeleton organization; cellular process; cellular component organization or biogenesis;		cation binding; metal ion binding; calcium ion binding; binding; ion binding;	K05699	ACTN; actinin alpha	dre04510 Focal adhesion - Danio rerio (zebrafish); dre04530 Tight junction - Danio rerio (zebrafish); dre04810 Regulation of actin cytoskeleton - Danio rerio (zebrafish); dre04520 Adherens junction - Danio rerio (zebrafish);	IPR014837; IPR002048; IPR011992; IPR001715;	EF-hand, Ca insensitive; EF-hand domain; EF-hand domain pair; Calponin homology domain;
Q802C9	eif4a1a	Eukaryotic translation initiation factor 4A, isoform 1A	_YLSPK(cr)YIK_	174	1	NRRYLSPKYIKMFAL	.......K.I.....	cytosol,nucle ar			nucleoside phosphate binding; ATP-dependent helicase activity; translation initiation factor activity; hydrolase activity, acting on acid anhydrides; hydrolase activity; ATPase activity; organic cyclic compound binding; heterocyclic compound binding; pyrophosphatase activity; catalytic activity; ribonucleotide binding; binding; purine ribonucleoside binding; anion binding; adenyl ribonucleotide binding; adenyl nucleotide binding; hydrolase activity, acting on acid anhydrides, in phosphorus-containing anhydrides; purine nucleotide binding; purine ribonucleoside triphosphate binding; RNA binding; ATP binding; purine NTP-dependent helicase activity; helicase activity; ion binding; nucleoside binding; nucleotide binding; nucleoside-triphosphatase activity; ribonucleoside binding; translation factor activity, nucleic acid binding; carbohydrate derivative binding; nucleic acid binding; ATPase activity, coupled; purine ribonucleotide binding; purine nucleoside binding; small molecule binding;	K03257	EIF4A; translation initiation factor 4A	dre03013 RNA transport - Danio rerio (zebrafish);	IPR014014; IPR011545; IPR027417; IPR014001; IPR001650;	RNA helicase, DEAD-box type, Q motif; DEAD/DEAH box helicase domain; P-loop containing nucleoside triphosphate hydrolase; Helicase, superfamily 1/2, ATP-binding domain; Helicase, C-terminal;
Q6XG62	icn	Protein S100 (Fragment)	_YSGK(cr)EGDK_	23	1	TFHKYSGKEGDKLTL		cytosol			cation binding; metal ion binding; calcium ion binding; binding; ion binding;				IPR011992; IPR002048; IPR013787;	EF-hand domain pair; EF-hand domain; S100/CaBP-9k-type, calcium binding, subdomain;

F1QUR3	-	Protein disulfide-isomerase (Fragment)	_YVMK(cr)EEFSR_	294	1	KGDKYVMKEEFSRDG	.......K.E.....	cytosol	regulation of cellular process; regulation of biological quality; small molecule metabolic process; cell redox homeostasis; single-organism cellular process; ether metabolic process; cellular homeostasis; single-organism process; organic substance metabolic process; homeostatic process; metabolic process; regulation of biological process; single-organism metabolic process; glycerol ether metabolic process; cellular process; biological regulation;	organelle; intracellular membrane-bounded organelle; intracellular organelle; endoplasmic reticulum; cell part; intracellular; cytoplasm; endomembrane system; intracellular part; membrane-bounded organelle; cell; cytoplasmic part;	protein disulfide oxidoreductase activity; oxidoreductase activity, acting on a sulfur group of donors; disulfide oxidoreductase activity; electron carrier activity; isomerase activity; catalytic activity; oxidoreductase activity;	K08056	PDIA3; protein disulfide isomerase family A, member 3 [EC:5.3.4.1]	dre04141 Protein processing in endoplasmic reticulum - Danio rerio (zebrafish);	IPR005788; IPR013766; IPR012336;	Disulphide isomerase; Thioredoxin domain; Thioredoxin-like fold;
E7FC21	nt5c2a	Uncharacterized protein	_YVVK(cr)DAK_	228	1	NLEKYVVKDAKLPLL	..E....K.......	cytosol			metal ion binding; cation binding; binding; ion binding;	K01081	E3.1.3.5; 5'-nucleotidase [EC:3.1.3.5]	dre00240 Pyrimidine metabolism - Danio rerio (zebrafish); dre01100 Metabolic pathways - Danio rerio (zebrafish); dre00760 Nicotinate and nicotinamide metabolism - Danio rerio (zebrafish); dre00230 Purine metabolism - Danio rerio (zebrafish);	IPR023214;	HAD-like domain;

Zebrafish							Human for proteins						Human for sequence				
Uniprot ID	Gene names	Protein names	Modified sequence	Position	# modificiation	Surrounding sequence	Uniprot ID	Gene names	Protein names	Identities (%)	Blast Score	E-value	Sequence identity	Position	Amino acid	Blast Score	E-value
B8A568	myhz1.1	Uncharacterized protein	_GK(cr)QAFTQQIEELK(cr)R_	1307	2	VSQLTRGKQAFTQQI	P12882	MYH1	Myosin-1	82.1	3208	0E+00	VSQLSRGKQAFTQQI	1309	K	46.9	1E-08
B8A568	myhz1.1	Uncharacterized protein	_GK(cr)QAFTQQIEELK(cr)R_	1318	2	TQQIEELKRQIEEEV	P12882	MYH1	Myosin-1	82.1	3208	0E+00	TQQIEELKRQLEEEI	1320	K	46.4	2E-08
B8A568	myhz1.1	Uncharacterized protein	_K(cr)LAEK(cr)DEEIEQIK_	1577	2	VKSEIDRKLAEKDEE	P12882	MYH1	Myosin-1	82.1	3208	0E+00	VKSEVDRKIAEKDEE	1579	K	44.3	9E-08
B8A568	myhz1.1	Uncharacterized protein	_K(cr)LAEK(cr)DEEIEQIK_	1581	2	IDRKLAEKDEEIEQI	P12882	MYH1	Myosin-1	82.1	3208	0E+00	VDRKIAEKDEEIDQM	1583	K	40.1	3E-06
B8A568	myhz1.1	Uncharacterized protein	_LQDLVDK(cr)LQLK(cr)VK_	1872	2	RLQDLVDKLQLKVKA	P12882	MYH1	Myosin-1	82.1	3208	0E+00	RLQDLVDKLQAKVKS	1874	K	41.4	1E-06
B8A568	myhz1.1	Uncharacterized protein	_LQDLVDK(cr)LQLK(cr)VK_	1876	2	LVDKLQLKVKAYKRQ	P12882	MYH1	Myosin-1	82.1	3208	0E+00	LVDKLQAKVKSYKRQ	1878	K	42.2	5E-07
B8A561	myhz1.2	Uncharacterized protein	_LAEK(cr)DEEIEQIK(cr)R_	1581	2	IDRKLAEKDEEIEQI	P12882	MYH1	Myosin-1	82.1	3207	0E+00	VDRKIAEKDEEIDQM	1583	K	40.1	3E-06
B8A561	myhz1.2	Uncharacterized protein	_LAEK(cr)DEEIEQIK(cr)R_	1589	2	DEEIEQIKRNSQRIT	P12882	MYH1	Myosin-1	82.1	3207	0E+00	DEEIDQMKRNHIRIV	1591	K	31.2	4E-03
B8A561	myhz1.2	Uncharacterized protein	_K(cr)LAEK(cr)DEEIEQIK_	1577	2	VKSEIDRKLAEKDEE	P12882	MYH1	Myosin-1	82.1	3207	0E+00	VKSEVDRKIAEKDEE	1579	K	44.3	9E-08
B8A561	myhz1.2	Uncharacterized protein	_K(cr)LAEK(cr)DEEIEQIK_	1581	2	IDRKLAEKDEEIEQI	P12882	MYH1	Myosin-1	82.1	3207	0E+00	VDRKIAEKDEEIDQM	1583	K	40.1	3E-06
B8A561	myhz1.2	Uncharacterized protein	_GK(cr)QAFTQQIEELK(cr)R_	1307	2	VSQLTRGKQAFTQQI	P12882	MYH1	Myosin-1	82.1	3207	0E+00	VSQLSRGKQAFTQQI	1309	K	46.9	1E-08
B8A561	myhz1.2	Uncharacterized protein	_GK(cr)QAFTQQIEELK(cr)R_	1318	2	TQQIEELKRQIEEEV	P12882	MYH1	Myosin-1	82.1	3207	0E+00	TQQIEELKRQLEEEI	1320	K	46.4	2E-08
Q6NWK4	atp6v1e1b	Atp6v1e1 protein	_AEEEFNIEK(cr)GR_	42	1	EEEFNIEKGRLVQTQ	P36543	ATP6V1E1	V-type proton ATPase subunit E 1	78.3	367	1E-131	EEEFNIEKGRLVQTQ	42	K	52	3E-09
Q1MTC4	vtg2	Uncharacterized protein	_LVPEFAHDK(cr)TYVYK_	28	1	VPEFAHDKTYVYKYE	P04275	VWF	von Willebrand factor	25.7	51.2	1E-06	-	-	-	-	-
B0R198	vdac3	Uncharacterized protein	_SAK(cr)DIFSK_	40	1	ADLGKSAKDIFSKGY	P45880	VDAC2	Voltage-dependent anion-selective channel protein 2	71.9	402	1E-143	ADLGKAARDIFNKGF	26	R	37.1	3E-05
Q8AWD0	vdac2	Uncharacterized protein	_SAK(cr)DIFNK_	15	1	ADLGKSAKDIFNKGY	P45880	VDAC2	Voltage-dependent anion-selective channel protein 2	83.0	504	0E+00	ADLGKAARDIFNKGF	26	R	40.5	2E-06
B0R198	vdac3	Uncharacterized protein	_VK(cr)ELGLSLNQK_	90	1	LETKYKVKELGLSLN	P45880	VDAC2	Voltage-dependent anion-selective channel protein 2	71.9	402	1E-143	-	-	-	-	-
Q6IQK3	vamp3	Uncharacterized protein	_VNVDK(cr)VLER_	38	1	IMRVNVDKVLERDQK	Q15836	VAMP3	Vesicle-associated membrane protein 3	90.8	143	5E-47	IMRVNVDKVLERDQK	35	K	52.8	9E-11
B0R193	ubb	Uncharacterized protein	_TITLEVEPSDTIENVK(cr)AK_	27	1	SDTIENVKAKIQDKE	P62979	RPS27A	Ubiquitin-40S ribosomal protein S27a	97.5	159	6E-53	SDTIENVKAKIQDKE	27	K	50.3	7E-10
B0R193	ubb	Uncharacterized protein	_LIFAGK(cr)QLEDGR_	48	1	QRLIFAGKQLEDGRT	P62979	RPS27A	Ubiquitin-40S ribosomal protein S27a	97.5	159	6E-53	QRLIFAGKQLEDGRT	48	K	50.3	7E-10
Q6YI49	uchl1	Ubiquitin carboxyl-terminal hydrolase	_AK(cr)ELEQNK_	126	1	MSPAERAKELEQNKA	P09936	UCHL1	Ubiquitin carboxyl-terminal hydrolase isozyme L1	66.1	305	4E-107	-	-	-	-	-
Q6PE34	zgc:65894	Uncharacterized protein	_MSMK(cr)EVDEQMLNVQNK_	324	1	FRGRMSMKEVDEQML	P68371	TUBB4B	Tubulin beta-4B chain	97.1	914	0E+00	FRGRMSMKEVDEQML	324	K	55.4	1E-11
Q6NWJ5	tuba8l4	Tubulin alpha 6	_GDVVPK(cr)DVNSAIATIK_	326	1	YRGDVVPKDVNSAIA	Q71U36	TUBA1A	Tubulin alpha-1A chain	96.9	920	0E+00	YRGDVVPKDVNAAIA	326	K	47.3	8E-09
E7EXP0	tnnt3b	Uncharacterized protein	_IPDGDK(cr)VDFDDIQK_	32	1	PKIPDGDKVDFDDIQ	P45378	TNNT3	Troponin T, fast skeletal muscle	72.5	279	5E-96	PKIPEGEKVDFDDIQ	64	K	47.3	8E-09
E7EXP0	tnnt3b	Uncharacterized protein	_IPDGDKVDFDDIQK(cr)K_	40	1	VDFDDIQKKRQNKDL	P45378	TNNT3	Troponin T, fast skeletal muscle	72.5	279	5E-96	VDFDDIQKKRQNKDL	72	K	52.4	1E-10
Q9I8U9	tnnt3a	Fast skeletal muscle troponin T	_VDFDDIQK(cr)K_	38	1	VDFDDIQKKRHNKDT	P45378	TNNT3	Troponin T, fast skeletal muscle	70.6	269	3E-92	VDFDDIQKKRQNKDL	72	K	46.4	2E-08
E7EXP0	tnnt3b	Uncharacterized protein	_EEEELIALK(cr)DR_	72	1	EEELIALKDRIEKRR	P45378	TNNT3	Troponin T, fast skeletal muscle	72.5	279	5E-96	EEELVALKERIEKRR	104	K	46.4	2E-08
E7EXP0	tnnt3b	Uncharacterized protein	_KKEEEELIALK(cr)DR_	72	1	EEELIALKDRIEKRR	P45378	TNNT3	Troponin T, fast skeletal muscle	72.5	279	5E-96	EEELVALKERIEKRR	104	K	46.4	2E-08

Q9I8U9	tnnt3a	Fast skeletal muscle troponin T	_QLNIDHLNEDK(cr)LR_	173	1	IDHLNEDKLRDKAQE	P45378	TNNT3	Troponin T, fast skeletal muscle	70.6	269	3E-92	IDHLGEDKLRDKAKE	206	K	42.6	4E-07
E7EXP0	tnnt3b	Uncharacterized protein	_VEELSK(cr)YSK_	220	1	KRVEELSKYSKKGAA	P45378	TNNT3	Troponin T, fast skeletal muscle	72.5	279	5E-96	-	-	-	-	-
Q9I8U9	tnnt3a	Fast skeletal muscle troponin T	_TLESEK(cr)FEHMER_	192	1	IKTLESEKFEHMERL	P45378	TNNT3	Troponin T, fast skeletal muscle	70.6	269	3E-92	-	-	-	-	-
Q6IQ92	tnni1al	Troponin 1	_DLHQK(cr)IDVVDEER_	73	1	LCRDLHQKIDVVDEE	P19237	TNNI1	Troponin I, slow skeletal muscle	60.1	198	3E-66	LCRELHAKVEVVDEE	72	K	40.5	2E-06
Q6DHP2	tnni2b.2	Troponin I, skeletal, fast 2b.2	_ELHQK(cr)IDVVDEER_	67	1	LCKELHQKIDVVDEE	P48788	TNNI2	Troponin I, fast skeletal muscle	60.9	217	1E-73	LCKQLHAKIDAAEEE	71	K	34.6	3E-04
Q0D2W2	tnni2a.4	Troponin I, skeletal, fast 2b.2	_ANLK(cr)QVK_	138	1	MDLRANLKQVKKEVK	P48788	TNNI2	Troponin I, fast skeletal muscle	60.8	217	8E-74	MDLRANLKQVKKEDT	142	K	45.2	7E-07
Q0D2W2	tnni2a.4	Uncharacterized protein	_NIEDK(cr)AGMDGR_	162	1	WRKNIEDKAGMDGRK	P48788	TNNI2	Troponin I, fast skeletal muscle	60.8	217	8E-74	WRKNIEEKSGMEGRK	168	K	46	2E-08
F1QCC0	tnni2b.1	Uncharacterized protein	_SDKEIEDLK(cr)IK_	93	1	DKEIEDLKIKVQDLK	P48788	TNNI2	Troponin I, fast skeletal muscle	60.9	220	7E-75	-	-	-	-	-
F1QCC0	tnni2b.1	Uncharacterized protein	_SDK(cr)EIEDLK_	87	1	TKVAKSDKEIEDLKI	P48788	TNNI2	Troponin I, fast skeletal muscle	60.9	220	7E-75	-	-	-	-	-
Q0D2W2	tnni2a.4	Fast muscle troponin I	_ADK(cr)EIEDLK_	87	1	AKVAKADKEIEDLKI	P48788	TNNI2	Troponin I, fast skeletal muscle	60.8	217	8E-74	-	-	-	-	-
Q0D2W2	tnni2a.4	Fast muscle troponin I	_ADKEIEDLK(cr)IK_	93	1	DKEIEDLKIKVIDLK	P48788	TNNI2	Troponin I, fast skeletal muscle	60.8	217	8E-74	-	-	-	-	-
Q0D2W2	tnni2a.4	Fast muscle troponin I	_LHQQIDK(cr)VDEER_	70	1	KLHQQIDKVDEERYD	P19429	TNNI3	Troponin I, cardiac muscle	61.0	169	9E-54	QLHARVDKVDEERYD	108	K	38.8	8E-06
Q6IQD7	tpm2	Uncharacterized protein	_AEVAEAK(cr)SGDLEEELK_	189	1	RAEVAEAKSGDLEEE	P07951	TPM2	Tropomyosin beta chain	89.0	444	4E-159	RAEVAESKCGDLEEE	189	K	42.2	5E-07
Q6P0W3	tpm3	Uncharacterized protein	_EDKYEEEIK(cr)ILTDK_	226	1	DKYEEEIKILTDKLK	P06753	TPM3	Tropomyosin alpha-3 chain	94.7	479	6E-174	DKYEEEIKILTDKLK	227	K	52	2E-10
P13104	tpma	Tropomyosin alpha-1 chain	_AEQAETDK(cr)K_	29	1	AEQAETDKKAAEERS	P09493	TPM1	Tropomyosin alpha-1 chain	92.3	492	5E-179	AEQAEADKKAAEDRS	29	K	42.6	5E-06
P13104	tpma	Tropomyosin alpha-1 chain	_SK(cr)QLEDDLVALQK_	37	1	KAAEERSKQLEDDLV	P09493	TPM1	Tropomyosin alpha-1 chain	92.3	492	5E-179	KAAEDRSKQLEDELV	37	K	44.3	9E-08
P13104	tpma	Tropomyosin alpha-1 chain	_QLEDDLVALQK(cr)K_	48	1	DDLVALQKKLKATED	P09493	TPM1	Tropomyosin alpha-1 chain	92.3	492	5E-179	DELVSLQKKLKGTED	48	K	40.5	2E-06
P13104	tpma	Tropomyosin alpha-1 chain	_LK(cr)ATEDELDK_	51	1	VALQKKLKATEDELD	P09493	TPM1	Tropomyosin alpha-1 chain	92.3	492	5E-179	VSLQKKLKGTEDELD	51	K	43.1	3E-07
P13104	tpma	Tropomyosin alpha-1 chain	_LK(cr)ATEDELDKYSEALK_	51	1	VALQKKLKATEDELD	P09493	TPM1	Tropomyosin alpha-1 chain	92.3	492	5E-179	VSLQKKLKGTEDELD	51	K	43.1	3E-07
P13104	tpma	Tropomyosin alpha-1 chain	_ATEDELDK(cr)YSEALK_	59	1	ATEDELDKYSEALKD	P09493	TPM1	Tropomyosin alpha-1 chain	92.3	492	5E-179	GTEDELDKYSEALKD	61	K	47.7	9E-08
P13104	tpma	Tropomyosin alpha-1 chain	_ATEDELDKYSEALK(cr)DAQEK_	65	1	DKYSEALKDAQEKLE	P09493	TPM1	Tropomyosin alpha-1 chain	92.3	492	5E-179	DKYSEALKDAQEKLE	65	K	50.3	1E-08
P13104	tpma	Tropomyosin alpha-1 chain	_DAQEK(cr)LELAEK_	70	1	ALKDAQEKLELAEKK	P09493	TPM1	Tropomyosin alpha-1 chain	92.3	492	5E-179	ALKDAQEKLELAEKK	70	K	48.6	3E-09
P13104	tpma	Uncharacterized protein	_LATALQK(cr)LEEAEK_	112	1	RLATALQKLEEAEKA	P09493	TPM1	Tropomyosin alpha-1 chain	92.3	492	5E-179	RLATALQKLEEAEKA	112	K	48.1	4E-09
P13104	tpma	Tropomyosin alpha-1 chain	_LEEAEK(cr)AADESER_	118	1	QKLEEAEKAADESER	P09493	TPM1	Tropomyosin alpha-1 chain	92.3	492	5E-179	QKLEEAEKAADESER	118	K	49.4	1E-09
P13104	tpma	Uncharacterized protein	_ALK(cr)DEEK_	136	1	VIENRALKDEEKMEL	P09493	TPM1	Tropomyosin alpha-1 chain	92.3	492	5E-179	VIESRAQKDEEKMEI	136	K	40.9	2E-05
P13104	tpma	Uncharacterized protein	_EAK(cr)HIAEEADR_	152	1	EIQLKEAKHIAEEAD	P09493	TPM1	Tropomyosin alpha-1 chain	92.3	492	5E-179	EIQLKEAKHIAEDAD	152	K	48.1	4E-09
P13104	tpma	Uncharacterized protein	_K(cr)YEEVAR_	161	1	IAEEADRKYEEVARK	P09493	TPM1	Tropomyosin alpha-1 chain	92.3	492	5E-179	IAEDADRKYEEVARK	161	K	48.6	3E-09
P13104	tpma	Uncharacterized protein	_K(cr)LVIVEGELER_	168	1	KYEEVARKLVIVEGE	P09493	TPM1	Tropomyosin alpha-1 chain	92.3	492	5E-179	KYEEVARKLVIIESD	168	K	42.6	4E-07
Q6IQD7	tpm2	Uncharacterized protein	_K(cr)LVILEGELER_	168	1	KYEEVARKLVILEGE	P09493	TPM1	Tropomyosin alpha-1 chain	85.6	452	2E-163	KYEEVARKLVIIESD	168	K	41.4	1E-06
P13104	tpma	Tropomyosin alpha-1 chain	_TVTNNMK(cr)SLEAQAEK_	205	1	KTVTNNMKSLEAQAE	P09493	TPM1	Tropomyosin alpha-1 chain	92.3	492	5E-179	KTVTNNLKSLEAQAE	205	K	46	2E-08
P13104	tpma	Uncharacterized protein	_EDKYEEEIK(cr)VLTDK_	226	1	DKYEEEIKVLTDKLK	P09493	TPM1	Tropomyosin alpha-1 chain	92.3	492	5E-179	DRYEEEIKVLSDKLK	226	K	45.6	3E-08
P13104	tpma	Tropomyosin alpha-1 chain	_VLTDK(cr)LK_	231	1	EIKVLTDKLKEAETR	P09493	TPM1	Tropomyosin alpha-1 chain	92.3	492	5E-179	EIKVLSDKLKEAETR	231	K	46.9	1E-08
E7FBZ3	zgc:171719	Uncharacterized protein	_LEK(cr)TIDDLEEK_	251	1	RSVAKLEKTIDDLEE	P09493	TPM1	Tropomyosin alpha-1 chain	85.8	406	4E-145	RSVTKLEKSIDDLED	251	K	41.4	1E-06

P13104	tpma	Tropomyosin alpha-1 chain	_LEK(cr)TIDDLEDELYAQK_	251	1	RSVAKLEKTIDDLED	P09493	TPM1	Tropomyosin alpha-1 chain	92.3	492	5E-179	RSVTKLEKSIDDLED	251	K	43.9	1E-07
P13104	tpma	Tropomyosin alpha-1 chain	_TIDDLEDELYAQK(cr)LK_	264	1	EDELYAQKLKYKAIS	P09493	TPM1	Tropomyosin alpha-1 chain	92.3	492	5E-179	EDELYAQKLKYKAIS	264	K	51.1	4E-10
E9QBF0	tpi1b	Triosephosphate isomerase	_TASPQQAQEVHDK(cr)LR_	194	1	QAQEVHDKLRQW___	P60174	TPI1	Triosephosphate isomerase	82.9	327	2E-115	QAQEVHEKLRGW___	225	K	35.4	7E-05
B8JHR2	tpt1	Translationally-controlled tumor protein homolog	_LQETSYDK(cr)K_	72	1	LQETSYDKKSYTAYI	P13693	TPT1	Translationally-controlled tumor protein	67.5	233	1E-80	-	-	-	-	-
Q29RA2	zgc:136908	Uncharacterized protein	_NAPAIIFIDELDAIAPK(cr)R_	314	1	ELDAIAPKREKTHGE	P55072	VCP	Transitional endoplasmic reticulum ATPase	85.7	1382	0E+00	ELDAIAPKREKTHGE	312	K	50.3	7E-10
Q29RA2	zgc:136908	Uncharacterized protein	_LEILQIHTK(cr)NMK_	388	1	EILQIHTKNMKLSED	P55072	VCP	Transitional endoplasmic reticulum ATPase	85.7	1382	0E+00	EILQIHTKNMKLADD	386	K	47.7	6E-09
G1K2X0	ttnb	Uncharacterized protein (Fragment)	_AYHVEK(cr)R_	22276	1	VKAYHVEKREASKKA	Q8WZ42	TTN	Titin	63.4	29927	0E+00	VKNYHIEKREASKKA	27243	K	43.1	2E-07
G1K2X0	ttnb	Uncharacterized protein (Fragment)	_VHHYVVEK(cr)R_	24446	1	VHHYVVEKREASRRT	Q8WZ42	TTN	Titin	63.4	29927	0E+00	IHHYVVEKREASRRS	29414	K	46.9	1E-08
G1K2X0	ttnb	Uncharacterized protein (Fragment)	_VHEK(cr)NLR_	25335	1	EWTKVHEKNLRVTEH	Q8WZ42	TTN	Titin	63.4	29927	0E+00	-	-	-	-	-
G1K2X0	ttnb	Uncharacterized protein (Fragment)	_VK(cr)IIDVSK_	11960	1	PDPPTRVKIIDVSKG	Q8WZ42	TTN	Titin	63.4	29927	0E+00	-	-	-	-	-
G1K2X0	ttnb	Uncharacterized protein (Fragment)	_GVTISK(cr)VK_	27496	1	QRGVTISKVKIAPFE	Q8WZ42	TTN	Titin	63.4	29927	0E+00	-	-	-	-	-
B0S758	ttna	Uncharacterized protein	_EK(cr)ELESGRPEGEIVGR_	7294	1	IEFLKKEKELESGRP	Q8WZ42	TTN	Titin	57.5	3667	0E+00	-	-	-	-	-
Q7ZUI4	zgc:56493	Thioredoxin	_LEEMVK(cr)QHK_	104	1	TKLEEMVKQHKN___	P10599	TXN	Thioredoxin	60.0	137	2E-44	-	-	-	-	-
Q7ZYX4	cct6a	Chaperonin containing TCP1, subunit 6A (Zeta 1)	_DK(cr)ALAVLEEVK_	129	1	GFEAAKDKALAVLEE	P40227	CCT6A	T-complex protein 1 subunit zeta	87.6	970	0E+00	GFEAAKEKALQFLEE	129	K	35	2E-04
E9QD64	cct6a	Uncharacterized protein	_VMK(cr)IIDLK_	228	1	FIEDRVMKIIDLKNK	P40227	CCT6A	T-complex protein 1 subunit zeta	72.8	410	7E-144	FIEDRVKKIIELKRK	273	K	39.7	4E-06
Q7ZYX4	cct6a	Chaperonin containing TCP1, subunit 6A (Zeta 1)	_GIDPFSLDALAK(cr)EGIVALR_	307	1	FSLDALAKEGIVALR	P40227	CCT6A	T-complex protein 1 subunit zeta	87.6	970	0E+00	FSLDALSKEGIVALR	307	K	45.6	3E-08
E9QD64	cct6a	Uncharacterized protein	_DK(cr)ALAVLEEVK_	84	1	IAKAAKDKALAVLEE	P40227	CCT6A	T-complex protein 1 subunit zeta	72.8	410	7E-144	-	-	-	-	-
Q6PH46	cct4	T-complex protein 1 subunit delta	_MK(cr)IMVIK_	315	1	LHFLNKMKIMVIKDI	P50991	CCT4	T-complex protein 1 subunit delta	88.8	946	0E+00	LHFLNKMKIMVIKDI	321	K	54.9	2E-11
F1Q9K1	smarcc1a	Uncharacterized protein	_HQGTITEDK(cr)SK_	196	1	QGTITEDKSKATHII	Q8TAQ2	SMARCC2	SWI/SNF complex subunit SMARCC2	67.0	1237	0E+00	QGTVTEDKNNASHVV	171	K	33.3	7E-04
F1REG7	oxct1a	Uncharacterized protein (Fragment)	_AVFDVDK(cr)DK_	75	1	KAVFDVDKDKGLTLI	P55809	OXCT1	Succinyl-CoA:3-ketoacid coenzyme A transferase 1, mitochondrial	83.3	173	9E-54	KAVFDVDKKKGLTLI	480	K	44.3	1E-06
F1QFC0	hspa9	Uncharacterized protein	_NAK(cr)VLENAEGAR_	79	1	VMDGKNAKVLENAEG	P38646	HSPA9	Stress-70 protein, mitochondrial	89.4	1216	0E+00	VMEGKQAKVLENAEG	76	K	42.6	4E-07
F1QFC0	hspa9	Uncharacterized protein	_RFDDAEVQK(cr)DLK_	138	1	FDDAEVQKDLKNVPY	P38646	HSPA9	Stress-70 protein, mitochondrial	89.4	1216	0E+00	YDDPEVQKDIKNVPF	135	K	39.2	6E-06
F1R446	spna2	Uncharacterized protein	_K(cr)HEDFDK_	1489	1	SVEALIKKHEDFDKA	Q13813	SPTAN1	Spectrin alpha chain, non-erythrocytic 1	90.0	4571	0E+00	SVEALIKKHEDFDKA	1486	K	50.3	7E-10
F1Q560	shank3b	Uncharacterized protein	_GANK(cr)EIK_	354	1	LLFRGANKEIKNYNN	Q9BYB0	SHANK3	SH3 and multiple ankyrin repeat domains protein 3	53.9	1303	0E+00	LLFRGANRDVRNYNS	309	R	37.5	2E-05
E9QIC5	srsf1a	Serine/arginine-rich-splicing factor 1A	_TK(cr)DVEDVFYK_	29	1	LPPDIRTKDVEDVFY	Q07955	SRSF1	Serine/arginine-rich splicing factor 1	96.5	170	3E-55	LPPDIRTKDIEDVFY	30	K	51.1	4E-10
Q642Z0	atp2a1	Calcium-transporting ATPase	_NAESAIEALK(cr)EYEPEMGK_	120	1	ESAIEALKEYEPEMG	O14983	ATP2A1	Sarcoplasmic/endoplasmic reticulum calcium ATPase 1	85.9	1736	0E+00	ENAIEALKEYEPEMG	120	K	49.4	1E-09
Q5U3A4	atp2a1	Calcium-transporting ATPase	_EYEPEM(ox)GK(cr)VYR_	128	1	EYEPEMGKVYRSDRK	O14983	ATP2A1	Sarcoplasmic/endoplasmic reticulum calcium ATPase 1	85.6	1734	0E+00	EYEPEMGKVYRADRK	128	K	51.5	2E-10
Q642Z0	atp2a1	Calcium-transporting ATPase	_EYEPEMGK(cr)VYR_	128	1	EYEPEMGKVYRSDRK	O14983	ATP2A1	Sarcoplasmic/endoplasmic reticulum calcium ATPase 1	85.9	1736	0E+00	EYEPEMGKVYRADRK	128	K	51.5	2E-10

Q642Z0	atp2a1	Calcium-transporting ATPase	_EIVPGDIVEVSVGDK(cr)VPADIR_	158	1	VEVSVGDKVPADIRI	O14983	ATP2A1	Sarcoplasmic/endoplasmic reticulum calcium ATPase 1	85.9	1736	0E+00	VEVAVGDKVPADIRI	158	K	46.9	1E-08
Q5U3A4	atp2a1	Calcium-transporting ATPase	_AVNQDK(cr)K_	204	1	PRAVNQDKKNMLFSG	O14983	ATP2A1	Sarcoplasmic/endoplasmic reticulum calcium ATPase 1	85.6	1734	0E+00	PRAVNQDKKNMLFSG	204	K	51.5	4E-09
Q642Z0	atp2a1	Calcium-transporting ATPase	_AVNQDK(cr)K_	204	1	PRAVNQDKKNMLFSG	O14983	ATP2A1	Sarcoplasmic/endoplasmic reticulum calcium ATPase 1	85.9	1736	0E+00	PRAVNQDKKNMLFSG	204	K	51.5	4E-09
Q5U3A4	atp2a1	Calcium-transporting ATPase	_TPLQQK(cr)LDEFGEQLSK_	252	1	EKTPLQQKLDEFGEQ	O14983	ATP2A1	Sarcoplasmic/endoplasmic reticulum calcium ATPase 1	85.6	1734	0E+00	DKTPLQQKLDEFGEQ	252	K	49	2E-09
Q5U3A4	atp2a1	Calcium-transporting ATPase	_APVGNK(cr)MFVK_	511	1	SKAPVGNKMFVKGAP	O14983	ATP2A1	Sarcoplasmic/endoplasmic reticulum calcium ATPase 1	85.6	1734	0E+00	SRAAVGNKMFVKGAP	511	K	42.2	8E-06
Q642Z0	atp2a1	Calcium-transporting ATPase	_APVGNK(cr)M(ox)FVK_	511	1	SKAPVGNKMFVKGAP	O14983	ATP2A1	Sarcoplasmic/endoplasmic reticulum calcium ATPase 1	85.9	1736	0E+00	SRAAVGNKMFVKGAP	511	K	42.2	8E-06
Q642Z0	atp2a1	Calcium-transporting ATPase	_APVGNK(cr)MFVK_	511	1	SKAPVGNKMFVKGAP	O14983	ATP2A1	Sarcoplasmic/endoplasmic reticulum calcium ATPase 1	85.9	1736	0E+00	SRAAVGNKMFVKGAP	511	K	42.2	8E-06
Q5U3A4	atp2a1	Calcium-transporting ATPase	_DK(cr)IMAVIK_	544	1	LTGPVKDKIMAVIKE	O14983	ATP2A1	Sarcoplasmic/endoplasmic reticulum calcium ATPase 1	85.6	1734	0E+00	LTGPVKEKIMAVIKE	544	K	48.1	4E-09
Q642Z0	atp2a1	Calcium-transporting ATPase	_DK(cr)IMAVIK_	544	1	LTGPVKDKIMAVIKE	O14983	ATP2A1	Sarcoplasmic/endoplasmic reticulum calcium ATPase 1	85.9	1736	0E+00	LTGPVKEKIMAVIKE	544	K	48.1	4E-09
Q6NXC1	rbm4.3	Uncharacterized protein	_LHVANVEK(cr)GTDDELR_	87	1	LHVANVEKGTDDELR	Q9BWF3	RBM4	RNA-binding protein 4	39.7	241	3E-78	-	-	-	-	-
Q6P027	rlbp1b	Cellular retinaldehyde-binding protein b	_AKDELNETDEK(cr)R_	58	1	ELNETDEKRTSAVKE	P12271	RLBP1	Retinaldehyde-binding protein 1	71.1	462	2E-166	-	-	-	-	-
O42248	gnb2l1	Guanine nucleotide-binding protein subunit beta-2-like 1	_DK(cr)TIIMWK_	38	1	ILSASRDKTIIMWKL	P63244	RACK1	Receptor of activated protein C kinase 1	95.6	636	0E+00	ILSASRDKTIIMWKL	38	K	52.8	9E-11
F1RBK3	-	Pyruvate kinase	_IISK(cr)LENHEGVR_	269	1	KDIRIISKLENHEGV	P14618	PKM	Pyruvate kinase PKM	77.0	865	0E+00	KNIKIISKIENHEGV	270	K	41.8	7E-07
F1RBK3	-	Pyruvate kinase	_GSGTEEVK(cr)LVK_	132	1	GSGTEEVKLVKGNII	P14618	PKM	Pyruvate kinase PKM	77.0	865	0E+00	-	-	-	-	-
Q90ZM2	sec61al1	Protein transport protein Sec61 subunit alpha-like 1	_IIEVGDTPK(cr)DR_	107	1	IEVGDTPKDRALFNG	P61619	SEC61A1	Protein transport protein Sec61 subunit alpha isoform 1	97.1	949	0E+00	IEVGDTPKDRALFNG	107	K	50.3	7E-10
Q90ZM2	sec61al1	Protein transport protein Sec61 subunit alpha-like 1	_DVAK(cr)QLK_	392	1	SSAKDVAKQLKEQQM	P61619	SEC61A1	Protein transport protein Sec61 subunit alpha isoform 1	97.1	949	0E+00	SSAKDVAKQLKEQQM	392	K	49.8	1E-09
Q6XG62	icn	Protein S100 (Fragment)	_YSGK(cr)EGDK_	23	1	TFHKYSGKEGDKLTL	P26447	S100A4	Protein S100-A4	57.5	115	7E-36	TFHKYSGKEGDKFKL	22	K	41.8	7E-07
Q6XG62	icn	Protein S100 (Fragment)	_LTLSK(cr)GELK_	32	1	GDKLTLSKGELKELL	P26447	S100A4	Protein S100-A4	57.5	115	7E-36	GDKFKLNKSELKELL	31	K	33.3	7E-04
Q6XG62	icn	Protein S100	_TTDK(cr)AALDK_	53	1	IFGKTTDKAALDKIF	P26447	S100A4	Protein S100-A4	57.5	115	7E-36	-	-	-	-	-
Q6XG62	icn	Protein S100	_AALDK(cr)IFK_	58	1	TDKAALDKIFKDLDA	P26447	S100A4	Protein S100-A4	57.5	115	7E-36	-	-	-	-	-
F1R8T2	-	Protein S100 (Fragment)	_TTDK(cr)ASLDNIFK_	53	1	VFGKTTDKASLDNIF	P26447	S100A4	Protein S100-A4	55.0	110	4E-33	-	-	-	-	-
Q1ECX9	pdia4	Protein disulfide-isomerase A4	_SK(cr)VLEVAK_	434	1	ATQFWRSKVLEVAKD	P13667	PDIA4	Protein disulfide-isomerase A4	76.5	952	0E+00	ATQFWRSKVLEVAKD	437	K	51.5	2E-10
F1QUR3	-	Protein disulfide-isomerase (Fragment)	_YVMK(cr)EEFSR_	294	1	KGDKYVMKEEFSRDG	P30101	PDIA3	Protein disulfide-isomerase A3	79.0	751	0E+00	KGEKFVMQEEFSRDG	339	Q	42.2	5E-07
F1QUR3	-	Protein disulfide-isomerase (Fragment)	_FAHTNNEDLLK(cr)K_	149	1	TNNEDLLKKHGIDGE	P30101	PDIA3	Protein disulfide-isomerase A3	79.0	751	0E+00	-	-	-	-	-
B0S564	-	Protein disulfide-isomerase	_TLDGFTK(cr)FLESGGK_	466	1	RTLDGFTKFLESGGK	P07237	P4HB	Protein disulfide-isomerase	76.0	741	0E+00	RTLDGFKKFLESGGQ	468	K	41.8	7E-07
B0S564	-	Protein disulfide-isomerase	_DFQDK(cr)MDQFK_	269	1	AAKDFQDKMDQFKKA	P07237	P4HB	Protein disulfide-isomerase	76.0	741	0E+00	-	-	-	-	-
B0S564	-	Protein disulfide-isomerase	_DVESEDSK(cr)AFIK_	168	1	DVESEDSKAFIKTAE	P07237	P4HB	Protein disulfide-isomerase	76.0	741	0E+00	-	-	-	-	-
B0S564	-	Protein disulfide-isomerase	_GNPK(cr)EYSAGR_	112	1	GGEKGNPKEYSAGRQ	P07237	P4HB	Protein disulfide-isomerase	76.0	741	0E+00	-	-	-	-	-

Q6TGV6	psma5	Proteasome subunit alpha type	_EELEDVIK(cr)DI_	239	1	EELEDVIKDI_____	P28066	PSMA5	Proteasome subunit alpha type-5	96.3	485	9E-178	EELEEVIKDI_____	239	K	33.3	3E-04
Q7ZV77	psma4	Proteasome subunit alpha type	_TMDVSK(cr)LSAEK_	205	1	NKTMDVSKLSAEKVE	P25789	PSMA4	Proteasome subunit alpha type-4	96.2	525	0E+00	NKTMDVSKLSAEKVE	205	K	49.8	1E-09
Q7ZV77	psma4	Proteasome subunit alpha type	_QK(cr)EVEELIK_	231	1	KIKVLKQKEVEELIK	P25789	PSMA4	Proteasome subunit alpha type-4	96.2	525	0E+00	VIRVLKQKEVEQLIK	233	K	41.4	1E-06
E9QIC9	lmnb1	Uncharacterized protein	_LK(cr)DVEALLNAK_	137	1	TSSQNRLKDVEALLN	P02545	LMNA	Prelamin-A/C [Cleaved into: Lamin-A/C	58.0	171	1E-50	IAAQARLKDLEALLN	141	K	32	2E-03
I3ITD9	pabpc1b	Uncharacterized protein	_NLDK(cr)SIDNK_	108	1	IFIKNLDKSIDNKAL	P11940	PABPC1	Polyadenylate-binding protein 1	92.2	271	7E-90	IFIKNLDKSIDNKAL	108	K	50.7	5E-10
F1QXV8	pgk1	Phosphoglycerate kinase	_LHLDK(cr)VDVK_	11	1	SNKLHLDKVDVKGKR	P00558	PGK1	Phosphoglycerate kinase 1	88.7	759	0E+00	SNKLTLDKLDVKGKR	11	K	38.8	8E-06
E9QF19	pgk1	Phosphoglycerate kinase	_NLLGK(cr)DVQFLK_	100	1	ELKNLLGKDVQFLKD	P00558	PGK1	Phosphoglycerate kinase 1	87.8	357	9E-125	ELKSLLGKDVLFLKD	91	K	41.4	1E-06
F1QXV8	pgk1	Phosphoglycerate kinase	_ITLPVDFITADK(cr)FDEK_	291	1	VDFITADKFDEKATT	P00558	PGK1	Phosphoglycerate kinase 1	88.7	759	0E+00	VDFVTADKFDENAKT	291	K	40.5	2E-06
Q6PC53	ppiab	Peptidyl-prolyl cis-trans isomerase	_ADVVPK(cr)TAENFR_	31	1	LRADVVPKTAENFRA	P30405	PPIF	Peptidyl-prolyl cis-trans isomerase F, mitochondrial	75.9	262	2E-91	LKADVVPKTAENFRA	73	K	46.4	2E-07
Q6PC53	ppiab	Peptidyl-prolyl cis-trans isomerase	_FEDENFTLK(cr)HGGK_	91	1	EDENFTLKHGGKGTL	P30405	PPIF	Peptidyl-prolyl cis-trans isomerase F, mitochondrial	75.9	262	2E-91	PDENFTLKHVGPGVL	135	K	32	2E-03
B8JKN7	ppiaa	Uncharacterized protein	_HVVFGQVVEGLDVIK(cr)K_	80	1	VEGLDVIKKVEGFGS	P30405	PPIF	Peptidyl-prolyl cis-trans isomerase F, mitochondrial	78.8	179	8E-60	KEGMDVVKKIESFGS	183	K	35	2E-04
Q6PC53	ppiab	Peptidyl-prolyl cis-trans isomerase	_VVDGLDVVDAIEK(cr)K_	144	1	DVVDAIEKKGSSSGK	P30405	PPIF	Peptidyl-prolyl cis-trans isomerase F, mitochondrial	75.9	262	2E-91	-	-	-	-	-
F6NLI6	ppib	Peptidyl-prolyl cis-trans isomerase	_GDGTGGK(cr)SIYGDR_	108	1	RGDGTGGKSIYGDRF	P23284	PPIB	Peptidyl-prolyl cis-trans isomerase B	82.2	357	5E-128	RGDGTGGKSIYGERF	116	K	46.9	1E-08
F6NLI6	ppib	Peptidyl-prolyl cis-trans isomerase	_HVVFGK(cr)ILEGMDVVR_	163	1	GKHVVFGKILEGMDV	P23284	PPIB	Peptidyl-prolyl cis-trans isomerase B	82.2	357	5E-128	GKHVVFGKVLEGMEV	171	K	46	2E-08
Q6IMW7	pvalb4	Parvalbumin 4	_AFAIIDQDK(cr)SGFIEEEELK_	55	1	FAIIDQDKSGFIEEE	P20472	PVALB	Parvalbumin alpha	57.0	118	8E-37	FHMLDKDKSGFIEED	54	K	31.6	3E-03
Q9I8V0	pvalb2	Parvalbumin-2	_AFFVIDQDK(cr)SGFIEEDELK_	55	1	FFVIDQDKSGFIEED	P20472	PVALB	Parvalbumin alpha	55.1	114	2E-35	FHMLDKDKSGFIEED	55	K	34.6	4E-03
Q9I8V0	pvalb2	Parvalbumin-2	_AFLSAGDSDGDGK(cr)IGVDEFALLVK_	97	1	GDSDGDGKIGVDEFA	P20472	PVALB	Parvalbumin alpha	55.1	114	2E-35	GDKDGDGKIGVDEFS	97	K	41.8	1E-05
Q804W0	pvalb1	Parvalbumin 1	_AFLSAGDSDGDGK(cr)IGAEEFAALVK_	97	1	GDSDGDGKIGAEEFA	P20472	PVALB	Parvalbumin alpha	55.1	117	3E-36	GDKDGDGKIGVDEFS	97	K	35.4	2E-03
Q6IMW7	pvalb4	Parvalbumin 4	_ALTDGETK(cr)TFLK_	84	1	ALTDGETKTFLKAGD	P20472	PVALB	Parvalbumin alpha	57.0	118	8E-37	-	-	-	-	-
Q6IMW7	pvalb4	Parvalbumin 4	_TFLK(cr)AGDSDGDGK_	88	1	GETKTFLKAGDSDGD	P20472	PVALB	Parvalbumin alpha	57.0	118	8E-37	-	-	-	-	-
Q9I8V0	pvalb2	Parvalbumin-2	_VGLSAK(cr)SPDDIK_	39	1	AKVGLSAKSPDDIKK	P20472	PVALB	Parvalbumin alpha	55.1	114	2E-35	-	-	-	-	-
Q9I8V0	pvalb2	Parvalbumin-2	_ALTDAETK(cr)AFLSAGDSDGDGK_	84	1	ALTDAETKAFLSAGD	P20472	PVALB	Parvalbumin alpha	55.1	114	2E-35	-	-	-	-	-
Q9I8V0	pvalb2	Parvalbumin-2	_IGVDEFALLVK(cr)A_	108	1	DEFALLVKA______	P20472	PVALB	Parvalbumin alpha	55.1	114	2E-35	-	-	-	-	-
Q9I8V0	pvalb2	Parvalbumin-2	_K(cr)AFFVIDQDK_	46	1	KSPDDIKKAFFVIDQ	P20472	PVALB	Parvalbumin alpha	55.1	114	2E-35	-	-	-	-	-
Q9I8V0	pvalb2	Parvalbumin-2	_NFFAK(cr)VGLSAK_	33	1	NYKNFFAKVGLSAKS	P20472	PVALB	Parvalbumin alpha	55.1	114	2E-35	-	-	-	-	-
Q804W0	pvalb1	Parvalbumin 4	_SADEVK(cr)K_	45	1	AKSADEVKKAFAIID	P20472	PVALB	Parvalbumin alpha	55.1	117	3E-36	-	-	-	-	-
Q804W0	pvalb1	Parvalbumin 1	_SFFAK(cr)VGLSAK_	33	1	DYKSFFAKVGLSAKS	P20472	PVALB	Parvalbumin alpha	55.1	117	3E-36	-	-	-	-	-
Q804W0	pvalb1	Parvalbumin 1	_IGAEEFAALVK(cr)A_	108	1	EEFAALVKA______	P20472	PVALB	Parvalbumin alpha	55.1	117	3E-36	-	-	-	-	-
Q804W0	pvalb1	Parvalbumin 1	_K(cr)AFAIIDQDNSGFIEEEELK_	46	1	KSADEVKKAFAIIDQ	P20472	PVALB	Parvalbumin alpha	55.1	117	3E-36	-	-	-	-	-
Q804W0	pvalb1	Parvalbumin 1	_VLTDK(cr)ETK_	81	1	NARVLTDKETKAFLS	P20472	PVALB	Parvalbumin alpha	55.1	117	3E-36	-	-	-	-	-
Q804W0	pvalb1	Parvalbumin 1	_ETK(cr)AFLSAGDSDGDGK_	84	1	VLTDKETKAFLSAGD	P20472	PVALB	Parvalbumin alpha	55.1	117	3E-36	-	-	-	-	-
Q803X7	nap1l1	Nap1l1 protein	_NVTLK(cr)TIK_	263	1	KGKNVTLKTIKKKQK	P55209	NAP1L1	Nucleosome assembly protein 1-like 1	83.0	541	0E+00	KGKNVTLKTIKKKQK	271	K	48.6	3E-09
F1RB19	ybx1	Uncharacterized protein	_VIATK(cr)VLGTVK_	38	1	DKKVIATKVLGTVKW	P67809	YBX1	Nuclease-sensitive element-binding protein 1	53.7	98.6	4E-25	DKKVIATKVLGTVKW	58	K	50.3	7E-10
A2BGU3	nono	Uncharacterized protein	_ASEIFINK(cr)DR_	105	1	ASEIFINKDRGFGFI	Q15233	NONO	Non-POU domain-containing octamer-binding protein	66.5	355	1E-122	AGEVFIHKDKGFGFI	107	K	38.8	1E-04
F1R1J9	ahnak	Uncharacterized protein	_IPK(cr)TDIDIK_	1275	1	KVKGSKFKIPFSGPT	Q09666	AHNAK	Neuroblast differentiation-associated protein AHNAK	46.4	4398	0E+00	KVKGSKFKMPFLSIS	3404	K	33.7	5E-04

B0UY61	neb	Uncharacterized protein	_AAK(cr)EAYK_	5231	1	AHEIRAAKEAYKNIS	P20929	NEB	Nebulin	55.2	6620	0E+00	-	-	-	-	-
E9QGC9	myoz1b	Uncharacterized protein	_TPK(cr)DVMLEELSLMK_	51	1	GTKIKTPKDVMLEEL	Q9NP98	MYOZ1	Myozenin-1	44.0	240	6E-24	GKKISVPRDVMLEEL	42	R	35	2E-04
F1QJK4	myh9b	Uncharacterized protein	_AEMEDLVSSK(cr)DDVGK_	1512	1	MEDLVSSKDDVGKSV	P35579	MYH9	Myosin-9	83.0	2808	0E+00	MEDLMSSKDDVGKSV	1513	K	46	3E-07
B8A561	myhz1.2	Uncharacterized protein	_AVETEK(cr)AEIQTALEEAEGTLEHEESK_	1539	1	KKAVETEKAEIQTAL	A7E2Y1	MYH7B	Myosin-7B	67.0	2492	0E+00	KKALEGEKSEIQAAL	1544	K	33.3	7E-04
F1QIR4	-	Uncharacterized protein	_LK(cr)EAYAK_	863	1	KEEFTKLKEAYAKSE	P12883	MYH7	Myosin-7	85.0	3349	0E+00	KEEFTRLKEALEKSE	860	K	36.7	4E-05
B8A561	myhz1.2	Uncharacterized protein	_SSVK(cr)LLATLYPPVVEETGGGK_	619	1	LYQKSSVKLLATLYP	P13533	MYH6	Myosin-6	77.0	3033	0E+00	LYQKSSLKLMATLFS	616	K	36.7	4E-05
B8A568	myhz1.1	Uncharacterized protein	_SSVK(cr)LLATLYPPVVEETGGGK_	619	1	LYQKSSVKLLATLYP	P13533	MYH6	Myosin-6	77.0	3031	0E+00	LYQKSSLKLMATLFS	616	K	36.7	4E-05
F1QIR4	-	Uncharacterized protein	_TINDFTMQK(cr)AK_	1280	1	INDFTMQKAKLQTEN	P13533	MYH6	Myosin-6	85.0	3315	0E+00	LNDFTTQRAKLQTEN	1280	R	40.1	3E-06
F1QIR4	-	Uncharacterized protein	_AQLEFNQVK(cr)ADIER_	1572	1	QLEFNQVKADIERKL	P13533	MYH6	Myosin-6	85.0	3315	0E+00	QLEFNQIKAEIERKL	1571	K	46.9	1E-08
F1QIR4	-	Uncharacterized protein	_K(cr)LSEKDEEMEQAK_	1578	1	VKADIERKLSEKDEE	P13533	MYH6	Myosin-6	85.0	8594	0E+00	IKAEIERKLAEKDEE	1577	K	43.1	2E-07
F1QIR4	-	Uncharacterized protein	_KLSEK(cr)DEEM(ox)EQAK_	1582	1	IERKLSEKDEEMEQA	P13533	MYH6	Myosin-6	85.0	3315	0E+00	IERKLAEKDEEMEQA	1581	K	49.8	1E-09
F1QIR4	-	Uncharacterized protein	_KLSEK(cr)DEEMEQAK_	1582	1	IERKLSEKDEEMEQA	P13533	MYH6	Myosin-6	85.0	3315	0E+00	IERKLAEKDEEMEQA	1581	K	49.8	1E-09
Q6IQX1	myhz2	Myosin, heavy polypeptide 2, fast muscle specific	_LAEKDEEMEQIK(cr)R_	1589	1	DEEMEQIKRNSQRVT	P13533	MYH6	Myosin-6	77.0	3039	0E+00	DEEMEQAKRNHQRVV	1589	K	39.7	4E-06
B8A561	myhz1.2	Uncharacterized protein	_QSEEAEEQANSHLSK(cr)LR_	1897	1	QANSHLSKLRKVQHE	P13533	MYH6	Myosin-6	77.0	3033	0E+00	QANTNLSKFRKVQHE	1897	K	39.7	4E-06
B8A568	myhz1.1	Uncharacterized protein	_QSEEAEEQANSHLSK(cr)LR_	1897	1	QANSHLSKLRKVQHE	P13533	MYH6	Myosin-6	77.0	3031	0E+00	QANTNLSKFRKVQHE	1897	K	39.7	4E-06
E7FAD0	myhz1.1	Uncharacterized protein	_ELEEK(cr)MVSLLQEK_	878	1	KKKELEEKMVSLLQE	Q9Y623	MYH4	Myosin-4	83.4	3249	0E+00	KRKELEEKMVTLMQE	880	K	43.1	2E-07
B8A561	myhz1.2	Uncharacterized protein	_ANALAANLDK(cr)K_	1446	1	ALAANLDKKQRNFDK	P11055	MYH3	Myosin-3	80.0	3081	0E+00	SLAAALDKKQRNFDK	1447	K	42.2	5E-07
B8A568	myhz1.1	Uncharacterized protein	_ANALAANLDK(cr)K_	1446	1	ALAANLDKKQRNFDK	P11055	MYH3	Myosin-3	80.0	3081	0E+00	SLAAALDKKQRNFDK	1447	K	42.2	5E-07
B8A561	myhz1.2	Uncharacterized protein	_QAAEAQK(cr)QLR_	1643	1	RQAAEAQKQLRNVQA	P11055	MYH3	Myosin-3	80.0	3081	0E+00	RQAAETLKHLRSVQG	1642	K	32	2E-03
B8A568	myhz1.1	Uncharacterized protein	_QAAEAQK(cr)QLR_	1643	1	RQAAEAQKQLRNVQA	P11055	MYH3	Myosin-3	80.0	3081	0E+00	RQAAETLKHLRSVQG	1642	K	32	2E-03
F1QIR4	-	Uncharacterized protein	_IK(cr)ELTYQTEEDRK_	1851	1	RKYERRIKELTYQTE	P11055	MYH3	Myosin-3	78.0	2991	0E+00	RKYERRVKELTYQSE	1849	K	48.1	4E-09
B8A561	myhz1.2	Uncharacterized protein	_DFETSQLLSK(cr)IEDEQSLGAQLQK_	1095	1	ETSQLLSKIEDEQSL	Q9UKX2	MYH2	Myosin-2	82.0	3197	0E+00	EISNLQSKIEDEQAL	1099	K	33.3	7E-04
B8A568	myhz1.1	Uncharacterized protein	_DFETSQLLSK(cr)IEDEQSLGAQLQK_	1095	1	ETSQLLSKIEDEQSL	Q9UKX2	MYH2	Myosin-2	82.0	3197	0E+00	EISNLQSKIEDEQAL	1099	K	33.3	7E-04
X1WF87	myhb	Uncharacterized protein	_TEELEEAK(cr)K_	1392	1	TEELEEAKKKLAQRL	Q9UKX2	MYH2	Myosin-2	82.4	3242	0E+00	TEELEEAKKKLAQRL	1396	K	49.4	1E-09
F1QIR4	-	Uncharacterized protein	_TEELEDAK(cr)K_	1393	1	TEELEDAKKKLAQRL	Q9UKX2	MYH2	Myosin-2	79.0	3103	0E+00	TEELEEAKKKLAQRL	1396	K	46.9	1E-08
F1QIR4	-	Uncharacterized protein	_NSYEEVLDQLETMK(cr)R_	1502	1	LDQLETMKRENKNLQ	Q9UKX2	MYH2	Myosin-2	79.0	3103	0E+00	LDQLETLKRENKNLQ	1505	K	48.6	3E-09
F1QIR4	-	Uncharacterized protein	_K(cr)QLEQEK_	1534	1	HELEKIRKQLEQEKA	Q9UKX2	MYH2	Myosin-2	79.0	3103	0E+00	HELEKIKKQVEQEKC	1537	K	41.8	7E-07
Q6IQX1	myhz2	Myosin, heavy polypeptide 2, fast muscle specific	_KK(cr)DFETAQLLSK_	1085	1	SEEKIKKKDFETAQL	Q9UKX3	MYH13	Myosin-13	79.0	3106	0E+00	IEEKLKKKEFELSQL	1089	K	32.9	1E-03
Q6IQX1	myhz2	Myosin, heavy polypeptide 2, fast muscle specific	_EDDVHPM(ox)NPPK(cr)FDK_	85	1	VHPMNPPKFDKIEDM	P12882	MYH1	Myosin-1	82.1	3210	0E+00	VFPMNPPKYDKIEDM	84	K	46.9	1E-08
B8A561	myhz1.2	Uncharacterized protein	_M(ox)QGSLEDQIIAANPLLEAYGNAK(cr)TVR_	238	1	LEAYGNAKTVRNDNS	P12882	MYH1	Myosin-1	82.1	3207	0E+00	LEAFGNAKTVRNDNS	237	K	46.4	2E-08
B8A568	myhz1.1	Uncharacterized protein	_GQTVPQVYNSVSALSK(cr)SIYER_	433	1	NSVSALSKSIYERMF	P12882	MYH1	Myosin-1	82.1	3208	0E+00	NAVGALAKAVYDKMF	432	K	31.6	3E-03

B8A561	myhz1.2	Uncharacterized protein	_ENLGK(cr)LM(ox)TNLR_	659	1	QFRENLGKLMTNLRS	P12882	MYH1	Myosin-1	82.1	3207	0E+00	LFRENLNKLMTNLRS	663	K	44.8	6E-08
B8A561	myhz1.2	Uncharacterized protein	_ENLGK(cr)LMTNLR_	659	1	QFRENLGKLMTNLRS	P12882	MYH1	Myosin-1	82.1	3207	0E+00	LFRENLNKLMTNLRS	663	K	44.8	6E-08
B8A568	myhz1.1	Uncharacterized protein	_ENLGK(cr)LMTNLR_	659	1	QFRENLGKLMTNLRS	P12882	MYH1	Myosin-1	82.1	3208	0E+00	LFRENLNKLMTNLRS	663	K	44.8	6E-08
B8A561	myhz1.2	Uncharacterized protein	_VLNASVIPEGQFIDNK(cr)K_	741	1	EGQFIDNKKASEKLL	P12882	MYH1	Myosin-1	82.1	3207	0E+00	EGQFIDSKKASEKLL	743	K	46.4	2E-08
B8A561	myhz1.2	Uncharacterized protein	_FGHTK(cr)VFFK_	764	1	EYRFGHTKVFFKAGL	P12882	MYH1	Myosin-1	82.1	3207	0E+00	QYKFGHTKVFFKAGL	766	K	45.6	3E-08
B8A568	myhz1.1	Uncharacterized protein	_FGHTK(cr)VFFK_	764	1	EYRFGHTKVFFKAGL	P12882	MYH1	Myosin-1	82.1	3208	0E+00	QYKFGHTKVFFKAGL	766	K	45.6	3E-08
B8A561	myhz1.2	Uncharacterized protein	_SAETEK(cr)ELATM(ox)K_	849	1	LKSAETEKELATMKE	P12882	MYH1	Myosin-1	82.1	3207	0E+00	LKSAETEKEMANMKE	851	K	43.5	2E-07
B8A561	myhz1.2	Uncharacterized protein	_SAETEK(cr)ELATMK_	849	1	LKSAETEKELATMKE	P12882	MYH1	Myosin-1	82.1	3207	0E+00	LKSAETEKEMANMKE	851	K	43.5	2E-07
B8A568	myhz1.1	Uncharacterized protein	_SAETEK(cr)ELATMK_	849	1	LKSAETEKELATMKE	P12882	MYH1	Myosin-1	82.1	3208	0E+00	LKSAETEKEMANMKE	851	K	43.5	2E-07
B8A561	myhz1.2	Uncharacterized protein	_ELATMK(cr)EDFVK_	855	1	EKELATMKEDFVKCK	P12882	MYH1	Myosin-1	82.1	3207	0E+00	EKEMANMKEEFEKTK	857	K	31.2	4E-03
B8A568	myhz1.1	Uncharacterized protein	_ELATMK(cr)EDFVK_	855	1	EKELATMKEDFVKCK	P12882	MYH1	Myosin-1	82.1	3208	0E+00	EKEMANMKEEFEKTK	857	K	31.2	4E-03
B8A568	myhz1.1	Uncharacterized protein	_EDFVK(cr)CK_	860	1	TMKEDFVKCKEDLAK	P12882	MYH1	Myosin-1	82.1	3208	0E+00	NMKEEFEKTKEELAK	864	K	31.6	3E-03
B8A569	myhz1.3	Uncharacterized protein	_EALAK(cr)AEAK_	867	1	KCKEALAKAEAKKKE	P12882	MYH1	Myosin-1	82.1	3211	0E+00	KTKEELAKTEAKRKE	873	K	32	2E-03
B8A561	myhz1.2	Uncharacterized protein	_EDLAK(cr)AEAK_	867	1	KCKEDLAKAEAKKKE	P12882	MYH1	Myosin-1	82.1	3207	0E+00	KTKEELAKTEAKRKE	873	K	33.7	5E-04
B8A568	myhz1.1	Uncharacterized protein	_EDLAK(cr)AEAK_	867	1	KCKEDLAKAEAKKKE	P12882	MYH1	Myosin-1	82.1	3208	0E+00	KTKEELAKTEAKRKE	873	K	33.7	5E-04
B8A561	myhz1.2	Uncharacterized protein	_KK(cr)ELEEK_	873	1	AKAEAKKKELEEKMV	P12882	MYH1	Myosin-1	82.1	3207	0E+00	AKTEAKRKELEEKMV	875	K	43.9	1E-07
B8A568	myhz1.1	Uncharacterized protein	_KK(cr)ELEEK_	873	1	AKAEAKKKELEEKMV	P12882	MYH1	Myosin-1	82.1	3208	0E+00	AKTEAKRKELEEKMV	875	K	43.9	1E-07
B8A561	myhz1.2	Uncharacterized protein	_ELEEK(cr)MVALLQEK_	878	1	KKKELEEKMVALLQE	P12882	MYH1	Myosin-1	82.1	3207	0E+00	KRKELEEKMVTLMQE	880	K	42.6	3E-07
B8A568	myhz1.1	Uncharacterized protein	_ELEEK(cr)MVALLQEK_	878	1	KKKELEEKMVALLQE	P12882	MYH1	Myosin-1	82.1	3208	0E+00	KRKELEEKMVTLMQE	880	K	42.6	3E-07
B8A568	myhz1.1	Uncharacterized protein	_LK(cr)ETTERLEDEEEINAELTAK_	922	1	IQLEAKLKETTERLE	P12882	MYH1	Myosin-1	82.1	3208	0E+00	IQLEAKIKEVTERAE	924	K	37.1	3E-05
B8A561	myhz1.2	Uncharacterized protein	_LEDEEEINAELTAK(cr)K_	941	1	INAELTAKKRKLEDE	P12882	MYH1	Myosin-1	82.1	3207	0E+00	INAELTAKKRKLEDE	943	K	49.8	1E-09
B8A568	myhz1.1	Uncharacterized protein	_LEDEEEINAELTAK(cr)K_	941	1	INAELTAKKRKLEDE	P12882	MYH1	Myosin-1	82.1	3208	0E+00	INAELTAKKRKLEDE	943	K	49.8	1E-09
B8A561	myhz1.2	Uncharacterized protein	_HATENK(cr)VK_	976	1	EKHATENKVKNLTEE	P12882	MYH1	Myosin-1	82.1	3207	0E+00	EKHATENKVKNLTEE	978	K	50.7	5E-10
B8A568	myhz1.1	Uncharacterized protein	_HATENK(cr)VK_	976	1	EKHATENKVKNLTEE	P12882	MYH1	Myosin-1	82.1	3208	0E+00	EKHATENKVKNLTEE	978	K	50.7	5E-10
Q6IQX1	myhz2	Uncharacterized protein	_VK(cr)NLTEEM(ox)ASQDESIAK_	978	1	HATENKVKNLTEEMA	P12882	MYH1	Myosin-1	82.1	3210	0E+00	HATENKVKNLTEEMA	980	K	51.5	2E-10
Q6IQX1	myhz2	Uncharacterized protein (Fragment)	_VK(cr)NLTEEMASQDESIAK_	978	1	HATENKVKNLTEEMA	P12882	MYH1	Myosin-1	82.1	3210	0E+00	HATENKVKNLTEEMA	980	K	51.5	2E-10
B8A561	myhz1.2	Uncharacterized protein	_VK(cr)NLTEEMAAQDESIGK_	978	1	HATENKVKNLTEEMA	P12882	MYH1	Myosin-1	82.1	3207	0E+00	HATENKVKNLTEEMA	980	K	51.5	2E-10
B8A568	myhz1.1	Uncharacterized protein	_VK(cr)NLTEEMAAQDESIGK_	978	1	HATENKVKNLTEEMA	P12882	MYH1	Myosin-1	82.1	3208	0E+00	HATENKVKNLTEEMA	980	K	51.5	2E-10
A2BGX6	myhc4	Uncharacterized protein	_VK(cr)NLTEEMASQDESIAK_	978	1	HATENKVKNLTEEMA	P12882	MYH1	Myosin-1	82.1	3208	0E+00	HATENKVKNLTEEMA	980	K	51.5	2E-10
Q6IQX1	myhz2	Uncharacterized protein (Fragment)	_NLTEEMASQDESIAK(cr)LTK_	993	1	SQDESIAKLTKEKKA	P12882	MYH1	Myosin-1	82.1	3210	0E+00	GLDETIAKLTKEKKA	999	K	39.7	4E-06
B8A561	myhz1.2	Uncharacterized protein	_NLTEEMAAQDESIGK(cr)LTK_	993	1	AQDESIGKLTKEKKA	P12882	MYH1	Myosin-1	82.1	3207	0E+00	GLDETIAKLTKEKKA	999	K	36.3	6E-05
B8A568	myhz1.1	Uncharacterized protein	_NLTEEMAAQDESIGK(cr)LTK_	993	1	AQDESIGKLTKEKKA	P12882	MYH1	Myosin-1	82.1	3208	0E+00	GLDETIAKLTKEKKA	999	K	36.3	6E-05
A2BGX6	myhc4	Uncharacterized protein	_NLTEEM(ox)ASQDESIAK(cr)LTK_	993	1	SQDESIAKLTKEKKA	P12882	MYH1	Myosin-1	82.1	3208	0E+00	GLDETIAKLTKEKKA	999	K	39.7	4E-06

B8A561	myhz1.2	Uncharacterized protein	_ALQEAHQQTLDDLQAEEDK(cr)VNTLTK_	1018	1	DLQAEEDKVNTLTKS	P12882	MYH1	Myosin-1	82.1	3207	0E+00	DLQAEEDKVNTLTKA	1020	K	46.9	2E-07
B8A568	myhz1.1	Uncharacterized protein	_ALQEAHQQTLDDLQAEEDK(cr)VNTLTK_	1018	1	DLQAEEDKVNTLTKS	P12882	MYH1	Myosin-1	82.1	3208	0E+00	DLQAEEDKVNTLTKA	1020	K	46.9	2E-07
B8A561	myhz1.2	Uncharacterized protein	_ALQEAHQQTLDDLQAEEDKVNTLTK(cr)SK_	1024	1	DKVNTLTKSKTKLEQ	P12882	MYH1	Myosin-1	82.1	3207	0E+00	DKVNTLTKAKIKLEQ	1026	K	42.6	5E-06
B8A568	myhz1.1	Uncharacterized protein	_ALQEAHQQTLDDLQAEEDKVNTLTK(cr)SK_	1024	1	DKVNTLTKSKTKLEQ	P12882	MYH1	Myosin-1	82.1	3208	0E+00	DKVNTLTKAKIKLEQ	1026	K	42.6	5E-06
B8A561	myhz1.2	Uncharacterized protein	_LEQQVDDLEGSLEQEK(cr)K_	1044	1	EGSLEQEKKLRMDLE	P12882	MYH1	Myosin-1	82.1	3207	0E+00	EGSLEQEKKIRMDLE	1046	K	48.1	4E-09
B8A568	myhz1.1	Uncharacterized protein	_LEQQVDDLEGSLEQEK(cr)K_	1044	1	EGSLEQEKKLRMDLE	P12882	MYH1	Myosin-1	82.1	3208	0E+00	EGSLEQEKKIRMDLE	1046	K	48.1	4E-09
B8A561	myhz1.2	Uncharacterized protein	_LAQESIMDLENDK(cr)QQSEEK_	1075	1	IMDLENDKQQSEEKL	P12882	MYH1	Myosin-1	82.1	3207	0E+00	IMDLENDKQQIEEKL	1077	K	47.3	8E-09
B8A568	myhz1.1	Uncharacterized protein	_LAQESIMDLENDK(cr)QQSEEK_	1075	1	IMDLENDKQQSEEKL	P12882	MYH1	Myosin-1	82.1	3208	0E+00	IMDLENDKQQIEEKL	1077	K	47.3	8E-09
B8A561	myhz1.2	Uncharacterized protein	_K(cr)DFETSQLLSK_	1085	1	SEEKLKKKDFETSQL	P12882	MYH1	Myosin-1	82.1	3207	0E+00	LDEKLKKKEFEMSGL	1089	K	31.2	4E-03
B8A568	myhz1.1	Uncharacterized protein	_K(cr)DFETSQLLSK_	1085	1	SEEKLKKKDFETSQL	P12882	MYH1	Myosin-1	82.1	3208	0E+00	LDEKLKKKEFEMSGL	1089	K	31.2	4E-03
Q6IQX1	myhz2	Myosin, heavy polypeptide 2, fast muscle specific	_IEDEQSLGAQLQK(cr)K_	1108	1	SLGAQLQKKIKELQA	P12882	MYH1	Myosin-1	82.1	3210	0E+00	ALGMQLQKKIKELQA	1111	K	40.9	1E-06
B8A561	myhz1.2	Uncharacterized protein	_IEDEQSLGAQLQK(cr)K_	1108	1	SLGAQLQKKIKELQA	P12882	MYH1	Myosin-1	82.1	3207	0E+00	ALGMQLQKKIKELQA	1112	K	40.9	1E-06
B8A561	myhz1.2	Uncharacterized protein	_LEEAGGATAAQIEMNK(cr)K_	1167	1	AAQIEMNKKREAEFQ	P12882	MYH1	Myosin-1	82.1	3207	0E+00	SAQIEMNKKREAEFQ	1171	K	49.8	1E-09
B8A568	myhz1.1	Uncharacterized protein	_LEEAGGATAAQIEMNK(cr)K_	1167	1	AAQIEMNKKREAEFQ	P12882	MYH1	Myosin-1	82.1	3208	0E+00	SAQIEMNKKREAEFQ	1171	K	49.8	1E-09
B8A561	myhz1.2	Uncharacterized protein	_K(cr)QADSVAELGEQIDNLQR_	1197	1	TAAALRKKQADSVAE	P12882	MYH1	Myosin-1	82.1	3207	0E+00	TAATLRKKHADSVAE	1199	K	40.9	1E-06
B8A568	myhz1.1	Uncharacterized protein	_K(cr)QADSVAELGEQIDNLQR_	1197	1	TAAALRKKQADSVAE	P12882	MYH1	Myosin-1	82.1	3208	0E+00	TAATLRKKHADSVAE	1199	K	40.9	1E-06
B8A561	myhz1.2	Uncharacterized protein	_SEYK(cr)MEIDDLSSNMEAVAK_	1227	1	EKEKSEYKMEIDDLS	P12882	MYH1	Myosin-1	82.1	3207	0E+00	EKEKSEMKMEIDDLA	1229	K	41.4	1E-06
B8A568	myhz1.1	Uncharacterized protein	_SEYK(cr)MEIDDLSSNMEAVAK_	1227	1	EKEKSEYKMEIDDLS	P12882	MYH1	Myosin-1	82.1	3208	0E+00	EKEKSEMKMEIDDLA	1229	K	41.4	1E-06
B8A561	myhz1.2	Uncharacterized protein	_M(ox)EIDDLSSNM(ox)EAVAK(cr)AK_	1242	1	SNMEAVAKAKANLEK	P12882	MYH1	Myosin-1	82.1	3207	0E+00	SNMETVSKAKGNLEK	1244	K	40.1	3E-06
B8A561	myhz1.2	Uncharacterized protein	_MEIDDLSSNM(ox)EAVAK(cr)AK_	1242	1	SNMEAVAKAKANLEK	P12882	MYH1	Myosin-1	82.1	3207	0E+00	SNMETVSKAKGNLEK	1244	K	40.1	3E-06
B8A568	myhz1.1	Uncharacterized protein	_MEIDDLSSNM(ox)EAVAK(cr)AK_	1242	1	SNMEAVAKAKANLEK	P12882	MYH1	Myosin-1	82.1	3208	0E+00	SNMETVSKAKGNLEK	1244	K	40.1	3E-06
B8A561	myhz1.2	Uncharacterized protein	_AK(cr)ANLEK_	1244	1	MEAVAKAKANLEKMC	P12882	MYH1	Myosin-1	82.1	3207	0E+00	METVSKAKGNLEKMC	1246	K	43.1	4E-06
B8A568	myhz1.1	Uncharacterized protein	_AK(cr)ANLEK_	1244	1	MEAVAKAKANLEKMC	P12882	MYH1	Myosin-1	82.1	3208	0E+00	METVSKAKGNLEKMC	1246	K	43.1	4E-06
Q6IQX1	myhz2	Myosin, heavy polypeptide 2, fast muscle specific	_TVEDQLSEIK(cr)SK_	1262	1	EDQLSEIKSKNDENL	P12882	MYH1	Myosin-1	82.1	3210	0E+00	EDQLSEIKTKEEEQQ	1264	K	35	2E-04
B8A561	myhz1.2	Uncharacterized protein	_TLEDQLSEIK(cr)SK_	1262	1	EDQLSEIKSKNDENL	P12882	MYH1	Myosin-1	82.1	3207	0E+00	EDQLSEIKTKEEEQQ	1264	K	35	2E-04
B8A568	myhz1.1	Uncharacterized protein	_TLEDQLSEIK(cr)SK_	1262	1	EDQLSEIKSKNDENL	P12882	MYH1	Myosin-1	82.1	3208	0E+00	EDQLSEIKTKEEEQQ	1264	K	35	2E-04
B8A561	myhz1.2	Uncharacterized protein	_QLEEK(cr)EALVSQLTR_	1296	1	FGRQLEEKEALVSQL	P12882	MYH1	Myosin-1	82.1	3207	0E+00	YSRQLDEKDTLVSQL	1302	K	35.4	1E-04
B8A561	myhz1.2	Uncharacterized protein	_QAFTQQIEELK(cr)R_	1318	1	TQQIEELKRQIEEEV	P12882	MYH1	Myosin-1	82.1	3207	0E+00	TQQIEELKRQLEEEI	1320	K	46.4	2E-08
B8A568	myhz1.1	Uncharacterized protein	_QAFTQQIEELK(cr)R_	1318	1	TQQIEELKRQIEEEV	P12882	MYH1	Myosin-1	82.1	3208	0E+00	TQQIEELKRQLEEEI	1320	K	46.4	2E-08
B8A561	myhz1.2	Uncharacterized protein	_TEELEESK(cr)K_	1392	1	TEELEESKKKLAQRL	P12882	MYH1	Myosin-1	82.1	3207	0E+00	TEELEEAKKKLAQRL	1394	K	46.9	1E-08
B8A568	myhz1.1	Uncharacterized protein	_TEELEESK(cr)K_	1392	1	TEELEESKKKLAQRL	P12882	MYH1	Myosin-1	82.1	3208	0E+00	TEELEEAKKKLAQRL	1394	K	46.9	1E-08
B8A561	myhz1.2	Uncharacterized protein	_CASLEK(cr)TK_	1418	1	SKCASLEKTKQRLQG	P12882	MYH1	Myosin-1	82.1	3207	0E+00	AKCASLEKTKQRLQN	1422	K	43.9	1E-07

B8A568	myhz1.1	Uncharacterized protein	_CASLEK(cr)TK_	1418	1	SKCASLEKTKQRLQG	P12882	MYH1	Myosin-1	82.1	3208	0E+00	AKCASLEKTKQRLQN	1422	K	43.9	1E-07
B8A561	myhz1.2	Uncharacterized protein	_NFDK(cr)VLAEWK_	1453	1	KKQRNFDKVLAEWKQ	P12882	MYH1	Myosin-1	82.1	3207	0E+00	KKQRNFDKILAEWKQ	1455	K	51.5	3E-10
B8A568	myhz1.1	Uncharacterized protein	_NFDK(cr)VLAEWK_	1453	1	KKQRNFDKVLAEWKQ	P12882	MYH1	Myosin-1	82.1	3208	0E+00	KKQRNFDKILAEWKQ	1455	K	51.5	3E-10
B8A561	myhz1.2	Uncharacterized protein	_QK(cr)YEEGQAELEGAQK_	1461	1	VLAEWKQKYEEGQAE	P12882	MYH1	Myosin-1	82.1	3207	0E+00	ILAEWKQKCEETHAE	1463	K	36.7	4E-05
B8A568	myhz1.1	Uncharacterized protein	_QK(cr)YEEGQAELEGAQK_	1461	1	VLAEWKQKYEEGQAE	P12882	MYH1	Myosin-1	82.1	3208	0E+00	ILAEWKQKCEETHAE	1463	K	36.7	4E-05
B8A561	myhz1.2	Uncharacterized protein	_YEEGQAELEGAQK(cr)EAR_	1474	1	AELEGAQKEARSLST	P12882	MYH1	Myosin-1	82.1	3207	0E+00	AELEASQKESRSLST	1476	K	38.8	8E-06
B8A568	myhz1.1	Uncharacterized protein	_YEEGQAELEGAQK(cr)EAR_	1474	1	AELEGAQKEARSLST	P12882	MYH1	Myosin-1	82.1	3208	0E+00	AELEASQKESRSLST	1476	K	38.8	8E-06
B8A561	myhz1.2	Uncharacterized protein	_M(ox)K(cr)NSYEETLDQLETLK_	1487	1	STELFKMKNSYEETL	P12882	MYH1	Myosin-1	82.1	3207	0E+00	STELFKIKNAYEESL	1489	K	41.8	7E-07
B8A561	myhz1.2	Uncharacterized protein	_MK(cr)NSYEETLDQLETLK_	1487	1	STELFKMKNSYEETL	P12882	MYH1	Myosin-1	82.1	3207	0E+00	STELFKIKNAYEESL	1489	K	41.8	7E-07
B8A568	myhz1.1	Uncharacterized protein	_MK(cr)NSYEETLDQLETLK_	1487	1	STELFKMKNSYEETL	P12882	MYH1	Myosin-1	82.1	3208	0E+00	STELFKIKNAYEESL	1489	K	41.8	7E-07
B8A561	myhz1.2	Uncharacterized protein	_NSYEETLDQLETLK(cr)R_	1501	1	LDQLETLKRENKNLQ	P12882	MYH1	Myosin-1	82.1	3207	0E+00	LDQLETLKRENKNLQ	1503	K	51.1	3E-10
B8A568	myhz1.1	Uncharacterized protein	_NSYEETLDQLETLK(cr)R_	1501	1	LDQLETLKRENKNLQ	P12882	MYH1	Myosin-1	82.1	3208	0E+00	LDQLETLKRENKNLQ	1503	K	51.1	3E-10
B8A561	myhz1.2	Uncharacterized protein	_K(cr)AVETEK_	1533	1	HELEKSKKAVETEKA	P12882	MYH1	Myosin-1	82.1	3207	0E+00	HELEKIKKQVEQEKS	1535	K	31.6	3E-03
Q6IQX1	myhz2	Uncharacterized protein	_VQLELNQVK(cr)GEIDR_	1571	1	QLELNQVKGEIDRKL	P12882	MYH1	Myosin-1	82.1	3210	0E+00	QLELNQVKSEVDRKI	1573	K	41.8	7E-07
B8A561	myhz1.2	Uncharacterized protein	_VQLELNQVK(cr)SEIDRK_	1571	1	QLELNQVKSEIDRKL	P12882	MYH1	Myosin-1	82.1	3207	0E+00	QLELNQVKSEVDRKI	1573	K	45.2	4E-08
B8A568	myhz1.1	Uncharacterized protein	_VQLELNQVK(cr)SEIDRK_	1571	1	QLELNQVKSEIDRKL	P12882	MYH1	Myosin-1	82.1	3208	0E+00	QLELNQVKSEVDRKI	1573	K	45.2	4E-08
Q6IQX1	myhz2	Myosin, heavy polypeptide 2, fast muscle specific	_LAEK(cr)DEEMEQIK_	1581	1	IDRKLAEKDEEMEQI	P12882	MYH1	Myosin-1	82.1	3210	0E+00	VDRKIAEKDEEIDQM	1583	K	36.3	6E-05
B8A568	myhz1.1	Uncharacterized protein	_LAEK(cr)DEEIEQIKR_	1581	1	IDRKLAEKDEEIEQI	P12882	MYH1	Myosin-1	82.1	3208	0E+00	VDRKIAEKDEEIDQM	1583	K	40.1	3E-06
B8A568	myhz1.1	Uncharacterized protein	_LAEKDEEIEQIK(cr)R_	1589	1	DEEIEQIKRNSQRIT	P12882	MYH1	Myosin-1	82.1	3208	0E+00	DEEIDQMKRNHIRIV	1591	K	31.2	4E-03
B8A561	myhz1.2	Uncharacterized protein	_NVQAQLK(cr)DAQLHLDDAVR_	1653	1	RNVQAQLKDAQLHLD	P12882	MYH1	Myosin-1	82.1	3207	0E+00	RNTQAILKDTQLHLD	1655	K	36.7	4E-05
B8A561	myhz1.2	Uncharacterized protein	_GQEDMK(cr)EQVAMVER_	1670	1	VRGQEDMKEQVAMVE	P12882	MYH1	Myosin-1	82.1	3207	0E+00	LRSQEDLKEQLAMVE	1674	K	38.8	8E-06
B8A568	myhz1.1	Uncharacterized protein	_GQEDMK(cr)EQVAMVER_	1670	1	VRGQEDMKEQVAMVE	P12882	MYH1	Myosin-1	82.1	3208	0E+00	LRSQEDLKEQLAMVE	1674	K	38.8	8E-06
B8A561	myhz1.2	Uncharacterized protein	_NAEDK(cr)AK_	1756	1	EARNAEDKAKKAITD	P12882	MYH1	Myosin-1	82.1	3207	0E+00	EARNAEEKAKKAITD	1758	K	43.4	2E-08
B8A568	myhz1.1	Uncharacterized protein	_NAEDK(cr)AK_	1756	1	EARNAEDKAKKAITD	P12882	MYH1	Myosin-1	82.1	3208	0E+00	EARNAEEKAKKAITD	1758	K	43.4	2E-08
B8A561	myhz1.2	Uncharacterized protein	_K(cr)AITDAAMMAEELKK_	1759	1	NAEDKAKKAITDAAM	P12882	MYH1	Myosin-1	82.1	3207	0E+00	NAEEKAKKAITDAAM	1761	K	46.9	1E-08
B8A568	myhz1.1	Uncharacterized protein	_K(cr)AITDAAMMAEELKK_	1759	1	NAEDKAKKAITDAAM	P12882	MYH1	Myosin-1	82.1	3208	0E+00	NAEEKAKKAITDAAM	1761	K	46.9	1E-08
B8A561	myhz1.2	Uncharacterized protein	_AITDAAM(ox)M(ox)AEELK(cr)K_	1772	1	AMMAEELKKEQDTSA	P12882	MYH1	Myosin-1	82.1	3207	0E+00	AMMAEELKKEQDTSA	1774	K	51.5	4E-09
B8A561	myhz1.2	Uncharacterized protein	_AITDAAMMAEELK(cr)K_	1772	1	AMMAEELKKEQDTSA	P12882	MYH1	Myosin-1	82.1	3207	0E+00	AMMAEELKKEQDTSA	1774	K	51.5	4E-09
B8A568	myhz1.1	Uncharacterized protein	_AITDAAMMAEELK(cr)K_	1772	1	AMMAEELKKEQDTSA	P12882	MYH1	Myosin-1	82.1	3208	0E+00	AMMAEELKKEQDTSA	1774	K	51.5	4E-09
B8A561	myhz1.2	Uncharacterized protein	_K(cr)NLEVTVK_	1786	1	AHLERMKKNLEVTVK	P12882	MYH1	Myosin-1	82.1	3207	0E+00	AHLERMKKNLEQTVK	1788	K	45.6	3E-08
B8A561	myhz1.2	Uncharacterized protein	_VK(cr)ELTYQTEEDKK_	1850	1	RKYERRVKELTYQTE	P12882	MYH1	Myosin-1	82.1	3207	0E+00	RKHERKVKELTYQTE	1852	K	44.3	9E-08
B8A568	myhz1.1	Uncharacterized protein	_VK(cr)ELTYQTEEDKK_	1850	1	RKYERRVKELTYQTE	P12882	MYH1	Myosin-1	82.1	3208	0E+00	RKHERKVKELTYQTE	1852	K	44.3	9E-08

B8A561	myhz1.2	Uncharacterized protein	_ELTYQTEEDK(cr)K_	1860	1	TYQTEEDKKNVNRLQ	P12882	MYH1	Myosin-1	82.1	3207	0E+00	TYQTEEDRKNILRLQ	1862	R	40.9	1E-06
B8A568	myhz1.1	Uncharacterized protein	_ELTYQTEEDK(cr)K_	1860	1	TYQTEEDKKNVNRLQ	P12882	MYH1	Myosin-1	82.1	3208	0E+00	TYQTEEDRKNILRLQ	1862	R	40.9	1E-06
B8A561	myhz1.2	Uncharacterized protein	_ELTYQTEEDKK(cr)NVNR_	1861	1	YQTEEDKKNVNRLQD	P12882	MYH1	Myosin-1	82.1	3207	0E+00	YQTEEDRKNILRLQD	1863	K	41.4	1E-06
B8A568	myhz1.1	Uncharacterized protein	_ELTYQTEEDKK(cr)NVNR_	1861	1	YQTEEDKKNVNRLQD	P12882	MYH1	Myosin-1	82.1	3208	0E+00	YQTEEDRKNILRLQD	1863	K	41.4	1E-06
B8A561	myhz1.2	Uncharacterized protein	_ADIAESQVNK(cr)LR_	1921	1	IAESQVNKLRAKSRD	P12882	MYH1	Myosin-1	82.1	3207	0E+00	IAESQVNKLRVKSRE	1923	K	43.1	4E-06
B8A568	myhz1.1	Uncharacterized protein	_ADIAESQVNK(cr)LR_	1921	1	IAESQVNKLRAKSRD	P12882	MYH1	Myosin-1	82.1	3208	0E+00	IAESQVNKLRVKSRE	1923	K	43.1	4E-06
Q6IQX1	myhz2	Myosin, heavy polypeptide 2, fast muscle specific	_DFETAQLLSK(cr)IEDEQSLGAQLQK_	1095	1	ETAQLLSKIEDEQSL	P12882	MYH1	Myosin-1	82.1	3210	0E+00	-	-	-	-	-
B8A561	myhz1.2	Uncharacterized protein	_VAK(cr)EDDVHPM(ox)NPPK_	74	1	TKEERVAKEDDVHPM	P12882	MYH1	Myosin-1	82.1	3207	0E+00	-	-	-	-	-
B8A561	myhz1.2	Uncharacterized protein	_VAK(cr)EDDVHPMNPPK_	74	1	TKEERVAKEDDVHPM	P12882	MYH1	Myosin-1	82.1	3207	0E+00	-	-	-	-	-
B8A568	myhz1.1	Uncharacterized protein	_EEK(cr)VVKEDDVHPM(ox)NPPK_	71	1	TLDTKEEKVVKEDDV	P12882	MYH1	Myosin-1	82.1	3208	0E+00	-	-	-	-	-
F1QJP3	myl10	Uncharacterized protein	_DGFIDK(cr)NDLR_	45	1	NRDGFIDKNDLRDTF	P10916	MYL2	Myosin regulatory light chain 2, ventricular/cardiac muscle isoform	71.1	251	2E-87	NRDGFIDKNDLRDTF	46	K	52.8	9E-11
F1QJP3	myl10	Uncharacterized protein	_GEEIK(cr)YHLMSQADK_	119	1	ILKGEEIKYHLMSQA	P10916	MYL2	Myosin regulatory light chain 2, ventricular/cardiac muscle isoform	71.1	251	2E-87	-	-	-	-	-
O93409	mylpfa	Uncharacterized protein	_DGIISK(cr)DDLR_	48	1	NRDGIISKDDLRDVL	Q96A32	MYLPF	Myosin regulatory light chain 2, skeletal muscle isoform	81.1	293	3E-104	NRDGIIDKEDLRDTF	47	K	38	2E-05
O93409	mylpfa	Mylz2 protein	_DVLASMGQLNVK(cr)NEELEAMIK_	64	1	SMGQLNVKNEELEAM	Q96A32	MYLPF	Myosin regulatory light chain 2, skeletal muscle isoform	81.1	293	3E-104	AMGRLNVKNEELDAM	65	K	43.1	3E-07
O93409	mylpfa	Uncharacterized protein	_LK(cr)GADPEDVIVSAFK_	93	1	TMFGEKLKGADPEDV	Q96A32	MYLPF	Myosin regulatory light chain 2, skeletal muscle isoform	81.1	293	3E-104	TMFGEKLKGADPEDV	92	K	51.1	4E-10
E9QG51	mylpfb	Uncharacterized protein	_GADPEDVIVSAFK(cr)VLDPEATGTIK_	85	1	DVIVSAFKVLDPEAT	Q96A32	MYLPF	Myosin regulatory light chain 2, skeletal muscle isoform	83.8	244	1E-85	DVITGAFKVLDPEGK	105	K	36.3	6E-05
O93409	mylpfa	Mylz2 protein	_GADPEDVIVSAFK(cr)VLDPEGTGSIK_	106	1	DVIVSAFKVLDPEGT	Q96A32	MYLPF	Myosin regulatory light chain 2, skeletal muscle isoform	81.1	293	3E-104	DVITGAFKVLDPEGK	105	K	38.8	8E-06
O93409	mylpfa	Mylz2 protein	_FTAEEMK(cr)NLWAAFPPDVAGNVDYK_	138	1	RFTAEEMKNLWAAFP	Q96A32	MYLPF	Myosin regulatory light chain 2, skeletal muscle isoform	81.1	293	3E-104	RFSQEEIKNMWAAFP	137	K	39.7	4E-06
Q9I8U7	mylz3	Fast skeletal muscle myosin light polypeptide 3	_ALGQNPTNK(cr)DVK_	47	1	LGQNPTNKDVKKILG	P14649	MYL6B	Myosin light chain 6B	63.1	209	2E-70	LGQNPTNAEVLKVLG	103	A	32	2E-03
B8JKH7	mylz3	Uncharacterized protein	_ALGQNPTNK(cr)DVK_	71	1	LGQNPTNKDVKKILG	P14649	MYL6B	Myosin light chain 6B	62.5	135	3E-42	LGQNPTNAEVLKVLG	103	A	32	2E-03
Q6P0G6	myl1	Uncharacterized protein	_ALGQNPTNK(cr)EVTK_	85	1	LGQNPTNKEVTKILG	P14649	MYL6B	Myosin light chain 6B	57.1	237	3E-81	LGQNPTNAEVLKVLG	103	A	35	3E-03
Q9I8U7	mylz3	Fast skeletal muscle myosin light polypeptide 3	_IDFEAFLPMLK(cr)TVDANQK_	76	1	EAFLPMLKTVDANQK	P14649	MYL6B	Myosin light chain 6B	63.1	209	2E-70	-	-	-	-	-
Q9I8U7	mylz3	Fast skeletal muscle myosin light polypeptide 3	_RIDFEAFLPMLK(cr)TVDANQK_	76	1	EAFLPMLKTVDANQK	P14649	MYL6B	Myosin light chain 6B	63.1	209	2E-70	-	-	-	-	-
B8JKH7	mylz3	Uncharacterized protein	_IDFEAFLPMLK(cr)TVDANQK_	100	1	EAFLPMLKTVDANQK	P14649	MYL6B	Myosin light chain 6B	62.5	135	3E-42	-	-	-	-	-
Q9I8U7	mylz3	Fast skeletal muscle myosin light polypeptide 3	_TVDANQK(cr)GTYDDYVEGLR_	83	1	KTVDANQKGTYDDYV	P14649	MYL6B	Myosin light chain 6B	63.1	209	2E-70	-	-	-	-	-

Q9I8U7	mylz3	Fast skeletal muscle myosin light polypeptide 3	_K(cr)ILGDPSADDM(ox)ANK_	51	1	PTNKDVKKILGDPSA	P14649	MYL6B	Myosin light chain 6B	63.1	209	2E-70	-	-	-	-	-
Q9I8U7	mylz3	Fast skeletal muscle myosin light polypeptide 3	_K(cr)ILGDPSADDMANK_	51	1	PTNKDVKKILGDPSA	P14649	MYL6B	Myosin light chain 6B	63.1	209	2E-70	-	-	-	-	-
B8JKH7	mylz3	Uncharacterized protein	_K(cr)ILGDPSADDMANK_	75	1	PTNKDVKKILGDPSA	P14649	MYL6B	Myosin light chain 6B	62.5	135	3E-42	-	-	-	-	-
B8JKH7	mylz3	Uncharacterized protein	_ILGDPSADDM(ox)ANK(cr)R_	88	1	SADDMANKRIDFEAF	P14649	MYL6B	Myosin light chain 6B	62.5	135	3E-42	-	-	-	-	-
Q6P0G6	myl1	Uncharacterized protein	_ADAVDLSGVK(cr)LDFTQDQM(ox)EDYR_	40	1	AVDLSGVKLDFTQDQ	P14649	MYL6B	Myosin light chain 6B	57.1	237	3E-81	-	-	-	-	-
Q6P0G6	myl1	Uncharacterized protein	_ADAVDLSGVK(cr)LDFTQDQMEDYR_	40	1	AVDLSGVKLDFTQDQ	P14649	MYL6B	Myosin light chain 6B	57.1	237	3E-81	-	-	-	-	-
Q6P0G6	myl1	Uncharacterized protein	_EVTK(cr)ILGNPTADDMVNKR_	89	1	PTNKEVTKILGNPTA	P14649	MYL6B	Myosin light chain 6B	57.1	237	3E-81	-	-	-	-	-
Q6P0G6	myl1	Uncharacterized protein	_ILGNPTADDMVNK(cr)R_	102	1	TADDMVNKRVDFEGF	P14649	MYL6B	Myosin light chain 6B	57.1	237	3E-81	-	-	-	-	-
E7EYE1	si:dkey-78p20.4	Uncharacterized protein	_TELNYEILEK(cr)GHVR_	826	1	LNYEILEKGHVRFWV	P54296	MYOM2	Myomesin-2	56.1	1132	0E+00	LAYEIFDKGRVRFWL	1029	K	35.4	1E-04
Q7T334	mdh2	Malate dehydrogenase	_NLGLGK(cr)LSAFEEK_	306	1	EKNLGLGKLSAFEEK	P40926	MDH2	Malate dehydrogenase, mitochondrial	84.6	595	0E+00	EKNLGIGKVSSFEEK	307	K	38.8	8E-06
Q7T334	mdh2	Malate dehydrogenase	_LVADAMTELK(cr)GSIK_	323	1	ADAMTELKGSIKKGE	P40926	MDH2	Malate dehydrogenase, mitochondrial	84.6	595	0E+00	SDAIPELKASIKKGE	323	K	33.3	7E-04
H0WE86	mdh1aa	Uncharacterized protein	_EVIPTDK(cr)VEVGFK_	90	1	REVIPTDKVEVGFKD	P40925	MDH1	Malate dehydrogenase, cytoplasmic	84.2	165	1E-52	-	-	-	-	-
B3DFN3	lmnb2	Lamin B2	_VSEK(cr)EEVTTR_	61	1	LQFKVSEKEEVTTRE	Q03252	LMNB2	Lamin-B2	67.7	754	0E+00	LLLKISEKEEVTTRE	87	K	38.4	1E-05
B3DFN3	lmnb2	Lamin B2	_EVSGIK(cr)SLYEAELADAR_	73	1	TREVSGIKSLYEAEL	Q03252	LMNB2	Lamin-B2	67.7	754	0E+00	TREVSGIKALYESEL	93	K	44.3	9E-08
B3DFN3	lmnb2	Lamin B2	_K(cr)SMFDEEVR_	203	1	TEELEFRKSMFDEEV	Q03252	LMNB2	Lamin-B2	67.7	754	0E+00	QEELDFRKSVFEEEV	225	K	40.5	2E-06
B3DFN3	lmnb2	Lamin B2	_FSPK(cr)FVLK_	486	1	IVYKFSPKFVLKAGQ	Q03252	LMNB2	Lamin-B2	67.7	754	0E+00	IAYKFTPKYILRAGQ	520	K	36.3	6E-05
B3DFN3	lmnb2	Lamin B2	_IK(cr)DLEAQYNK_	130	1	AAALVRIKDLEAQYN	Q03252	LMNB2	Lamin-B2	67.7	754	0E+00	-	-	-	-	-
F1QHC4	lmnb1	Uncharacterized protein	_INELEGALSHEK(cr)DLSR_	333	1	EGALSHEKDLSRRLL	P20700	LMNB1	Lamin-B1	70.0	749	0E+00	-	-	-	-	-
A2CE73	khdrbs1a	Uncharacterized protein	_FNFVGK(cr)ILGPQGSTIK_	88	1	PRFNFVGKILGPQGS	Q07666	KHDRBS1	KH domain-containing, RNA-binding, signal transduction-associated protein 1	56.8	360	2E-123	PKFNFVGKILGPQGN	175	K	44.3	9E-08
A2CE73	khdrbs1a	Uncharacterized protein	_M(ox)AHAMDEVK(cr)K_	165	1	AHAMDEVKKFLMPVE	Q07666	KHDRBS1	KH domain-containing, RNA-binding, signal transduction-associated protein 1	56.8	360	2E-123	AHAMEEVKKFLVPDM	252	K	39.7	4E-06
A2CE73	khdrbs1a	Uncharacterized protein	_MAHAMDEVK(cr)K_	165	1	AHAMDEVKKFLMPVE	Q07666	KHDRBS1	KH domain-containing, RNA-binding, signal transduction-associated protein 1	56.8	360	2E-123	AHAMEEVKKFLVPDM	252	K	39.7	4E-06
F1QK60	krt4	Uncharacterized protein	_LEGELK(cr)NMQGLVEDFK_	189	1	MKLEGELKNMQGLVE	P05787	KRT8	Keratin, type II cytoskeletal 8	76.8	578	0E+00	LKLEAELGNMQGLVE	166	G	38.8	8E-06
F1R5A5	krt5	Uncharacterized protein	_AAVENEFVLLK(cr)K_	263	1	ENEFVLLKKDVDAAY	P05787	KRT8	Keratin, type II cytoskeletal 8	74.1	573	0E+00	ENEFVLIKKDVDEAY	197	K	44.3	1E-06
F1QK60	krt4	Uncharacterized protein	_ASVENEFVLLK(cr)K_	220	1	ENEFVLLKKDVDAAY	P05787	KRT8	Keratin, type II cytoskeletal 8	76.8	578	0E+00	ENEFVLIKKDVDEAY	197	K	44.3	1E-06
Q6NWF6	krt8	Keratin, type II cytoskeletal 8	_DVDEAYMNK(cr)VELEAK_	231	1	VDEAYMNKVELEAKL	P05787	KRT8	Keratin, type II cytoskeletal 8	68.4	588	0E+00	VDEAYMNKVELESRL	207	K	46.4	2E-08
Q6NWF6	krt8	Lamin B2	_K(cr)LLEGEEDR_	417	1	IEIATYRKLLEGEED	P05787	KRT8	Keratin, type II cytoskeletal 8	68.4	588	0E+00	IEIATYRKLLEGEES	393	K	48.1	4E-09
F1QK60	krt4	Uncharacterized protein	_GELAVK(cr)DAK_	370	1	ERGELAVKDAKLRIR	P05787	KRT8	Keratin, type II cytoskeletal 8	76.8	578	0E+00	-	-	-	-	-
F1QK60	krt4	Uncharacterized protein	_QLDGLGNEK(cr)MK_	181	1	LDGLGNEKMKLEGEL	P05787	KRT8	Keratin, type II cytoskeletal 8	76.8	578	0E+00	-	-	-	-	-

F1QEW2	si:dkey-183i3.5	Uncharacterized protein (Fragment)	_FASFIDK(cr)AR_	191	1	HNAVLRAKISMFTNP	O95678	KRT75	Keratin, type II cytoskeletal 75	30.8	186	1E-52	-	-	-	-	-
F1QK60	krt4	Uncharacterized protein	_AEAESWYK(cr)QK_	306	1	AEAESWYKQKFEEMQ	P02538	KRT6A	Keratin, type II cytoskeletal 6A	70.0	533	0E+00	AEAESWYQTKYEELQ	357	Q	38.4	1E-05
F1QK60	krt4	Uncharacterized protein	_QK(cr)FEEMQSTAGQYGDDLR_	308	1	AESWYKQKFEEMQST	Q9NSB2	KRT84	Keratin, type II cuticular Hb4	52.0	496	2E-171	AEAWYQTKYEEMQVT	359	K	34.6	3E-04
Q7ZTS4	krt18	Keratin, type I cytoskeletal 18	_K(cr)VIDDTNLNR_	191	1	ADITGLRKVIDDTNL	P05783	KRT18	Keratin, type I cytoskeletal 18	54.1	436	2E-152	NDIHGLRKVIDDTNI	189	K	38.4	1E-05
Q7ZTS4	krt18	Keratin, type I cytoskeletal 18	_LLDGGDFK(cr)LQDALEEQK_	394	1	LLDGGDFKLQDALEE	P05783	KRT18	Keratin, type I cytoskeletal 18	54.1	436	2E-152	-	-	-	-	-
E9QDY3	cyt1	Uncharacterized protein	_K(cr)VLDELTM(ox)TR_	211	1	ADIVGLRKVLDELTM	Q04695	KRT17	Keratin, type I cytoskeletal 17	60.5	416	5E-144	ADINGLRRVLDELTL	192	R	37.1	3E-05
E9QDY3	cyt1	Uncharacterized protein	_K(cr)VLDELTMTR_	211	1	ADIVGLRKVLDELTM	Q04695	KRT17	Keratin, type I cytoskeletal 17	60.5	416	5E-144	ADINGLRRVLDELTL	192	R	37.1	3E-05
E9QDY3	cyt1	Uncharacterized protein	_EELIFLK(cr)K_	238	1	KEELIFLKKNHEEEL	Q04695	KRT17	Keratin, type I cytoskeletal 17	60.5	416	5E-144	KEELAYLKKNHEEEM	219	K	41.4	1E-06
E9QDY3	cyt1	Uncharacterized protein	_SEITEVK(cr)R_	323	1	RSEITEVKRTLQSLE	Q04695	KRT17	Keratin, type I cytoskeletal 17	60.5	416	5E-144	KSEISELRRTMQALE	305	R	31.6	3E-03
E9QDY3	cyt1	Uncharacterized protein	_SESLNK(cr)EVAVSTETLQTSR_	303	1	AKSESLNKEVAVSTE	Q04695	KRT17	Keratin, type I cytoskeletal 17	60.5	416	5E-144	-	-	-	-	-
E9QDY3	cyt1	Uncharacterized protein	_ANADLELK(cr)IR_	134	1	ANADLELKIRQFLDS	Q04695	KRT17	Keratin, type I cytoskeletal 17	60.5	416	5E-144	-	-	-	-	-
E9QDY3	cyt1	Uncharacterized protein	_K(cr)DLESWFQAK_	288	1	AVSAKNRKDLESWFQ	Q04695	KRT17	Keratin, type I cytoskeletal 17	60.5	416	5E-144	-	-	-	-	-
E9QDY3	cyt1	Uncharacterized protein	_ATTETK(cr)TT_	448	1	SKATTETKTT_____	Q04695	KRT17	Keratin, type I cytoskeletal 17	60.5	416	5E-144	-	-	-	-	-
E9QDY3	cyt1	Uncharacterized protein	_DLESWFQAK(cr)SESLNK_	297	1	LESWFQAKSESLNKE	P08779	KRT16	Keratin, type I cytoskeletal 16	60.0	398	1E-135	AETWFLSKTEELNKE	313	K	31.2	4E-03
E7F4R9	-	Isocitrate dehydrogenase [NADP]	_NTILK(cr)AYDGR_	252	1	STKNTILKAYDGRFK	P48735	IDH2	Isocitrate dehydrogenase [NADP], mitochondrial	61.4	536	0E+00	STKNTILKAYDGRFK	256	K	50.3	7E-10
E7F4R9	-	Isocitrate dehydrogenase [NADP]	_FK(cr)DIFQDIFEK_	259	1	KAYDGRFKDIFQDIF	P48735	IDH2	Isocitrate dehydrogenase [NADP], mitochondrial	61.4	536	0E+00	KAYDGRFKDIFQEIF	263	K	51.1	4E-10
E7F4R9	-	Isocitrate dehydrogenase [NADP]	_TNLDK(cr)ALGK_	443	1	AIKTNLDKALGK___	P48735	IDH2	Isocitrate dehydrogenase [NADP], mitochondrial	61.4	536	0E+00	-	-	-	-	-
Q08CK7	igf2bp1	Insulin-like growth factor 2 mRNA-binding protein 1	_QTQSK(cr)IDVHR_	231	1	ITKQTQSKIDVHRKE	Q9NZI8	IGF2BP1	Insulin-like growth factor 2 mRNA-binding protein 1	80.6	912	0E+00	ITKQTQSKIDVHRKE	228	K	51.1	4E-10
Q08CK7	igf2bp1	Insulin-like growth factor 2 mRNA-binding protein 1	_LKEENFFGPK(cr)EEVK_	485	1	EENFFGPKEEVKLET	Q9NZI8	IGF2BP1	Insulin-like growth factor 2 mRNA-binding protein 1	80.6	912	0E+00	EENFFGPKEEVKLET	483	K	52	2E-10
Q08CK7	igf2bp1	Insulin-like growth factor 2 mRNA-binding protein 1	_VTAEDLVK(cr)TFEDYK_	20	1	VTAEDLVKTFEDYKI	Q9NZI8	IGF2BP1	Insulin-like growth factor 2 mRNA-binding protein 1	80.6	912	0E+00	-	-	-	-	-
E7F7C7	-	Histone H4	_GVLK(cr)VFLENVIR_	60	1	EETRGVLKVFLENVI	P62805	HIST1H4A	Histone H4	97.0	190	9E-65	EETRGVLKVFLENVI	60	K	50.7	5E-10
E7F7C7	-	Histone H4	_DAVTYTEHAK(cr)R_	78	1	VTYTEHAKRKTVTAM	P62805	HIST1H4A	Histone H4	97.0	190	9E-65	VTYTEHAKRKTVTAM	78	K	51.5	3E-10
Q4QRF4	si:dkeyp-46h3.6	Histone H3.2	_VTIM(ox)PK(cr)DIQLAR_	123	1	KRVTIMPKDIQLARR	Q71DI3	HIST2H3A;	Histone H3.2	100.0	274	9E-98	KRVTIMPKDIQLARR	123	K	52.4	1E-10
Q4QRF4	si:dkeyp-46h3.6	Histone H3.2	_VTIMPK(cr)DIQLAR_	123	1	KRVTIMPKDIQLARR	Q71DI3	HIST2H3A;	Histone H3.2	100.0	274	9E-98	KRVTIMPKDIQLARR	123	K	52.4	1E-10
Q05AK8	si:ch73-368j24.9	Histone H2B	_PEPAK(cr)TAPK_	6	1	__MPEPAKTAPKKGS	Q16778	HIST2H2B E	Histone H2B type 2-E	92.9	231	3E-81	__MPEPAKSAPAPKK	6	K	35.8	6E-05
E7F2T3	si:ch1073-159d7.7	Uncharacterized protein	_DLIVK(cr)TVTASK_	53	1	NVRDLIVKTVTASKE	P16401	HIST1H1B	Histone H1.5	64.3	105	6E-29	-	-	-	-	-
A3KPR3	histh1l	Uncharacterized protein	_IALK(cr)ALVK_	75	1	SRVKIALKALVKRGA	P16401	HIST1H1B	Histone H1.5	64.7	109	1E-30	-	-	-	-	-
E7F225	zgc:163061	Uncharacterized protein	_GMVTK(cr)GVLK_	82	1	AIKGMVTKGVLKQVK	P16402	HIST1H1D	Histone H1.3	66.4	103	2E-29	-	-	-	-	-
E7F225	zgc:163061	Uncharacterized protein	_LAIK(cr)GMVTK_	77	1	SRVKLAIKGMVTKGV	P16402	HIST1H1D	Histone H1.3	66.4	103	2E-29	-	-	-	-	-

E7F225	zgc:163061	Uncharacterized protein	_GVLK(cr)QVK_	86	1	MVTKGVLKQVKGTGA	P07305	H1F0	Histone H1.0	44.0	73.9	1E-17	LVTTGVLKQTKGVGA	82	K	31.2	4E-03
Q5RHQ7	hnrnpub	Uncharacterized protein	_DEASK(cr)LLEK_	581	1	LQKDEASKLLEKYKE	Q00839	HNRNPU	Heterogeneous nuclear ribonucleoprotein U	58.6	723	0E+00	LQKEEAQKLLEQYKE	664	K	38.8	8E-06
E7F3J2	hnrnpua	Uncharacterized protein	_AFTVSK(cr)DELK_	345	1	GKAFTVSKDELKGKP	Q00839	HNRNPU	Heterogeneous nuclear ribonucleoprotein U	59.0	693	0E+00	-	-	-	-	-
Q7SXN2	hnrpdl	Heterogeneous nuclear ribonucleoprotein D-like	_VAQPK(cr)EVYR_	193	1	EIKVAQPKEVYRQQQ	O14979	HNRNPDL	Heterogeneous nuclear ribonucleoprotein D-like	74.0	390	1E-136	EIKVAQPKEVYRQQQ	311	K	52.4	1E-10
E7FAQ8	hnrnpa1a	Uncharacterized protein	_K(cr)MFVGGIK_	107	1	GAHSTVKKMFVGGIK	P09651	HNRNPA1	Heterogeneous nuclear ribonucleoprotein A1	81.8	347	1E-118	GAHLTVKKIFVGGIK	106	K	38.4	1E-05
F1QTL9	hnrnpa0b	Uncharacterized protein	_PHVVDGK(cr)NVEVK_	76	1	RPHVVDGKNVEVKRA	Q13151	HNRNPA0	Heterogeneous nuclear ribonucleoprotein A0	55.0	229	5E-74	SPHAVDGNTVELKRA	76	N	31.6	3E-03
F1QS28	hnrnpa0a	Uncharacterized protein	_K(cr)IFIGGLK_	102	1	EALAKVKKIFIGGLK	Q13151	HNRNPA0	Heterogeneous nuclear ribonucleoprotein A0	68.7	261	2E-87	GAHAKVKKLFVGGLK	105	K	32.9	1E-03
F1QTL9	hnrnpa0b	Uncharacterized protein	_SEVITDK(cr)DTGK_	134	1	KSEVITDKDTGKKRG	Q13151	HNRNPA0	Heterogeneous nuclear ribonucleoprotein A0	55.0	229	5E-74	KAEIIADKQSGKKRG	133	K	33.3	7E-04
F1Q6U4	hbae3	Uncharacterized protein	_ANVK(cr)AFFDK_	12	1	AKDKANVKAFFDKVA	P02008	HBZ	Hemoglobin subunit zeta	42.7	112	5E-34	-	-	-	-	-
F1Q6U4	hbae3	Uncharacterized protein	_VAPK(cr)AEEIGR_	21	1	FFDKVAPKAEEIGRE	P02008	HBZ	Hemoglobin subunit zeta	42.7	112	5E-34	-	-	-	-	-
F1Q6U4	hbae3	Uncharacterized protein	_AFFDK(cr)VAPK_	17	1	NVKAFFDKVAPKAEE	P02008	HBZ	Hemoglobin subunit zeta	42.7	112	5E-34	-	-	-	-	-
Q7ZT21	hbae1	Embryonic alpha globin e1	_AAVK(cr)TLWAK_	12	1	AKDKAAVKTLWAKIA	P02008	HBZ	Hemoglobin subunit zeta	54.5	164	7E-54	-	-	-	-	-
F6NT71	hbbe1.1	Uncharacterized protein	_TVLK(cr)GLELAVK_	11	1	AHGKTVLKGLELAVK	P02100	HBE1	Hemoglobin subunit epsilon	52.9	101	7E-30	-	-	-	-	-
F6NT71	hbbe1.1	Uncharacterized protein	_GLELAVK(cr)NMDNIK_	18	1	KGLELAVKNMDNIKA	P02100	HBE1	Hemoglobin subunit epsilon	52.9	101	7E-30	-	-	-	-	-
O93548	bE1	Embryonic 1 beta-globin	_TVLK(cr)GLELAVK_	70	1	AHGKTVLKGLELAVK	P02100	HBE1	Hemoglobin subunit epsilon	51.7	164	9E-54	-	-	-	-	-
O93548	bE1	Embryonic 1 beta-globin	_GLELAVK(cr)NM(ox)DNIK_	77	1	KGLELAVKNMDNIKA	P02100	HBE1	Hemoglobin subunit epsilon	51.7	164	9E-54	-	-	-	-	-
O93548	bE1	Embryonic 1 beta-globin	_ATIQDIFAK(cr)ADYDVIGPQALAR_	18	1	TIQDIFAKADYDVIG	P02100	HBE1	Hemoglobin subunit epsilon	51.7	164	9E-54	-	-	-	-	-
O93548	bE1	Embryonic 1 beta-globin	_GLELAVK(cr)NMDNIK_	18	1	TIQDIFAKADYDVIG	P02100	HBE1	Hemoglobin subunit epsilon	51.7	164	9E-54	-	-	-	-	-
E7EZ16	-	Uncharacterized protein	_DLK(cr)IDIIPNVQER_	71	1	LDSGKDLKIDIIPNV	P08238	HSP90AB1	Heat shock protein HSP 90-beta	90.0	1220	0E+00	LDSGKELKIDIIPNP	72	K	44.8	6E-08
E7EZ16	-	Uncharacterized protein	_EK(cr)YIDQEELNK_	259	1	KKKKIKEKYIDQEEL	P07900	HSP90AA1	Heat shock protein HSP 90-alpha	84.0	1187	0E+00	KKKKIKEKYIDQEEL	283	K	52	2E-10
E7EZ16	-	Uncharacterized protein	_EMLQQSK(cr)ILK_	383	1	REMLQQSKILKVIRK	P07900	HSP90AA1	Heat shock protein HSP 90-alpha	84.0	1187	0E+00	REMLQQSKILKVIRK	407	K	52	2E-10
Q90474	hsp90a.1	Heat shock protein HSP 90-alpha 1	_DTQK(cr)HIYYITGETK_	482	1	SRMKDTQKHIYYITG	P07900	HSP90AA1	Heat shock protein HSP 90-alpha	84.4	1191	0E+00	TRMKENQKHIYYITG	491	K	45.2	4E-08
E7EZ16	-	Uncharacterized protein	_AEADKNDK(cr)AVK_	633	1	AEADKNDKAVKDLVI	P07900	HSP90AA1	Heat shock protein HSP 90-alpha	84.0	1187	0E+00	AEADKNDKSVKDLVI	657	K	46.4	2E-08
Q6NYR4	hspa8	Heat shock cognate 71 kDa protein	_NQVAM(ox)NPTNTVFDAK(cr)R_	71	1	TNTVFDAKRLIGRRF	P11142	HSPA8	Heat shock cognate 71 kDa protein	95.8	1218	0E+00	TNTVFDAKRLIGRRF	71	K	51.1	4E-10
Q6NYR4	hspa8	Heat shock cognate 71 kDa protein	_PK(cr)VQVEYK_	102	1	INDNSRPKVQVEYKG	P11142	HSPA8	Heat shock cognate 71 kDa protein	95.8	1218	0E+00	VNDAGRPKVQVEYKG	102	K	40.5	2E-06
Q90473	hspa8	Heat shock cognate 71 kDa protein	_MK(cr)EIAEAYLGK_	128	1	SMVLTKMKEIAEAYL	P11142	HSPA8	Heat shock cognate 71 kDa protein	94.6	1202	0E+00	SMVLTKMKEIAEAYL	128	K	52.4	1E-10
Q6NYR4	hspa8	Heat shock cognate 71 kDa protein	_MK(cr)EIAEAYLGK_	128	1	SMVLTKMKEIAEAYL	P11142	HSPA8	Heat shock cognate 71 kDa protein	95.8	1218	0E+00	SMVLTKMKEIAEAYL	128	K	52.4	1E-10
Q90473	hspa8	Heat shock cognate 71 kDa protein	_GTLDPVEK(cr)ALR_	319	1	GTLDPVEKALRDAKM	P11142	HSPA8	Heat shock cognate 71 kDa protein	94.6	1202	0E+00	GTLDPVEKALRDAKL	319	K	46	2E-08
Q6NYR4	hspa8	Heat shock cognate 71 kDa protein	_GTLDPVEK(cr)ALR_	319	1	GTLDPVEKALRDAKM	P11142	HSPA8	Heat shock cognate 71 kDa protein	95.8	1218	0E+00	GTLDPVEKALRDAKL	319	K	46	2E-08

Q6NYR4	hspa8	Heat shock cognate 71 kDa protein	_ENK(cr)ITITNDK_	500	1	KSTGKENKITITNDK	P11142	HSPA8	Heat shock cognate 71 kDa protein	95.8	1218	0E+00	KSTGKENKITITNDK	500	K	49.4	1E-09
Q90473	hspa8	Heat shock cognate 71 kDa protein	_STVEDEK(cr)LK_	557	1	KSTVEDEKLKGKISD	P11142	HSPA8	Heat shock cognate 71 kDa protein	94.6	1202	0E+00	KATVEDEKLQGKIND	557	K	39.2	6E-06
Q90473	hspa8	Heat shock cognate 71 kDa protein	_EEFEHQQK(cr)ELEK_	597	1	EEFEHQQKELEKVCN	P11142	HSPA8	Heat shock cognate 71 kDa protein	94.6	1202	0E+00	EEFEHQQKELEKVCN	597	K	54.1	3E-11
Q6NYR4	hspa8	Heat shock cognate 71 kDa protein	_EEFEHQQK(cr)ELEK_	597	1	EEFEHQQKELEKVCN	P11142	HSPA8	Heat shock cognate 71 kDa protein	95.8	1218	0E+00	EEFEHQQKELEKVCN	597	K	54.1	3E-11
Q6NZU0	hspa4b	Heat shock protein 4	_QIYIDK(cr)LAELK_	676	1	PKQIYIDKLAELKNL	P34932	HSPA4	Heat shock 70 kDa protein 4	71.6	1236	0E+00	PKQVYVDKLAELKNL	674	K	46.4	2E-08
I3ISK4	gnb3b	Uncharacterized protein	_TPVIK(cr)TVK_	132	1	NLKTPVIKTVKELDA	P16520	GNB3	Guanine nucleotide-binding protein G	62.9	200	2E-65	-	-	-	-	-
Q71N41	gamt	Guanidinoacetate N-methyltransferase	_NK(cr)YDNIDK_	179	1	WGELLKNKYDNIDKM	Q14353	GAMT	Guanidinoacetate N-methyltransferase	73.6	365	1E-130	-	-	-	-	-
P79735	ran	GTP-binding nuclear protein Ran	_AK(cr)SIVFHR_	133	1	KDRKVKAKSIVFHRK	P62826	RAN	GTP-binding nuclear protein Ran	94.9	428	4E-156	KDRKVKAKSIVFHRK	134	K	50.3	1E-08
Q6NV37	gyg1a	Glycogenin 1	_AVLHK(cr)IYDEVR_	53	1	PSRAVLHKIYDEVRL	P46976	GYG1	Glycogenin-1	60.1	445	1E-158	-	-	-	-	-
Q503C7	pygma	Alpha-1,4 glucan phosphorylase	_VLYPNDNFFEGK(cr)ELR_	290	1	NDNFFEGKELRLKQE	P11217	PYGM	Glycogen phosphorylase, muscle form	85.0	1495	0E+00	NDNFFEGKELRLKQE	290	K	52.4	1E-10
E9QGA9	gapdh	Glyceraldehyde-3-phosphate dehydrogenase	_AAFLTK(cr)K_	24	1	TRAAFLTKKVEIVAI	P04406	GAPDH	Glyceraldehyde-3-phosphate dehydrogenase	86.5	404	2E-144	TRAAFNSGKVDIVAI	26	G	31.6	3E-03
E9QGA9	gapdh	Glyceraldehyde-3-phosphate dehydrogenase	_GASQNIIPASTGAAK(cr)AVGK_	213	1	PASTGAAKAVGKVIP	P04406	GAPDH	Glyceraldehyde-3-phosphate dehydrogenase	86.5	404	2E-144	PASTGAAKAVGKVIP	215	K	46	2E-08
Q6P3L9	glud1b	Glutamate dehydrogenase 1	_GATIVENK(cr)LVEDLK_	68	1	GATIVENKLVEDLKT	P00367	GLUD1	Glutamate dehydrogenase 1, mitochondrial	83.2	974	0E+00	GASIVEDKLVEDLRT	84	K	40.5	2E-06
Q58EE9	gfap	Glial fibrillary acidic protein	_SK(cr)FADLTDAANR_	272	1	TEEWYRSKFADLTDA	P14136	GFAP	Glial fibrillary acidic protein	65.7	568	0E+00	AEEWYRSKFADLTDA	261	K	50.3	7E-10
F1R431	-	Uncharacterized protein	_NLQK(cr)TVK_	603	1	QTSKNLQKTVKLPRL	P06396	GSN	Gelsolin	59.0	911	0E+00	-	-	-	-	-
Q6TH14	-	Enolase	_AVDHVNK(cr)DIAPK_	71	1	KAVDHVNKDIAPKLI	P09104	ENO2	Gamma-enolase	83.0	756	0E+00	KAVDHINSTIAPALI	71	S	32	2E-03
Q6GQM9	eno2	Eno2 protein	_TAIDK(cr)AGFTDK_	233	1	LIKTAIDKAGFTDKV	P09104	ENO2	Gamma-enolase	83.6	769	0E+00	LVKEAIDKAGYTEKI	233	K	33.3	7E-04
Q0D276	zgc:153846	Uncharacterized protein	_GK(cr)VIFYEDR_	3	1	_____MGKVIFYEDR	P07316	CRYGB	Gamma-crystallin B	52.5	206	2E-69	_____MGKITFYEDR	3	K	31.6	1E-03
Q15I85	crygEM2-4	Crygm2d3 protein	_MK(cr)VTFFEDR_	2	1	MKVTFFEDR______	P07316	CRYGB	Gamma-crystallin B	50.3	194	7E-65	-	-	-	-	-
I3ISQ0	lgals2b	Galectin	_GDVK(cr)IISVK_	118	1	VHVKGDVKIISVKAK	P09382	LGALS1	Galectin-1	47.2	127	1E-39	-	-	-	-	-
E9QBQ4	aldob	Fructose-bisphosphate aldolase	_GVLFPK(cr)VIK_	96	1	DKGVLFPKVIKDKGI	P09972	ALDOC	Fructose-bisphosphate aldolase C	79.3	346	1E-121	-	-	-	-	-
Q8UVG7	fabp3	Fatty acid binding protein 3, muscle and heart	_LLHVQK(cr)WDGK_	97	1	GKLLHVQKWDGKETT	P05413	FABP3	Fatty acid-binding protein, heart	74.0	199	4E-68	GKLVHLQKWDGQETT	97	K	39.7	4E-06
F8W3N3	fabp7a	Uncharacterized protein	_LVDSQNFDEYMK(cr)SLGVGFATR_	22	1	QNFDEYMKSLGVGFA	O15540	FABP7	Fatty acid-binding protein, brain	83.9	212	2E-73	QNFDEYMKALGVGFA	22	K	49.8	1E-09
H0WEH3	khsrp	Uncharacterized protein	_GGETIK(cr)QLQER_	198	1	GKGGETIKQLQERAG	Q92945	KHSRP	Far upstream element-binding protein 2	68.6	261	3E-84	GKGGETIKQLQERAG	257	K	48.1	4E-09
Q6ZM50	capza1b	Si:dkey-16k6.1	_TK(cr)IDWNK_	268	1	QLPVTRTKIDWNKIL	P52907	CAPZA1	F-actin-capping protein subunit alpha-1	81.5	486	2E-176	QLPVTRTKIDWNKIL	268	K	53.2	6E-11
Q6PH15	slc1a2b	Amino acid transporter	_PK(cr)HVEVR_	3	1	_____MPKHVEVRMH	P43004	SLC1A2	Excitatory amino acid transporter 2	78.7	864	0E+00	EGANNMPKQVEVRMH	17	K	35.8	3E-05
B0UY94	eif5a2	Eukaryotic translation initiation factor 5A	_LPEGDLGK(cr)EIESK_	122	1	LPEGDLGKEIESKF_	P63241	EIF5A	Eukaryotic translation initiation factor 5A-1	84.4	230	4E-80	LPEGDLGKEIEQKYD	121	K	39.2	5E-06
Q803E5	etf1b	Eukaryotic translation termination factor 1	_LQAK(cr)VLK_	249	1	FDPRLQAKVLKLVDI	P62495	ETF1	Eukaryotic peptide chain release factor subunit 1	96.2	867	0E+00	FDQRLQSKVLKLVDI	249	K	43.5	2E-07
Q68EH0	gspt1	Uncharacterized protein	_TIAIGK(cr)VLK_	555	1	GKTIAIGKVLKLVPE	P15170	GSPT1	Eukaryotic peptide chain release factor GTP-binding subunit ERF3A	87.3	875	0E+00	GKTIAIGKVLKLVPE	490	K	48.1	4E-09

Q802C9	eif4a1a	Eukaryotic translation initiation factor 4A, isoform 1A	_YLSPK(cr)YIK_	174	1	NRRYLSPKYIKMFAL	P60842	EIF4A1	Eukaryotic initiation factor 4A-I	89.7	745	0E+00	NRRYLSPKYIKMFVL	174	K	50.7	5E-10
Q7T3L3	hsp90b1	Chaperone protein GP96	_EELVK(cr)NLGTIAK_	161	1	MTKEELVKNLGTIAK	P14625	HSP90B1	Endoplasmin	85.2	1304	0E+00	MTREELVKNLGTIAK	161	K	47.3	8E-09
Q7T3L3	hsp90b1	Chaperone protein GP96	_ETLQQHK(cr)LLK_	455	1	RETLQQHKLLKVIRK	P14625	HSP90B1	Endoplasmin	85.2	1304	0E+00	RETLQQHKLLKVIRK	455	K	51.1	4E-10
Q7T3L3	hsp90b1	Chaperone protein GP96	_EAESSPFVEK(cr)LLK_	557	1	ESSPFVEKLLKKGYE	P14625	HSP90B1	Endoplasmin	85.2	1304	0E+00	ESSPFVERLLKKGYE	557	R	47.3	8E-09
Q7T3L3	hsp90b1	Chaperone protein GP96	_FDESDK(cr)AK_	603	1	VKFDESDKAKEKREA	P14625	HSP90B1	Endoplasmin	85.2	1304	0E+00	VKFDESEKTKESREA	603	K	39.7	4E-06
Q6P3J5	eef2b	Eukaryotic translation elongation factor 2	_STLTDSLVSK(cr)AGIIASAR_	42	1	LTDSLVSKAGIIASA	P13639	EEF2	Elongation factor 2	92.9	1643	0E+00	LTDSLVCKAGIIASA	42	K	42.2	5E-07
Q6P3J5	eef2b	Eukaryotic translation elongation factor 2	_IK(cr)PVLMMNK_	152	1	QAIAERIKPVLMMNK	P13639	EEF2	Elongation factor 2	92.9	1643	0E+00	QAIAERIKPVLMMNK	152	K	53.2	6E-11
Q6P3J5	eef2b	Eukaryotic translation elongation factor 2	_EETAK(cr)LIEK_	314	1	FKKEETAKLIEKLDI	P13639	EEF2	Elongation factor 2	92.9	1643	0E+00	FKKEETAKLIEKLDI	314	K	50.7	5E-10
Q6P3J5	eef2b	Eukaryotic translation elongation factor 2	_LDIK(cr)LDTEDKDK_	322	1	LIEKLDIKLDTEDKD	P13639	EEF2	Elongation factor 2	92.9	1643	0E+00	LIEKLDIKLDSEDKD	322	K	48.1	4E-09
Q6P3J5	eef2b	Eukaryotic translation elongation factor 2	_ARPFPDGLAEDIDK(cr)GDVSSR_	619	1	GLAEDIDKGDVSSRQ	P13639	EEF2	Elongation factor 2	92.9	1643	0E+00	GLAEDIDKGEVSARQ	619	K	43.5	3E-06
Q6PE25	eef1g	Elongation factor 1-gamma	_QATEQAK(cr)EEVK_	132	1	KQATEQAKEEVKRVL	P26641	EEF1G	Elongation factor 1-gamma	77.2	731	0E+00	KQATENAKEEVRRIL	132	K	39.7	4E-06
Q92005	eef1a	Elongation factor 1-alpha	_TIEK(cr)FEK_	41	1	IDKRTIEKFEKEAAE	P68104	EEF1A1	Elongation factor 1-alpha 1	92.0	882	0E+00	IDKRTIEKFEKEAAE	41	K	51.1	4E-10
Q92005	eef1a	Elongation factor 1-alpha	_FEK(cr)EAAEMGK_	44	1	RTIEKFEKEAAEMGK	P68104	EEF1A1	Elongation factor 1-alpha 1	92.0	882	0E+00	RTIEKFEKEAAEMGK	44	K	51.5	3E-10
Q92005	eef1a	Elongation factor 1-alpha	_FEEITK(cr)EVSAYIK_	172	1	ARFEEITKEVSAYIK	P68104	EEF1A1	Elongation factor 1-alpha 1	92.0	882	0E+00	KRYEEIVKEVSTYIK	174	K	38.4	1E-05
Q92005	eef1a	Elongation factor 1-alpha	_KLEDNPK(cr)ALK_	392	1	KKLEDNPKALKSGDA	P68104	EEF1A1	Elongation factor 1-alpha 1	92.0	882	0E+00	KKLEDGPKFLKSGDA	392	K	37.5	2E-05
Q92005	eef1a	Elongation factor 1-alpha	_QTVAVGVIK(cr)SVEK_	439	1	TVAVGVIKSVEKKIG	P68104	EEF1A1	Elongation factor 1-alpha 1	92.0	882	0E+00	TVAVGVIKAVDKKAA	439	K	36.3	6E-05
F1R0W9	dctn1a	Uncharacterized protein	_EALEAK(cr)DR_	239	1	AREALEAKDRYMEEM	Q14203	DCTN1	Dynactin subunit 1	69.8	1720	0E+00	AKEALEAKERYMEEM	290	K	48.1	4E-09
Q6TNU6	dldh	Dihydrolipoyl dehydrogenase	_TNADTDGLVK(cr)ILSHK_	438	1	ADTDGLVKILSHKDT	P09622	DLD	Dihydrolipoyl dehydrogenase, mitochondrial	83.6	870	0E+00	ADTDGMVKILGQKST	440	K	34.6	3E-04
F1R8W4	desma	Uncharacterized protein	_TVLIK(cr)TIETR_	452	1	SKKTVLIKTIETRDG	P17661	DES	Desmin	70.3	614	0E+00	TKKTVMIKTIETRDG	451	K	43.9	1E-07
E7FC21	nt5c2a	Uncharacterized protein	_YVVK(cr)DAK_	228	1	NLEKYVVKDAKLPLL	P49902	NT5C2	Cytosolic purine 5'-nucleotidase	90.2	737	0E+00	NLEKYVVKDGKLPLL	228	K	46.9	1E-08
Q4VBU7	cox5aa	Cox5aa protein	_GMNTLIGYDLVPEPK(cr)ILDAALR_	78	1	YDLVPEPKILDAALR	P20674	COX5A	Cytochrome c oxidase subunit 5A, mitochondrial	73.7	212	5E-73	YDMVPEPKIIDAALR	87	K	45.2	5E-08
Q6TNV0	cox4i1	Cytochrome c oxidase subunit IV isoform 1	_FVQQLSADQK(cr)SLK_	60	1	QQLSADQKSLKEKEK	P13073	COX4I1	Cytochrome c oxidase subunit 4 isoform 1, mitochondrial	63.3	244	1E-84	KHLSASQKALKEKEK	62	K	35	2E-04
A2BHA3	ckma	Uncharacterized protein	_HNNHMAK(cr)VLTK_	32	1	KHNNHMAKVLTKEMY	P06732	CKM	Creatine kinase M-type	88.5	708	0E+00	KHNNHMAKVLTLELY	32	K	44.3	9E-08
Q7T306	ckmb	Ckmb protein	_VLTK(cr)DIYNK_	35	1	HMSKVLTKDIYNKLR	P06732	CKM	Creatine kinase M-type	85.4	682	0E+00	HMAKVLTLELYKKLR	36	L	33.7	5E-04
A2BHA3	ckma	Uncharacterized protein	_VLTK(cr)EMYGK_	36	1	HMAKVLTKEMYGKLR	P06732	CKM	Creatine kinase M-type	88.5	708	0E+00	HMAKVLTLELYKKLR	36	L	37.1	3E-05
A2BHA3	ckma	Uncharacterized protein	_TDLNFENLK(cr)GGDDLDPNYVLSSR_	116	1	DLNFENLKGGDDLDP	P06732	CKM	Creatine kinase M-type	88.5	708	0E+00	DLNHENLKGGDDLDP	116	K	44.8	6E-08
Q7T306	ckmb	Uncharacterized protein	_GK(cr)YYPLK_	171	1	LDGEFKGKYYPLKDM	P06732	CKM	Creatine kinase M-type	85.4	682	0E+00	LTGEFKGKYYPLKSM	172	K	43.1	2E-07
A2BHA3	ckma	Uncharacterized protein	_GK(cr)YYPLK_	172	1	LDGEFKGKYYPLKSM	P06732	CKM	Creatine kinase M-type	88.5	708	0E+00	LTGEFKGKYYPLKSM	172	K	47.3	8E-09
A2BHA3	ckma	Uncharacterized protein	_LSVEALSSLDGEFK(cr)GK_	170	1	SSLDGEFKGKYYPLK	P06732	CKM	Creatine kinase M-type	88.5	708	0E+00	NSLTGEFKGKYYPLK	172	K	42.6	4E-07
Q7T306	ckmb	Uncharacterized protein	_GGNMK(cr)EVFK_	246	1	MQKGGNMKEVFKRFC	P06732	CKM	Creatine kinase M-type	85.4	682	0E+00	MEKGGNMKEVFRRFC	247	K	49	2E-09

A2BHA3	ckma	Uncharacterized protein	_GGNMK(cr)EVFK_	247	1	MQKGGNMKEVFKRFC	P06732	CKM	Creatine kinase M-type	88.5	708	0E+00	MEKGGNMKEVFRRFC	247	K	49	2E-09
Q7T306	ckmb	Uncharacterized protein	_GGVHVK(cr)LPK_	297	1	LRGGVHVKLPKLSTH	P06732	CKM	Creatine kinase M-type	85.4	682	0E+00	LRGGVHVKLAHLSKH	298	K	35	2E-04
A2BHA3	ckma	Uncharacterized protein	_GGVHVK(cr)LPK_	298	1	LRGGVHVKLPKLSTH	P06732	CKM	Creatine kinase M-type	88.5	708	0E+00	LRGGVHVKLAHLSKH	298	K	35	2E-04
Q7T306	ckmb	Uncharacterized protein	_LSTHAK(cr)FEEILTR_	306	1	PKLSTHAKFEEILTR	P06732	CKM	Creatine kinase M-type	85.4	682	0E+00	AHLSKHPKFEEILTR	311	K	36.7	4E-05
A2BHA3	ckma	Uncharacterized protein	_LSTHAK(cr)FEEILTR_	307	1	PKLSTHAKFEEILTR	P06732	CKM	Creatine kinase M-type	88.5	708	0E+00	AHLSKHPKFEEILTR	311	K	36.7	4E-05
Q7T306	ckmb	Uncharacterized protein	_LMVEMEK(cr)K_	364	1	KLMVEMEKKLEKGES	P06732	CKM	Creatine kinase M-type	85.4	682	0E+00	KLMVEMEKKLEKGQS	365	K	49	2E-09
A2BHA3	ckma	Uncharacterized protein	_LMVEMEK(cr)K_	365	1	KLMVEMEKKLEKGES	P06732	CKM	Creatine kinase M-type	88.5	708	0E+00	KLMVEMEKKLEKGQS	365	K	49	2E-09
Q8AY63	ckbb	Brain-subtype creatine kinase	_GK(cr)YYALK_	172	1	LDGDLKGKYYALKDM	P12277	CKB	Creatine kinase B-type	85.8	706	0E+00	LDGDLAGRYYALKSM	172	R	38	2E-05
Q8AY63	ckbb	Brain-subtype creatine kinase	_LPNVSK(cr)HEK_	304	1	VKLPNVSKHEKFGEI	P12277	CKB	Creatine kinase B-type	85.8	706	0E+00	IKLPNLGKHEKFSEV	304	K	35.4	1E-04
Q8AY63	ckbb	Brain-subtype creatine kinase	_HEK(cr)FGEILK_	307	1	PNVSKHEKFGEILKR	P12277	CKB	Creatine kinase B-type	85.8	706	0E+00	PNLGKHEKFSEVLKR	307	K	38	2E-05
B8JN01	-	Histone H2A	_DFLETVK(cr)ELR_	241	1	KDFLETVKELRKSQG	Q9P0M6	H2AFY2	Core histone macro-H2A.2	69.0	335	2E-115	KEFLETVKELRKSQG	246	K	47.3	8E-09
E7FEZ9	atox1	Copper chaperone Atox1	_FDIDLPNK(cr)K_	37	1	FDIDLPNKKVFIESD	O00244	ATOX1	Copper transport protein ATOX1	71.2	93.6	3E-28	YDIDLPNKKVCIESE	38	K	37.5	2E-05
E7EYX7	cirbpb	Uncharacterized protein	_YGTIAK(cr)VDVIR_	33	1	SKYGTIAKVDVIRDR	Q14011	CIRBP	Cold-inducible RNA-binding protein	44.9	110	2E-31	-	-	-	-	-
Q6PFT7	cotl1	Coactosin-like 1	_ISTDK(cr)ALVK_	98	1	RAKISTDKALVKDAV	Q14019	COTL1	Coactosin-like	71.1	218	2E-75	RAKTGTDKTLVKEVV	98	K	31.2	4E-03
E9QBV1	cltcb	Clathrin heavy chain	_AQLQK(cr)GLVK_	1118	1	LAKAQLQKGLVKEAI	Q00610	CLTC	Clathrin heavy chain 1	95.6	3334	0E+00	LAKAQLQKGMVKEAI	1118	K	45.2	7E-07
E9QBV1	cltcb	Clathrin heavy chain	_VNIPK(cr)VLR_	1347	1	WSRVNIPKVLRAAEQ	Q00610	CLTC	Clathrin heavy chain 1	95.6	3334	0E+00	WSRVNIPKVLRAAEQ	1347	K	52	2E-10
E7FF12	-	Uncharacterized protein	_TMATEK(cr)LLK_	163	1	MRTMATEKLLKAMPA	O60641	SNAP91	Clathrin coat assembly protein AP180	83.0	407	9E-138	MRTMAPEKLLKSMPI	163	K	43.1	2E-07
Q6IQV0	zgc:86598	Uncharacterized protein	_IAK(cr)VLGTEDLYDYIDK_	247	1	DQLVRIAKVLGTEDL	P68400	CSNK2A1	Casein kinase II subunit alpha	89.3	688	0E+00	DQLVRIAKVLGTEDL	247	K	49.4	1E-09
A5WWJ4	matn1	Uncharacterized protein	_AK(cr)ALGFK_	393	1	GDAAKKAKALGFKMY	P21941	MATN1	Cartilage matrix protein	71.8	704	0E+00	NDAAKKAKDLGFKMF	402	K	39.7	6E-05
A5WWJ4	matn1	Uncharacterized protein	_AALVK(cr)AVSK_	103	1	KTKAALVKAVSKIEP	P21941	MATN1	Cartilage matrix protein	71.8	704	0E+00	-	-	-	-	-
A5WWJ4	matn1	Uncharacterized protein	_VFLAK(cr)VIDGLSVGPDATR_	63	1	QVKVFLAKVIDGLSV	P21941	MATN1	Cartilage matrix protein	71.8	704	0E+00	-	-	-	-	-
Q92051	cahz	Carbonic anhydrase	_LQK(cr)VLDALDDIK_	158	1	AANPRLQKVLDALDD	P00915	CA1	Carbonic anhydrase 1	61.0	329	1E-114	EANPKLQKVLDALQA	162	K	36.7	4E-05
Q6PI52	calm1a	Calmodulin	_M(ox)K(cr)DTDSEEEIR_	78	1	TMMARKMKDTDSEEE	P62158	CALM1	Calmodulin	100.0	298	5E-107	TMMARKMKDTDSEEE	78	K	54.5	2E-11
Q6PI52	calm1a	Calmodulin	_MK(cr)DTDSEEEIR_	78	1	TMMARKMKDTDSEEE	P62158	CALM1	Calmodulin	100.0	298	5E-107	TMMARKMKDTDSEEE	78	K	54.5	2E-11
Q6TH14	-	Enolase	_IDK(cr)FMLELDGTENK_	126	1	CKAGAAEKGVPLYRH	P13929	ENO3	Beta-enolase	85.9	784	0E+00	CKAGAAEKGVPLYRH	126	K	50.3	7E-10
Q6TH14	-	Enolase	_IEEELGDK(cr)AK_	420	1	IEEELGDKAKFAGKD	P13929	ENO3	Beta-enolase	85.9	784	0E+00	IEEELGDEARFAGRK	420	E	35.4	1E-04
Q6TH14	-	Enolase	_DIAPK(cr)LIEK_	76	1	VNKDIAPKLIEKKFS	P13929	ENO3	Beta-enolase	85.9	784	0E+00	-	-	-	-	-
Q6DRD1	atp5o	ATP synthase oligomycin sensitivity conferral protein	_QK(cr)TFVDALTK_	87	1	VKRSVKQKTFVDALT	P48047	ATP5O	ATP synthase subunit O, mitochondrial	61.4	273	9E-95	-	-	-	-	-
Q5RH26	atp5c1	ATP synthase subunit gamma	_SIK(cr)NIQK_	34	1	TIRLKSIKNIQKITK	P36542	ATP5C1	ATP synthase subunit gamma, mitochondrial	77.8	479	2E-173	TRRLKSIKNIQKITK	39	K	44.8	6E-08
Q6PC77	atp5h	ATP synthase, H+ transporting, mitochondrial F0 complex, subunit d	_AAVK(cr)AIDWLAFAER_	9	1	AGRRAAVKAIDWLAF	O75947	ATP5H	ATP synthase subunit d, mitochondrial	60.2	213	2E-72	-	-	-	-	-
Q6PC77	atp5h	ATP synthase, H+ transporting, mitochondrial F0 complex, subunit d	_AVVAK(cr)AGMVDEFEK_	63	1	HYRAVVAKAGMVDEF	O75947	ATP5H	ATP synthase subunit d, mitochondrial	60.2	213	2E-72	-	-	-	-	-
A8WGC6	atp5b	ATP synthase subunit beta	_GQK(cr)VLDTGAPIR_	113	1	EGLVRGQKVLDTGAP	P06576	ATP5B	ATP synthase subunit beta, mitochondrial	89.2	927	0E+00	EGLVRGQKVLDSGAP	124	K	45.2	4E-08
A8WGC6	atp5b	ATP synthase subunit beta	_GVQK(cr)ILQDYK_	415	1	DVARGVQKILQDYKS	P06576	ATP5B	ATP synthase subunit beta, mitochondrial	89.2	927	0E+00	DVARGVQKILQDYKS	426	K	50.3	7E-10

Q08BA1	atp5a1	ATP synthase subunit alpha	_LIK(cr)EGDIVK_	125	1	FGNDKLIKEGDIVKR	P25705	ATP5A1	ATP synthase subunit alpha, mitochondrial	91.3	1010	0E+00	FGNDKLIKEGDIVKR	126	K	50.7	5E-10
Q08BA1	atp5a1	ATP synthase subunit alpha	_FNEGTEEK(cr)K_	238	1	FNEGTEEKKKLYCIY	P25705	ATP5A1	ATP synthase subunit alpha, mitochondrial	91.3	1010	0E+00	FNDGSDEKKKLYCIY	239	K	46	2E-08
Q08BA1	atp5a1	ATP synthase subunit alpha	_GHLDK(cr)MEPSK_	497	1	GVRGHLDKMEPSKIT	P25705	ATP5A1	ATP synthase subunit alpha, mitochondrial	91.3	1010	0E+00	GVRGYLDKLEPSKIT	498	K	41.8	7E-07
B8JIS1	atp5f1	Uncharacterized protein	_LNADK(cr)IAK_	127	1	ADKLNADKIAKAQEV	P24539	ATP5F1	ATP synthase F	57.7	285	2E-98	-	-	-	-	-
F1QA12	-	Uncharacterized protein (Fragment)	_LAK(cr)EQAEK_	539	1	AEKERLAKEQAEKEK	Q12797	ASPH	Aspartyl/asparaginyl beta-hydroxylase	58.0	68.2	1E-11	-	-	-	-	-
B8A5D3	nars	Uncharacterized protein	_EK(cr)NLEEAK_	101	1	EDAERREKNLEEAKK	O43776	NARS	Asparagine--tRNA ligase, cytoplasmic	81.8	973	0E+00	EDSLRREKNLEEAKK	93	K	40.9	1E-06
Q503V2	apoea	Uncharacterized protein	_FNEDLQLLVTK(cr)LR_	107	1	DLQLLVTKLRTHMEE	P02649	APOE	Apolipoprotein E	26.2	110	2E-29	-	-	-	-	-
Q503V2	apoea	Uncharacterized protein	_THMEEAK(cr)DR_	116	1	RTHMEEAKDRVTEYT	P02649	APOE	Apolipoprotein E	26.2	110	2E-29	-	-	-	-	-
E9QFX0	apoc1	Uncharacterized protein	_EIAEDLADK(cr)TK_	47	1	IAEDLADKTKTAFQN	P02654	APOC1	Apolipoprotein C-I	38.6	40	1E-06	-	-	-	-	-
E9QFX0	apoc1	Uncharacterized protein	_FGTQMK(cr)EIAEDLADK_	38	1	TKFGTQMKEIAEDLA	P02654	APOC1	Apolipoprotein C-I	38.6	40	1E-06	-	-	-	-	-
Q5TZ29	apobb.1	Uncharacterized protein	_EEIQK(cr)FLK_	2362	1	DIREEIQKFLKHASS	P04114	APOB	Apolipoprotein B-100	30.3	1487	0E+00	-	-	-	-	-
Q5TZ29	apobb.1	Uncharacterized protein	_EVLMK(cr)VIFDK_	518	1	EGREVLMKVIFDKAA	P04114	APOB	Apolipoprotein B-100	30.3	1487	0E+00	-	-	-	-	-
Q5TZ29	apobb.1	Uncharacterized protein	_SK(cr)VEEILSELK_	2104	1	EYFAIKSKVEEILSE	P04114	APOB	Apolipoprotein B-100	30.3	1487	0E+00	-	-	-	-	-
Q5TZ29	apobb.1	Uncharacterized protein	_VK(cr)VVDVVK_	2320	1	IEKLEKVKVVDVVKK	P04114	APOB	Apolipoprotein B-100	30.3	1487	0E+00	-	-	-	-	-
Q5TZ29	apobb.1	Uncharacterized protein	_VVELIK(cr)K_	2203	1	KKVVELIKKFNIEET	P04114	APOB	Apolipoprotein B-100	30.3	1487	0E+00	-	-	-	-	-
Q5TZ29	apobb.1	Uncharacterized protein	_IPALPEFTFPK(cr)K_	1208	1	LPEFTFPKKLFLNAE	P04114	APOB	Apolipoprotein B-100	30.3	1487	0E+00	-	-	-	-	-
Q5TZ29	apobb.1	Uncharacterized protein	_K(cr)LFLNAEGAAK_	1209	1	PEFTFPKKLFLNAEG	P04114	APOB	Apolipoprotein B-100	30.3	1487	0E+00	-	-	-	-	-
Q5TZ29	apobb.1	Uncharacterized protein	_AK(cr)QLLNLLGNR_	2161	1	SKILEKAKQLLNLLG	P04114	APOB	Apolipoprotein B-100	30.3	1487	0E+00	-	-	-	-	-
Q5TZ29	apobb.1	Uncharacterized protein	_QFVANFDQEK(cr)FTEDVK_	2123	1	VANFDQEKFTEDVKN	P04114	APOB	Apolipoprotein B-100	30.3	1487	0E+00	-	-	-	-	-
Q5TZ29	apobb.1	Uncharacterized protein	_VVDVVK(cr)K_	2326	1	VKVVDVVKKFSDIVD	P04114	APOB	Apolipoprotein B-100	30.3	1487	0E+00	-	-	-	-	-
O42363	apoa1	Apolipoprotein A-I	_AFESNIEETK(cr)SK_	180	1	ESNIEETKSKVVPMV	P02647	APOA1	Apolipoprotein A-I	25.4	95.9	3E-24	-	-	-	-	-
O42363	apoa1	Apolipoprotein A-I	_EQLVK(cr)AVEEAR_	218	1	EYKEQLVKAVEEARE	P02647	APOA1	Apolipoprotein A-I	25.4	95.9	3E-24	-	-	-	-	-
O42363	apoa1	Apolipoprotein A-I	_AAALVYLNQVK(cr)DQAEK_	44	1	LVYLNQVKDQAEKAL	P02647	APOA1	Apolipoprotein A-I	25.4	95.9	3E-24	-	-	-	-	-
O42363	apoa1	Apolipoprotein A-I	_SK(cr)VVPMVEAVR_	182	1	NIEETKSKVVPMVEA	P02647	APOA1	Apolipoprotein A-I	25.4	95.9	3E-24	-	-	-	-	-
E7FES0	-	Uncharacterized protein	_ELTELGEQVK(cr)PHFEGIFK_	240	1	TELGEQVKPHFEGIF	P02647	APOA1	Apolipoprotein A-I	29.0	84.3	3E-19	-	-	-	-	-
E7FES0	-	Uncharacterized protein	_K(cr)ELTELGEQVKPHFEGIFK_	230	1	VKSGELRKELTELGE	P02647	APOA1	Apolipoprotein A-I	29.0	84.3	3E-19	-	-	-	-	-
Q804G7	anxa4	Annexin	_AEIDMLDIK(cr)AEFLK_	286	1	EIDMLDIKAEFLKMY	P09525	ANXA4	Annexin A4	64.5	428	1E-152	EIDMLDIRAHFKRLY	284	R	33.3	7E-04
A2BFV8	anxa1a	Annexin	_EIGDIK(cr)QVYK_	148	1	NKEIGDIKQVYKQEY	P04083	ANXA1	Annexin A1	62.6	276	7E-94	-	-	-	-	-
B8A4A7	sncga	Gamma-synuclein	_TK(cr)AGVEEAAAK_	23	1	VAAAEKTKAGVEEAA	P37840	SNCA	Alpha-synuclein	58.0	95.9	7E-27	VAAAEKTKQGVAEAA	23	K	37.5	2E-05
F1R8P5	-	Uncharacterized protein	_TK(cr)AGVEEAALK_	23	1	VAAAEKTKAGVEEAA	P37840	SNCA	Alpha-synuclein	64.0	104	3E-30	VAAAEKTKQGVAEAA	23	K	37.5	2E-05
E9QJI6	eno3	Enolase	_(ac)SISK(cr)IHAR_	5	1	__MSISKIHAREILD	P06733	ENO1	Alpha-enolase	84.5	491	1E-176	__MSILKIHAREIFD	6	I	35.4	9E-05
Q6PC12	eno1a	Enolase 1, (Alpha)	_GGK(cr)YDLDFK_	256	1	SEFYKGGKYDLDFKS	P06733	ENO1	Alpha-enolase	90.5	823	0E+00	SEFFRSGKYDLDFKS	256	K	41.8	7E-07
Q6PC12	eno1a	Enolase 1, (Alpha)	_IEEELGDK(cr)AR_	420	1	IEEELGDKARFAGKN	P06733	ENO1	Alpha-enolase	90.5	823	0E+00	IEEELGSKAKFAGRN	420	K	38.4	1E-05
D1GJ56	actn3a	Actn3a	_FLDIPK(cr)MLDADDIVNTPKPDEK_	229	1	EKFLDIPKMLDADDI	Q08043	ACTN3	Alpha-actinin-3	83.9	1570	0E+00	EKYLDIPKMLDAEDI	234	K	47.7	6E-09
Q8AX99	actn3b	Actn3b	_YLDIPK(cr)MLDAEDIVNTPKPDEK_	231	1	EKYLDIPKMLDAEDI	P35609	ACTN2	Alpha-actinin-2	83.7	1587	0E+00	EKHLDIPKMLDAEDI	227	K	48.1	4E-09
Q8AX99	actn3b	Actn3b	_K(cr)HEAFESDLAAHQDR_	447	1	EIRALMRKHEAFESD	P35609	ACTN2	Alpha-actinin-2	83.7	1587	0E+00	EVRALLRKHEAFESD	443	K	46	2E-08
D1GJ56	actn3a	Actn3a	_K(cr)HEAFESDLAAHQDR_	445	1	EIRALMRKHEAFESD	P35609	ACTN2	Alpha-actinin-2	83.7	1587	0E+00	EVRALLRKHEAFESD	443	K	46	2E-08

F1QZU7	aldh2.2	Uncharacterized protein (Fragment)	_ADVDK(cr)AVK_	77	1	GDKADVDKAVKAARD	P05091	ALDH2	Aldehyde dehydrogenase, mitochondrial	76.6	852	0E+00	GDKEDVDKAVKAARA	78	K	40.9	2E-05
Q8JHI0	slc25a5	Solute carrier family 25 alpha, member 5	_DFLAGGIAAAISK(cr)TAVAPIER_	23	1	GIAAAISKTAVAPIE	P05141	SLC25A5	ADP/ATP translocase 2	92.9	574	0E+00	GVAAAISKTAVAPIE	23	K	44.3	9E-08
Q8JHI0	slc25a5	Solute carrier family 25 alpha, member 5	_K(cr)VFLDGVDK_	97	1	AFKDKYKKVFLDGVD	P05141	SLC25A5	ADP/ATP translocase 2	92.9	574	0E+00	AFKDKYKQIFLGGVD	97	Q	40.1	3E-06
Q8JHI0	slc25a5	Solute carrier family 25 alpha, member 5	_LAADVGK(cr)AGAER_	147	1	RLAADVGKAGAEREF	P05141	SLC25A5	ADP/ATP translocase 2	92.9	574	0E+00	RLAADVGKAGAEREF	147	K	48.1	4E-09
Q8JHI0	slc25a5	Solute carrier family 25 alpha, member 5	_DEGGK(cr)AFFK_	268	1	IARDEGGKAFFKGAW	P05141	SLC25A5	ADP/ATP translocase 2	92.9	574	0E+00	IARDEGGKAFFKGAW	268	K	51.1	4E-10
Q68EH2	ak1	Adenylate kinase isoenzyme 1	_ADVSK(cr)GYLIDGYPR_	88	1	IAKADVSKGYLIDGY	P00568	AK1	Adenylate kinase isoenzyme 1	73.7	305	6E-108	VAKVNTSKGFLIDGY	88	K	33.7	5E-04
Q68EH2	ak1	Adenylate kinase isoenzyme 1	_DAMIAK(cr)ADVSK_	83	1	IKDAMIAKADVSKGY	P00568	AK1	Adenylate kinase isoenzyme 1	73.7	305	6E-108	-	-	-	-	-
Q6P5L2	dbi	Uncharacterized protein	_AAEEVK(cr)QLK_	14	1	QKAAEEVKQLKAKPT	P07108	DBI	Acyl-CoA-binding protein	79.3	142	5E-47	EKAAEEVRHLKTKPS	14	R	33.7	5E-04
Q6P5L2	dbi	Uncharacterized protein	_AYIAK(cr)VEELK_	77	1	AVKAYIAKVEELKGK	P07108	DBI	Acyl-CoA-binding protein	79.3	142	5E-47	AMKAYINKVEELKKK	77	K	35.4	1E-04
Q7ZVI7	actba	Actin, cytoplasmic 1	_M(ox)QK(cr)EITSLAPSTMK_	315	1	GIADRMQKEITSLAP	P62736	ACTA2	Actin, aortic smooth muscle	94.0	744	0E+00	GIADRMQKEITALAP	317	K	48.1	4E-09
Q9I8V1	actc1b	Actin, alpha 1, skeletal muscle	_DLTDYLMK(cr)ILTER_	193	1	DLTDYLMKILTERGY	P68032	ACTC1	Actin, alpha cardiac muscle 1	98.9	785	0E+00	DLTDYLMKILTERGY	193	K	53.7	4E-11
Q9I8V1	actc1b	Actin, alpha 1, skeletal muscle	_MQK(cr)EITALAPSTMK_	317	1	GIADRMQKEITALAP	P68032	ACTC1	Actin, alpha cardiac muscle 1	98.9	785	0E+00	GIADRMQKEITALAP	317	K	50.7	5E-10
Q7ZVI7	actba	Actin, cytoplasmic 1	_MQK(cr)EITSLAPSTMK_	315	1	GIADRMQKEITSLAP	P68032	ACTC1	Actin, alpha cardiac muscle 1	94.0	746	0E+00	GIADRMQKEITALAP	317	K	48.1	4E-09
Q9I8V1	actc1b	Actin, alpha 1, skeletal muscle	_IK(cr)IIAPPER_	330	1	APSTMKIKIIAPPER	P68032	ACTC1	Actin, alpha cardiac muscle 1	98.9	785	0E+00	APSTMKIKIIAPPER	330	K	51.5	3E-10
Q6NUW5	anp32e	Acidic leucine-rich nuclear phosphoprotein 32 family member E	_ELSTLEALQNLK(cr)NLK_	113	1	LEALQNLKNLKSLDL	Q9BTT0	ANP32E	Acidic leucine-rich nuclear phosphoprotein 32 family member E	82.4	248	2E-83	VEALQNLKNLKSLDL	115	K	46	2E-08
Q6AZA0	acat1	Acetyl-CoA acetyltransferase, mitochondrial	_GAIDK(cr)AGIPVEEVK_	71	1	AIKGAIDKAGIPVEE	P24752	ACAT1	Acetyl-CoA acetyltransferase, mitochondrial	78.0	681	0E+00	AIQGAIEKAGIPKEE	78	K	35	2E-04
Q6AZA0	acat1	Acetyl-CoA acetyltransferase, mitochondrial	_LK(cr)AVFLK_	261	1	FSKVPKLKAVFLKEN	P24752	ACAT1	Acetyl-CoA acetyltransferase, mitochondrial	78.0	681	0E+00	FSKVPKLKTVFQKEN	268	K	41.8	7E-07
Q6P3L3	hspa5	Heat shock protein 5	_AVEEK(cr)IEWLEAHQDADLEEFQAK_	599	1	IEKAVEEKIEWLEAH	P11021	HSPA5	78 kDa glucose-regulated protein	93.4	1214	0E+00	MEKAVEEKIEWLESH	603	K	47.3	1E-07
Q6PBZ1	rpl7a	OTTDARP00000018222	_AALAK(cr)LVEAIK_	217	1	EDKAALAKLVEAIKT	P62424	RPL7A	60S ribosomal protein L7a	92.4	479	1E-174	EDKGALAKLVEAIRT	217	K	41.4	2E-05
Q6PBZ1	rpl7a	OTTDARP00000018222	_AK(cr)ELATK_	259	1	KLEKAKAKELATKLG	P62424	RPL7A	60S ribosomal protein L7a	92.4	479	1E-174	KLEKAKAKELATKLG	259	K	46.9	2E-07
Q7ZW95	rpl4	Ribosomal protein L4	_SEEVQK(cr)AIR_	300	1	IKSEEVQKAIRPANR	P36578	RPL4	60S ribosomal protein L4	85.3	588	0E+00	-	-	-	-	-
Q6DGL9	rpl38	Ribosomal protein L38	_QSLPPGLAVK(cr)ELK_	67	1	LPPGLAVKELK____	P63173	RPL38	60S ribosomal protein L38	100.0	137	2E-45	LPPGLAVKELK____	67	K	36.3	3E-05
Q7ZWJ7	rpl34	60S ribosomal protein L34	_IVVK(cr)VLK_	105	1	EEQKIVVKVLKAQAQ	P49207	RPL34	60S ribosomal protein L34	94.0	226	2E-79	EEQKIVVKVLKAQAQ	105	K	49.4	1E-09
Q7ZV82	rpl27	60S ribosomal protein L27	_TVVNK(cr)DVFR_	98	1	LDKTVVNKDVFRDPA	P61353	RPL27	60S ribosomal protein L27	95.6	266	2E-94	LDKTVVNKDVFRDPA	98	K	50.7	5E-10
Q6P5L3	rpl19	60S ribosomal protein L19	_ILMEHIHK(cr)LK_	144	1	ILMEHIHKLKADKAR	P84098	RPL19	60S ribosomal protein L19	93.9	340	4E-122	ILMEHIHKLKADKAR	144	K	52.4	1E-10
Q6P5L3	rpl19	60S ribosomal protein L19	_TLSK(cr)EDETK_	190	1	EIIKTLSKEDETKK_	P84098	RPL19	60S ribosomal protein L19	94.0	341	3E-121	EIIKTLSKEEETKK_	190	K	44.3	7E-08
E9QF69	rpl18	60S ribosomal protein L18	_IQNIPK(cr)LK_	97	1	VRIQNIPKLKVCALK	Q07020	RPL18	60S ribosomal protein L18	84.8	291	3E-103	VRVQEVPKLKVCALR	97	K	38.8	8E-06
E9QF69	rpl18	60S ribosomal protein L18	_SDAPFNK(cr)VILR_	45	1	RSDAPFNKVILRRLF	Q07020	RPL18	60S ribosomal protein L18	84.8	291	3E-103	-	-	-	-	-
Q1LYB7	rpl13a	OTTDARP00000009674	_MVVPAALK(cr)IVR_	127	1	MVVPAALKIVRLKPT	P40429	RPL13A	60S ribosomal protein L13a	87.9	360	2E-129	MVVPAALKVVRLKPT	125	K	47.7	6E-09
Q1LYB7	rpl13a	OTTDARP00000009674	_NVESK(cr)IAVYTDVLK_	190	1	AEKNVESKIAVYTDV	P40429	RPL13A	60S ribosomal protein L13a	87.9	360	2E-129	AEKNVEKKIDKYTEV	188	K	32.5	1E-03

Q1LYB7	rpl13a	OTTDARP00000009674	_FNK(cr)VLIIDGR_	7	1	MADRFNKVLIIDGRG	P40429	RPL13A	60S ribosomal protein L13a	87.9	360	2E-129	-	-	-	-	-
Q90Z10	rpl13	60S ribosomal protein L13	_TK(cr)LIIFPR_	123	1	RLKEYRTKLIIFPRK	P26373	RPL13	60S ribosomal protein L13	86.7	363	2E-130	RLKEYRSKLILFPRK	123	K	46	2E-08
Q90Z10	rpl13	60S ribosomal protein L13	_EAAEQDVEK(cr)K_	209	1	AAEQDVEKKK_____	P26373	RPL13	60S ribosomal protein L13	86.7	363	2E-130	AAEQDVEKKK_____	209	K	33.7	2E-04
F8W4I2	rplp0	60S acidic ribosomal protein P0	_GK(cr)AVVLMGK_	50	1	IRLSLRGKAVVLMGK	P05388	RPLP0	60S acidic ribosomal protein P0	96.8	319	2E-112	IRMSLRGKAVVLMGK	50	K	46.4	2E-08
A3KQ06	rps9	Uncharacterized protein	_MK(cr)LDYILGLK_	93	1	VLDEGKMKLDYILGL	P46781	RPS9	40S ribosomal protein S9	95.6	264	8E-93	VLDEGKMKLDYILGL	93	K	51.5	3E-10
Q6DHL6	rps6	40S ribosomal protein S6	_LFNLSK(cr)EDDVR_	149	1	RKLFNLSKEDDVRQY	P62753	RPS6	40S ribosomal protein S6	95.6	482	2E-176	RKLFNLSKEDDVRQY	149	K	52.4	1E-10
Q6PBI5	rps25	40S ribosomal protein S25	_ATYDK(cr)LYK_	56	1	FDKATYDKLYKEVPN	P62851	RPS25	40S ribosomal protein S25	91.6	159	1E-52	FDKATYDKLCKEVPN	57	K	46.9	1E-08
Q6PBI5	rps25	40S ribosomal protein S25	_AALQELLGK(cr)GLIK_	93	1	ALQELLGKGLIKLVS	P62851	RPS25	40S ribosomal protein S25	91.6	159	1E-52	ALQELLSKGLIKLVS	94	K	43.9	2E-06
Q6PBI5	rps25	40S ribosomal protein S25	_GLIK(cr)LVSK_	97	1	LLGKGLIKLVSKHRA	P62851	RPS25	40S ribosomal protein S25	91.6	159	1E-52	LLSKGLIKLVSKHRA	98	K	44.3	9E-08
B7ZD32	rps24	40S ribosomal protein S24	_ATVPK(cr)TEIR_	25	1	PGKATVPKTEIREKL	P62847	RPS24	40S ribosomal protein S24	89.8	195	1E-66	PGKATVPKTEIREKL	37	K	49.4	2E-08
A8KB78	rps23	Uncharacterized protein	_WHDK(cr)QYK_	25	1	REQKWHDKQYKKAHL	P62266	RPS23	40S ribosomal protein S23	98.6	286	2E-102	RDQKWHDKQYKKAHL	25	K	52.4	1E-10
A8KB78	rps23	Uncharacterized protein	_ANPFGGASHAK(cr)GIVLEK_	48	1	FGGASHAKGIVLEKV	P62266	RPS23	40S ribosomal protein S23	98.6	286	2E-102	FGGASHAKGIVLEKV	48	K	47.7	9E-08
A8KB78	rps23	Uncharacterized protein	_GIVLEK(cr)VGVEAK_	54	1	AKGIVLEKVGVEAKQ	P62266	RPS23	40S ribosomal protein S23	98.6	286	2E-102	AKGIVLEKVGVEAKQ	54	K	47.7	6E-09
E9QDR0	rps2	Uncharacterized protein	_IK(cr)SLEEIYLYSLPIK_	62	1	LVKDMKIKSLEEIYL	P15880	RPS2	40S ribosomal protein S2	94.1	138	2E-42	LVKDMKIKSLEEIYL	76	K	52.4	1E-10
Q6PBW7	rps19	Ribosomal protein S19	_PGGVTVK(cr)DVNQQEFVR_	8	1	MPGGVTVKDVNQQEF	P39019	RPS19	40S ribosomal protein S19	88.3	260	9E-92	MPG-VTVKDVNQQEF	7	K	45.2	5E-08
Q6PBW7	rps19	Ribosomal protein S19	_LK(cr)VPDWVDIVK_	30	1	LKKSGKLKVPDWVDI	P39019	RPS19	40S ribosomal protein S19	88.3	260	9E-92	LKKSGKLKVPEWVDT	29	K	45.2	5E-08
Q6PBW7	rps19	Ribosomal protein S19	_VPDWVDIVK(cr)LAK_	39	1	PDWVDIVKLAKHKEL	P39019	RPS19	40S ribosomal protein S19	88.3	260	9E-92	PEWVDTVKLAKHKEL	38	K	46	2E-08
F8W246	rps13	Uncharacterized protein	_FVTGNK(cr)ILR_	70	1	VRFVTGNKILRILKS	P62277	RPS13	40S ribosomal protein S13	99.1	217	3E-75	VRFVTGNKILRILKS	70	K	49.8	1E-09
Q6PC90	rps12	40S ribosomal protein S12	_EAAK(cr)ALDK_	40	1	RGIREAAKALDKRQA	P25398	RPS12	40S ribosomal protein S12	97.0	258	1E-91	RGIREAAKALDKRQA	40	K	48.6	3E-09
Q7ZV05	rps11	Ribosomal protein S11	_EAIDGTYIDK(cr)K_	59	1	IDGTYIDKKCPFTGN	P62280	RPS11	40S ribosomal protein S11	91.2	300	3E-107	IEGTYIDKKCPFTGN	58	K	50.3	7E-10
Q803M8	ywhaqb	Tryosine 3-monooxygenase/tryptophan 5-monooxygenase activation protein, zeta polypeptide	_VFYLK(cr)MK_	120	1	ESKVFYLKMKGDYYR	P63104	YWHAZ	14-3-3 protein zeta/delta	84.1	432	1E-156	ESKVFYLKMKGDYYR	120	K	54.5	2E-11
E9QJ96	ywhaba	14-3-3 protein beta/alpha-A	_VFYLK(cr)MK_	120	1	ESKVFYLKMKGDYYR	P63104	YWHAZ	14-3-3 protein zeta/delta	88.0	413	1E-148	ESKVFYLKMKGDYYR	120	K	54.5	2E-11
Q803M8	ywhaqb	Tryosine 3-monooxygenase/tryptophan 5-monooxygenase activation protein, zeta polypeptide	_AFDISK(cr)TEMQPTHPIR_	157	1	QKAFDISKTEMQPTH	P63104	YWHAZ	14-3-3 protein zeta/delta	84.1	432	1E-156	QEAFEISKKEMQPTH	157	K	41.4	2E-05
E9QJ96	ywhaba	14-3-3 protein beta/alpha-A	_AYQDAFEISK(cr)K_	157	1	QDAFEISKKEMQPTH	P31946	YWHAB	14-3-3 protein beta/alpha	90.7	422	5E-153	QEAFEISKKEMQPTH	159	K	50.7	5E-10
F1R2S5	vtg5	Uncharacterized protein (Fragment)	_IVTTYAK(cr)K_	1277	1	KIVTTYAKKVCKHIL	-	-	-	-	-	-	-	-	-	-	-
Q1LWN2	vtg1	Uncharacterized protein	_LPIIVTTYAK(cr)K_	1274	1	IIVTTYAKKLGKHIL	-	-	-	-	-	-	-	-	-	-	-
A3KPI6	si:ch211-288g17.3	Uncharacterized protein	_IK(cr)VVVVK_	247	1	PGTGRKIKVVVVKKV	-	-	-	-	-	-	-	-	-	-	-
E9QEQ1	apoc2	Uncharacterized protein	_AK(cr)TIYSDTVR_	69	1	YKLEEKAKTIYSDTV	-	-	-	-	-	-	-	-	-	-	-
B3DFP9	apoa2	Uncharacterized protein	_IAGGLASELEPVVDK(cr)AR_	101	1	ELEPVVDKARLNALG	-	-	-	-	-	-	-	-	-	-	-

B3DFP9	apoa2	Uncharacterized protein	_DVVEELK(cr)K_	76	1	KDVVEELKKSPRVES	-	-	-	-	-	-	-	-	-	-	-
B3DFP9	apoa2	Uncharacterized protein	_ELVDK(cr)YDGLR_	32	1	PDKELVDKYDGLRTV	-	-	-	-	-	-	-	-	-	-	-
